# Supplementary material for: Catalytic enantioselective nitrone cycloadditions enabling collective syntheses of indole alkaloids
Source: Nat Commun. 2024 Jul 31;15:6429. doi: 10.1038/s41467-024-50509-4 (PMC11289135; doi:10.1038/s41467-024-50509-4)
Supplement: Supplementary file 1 — Supplementary Information [file 41467_2024_50509_MOESM1_ESM.pdf]

Supplementary Information

Catalytic Enantioselective Nitrone Cycloadditions Enabling Collective Syntheses of Indole Alkaloids

Xiaochen Tian,[1,2,4] Tengfei Xuan,[1,2,4] Jingkun Gao,[3,4] Xinyu Zhang,[1] Tao Liu,[1] Fengbiao Luo,[1] Ruochen Pang,[1] Pengcheng Shao,[1] Yun-Fang Yang,[3]* and Yang Wang[1,2]*

[1]Molecular Synthesis Center & Key Laboratory of Marine Drugs, Chinese Ministry of Education, School of Medicine and Pharmacy, Ocean University of China, 5 Yushan Road, Qingdao 266003, China

[2]Laboratory for Marine Drugs and Bioproducts, Qingdao Marine Science and Technology Center, Qingdao 266237, China

[3]State Key Laboratory Breeding Base of Green Chemistry-Synthesis Technology, Key Laboratory of Green Chemistry-Synthesis Technology of Zhejiang Province, College of Chemical Engineering, Zhejiang University of Technology, Hangzhou, Zhejiang 310014, China

[4]These authors contributed equally

*Correspondence: yangyf@zjut.edu.cn, wangyang@ouc.edu.cn

Content

1. Proposed transition states of stereoselective alkylation......S2

2. Experimental procedures and characterization data......S2

2.1 Synthesis of nitrones......S2

2.2 Synthesis of **3**......S9

2.3 Synthesis of **5**......S14

2.4 Synthesis of **7**......S21

2.5 Procedures for further synthetic transformation......S24

2.6 Synthesis of (−)-harmicine......S27

2.7 Synthesis of (−)-desbromoarborescidine A......S30

2.8 Synthesis of (−)-arboricine and corynanthe family alkaloids......S33

2.9 Synthesis of yohimbine family alkaloids......49

2.10 Synthesis of (+)-arborescidine B and (+)-arborescidine C......S59

2.11 Synthesis of eburnamine family alkaloids......S63

2.12 Synthesis of (−)-Arbornamine......S86

3. Crystallographic data......S92

4. Computational details......S100

5. Copies of NMR spectra and HPLC spectra......S106

6. Supplementary references......S306

1. Proposed transition states of stereoselective alkylation

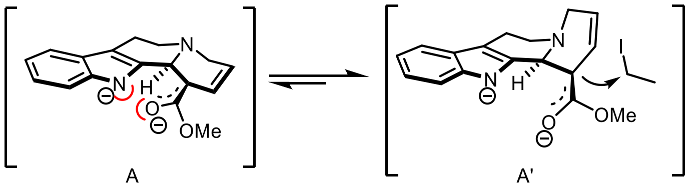

A A'

Supplementary Figure 1 Proposed transition states of stereoselective alkylation

2. Experimental procedures and characterization data

2.1 Synthesis of nitrones

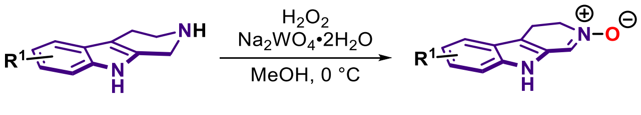

S1a-S1w **1a-1w**

Typical Procedure: S1a-S1w were prepared according to a reported procedure[1]. To a stirred solution of **S1** (1.0 equiv) and $Na_2WO_4·2H_2O$ (0.12 equiv) in MeOH (0.1 M) was dropwise added H_2O_2 (30% wt, 3.35 equiv) at 0 °C. The reaction was stirred at 0 °C for 2-12 h. Then the reaction mixture was added H_2O and extracted with DCM. The combined organic layers were washed with brine, dried over Na_2SO_4, filtered and concentrated *in vacuo*. The residue was purified by column chromatography on silica gel (EtOAc: MeOH, 20:1) to give **1a-1w**.

4,9-dihydro-3*H*-pyrido[3,4-*b*]indole 2-oxide (1a)

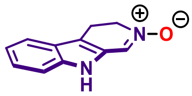

Yield: 51%; Yellow solid; ^{1}H NMR (400 MHz, DMSO-d$_6$): δ 11.15 (s, 1H), 7.78 (s, 1H), 7.48 (d, J = 7.9 Hz, 1H), 7.37 (d, J = 8.1 Hz, 1H), 7.12-7.08 (m, 1H), 7.05-7.01 (m, 1H), 4.13 (t, J = 8.4 Hz, 2H), 3.15 (t, J = 8.5 Hz, 2H); ^{13}C NMR (101 MHz, DMSO-d$_6$): δ 137.6, 128.8, 125.3, 125.1, 122.3, 119.8, 118.6, 111.9, 106.0, 60.2, 19.6.

5-fluoro-4,9-dihydro-3*H*-pyrido[3,4-*b*]indole 2-oxide (1b)

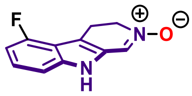

Yield: 41%; Yellow solid; ^{1}H NMR (400 MHz, DMSO-d$_6$): δ 11.45 (s, 1H), 7.79 (s, 1H), 7.21 (d, J = 8.2 Hz, 1H), 7.08 (td, J = 8.0, 5.4 Hz, 1H), 6.80 (dd, J = 11.0, 7.8 Hz, 1H), 4.14 (t, J = 8.4 Hz, 2H), 3.26 (t, J = 8.5 Hz, 2H); ^{13}C NMR (101 MHz, DMSO-d$_6$): δ 157.1 (d, J_{C-F} = 245.4 Hz), 140.0 (d, J_{C-F} = 11.1 Hz),

129.1, 124.9, 122.8 (d, $J_{C\text{-}F}$ = 7.6 Hz), 114.3 (d, $J_{C\text{-}F}$ = 21.2 Hz), 108.5 (d, $J_{C\text{-}F}$ = 3.4 Hz), 104.6 (d, $J_{C\text{-}F}$ = 18.3 Hz), 103.3 (d, $J_{C\text{-}F}$ = 1.6 Hz), 60.2, 20.5.

5-chloro-4,9-dihydro-3*H*-pyrido[3,4-*b*]indole 2-oxide (1c)

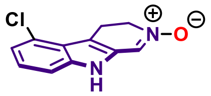

Yield: 52%; Yellow solid; ^{1}H NMR (400 MHz, DMSO-d$_6$): δ 11.52 (s, 1H), 7.79 (s, 1H), 7.35 (dd, J = 7.4, 1.5 Hz, 1H), 7.08-7.02 (m, 2H), 4.15 (t, J = 8.4 Hz, 2H), 3.41 (t, J = 8.5 Hz, 2H); ^{13}C NMR (101 MHz, DMSO-d$_6$): δ 138.6, 130.0, 124.9, 124.4, 123.0, 122.6, 120.0, 111.1, 105.1, 60.2, 20.8.

5-bromo-4,9-dihydro-3*H*-pyrido[3,4-*b*]indole 2-oxide (1d)

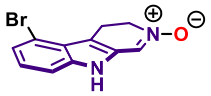

Yield: 46%; Yellow solid; ^{1}H NMR (400 MHz, DMSO-d$_6$): δ 11.51 (s, 1H), 7.78 (s, 1H), 7.39 (d, J = 7.8 Hz, 1H), 7.21 (d, J = 7.2 Hz, 1H), 7.02 (t, J = 7.9 Hz, 1H), 4.14 (t, J = 8.4 Hz, 2H), 3.46 (t, J = 8.4 Hz, 2H); ^{13}C NMR (101 MHz, DMSO-d$_6$): δ 138.4, 130.2, 124.9, 124.0, 123.3 (2C), 122.7, 111.6, 105.9, 60.0, 20.9.

5-methyl-4,9-dihydro-3*H*-pyrido[3,4-*b*]indole 2-oxide (1e)

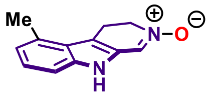

Yield: 43%; Yellow solid; ^{1}H NMR (400 MHz, DMSO-d$_6$): δ 11.13 (s, 1H), 7.74 (s, 1H), 7.18 (d, J = 8.2 Hz, 1H), 6.98 (t, J=7.2Hz, 1H), 6.75 (d, J = 7.1 Hz, 1H), 4.11 (t, J = 8.4 Hz, 2H), 3.37 (m, 2H), 2.53 (s, 1H); ^{13}C NMR (101 MHz, DMSO-d$_6$): δ 137.6, 129.6, 128.3, 125.3, 124.4, 122.5, 120.6, 109.7, 106.5, 60.0, 21.5, 19.3.

5-methoxy-4,9-dihydro-3*H*-pyrido[3,4-*b*]indole 2-oxide (1f)

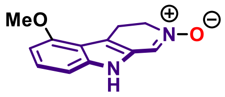

Yield: 45%; Yellow solid; ^{1}H NMR (400 MHz, DMSO-d$_6$): δ 11.15 (s, 1H), 7.72 (s, 1H), 7.03 (t, J = 8.0 Hz, 1H), 6.95 (d, J = 8.0 Hz, 1H), 6.49 (d, J = 7.6 Hz, 1H), 4.08 (t, J = 8.5 Hz, 2H), 3.83 (s, 3H), 3.27 (t, J = 8.5 Hz, 2H); ^{13}C NMR (101 MHz, DMSO-d$_6$): δ 153.7, 138.8, 127.4, 125.2, 123.5, 115.7, 105.8, 105.2, 99.8, 60.2, 55.1, 21.4.

6-fluoro-4,9-dihydro-3*H*-pyrido[3,4-*b*]indole 2-oxide (1g)

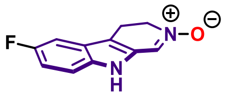

Yield: 27%; Yellow solid; ^{1}H NMR (400 MHz, DMSO-d$_6$): δ 11.24 (s, 1H), 7.79 (s, 1H), 7.37 (dd, J = 8.9, 4.6 Hz, 1H), 7.28 (dd, J = 9.8, 2.4 Hz, 1H), 6.96 (td, J = 9.3, 2.5 Hz, 1H), 4.13 (t, J = 8.3 Hz, 2H), 3.13 (t, J = 8.5 Hz, 2H); ^{13}C NMR (101 MHz, DMSO-d$_6$): δ 158.5 (d, $J_{C\text{-}F}$ = 233.7 Hz), 134.2, 130.7, 125.4 (d, $J_{C\text{-}F}$ = 10.4 Hz), 125.1, 113.0 (d, $J_{C\text{-}F}$ = 10.0 Hz), 110.5 (d, $J_{C\text{-}F}$ = 26.6 Hz), 106.1 (d, $J_{C\text{-}F}$ = 5.3 Hz), 103.3 (d, $J_{C\text{-}F}$ = 23.6 Hz), 60.3, 19.5.

6-chloro-4,9-dihydro-3*H*-pyrido[3,4-*b*]indole 2-oxide (1h)

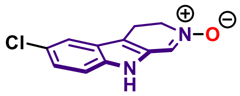

Yield: 34%; Yellow solid; ^{1}H NMR (400 MHz, DMSO-d$_6$): δ 11.35 (s, 1H), 7.79 (s, 1H), 7.56 (d, J = 1.8 Hz, 1H), 7.39 (d, J = 8.7 Hz, 1H), 7.09 (dd, J = 8.6, 2.0 Hz, 1H), 4.13 (t, J = 8.5 Hz, 2H), 3.14 (t, J = 8.5 Hz, 2H); ^{13}C NMR (101 MHz, DMSO-d$_6$): δ 136.0, 130.4, 126.2, 125.0, 124.4, 122.0, 117.8, 113.4, 105.6, 60.3, 19.4.

6-bromo-4,9-dihydro-3*H*-pyrido[3,4-*b*]indole 2-oxide (1i)

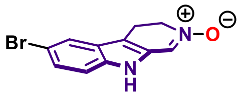

Yield: 29%; Yellow solid; ^{1}H NMR (400 MHz, DMSO-d$_6$): δ 11.36 (s, 1H), 7.80 (s, 1H), 7.70 (d, J = 1.8 Hz, 1H), 7.34 (d, J = 8.6 Hz, 1H), 7.20 (dd, J = 8.6, 1.9 Hz, 1H), 4.13 (t, J = 8.2 Hz, 2H), 3.14 (t, J = 8.4 Hz, 2H); ^{13}C NMR (101 MHz, DMSO-d$_6$): δ 136.2, 130.2, 126.9, 125.0, 124.5, 120.8, 113.8, 112.3, 105.4, 60.3, 19.4.

6-methyl-4,9-dihydro-3*H*-pyrido[3,4-*b*]indole 2-oxide (1j)

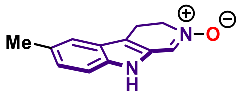

Yield: 34%; Yellow solid; ^{1}H NMR (400 MHz, DMSO-d$_6$): δ 11.00 (s, 1H), 7.75 (s, 1H), 7.26-7.24 (m, 2H), 6.94-6.92 (m, 1H), 4.11 (t, J = 8.4 Hz, 2H), 3.11 (t, J = 8.5 Hz, 2H), 2.35 (s, 3H); ^{13}C NMR (101 MHz, DMSO-d$_6$): δ 136.0, 128.8, 128.4, 125.4, 125.3, 124.0, 118.1, 111.6, 105.5, 60.2, 21.2, 19.6.

6-methoxy-4,9-dihydro-3*H*-pyrido[3,4-*b*]indole 2-oxide (1k)

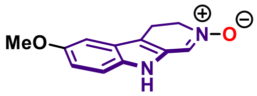

Yield: 31%; Yellow solid; ^{1}H NMR (400 MHz, DMSO-d$_6$): δ 10.99 (s, 1H), 7.74 (s, 1H), 7.26 (d, J = 8.8 Hz, 1H), 6.96 (d, J = 2.3Hz, 1H), 6.75 (dd, J = 8.8, 2.4 Hz, 1H), 4.11 (t, J = 8.4 Hz, 2H), 3.75 (s, 3H), 3.12 (t, J=8.4 Hz, 2H); ^{13}C NMR (101 MHz, DMSO-d$_6$): δ 153.9, 132.8, 129.3, 125.5, 125.3, 112.8, 112.7, 105.8, 99.8, 60.2, 55.3, 19.7.

7-fluoro-4,9-dihydro-3*H*-pyrido[3,4-*b*]indole 2-oxide (1l)

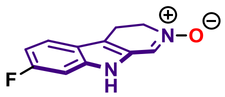

Yield: 38%; Yellow solid; ^{1}H NMR (400 MHz, DMSO-d$_6$): δ 11.23 (s, 1H), 7.77 (s, 1H), 7.50 (dd, J = 8.7, 5.6 Hz, 1H), 7.19 (dd, J = 10.1, 2.2 Hz, 1H), 6.92-6.87 (m, 1H), 4.12 (t, J = 8.8 Hz, 2H), 3.14 (t, J = 8.5 Hz, 2H); ^{13}C NMR (101 MHz, DMSO-d$_6$): δ 160.4 (d, $J_{C\text{-}F}$ = 237.2 Hz), 137.6 (d, $J_{C\text{-}F}$ = 13.0 Hz), 129.5 (d, $J_{C\text{-}F}$ = 3.8 Hz), 125.1, 122.1, 119.7 (d, $J_{C\text{-}F}$ = 10.2 Hz), 108.5 (d, $J_{C\text{-}F}$ = 24.8 Hz), 106.1 (d, $J_{C\text{-}F}$ = 1.0 Hz), 98.1 (d, $J_{C\text{-}F}$ = 26.3 Hz), 60.1, 19.5.

7-chloro-4,9-dihydro-3*H*-pyrido[3,4-*b*]indole 2-oxide (1m)

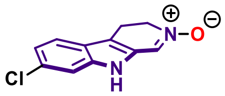

Yield: 20%; Yellow solid; ^{1}H NMR (400 MHz, DMSO-d$_6$): δ 11.27 (s, 1H), 7.80 (s, 1H), 7.49 (d, J = 8.5 Hz, 1H), 7.43 (d, J = 1.6 Hz, 1H), 7.05 (dd, J = 8.5, 1.9 Hz, 1H), 4.13 (t, J = 8.4 Hz, 2H), 3.15 (t, J = 8.5 Hz, 2H); ^{13}C NMR (101 MHz, DMSO-d$_6$): δ 137.8, 129.9, 126.6, 125.1, 123.9, 120.1, 119.9, 111.5, 106.0, 60.2, 19.4.

7-bromo-4,9-dihydro-3*H*-pyrido[3,4-*b*]indole 2-oxide (1n)

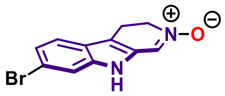

Yield: 19%; Yellow solid; ^{1}H NMR (400 MHz, DMSO-d$_6$): δ 11.27 (s, 1H), 7.80 (s, 1H), 7.58 (d, J = 1.5 Hz, 1H), 7.45 (d, J = 8.5 Hz, 1H), 7.16 (dd, J = 8.5, 1.7 Hz, 1H), 4.13 (t, J = 8.4 Hz, 2H), 3.15 (t, J = 8.5 Hz, 2H); ^{13}C NMR (101 MHz, DMSO-d$_6$): δ 138.3, 129.8, 125.1, 124.1, 122.7, 120.2, 114.7, 114.5, 106.1, 60.2, 19.4.

7-(methoxycarbonyl)-4,9-dihydro-3*H*-pyrido[3,4-*b*]indole 2-oxide (1o)

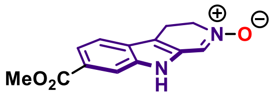

Yield: 21%; Yellow solid; ^{1}H NMR (400 MHz, DMSO-d$_6$): δ 11.52 (s, 1H), 8.02-8.01 (m, 1H), 7.88 (s, 1H), 7.65 (dd, J = 8.4, 1.4 Hz, 1H), 7.56 (d, J = 8.4 Hz, 1H), 4.17 (t, J = 8.2 Hz, 2H), 3.85 (s, 3H), 3.19 (t, J = 8.4 Hz, 2H); ^{13}C NMR (101 MHz, DMSO-d$_6$): δ 167.0, 136.7, 132.3, 128.3, 125.2, 122.7, 120.4, 118.3, 113.7, 106.1, 60.4, 51.9, 19.3.

7-methyl-4,9-dihydro-3*H*-pyrido[3,4-*b*]indole 2-oxide (1p)

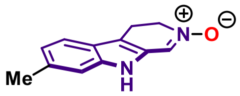

Yield: 32%; Yellow solid; ^{1}H NMR (400 MHz, DMSO-d$_6$): δ 10.98 (s, 1H), 7.74 (s, 1H), 7.27 (d, J = 8.1 Hz, 1H), 7.15 (s, 1H), 6.87 (d, J = 8.2 Hz, 1H), 4.10 (t, J = 8.4 Hz, 2H), 3.12 (t, J = 8.6 Hz, 2H), 2.35 (s,

3H); ^{13}C NMR (101 MHz, DMSO-d$_6$): δ 138.1, 131.6, 128.2, 125.3, 123.1, 121.6, 118.3, 111.7, 106.0, 60.1, 21.5, 19.7.

7-methoxy-4,9-dihydro-3*H*-pyrido[3,4-*b*]indole 2-oxide (1q)

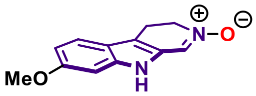

Yield: 23%; Yellow solid; ^{1}H NMR (400 MHz, DMSO-d$_6$): δ 10.94 (s, 1H), 7.72 (s, 1H), 7.37 (d, J = 8.7 Hz, 1H), 6.87 (s, 1H), 6.71 (d, J = 10.9 Hz, 1H), 4.08 (t, J = 8.4 Hz, 2H), 3.76 (s, 3H), 3.10 (t, J = 8.6 Hz, 2H); ^{13}C NMR (101 MHz, DMSO-d$_6$): δ 156.4, 138.6, 127.7, 125.3, 119.7, 119.4, 110.4, 106.4, 94.7, 59.9, 55.2, 19.7.

8-fluoro-4,9-dihydro-3*H*-pyrido[3,4-*b*]indole 2-oxide (1r)

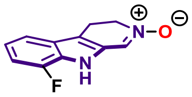

Yield: 44%; Yellow solid; ^{1}H NMR (400 MHz, DMSO-d$_6$): δ 11.66 (s, 1H), 7.69 (s, 1H), 7.32 (d, J = 7.8 Hz, 1H), 7.03-6.92 (m, 2H), 4.16 (t, J = 8.5 Hz, 2H), 3.17 (t, J = 8.5 Hz, 2H); ^{13}C NMR (101 MHz, DMSO-d$_6$): δ 150.4 (d, $J_{C\text{-}F}$ = 244.0 Hz), 130.0, 128.9 (d, $J_{C\text{-}F}$ = 5.9 Hz), 125.2 (d, $J_{C\text{-}F}$ = 13.2 Hz), 124.8, 120.1 (d, $J_{C\text{-}F}$ = 6.2 Hz), 114.9 (d, $J_{C\text{-}F}$ = 3.3 Hz), 107.1 (d, $J_{C\text{-}F}$ = 16.2 Hz), 106.7 (d, $J_{C\text{-}F}$ = 2.4 Hz), 60.3, 19.5.

8-chloro-4,9-dihydro-3*H*-pyrido[3,4-*b*]indole 2-oxide (1s)

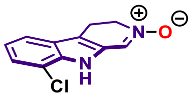

Yield: 46%; Yellow solid; ^{1}H NMR (400 MHz, DMSO-d$_6$): δ 11.48 (s, 1H), 7.69 (s, 1H), 7.48 (d, J = 7.9 Hz, 1H), 7.20 (d, J = 7.6 Hz, 1H), 7.06 (t, J = 7.8 Hz, 1H), 4.16 (t, J = 8.3 Hz, 2H), 3.17 (t, J = 8.5 Hz, 2H); ^{13}C NMR (101 MHz, DMSO-d$_6$): δ 134.2, 130.1, 127.0, 124.8, 121.5 , 120.8, 117.7, 116.0, 106.9, 60.3, 19.5.

8-bromo-4,9-dihydro-3*H*-pyrido[3,4-*b*]indole 2-oxide (1t)

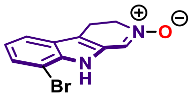

Yield: 48%; Yellow solid; ^{1}H NMR (400 MHz, DMSO-d$_6$): δ 11.34 (s, 1H), 7.70 (s, 1H), 7.52 (d, J = 7.9 Hz, 1H), 7.33 (d, J = 7.6 Hz, 1H), 7.01 (t, J = 7.8 Hz, 1H), 4.15 (t, J = 8.4 Hz, 2H), 3.17 (t, J = 8.5 Hz, 2H); ^{13}C NMR (101 MHz, DMSO-d$_6$): δ 135.7, 130.0, 126.7, 124.9, 124.5, 121.2, 118.1, 106.9, 104.3, 60.3, 19.6.

8-methyl-4,9-dihydro-3*H*-pyrido[3,4-*b*]indole 2-oxide (1u)

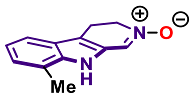

Yield: 42%; Yellow solid; ^{1}H NMR (400 MHz, DMSO-d$_6$): δ 11.13 (s, 1H), 7.71 (s, 1H), 7.31 (d, J = 7.6 Hz, 1H), 6.96-6.89 (m, 2H), 4.13 (t, J = 8.4 Hz, 2H), 3.14 (t, J = 8.5 Hz, 2H), 2.43 (s, 3H); ^{13}C NMR (101 MHz, DMSO-d$_6$): δ 137.1, 128.7, 125.2, 124.8, 122.8, 120.9, 120.0, 116.2, 106.4, 60.2, 19.7, 16.7.

8-methoxy-4,9-dihydro-3*H*-pyrido[3,4-*b*]indole 2-oxide (1v)

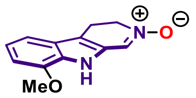

Yield: 42%; Yellow solid; ^{1}H NMR (400 MHz, DMSO-d$_6$): δ 11.25 (s, 1H), 7.62 (s, 1H), 7.08 (d, J = 8.0 Hz, 1H), 6.98 (t, J = 7.8 Hz, 1H), 6.69 (d, J = 7.6 Hz, 1H), 4.12 (t, J = 8.4 Hz, 2H), 3.91 (s, 3H), 3.13 (t, J = 8.5 Hz, 2H); ^{13}C NMR (101 MHz, DMSO-d$_6$): δ 146.2, 128.6, 127.6, 126.4, 125.1, 120.4, 111.4, 106.3, 102.7, 60.2, 55.2, 19.7.

6,7-dichloro-4,9-dihydro-3*H*-pyrido[3,4-*b*]indole 2-oxide (1w)

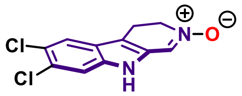

Yield: 14%; Yellow solid; ^{1}H NMR (400 MHz, DMSO-d$_6$): δ 11.41 (s, 1H), 7.81 (d, J = 8.0 Hz, 2H), 7.63 (s, 1H), 4.14 (t, J = 8.5 Hz, 2H), 3.15 (t, J = 8.5 Hz, 2H); ^{13}C NMR (101 MHz, DMSO-d$_6$): δ 136.3, 131.3, 125.0, 124.9, 124.0, 122.4, 119.6, 113.4, 105.5, 60.3, 19.3.

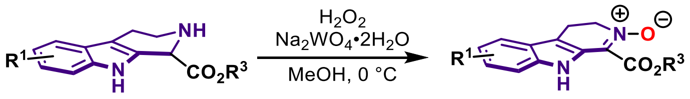

Typical Procedure: S6a-S6g were prepared according to a reported procedure[2]. To a stirred solution of **S6a** (1.0 equiv) and $Na_2WO_4\cdot 2H_2O$ (0.12 equiv) in MeOH (0.1 M) was dropwise added H_2O_2 (30% wt, 3.35 equiv) at 0 °C. The reaction was stirred at 0 °C for 2 h. Then the reaction mixture was added H_2O and extracted with DCM. The combined organic layers were washed with brine, dried over Na_2SO_4, filtered and concentrated *in vacuo*. The residue was purified by column chromatography on silica gel (DCM: EtOAc, 5:1) to give **6a-6g**.

1-(ethoxycarbonyl)-4,9-dihydro-3*H*-pyrido[3,4-*b*]indole 2-oxide (6a)

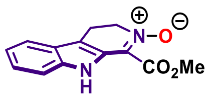

Yield: 28%; Red solid; ^{1}H NMR (400 MHz, CHCl$_3$): δ 9.29 (s, 1H), 7.51 (d, J = 8.3 Hz, 1H), 7.39 (d, J = 8.2 Hz, 1H), 7.26 (t, J = 8.3 Hz, 1H), 7.17 (t, J = 8.0 Hz, 1H), 4.46 (t, J = 8.1 Hz, 2H), 4.04 (s, 3H), 3.24 (t, J = 8.4 Hz, 2H); ^{13}C NMR (101 MHz, CHCl$_3$): δ 162.3, 137.5, 129.2, 127.3, 125.0, 123.8, 120.8,

118.9, 111.8, 108.4, 65.5, 53.1, 19.6.

1-(ethoxycarbonyl)-4,9-dihydro-3*H*-pyrido[3,4-*b*]indole 2-oxide (6b)

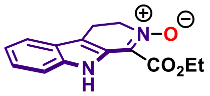

Yield: 37%; Red solid; ^{1}H NMR (400 MHz, CHCl$_3$): δ 9.27 (s, 1H), 7.50 (d, J = 7.9 Hz, 1H), 7.38 (d, J = 8.2 Hz, 1H), 7.25-7.21 (m, 1H), 7.17-7.13 (m, 1H), 4.53 (q, J = 7.1 Hz, 2H), 4.44 (t, J = 8.2 Hz, 2H), 3.24 (t, J = 8.2 Hz, 2H), 1.48 (t, J = 7.1 Hz, 3H); ^{13}C NMR (101 MHz, CHCl$_3$): δ 161.7, 137.5, 129.4, 127.3, 125.0, 123.7, 120.7, 118.8, 111.8, 108.3, 65.2, 62.5, 19.6, 14.2.

1-(ethoxycarbonyl)-6-methyl-4,9-dihydro-3*H*-pyrido[3,4-*b*]indole 2-oxide (6c)

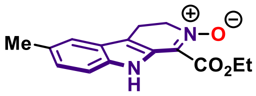

Yield: 35%; Red solid; ^{1}H NMR (400 MHz, CHCl$_3$): δ 9.14 (s, 1H), 7.25-7.24 (m, 2H), 7.06-7.03 (m, 1H), 4.51 (q, J = 7.1 Hz, 2H), 4.41 (t, J = 8.2 Hz, 2H), 3.19 (t, J = 8.2 Hz, 2H), 2.43 (s, 3H), 1.47 (t, J = 7.1 Hz, 3H); ^{13}C NMR (101 MHz, CHCl$_3$): δ 161.7, 135.9, 130.1, 129.5, 127.4, 125.5, 125.3, 118.3, 111.4, 107.8, 65.2, 62.4, 21.5, 19.6, 14.2.

1-(ethoxycarbonyl)-6-methoxy-4,9-dihydro-3*H*-pyrido[3,4-*b*]indole 2-oxide (6d)

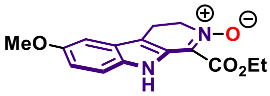

Yield: 31%; Orange solid; ^{1}H NMR (400 MHz, CHCl$_3$): δ 9.14 (s, 1H), 7.27-7.25 (m, 1H), 6.90-6.88 (m, 2H), 4.53 (q, J = 7.2 Hz, 2H), 4.43 (t, J = 8.2 Hz, 2H), 3.85 (s, 3H), 3.20 (t, J = 8.2 Hz, 1H), 1.48 (t, J = 7.1 Hz, 2H); ^{13}C NMR (101 MHz, CHCl$_3$): δ 161.7, 154.8, 132.8, 129.4, 127.9, 125.4, 114.5, 112.6, 107.9, 99.8, 65.3, 62.5, 55.8, 19.7, 14.2.

1-(ethoxycarbonyl)-7-fluoro-4,9-dihydro-3*H*-pyrido[3,4-*b*]indole 2-oxide (6e)

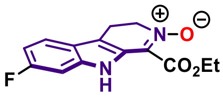

Yield: 39%; Yellow solid; ^{1}H NMR (400 MHz, CHCl$_3$): δ 9.32 (s, 1H), 7.42 (dd, J = 8.7, 5.3 Hz, 1H), 7.07 (dd, J = 9.5, 2.1 Hz, 1H), 6.94 (td, J = 9.4, 2.2 Hz, 1H), 4.52 (q, J = 7.1 Hz, 2H), 4.43 (t, J = 8.3 Hz, 2H), 3.21 (t, J = 8.3 Hz, 2H), 1.47 (t, J = 7.1 Hz, 3H); ^{13}C NMR (101 MHz, CHCl$_3$): δ 161.8 (d, $J_{C\text{-}F}$ = 242.0 Hz), 161.7, 137.6 (d, $J_{C\text{-}F}$ = 12.6 Hz), 129.1, 127.8 (d, $J_{C\text{-}F}$ = 3.9 Hz), 121.8, 119.8 (d, $J_{C\text{-}F}$ = 10.2 Hz), 109.9 (d, $J_{C\text{-}F}$ = 25.3 Hz), 108.3, 98.2 (d, $J_{C\text{-}F}$ = 26.8 Hz), 65.2, 62.6, 19.6, 14.2.

1-(ethoxycarbonyl)-7-methyl-4,9-dihydro-3*H*-pyrido[3,4-*b*]indole 2-oxide (6f)

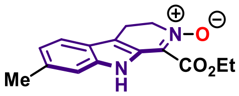

Yield: 27%; Yellow solid; ^{1}H NMR (400 MHz, $CHCl_3$): δ 9.11 (s, 1H), 7.38 (d, J = 8.2 Hz, 1H), 7.16 (s, 1H), 6.99 (d, J = 8.1 Hz, 1H), 4.53 (q, J = 7.1 Hz, 2H), 4.42 (t, J = 8.3 Hz, 2H), 3.20 (t, J = 8.3 Hz, 2H), 2.46 (s, 3H), 1.48 (t, J = 7.1 Hz, 3H); ^{13}C NMR (101 MHz, $CHCl_3$): δ 161.7, 138.0, 133.9, 129.5, 126.8, 123.0, 122.7, 118.5, 111.6, 108.4, 65.1, 62.4, 21.9, 19.7, 14.2

1-(ethoxycarbonyl)-7-methoxy-4,9-dihydro-3H-pyrido[3,4-b]indole 2-oxide (6g)

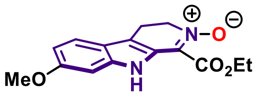

Yield: 33%; Yellow solid; ^{1}H NMR (400 MHz, $CHCl_3$): δ 9.15 (s, 1H), 7.36 (d, J = 8.6 Hz, 1H), 6.83-6.79 (m, 2H), 4.52 (q, J = 7.1 Hz, 2H), 4.40 (t, J = 8.3 Hz, 2H), 3.85 (s, 3H), 3.18-3.14 (m, 2H), 1.47 (t, J = 7.1 Hz, 3H); ^{13}C NMR (101 MHz, $CHCl_3$): δ 161.8, 157.7, 138.6, 129.4, 126.3, 119.6, 111.4, 108.7, 94.5, 64.9, 62.4, 55.6, 19.8, 14.2.

2.2 Synthesis of 3

Typical Procedure: To a stirred solution of 3,4-dihydro-*β*-carboline 2-oxide **1** (0.20 mmol, 1.0 equiv), (*S*)-phosphoric acid **4b** (0.02 mmol, 0.1 equiv) and 3 Å molecular sieves (300 mg) in $CHCl_3$ (2.0 mL) was added ethyl vinyl ether **2a** (0.40 mmol, 2.0 equiv) at −60 °C. The reaction was stirred at −60 °C until TLC indicated that the 3,4-dihydro-*β*-carboline 2-oxide disappeared. The reaction mixture was directly charged to column chromatography on silica gel (petroleum ether: EtOAc, 4:1-2:1) to give the product **3**.

(2*R*,11b*S*)-2-ethoxy-1,2,5,6,11,11b-hexahydroisoxazolo[2',3':1,2]pyrido[3,4-*b*]indole (3a)

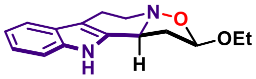

Pale yellow solid, isolated yield 85% (44 mg); m.p.: 172.5-173.1 °C; ^{1}H NMR (400 MHz, $CDCl_3$): δ 8.34 (s, 1H), 7.51 (d, J = 8.8 Hz, 1H), 7.21 (d, J = 8.9 Hz, 1H), 7.15-7.09 (m, 2H), 5.38 (dd, J = 6.5, 3.8 Hz, 1H), 4.53 (t, J = 8.8 Hz, 1H), 3.89-3.82 (m, 1H), 3.73-3.67 (m, 1H), 3.56-3.48 (m, 2H), 2.97-2.73 (m, 3H), 2.37 (ddd, J = 13.1, 9.6, 3.7 Hz, 1H), 1.18 (t, J = 7.1 Hz, 3H); ^{13}C NMR (101 MHz, $CDCl_3$): δ 136.7, 132.0, 126.3, 121.7, 119.5, 118.3, 110.9, 107.8, 106.9, 64.1, 58.6, 50.9, 41.8, 20.7, 15.1; IR (neat): ν 3449, 1718, 1665, 1637, 1560, 1508, 1459, 1080, 643 cm^{-1}; HRMS (ESI): m/z $[M + H]^+$ calcd. for $C_{15}H_{19}N_2O_2$: 259.1441; found: 259.1444; $[\alpha]_D^{23}$ = −15.7 (c = 0.5, DCM); The enantiomeric ratio of the product was determined by HPLC analysis (Column Daicel Chiracel IC; ^{i}PrOH/Hexane = 10/90; flow rate = 1.0 mL/min; t_{R1} = 16.98 min, 99.0%; t_{R2} = 21.96 min, 1.0%).

(2*R*,11b*S*)-2-ethoxy-8-fluoro-1,2,5,6,11,11b-hexahydroisoxazolo[2',3':1,2]pyrido[3,4-*b*]indole (3b)

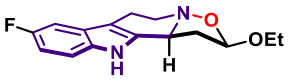

Yellow solid, isolated yield 54% (30 mg); m.p.: 168.0-168.6 °C; ^{1}H NMR (400 MHz, $CDCl_3$): δ 7.90 (s, 1H), 7.21 (dd, J = 8.8, 4.3 Hz, 1H), 7.13 (dd, J = 9.4, 2.2 Hz, 1H), 6.91 (td, J = 9.1, 2.4 Hz, 1H), 5.42 (dd, J = 6.4, 3.5 Hz, 1H), 4.59 (t, J = 8.6 Hz, 1H), 3.88-3.80 (m, 1H), 3.71-3.65 (m, 1H), 3.55-3.47 (m,

2H), 2.90-2.75 (m, 3H), 2.42-2.36 (m, 1H), 1.18 (t, J = 7.1 Hz, 3H); ^{13}C NMR (101 MHz, $CDCl_3$): δ 159.1 (d, $J_{C\text{-}F}$ = 235.6 Hz), 133.9, 133.1, 126.9 (d, $J_{C\text{-}F}$ = 10.0 Hz), 111.4 (d, $J_{C\text{-}F}$ = 9.7 Hz), 110.0 (d, $J_{C\text{-}F}$ = 26.3 Hz), 108.6 (d, $J_{C\text{-}F}$ = 4.6 Hz), 106.6, 103.7 (d, $J_{C\text{-}F}$ = 23.5 Hz), 64.1, 58.5, 50.8, 41.9, 20.5, 15.1; IR (neat): ν 3569, 2931, 1704, 1658, 1563, 1544, 1431, 1103, 708 cm^{-1}; HRMS (ESI): m/z $[M + H]^+$ calcd. for $C_{15}H_{18}N_2O_2F$: 277.1347; found: 277.1347; $[\alpha]_D^{23}$ = −38.0 (c = 0.20, DCM); The enantiomeric ratio of the product was determined by HPLC analysis (Column Daicel Chiracel IC; ^{i}PrOH/Hexane = 10/90; flow rate = 1.0 mL/min; t_{R1} = 8.44 min, 94.9%; t_{R2} = 10.99 min, 5.1%).

(2*R*,11b*S*)-8-chloro-2-ethoxy-1,2,5,6,11,11b-hexahydroisoxazolo[2',3':1,2]pyrido[3,4-*b*]indole (3c)

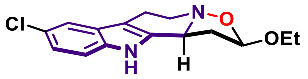

Pale yellow solid, isolated yield 83% (49 mg); m.p.: 156.3-157.1 °C; ^{1}H NMR (400 MHz, $CDCl_3$): δ 8.13 (s, 1H), 7.44 (s, 1H), 7.17 (d, J = 9.0 Hz, 1H), 7.09 (dd, J = 8.6, 2.0 Hz, 1H), 5.40 (dd, J = 6.4, 3.4 Hz, 1H), 4.56 (t, J = 8.6 Hz, 1H), 3.87-3.79 (m, 1H), 3.71-3.64 (m, 1H), 3.54-3.46 (m, 2H), 2.88-2.76 (m, 3H), 2.40-2.34 (m, 1H), 1.18 (t, J = 7.6 Hz, 3H); ^{13}C NMR (101 MHz, $CDCl_3$): δ 135.0, 133.5, 127.5, 125.3, 122.0, 118.0, 111.8, 108.0, 106.6, 64.2, 58.4, 50.7, 41.8, 20.4, 15.1; IR (neat): ν 3630, 2921, 1793, 1654, 1560, 1534, 1508, 1490, 1080, 668 cm^{-1}; HRMS (ESI): m/z $[M + H]^+$ calcd. for $C_{15}H_{18}N_2O_2Cl$: 293.1051; found: 293.1051; $[\alpha]_D^{23}$ = −46.3 (c = 0.20, DCM); The enantiomeric ratio of the product was determined by HPLC analysis (Column Daicel Chiracel IC; ^{i}PrOH/Hexane = 5/95; flow rate = 1.0 mL/min; t_{R1} = 17.07 min, 99.1%; t_{R2} = 18.50 min, 0.9%).

(2*R*,11b*S*)-2-ethoxy-8-methyl-1,2,5,6,11,11b-hexahydroisoxazolo[2',3':1,2]pyrido[3,4-*b*]indole (3d)

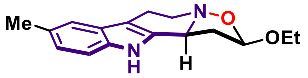

Pale yellow solid, isolated yield 84% (46 mg); m.p.: 167.3-168.0 °C; ^{1}H NMR (400 MHz, $CDCl_3$): δ 7.88 (s, 1H), 7.27 (s, 1H), 7.17 (d, J = 8.2 Hz, 1H), 6.98 (d, J = 8.1 Hz, 1H), 5.40-5.37 (m, 1H), 4.54 (t, J = 8.9 Hz, 1H), 3.89-3.81 (m, 1H), 3.70-3.63 (m, 1H), 3.55-3.48 (m, 2H), 2.92-2.78 (m, 3H), 2.44 (s, 3H), 2.38-2.33 (m, 1H), 1.20 (t, J = 7.0 Hz, 3H); ^{13}C NMR (101 MHz, $CDCl_3$): δ 135.0, 132.1, 128.8, 126.6, 123.2, 118.1, 110.5, 107.7, 106.8, 64.1, 58.7, 51.0, 41.9, 21.4, 20.8, 15.1; IR (neat): ν 3569, 2918, 1793, 1654, 1560, 1508, 1459, 1420, 1083, 701 cm^{-1}; HRMS (ESI): m/z $[M + H]^+$ calcd. for $C_{16}H_{21}N_2O_2$: 273.1598; found: 273.1598; $[\alpha]_D^{23}$ = −51.5 (c = 0.50, DCM); The enantiomeric ratio of the product was determined by HPLC analysis (Column Daicel Chiracel IC; ^{i}PrOH/Hexane = 20/80; flow rate = 1.0 mL/min; t_{R1} = 10.70 min, 95.0%; t_{R2} = 12.94 min, 5.0%).

(2*R*,11b*S*)-2-ethoxy-8-methoxy-1,2,5,6,11,11b-hexahydroisoxazolo[2',3':1,2]pyrido[3,4-*b*]indole (3e)

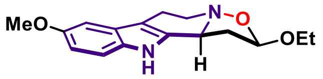

Pale yellow solid, isolated yield 80% (46 mg); m.p.: 169.9-170.3 °C; ^{1}H NMR (400 MHz, $CDCl_3$): δ 7.96 (s, 1H), 7.16 (d, J = 8.7 Hz, 1H), 6.94 (d, J = 2.2 Hz, 1H), 6.81 (dd, J = 8.7, 2.4 Hz, 1H), 5.41 (dd, J = 6.5, 3.8 Hz, 1H), 4.56 (t, J = 8.7 Hz, 1H), 3.87-3.81 (m, 4H), 3.71-3.65 (m, 1H), 3.55-3.48 (m, 2H), 2.92-2.76 (m, 3H), 2.40-2.33 (m, 1H), 1.20 (t, J = 7.2 Hz, 3H); ^{13}C NMR (101 MHz, $CDCl_3$): δ 154.1, 132.9, 131.7, 126.7, 111.6, 111.5, 107.9, 106.8, 100.6, 64.1, 58.7, 55.9, 50.9, 41.9, 20.8, 15.1; IR (neat):

ν 3568, 2918, 1793, 1654, 1560, 1508, 1459, 1420, 1083, 701 cm^{-1}; HRMS (ESI): m/z [M + H]$^+$ calcd. for $C_{16}H_{21}N_2O_3$: 289.1547; found: 289.1546; $[\alpha]_D^{23}$ = −50.8 (*c* = 0.50, DCM); The enantiomeric ratio of the product was determined by HPLC analysis (Column Daicel Chiracel IC; ^{i}PrOH/Hexane = 20/80; flow rate = 1.0 mL/min; t_{R1} = 12.91 min, 97.0%; t_{R2} = 23.39 min, 3.0%).

(2*R*,11b*S*)-9-chloro-2-ethoxy-1,2,5,6,11,11b-hexahydroisoxazolo[2',3':1,2]pyrido[3,4-*b*]indole (3f)

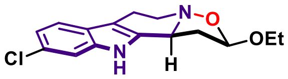

Pale yellow solid, isolated yield 82% (48 mg); m.p.: 191.7-192.3 °C; ^{1}H NMR (400 MHz, $CDCl_3$): *δ* 8.28 (s, 1H), 7.38 (d, *J* = 8.4 Hz, 1H), 7.19 (s, 1H), 7.07 (d, *J* = 9.7 Hz, 1H), 5.38 (dd, *J* = 6.5, 3.7 Hz, 1H), 4.54 (t, *J* = 8.7 Hz, 1H), 3.87-3.79 (m, 1H), 3.71-3.65 (m, 1H), 3.54-3.46 (m, 2H), 2.86-2.77 (m, 3H), 2.38-2.31 (m, 1H), 1.18 (t, *J* = 7.1 Hz, 3H); ^{13}C NMR (101 MHz, $CDCl_3$): *δ* 137.0, 132.7, 127.5, 124.9, 120.2, 119.2, 110.9, 108.2, 106.7, 64.2, 58.4, 50.7, 41.7, 20.5, 15.1; IR (neat): *ν* 3448, 2945, 1700, 1654, 1559, 1507, 1457, 1109, 669 cm^{-1}; HRMS (ESI): m/z [M + H]$^+$ calcd. for $C_{15}H_{18}N_2O_2Cl$: 293.1051; found: 293.1054; $[\alpha]_D^{23}$ = −81.4 (*c* = 0.70, DCM); The enantiomeric ratio of the product was determined by HPLC analysis (Column Daicel Chiracel IC; ^{i}PrOH/Hexane = 5/95; flow rate = 1.0 mL/min; t_{R1} = 21.37 min, 6.5%; t_{R2} = 24.66 min, 93.5%).

(2*R*,11b*S*)-9-bromo-2-ethoxy-1,2,5,6,11,11b-hexahydroisoxazolo[2',3':1,2]pyrido[3,4-*b*]indole (3g)

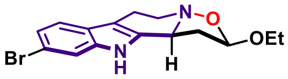

Pale yellow solid, isolated yield 87% (59 mg); m.p.: 205.7-206.2 °C; ^{1}H NMR (400 MHz, $CDCl_3$): *δ* 8.23 (s, 1H), 7.36-7.32 (m, 2H), 7.20 (d, *J* = 8.4 Hz, 1H), 5.39 (dd, *J* = 6.4, 3.6 Hz, 1H), 4.55 (t, *J* = 8.7 Hz, 1H), 3.87-3.79 (m, 1H), 3.71-3.64 (m, 1H), 3.54-3.47 (m, 2H), 2.88-2.78 (m, 3H), 2.39-2.32 (m, 1H), 1.18 (t, *J* = 7.0 Hz, 1H); ^{13}C NMR (101 MHz, $CDCl_3$): *δ* 137.4, 132.7, 125.3, 122.8, 119.6, 115.1, 113.9, 108.3, 106.7, 64.2, 58.4, 50.7, 41.7, 20.5, 15.1; IR (neat): *ν* 3568, 1654, 1539, 1541, 1508, 1458, 1437, 1094, 669cm^{-1}; HRMS (ESI): m/z [M + H]$^+$ calcd. for $C_{15}H_{18}N_2O_2Br$: 337.0546; found: 337.0547; $[\alpha]_D^{23}$ = −51.8 (*c* = 0.50, DCM); The enantiomeric ratio of the product was determined by HPLC analysis (Column Daicel Chiracel IA; ^{i}PrOH/Hexane = 20/80; flow rate = 1.0 mL/min; t_{R1} = 7.82 min, 95.0%; t_{R2} = 8.39 min, 5.0%).

methyl (2*R*,11b*S*)-2-ethoxy-1,2,5,6,11,11b-hexahydroisoxazolo[2',3':1,2]pyrido[3,4-*b*]indole-9-carboxylate (3h)

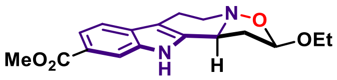

Pale yellow solid, isolated yield 70% (44 mg); m.p.: 123.8-124.5 °C; ^{1}H NMR (400 MHz, $CDCl_3$): *δ* 8.68 (s, 1H), 8.05 (s, 1H), 7.80 (d, *J* = 8.3 Hz, 1H), 7.50 (d, *J* = 8.3 Hz, 1H), 5.42 (dd, *J* = 6.4, 3.5 Hz, 1H), 4.65 (t, *J* = 8.7 Hz, 1H), 3.90 (s, 3H), 3.85-3.79 (m, 1H), 3.73-3.66 (m, 1H), 3.54-3.46 (m, 2H), 2.94-2.83 (m, 3H), 2.44-2.38 (m, 1H), 1.17 (t, *J* = 7.1 Hz, 3H); ^{13}C NMR (101 MHz, $CDCl_3$): *δ* 168.3, 135.9, 129.9, 123.1, 120.7, 117.9, 113.2, 108.6, 106.5, 64.1, 58.5, 52.0 (2C), 50.8, 41.8, 20.4, 15.1; IR (neat): *ν* 3568, 1773, 1684, 1654, 1559, 1541, 1507, 1457, 989, 673 cm^{-1}; HRMS (ESI): m/z [M + H]$^+$ calcd. for $C_{17}H_{21}N_2O_4$: 317.1496; found: 317.1495; $[\alpha]_D^{23}$ = −91.6 (*c* = 0.20, DCM); The enantiomeric

ratio of the product was determined by HPLC analysis (Column Daicel Chiracel IC; ^{i}PrOH/Hexane = 20/80; flow rate = 1.0 mL/min; t_{R1} = 19.46 min, 96.4%; t_{R2} = 22.41 min, 3.6%).

(2*R*,11b*S*)-2-ethoxy-9-methyl-1,2,5,6,11,11b-hexahydroisoxazolo[2',3':1,2]pyrido[3,4-*b*]indole (3i)

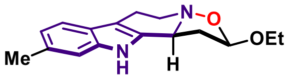

Pale yellow solid, isolated yield 81% (44 mg); m.p.: 198.6-199.4 °C; ^{1}H NMR (400 MHz, $CDCl_3$): δ 7.92 (s, 1H), 7.37 (d, J = 8.0 Hz, 1H), 7.01 (s, 1H), 6.94 (d, J = 8.8 Hz, 1H), 5.41 (dd, J = 6.6, 3.8 Hz, 1H), 4.54 (t, J = 9.0 Hz, 1H), 3.89-3.81 (m, 1H), 3.70-3.64 (m, 1H), 3.55-3.47 (m, 2H), 2.92-2.77 (m, 3H), 2.40-2.33 (m, 4H), 1.20 (t, J = 7.1 Hz, 3H); ^{13}C NMR (101 MHz, $CDCl_3$): δ 137.1, 131.5, 131.3, 124.2, 121.2, 118.0, 110.9, 107.9, 106.9, 64.1, 58.7, 50.9, 41.9, 21.6, 20.8, 15.1; IR (neat): v 3547, 2921, 1705, 1689, 1574, 1465, 1205, 1109, 671 cm^{-1}; HRMS (ESI): m/z $[M + H]^+$ calcd. for $C_{16}H_{21}N_2O_2$: 273.1598; found: 273.1599; $[\alpha]_D^{23}$ = −64.0 (c = 0.20, DCM); The enantiomeric ratio of the product was determined by HPLC analysis (Column Daicel Chiracel IC; ^{i}PrOH/Hexane = 20/80; flow rate = 1.0 mL/min; t_{R1} = 9.82 min, 96.4%; t_{R2} = 13.08 min, 3.6%).

(2*R*,11b*S*)-8,9-dichloro-2-ethoxy-1,2,5,6,11,11b-hexahydroisoxazolo[2',3':1,2]pyrido[3,4-*b*]indole (3j)

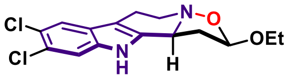

Pale yellow solid, isolated yield 55% (36 mg); m.p.: 180.2-181.0 °C; ^{1}H NMR (400 MHz, $CDCl_3$): δ 8.25 (s, 1H), 7.53 (s, 1H), 7.31 (s, 1H), 5.36 (dd, J = 6.4, 3.5 Hz, 1H), 4.48 (t, J = 8.5 Hz, 1H), 3.86-3.78 (m, 1H), 3.71-3.65 (m, 1H), 3.54-3.45 (m, 2H), 2.86-2.78 (m, 3H), 2.39-2.33 (m, 1H), 1.17 (t, J = 7.1 Hz, 3H); ^{13}C NMR (101 MHz, $CDCl_3$): δ 135.4, 134.1, 126.2, 125.5, 123.7, 119.4, 112.4, 108.1, 106.4, 64.2, 58.2, 50.6, 41.7, 20.3, 15.1; IR (neat): v 3560, 1704, 1654, 1610, 1541, 1507, 1369, 1001, 679 cm^{-1}; $[\alpha]_D^{23}$ = −44.0 (c = 0.20, DCM); HRMS (ESI): m/z $[M + H]^+$ calcd. for $C_{15}H_{17}N_2O_2Cl_2$: 327.0662; found: 327.0663; The enantiomeric ratio of the product was determined by HPLC analysis (Column Daicel Chiracel IC; ^{i}PrOH/Hexane = 10/90; flow rate = 1.0 mL/min; t_{R1} = 7.01 min, 9.5%; t_{R2} = 7.90 min, 90.5%).

(2*R*,11b*S*)-2-ethoxy-7-fluoro-1,2,5,6,11,11b-hexahydroisoxazolo[2',3':1,2]pyrido[3,4-*b*]indole (3k)

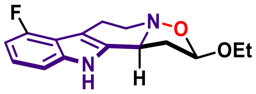

Pale yellow solid, isolated yield 51% (28 mg); m.p.: 135.4-136.0 °C; ^{1}H NMR (400 MHz, $CDCl_3$): δ 8.21 (s, 1H), 7.03-6.98 (m, 2H), 6.75-6.70 (m, 1H), 5.39 (dd, J = 6.4, 3.6 Hz, 1H), 4.52 (t, J = 8.7 Hz, 1H), 3.88-3.80 (m, 1H), 3.72-3.66 (m, 1H), 3.55-3.46 (m, 2H), 3.06-3.03 (m, 2H), 2.87-2.80 (m, 1H), 2.41-2.35 (m, 1H), 1.19 (t, J = 7.0 Hz, 3H); ^{13}C NMR (101 MHz, $CDCl_3$): δ 158.2 (d, $J_{C\text{-}F}$ = 246.8 Hz), 139.3 (d, $J_{C\text{-}F}$ = 11.8 Hz), 131.8, 122.3 (d, $J_{C\text{-}F}$ = 7.6 Hz), 115.4 (d, $J_{C\text{-}F}$ = 21.0 Hz), 107.0 (d, $J_{C\text{-}F}$ = 3.3 Hz), 106.6, 106.4, 104.9 (d, $J_{C\text{-}F}$ = 18.9 Hz), 64.1, 58.3, 50.9, 41.9, 21.9, 15.1; IR (neat): v 3560, 2922, 1770, 1654, 1559, 1507, 1458, 1022, 669 cm^{-1}; HRMS (ESI): m/z $[M + H]^+$ calcd. for $C_{15}H_{18}N_2O_2F$: 277.1347; found: 277.1348; $[\alpha]_D^{23}$ = −89.1 (c = 0.20, DCM); The enantiomeric ratio of the product was determined

by HPLC analysis (Column Daicel Chiracel IC; ^{i}PrOH/Hexane = 20/80; flow rate = 1.0 mL/min; t_{R1} = 5.71 min, 89.8%; t_{R2} = 6.25 min, 10.2%).

(2*R*,11b*S*)-2-ethoxy-10-fluoro-1,2,5,6,11,11b-hexahydroisoxazolo[2',3':1,2]pyrido[3,4-*b*]indole (3l)

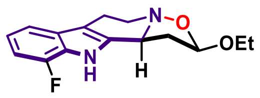

Pale yellow solid, isolated yield 58% (32 mg); m.p.: 143.1-143.8 °C; ^{1}H NMR (400 MHz, $CDCl_3$): δ 8.10 (s, 1H), 7.26 (d, J = 8.6 Hz, 1H), 7.04-6.98 (m, 1H), 6.90 (dd, J = 11.2, 7.9 Hz, 1H), 5.45 (dd, J = 6.4, 3.5 Hz, 1H), 4.65 (t, J = 8.6 Hz, 1H), 3.88-3.80 (m, 1H), 3.73-3.66 (m, 1H), 3.55-3.48 (m, 2H), 2.96-2.80 (m, 3H), 2.45-2.38 (m, 1H), 1.18 (t, J = 7.0 Hz, 3H); ^{13}C NMR (101 MHz, $CDCl_3$): δ 150.5 (d, $J_{C\text{-}F}$ = 244.2 Hz), 132.9, 130.1 (d, $J_{C\text{-}F}$ = 5.4 Hz), 124.7 (d, $J_{C\text{-}F}$ = 13.1 Hz), 120.0 (d, $J_{C\text{-}F}$ = 6.4 Hz), 114.2 (d, $J_{C\text{-}F}$ = 3.2 Hz), 109.2 (d, $J_{C\text{-}F}$ = 2.3 Hz), 107.0 (d, $J_{C\text{-}F}$ = 16.3 Hz), 106.5, 64.1, 58.4, 50.8, 42.0, 20.6, 15.1; IR (neat): ν 3434, 2954, 1700, 1653, 1559, 1467, 1208, 1096, 671 cm^{-1}; HRMS (ESI): m/z [M + H]$^{+}$ calcd. for $C_{15}H_{18}N_2O_2F$: 277.1347; found: 277.1349; $[\alpha]_D^{23}$ = −75.3 (c = 0.30, DCM); The enantiomeric ratio of the product was determined by HPLC analysis (Column Daicel Chiracel IC; ^{i}PrOH/Hexane = 10/90; flow rate = 1.0 mL/min; t_{R1} = 9.45 min, 10.2%; t_{R2} = 11.73 min, 89.8%).

(2*R*,11b*S*)-2-ethoxy-10-methyl-1,2,5,6,11,11b-hexahydroisoxazolo[2',3':1,2]pyrido[3,4-*b*]indole (3m)

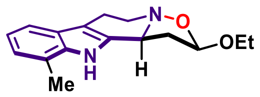

Pale yellow solid, isolated yield 55% (30 mg); m.p.: 181.5-182.2 °C; ^{1}H NMR (400 MHz, $CDCl_3$): δ 7.73 (s, 1H), 7.35 (d, J = 7.8 Hz, 1H), 7.04 (t, J = 7.4 Hz, 1H), 6.98 (d, J = 7.1 Hz, 1H), 5.46-5.44 (m, 1H), 4.65 (t, J = 9.1 Hz, 1H), 3.90-3.82 (m, 1H), 3.72-3.65 (m, 1H), 3.57-3.48 (m, 2H), 2.95-2.80 (m, 3H), 2.47 (s, 3H), 2.45-2.38 (m, 1H), 1.20 (t, J = 7.1 Hz, 3H); ^{13}C NMR (101 MHz, $CDCl_3$): δ 136.1, 131.7, 125.9, 122.5, 120.0, 119.9, 116.1, 108.8, 106.8, 64.1, 58.7, 51.0, 42.1, 20.8, 16.7, 15.1; IR (neat): ν 3560, 2921, 1700, 1658, 1559, 1507, 1457, 1025, 698 cm^{-1}; HRMS (ESI): m/z [M + H]$^{+}$ calcd. for $C_{16}H_{21}N_2O_2$: 273.1598; found: 273.1599; $[\alpha]_D^{23}$ = −92.2 (c = 0.30, DCM); The enantiomeric ratio of the product was determined by HPLC analysis (Column Daicel Chiracel IC; ^{i}PrOH/Hexane = 10/90; flow rate = 1.0 mL/min; t_{R1} = 13.11 min, 3.9%; t_{R2} = 13.88 min, 96.1%).

(3a*R*,12b*S*,12c*S*)-1,2,3a,6,7,12,12b,12c-octahydrofuro[3'',2'':4',5']isoxazolo[2',3':1,2]pyrido[3,4-*b*]indole (3n)

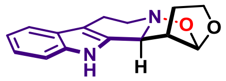

Yellow oil, isolated yield 78% (40 mg); ^{1}H NMR (400 MHz, $CDCl_3$): δ 8.11 (s, 1H), 7.53 (d, J = 7.6 Hz, 1H), 7.35 (d, J = 8.0 Hz, 1H), 7.21 (t, J = 7.2 Hz, 1H), 7.15 (t, J = 7.5 Hz, 1H), 5.68 (d, J = 5.2 Hz, 1H), 4.49 (s, 1H), 4.29-4.23 (m, 1H), 4.06-4.02 (m, 1H), 3.85 (dd, J = 13.7, 4.4 Hz, 1H), 3.35-3.32 (m, 1H), 3.19-3.12 (m, 1H), 3.06-2.99 (m, 1H), 2.64-2.59 (m, 1H), 2.30-2.23 (m, 1H), 2.20-2.15 (m, 1H); ^{13}C NMR (101 MHz, $CDCl_3$): δ 136.2, 130.8, 126.7, 122.0, 119.6, 118.4, 110.8, 108.2, 106.1, 69.0, 64.8, 52.4, 48.6, 32.1, 16.2; IR (neat): ν 3340, 2911, 1658, 1601, 1550, 1499, 999, 698 cm^{-1}; HRMS (ESI):

m/z [M + H]$^+$ calcd. for $C_{15}H_{17}N_2O_2$: 257.1285; found: 257.1286; $[\alpha]_D^{23}$ = −10.2 (*c* = 0.15, DCM); The enantiomeric ratio of the product was determined by HPLC analysis (Column Daicel Chiracel IA; ^{i}PrOH/Hexane = 10/90; flow rate = 1.0 mL/min; t_{R1} = 17.08 min, 5.3%; t_{R2} = 19.36 min, 94.7%).

2.3 Synthesis of 5

Typical Procedure: To a stirred solution of 3,4-dihydro-*β*-carboline 2-oxide **1** (0.20 mmol, 1.0 equiv), (*S*)-phosphoric acid **4b** (0.02 mmol, 0.1 equiv) and 3 Å molecular sieves (300 mg) in $CHCl_3$ (2.0 mL) was added (*E*)-4-methoxy-3-buten-2-one **2b** (0.40 mmol, 2.0 equiv) at −60 °C. The reaction was stirred at −60 °C until TLC indicated that the 3,4-dihydro-*β*-carboline 2-oxide disappeared. The reaction mixture was directly charged to column chromatography on silica gel (DCM: EtOAc, 20:1) to give the product **5**.

1-((1*R*,2*R*,11b*S*)-2-methoxy-1,2,5,6,11,11b-hexahydroisoxazolo[2',3':1,2]pyrido[3,4-*b*]indol-1-yl)ethan-1-one (5a)

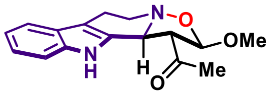

White solid, isolated yield 93% (53 mg); m.p.: 84.3-85.0 °C; ^{1}H NMR (400 MHz, $CDCl_3$): *δ* 8.38 (s, 1H), 7.49 (d, *J* = 7.8 Hz, 1H), 7.33 (d, *J* = 8.0 Hz, 1H), 7.19 (t, *J* = 7.1 Hz, 1H), 7.13 (t, *J* = 7.9 Hz, 1H), 5.37 (d, *J* = 4.8 Hz, 1H), 4.89 (d, *J* = 10.2 Hz, 1H), 3.66-3.63 (m, 2H), 3.56-3.50 (m, 4H), 3.00-2.91 (m, 1H), 2.85 (dq, *J* = 15.5, 1.8 Hz, 1H), 2.35 (s, 3H); ^{13}C NMR (101 MHz, $CDCl_3$): *δ* 206.7, 136.4, 130.8, 126.1, 121.9, 119.5, 118.3, 111.1, 109.1, 107.6, 68.8, 60.0, 56.5, 52.0, 29.2, 21.2; IR (neat): *ν* 3629, 2917, 1718, 1701, 1654, 1560, 1458, 1363, 1095, 740 cm^{-1}; HRMS (ESI): m/z [M + H]$^+$ calcd. for $C_{16}H_{19}N_2O_3$: 287.1390; found: 287.1391; $[\alpha]_D^{23}$ = −28.0 (*c* = 0.25, DCM); The enantiomeric ratio of the product was determined by HPLC analysis (Column Daicel Chiracel IA; ^{i}PrOH/Hexane = 20/80; flow rate = 1.0 mL/min; t_{R1} = 12.60 min, 4.2%; t_{R2} = 14.10 min, 95.8%).

1-((1*S*,2*S*,11b*R*)-2-methoxy-1,2,5,6,11,11b-hexahydroisoxazolo[2',3':1,2]pyrido[3,4-*b*]indol-1-yl)ethan-1-one (ent-5a)

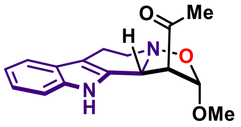

White solid, isolated yield 91% (52 mg); $[\alpha]_D^{23}$ = +28.4 (*c* = 0.25, DCM); The enantiomeric ratio of the product was determined by HPLC analysis (Column Daicel Chiracel IA; ^{i}PrOH/Hexane = 20/80; flow rate = 1.0 mL/min; t_{R1} = 11.64 min, 96.6%; t_{R2} = 13.08 min, 3.4%).

1-((1*R*,2*R*,11b*S*)-7-fluoro-2-methoxy-1,2,5,6,11,11b-hexahydroisoxazolo[2',3':1,2]pyrido[3,4-*b*]indol-1-yl)ethan-1-one (5b)

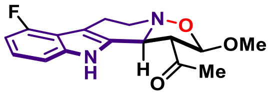

White solid, isolated yield 90% (55 mg); m.p.: 141.6-142.4 °C; ^{1}H NMR (400 MHz, $CDCl_3$): δ 8.46 (s, 1H), 7.08-7.00 (m, 2H), 6.74-6.70 (m, 1H), 5.37 (d, J = 4.7 Hz, 1H), 4.84 (d, J = 10.2 Hz, 1H), 3.66-3.60 (m, 2H), 3.56-3.48 (m, 4H), 3.13-2.98 (m, 2H), 2.35 (s, 3H); ^{13}C NMR (101 MHz, $CDCl_3$): δ 206.8, 158.1 (d, $J_{C\text{-}F}$ = 246.8 Hz), 139.1 (d, $J_{C\text{-}F}$ = 11.9 Hz), 130.7, 122.4 (d, $J_{C\text{-}F}$ = 7.8 Hz), 115.2 (d, $J_{C\text{-}F}$ = 22.6 Hz), 108.9, 107.1 (d, $J_{C\text{-}F}$ = 3.5 Hz), 105.9 (d, $J_{C\text{-}F}$ = 1.7 Hz), 104.7, 104.5, 68.9, 59.7, 56.5, 52.0, 29.2, 22.4; IR (neat): ν 3430, 1721, 1705, 1656, 1563, 1511, 1461, 1101, 670 cm^{-1}; HRMS (ESI): m/z [M + H]$^+$ calcd. for $C_{16}H_{18}N_2O_3F$: 305.1296; found: 305.1298; $[\alpha]_D^{23}$ = −27.2 (c = 0.20, DCM); The enantiomeric ratio of the product was determined by HPLC analysis (Column Daicel Chiracel IA; ^{i}PrOH/Hexane = 50/50; flow rate = 1.0 mL/min; t_{R1} = 5.41min, 2.8%; t_{R2} = 5.79 min, 97.2%).

1-((1*R*,2*R*,11b*S*)-7-chloro-2-methoxy-1,2,5,6,11,11b-hexahydroisoxazolo[2',3':1,2]pyrido[3,4-*b*]indol-1-yl)ethan-1-one (5c)

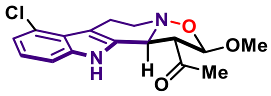

White solid, isolated yield 74% (48 mg); m.p.: 155.7-156.2 °C;^{1}H NMR (400 MHz, $CDCl_3$): δ 8.50 (s, 1H), 7.18-7.16 (m, 1H), 7.03-6.98 (m, 2H), 5.36 (d, J = 4.7 Hz, 1H), 4.83 (d, J = 10.2 Hz, 1H), 3.67-3.60 (m, 2H), 3.56 (s, 3H), 3.53-3.47 (m, 1H), 3.32 (dq, J = 16.0, 1.8 Hz, 1H), 3.17 (ddd, J = 16.6, 11.7, 5.0 Hz, 1H), 2.35 (s, 3H); ^{13}C NMR (101 MHz, $CDCl_3$): δ 206.9, 137.5, 131.7, 126.1, 123.7, 122.5, 120.0, 109.7, 108.9, 107.8, 68.9, 59.8, 56.5, 52.1, 29.2, 22.9; IR (neat): ν 3568, 1734, 1700, 1654, 1559, 1541, 1457, 1095, 669 cm^{-1}; HRMS (ESI): m/z [M + H]$^+$ calcd. for $C_{16}H_{18}N_2O_3Cl$: 321.1000; found: 321.1001; $[\alpha]_D^{23}$ = −35.6 (c = 0.20, DCM); The enantiomeric ratio of the product was determined by HPLC analysis (Column Daicel Chiracel IA; ^{i}PrOH/Hexane = 20/80; flow rate = 1.0 mL/min; t_{R1} = 9.35 min, 2.0%; t_{R2} = 10.82 min, 98.0%).

1-((1*R*,2*R*,11b*S*)-7-bromo-2-methoxy-1,2,5,6,11,11b-hexahydroisoxazolo[2',3':1,2]pyrido[3,4-*b*]indol-1-yl)ethan-1-one (5d)

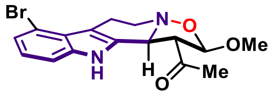

White solid, isolated yield 79% (58 mg); m.p.: 153.6-154.4 °C; ^{1}H NMR (400 MHz, $CDCl_3$): δ 8.54 (s, 1H), 7.22-7.18 (m, 2H), 6.95 (t, J = 7.8 Hz, 1H), 5.35 (d, J = 4.7 Hz, 1H), 4.82 (d, J = 10.2 Hz, 1H), 3.66-3.60 (m, 2H), 3.55 (s, 3H), 3.52-3.45 (m, 1H), 3.39-3.34 (m, 1H), 3.16 (ddd, J = 16.5, 11.8, 5.0 Hz, 1H), 2.34 (s, 3H); ^{13}C NMR (101 MHz, $CDCl_3$): δ 206.9, 137.3, 131.9, 125.1, 123.3, 122.7, 114.0, 110.3, 108.9, 108.4, 68.9, 59.8, 56.5, 52.0, 29.2, 23.0; IR (neat): ν 3430, 1704, 1657, 1562, 1461, 1365, 1065, 671 cm^{-1}; HRMS (ESI): m/z [M + H]$^+$ calcd. for $C_{16}H_{18}N_2O_3Br$: 365.0495; found: 365.0495; $[\alpha]_D^{23}$ = −16.3 (c = 0.20, DCM); The enantiomeric ratio of the product was determined by HPLC analysis (Column Daicel Chiracel IA; ^{i}PrOH/Hexane = 20/80; flow rate = 1.0 mL/min; t_{R1} = 9.55 min, 1.9%; t_{R2} = 11.17 min, 98.1%).

1-((1*R*,2*R*,11b*S*)-2,7-dimethoxy-1,2,5,6,11,11b-hexahydroisoxazolo[2',3':1,2]pyrido[3,4-*b*]indol-1-yl)ethan-1-one (5e)

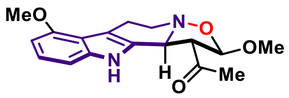

White solid, isolated yield 85% (54 mg); m.p.: 158.7-159.5 °C; ^{1}H NMR (400 MHz, $CDCl_3$): δ 8.31 (s, 1H), 7.05 (t, J = 8.0 Hz, 1H), 6.91 (d, J = 8.1 Hz, 1H), 6.47 (d, J = 7.8 Hz, 1H), 5.35 (d, J = 4.8 Hz, 1H), 4.84 (d, J = 10.2 Hz, 1H), 3.88 (s, 3H), 3.65-3.55 (m, 5H), 3.53-3.46 (m, 1H), 3.16-3.03 (m, 2H), 2.33 (s, 3H); ^{13}C NMR (101 MHz, $CDCl_3$): δ 206.8, 154.4, 137.8, 128.9, 122.7, 116.3, 109.0, 107.5, 104.4, 99.7, 69.1, 60.0, 56.5, 55.2, 52.3, 29.3, 23.1; IR (neat): ν 3569, 1738, 1704, 1658, 1563, 1461, 1259, 1109, 670 cm^{-1}; HRMS (ESI): m/z $[M + H]^+$ calcd. for $C_{17}H_{21}N_2O_4$: 317.1496; found: 317.1498; $[\alpha]_D^{23}$ = −35.6 (c = 0.20, DCM); The enantiomeric ratio of the product was determined by HPLC analysis (Column Daicel Chiracel IA; ^{i}PrOH/Hexane = 20/80; flow rate = 1.0 mL/min; t_{R1} = 10.13min, 2.9%; t_{R2} = 12.30 min, 97.1%).

1-((1*R*,2*R*,11b*S*)-8-fluoro-2-methoxy-1,2,5,6,11,11b-hexahydroisoxazolo[2',3':1,2]pyrido[3,4-*b*]indol-1-yl)ethan-1-one (5f)

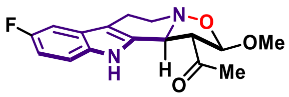

White solid, isolated yield 76% (46 mg); m.p.: 75.6-76.2 °C; ^{1}H NMR (400 MHz, $CDCl_3$): δ 8.41 (s, 1H), 7.19 (dd, J = 8.3, 3.8 Hz, 1H), 7.11 (dd, J = 9.4, 1.8 Hz, 1H), 6.89 (t, J = 8.8 Hz, 1H), 5.36 (d, J = 4.6 Hz, 1H), 4.85 (d, J = 10.2 Hz, 1H), 3.65-3.61 (m, 2H), 3.55-3.48 (m, 4H), 2.94-2.86 (m, 1H), 2.77-2.72 (m, 1H), 2.33 (s, 3H). ^{13}C NMR (101 MHz, $CDCl_3$): δ 206.8, 159.0 (d, J_{C-F} = 235.6 Hz), 132.9 (d, J_{C-F} = 21.2 Hz), 126.5 (d, J_{C-F} = 9.9 Hz), 111.6 (d, J_{C-F} = 9.8 Hz), 110.1 (d, J_{C-F} = 26.0 Hz), 109.1, 107.8 (d, J_{C-F} = 4.7 Hz), 103.6 (d, J_{C-F} = 23.6 Hz), 99.9, 68.7, 60.0, 56.5, 51.9, 29.2, 21.1; IR (neat): ν 3446, 1773, 1654, 1559, 1507, 1457, 1419, 669 cm^{-1}; HRMS (ESI): m/z $[M + H]^+$ calcd. for $C_{16}H_{18}N_2O_3F$: 305.1296; found: 305.1296; $[\alpha]_D^{23}$ = −26.5 (c = 0.24, DCM); The enantiomeric ratio of the product was determined by HPLC analysis (Column Daicel Chiracel IA; ^{i}PrOH/Hexane = 20/80; flow rate = 1.0 mL/min; t_{R1} = 11.93 min, 1.3%; t_{R2} = 13.75 min, 98.7%).

1-((1*R*,2*R*,11b*S*)-8-chloro-2-methoxy-1,2,5,6,11,11b-hexahydroisoxazolo[2',3':1,2]pyrido[3,4-*b*]indol-1-yl)ethan-1-one (5g)

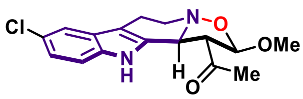

White solid, isolated yield 89% (57 mg); m.p.: 65.0-65.6 °C; ^{1}H NMR (400 MHz, $CDCl_3$): δ 8.41 (s, 1H), 7.42 (d, J = 1.4 Hz, 1H), 7.23 (d, J = 8.6 Hz, 1H), 7.11 (dd, J = 8.6, 1.9 Hz, 1H), 5.37 (d, J = 4.7 Hz, 1H), 4.85 (d, J = 10.3 Hz, 1H), 3.64-3.60 (m, 2H), 3.56 (s, 3H), 3.53 (td, J = 11.3, 4.0 Hz, 1H), 2.94-2.86 (m, 1H), 2.78 (dq, J = 16.0, 2.2 Hz, 1H), 2.35 (s, 3H); ^{13}C NMR (101 MHz, $CDCl_3$): δ 206.7, 134.8, 132.3, 127.2, 125.2, 122.1, 118.0, 112.0, 109.0, 107.4, 68.7, 59.8, 56.6, 51.9, 29.2, 21.0; IR (neat): ν 3568, 1870, 1718, 1654, 1560, 1508, 1459, 1001, 657 cm^{-1}; HRMS (ESI): m/z $[M + H]^+$ calcd. for $C_{16}H_{18}N_2O_3Cl$: 321.1000; found: 321.1001; $[\alpha]_D^{23}$ = −20.9 (c = 0.20, DCM); The enantiomeric ratio of the product was determined by HPLC analysis (Column Daicel Chiracel IA; ^{i}PrOH/Hexane = 20/80; flow rate = 1.0 mL/min; t_{R1} = 13.36 min, 5.0%; t_{R2} = 15.77 min, 95.0%).

1-((1*R*,2*R*,11b*S*)-8-bromo-2-methoxy-1,2,5,6,11,11b-hexahydroisoxazolo[2',3':1,2]pyrido[3,4-*b*]indol-1-yl)ethan-1-one (5h)

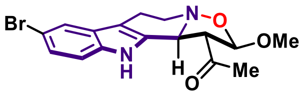

Pale yellow solid, isolated yield 90% (65 mg); m.p.: 65.1-65.8 °C; ^{1}H NMR (400 MHz, $CDCl_3$): δ 8.42 (s, 1H), 7.58 (s, 1H), 7.24 (dd, J = 8.6, 1.8 Hz, 1H), 7.18 (d, J = 9.0 Hz, 1H), 5.36 (d, J = 4.7 Hz, 1H), 4.84 (d, J = 10.3 Hz, 1H), 3.64-3.60 (m, 2H), 3.56 (s, 3H), 3.52 (td, J = 11.3, 4.0 Hz, 1H), 2.93 (td, J = 11.7, 4.8 Hz, 1H), 2.77 (dq, J = 15.4, 1.6 Hz, 1H), 2.34 (s, 3H); ^{13}C NMR (101 MHz, $CDCl_3$): δ 206.7, 135.0, 132.2, 127.8, 124.6, 121.1, 112.7, 112.5, 109.0, 107.3, 68.7, 59.8, 56.6, 51.8, 29.2, 21.0; IR (neat): v 3505, 1774, 1654, 1560, 1508, 1459, 1419, 1050, 685 cm^{-1}; HRMS (ESI): m/z [M + H]$^+$ calcd. for $C_{16}H_{18}N_2O_3Br$: 365.0495; found: 365.0494; $[\alpha]_D^{23}$ = −80.5 (c = 0.04, DCM); The enantiomeric ratio of the product was determined by HPLC analysis (Column Daicel Chiracel IA; ^{i}PrOH/Hexane = 20/80; flow rate = 1.0 mL/min; t_{R1} = 14.65 min, 5.0%; t_{R2} = 17.30 min, 95.0%).

1-((1*R*,2*R*,11b*S*)-2-methoxy-8-methyl-1,2,5,6,11,11b-hexahydroisoxazolo[2',3':1,2]pyrido[3,4-*b*]indol-1-yl)ethan-1-one (5i)

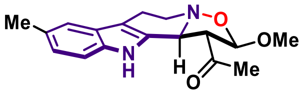

White solid, isolated yield 93% (56 mg); m.p.: 86.2-86.7 °C; ^{1}H NMR (400 MHz, $CDCl_3$): δ 8.28 (s, 1H), 7.27 (s, 1H), 7.21 (d, J = 8.3 Hz, 1H), 7.00 (d, J = 8.2 Hz, 1H), 5.36 (d, J = 4.7 Hz, 1H), 4.87 (d, J = 10.2 Hz, 1H), 3.65-3.62 (m, 2H), 3.56-3.49 (m, 4H), 2.96-2.89 (m, 1H), 2.81-2.76 (m, 1H), 2.45 (s, 3H), 2.34 (s, 3H); ^{13}C NMR (101 MHz, $CDCl_3$): δ 206.6, 134.7, 130.9, 128.8, 126.3, 123.4, 118.1, 110.7, 109.1, 107.1, 68.8, 60.0, 56.5, 52.1, 29.2, 21.4, 21.2; IR (neat): v 3438, 1704, 1658, 1563, 1544, 1461, 1100, 669 cm^{-1}; HRMS (ESI): m/z [M + H]$^+$ calcd. for $C_{17}H_{21}N_2O_3$: 301.1547; found: 301.1548; $[\alpha]_D^{23}$ = −19.6 (c = 0.23, DCM); The enantiomeric ratio of the product was determined by HPLC analysis (Column Daicel Chiracel IA; ^{i}PrOH/Hexane = 20/80; flow rate = 1.0 mL/min; t_{R1} = 13.01 min, 6.3%; t_{R2} = 15.89 min, 93.7%).

1-((1*R*,2*R*,11b*S*)-2,8-dimethoxy-1,2,5,6,11,11b-hexahydroisoxazolo[2',3':1,2]pyrido[3,4-*b*]indol-1-yl)ethan-1-one (5j)

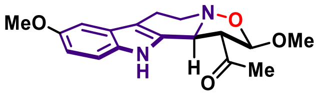

Pale yellow solid, isolated yield 90% (57 mg); m.p.: 60.6-61.2 °C; ^{1}H NMR (400 MHz, $CDCl_3$): δ 8.22 (s, 1H), 7.21 (d, J = 9.1 Hz, 1H), 6.92 (d, J = 2.4 Hz, 1H), 6.83 (dd, J = 8.8, 2.4 Hz, 1H), 5.36 (d, J = 4.8 Hz, 1H), 4.87 (d, J = 10.2 Hz, 1H), 3.84 (s, 3H), 3.65-3.61 (m, 2H), 3.56-3.49 (m, 4H), 2.96-2.88 (m, 1H), 2.80-2.75 (m, 1H), 2.34 (s, 3H); ^{13}C NMR (101 MHz, $CDCl_3$): δ 206.6, 154.1, 131.6, 131.5, 126.4, 111.8, 111.7, 109.1, 107.4, 100.5, 68.8, 60.0, 56.5, 55.9, 52.0, 29.2, 21.2; IR (neat): v 3568, 1774, 1654, 1560, 1568, 1458, 1438, 1021, 669 cm^{-1}; HRMS (ESI): m/z [M + H]$^+$ calcd. for $C_{17}H_{21}N_2O_4$: 317.1496; found: 317.1497; $[\alpha]_D^{23}$ = −35.1 (c = 0.20, DCM); The enantiomeric ratio of the product was determined

by HPLC analysis (Column Daicel Chiracel IA; ^{i}PrOH/Hexane = 20/80; flow rate = 1.0 mL/min; t_{R1} = 17.82 min, 4.8%; t_{R2} = 19.54 min, 95.2%).

1-((1*R*,2*R*,11b*S*)-9-chloro-2-methoxy-1,2,5,6,11,11b-hexahydroisoxazolo[2',3':1,2]pyrido[3,4-*b*]indol-1-yl)ethan-1-one (5k)

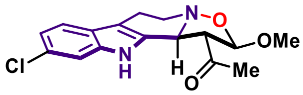

White solid, isolated yield 84% (54 mg); m.p.: 74.0-74.6 °C; ^{1}H NMR (400 MHz, $CDCl_3$): δ 8.41 (s, 1H), 7.36 (d, J = 8.4 Hz, 1H), 7.29 (d, J = 1.7 Hz, 1H), 7.06 (dd, J = 8.4, 1.8 Hz, 1H), 5.36 (d, J = 4.7 Hz, 1H), 4.84 (d, J = 10.2 Hz, 1H), 3.63-3.60 (m, 2H), 3.55 (s, 3H), 3.53 (td, J = 11.4, 4.0 Hz, 1H), 2.95-2.87 (m, 1H), 2.79 (dq, J = 15.5, 1.8 Hz, 1H), 2.34 (s, 3H); ^{13}C NMR (101 MHz, $CDCl_3$): δ 206.7, 136.8, 131.5, 127.7, 124.7, 120.2, 119.2, 111.0, 109.0, 107.8, 68.7, 59.8, 56.6, 51.8, 29.2, 21.1; IR (neat): v 3431, 1705, 1658, 1544, 1461, 1366, 1100, 671 cm^{-1}; HRMS (ESI): m/z $[M + H]^+$ calcd. for $C_{16}H_{18}N_2O_3Cl$: 321.1000; found: 321.1001; $[\alpha]_D^{23}$ = −35.0 (c = 0.20, DCM); The enantiomeric ratio of the product was determined by HPLC analysis (Column Daicel Chiracel IA; ^{i}PrOH/Hexane = 20/80; flow rate = 1.0 mL/min; t_{R1} = 10.53 min, 4.9%; t_{R2} = 15.55 min, 95.1%).

1-((1*R*,2*R*,11b*S*)-9-bromo-2-methoxy-1,2,5,6,11,11b-hexahydroisoxazolo[2',3':1,2]pyrido[3,4-*b*]indol-1-yl)ethan-1-one (5l)

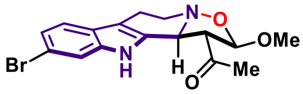

White solid, isolated yield 90% (66 mg); m.p.: 83.5-84.2 °C; ^{1}H NMR (400 MHz, $CDCl_3$): δ 8.40 (s, 1H), 7.45 (s, 1H), 7.32 (d, J = 8.4 Hz, 1H), 7.19 (d, J = 8.4 Hz, 1H), 5.37 (d, J = 4.7 Hz, 1H), 4.83 (d, J = 10.3 Hz, 1H), 3.63-3.59 (m, 2H), 3.56 (s, 3H), 3.53 (td, J = 11.2, 3.9 Hz, 1H), 2.95-2.87 (m, 1H), 2.79 (d, J = 15.6 Hz, 1H), 2.34 (s, 3H); ^{13}C NMR (101 MHz, $CDCl_3$): δ 206.7, 137.2, 131.5, 125.0, 122.8, 119.6, 115.3, 114.0, 109.0, 107.8, 68.7, 59.8, 56.6, 51.8, 29.2, 21.1; IR (neat): v 3577, 1777, 1704, 1656, 1544, 1461, 1100, 804 cm^{-1}; HRMS (ESI): m/z $[M + H]^+$ calcd. for $C_{16}H_{18}N_2O_3Br$: 365.0495; found: 365.0495; $[\alpha]_D^{23}$ = −44.5 (c = 0.20, DCM); The enantiomeric ratio of the product was determined by HPLC analysis (Column Daicel Chiracel IA; ^{i}PrOH/Hexane = 20/80; flow rate = 1.0 mL/min; t_{R1} = 11.01 min, 2.0%; t_{R2} = 17.09 min, 98.0%).

methyl (1*R*,2*R*,11b*S*)-1-acetyl-2-methoxy-1,2,5,6,11,11b-hexahydroisoxazolo[2',3':1,2]pyrido[3,4-*b*]indole-9-carboxylate (5m)

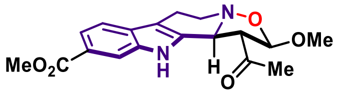

White solid, isolated yield 90% (62 mg); m.p.: 83.7-84.2 °C; ^{1}H NMR (400 MHz, $CDCl_3$): δ 8.66 (s, 1H), 8.07 (s, 1H), 7.80 (d, J = 8.4 Hz, 1H), 7.48 (d, J = 8.3 Hz, 1H), 5.38 (d, J = 4.7 Hz, 1H), 4.89 (d, J = 10.3 Hz, 1H), 3.92 (s, 3H), 3.66 (dd, J = 10.3, 4.6 Hz, 2H), 3.56-3.48 (m, 4H), 2.98-2.90 (m, 1H), 2.84 (d, J = 15.5 Hz, 1H), 2.35 (s, 3H); ^{13}C NMR (101 MHz, $CDCl_3$): δ 206.6, 168.1, 135.7, 134.5, 129.6, 123.5, 120.7, 117.9, 113.4, 109.0, 108.1, 68.6, 59.9, 56.6, 51.9, 51.8, 29.2, 21.0; IR (neat): v 3421, 1738, 1704,

1658, 1562, 1461, 1096, 670 cm^{-1}; HRMS (ESI): m/z $[M + H]^+$ calcd. for $C_{18}H_{21}N_2O_5$: 345.1445; found: 345.1446; $[\alpha]_D^{23}$ = −74.6 (c = 0.20, DCM); The enantiomeric ratio of the product was determined by HPLC analysis (Column Daicel Chiracel IA; ^{i}PrOH/Hexane = 50/50; flow rate = 1.0 mL/min; t_{R1} = 8.29 min, 2.8%; t_{R2} = 14.50 min, 97.2%).

1-((1*R*,2*R*,11b*S*)-2-methoxy-9-methyl-1,2,5,6,11,11b-hexahydroisoxazolo[2',3':1,2]pyrido[3,4-*b*]indol-1-yl)ethan-1-one (5n)

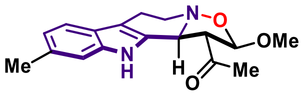

White solid, isolated yield 85% (51 mg); m.p.: 69.5-70.0 °C; ^{1}H NMR (400 MHz, $CDCl_3$): δ 8.24 (s, 1H), 7.36 (d, J = 8.0 Hz, 1H), 7.11(s, 1H), 6.94 (d, J = 8.0 Hz, 1H), 5.35 (d, J = 3.3 Hz, 1H), 4.87 (d, J = 10.2 Hz, 1H), 3.65-3.61 (m, 2H), 3.56-3.49 (m, 4H), 2.97-2.89 (m, 1H), 2.82 (d, J = 16.8 Hz, 1H), 2.45 (s, 3H), 2.34 (s, 3H); ^{13}C NMR (101 MHz, $CDCl_3$): δ 206.6, 136.9, 131.7, 130.0, 123.9, 121.2, 118.0, 111.1, 109.1, 107.4, 68.8, 60.0, 56.5, 52.0, 29.2, 21.7, 21.2; IR (neat): v 3444, 1779, 1706, 1659, 1463, 1307, 1096, 677 cm^{-1}; HRMS (ESI): m/z $[M + H]^+$ calcd. for $C_{17}H_{21}N_2O_3$: 301.1547; found: 301.1547; $[\alpha]_D^{23}$ = −49.3 (c = 0.20, DCM); The enantiomeric ratio of the product was determined by HPLC analysis (Column Daicel Chiracel IA; ^{i}PrOH/Hexane = 20/80; flow rate = 1.0 mL/min; t_{R1} = 10.06 min, 4.7%; t_{R2} = 15.02 min, 95.3%).

1-((1*R*,2*R*,11b*S*)-2,9-dimethoxy-1,2,5,6,11,11b-hexahydroisoxazolo[2',3':1,2]pyrido[3,4-*b*]indol-1-yl)ethan-1-one (5o)

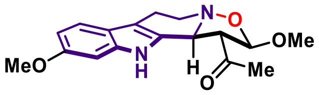

Yellow solid, isolated yield 89% (56 mg); m.p.: 121.1-121.9 °C; ^{1}H NMR (400 MHz, $CDCl_3$): δ 8.24 (s, 1H), 7.34 (d, J = 8.6 Hz, 1H), 6.82 (s, 1H), 6.77 (d, J = 8.6 Hz, 1H), 5.35 (d, J = 4.7 Hz, 1H), 4.84 (d, J = 10.2 Hz, 1H), 3.82 (s, 3H), 3.65-3.61 (m, 2H), 3.55-3.48 (m, 4H), 2.95-2.88 (m, 1H), 2.74 (dq, J = 15.1, 1.5 Hz, 1H), 2.33 (s, 3H); ^{13}C NMR (101 MHz, $CDCl_3$): δ 206.7, 156.3, 137.2, 129.4, 120.5, 118.9, 109.22, 109.16, 107.5, 94.9, 68.9, 60.0, 56.5, 55.6, 52.0, 29.2, 21.2; IR (neat): v 3426, 1721, 1704, 1658, 1563, 1544, 1461, 1378, 1095, 670 cm^{-1}; HRMS (ESI): m/z $[M + H]^+$ calcd. for $C_{17}H_{21}N_2O_4$: 317.1496; found: 317.1495; $[\alpha]_D^{23}$ = −66.4 (c = 0.50, DCM); The enantiomeric ratio of the product was determined by HPLC analysis (Column Daicel Chiracel IA; ^{i}PrOH/Hexane = 20/80; flow rate = 1.0 mL/min; t_{R1} = 15.13 min, 3.6%; t_{R2} = 25.05 min, 96.4%).

1-((1*R*,2*R*,11b*S*)-10-fluoro-2-methoxy-1,2,5,6,11,11b-hexahydroisoxazolo[2',3':1,2]pyrido[3,4-*b*]indol-1-yl)ethan-1-one (5p)

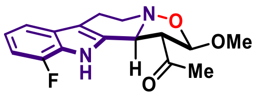

White solid, isolated yield 85% (52 mg); m.p.: 148.5-149.2 °C; ^{1}H NMR (400 MHz, $CDCl_3$): δ 8.55 (s, 1H), 7.23 (d, J = 7.9 Hz, 1H), 7.02-6.97 (m, 1H), 6.89-6.84 (m, 1H), 5.36 (d, J = 4.7 Hz, 1H), 4.89 (d, J = 10.2 Hz, 1H), 3.66-3.62 (m, 2H), 3.56-3.49 (m, 4H), 2.97-2.89 (m, 1H), 2.81 (d, J = 15.4 Hz, 1H), 2.35

(s, 3H); ^{13}C NMR (101 MHz, $CDCl_3$): δ 206.5, 150.5 (d, $J_{C\text{-}F}$ = 245.1 Hz), 131.7, 129.8 (d, $J_{C\text{-}F}$ = 5.5 Hz), 124.6 (d, $J_{C\text{-}F}$ = 13.1 Hz), 119.8 (d, $J_{C\text{-}F}$ = 6.3 Hz), 114.1 (d, $J_{C\text{-}F}$ = 3.4 Hz), 109.1, 108.5 (d, $J_{C\text{-}F}$ = 2.4 Hz), 107.0 (d, $J_{C\text{-}F}$ = 16.4 Hz), 68.7, 59.9, 56.5, 51.8, 29.2, 21.2; IR (neat): v 3450, 1707, 1659, 1582, 1373, 1228, 1098, 670 cm^{-1}; HRMS (ESI): m/z $[M + H]^+$ calcd. for $C_{16}H_{18}N_2O_3F$: 305.1296; found: 305.1297; $[\alpha]_D^{23}$ = −21.3 (c = 0.20, DCM); The enantiomeric ratio of the product was determined by HPLC analysis (Column Daicel Chiracel IA; ^{i}PrOH/Hexane = 30/70; flow rate = 1.0 mL/min; t_{R1} = 7.51 min, 98.1%; t_{R2} = 8.24 min, 1.9%).

1-((1*R*,2*R*,11b*S*)-10-bromo-2-methoxy-1,2,5,6,11,11b-hexahydroisoxazolo[2',3':1,2]pyrido[3,4-*b*]indol-1-yl)ethan-1-one (5q)

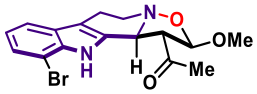

White solid, isolated yield 55% (40 mg); m.p.: 150.8-151.5 °C; ^{1}H NMR (400 MHz, $CDCl_3$): δ 8.46 (s, 1H), 7.41 (d, J = 7.8 Hz, 1H), 7.32 (d, J = 7.6 Hz, 1H), 6.99 (t, J = 7.8 Hz, 1H), 5.38 (d, J = 4.7 Hz, 1H), 4.90 (d, J = 10.2 Hz, 1H), 3.66-3.62 (m, 2H), 3.56-3.49 (m, 4H), 2.98-2.90 (m, 1H), 2.81 (d, J = 15.2 Hz, 1H), 2.37 (s, 3H); ^{13}C NMR (101 MHz, $CDCl_3$): δ 206.4, 135.1, 131.6, 127.3, 124.3, 120.7, 117.6, 109.1, 109.0, 104.6, 68.7, 59.9, 56.6, 51.8, 29.2, 21.3; IR (neat): v 3449, 1721, 1704, 1656, 1554, 1461, 1264, 1098, 670 cm^{-1}; HRMS (ESI): m/z $[M + H]^+$ calcd. for $C_{16}H_{18}N_2O_3Br$: 365.0495; found: 365.0494; $[\alpha]_D^{23}$ = −48.8 (c = 0.30, DCM); The enantiomeric ratio of the product was determined by HPLC analysis (Column Daicel Chiracel IA; ^{i}PrOH/Hexane = 20/80; flow rate = 1.0 mL/min; t_{R1} = 9.55 min, 96.8%; t_{R2} = 17.84 min, 3.2%).

1-((1*R*,2*R*,11b*S*)-2-methoxy-10-methyl-1,2,5,6,11,11b-hexahydroisoxazolo[2',3':1,2]pyrido[3,4-*b*]indol-1-yl)ethan-1-one (5r)

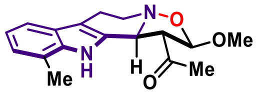

White solid, isolated yield 82% (49 mg); m.p.: 162.2-162.5 °C; ^{1}H NMR (400 MHz, $CDCl_3$): δ 8.22 (s, 1H), 7.34 (d, J = 7.7 Hz, 1H), 7.05 (t, J = 7.4 Hz, 1H), 6.98 (d, J = 7.1 Hz, 1H), 5.37 (d, J = 4.7 Hz, 1H), 4.91 (d, J = 10.2 Hz, 1H), 3.67-3.63 (m, 2H), 3.57-3.50 (m, 4H), 2.99 (ddd, J = 15.6, 11.6, 5.1 Hz, 1H), 2.84 (dq, J = 15.3, 2.1 Hz, 1H), 2.47 (s, 3H), 2.36 (s, 3H); ^{13}C NMR (101 MHz, $CDCl_3$): δ 206.8, 136.0, 130.5, 125.6, 122.5, 120.3, 119.8, 116.0, 109.1, 108.1, 68.9, 60.0, 56.5, 52.0, 29.3, 21.3, 16.6; IR (neat): v 3577, 1704, 1658, 1544, 1461, 1400, 1054, 670 cm^{-1}; HRMS (ESI): m/z $[M + H]^+$ calcd. for $C_{17}H_{21}N_2O_3$:301.1547; found: 301.1546; $[\alpha]_D^{23}$ = −50.0 (c = 0.30, DCM); The enantiomeric ratio of the product was determined by HPLC analysis (Column Daicel Chiracel IA; ^{i}PrOH/Hexane = 50/50; flow rate = 1.0 mL/min; t_{R1} = 6.53 min, 96.8%; t_{R2} = 7.93 min, 3.2%).

1-((1*R*,2*R*,11bS)-2,10-dimethoxy-1,2,5,6,11,11b-hexahydroisoxazolo[2',3':1,2]pyrido[3,4-*b*]indol-1-yl)ethan-1-one (5s)

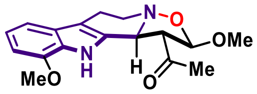

White solid, isolated yield 82% (52 mg); m.p.: 169.5-170.0 °C; ^{1}H NMR (400 MHz, $CDCl_3$): δ 8.44 (s, 1H), 7.09 (d, *J* = 7.8 Hz, 1H), 7.04 (t, *J* = 7.7 Hz, 1H), 6.64 (d, *J* = 7.6 Hz, 1H), 5.35 (d, *J* = 4.7 Hz, 1H), 4.92 (d, *J* = 10.2 Hz, 1H), 3.94 (s, 3H), 3.66-3.62 (m, 2H), 3.56-3.50 (m, 4H), 2.98-2.90 (m, 1H), 2.81 (dq, *J* = 15.3, 1.6 Hz, 1H), 2.34 (s, 3H); ^{13}C NMR (101 MHz, $CDCl_3$): δ 206.3, 146.0, 130.3, 127.3, 126.7, 120.0, 111.1, 109.2, 108.1, 102.0, 68.7, 60.0, 56.5, 55.2, 52.0, 29.3, 21.3; IR (neat): ν 3461, 1739, 1705, 1658, 1545, 1461, 1262, 1109, 671 cm^{-1}; HRMS (ESI): m/z [M + H]$^+$ calcd. for $C_{17}H_{21}N_2O_4$: 317.1496; found: 317.1495; $[\alpha]_D^{23}$ = −70.4 (*c* = 0.60, DCM); The enantiomeric ratio of the product was determined by HPLC analysis (Column Daicel Chiracel IA; ^{i}PrOH/Hexane = 20/80; flow rate = 1.0 mL/min; t_{R1} = 11.31 min, 93.1%; t_{R2} = 15.59 min, 6.9%).

1-((1*R*,2*R*,11b*S*)-8,9-dichloro-2-methoxy-1,2,5,6,11,11b-hexahydroisoxazolo[2',3':1,2]pyrido[3,4-*b*]indol-1-yl)ethan-1-one (5t)

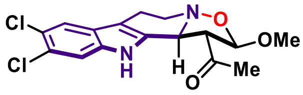

White solid, isolated yield 78% (56 mg); m.p.: 84.6-85.1 °C; ^{1}H NMR (400 MHz, $CDCl_3$): δ 8.42 (s, 1H), 7.50 (s, 1H), 7.39 (s, 1H), 5.37 (d, *J* = 4.7 Hz, 1H), 4.80 (d, *J* = 10.3 Hz, 1H), 3.62-3.58 (m, 2H), 3.56 (s, 3H), 3.52-3.45 (m, 1H), 2.92-2.83 (m, 1H), 2.75 (dq, *J* = 15.5, 1.7 Hz, 1H), 2.35 (s, 3H); ^{13}C NMR (101 MHz, $CDCl_3$): δ 206.7, 135.2, 133.0, 125.9, 125.5, 123.5, 119.4, 112.5, 109.0, 107.5, 68.6, 59.7, 56.6, 51.7, 29.1, 21.0; IR (neat): ν 3560, 1734, 1700, 1559, 1457, 1355, 1095, 668 cm^{-1}; HRMS (ESI): m/z [M + H]$^+$ calcd. for $C_{16}H_{17}N_2O_3Cl_2$: 355.0611; found: 355.0606; $[\alpha]_D^{23}$ = −31.7 (*c* = 0.2, DCM); The enantiomeric ratio of the product was determined by HPLC analysis (Column Daicel Chiracel IA; ^{i}PrOH/Hexane = 20/80; flow rate = 1.0 mL/min; t_{R1} = 12.31 min, 2.1%; t_{R2} = 17.92 min, 97.9%).

2.4 Synthesis of 7

Typical Procedure: To a stirred solution of 1-oxycarbonyl-3,4-dihydro-β-carboline 2-oxide **6** (0.20 mmol, 1.0 equiv), (*S*)-phosphoric acid **4a** (0.02 mmol, 0.1 equiv) and 3 Å molecular sieves (300 mg) in DCE (2.0 mL) was added ethyl vinyl ether **2a** (0.40 mmol, 2.0 equiv) at −60 °C. The reaction was stirred at −60 °C until TLC indicated that the 1-oxycarbonyl-3,4-dihydro-β-carboline 2-oxide disappeared. The reaction mixture was directly charged to column chromatography on silica gel (petroleum ether: EtOAc, 4:1) to give the product **7**.

methyl (2*R*,11b*R*)-2-ethoxy-1,2,6,11-tetrahydroisoxazolo[2',3':1,2]pyrido[3,4-*b*]indole-11b(5*H*)-carboxylate (7a)

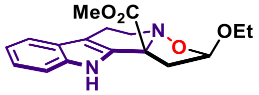

Yellow solid, isolated yield 51% (32 mg); m.p.: 138.1-138.9 °C; ^{1}H NMR (400 MHz, $CDCl_3$): δ 8.28 (s, 1H), 7.52 (d, *J* = 7.8 Hz, 1H), 7.36 (d, *J* = 8.1 Hz, 1H), 7.22 (td, *J* = 7.2, 1.2 Hz, 1H), 7.14 (td, *J* = 7.5, 0.9 Hz, 1H), 5.18 (dd, *J* = 5.7, 1.6 Hz, 1H), 3.91-3.83 (m, 1H), 3.79 (s, 3H), 3.57-3.54 (m, 2H), 3.49-3.41 (m, 1H), 3.04-2.93 (m, 2H), 2.90 (dd, *J* = 13.2, 5.8 Hz, 1H), 2.80-2.73 (m, 1H), 1.23 (t, *J* = 7.0 Hz, 3H); ^{13}C NMR (101 MHz, $CDCl_3$): δ 171.6, 136.5, 129.0, 126.2, 122.6, 119.8, 118.7, 111.2, 109.7, 100.5,

68.1, 63.4, 53.2, 49.5, 46.8, 17.5, 14.9; IR (neat): *ν* 3703, 2954, 1657, 1602, 1410, 1299, 1100, 756 cm^{-1}; HRMS (ESI): m/z [M − H]$^-$ calcd. for $C_{17}H_{21}N_2O_4$: 317.1496; found: 317.1497; $[\alpha]_D^{23}$ = −14.7 (*c* = 0.05, DCM); The enantiomeric ratio of the product was determined by HPLC analysis (Column Daicel Chiracel IC; ^{i}PrOH/Hexane = 10/90; flow rate = 0.5 mL/min; t_{R1} = 18.38 min, 90.3%; t_{R2} = 24.95 min, 9.7%).

ethyl (2*R*,11b*R*)-2-ethoxy-1,2,6,11-tetrahydroisoxazolo[2',3':1,2]pyrido[3,4-*b*]indole-11b(5*H*)-carboxylate (7b)

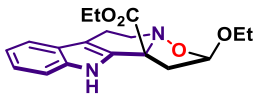

White solid, isolated yield 61% (40 mg); m.p.: 145.4-146.0 °C; ^{1}H NMR (400 MHz, $CDCl_3$): *δ* 8.30 (s, 1H), 7.54 (d, *J* = 7.8 Hz, 1H), 7.37 (d, *J* = 8.1 Hz, 1H), 7.23 (t, *J* = 7.3 Hz, 1H), 7.15 (t, *J* = 7.4 Hz, 1H), 5.15 (d, *J* = 5.4 Hz, 1H), 4.29 (q, *J* = 7.0 Hz, 2H), 3.92-3.84 (m, 1H), 3.65-3.41 (m, 3H), 3.03-2.89 (m, 3H), 2.78 (dt, *J* = 15.8, 4.4 Hz, 1H), 1.32 (d, *J* = 7.1 Hz, 3H), 1.24 (t, *J* = 7.1 Hz, 3H); ^{13}C NMR (101 MHz, $CDCl_3$): *δ* 170.7, 136.4, 129.2, 126.2, 122.5, 119.7, 118.7, 111.1, 109.6, 100.4, 68.0, 63.5, 62.3, 49.0, 46.8, 17.2, 14.9, 14.1; IR (neat): *ν* 3659, 2931, 1738, 1658, 1461, 1264, 1101, 746 cm^{-1}; HRMS (ESI): m/z [M − H]$^-$ calcd. for $C_{18}H_{21}N_2O_4$: 329.1507; found: 329.1508; $[\alpha]_D^{23}$ = −28.0 (*c* = 0.10, DCM); The enantiomeric ratio of the product was determined by HPLC analysis (Column Daicel Chiracel IC; ^{i}PrOH/Hexane = 5/95; flow rate = 1.0 mL/min; t_{R1} = 12.24 min, 95.9%; t_{R2} = 19.47 min, 4.1%).

ethyl (2*R*,11b*R*)-2-ethoxy-8-methyl-1,2,6,11-tetrahydroisoxazolo[2',3':1,2]pyrido[3,4-*b*]indole-11b(5*H*)-carboxylate (7c)

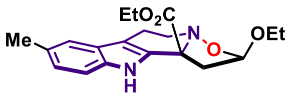

Yellow solid, isolated yield 52% (36 mg); m.p.: 128.2-128.9 °C; ^{1}H NMR (400 MHz, $CDCl_3$): *δ* 8.17 (s, 1H), 7.31 (s, 1H), 7.25 (d, *J* = 8.4 Hz, 1H), 7.04 (d, *J* = 8.2 Hz, 1H), 5.14 (d, *J* = 5.6 Hz, 1H), 4.28 (q, *J* = 7.2 Hz, 2H), 3.91-3.83 (m, 1H), 3.63-3.52 (m, 2H), 3.48-3.40 (m, 1H), 3.01-2.87 (m, 3H), 2.75-2.69 (m, 1H), 2.45 (s, 3H), 1.31 (t, *J* = 7.1 Hz, 3H), 1.23 (t, *J* = 7.1 Hz, 3H); ^{13}C NMR (101 MHz, $CDCl_3$): *δ* 170.8, 134.7, 129.3, 129.1, 126.5, 124.0, 118.3, 110.8, 109.2, 100.4, 68.1, 63.5, 62.3, 49.1, 46.7, 21.4, 17.3, 14.9, 14.1; IR (neat): *ν* 3638, 2936, 1738, 1657, 1562, 1544, 1264, 1102, 753 cm^{-1}; HRMS (ESI): m/z [M − H]$^-$ calcd. for $C_{19}H_{23}N_2O_4$: 343.1663; found: 343.1660; $[\alpha]_D^{23}$ = −40.2 (*c* = 0.35, DCM); The enantiomeric ratio of the product was determined by HPLC analysis (Column Daicel Chiracel IC; ^{i}PrOH/Hexane = 5/95; flow rate = 1.0 mL/min; t_{R1} = 13.41 min, 97.4%; t_{R2} = 19.40 min, 2.6%).

ethyl (2*R*,11b*R*)-2-ethoxy-8-methoxy-1,2,6,11-tetrahydroisoxazolo[2',3':1,2]pyrido[3,4-*b*]indole-11b(5*H*)-carboxylate (7d)

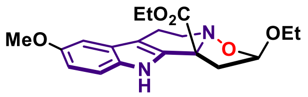

Yellow solid, isolated yield 53% (38 mg); m.p.: 100.2-100.8 °C; ^{1}H NMR (400 MHz, $CDCl_3$): *δ* 8.18 (s, 1H), 7.25 (d, *J* = 8.8 Hz, 1H), 6.96 (s, 1H), 6.87 (d, *J* = 8.8 Hz, 1H), 5.14 (d, *J* = 5.6 Hz, 1H), 4.27 (q, *J*

= 7.1 Hz, 2H), 3.90-3.85 (m, 4H), 3.62-3.40 (m, 3H), 3.00-2.87 (m, 3H), 2.73-2.67 (m, 1H), 1.31 (d, *J* = 7.2 Hz, 3H), 1.23 (d, *J* = 7.0 Hz, 3H); ^{13}C NMR (101 MHz, $CDCl_3$): *δ* 170.7, 154.2, 131.5, 130.0, 126.6, 112.6, 111.9, 109.4, 100.6, 100.4, 68.1, 63.5, 62.3, 55.9, 49.0, 46.8, 17.3, 14.9, 14.1; IR (neat): *ν* 3638, 2935, 1738, 1657, 1582, 1544, 1461, 1220, 1101, 750 cm^{-1}; HRMS (ESI): m/z [M − H]$^-$ calcd. for $C_{19}H_{23}N_2O_5$: 359.1613; found: 359.1612; $[\alpha]_D^{23}$ = −18.3 (*c* = 0.10, DCM); The enantiomeric ratio of the product was determined by HPLC analysis (Column Daicel Chiracel IC; ^{i}PrOH/Hexane = 5/95; flow rate = 1.0 mL/min; t_{R1} = 15.08 min, 99.99%; t_{R2} = 19.14 min, 0.001%).

ethyl (2*R*,11b*R*)-2-ethoxy-9-fluoro-1,2,6,11-tetrahydroisoxazolo[2',3':1,2]pyrido[3,4-*b*]indole-11b(5*H*)-carboxylate (7e)

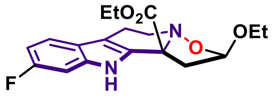

Yellow solid, isolated yield 52% (36 mg); m.p.: 161.8-162.5 °C; ^{1}H NMR (400 MHz, $CDCl_3$): *δ* 8.31 (s, 1H), 7.42 (dd, *J* = 8.5, 5.4 Hz, 1H), 7.05 (d, *J* = 9.6 Hz, 1H), 6.88 (t, *J* = 9.4 Hz, 1H), 5.13 (d, *J* = 5.3 Hz, 1H), 4.28 (q, *J* = 7.1 Hz, 2H), 3.90-3.83 (m, 1H), 3.64-3.59 (m, 1H), 3.55-3.40 (m, 2H), 2.99-2.87 (m, 3H), 2.73-2.67 (m, 1H), 1.31 (t, *J* = 7.1 Hz, 3H), 1.23 (t, *J* = 7.1 Hz, 3H); ^{13}C NMR (101 MHz, $CDCl_3$): *δ* 170.7, 161.3 (d, $J_{C\text{-}F}$ = 239.5 Hz), 136.5 (d, $J_{C\text{-}F}$ = 12.5 Hz), 129.4 (d, $J_{C\text{-}F}$ = 3.5 Hz), 122.9, 119.4 (d, $J_{C\text{-}F}$ = 10.2 Hz), 109.7, 108.5 (d, $J_{C\text{-}F}$ = 24.7 Hz), 100.4, 97.8 (d, $J_{C\text{-}F}$ = 26.5 Hz), 67.9, 63.6, 62.4, 48.9, 46.8, 17.1, 14.9, 14.1; IR (neat): *ν* 3638, 2927, 1738, 1667, 1562, 1461, 1205, 1101, 799 cm^{-1}; HRMS (ESI): m/z [M − H]$^-$ calcd. for $C_{18}H_{20}N_2O_4F$: 347.1413; found: 347.1410; $[\alpha]_D^{23}$ = −17.1 (*c* = 0.70, DCM); The enantiomeric ratio of the product was determined by HPLC analysis (Column Daicel Chiracel IC; ^{i}PrOH/Hexane = 10/90; flow rate = 1.0 mL/min; t_{R1} = 8.94 min, 91.5%; t_{R2} = 13.35 min, 8.5%).

ethyl (2*R*,11b*R*)-2-ethoxy-9-methyl-1,2,6,11-tetrahydroisoxazolo[2',3':1,2]pyrido[3,4-*b*]indole-11b(5*H*)-carboxylate (7f)

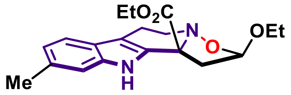

Yellow solid, isolated yield 55% (38 mg); m.p.: 169.3-170.2 °C; ^{1}H NMR (400 MHz, $CDCl_3$): *δ* 8.13 (s, 1H), 7.40 (d, *J* = 8.0 Hz, 1H), 7.15 (s, 1H), 6.97 (d, *J* = 8.0 Hz, 1H), 5.14 (d, *J* = 4.3 Hz, 1H), 4.28 (q, *J* = 7.1 Hz, 2H), 3.91-3.83 (m, 1H), 3.61-3.42 (m, 3H), 3.00-2.87 (m, 3H), 2.75-2.69 (m, 1H), 2.45 (s, 3H), 1.31 (t, *J* = 7.2 Hz, 3H), 1.23 (t, *J* = 7.1 Hz, 3H); ^{13}C NMR (101 MHz, $CDCl_3$): *δ* 170.8, 136.9, 132.4, 128.4, 124.1, 121.4, 118.3, 111.1, 109.5, 100.4, 68.0, 63.5, 62.2, 49.1, 46.7, 21.7, 17.3, 14.9, 14.1; IR (neat): *ν* 3659, 2931, 1704, 1657, 1544, 1461, 1299, 1009, 751 cm^{-1}; HRMS (ESI): m/z [M − H]$^-$ calcd. for $C_{19}H_{23}N_2O_4$: 343.1663; found: 343.1661; $[\alpha]_D^{23}$ = −17.0 (*c* = 0.40, DCM); The enantiomeric ratio of the product was determined by HPLC analysis (Column Daicel Chiracel IC; ^{i}PrOH/Hexane = 10/90; flow rate = 1.0 mL/min; t_{R1} = 9.68 min, 98.0%; t_{R2} = 16.13 min, 2.0%).

ethyl (2*R*,11b*R*)-2-ethoxy-9-methoxy-1,2,6,11-tetrahydroisoxazolo[2',3':1,2]pyrido[3,4-*b*]indole-11b(5*H*)-carboxylate (7g)

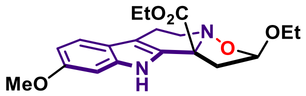

Yellow solid, isolated yield 64% (46 mg); m.p.: 113.1-113.8 °C; ^{1}H NMR (400 MHz, $CDCl_3$): δ 8.18 (s, 1H), 7.39 (d, J = 8.6 Hz, 1H), 6.86 (s, 1H), 6.80 (d, J = 8.6 Hz, 1H), 5.14 (d, J = 5.4 Hz, 1H), 4.27 (q, J = 7.2 Hz, 2H), 3.88-3.83 (m, 4H), 3.61-3.42 (m, 3H), 2.99-2.86 (m, 3H), 2.73-2.66 (m, 1H), 1.31 (t, J = 7.1 Hz, 3H), 1.22 (t, J = 7.1 Hz, 3H); ^{13}C NMR (101 MHz, $CDCl_3$): δ 170.8, 156.8, 137.3, 127.8, 120.7, 119.2, 109.5, 100.4, 94.9, 68.0, 63.5, 62.2, 55.6, 49.0, 46.7, 17.3, 14.9, 14.1; IR (neat): v 3659, 2933, 1738, 1657, 1562, 1510, 1299, 1109, 748 cm^{-1}; HRMS (ESI): m/z [M − H]$^-$ calcd. for $C_{19}H_{23}N_2O_5$: 359.1613; found: 359.1610; $[\alpha]_D^{23}$ = −11.6 (c = 0.30, DCM); The enantiomeric ratio of the product was determined by HPLC analysis (Column Daicel Chiracel IC; ^{i}PrOH/Hexane = 10/90; flow rate = 1.0 mL/min; t_{R1} = 17.05 min, 85.7%; t_{R2} = 20.95 min, 14.3%).

ethyl (1*R*,2*R*,11b*R*)-1-acetyl-2-methoxy-1,2,6,11-tetrahydroisoxazolo[2',3':1,2]pyrido[3,4-*b*]indole-11b(5*H*)-carboxylate (7h)

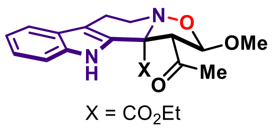

White form, isolated yield 88% (64 mg); ^{1}H NMR (400 MHz, $CDCl_3$): δ 8.92 (s, 1H), 7.52 (d, J = 7.9 Hz, 1H), 7.38 (d, J = 8.1 Hz, 1H), 7.22 (d, J = 8.2 Hz, 1H), 7.14 (t, J = 8.0 Hz, 1H), 5.69 (d, J = 4.9 Hz, 1H), 4.24-4.10 (m, 2H), 3.76-3.70 (m, 2H), 3.57-3.50 (m, 4H), 3.06-2.98 (m, 1H), 2.88-2.82 (m, 1H), 2.42 (s, 3H), 1.20 (t, J = 7.1 Hz, 3H); ^{13}C NMR (101 MHz, $CDCl_3$): δ 206.5, 170.1, 136.8, 129.5, 125.7, 122.4, 119.5, 118.7, 111.4, 109.5, 108.9, 74.2, 70.2, 62.5, 56.7, 53.5, 29.3, 20.8, 13.9; IR (neat): v 3666, 2933, 1738, 1557, 1462, 1410, 1199, 1097, 749 cm^{-1}; HRMS (ESI): m/z [M + H]$^+$ calcd. for $C_{19}H_{23}N_2O_5$: 359.1601; found: 359.1601; $[\alpha]_D^{23}$ = −22.58 (c = 0.31, DCM); The enantiomeric ratio of the product was determined by HPLC analysis (Column Daicel Chiracel AD-H; ^{i}PrOH/Hexane = 10/90; flow rate = 1.0 mL/min; t_{R1} = 22.95 min, 99.7%; t_{R2} = 29.27 min, 0.3%).

2.5 Procedures for further synthetic transformation

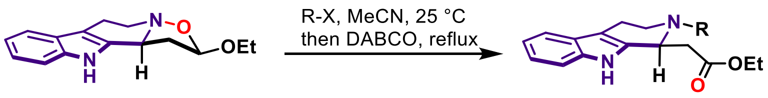

ethyl (*S*)-2-(2-methyl-2,3,4,9-tetrahydro-1*H*-pyrido[3,4-*b*]indol-1-yl)acetate (8a)

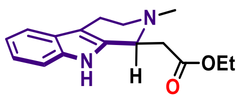

To a solution of **3a** (39 mg, 0.15 mmol, 1.0 equiv) in MeCN (1.5 mL) was added MeI (26 mg, 0.18 mmol, 1.2 equiv). The reaction mixture was stirred at 25 °C for 1 h. Then DABCO (20 mg, 0.18 mmol, 1.2 equiv) was added and the reaction was refluxed for 1 h. The reaction solution was concentrated *in vacuo*. The residue was purified by column chromatography on silica gel (petroleum ether: EtOAc, 5:1) to give **8a**.

White solid, isolated yield 64% (26 mg); m.p.: 65.8-69.1 °C; ^{1}H NMR (400 MHz, $CDCl_3$): δ 8.58 (s, 1H),

7.52 (d, J = 7.7 Hz, 1H), 7.34 (d, J = 8.4 Hz, 1H), 7.16 (t, J = 8.0 Hz, 1H), 7.09 (t, J = 7.0 Hz, 1H), 4.25 (q, J = 7.2 Hz, 2H), 4.08 (dd, J = 9.9, 3.4 Hz, 1H), 3.14-3.07 (m, 1H), 3.02 (dd, J = 17.0, 3.6 Hz, 1H), 2.94-2.86 (m, 2H), 2.79-2.66 (m, 2H), 2.54 (s, 3H), 1.31 (t, J = 7.2 Hz, 3H); ^{13}C NMR (101 MHz, $CDCl_3$): δ 173.8, 135.7, 134.1, 126.8, 121.6, 119.1, 118.1, 110.9, 107.4, 61.0, 55.4, 48.1, 42.0, 39.4, 18.4, 14.1; IR (neat): v 3442, 2928, 1723, 1658, 1461, 1180, 1031, 744, 670 cm^{-1}; HRMS (ESI): m/z [M + H]$^+$ calcd. for $C_{16}H_{21}N_2O_2$: 273.1598; found: 273.1594; $[\alpha]_D^{23}$ = +35.3 (c = 0.10, DCM); The enantiomeric ratio of the product was determined by HPLC analysis (Column Daicel Chiracel IC; ^{i}PrOH/Hexane = 10/90; flow rate = 1.0 mL/min; t_{R1} = 6.33 min, 96.9%; t_{R2} = 13.05 min, 3.1%).

ethyl (*S*)-2-(2-benzyl-2,3,4,9-tetrahydro-1*H*-pyrido[3,4-*b*]indol-1-yl)acetate (8b)

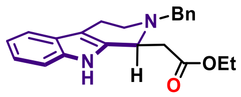

To a solution of **3a** (39 mg, 0.15 mmol, 1 equiv) in MeCN (1.5 mL) was added BnBr (37 mg, 0.18 mmol, 1.2 equiv). The reaction mixture was stirred at 25 °C for 1 h. Then DABCO (20 mg, 0.18 mmol, 1.2 equiv) was added and the reaction was refluxed for 1 h. The reaction solution was concentrated *in vacuo*. The residue was purified by column chromatography on silica gel (petroleum ether: EtOAc, 20:1) to give **8b**.

Yellow oil, isolated yield 96% (50 mg);^{1}H NMR (400 MHz, $CDCl_3$): δ 8.52 (s, 1H), 7.57 (d, J = 7.6 Hz, 1H), 7.41-7.35 (m, 5H), 7.32-7.30 (m, 1H), 7.22 (t, J = 7.0 Hz, 1H), 7.16 (t, J = 7.0 Hz, 1H), 4.26-4.15 (m, 3H), 3.83 (s, 2H), 3.22-3.15 (m, 1H), 3.08-2.85 (m, 4H), 2.64 (ddd, J = 15.4, 4.3, 1.6 Hz, 1H), 1.31 (t, J = 7.2 Hz, 3H); ^{13}C NMR (101 MHz, $CDCl_3$): δ 173.5, 139.3, 135.6, 134.2, 128.6, 128.3, 127.0, 126.9, 121.6, 119.1, 118.1, 110.9, 107.5, 60.8, 57.4, 52.8, 44.5, 40.3, 17.7, 14.1; IR (neat): v 3865, 3414, 2928, 1722, 1454, 1335, 865, 729 cm^{-1}; HRMS (ESI): m/z [M + H]$^+$ calcd. for $C_{22}H_{25}N_2O_2$: 349.1911; found: 349.1906; $[\alpha]_D^{23}$ = +5.6 (c = 0.36, DCM); The enantiomeric ratio of the product was determined by HPLC analysis (Column Daicel Chiracel ID; ^{i}PrOH/Hexane = 10/90; flow rate = 1.0 mL/min; t_{R1} = 5.92 min, 97.7%; t_{R2} = 6.25 min, 2.3%).

ethyl (*S*)-2-(2-allyl-2,3,4,9-tetrahydro-1*H*-pyrido[3,4-*b*]indol-1-yl)acetate (8c)

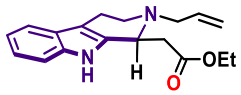

To a solution of **3a** (39 mg, 0.15 mmol, 1.0 equiv) in MeCN (1.5 mL) was added allyl bromide (22 mg, 0.18 mmol, 1.2 equiv). The reaction mixture was stirred at 25 °C for 1 h. Then DABCO (20 mg, 0.18 mmol, 1.2 equiv) was added and the reaction was refluxed for 1 h. The reaction solution was concentrated *in vacuo*. The residue was purified by column chromatography on silica gel (petroleum ether: EtOAc, 15:1) to give **8c**.

Yellow oil, isolated yield 80% (36 mg); ^{1}H NMR (400 MHz, $CDCl_3$): δ 8.49 (s, 1H), 7.52 (d, J = 7.8 Hz, 1H), 7.34 (d, J = 8.0 Hz, 1H), 7.19 (t, J = 7.0 Hz, 1H), 7.12 (t, J = 6.8 Hz, 1H), 5.98-5.88 (m, 1H), 5.22-5.16 (m, 2H), 4.28-4.19 (m, 3H), 3.30 (d, J = 6.4 Hz, 2H), 3.17-3.05 (m, 2H), 2.98-2.80 (m, 3H), 2.61 (dq, J = 15.7, 2.3 Hz, 1H), 1.31 (d, J = 7.2 Hz, 3H); ^{13}C NMR (101 MHz, $CDCl_3$): δ 173.6, 136.1, 135.5, 134.1, 126.9, 121.6, 119.1, 118.1, 117.6, 110.9, 107.5, 60.9, 56.4, 52.1, 44.8, 40.0, 17.7, 14.1; IR (neat):

ν 2929, 1721, 1705, 1658, 1461, 1366, 1100, 670 cm^{-1}; HRMS (ESI): m/z [M + H]$^+$ calcd. for $C_{18}H_{23}N_2O_2$: 299.1754; found: 299.1750; $[\alpha]_D^{23}$ = +44.3 (c = 0.20, DCM); The enantiomeric ratio of the product was determined by HPLC analysis (Column Daicel Chiracel IC; ^{i}PrOH/Hexane = 5/95; flow rate = 0.5 mL/min; t_{R1} = 12.53 min, 97.2%; t_{R2} = 13.48 min, 2.8%).

ethyl (*S*, *Z*)-2-(2-(2-iodobut-2-en-1-yl)-2,3,4,9-tetrahydro-1*H*-pyrido[3,4-*b*]indol-1-yl)acetate (8d)

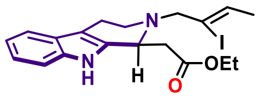

To a solution of **3a** (39 mg, 0.15 mmol, 1.0 equiv) in MeCN (1.5 mL) was added (*Z*)-1-bromo-2-iodo-2-butene (47 mg, 0.18 mmol, 1.2 equiv). The reaction mixture was stirred at 25 °C for 10 h. Then DABCO (20 mg, 0.18 mmol, 1.2 equiv) was added and the reaction was refluxed for 1 h. The reaction solution was concentrated *in vacuo*. The residue was purified by column chromatography on silica gel (petroleum ether: EtOAc, 15:1) to give **8d**.

Yellow oil, isolated yield 85% (56 mg); ^{1}H NMR (400 MHz, $CDCl_3$): δ 8.57 (s, 1H), 7.54 (d, J = 7.7 Hz, 1H), 7.36 (d, J = 8.0 Hz, 1H), 7.21 (t, J = 7.2 Hz, 1H), 7.14 (t, J = 7.9 Hz, 1H), 5.88 (q, J = 6.4 Hz, 1H), 4.27 (qd, J = 7.2, 1.2 Hz, 2H), 4.20 (dd, J = 10.2, 4.2 Hz, 1H), 3.43 (s, 2H), 3.22-3.15 (m, 1H), 3.08-3.01 (m, 2H), 2.93-2.84 (m, 2H), 2.64-2.58 (m, 1H), 1.85 (d, J = 6.4 Hz, 3H), 1.33 (t, J = 7.2 Hz, 3H); ^{13}C NMR (101 MHz, $CDCl_3$): δ 173.7, 135.4, 134.1, 132.2, 126.8, 121.6, 119.1, 118.0, 110.9, 110.1, 107.5, 64.9, 60.9, 51.8, 44.2, 40.7, 21.7, 17.7, 14.1; IR (neat): ν 2956, 1774, 1752, 1657, 1452, 1268, 1001, 709 cm^{-1}; HRMS (ESI): m/z [M − H]$^-$ calcd. for $C_{19}H_{22}N_2O_2I$: 437.0731; found: 437.0729; $[\alpha]_D^{23}$ = +13.0 (c = 0.20, DCM); The enantiomeric ratio of the product was determined by HPLC analysis (Column Daicel Chiracel IC; ^{i}PrOH/Hexane = 10/90; flow rate = 1.0 mL/min; t_{R1} = 10.35 min, 4.4%; t_{R2} = 12.83 min, 95.6%).

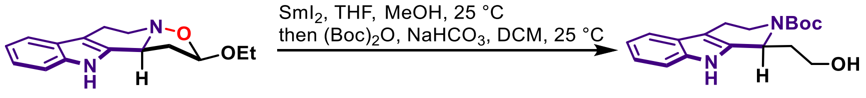

3a **9**

***tert*-butyl (*S*)-1-(2-hydroxyethyl)-1,3,4,9-tetrahydro-2*H*-pyrido[3,4-*b*]indole-2-carboxylate (9)**

To a stirred solution of **3a** (39 mg, 0.15 mmol, 1.0 equiv) in MeOH (1 mL) was added SmI_2 (7.5 mL, 0.1 mmol in THF, 0.75 mmol, 5.0 equiv) at 25 °C. After stirring for 10 min at 25 °C, the reaction mixture was quenched with saturated aqueous $NaHCO_3$. The aqueous layer was extracted with EtOAc. The combined organic layers were washed with brine, dried over Na_2SO_4, and evaporated *in vacuo*. The above residue was dissolved in DCM (1.5 mL), and $NaHCO_3$ (15 mg, 0.18 mmol, 1.2 equiv), $(Boc)_2O$ (39 mg, 0.18 mmol, 1.2 equiv) were added. The reaction was stirred at 25 °C overnight then was concentrated in *vacuo*. The residue was purified by column chromatography on silica gel (petroleum ether: EtOAc, 4:1-2:1) to give **9**.

Yellow solid, isolated yield 59% (28 mg); m.p.: 160.1-160.9 °C; ^{1}H NMR (400 MHz, $CDCl_3$): δ 9.21 (s, 1H), 7.49 (d, J = 7.6 Hz, 1H), 7.38 (d, J = 7.9 Hz, 1H), 7.19 (t, J = 7.6 Hz, 1H), 7.12 (t, J = 7.6 Hz, 1H), 5.53 (d, J = 9.7 Hz, 1H), 4.58 (s, 1H), 4.38 (dd, J = 13.2, 4.4 Hz, 1H), 3.79-3.69 (m, 2H), 3.15-3.08 (m, 1H), 2.90-2.83 (m, 1H), 2.76 (dd, J = 15.2, 3.2 Hz, 1H), 2.29-2.23 (m, 1H), 1.90-1.84 (m, 1H), 1.55 (s,

9H); ^{13}C NMR (101 MHz, $CDCl_3$): δ 156.5, 136.1, 134.1, 126.6, 121.5, 119.2, 117.8, 111.2, 107.9, 80.8, 58.3, 47.6, 39.0, 36.4, 28.4, 21.6; IR (neat): ν 3865, 3404, 1659, 1423, 1226, 1168, 1047, 744, 670 cm^{-1}; HRMS (ESI): m/z [M + H]$^+$ calcd. for $C_{18}H_{25}N_2O_3$: 317.1860; found: 317.1857; $[\alpha]_D^{23}$ = +48.5 (c = 0.25, DCM); The enantiomeric ratio of the product was determined by HPLC analysis (Column Daicel Chiracel IC; ^{i}PrOH/Hexane = 10/90; flow rate = 1.0 mL/min; t_{R1} = 4.94 min, 4.5%; t_{R2} = 7.20 min, 95.5%).

2.6 Synthesis of (−)-harmicine

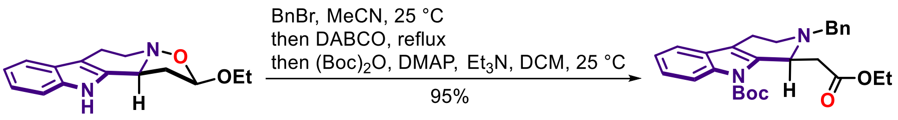

3a 10

***tert*-butyl (*S*)-2-benzyl-1-(2-ethoxy-2-oxoethyl)-1,2,3,4-tetrahydro-9*H*-pyrido[3,4-*b*]indole-9-carboxylate (10)**

To a solution of **3a** (25 mg, 0.10 mmol, 1.0 equiv) in MeCN (0.25 mL) was added BnBr (22 mg, 0.13 mmol, 1.3 equiv). The reaction mixture was stirred at 25 °C for 1 h. Then DABCO (14 mg, 0.13 mmol, 1.3 equiv) was added and the reaction was refluxed for 1 h. The reaction solution was concentrated *in vacuo*. The above residue was dissolved in DCM (1 mL), and Et_3N (59 mg, 0.58 mmol, 6.0 equiv), DMAP (3 mg, 0.02 mmol, 0.2 equiv) and $(Boc)_2O$ (85 mg, 0.39 mmol, 4.0 equiv) were added. The reaction was stirred at 25 °C overnight then was concentrated *in vacuo*. The residue was purified by column chromatography on silica gel (petroleum ether: EtOAc, 5:1) to give **10**.

Yellow oil, isolated yield 95% (43 mg); ^{1}H NMR (400 MHz, $CDCl_3$): δ 8.14 (d, J = 8.2 Hz, 1H), 7.37 (d, J = 7.5 Hz, 1H), 7.24-7.22 (m, 5H), 7.19-7.16 (m, 2H), 4.65 (dd, J = 11.0, 3.4 Hz, 1H), 4.21-4.13 (m, 1H), 4.07-3.98 (m, 1H), 3.65 (dd, J = 28.8, 13.1 Hz, 2H), 3.19 (td, J = 13.1, 6.2 Hz, 1H), 2.95-2.81 (m, 3H), 2.55 (t, J = 11.5 Hz, 1H), 2.40 (dd, J = 15.6, 4.5 Hz, 1H), 1.51 (s, 9H), 1.13 (t, J = 7.3 Hz, 3H); ^{13}C NMR (101 MHz, $CDCl_3$): δ 171.2, 150.1, 139.2, 136.1, 134.7, 129.2, 129.1, 128.1, 127.0, 124.3, 122.7, 117.9, 115.8, 114.7, 84.2, 60.3, 57.3, 55.4, 40.5, 40.4, 28.1, 16.5, 14.2; IR (neat): ν 3855, 3737, 3650, 2360, 2344, 1654, 1559, 1508, 1039, 669 cm^{-1}; HRMS (ESI): m/z [M + H]$^+$ calcd. for $C_{27}H_{33}N_2O_4$: 449.2435; found: 449.2434; $[\alpha]_D^{23}$ = −27.8 (c = 0.67, DCM).

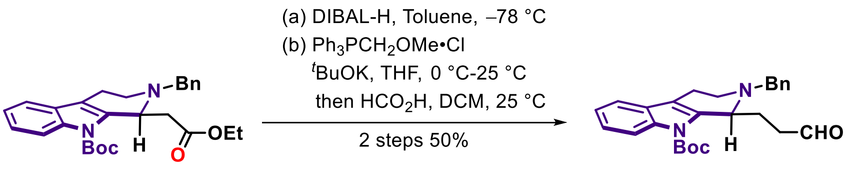

10 11

***tert*-butyl (*S*)-2-benzyl-1-(3-oxopropyl)-1,2,3,4-tetrahydro-9*H*-pyrido[3,4-*b*]indole-9-carboxylate (11)**

Under N_2, to a solution of **10** (41 mg, 0.09 mmol, 1.0 equiv) in dry toluene (0.5 mL) was added DIBAL-H (0.09 mL, 1.0 M in Hexane, 0.09 mmol, 1.0 equiv) at −78 °C. After being stirred for 30 min, the reaction was quenched with addition of aqueous saturated Rochelle salt solution and extracted with EtOAc. The combined organic layers were washed with brine, dried over Na_2SO_4, filtered and

concentrated *in vacuo*. The residue was purified by flash column chromatography on silica gel (petroleum ether:EtOAc, 5:1) to give crude product. ^{t}BuOK (67 mg, 0.60 mmol, 6.5 equiv) was added portion to a suspension of Ph_3PMOM•Cl (189 mg, 0.55 mmol, 6.0 equiv) in THF (5 mL) at 0 °C and the resultant mixture was stirred at 25 °C for 30 min. A solution of crude product in THF (1 mL) was then added dropwise. The reaction mixture was stirred at 25 °C for 16 h then aqueous saturated NH_4Cl (0.5 mL) was added. The aqueous layer was extracted with Et_2O and the combined organic extracts were washed with brine, dried over Na_2SO_4, filtered and concentrated *in vacuo*. Purification via flash column chromatography (petroleum ether: EtOAc, 6:1). The above crude product was dissolved in DCM/HCO_2H (v/v 4:1, 1.8 mL) was stirred for 16 h at 25 °C. $NaHCO_3$ (70 mg) was then added until pH >11 was achieved. The aqueous layer was then extracted with DCM and the combined organic extracts were dried and concentrated *in vacuo*. The residue was purified by column chromatography on silica gel (petroleum ether: EtOAc, 5:1) to give **11**.

Yellow oil, isolated yield 50% for 2 steps (19 mg); ^{1}H NMR (400 MHz, $CDCl_3$): δ 9.72 (s, 1H), 8.09 (d, J = 8.2 Hz, 1H), 7.37 (d, J = 7.2 Hz, 1H), 7.28-7.23 (m, 4H), 7.21-7.16 (m, 3H), 4.01 (dd, J = 9.8, 3.3 Hz, 1H), 3.70 (d, J = 12.7 Hz, 1H), 3.58 (d, J = 12.8 Hz, 1H), 3.17-3.09 (m, 1H), 2.91-2.79 (m, 2H), 2.54-2.41 (m, 2H), 2.29-2.22 (m, 1H), 2.05-1.93 (m, 2H), 1.46 (s, 9H); ^{13}C NMR (101 MHz, $CDCl_3$): δ 202.4, 150.1, 139.1, 136.0, 135.8, 129.4, 129.3, 128.3, 127.2, 124.1, 122.7, 117.9, 115.8, 114.1, 83.7, 57.4, 56.8, 41.3, 28.1, 27.6, 16.8; IR (neat): v 3838, 3673, 3650, 2339, 1699, 1680, 1649, 1556, 1538, 1509, 1549, 1020, 797 cm^{-1}; HRMS (ESI): m/z [M + H]$^+$ calcd. for $C_{26}H_{31}N_2O_3$: 419.2329; found: 419.2325; $[\alpha]_D^{23}$ = −3.9 (c = 1.0, DCM).

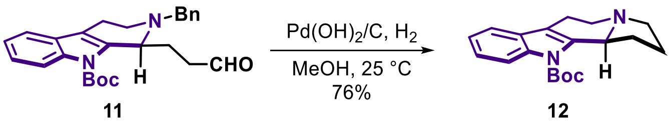

11 12

tert-butyl (*S*)-1,2,3,5,6,11b-hexahydro-11*H*-indolizino[8,7-*b*]indole-11-carboxylate (12)

To a solution of **11** (19 mg, 0.05 mmol) in MeOH (0.5 mL) was added $Pd(OH)_2$/C (20% w/w, 5 mg) and the resultant solution was stirred at 25 °C under an atmosphere of H_2 for 48 h. The reaction mixture was then filtered through a pad of celite and concentrated *in vacuo*. The residue was purified by column chromatography on silica gel (DCM: MeOH, 3:1) to give **12**.

Yellow oil, isolated yield 76% (12 mg); ^{1}H NMR (400 MHz, $CDCl_3$): δ 8.06 (d, J = 8.2 Hz, 1H), 7.38 (d, J = 7.5 Hz, 1H), 7.26-7.18 (m, 2H), 4.70 (t, J = 5.6 Hz, 1H), 3.18 (dt, J = 12.9, 4.3 Hz, 1H), 3.13-3.06 (m, 1H), 2.99-2.94 (m, 1H), 2.90-2.82 (m, 2H), 2.59 (d, J = 16.2 Hz, 1H), 2.56-2.47 (m, 1H), 1.93-1.73 (m, 3H), 1.64 (s, 9H); ^{13}C NMR (101 MHz, $CDCl_3$): δ 150.2, 136.1, 136.1, 129.1, 123.9, 122.6, 117.9, 115.4, 114.3, 83.7, 58.0, 50.3, 44.7, 31.2, 28.2, 23.3, 17.8; IR (neat): v 3906, 3855, 3841, 3747, 3737, 3691, 3651, 3631, 2361, 1654, 1560, 1508, 1458, 1020, 798 cm^{-1}; HRMS (ESI): m/z [M + H]$^+$ calcd. for $C_{19}H_{25}N_2O_2$: 313.1911; found: 313.1909; $[\alpha]_D^{23}$ = −5.1 (c = 1.0, DCM).

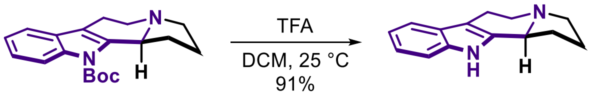

12 (–)-Harmicine (**13**)

(−)-Harmicine (13)

The **12** (7 mg, 0.02 mmol, 1.0 equiv) was dissolved in DCM/TFA (v/v 1:1, 0.2 mL) and stirred for 16 h at 25 °C. Aqueous saturated $NaHCO_3$ was added until pH > 7 was achieved. The aqueous layer was then extracted with DCM and the combined organic extracts were dried and concentrated *in vacuo*. The residue was purified by column chromatography on silica gel (DCM:MeOH, 3:1) to give (−)-harmicine (**13**).

Brown solid, isolated yield 91% (4 mg); m.p.:165.1-166.0 °C; ^1H NMR (400 MHz, $CDCl_3$): δ 7.74 (s, 1H), 7.48 (d, J = 7.6 Hz, 1H), 7.31 (d, J = 7.9 Hz, 1H), 7.16-7.08 (m, 2H), 4.25-4.22 (m, 1H), 3.33 (ddd, J = 13.0, 5.6, 2.4 Hz, 1H), 3.11-3.04 (m, 1H), 2.99-2.82 (m, 3H), 2.67-2.60 (m, 1H), 2.33-2.27 (m, 1H), 1.95-1.84 (m, 3H); ^{13}C NMR (101 MHz, $CDCl_3$): δ 135.9, 135.4, 127.3, 121.4, 119.4, 118.1, 110.7, 107.8, 56.9, 49.2, 45.9, 29.4, 23.4, 17.7; IR (neat): v 3854, 3745, 3676, 3650, 2360, 1700, 1653, 1559, 1507, 1457, 1020, 798 cm^{-1}; HRMS (ESI): m/z $[M + H]^+$ calcd. For $C_{14}H_{17}N_2$: 213.1386; found: 213.1387; $[\alpha]_D^{23}$ = −93.3 (c = 0.02, $CHCl_3$), lit[3]. $[\alpha]_D^{25}$= −92.5 (c = 0.51, $CHCl_3$).

Supplementary Table 1 ^1H NMR data comparison of the synthetic (−)-harmicine

**Lit[3]. Synthetic**	**This work**	**Δδ** (Lit – Our synthetic)
7.74 (brs, 1H)	7.74 (s, 1H)	0
7.49 (d, J = 7.6 Hz, 1H)	7.48 (d, J = 7.6 Hz, 1H)	0.01
7.32 (d, J = 7.7 Hz, 1H)	7.31 (d, J = 7.9 Hz, 1H)	0.01
7.14-7.09 (m, 2H)	7.16-7.08 (m, 2H)	-
4.22-4.26 (m, 1H)	4.25-4.22 (m, 1H)	-
3.33 (ddd, J = 13.0, 5.4, 2.0 Hz, 1H)	3.33 (ddd, J = 13.0, 5.6, 2.4 Hz, 1H)	0
3.09 (ddd, J = 12.8, 10.8, 4.7 Hz, 1H)	3.11-3.04 (m, 1H)	-
3.01-2.83 (m, 3H)	2.99-2.82 (m, 3H)	-
2.65 (tdd, J = 15.5, 4.5, 2.1 Hz, 2H)	2.67-2.60 (m, 1H)	-
2.34-2.24 (m, 1H)	2.33-2.27 (m, 1H)	-
1.98-1.80 (m, 3H)	1.95-1.84 (m, 3H)	-

Supplementary Table 2 ^{13}C NMR data comparison of the synthetic (−)-harmicine

**Lit[3]. Synthetic**	**This work**	**Δδ** (Lit – Our synthetic)
136.0	135.9	0.1
135.4	135.4	0
127.4	127.3	0.1
121.4	121.4	0
119.4	119.4	0
118.1	118.1	0
110.6	110.7	−0.1
107.9	107.8	0.1
57.0	56.9	0.1
49.2	49.2	0
46.0	45.9	0.1

29.4	29.4	0
23.4	23.4	0
17.8	17.7	0.1

2.7 Synthesis of (−)-desbromoarborescidine A

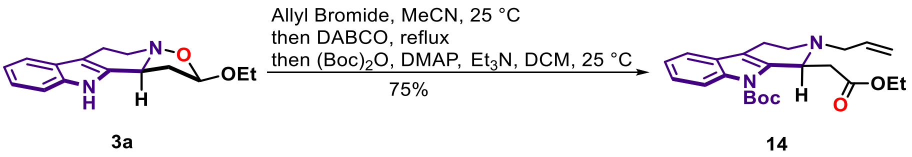

***tert*-butyl (*S*)-2-benzyl-1-(2-ethoxy-2-oxoethyl)-1,2,3,4-tetrahydro-9*H*-pyrido[3,4-*b*]indole-9-carboxylate (14)**

To a solution of **3a** (34 mg, 0.13 mmol, 1.0 equiv) in MeCN (1.3 mL) was added allyl bromide (16 mg, 0.20 mmol, 1.5 equiv). The reaction mixture was stirred at 25 °C for 1 h. Then DABCO (23 mg, 0.20 mg, 1.5 equiv) was added and the reaction was refluxed for 1 h. The reaction solution was concentrated *in vacuo*. The above residue was dissolved in DCM (1.3 mL), and Et_3N (40 mg, 0.39 mmol, 3.0 equiv), DMAP (1.6 mg, 0.013 mmol, 0.1 equiv) and $(Boc)_2O$ (56 mg, 0.26 mmol, 2.0 equiv) were added. The reaction was stirred at 25 °C overnight then was concentrated *in vacuo*. The residue was purified by column chromatography on silica gel (petroleum ether: EtOAc, 10:1) to give **14**.

Brown oil, isolated yield 75% (38 mg); ^{1}H NMR (400 MHz, $CDCl_3$) δ 8.22 (d, J = 8.0 Hz, 1H), 7.44 (d, J = 7.3 Hz, 1H), 7.32 (t, J = 7.5 Hz, 1H), 7.26 (t, J = 7.1 Hz, 1H), 5.99-5.89 (m, 1H), 5.14-5.10 (m, 2H), 4.82 (d, J = 9.8 Hz, 1H), 4.23 (q, J = 6.8 Hz, 2H), 3.31-3.21 (m, 3H), 3.09 (dd, J = 14.1, 5.7 Hz, 1H), 2.92-2.79 (m, 2H), 2.68-2.62 (m, 1H), 2.52 (dd, J = 16.4, 4.2 Hz, 1H), 1.68 (s, 9H), 1.31 (t, J = 7.0 Hz, 3H); ^{13}C NMR (101 MHz, $CDCl_3$) δ 171.2, 150.1, 136.8, 136.1, 134.5, 129.2, 124.2, 122.6, 117.8, 116.9, 115.7, 114.8, 84.1, 60.2, 56.3, 53.9, 41.7, 39.9, 28.2, 16.6, 14.2; IR (neat): v 3862, 3757, 2928, 2341, 1736, 1654, 1542, 1458, 1375, 1021 cm^{-1}; HRMS (ESI): m/z [M + H]$^+$ calcd. for $C_{23}H_{31}N_2O_4$: 399.2278; found: 399.2276; $[\alpha]_D^{23}$ = +7.8 (c = 1.32, DCM).

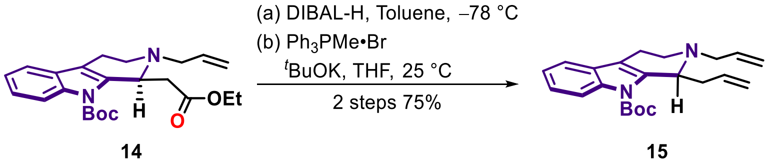

***tert*-butyl (*S*)-1,2-diallyl-1,2,3,4-tetrahydro-9*H*-pyrido[3,4-*b*]indole-9-carboxylate (15)**

Under N_2, to a solution of **14** (33 mg, 0.08 mmol, 1.0 equiv) in dry toluene (0.4 mL) was added DIBAL-H (0.08 mL, 1.0 M in Hexane, 0.08 mmol, 1.0 equiv) at −78 °C. After being stirred for 30 min, the reaction was quenched with addition of aqueous saturated Rochelle salt solution and extracted with EtOAc. The combined organic layers were washed with brine, dried over Na_2SO_4, filtered and concentrated *in vacuo*. The residue was purified by flash column chromatography on silica gel (petroleum ether: EtOAc, 5:1) to give the crude product. To a solution of Ph_3PMe•Br (264 mg, 0.74 mmol, 10 equiv) in dry THF (0.75 mL) was added a solution of ^{t}BuOK (79 mg, 0.70 mmol, 9.5 equiv) in dry THF (0.75 mL) dropwise. The reaction mixture was stirred at 25 °C for 30 min. Then above crude product in dry

THF (0.75 mL) was added and the reaction was stirred at 25 °C for 45 min. The reaction was quenched with aqueous saturated $NaHCO_3$ solution. The separated aqueous phase was extracted with EtOAc. The combined organic layers were washed with brine, dried over Na_2SO_4, filtered and concentrated *in vacuo*. The residue was purified by column chromatography on silica gel (petroleum ether: EtOAc, 10:1) to give **15**.

Colorless oil, isolated yield 75% for 2 steps (23 mg); ^{1}H NMR (400 MHz, $CDCl_3$) δ 8.19 (d, J = 8.1 Hz, 1H), 7.44-7.42 (m, 1H), 7.31-7.27 (m, 1H), 7.26-7.22 (m, 1H), 6.03-5.89 (m, 2H), 5.16-5.06 (m, 4H), 4.46 (dd, J = 10.2, 2.7 Hz, 1H), 3.36-3.21 (m, 3H), 3.08 (dd, J = 14.0, 6.1 Hz, 1H), 2.88-2.79 (m, 1H), 2.62-2.38 (m, 3H), 1.66 (s, 9H); ^{13}C NMR (101 MHz, $CDCl_3$) δ 150.2, 136.9, 136.7, 136.2, 136.1, 129.4, 123.9, 122.5, 117.8, 117.2, 115.7, 115.6, 114.1, 83.6, 56.4, 56.0, 42.0, 38.6, 28.3, 16.9; IR (neat): ν 3849, 3757, 3682, 2975, 2925, 2366, 1704, 1654, 1542, 1055 cm^{-1}; HRMS (ESI): m/z $[M + H]^+$ calcd. for $C_{22}H_{29}N_2O_2$: 353.2224; found: 353.2222; $[\alpha]_D^{23}$ = +3.9 (c = 0.88, DCM).

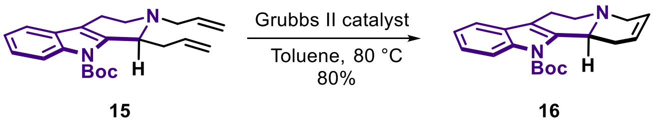

tert-butyl (_S_)-1,6,7,12_b_-tetrahydroindolo[2,3-_a_]quinolizine-12(4_H_)-carboxylate (16)

To a solution of **15** (23 mg, 0.06 mmol, 1.0 equiv) in dry toluene (6.1 mL) was added Grubbs II catalyst (5 mg, 0.006 mmol, 0.1 equiv). The reaction mixture was stirred at 80 °C for 3 h under N_2 atmosphere. The reaction solution was concentrated *in vacuo* and the residue was purified by column chromatography on silica gel (petroleum ether: EtOAc, 4:1) to give **16**.

Brown oil, isolated yield 80% (17 mg); ^{1}H NMR (400 MHz, $CDCl_3$) δ 8.08 (d, J = 7.9 Hz, 1H), 7.44-7.42 (m, 1H), 7.29-7.25 (m, 1H), 7.25-7.21 (m, 1H), 5.84-5.75 (m, 2H), 4.17 (d, J = 9.5 Hz, 1H), 3.49-3.38 (m, 2H), 3.18-3.13 (m, 1H), 2.96-2.88 (m, 1H), 2.80-2.70 (m, 3H), 2.20-2.13 (m, 1H), 1.66 (s, 9H); ^{13}C NMR (101 MHz, $CDCl_3$) δ 150.3, 136.8, 136.7, 129.2, 125.2, 125.1, 123.9, 122.6, 118.0, 116.2, 115.4, 83.7, 56.1, 54.6, 48.7, 30.6, 28.2, 22.1; IR (neat): ν 3847, 3757, 3682, 2926, 2367, 1704, 1654, 1542, 1463, 1064 cm^{-1}; HRMS (ESI): m/z $[M + H]^+$ calcd. for $C_{20}H_{25}N_2O_2$: 325.1911; found: 325.1910; $[\alpha]_D^{23}$ = +7.3 (c = 0.40, DCM).

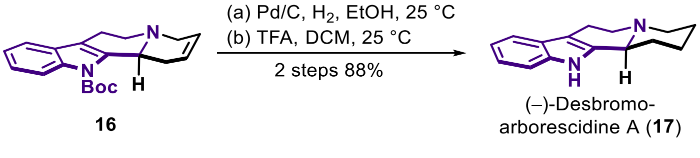

(−)-Desbromoarborescidine A (17)

To a solution of **16** (17 mg, 0.05 mmol) in EtOH (3.4 mL) was added Pd/C (9 mg) and the resultant solution was stirred at 25 ℃ under an atmosphere of H_2 for 8 h. The reaction mixture was then filtered through a pad of celite and concentrated *in vacuo*. The residue was dissolved in DCM/TFA (v/v 4:1, 2 mL) and stirred for 10 h at 0 °C. Aqueous saturated $NaHCO_3$ solution was added until pH > 7 was achieved. The aqueous layer was then extracted with DCM. The combined organic extracts were dried over Na_2SO_4, filtered and concentrated *in vacuo*. The residue was purified by column chromatography on silica gel (DCM: MeOH, 20:1) to give (−)-desbromoarborescidine A (**17**).

White solid, isolated yield 88% (10 mg); m.p.: 148.8-149.6 °C; ^{1}H NMR (400 MHz, $CDCl_3$) δ 7.80 (s, 1H), 7.48 (d, J = 7.6 Hz, 1H), 7.31 (d, J = 7.8 Hz, 1H), 7.15-7.07 (m, 2H), 3.27 (d, J = 10.6 Hz, 1H), 3.11-2.99 (m, 3H), 2.74-2.62 (m, 2H), 2.43-2.37 (m, 1H), 2.09 (dd, J = 12.8, 2.8 Hz, 1H), 1.91 (d, J = 12.4 Hz, 1H), 1.80-1.73 (m, 2H), 1.65-1.46 (m, 2H); ^{13}C NMR (101 MHz, $CDCl_3$) δ 135.9, 134.9, 127.4, 121.3, 119.3, 118.1, 110.7, 108.0, 60.2, 55.6, 53.5, 29.9, 25.6, 24.2, 21.5; IR (neat): ν 3444, 2970, 2928, 1639, 1408, 1263, 1071, 800 cm^{-1}; HRMS (ESI): m/z [M + H]$^{+}$ calcd. for $C_{15}H_{19}N_2$: 227.1543; found: 227.1540; $[\alpha]_D^{23}$ = −78.2 (c = 0.55, $CHCl_3$), for (+)-enantiomer: lit[4]. $[\alpha]_D^{26}$= +78.8 (c = 0.73, $CHCl_3$).

Supplementary Table 3 ^{1}H NMR data comparison of the synthetic (−)-desbromoarborescidine A

**Lit[4]. Synthetic**	**This work**	**Δδ** (Lit – Our synthetic)
7.87 (s, 1H)	7.80 (s, 1H)	0.07
7.48 (d, J = 7.6 Hz, 1H)	7.48 (d, J = 7.6 Hz, 1H)	0
7.31 (d, J = 7.6 Hz, 1H)	7.31 (d, J = 7.8 Hz, 1H)	0
7.17-7.06 (m, 2H)	7.15-7.07 (m, 2H)	-
3.25 (d, J = 10.0 Hz, 1H)	3.27 (d, J = 10.6 Hz, 1H)	−0.02
3.16-2.94 (m, 3H)	3.11-2.99 (m, 3H)	-
2.82-2.60 (m, 2H)	2.74-2.62 (m, 2H),	-
2.40 (td, J = 11.2, 4.0 Hz, 1H)	2.43-2.37 (m, 1H)	-
2.13-2.01 (m, 1H)	2.09 (dd, J = 12.8, 2.8 Hz, 1H)	-
1.95-1.80 (m, 1H)	1.91 (d, J = 12.4 Hz, 1H)	-
1.80-1.67 (m, 2H)	1.80-1.73 (m, 2H)	-
1.66-1.42 (m, 2H)	1.65-1.46 (m, 2H)	-

Supplementary Table 4 ^{13}C NMR data comparison of the synthetic (−)-desbromoarborescidine A

**Lit[4]. Synthetic**	**This work**	**Δδ** (Lit – Our synthetic)
135.9	135.9	0
134.9	134.9	0
127.4	127.4	0
121.2	121.3	−0.1
119.3	119.3	0
118.1	118.1	0
110.7	110.7	0
108.0	108.0	0
60.2	60.2	0
55.6	55.6	0
53.4	53.5	−0.1
29.8	29.9	−0.1
25.6	25.6	0
24.2	24.2	0
21.4	21.5	−0.1

2.8 Synthesis of (−)-arboricine and corynanthe family alkaloids

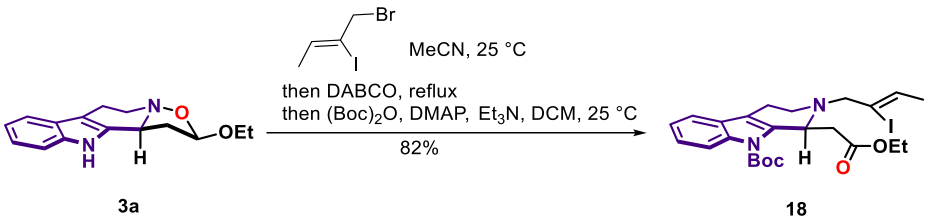

tert-butyl (_S, Z_)-1-(2-ethoxy-2-oxoethyl)-2-(2-iodobut-2-en-1-yl)-1,2,3,4-tetrahydro-9_H_-pyrido[3,4-_b_]indole-9-carboxylate (18)

To a solution of **3a** (994 mg, 3.85 mmol, 1.0 equiv) in MeCN (38.5 mL) was added (*Z*)-1-bromo-2-iodo-2-butene (1.51 g, 5.78 mmol, 1.5 equiv). The reaction mixture was stirred at 25 °C for 10 h. Then DABCO (6.48 mg, 5.78 mmol, 1.5 equiv) was added and the reaction was refluxed for 12 h. The reaction solution was concentrated *in vacuo*. The above residue was dissolved in DCM (38.5 mL), and Et_3N (1.17 g, 11.55 mmol, 3.0 equiv), DMAP (48 mg, 0.39 mmol, 0.1 equiv) and $(Boc)_2O$ (1.6 g, 7.70 mmol, 2.0 equiv) were added. The reaction was stirred at 25 °C overnight then was concentrated *in vacuo*. The residue was purified by column chromatography on silica gel (petroleum ether: EtOAc, 20:1) to give **18**.

Yellow oil, isolated yield 82% (1.70 g); ^{1}H NMR (400 MHz, $CDCl_3$): δ 8.20 (d, J = 8.2 Hz, 1H), 7.43 (d, J = 7.7 Hz, 1H), 7.32 (td, J = 7.3, 1.4 Hz, 1H), 7.26-7.22 (m, 1H), 5.84 (q, J = 6.4 Hz, 1H), 4.74 (dd, J = 11.1, 3.6 Hz, 1H), 4.31-4.16 (m, 2H), 3.49 (q, J = 13.9 Hz, 2H), 3.31-3.23 (m, 1H), 3.03 (dd, J = 14.5, 6.2 Hz, 1H), 2.94 (dd, J = 14.2, 3.9 Hz, 1H), 2.83-2.74 (m, 1H), 2.68 (dd, J = 14.2, 11.2 Hz, 1H), 2.54 (dd, J = 16.6, 5.0 Hz, 1H), 1.82 (d, J = 6.4 Hz, 3H), 1.69 (s, 9H), 1.32 (t, J = 7.2 Hz, 3H); ^{13}C NMR (101 MHz, $CDCl_3$): δ 170.9, 150.1, 136.0, 135.0, 132.1, 129.1, 124.3, 122.7, 117.9, 115.8, 115.0, 109.1, 84.3, 65.1, 60.7, 55.6, 40.4, 40.1, 28.3, 21.7, 16.6, 14.3; IR (neat): v 2994, 1752, 1654, 1554, 1359, 1245, 1105, 679 cm^{-1}; HRMS (ESI): m/z $[M + H]^+$ calcd. for $C_{24}H_{32}N_2O_4I$: 539.1401; found: 539.1400; $[\alpha]_D^{23}$ = −18.5 (c = 0.25, DCM).

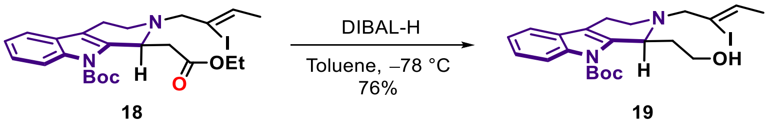

tert-butyl (_S, Z_)-1-(2-hydroxyethyl)-2-(2-iodobut-2-en-1-yl)-1,2,3,4-tetrahydro-9_H_-pyrido[3,4-_b_]indole-9-carboxylate (19)

Under N_2, to a solution of **18** (137 mg, 0.25 mmol, 1.0 equiv) in dry toluene (1.3 mL) was added DIBAL-H (0.5 mL, 1.0 M in Hexane, 0.5 mmol, 2.0 equiv) at −78 °C. After being stirred for 30 min, the reaction was quenched with addition of aqueous saturated Rochelle salt solution and extracted with EtOAc. The combined organic layers were washed with brine, dried over Na_2SO_4, filtered and concentrated *in vacuo*. The residue was purified by column chromatography on silica gel (petroleum ether: EtOAc, 5:1) to give **19**.

Yellow foam, isolated yield 76% (94 mg); ^{1}H NMR (400 MHz, $CDCl_3$) δ 8.13 (d, J = 8.1 Hz, 1H), 7.42 (d, J = 8.1 Hz, 1H), 7.31 (t, J = 7.7 Hz, 1H), 7.24 (t, J = 7.5 Hz, 2H), 5.83 (q, J = 6.4 Hz, 1H), 4.46 (dd,

J = 9.8, 4.4 Hz, 1H), 4.07 (td, J = 9.6, 3.2 Hz, 1H), 3.82 (dt, J = 11.0, 3.2 Hz, 1H), 3.52-3.38 (m, 3H), 3.02 (dd, J = 14.3, 6.2 Hz, 1H), 2.80-2.72 (m, 1H), 2.61 (dd, J = 16.7, 5.4 Hz, 1H), 2.06-1.98 (m, 2H), 1.82 (d, J = 6.3 Hz, 3H), 1.64 (s, 9H); ^{13}C NMR (101 MHz, $CDCl_3$) δ 150.0, 135.8, 135.4, 134.0, 129.1, 124.3, 122.8, 117.9, 115.8, 113.8, 108.1, 83.9, 64.9, 63.1, 59.0, 40.2, 34.9, 28.2, 21.9, 16.4; IR (neat): v 2967, 2368, 1729, 1459, 1262, 1023, 801 cm^{-1}; HRMS (ESI): m/z [M + H]$^+$ calcd. for $C_{22}H_{30}IN_2O_3$: 497.1296; found: 497.1295; $[\alpha]_D^{23}$ = −28.9 (c = 0.12, $CHCl_3$).

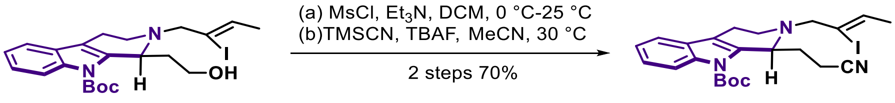

***tert*-butyl (*S*, *Z*)-1-(2-cyanoethyl)-2-(2-iodobut-2-en-1-yl)-1,2,3,4-tetrahydro-9*H*-pyrido[3,4-*b*]indole-9-carboxylate (20)**

To a solution of **19** (91 mg, 0.18 mmol, 1.0 equiv) and Et_3N (29 µL, 0.20 mmol, 1.1 equiv) in dry DCM (0.45 mL) was dropwise added MsCl (15 µL, 0.19 mmol, 1.04 equiv) in dry DCM (0.45 mL) at 0 °C. After being stirred at 25 °C for 1 h, the reaction was quenched with H_2O and extracted with DCM. The combined organic layers were washed with brine, dried over Na_2SO_4, filtered and concentrated *in vacuo*. The above residue was dissolved in MeCN (1.8 mL) followed by adding TMSCN (45 µL, 0.36 mmol, 2.0 equiv) and TBAF (0.54 mL, 1.0 M in THF, 0.54 mmol, 3.0 equiv) under N_2. The reaction mixture was stirred at 30 °C for 20 h and concentrated *in vacuo*. The residue was purified by column chromatography on silica gel (petroleum ether: EtOAc, 80:1) to give **20**.

White solid, isolated yield 70% for 2 steps (65 mg); m.p.: 138.8-139.6 °C; ^{1}H NMR (400 MHz, $CDCl_3$) δ 8.09 (d, J = 8.0 Hz, 1H), 7.43 (d, J = 7.5 Hz, 1H), 7.32 (t, J = 7.6 Hz, 1H), 7.24 (t, J = 7.3 Hz, 1H), 5.78 (q, J = 6.3 Hz, 1H), 4.16 (d, J = 10.9 Hz, 1H), 3.46 (d, J = 13.6 Hz, 1H), 3.36 (d, J = 13.6 Hz, 1H), 3.19-3.11 (m, 1H), 3.00-2.87 (m, 2H), 2.82-2.74 (m, 1H), 2.60-2.49 (m, 2H), 2.32-2.25 (m, 1H), 1.95-1.80 (m, 1H), 1.82 (d, J = 6.4 Hz, 3H), 1.68 (s, 9H); ^{13}C NMR (101 MHz,$CDCl_3$) δ 150.1, 135.6, 135.3, 133.1, 129.1, 124.3, 122.8, 120.6, 118.0, 115.8, 114.7, 109.7, 84.1, 64.7, 56.3, 40.6, 29.9, 28.3, 21.8, 16.8, 15.2; IR (neat): v 3753, 2928, 2373, 1730, 1649, 1370, 1023, 800 cm^{-1}; HRMS (ESI): m/z [M + H]$^+$ calcd. for $C_{23}H_{29}IN_3O_2$: 506.1299; found: 506.1299; $[\alpha]_D^{23}$ = −38.0 (c = 0.17, $CHCl_3$).

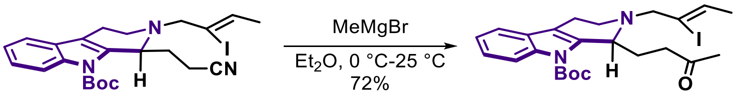

***tert*-butyl (*S*, *Z*)-2-(2-iodobut-2-en-1-yl)-1-(3-oxobutyl)-1,2,3,4-tetrahydro-9*H*-pyrido[3,4-*b*]indole-9-carboxylate (21)**

To a solution of **20** (52 mg, 0.1 mmol, 1.0 equiv) in dry Et_2O (0.5 mL) was dropwise added MeMgBr (0.1 mL, 3.0 M in ether 0.3 mmol, 3.0 equiv) in dry Et_2O (0.5 mL) under N_2 atmosphere at 0 °C. After being stirred at 25 °C for 4 h, the reaction was quenched with aqueous saturated $NaHCO_3$ and extracted with with Et_2O. The combined organic layers were washed with brine, dried over Na_2SO_4, filtered and concentrated *in vacuo*. The residue was purified by column chromatography on silica gel (petroleum ether: EtOAc, 20:1) to give **21**.

Colorless oil, isolated yield 72% (38 mg); ^{1}H NMR (400 MHz, $CDCl_3$): δ 8.13 (d, J = 8.1 Hz, 1H), 7.41 (d, J = 7.8 Hz, 1H), 7.30-7.20 (m, 2H), 5.77 (q, J = 6.3 Hz, 1H), 4.12 (dd, J = 10.8, 2.7 Hz, 1H), 3.45 (q, J = 13.6 Hz, 2H), 3.26-3.18 (m, 1H), 3.13-3.04 (m, 1H), 2.94 (dd, J = 14.3, 6.0 Hz, 1H), 2.81-2.72 (m, 1H), 2.66-2.58 (m, 1H), 2.53 (dd, J = 16.6, 5.1 Hz, 1H), 2.21 (s, 3H), 2.12-2.06 (m, 1H), 1.96-1.86 (m, 1H), 1.82 (d, J = 6.3 Hz, 3H), 1.65 (s, 9H); ^{13}C NMR (101 MHz, $CDCl_3$): δ 209.4, 150.1, 136.7, 135.8, 132.6, 129.3, 124.0. 122.6, 117.8, 115.7, 114.1, 110.2, 83.6, 64.9, 56.6, 41.9, 40.8, 30.2, 28.2, 27.8, 21.7, 16.9; IR (neat): v 2920, 2876, 1723, 1617, 1459 cm^{-1}; HRMS (ESI): m/z $[M + H]^+$ calcd. for $C_{24}H_{32}IN_2O_3$: 523.1452; found: 523.1451; $[\alpha]_D^{23}$ = −28.0 (c = 0.1, $CHCl_3$).

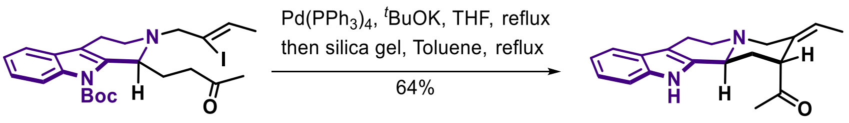

21

(−)-Arboricine (**22**)

(−)-Arboricine (22)

Under N_2, to a solution of **21** (28 mg, 0.053 mmol, 1.0 equiv) and phenol (15 mg, 0.16 mmol, 3.0 equiv) in dry THF (1.5 mL) was added ^{t}BuOK (16 mg, 0.13 mmol, 2.5 equiv) and $Pd(PPh_3)_4$ (6 mg, 0.0053 mmol, 0.1 equiv) at 0 °C. The reaction mixture was refluxed for 1 h, cooled to 25 °C, diluted with DCM and washed with saturated aqueous $NaHCO_3$ and 1M aqueous NaOH. The organic layers were washed with brine, dried over Na_2SO_4, filtered and concentrated *in vacuo*. The crude product was used in the next step without further purification. The above residue was dissolved in toluene (5.0 mL) followed by adding silica gel (200 mg). The reaction mixture was refluxed for 1 h, cooled to 25 °C, filtered and washed with DCM. The residue was concentrated *in vacuo* and purified by column chromatography on silica gel (petroleum ether: EtOAc, 1:1) to give (−)-arboricine (**22**).

Pale yellow solid, isolated yield 64% for 2 steps (10 mg); m.p.: 118.2-118.9 °C; 1H NMR (400 MHz, $CDCl_3$): δ 8.00 (s, 1H), 7.46 (d, J = 7.6 Hz, 1H), 7.33 (d, J = 8.2 Hz, 1H), 7.15 (t, J = 7.2 Hz, 1H), 7.09 (t, J = 7.1 Hz, 1H), 5.80 (q, J = 7.1 Hz, 1H), 3.72 (d, J = 2.9 Hz, 1H), 3.63 (d, J = 9.0 Hz, 1H), 3.33 (d, J = 12.4 Hz, 1H), 3.09-2.97 (m, 3H), 2.76-2.58 (m, 3H), 2.18 (s, 3H), 1.81 (d, J = 6.6 Hz, 3H), 1.64-1.56 (m, 1H); ^{13}C NMR (101 MHz, $CDCl_3$): δ 208.7, 136.0, 134.5, 132.2, 127.2, 124.3, 121.2, 119.2, 118.0, 110.8, 108.0, 61.6, 56.7, 52.4, 48.3, 30.0, 28.2, 21.7, 13.5; IR (neat): v 3405, 2967, 2860, 1708, 1263, 1023, 800 cm^{-1}; HRMS (ESI): m/z $[M + H]^+$ calcd. for $C_{19}H_{23}N_2O$: 295.1805; found: 295.1806; $[\alpha]_D^{23}$ = −245.0 (c = 0.20, $CHCl_3$), lit[5]. $[\alpha]_D^{20}$= −281 (c = 0.23, $CHCl_3$).

Supplementary Table 5 ^{1}H NMR data comparison of the synthetic (−)-arboricine

**Lit[5]. Synthetic**	**This work**	**Δδ** (Lit – Our synthetic)
7.93 (brs, 1H)	8.00 (s, 1H)	−0.07
7.48 (d, J = 7.6 Hz, 1H)	7.46 (d, J = 7.6 Hz, 1H)	0.02
7.34 (d, J = 7.9 Hz, 1H)	7.33 (d, J = 8.2 Hz, 1H)	0.01
7.1-7.2 (m, 2H)	7.15 (t, J = 7.2 Hz, 1H)	-
	7.09 (t, J = 7.1 Hz, 1H)	-
5.79 (q, J = 6.6 Hz, 1H)	5.80 (q, J = 7.1 Hz, 1H)	−0.01
3.75 (d, J = 5.1 Hz, 1H)	3.72 (d, J = 2.9 Hz, 1H)	0.03

3.63 (d, *J* = 11.8 Hz, 1H)	3.63 (d, *J* = 9.0 Hz, 1H)	0
3.33 (d, *J* = 12.2 Hz, 1H)	3.33 (d, *J* = 12.4 Hz, 1H)	0
2.97-3.09 (m, 3H)	3.09-2.97 (m, 3H)	-
2.60-2.80 (m, 3H)	2.76-2.58 (m, 3H)	-
2.21 (s, 3H)	2.18 (s, 3H)	0.03
1.84 ((dd, *J* = 6.6, 1.7 Hz, 3H)	1.81 (d, *J* = 6.6 Hz, 3H)	0.03
1.60 (m, 1H)	1.64-1.56 (m, 1H)	-

Supplementary Table 6 ^{13}C NMR data comparison of the synthetic (−)-arboricine

**Lit[5]. Synthetic**	**This work**	**Δδ** (Lit – Our synthetic)
208.9	208.7	0.2
136.0	136.0	0
134.6	134.5	0.1
132.2	132.2	0
127.1	127.2	−0.1
124.1	124.3	−0.2
121.0	121.2	−0.2
119.0	119.2	−0.2
117.9	118.0	−0.1
110.7	110.8	−0.1
107.8	108.0	−0.2
61.6	61.6	0
56.7	56.7	0
52.4	52.4	0
48.2	48.3	−0.1
29.9	30.0	−0.1
28.1	28.2	−0.1
21.6	21.7	−0.1
13.3	13.5	−0.2

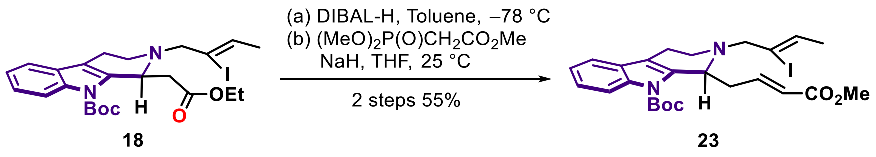

***tert*-butyl (*S*)-2-((*Z*)-2-iodobut-2-en-1-yl)-1-((*E*)-4-methoxy-4-oxobut-2-en-1-yl)-1,2,3,4-tetrahydro-9*H*-pyrido[3,4-*b*]indole-9-carboxylate (23)**

Under N_2, to a solution of **18** (1.50 g, 2.79 mmol, 1.0 equiv) in dry toluene (28 mL) at −78°C was added dropwise DIBAL-H (3.35 mL, 1.0 M in Hexane, 3.35 mmol, 1.2 equiv) and the mixture was stirred for 30 min at −78°C. The reaction was quenched with MeOH and warmed to 25 °C within 30 min. Rochelle salts were added and the mixture was stirred for 1 h. The aqueous layer was extracted with EtOAc. The combined organic layers were washed with brine, dried over Na_2SO_4 and concentrated in *vacuo*. Crude

product was used in the next step without further purification. To NaH (279 mg, 60% in mineral oil, 6.98 mmol, 2.5 equiv) in dry THF (25 mL) was added $(MeO)_2P(O)CH_2CO_2Me$ (1.27 g, 6.98 mmol, 2.5 equiv) and the mixture was stirred for 30 min at 25 °C, then a solution of above crude product in dry THF (10 mL) was added. After stirring for 40 min at 25 °C, the reaction was quenched with saturated aqueous $NaHCO_3$. The aqueous layer was extracted with EtOAc. The combined organic layers were washed with brine, dried over Na_2SO_4 and concentrated in *vacuo*. The residue was purified by column chromatography on silica gel (petroleum ether: EtOAc, 20:1) to give **23**.

Yellow oil, isolated yield 55% for 2 steps (846 mg); ^{1}H NMR (400 MHz, $CDCl_3$): δ 8.16 (d, J = 8.1 Hz, 1H), 7.44 (d, J = 7.4 Hz, 1H), 7.37-7.22 (m, 3H), 5.94 (d, J = 15.7 Hz, 1H), 5.84 (q, J = 6.5 Hz, 1H), 4.40 (d, J = 9.7 Hz, 1H), 3.75 (s, 3H), 3.48 (q, J = 13.8 Hz, 2H), 3.28-3.20 (m, 1H), 3.03-2.98 (m, 1H), 2.85-2.74 (m, 2H), 2.60-2.52 (m, 2H), 1.82 (d, J = 6.0 Hz, 3H), 1.66 (s, 9H); ^{13}C NMR (101 MHz, $CDCl_3$): δ 166.9, 150.0, 148.0, 135.8, 135.6, 132.3, 129.2, 124.1, 122.6, 121.6, 117.8, 115.7, 114.5, 109.4, 83.8, 64.9, 56.1, 51.3, 40.9, 37.0, 28.2, 21.7, 17.0; IR (neat): ν 3452, 2945, 1774, 1605, 1547, 1354, 1209, 1009, 670 cm^{-1}; HRMS (ESI): m/z $[M + H]^+$ calcd. for $C_{25}H_{32}N_2O_4I$: 551.1401; found: 551.1397; $[\alpha]_D^{23}$ = +9.5 (c = 0.30, DCM).

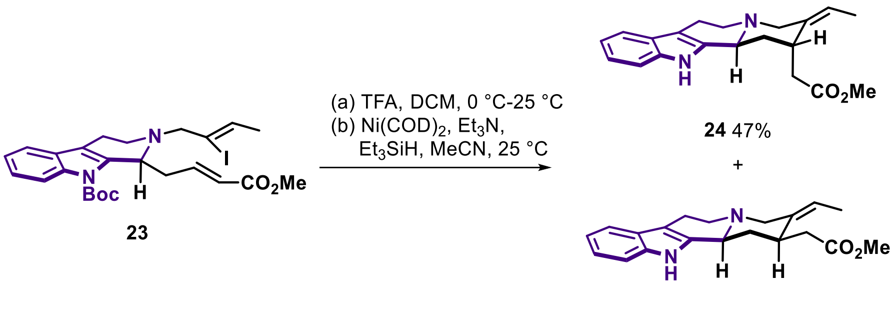

methyl 2-((2*S*, 12b*S*, *E*)-3-ethylidene-1,2,3,4,6,7,12,12b-octahydroindolo[2,3-*a*]quinolizin-2-yl)acetate (24) and **Methyl 2-((2*R*, 12b*S*, *E*)-3-ethylidene-1,2,3,4,6,7,12,12b-octahydroindolo[2,3-*a*]quinolizin-2-yl)acetate (25)**

To a solution of **23** (846 mg, 1.54 mmol, 1.0 equiv) in DCM (15.4 mL) was added TFA (7.8 mL) at 0 °C. The reaction mixture was stirred at 25 °C for 4 h. The reaction mixture was quenched with saturated aqueous $NaHCO_3$. The aqueous layer was extracted with EtOAc. The combined organic layers were washed with brine, dried over Na_2SO_4 and concentrated in *vacuo*. At 25 °C, to a suspension of $Ni(COD)_2$ (1.27 g, 4.62 mmol, 3.0 equiv) in MeCN (7.4 mL) was added a solution of the above residue and Et_3N (623 mg, 6.16 mmol, 4.0 equiv) in MeCN (94 mL) in the glovebox. The resulting solution was stirred for 30 min before adding Et_3SiH (532 mg, 4.62 mmol, 3.0 equiv). The reaction was stirred at 25 °C for 2 h, and was quenched by saturated aqueous $NaHCO_3$ solution and extracted with EtOAc. The combined organic layers were washed with brine, dried over Na_2SO_4 and concentrated *in vacuo*. The residue was purified by column chromatography on silica gel (petroleum: EtOAc, 1:1) to give **24** and **25**.

24: Yellow solid, isolated yield 47% (234 mg); m.p.: 64.7-65.0 °C; ^{1}H NMR (400 MHz, $CDCl_3$): δ 7.73 (s, 1H), 7.47 (d, J = 7.5 Hz, 1H), 7.32 (d, J = 8.0 Hz, 1H), 7.15-7.07 (m, 2H), 5.52 (q, J = 6.4 Hz, 1H), 3.73 (s, 3H), 3.62 (d, J = 14.3 Hz, 1H), 3.51-3.45 (m, 1H), 3.22 (s, 2H), 3.11-2.97 (m, 2H), 2.76-2.65 (m, 3H), 2.58 (dd, J = 15.0, 7.2 Hz, 1H), 2.11 (d, J = 13.4 Hz, 1H), 1.89-1.81 (m, 1H), 1.66 (d, J = 6.8 Hz, 3H); ^{13}C NMR (101 MHz, $CDCl_3$): δ 172.7, 135.9, 134.9, 134.1, 127.1, 121.6, 121.1, 119.1, 117.9, 110.6,

108.3, 59.7, 54.8, 52.5, 51.6, 36.5, 33.9, 30.7, 21.5, 12.4; IR (neat): *ν* 3556, 2902, 1765, 1556, 1435, 1269, 1164, 1009, 909, 679 cm^{-1}; HRMS (ESI): m/z [M + H]$^+$ calcd. for $C_{20}H_{25}N_2O_4$: 325.1911; found: 325.1909; $[\alpha]_D^{23}$ = −36.4 (*c* = 0.88, MeOH).

25: Yellow solid, isolated yield 39% (195 mg); m.p.: 88.5-89.1 °C; ^{1}H NMR (400 MHz, $CDCl_3$): *δ* 8.96 (s, 1H), 7.46 (d, *J* = 7.4 Hz, 1H), 7.38 (d, *J* = 7.7 Hz, 1H), 7.16-7.08 (m, 2H), 5.56 (q, *J* = 5.6 Hz, 1H), 4.34 (s, 1H), 3.66 (s, 3H), 3.60 (d, *J* = 12.3 Hz, 1H), 3.27-3.24 (m, 1H), 3.16-3.09 (m, 3H), 3.02-2.98 (m, 1H), 2.71-2.68 (m, 1H), 2.29-2.16 (m, 4H), 1.63 (d, *J* = 6.6 Hz, 3H); ^{13}C NMR (101 MHz, $CDCl_3$): *δ* 173.4, 136.0, 133.9, 132.2, 127.0, 122.8, 121.6, 119.4, 117.9, 111.2, 106.8, 53.2, 52.7, 51.7, 50.8, 37.0, 30.7, 30.5, 17.6, 12.7; IR (neat): *ν* 3547, 2945, 1714, 1645, 1369, 1245, 1009, 945, 671 cm^{-1}; HRMS (ESI): m/z [M + H]$^+$ calcd. for $C_{20}H_{25}N_2O_4$: 325.1911; found: 325.1910; $[\alpha]_D^{23}$ = +15.8 (*c* = 0.40, DCM).

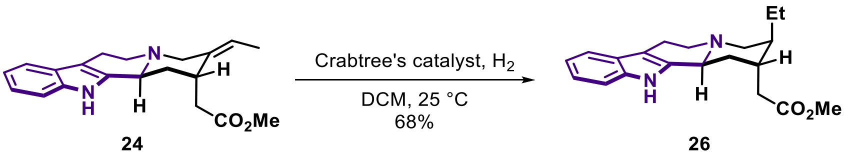

methyl 2-((2*S*, 3*S*, 12b*S*)-3-ethyl-1,2,3,4,6,7,12,12b-octahydroindolo[2,3-*a*]quinolizin-2-yl)acetate (26)

A mixture of **24** (117 mg, 0.36 mmol, 1.0 equiv) and Crabtree's catalyst (43.5 mg, 0.054 mmol, 0.15 equiv) in DCM (18 mL) was stirred at 25 °C under an atmosphere of H_2 for 24 h. The mixture was concentrated *in vacuo*. The residue was purified by column chromatography on silica gel (petroleum ether: EtOAc, 4:1-1:1) to give **26**.

Yellow oil, isolated yield 68% (80 mg); ^{1}H NMR (400 MHz, $CDCl_3$): δ 8.40 (s, 1H), 7.47 (d, *J* = 7.6 Hz, 1H), 7.40 (d, *J* = 7.9 Hz, 1H), 7.17 (t, *J* = 7.0 Hz, 1H), 7.12 (t, *J* = 8.3 Hz, 1H), 4.27 (s, 1H), 3.72 (s, 3H), 3.32 (dd, *J* = 12.6, 5.9 Hz, 1H), 3.16-3.08 (m, 1H), 3.04-2.95 (m, 1H), 2.89 (dd, *J* = 11.8, 3.2 Hz, 1H), 2.72-2.54 (m, 3H), 2.34-2.20 (m, 2H), 1.72-1.66 (m, 1H), 1.87-1.80 (m, 1H), 1.52-1.46 (m, 2H), 1.17-1.12 (m, 1H), 0.84 (t, *J* = 7.1 Hz, 3H); ^{13}C NMR (101 MHz, $CDCl_3$): *δ* 173.5, 136.2, 131.1, 127.0, 121.8, 119.5, 118.0, 111.4, 107.0, 54.6, 51.7, 51.6, 50.7, 40.4, 36.6, 32.1, 31.8, 23.7, 17.7, 11.0; IR (neat): *ν* 3554, 2952, 1755, 1645, 1515, 1359, 1239, 1009, 671 cm^{-1}; HRMS (ESI): m/z [M + H]$^+$ calcd. for $C_{20}H_{27}N_2O_2$: 327.2068; found: 327.2067; $[\alpha]_D^{23}$ = −11.9 (*c* = 0.10, DCM).

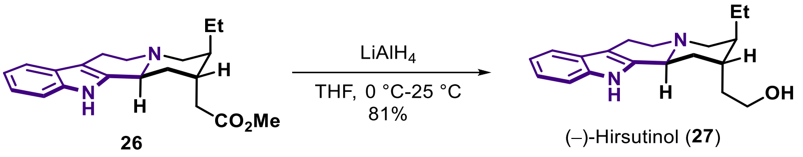

(−)-Hirsutinol (27)

To a solution of **26** (49 mg, 0.15 mmol, 1.0 equiv) in THF (3 mL) was added $LiAlH_4$ (0.15 mL, 1.0 M in THF, 0.15 mmol, 1.0 equiv) at 0 °C. After being stirred at 0 °C for 1 h, the reaction mixture was quenched with H_2O. The aqueous layer was extracted with EtOAc. The combined organic layers were washed with brine, dried over Na_2SO_4 and concentrated in *vacuo*. The residue was purified by column chromatography on silica gel (DCM: MeOH, 20:1-10:1) to give (−)-hirsutinol (**27**).

Yellow oil, isolated yield 81% (36 mg); ^{1}H NMR (400 MHz, $CDCl_3$): δ 8.42 (s, 1H), 7.48 (d, J = 7.3 Hz, 1H), 7.34 (d, J = 7.5 Hz, 1H), 7.15-7.07 (m, 2H), 4.05 (s, 1H), 3.79-3.72 (m, 2H), 3.18-3.13 (m, 1H), 3.01-2.93 (m, 2H), 2.74 (dd, J = 11.5, 3.4 Hz, 1H), 2.66-2.61 (m, 1H), 2.57 (dd, J = 11.5, 7.4 Hz, 1H), 2.24-2.18 (m, 1H), 1.91-1.84 (m, 1H), 1.80-1.73 (m, 1H), 1.56-1.47 (m, 3H), 1.43-1.33 (m, 1H), 0.86 (t, J = 7.4 Hz, 3H); ^{13}C NMR (101 MHz, $CDCl_3$): δ 135.9, 133.7, 127.5, 121.2, 119.3, 117.9, 110.9, 107.6, 60.6, 54.5, 52.2, 51.8, 41.5, 35.2, 32.4, 31.6, 24.3, 18.8, 11.7; IR (neat): ν 3452, 2954, 1756, 1652, 1435, 1320, 1215, 1009, 671 cm^{-1}; HRMS (ESI): m/z [M + H]$^+$ calcd. for $C_{19}H_{27}N_2O$: 299.2118; found: 299.2118; $[\alpha]_D^{23}$ = -55.1 (c = 0.15, $CHCl_3$), for (+)-enantiomer: lit[6]. $[\alpha]_D^{16.5}$= +58.2 (c = 0.2, $CHCl_3$).

Supplementary Figure 2: ^{1}H NMR data comparison of the synthetic (−)-hirsutinol

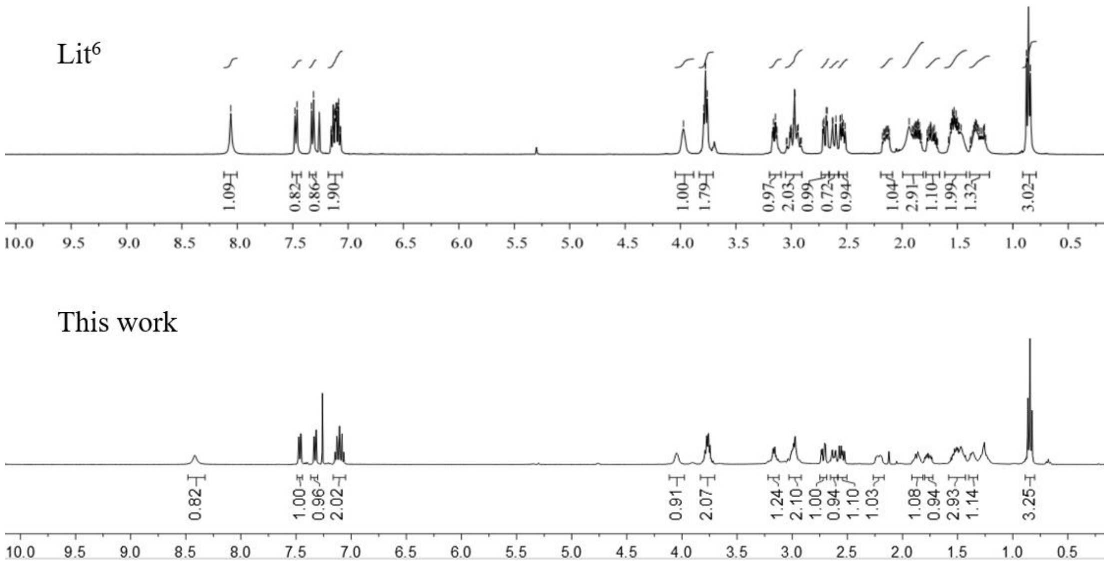

Supplementary Figure 3: ^{13}C NMR Data comparison of the synthetic (−)-hirsutinol

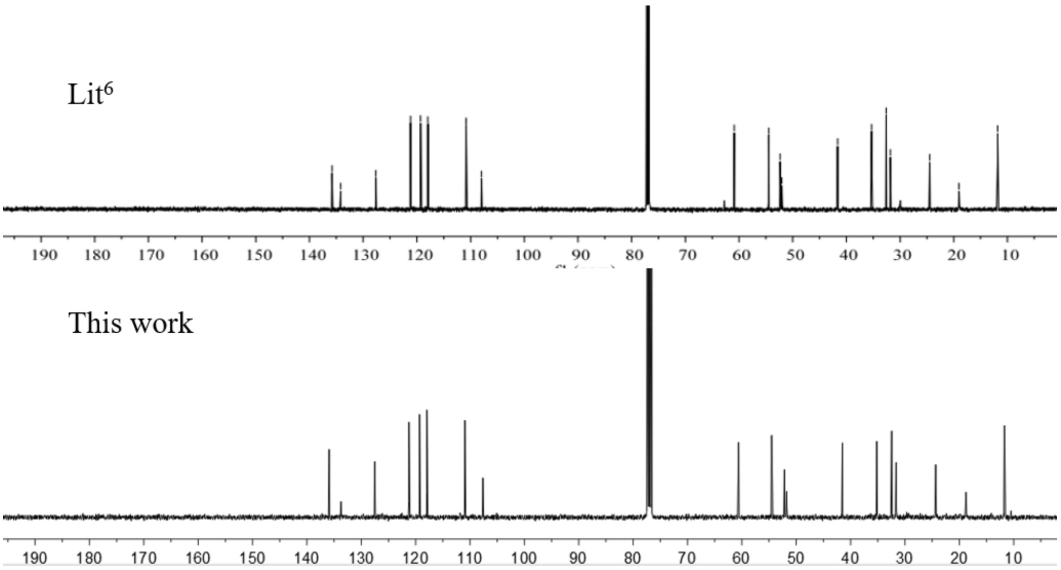

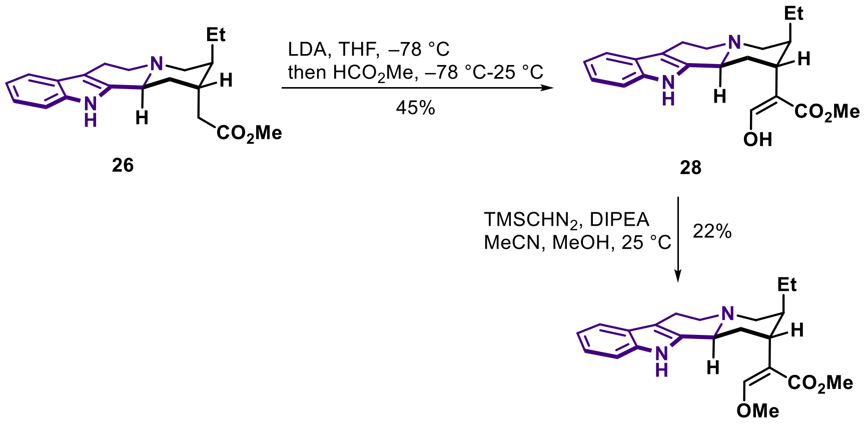

(–)-Hirsutine (**29**)

(−)-Hirsutine (29)

Under N_2, to a solution of ester **26** (72 mg, 0.22 mmol, 1.0 equiv) in dry THF (6 mL) was added LDA (1.0 mL, 1.32 M in THF, 1.32 mmol, 6.0 equiv) dropwise at −78 ºC. After 30 min, methyl formate (1.05 g, 17.6 mmol, 80.0 equiv) was added. The resulting mixture was then warmed to 25 °C and stirred overnight. The reaction was quenched with saturated aqueous NH_4Cl and extracted with EtOAc. The combined organic layers were washed with brine, dried over Na_2SO_4 and concentrated in *vacuo*. The residue was purified by column chromatography on silica gel (petroleum ether: EtOAc, 1:1) to give enol (35 mg, 45%) and recycled the started material (36 mg, 50%). The above residue was dissolved in MeCN (1.8 mL) and MeOH (0.2 mL), and $TMSCH_2N_2$ (60 µL, 2.0 M in Hexane, 0.12 mmol, 1.2 equiv) and DIPEA (16 mg, 0.12 mmol, 1.2 equiv) were added. The reaction was stirred at 25 °C for 24 h then was concentrated in *vacuo*. The residue was purified by column chromatography on silica gel (petroleum ether: Acetone, 4:1-1:1) to give (−)-hirsutine (**29**).

Yellow solid, isolated yield 10% for 2 steps (8 mg); m.p.: 57.2-57.9 °C; ^{1}H NMR (400 MHz, $CDCl_3$): δ 7.99 (s, 1H), 7.51 (d, J = 7.5 Hz, 1H), 7.40 (d, J = 7.8 Hz, 1H), 7.33 (s, 1H), 7.19-7.10 (m, 2H), 4.50 (s, 1H), 3.77 (s, 3H), 3.69 (s, 3H), 3.35-3.31 (m, 2H), 3.08-2.99 (m, 1H), 2.84 (d, J = 12.2 Hz, 1H), 2.63-2.58 (m, 1H), 2.51-2.45 (m, 1H), 2.42 (t, J = 10.5 Hz, 1H), 2.25-2.20 (m, 2H), 2.02-1.99 (m, 1H), 1.32-1.29 (m, 1H), 0.90-0.86 (m, 1H), 0.77-0.76 (m, 3H); ^{13}C NMR (101 MHz, $CDCl_3$): δ 168.9, 159.7, 135.9, 132.9, 127.8, 121.3, 119.3, 117.9, 111.6, 111.1, 107.8, 61.5, 54.2, 51.34, 51.26, 50.6, 38.9, 34.8, 31.7, 24.2, 16.9, 11.3; IR (neat): ν 2933, 1704, 1658, 1563, 1544, 1461, 1256, 670 cm^{-1}; HRMS (ESI): m/z $[M + H]^+$ calcd. for $C_{22}H_{29}N_2O_3$: 369.2173; found: 369.2169; $[\alpha]_D^{23}$ = −61.6 (c = 0.15, $CHCl_3$), for (+)-enantiomer: lit[7]. $[\alpha]_D^{22}$= +68.47 (c = 0.32, $CHCl_3$).

Supplementary Table 7 ^{1}H NMR data comparison of the synthetic (−)-hirsutine

**Lit[8]. Synthetic**	**This work**	**Δδ** (Lit – Our synthetic)
7.95 (brs, 1H)	7.99 (s, 1H)	−0.04
7.49 (d, J = 7.5 Hz, 1H)	7.51 (d, J = 7.5 Hz, 1H)	−0.02
7.39 (d, J = 8.0 Hz, 1H)	7.40 (d, J = 7.8 Hz, 1H)	−0.01
7.32 (s, 1H)	7.33 (s, 1H)	−0.01
7.16 (t, J = 7.0 Hz, 1H)	7.19-7.10 (m, 2H)	-

7.10 (t, J = 7.0 Hz, 1H)		
4.51 (brs, 1H)	4.50 (s, 1H)	0.01
3.77 (s, 3H)	3.77 (s, 3H)	0
3.69 (s, 3H)	3.69 (s, 3H)	0
3.38-3.28 (m, 1H)	3.35-3.31 (m, 2H)	-
3.07-3.00 (m, 1H)	3.08-2.99 (m, 1H)	-
2.81 (d, J = 10.0 Hz, 1H)	2.84 (d, J = 12.2 Hz, 1H)	−0.03
2.59 (d, J = 18.0 Hz, 1H)	2.63-2.58 (m, 1H)	-
2.49 (brs, 1H)	-	-
2.37 (t, J = 10.0 Hz, 1H)	2.42 (t, J = 10.5 Hz, 1H)	−0.05
2.17 (d, J = 9.5 Hz, 2H)	2.25-2.20 (m, 2H)	-
1.98 (d, J = 13.5 Hz, 1H)	2.02-1.99 (m, 1H)	-
1.34-1.26 (m, 1H)	1.32-1.29 (m, 1H)	-
0.95-0.82 (m, 1H)	0.90-0.86 (m, 1H)	-
0.76 (t, J = 6.6 Hz, 3H)	0.77-0.76 (m, 3H)	-

Supplementary Table 8 ^{13}C NMR data comparison of the synthetic (−)-hirsutine

**Lit[8]. Synthetic**	**This work**	**Δδ** (Lit – Our synthetic)
169.0	168.9	0.1
159.8	159.7	0.1
135.9	135.9	0
133.1	132.9	0.2
127.9	127.8	0.1
121.3	121.3	0
119.3	119.3	0
118.0	117.9	0.1
111.7	111.6	0.1
111.2	111.1	0.1
107.8	107.8	0
61.5	61.5	0
54.2	54.2	0
51.4	51.34	0.06
51.3	51.26	0.04
50.7	50.6	0.1
39.0	38.9	0.1
34.9	34.8	0.1
31.7	31.7	0
24.3	24.2	0.1
17.0	16.9	0.1
11.4	11.3	0.1

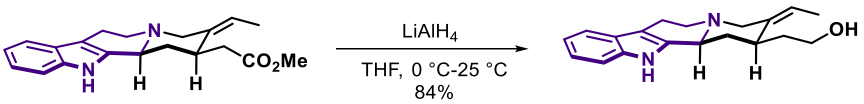

25 (–)-Geissoschizol (**30**)

(−)-Geissoschizol (30)

To a solution of **25** (16 mg, 0.05 mmol, 1.0 equiv) in THF (1.0 mL) was added $LiAlH_4$ (0.05 mL, 1.0 M in THF, 0.10 mmol, 1.0 equiv) at 0 °C. After being stirred at 0 °C for 1 h, the reaction mixture was quenched with H_2O. The aqueous layer was extracted with EtOAc. The combined organic layers were washed with brine, dried over Na_2SO_4 and concentrated in *vacuo*. The residue was purified by column chromatography on silica gel (DCM: MeOH, 20:1) to give (−)-geissoschizol (**30**).

White solid, isolated yield 84% (12.5 mg); m.p.: 91.7-92.2 °C; ^{1}H NMR (400 MHz, $CDCl_3$): δ 8.32 (s, 1H), 7.48 (d, J = 7.6 Hz, 1H), 7.34 (d, J = 7.8 Hz, 1H), 7.16-7.08 (m, 2H), 5.54 (q, J = 6.7 Hz, 1H), 4.22 (s, 1H), 3.66-3.52 (m, 3H), 3.28-3.23 (m, 1H), 3.12-2.96 (m, 4H), 2.69-2.66 (m, 1H), 2.34-2.29 (m, 1H), 2.21-2.16 (m, 1H), 1.65 (dd, J = 6.8, 1.2 Hz, 3H), 1.54-1.48 (m, 2H); ^{13}C NMR (101 MHz, $CDCl_3$): δ 137.1, 135.9, 134.5, 127.7, 121.4, 120.2, 119.4, 118.0, 110.9, 107.7, 61.9, 54.1, 53.4, 51.4, 36.1, 33.1, 31.6, 18.3, 12.9; IR (neat): v 3314, 2914, 1641, 1567, 1341, 1254, 1054, 671 cm^{-1}; HRMS (ESI): m/z [M + H]$^+$ calcd. for $C_{19}H_{25}N_2O$: 297.1961; found: 297.1961; $[\alpha]_D^{23}$ = −60.1 (c = 0.20, pyridine), lit[9]. $[\alpha]_D^{25}$= −43.5 (c = 0.5, pyridine).

Supplementary Table 9 ^{1}H NMR data comparison of the synthetic (−)-geissoschizol

**Lit[9]. Synthetic**	**This work**	**Δδ** (Lit – Our synthetic)
8.42 (brs, 1H)	8.32 (s, 1H)	0.1
7.47 (d, J = 7.6 Hz, 1H)	7.48 (d, J = 7.6 Hz, 1H)	−0.01
7.35 (d, J = 7.6 Hz, 1H)	7.34 (d, J = 7.8 Hz, 1H)	0.01
7.19-7.02 (m, 2H)	7.16-7.08 (m, 2H)	-
5.53 (q, J = 6.7 Hz, 1H)	5.54 (q, J = 6.7 Hz, 1H)	−0.01
4.24 (s, 1H)	4.22 (s, 1H)	0.02
3.76-3.51 (m, 3H)	3.66-3.52 (m, 3H)	-
3.32-3.22 (m, 1H)	3.28-3.23 (m, 1H)	-
3.15-2.95 (m, 4H)	3.12-2.96 (m, 4H)	-
2.74-2.63 (m, 1H)	2.69-2.66 (m, 1H)	-
2.44-2.30 (m, 1H)	2.34-2.29 (m, 1H)	-
2.23-2.16 (m, 1H)	2.21-2.16 (m, 1H)	-
1.65 (d, J = 5.5 Hz, 3H)	1.65 (dd, J = 6.8, 1.2 Hz, 3H)	0
1.57-1.46 (m, 2H)	1.54-1.48 (m, 2H)	-

Supplementary Table 10 ^{13}H NMR data comparison of the synthetic (−)-geissoschizol

**Lit[9]. Synthetic**	**This work**	**Δδ** (Lit – Our synthetic)
136.0	137.1	−1.1
135.9	135.9	0

133.5	134.5	−1.0
127.2	127.7	−0.5
121.6	121.4	0.2
121.4	120.2	1.2
119.3	119.4	−0.1
117.9	118.0	−0.1
111.1	110.9	0.2
106.9	107.7	−0.8
61.2	61.9	−0.7
54.1	54.1	0
53.6	53.4	0.2
51.0	51.4	−0.4
35.8	36.1	−0.3
32.5	33.1	−0.6
31.6	31.6	0
18.2	18.3	−0.1
12.9	12.9	0

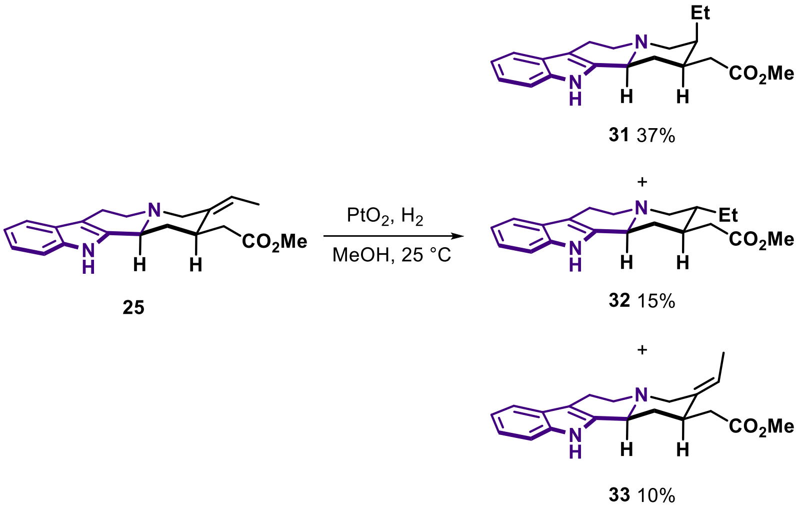

methyl 2-((2*R*, 3*S*, 12b*S*)-3-ethyl-1,2,3,4,6,7,12,12b-octahydroindolo[2,3-*a*]quinolizin-2-yl)acetate (31), **methyl 2-((2*R*, 3*R*, 12b*S*)-3-ethyl-1,2,3,4,6,7,12,12b-octahydroindolo[2,3-*a*]quinolizin-2-yl)acetate (32)** and **methyl 2-((2*R*, 12b*S*, *Z*)-3-ethylidene-1,2,3,4,6,7,12,12b-octahydroindolo[2,3-*a*]quinolizin-2-yl)acetate (33)**

A mixture of **25** (179 mg, 0.55 mmol, 1.0 equiv) and PtO_2 (25 mg, 0.11 mmol, 0.2 equiv) in MeOH (11 mL) was stirred at 25 °C under an atmosphere of H_2 for 1.5 h. The mixture was filtered through a pad of celite and the filter cake was washed with MeOH. The filtrate was concentrated *in vacuo*. The residue was purified by column chromatography on silica gel (petroleum ether: EtOAc, 10:1-4:1) to give **31**, **32**, **33**.

31: Brown oil, isolated yield 37% (66 mg); ^{1}H NMR (400 MHz, $CDCl_3$): δ 7.95 (s, 1H), 7.50 (d, J = 7.4 Hz, 1H), 7.30 (d, J = 7.7 Hz, 1H), 7.18-7.10 (m, 2H), 3.78 (s, 3H), 3.25 (d, J = 9.6 Hz, 1H), 3.05-2.94 (m, 3H), 2.71-2.61 (m, 2H), 2.44-2.30 (m, 4H), 1.94 (d, J = 12.2 Hz, 1H), 1.70-1.50 (m, 3H), 1.34-1.28 (m, 1H), 0.98 (t, J = 6.9 Hz, 3H); ^{13}C NMR (101 MHz, $CDCl_3$): δ 173.6, 135.9, 135.0, 127.3, 121.0, 119.2, 118.0, 110.7, 108.0, 59.9, 57.4, 53.2, 51.6, 39.8, 37.9, 36.6, 31.6, 21.6, 18.0, 12.4; IR (neat): v 3445, 2945, 1754, 1554, 1325, 1009, 679 cm^{-1}; HRMS (ESI): m/z [M + H]$^+$ calcd. for $C_{20}H_{27}N_2O_2$: 327.2068; found: 327.2067; $[\alpha]_D^{23}$ = −12.0 (c = 0.75, DCM).

32: Yellow solid, isolated yield 15% (27 mg); m.p.: 124.1-124.5 °C; ^{1}H NMR (400 MHz, $CDCl_3$): δ 7.83 (s, 1H), 7.48 (d, J = 7.6 Hz, 1H), 7.31 (d, J = 7.8 Hz, 1H), 7.15-7.06 (m, 2H), 3.73 (s, 3H), 3.28 (d, J = 11.6 Hz, 1H), 3.15-3.09 (m, 2H), 3.06-2.98 (m, 1H), 2.76-2.59 (m, 3H), 2.26 (dt, J = 12.4, 3.2 Hz, 1H), 2.17-2.08 (m, 2H), 1.87-1.79 (m, 1H), 1.66-1.59 (m, 1H), 1.55-1.48 (m, 1H), 1.43-1.34 (m, 1H), 1.21-1.14 (m, 1H), 0.94 (t, J = 7.4 Hz, 3H); ^{13}C NMR (101 MHz, $CDCl_3$): δ 173.6, 136.0, 134.4, 127.3, 121.4, 119.4, 118.1, 110.8, 108.1, 60.1, 59.6, 53.1, 51.7, 41.6, 37.9, 37.3, 36.0, 23.5, 21.7, 11.0; IR (neat): v 3441, 2942, 1774, 1654, 1549, 1257, 1109, 671 cm^{-1}; HRMS (ESI): m/z [M + H]$^+$ calcd. for $C_{20}H_{27}N_2O_2$: 327.2068; found: 327.2067; $[\alpha]_D^{23}$ = −8.2 (c = 0.2, DCM).

33: Yellow oil, isolated yield 10% (18 mg); ^{1}H NMR (400 MHz, $CDCl_3$): δ 7.99 (s, 1H), 7.47 (d, J = 7.5 Hz, 1H), 7.29 (d, J = 7.8 Hz, 1H), 7.15 (td, J = 7.1, 1.2 Hz, 1H), 7.10 (td, J = 7.7, 1.2 Hz, 1H), 5.25 (q, J = 6.5 Hz, 1H), 3.90 (d, J = 12.3 Hz, 1H), 3.75 (s, 3H), 3.51 (d, J = 11.2 Hz, 1H), 3.16-3.12 (m, 1H), 3.03-2.96 (m, 1H), 2.80-2.66 (m, 5H), 2.35-2.28 (m, 1H), 2.25-2.21 (m, 1H), 1.72 (d, J = 6.6 Hz, 3H), 1.42 (q, J = 11.9 Hz, 1H); ^{13}C NMR (101 MHz, $CDCl_3$): δ 173.5, 136.0, 136.0, 134.3, 127.2, 121.2, 119.2, 118.1, 116.3, 110.7, 108.1, 59.5, 55.4, 52.5, 51.7, 38.0, 36.7, 36.7, 21.6, 13.1; IR (neat): v 3455, 2952, 1752, 1642, 1453, 1042, 670 cm^{-1}; HRMS (ESI): m/z [M + H]$^+$ calcd. for $C_{20}H_{25}N_2O_2$: 325.1911; found: 325.1910; $[\alpha]_D^{23}$ = −37.3 (c = 0.20, DCM).

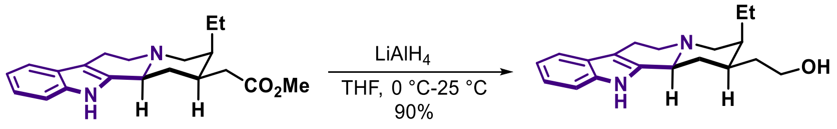

(−)-Corynantheidol (34)

To a solution of **31** (59 mg, 0.18 mmol, 1.0 equiv) in dry THF (3 mL) was added $LiAlH_4$ (0.18 mL, 1.0 M in THF, 0.18 mmol, 1.0 equiv) at 0 °C. After being stirred at 0 °C for 1 h, the reaction mixture was quenched with H_2O. The aqueous layer was extracted with EtOAc. The combined organic layers were washed with brine, dried over Na_2SO_4 and concentrated in *vacuo*. The residue was purified by column chromatography on silica gel (DCM: MeOH, 20:1-10:1) to give (−)-corynantheidol (**34**).

Yellow solid, isolated yield 90% (48 mg); m.p.: 164.2-164.9 °C; ^{1}H NMR (400 MHz, $CDCl_3$): δ 8.11 (s, 1H), 7.46 (d, J = 7.5 Hz, 1H), 7.31 (d, J = 7.8 Hz, 1H), 7.14-7.06 (m, 2H), 3.77-3.72 (m, 2H), 3.17 (d, J = 9.7 Hz, 1H), 3.04-2.91 (m, 3H), 2.69 (d, J = 14.4 Hz, 1H), 2.58-2.53 (m, 1H), 2.35 (d, J = 11.2 Hz, 1H), 1.87 (d, J = 11.5 Hz, 2H), 1.62-1.43 (m, 5H), 1.29-1.24 (m, 1H), 0.93 (t, J = 7.3 Hz, 3H); ^{13}C NMR (101 MHz, $CDCl_3$): δ 135.9, 135.3, 127.4, 121.1, 119.3, 118.0, 110.8, 107.9, 60.8, 60.3, 57.8, 53.4, 39.6, 36.0, 36.3, 31.8, 21.6, 17.7, 12.6; IR (neat): v 3441, 2925, 1741, 1582, 1451, 1321, 1108, 1001, 671cm$^-$

[1]; HRMS (ESI): m/z $[M + H]^+$ calcd. for $C_{19}H_{27}N_2O$: 299.2118; found: 299.2118; $[\alpha]_D^{23} = -97.6$ (*c* = 0.15, pyridine), for (+)-enantiomer: lit[6]. $[\alpha]_D^{25} = +98.7$ (*c* = 0.13, pyridine).

Supplementary Table 11 ^{1}H NMR data comparison of the synthetic (−)-corynantheidol

**Lit[6]. Synthetic**	**This work**	**Δδ** (Lit – Our synthetic)
7.80 (s, 1H)	8.11 (s, 1H)	−0.31
7.46 (d, *J* = 7.2 Hz, 1H)	7.46 (d, *J* = 7.5 Hz, 1H)	0
7.30 (d, *J* = 7.8 Hz, 1H)	7.31 (d, *J* = 7.8 Hz, 1H)	−0.01
7.13-7.05 (m, 2H)	7.14-7.06 (m, 2H)	-
3.76 (t, *J* = 6.0 Hz, 2H)	3.77-3.72 (m, 2H)	-
3.21 (d, *J* = 10.8 Hz, 1H)	3.17 (d, *J* = 9.7 Hz, 1H)	0.04
3.07-2.91 (m, 3H)	3.04-2.91 (m, 3H)	-
2.69 (d, *J* = 13.8 Hz, 1H)	2.69 (d, *J* = 14.4 Hz, 1H)	0
2.61-2.49 (m, 1H)	2.58-2.53 (m, 1H)	-
2.37 (d, *J* = 11.4 Hz, 1H)	2.35 (d, *J* = 11.2 Hz, 1H)	0.02
1.98-1.83 (m, 2H)	1.87 (d, *J* = 11.5 Hz, 2H)	-
1.71-1.45 (m, 8H)	1.62-1.43 (m, 5H)	-
	1.29-1.24 (m, 1H)	
0.93 (t, *J* = 7.2 Hz, 3H)	0.93 (t, *J* = 7.3 Hz, 3H)	0

Supplementary Table 12 ^{13}C NMR data comparison of the synthetic (−)-corynantheidol

**Lit[6]. Synthetic**	**This work**	**Δδ** (Lit – Our synthetic)
135.9	135.9	0
135.4	135.3	0.1
127.5	127.4	0.1
121.2	121.1	0.1
119.3	119.3	0
118.1	118.0	0.1
110.7	110.8	−0.1
108.2	107.9	0.3
60.9	60.8	0.1
60.4	60.3	0.1
58.0	57.8	0.2
53.5	53.4	0.1
39.6	39.6	0
36.4	36.0	0.4
36.1	36.3	−0.2
32.1	31.8	0.3
21.8	21.6	0.2
17.7	17.7	0
12.6	12.6	0

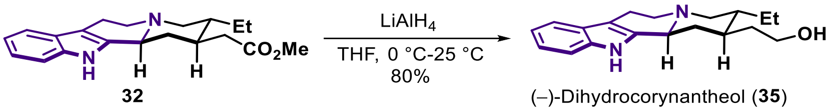

(−)-Dihydrocorynantheol (35)

To a solution of **32** (27 mg, 0.08 mmol, 1.0 equiv) in THF (1.6 mL) was added $LiAlH_4$ (0.08 mL, 1.0 M in THF, 0.08 mmol, 1.0 equiv) at 0 °C. After being stirred at 0 °C for 1 h, the reaction mixture was quenched with H_2O. The aqueous layer was extracted with EtOAc. The combined organic layers were washed with brine, dried over Na_2SO_4 and concentrated in *vacuo*. The residue was purified by column chromatography on silica gel (DCM: MeOH, 20:1-10:1) to give (−)-dihydrocorynantheol (**35**).

White solid, isolated yield 80% (19 mg); m.p.: 177.3-117.8 °C; ^{1}H NMR (400 MHz, $CDCl_3$): δ 8.54 (s, 1H), 7.45 (d, J = 7.6 Hz, 1H), 7.31 (d, J = 7.8 Hz, 1H), 7.13-7.05 (m, 2H), 3.72 (t, J = 6.2 Hz, 2H), 3.09-2.96 (m, 4H), 2.73 (dd, J = 13.3, 2.4 Hz, 1H), 2.56-2.51 (m, 1H), 2.25 (d, J = 11.4 Hz, 1H), 2.01-1.87 (m, 2H), 1.64-1.55 (m, 1H), 1.43-1.24 (m, 4H), 1.11-1.04 (m, 1H), 0.89 (t, J = 7.4 Hz, 3H); ^{13}C NMR (101 MHz, $CDCl_3$): δ 136.1, 134.5, 127.2, 121.3, 119.3, 118.0, 111.0, 107.5, 60.2, 60.1, 59.8, 52.9, 41.4, 37.0, 35.2, 35.0, 23.3, 21.4, 10.9; IR (neat): ν 3445, 2969, 1712, 1645, 1536, 1445, 1105, 1011, 679 cm^{-1}; HRMS (ESI): m/z $[M + H]^+$ calcd. for $C_{19}H_{27}N_2O$: 299.2118; found: 299.2118; $[\alpha]_D^{23}$ = −10.5 (c = 0.20, $CHCl_3$), lit[10]. $[\alpha]_D^{22.3}$= −10.7 (c = 0.5, $CHCl_3$).

Supplementary Table 13 ^{1}H NMR data comparison of the synthetic (−)-dihydrocorynantheol

**Lit[10]. Synthetic**	**This work**	**Δδ** (Lit – Our synthetic)
8.21 (br, 1H)	8.54 (s, 1H)	−0.33
7.48 (d, J = 7.8 Hz, 1H)	7.45 (d, J = 7.6 Hz, 1H)	0.03
7.33 (d, J = 7.8 Hz, 1H)	7.31 (d, J = 7.8 Hz, 1H)	0.02
7.16-7.08 (m, 2H)	7.13-7.05 (m, 2H)	-
3.79-3.72 (m, 2H)	3.72 (t, J = 6.2 Hz, 2H)	-
3.14-3.06 (m, 3H)	3.09-2.96 (m, 4H)	-
3.05-3.01 (m,1H)		
2.59-2.53 (m, 1H)	2.56-2.51 (m, 1H)	-
2.34 (br, 1H)	-	-
2.25 (d, J = 12.6 Hz, 1H)	2.25 (d, J = 11.4 Hz, 1H)	0
2.07-1.95 (m, 2H)	2.01-1.87 (m, 2H)	-
1.71-1.62 (m, 1H)	1.64-1.55 (m, 1H)	-
1.51-1.41 (m, 2H)	1.43-1.24 (m, 4H)	-
1.37-1.22 (m, 2H)		
1.16-1.11 (m, 1H)	1.11-1.04 (m, 1H)	-
0.92 (t, J = 7.4 Hz, 3H)	0.89 (t, J = 7.4 Hz, 3H)	0.03

Supplementary Table 14 ^{13}C NMR data comparison of the synthetic (−)-dihydrocorynantheol

**Lit[10]. Synthetic**	**This work**	**Δδ** (Lit – Our synthetic)

136.1	136.1	0
134.9	134.5	0.4
127.3	127.2	0.1
121.2	121.3	−0.1
119.2	119.3	−0.1
118.1	118.0	0.1
110.9	111.0	−0.1
107.7	107.5	0.2
60.3	60.2	0.1
60.1	60.1	0
59.8	59.8	0
53.1	52.9	0.2
41.6	41.4	0.2
37.2	37.0	0.2
35.4	35.2	0.2
35.2	35.0	0.2
23.4	23.3	0.1
21.6	21.4	0.2
11.1	10.9	0.2

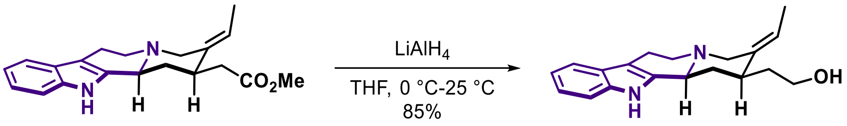

33

(–)-Isogeissoschizol (**36**)

(−)-Isogeissoschizol (36)

To a solution of **33** (10 mg, 0.03 mmol, 1.0 equiv) in THF (0.6 mL) was added $LiAlH_4$ (0.03 mL, 1.0 M in THF, 0.13 mmol, 1.0 equiv) at 0 °C. After being stirred at 0 °C for 1 h, the reaction mixture was quenched with H_2O. The aqueous layer was extracted with EtOAc. The combined organic layers were washed with brine, dried over Na_2SO_4 and concentrated in *vacuo*. The residue was purified by column chromatography on silica gel (petroleum ether: EtOAc, 1:2) to give (−)-isogeissoschizol (**36**).

Yellow solid, isolated yield 85% (7.6 mg); m.p.: 91.7-92.2 °C; ^{1}H NMR (400 MHz, $CDCl_3$): δ 8.23 (s, 1H), 7.43 (d, J = 6.3 Hz, 1H), 7.32 (d, J = 8.0 Hz, 1H), 7.13-7.04 (m, 2H), 5.31 (d, J = 6.6 Hz, 1H), 3.86 (d, J = 12.1 Hz, 1H), 3.79-3.74 (m, 2H), 3.43-3.33 (m, 1H), 3.14-3.10 (m, 1H), 3.02-2.95 (m, 1H), 2.75-2.62 (m, 3H), 2.33-2.18 (m, 2H), 2.05-1.97 (m, 1H), 1.70 (d, J = 6.6 Hz, 3H), 1.53-1.47 (m, 1H), 1.32-1.28 (m, 1H); ^{13}C NMR (101 MHz, $CDCl_3$): δ 136.7, 136.1, 134.6, 127.3, 121.2, 119.2, 118.1, 116.5, 110.9, 107.8, 60.4, 59.7, 55.6, 52.4, 37.7, 36.4, 34.2, 21.5, 13.1; IR (neat): ν 3445, 2914, 1745, 1556, 1428, 1341, 1163, 670 cm^{-1}; HRMS (ESI): m/z $[M + H]^+$ calcd. for $C_{19}H_{25}N_2O$: 297.1961; found: 297.1961; $[\alpha]_D^{23}$ = −34.2 (c = 0.08, MeOH), lit[10]. $[\alpha]_D^{16.5}$= −27.9 (c = 0.33, MeOH).

Supplementary Figure 4: ^{1}H NMR data comparison of the synthetic (−)-isogeissoschizol

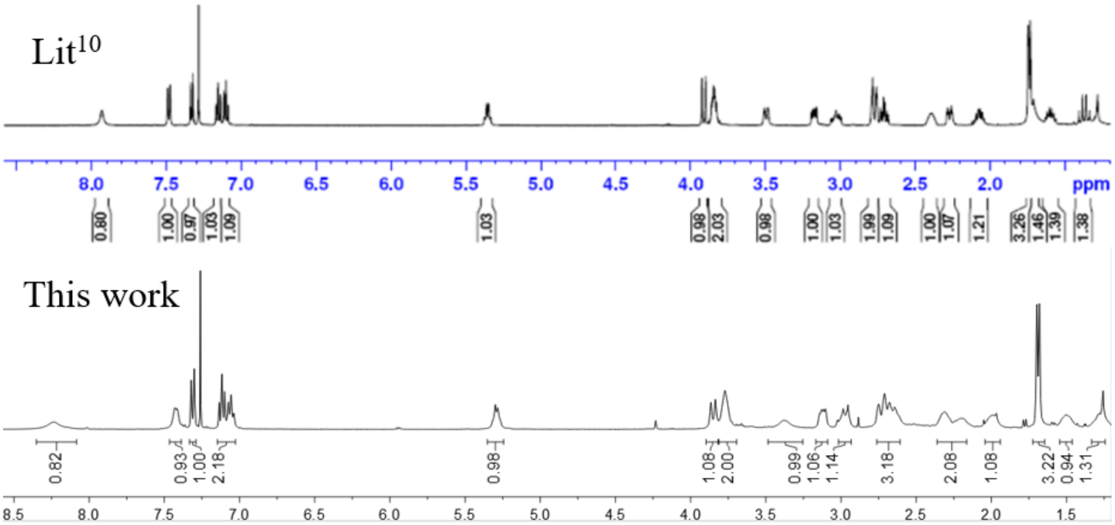

Supplementary Figure 5: ^{13}C NMR data comparison of the synthetic (−)-isogeissoschizol

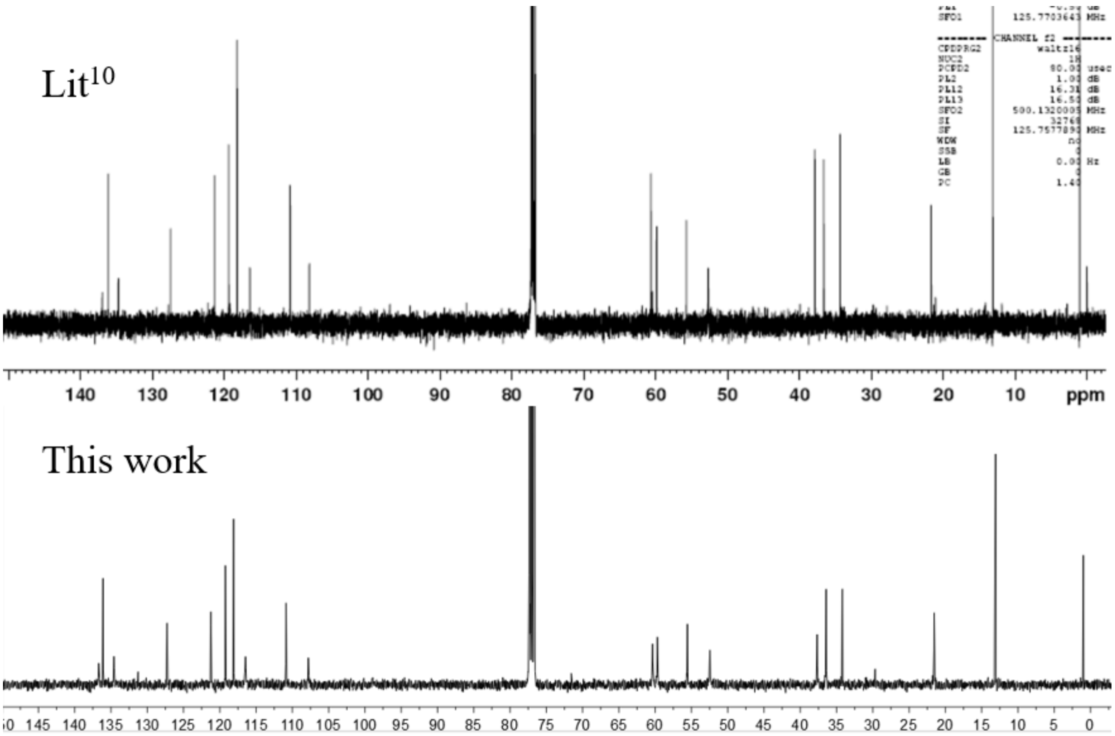

2.9 Synthesis of yohimbine family alkaloids

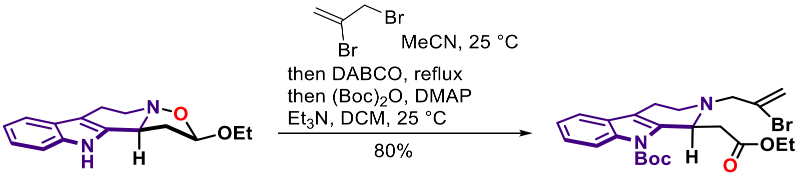

tert-butyl (*S*)-2-(2-bromoallyl)-1-(2-ethoxy-2-oxoethyl)-1,2,3,4-tetrahydro-9*H*-pyrido[3,4-*b*]indole-9-carboxylate (37)

To a solution of **3a** (1.23 g, 4.75 mmol, 1.0 equiv) in MeCN (10 mL) was added 2,3-dibromopropene (0.7 mL, 7.0 mmol, 1.5 equiv). The reaction mixture was stirred at 25 °C for 20 h. Then DABCO (693 mg, 6.2 mmol, 1.3 equiv) was added and the reaction was refluxed for 1 h. The reaction solution was concentrated *in vacuo*. The above residue was dissolved in DCM (67 mL), and Et_3N (2.0 mL, 14.3 mmol, 3.0 equiv), DMAP (59 mg, 0.48 mmol, 0.1 equiv) and $(Boc)_2O$ (2.0 g, 9.5 mmol, 2.0 equiv) were added. The reaction was stirred at 25 °C overnight then was concentrated *in vacuo*. The residue was purified by column chromatography on silica gel (petroleum ether: EtOAc, 20:1) to give **37**.

Pale yellow foam, isolated yield 80% (1.8 g); ^1H NMR (400 MHz, $CDCl_3$): δ 8.20 (d, J = 8.2 Hz, 1H), 7.45 (d, J = 7.2 Hz, 1H), 7.33 (t, J = 7.4 Hz, 1H), 7.27 (t, J = 7.6 Hz, 1H), 5.88 (s, 1H), 5.60 (s, 1H), 4.80 (dd, J = 11.0, 3.6 Hz, 1H), 4.29-4.14 (m, 2H), 3.52-3.39 (m, 2H), 3.37-3.29 (m, 1H), 3.09 (dd, J = 14.5, 6.2 Hz, 1H), 2.98 (dd, J = 14.4, 3.9 Hz, 1H), 2.89-2.81 (m, 1H), 2.70 (dd, J = 14.3, 11.1 Hz, 1H), 2.56 (dd, J = 16.7, 5.0 Hz, 1H), 1.71 (m, 9H), 1.33 (t, J = 7.2 Hz, 3H); ^{13}C NMR (101 MHz, $CDCl_3$) δ 170.9, 150.0, 136.0, 134.7, 131.7, 129.1, 124.4, 122.7, 118.2, 117.9, 115.7, 114.8, 84.3, 61.7, 60.5, 56.0, 40.7, 40.3, 28.2, 16.6, 14.2; IR (neat): ν 3863, 3759, 2372, 2348, 1739, 1461, 1142, 1115, 801, 671 cm^{-1}; HRMS (ESI): m/z $[M + H]^+$ calcd. For $C_{23}H_{30}BrN_2O_4$: 477.1383; found: 477.1379; $[\alpha]_D^{23}$ = −20.4 (c = 0.20, $CHCl_3$).

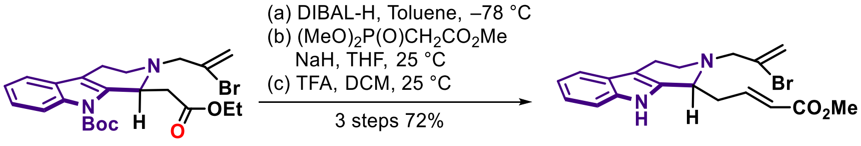

methyl (*S*, *E*)-4-(2-(2-bromoallyl)-2,3,4,9-tetrahydro-1*H*-pyrido[3,4-*b*]indol-1-yl)but-2-enoate (38)

Under N_2, to a solution of **37** (1.8 g, 3.8 mmol, 1.0 equiv) in dry toluene (19 mL) was added DIBAL-H (4.6 mL, 1.0 M in Hexane, 4.6 mmol, 1.2 equiv) at −78 °C. After being stirred for 30 min, the reaction was quenched with addition of aqueous saturated Rochelle salt solution and extracted with EtOAc. The combined organic layers were washed with brine, dried over Na_2SO_4, filtered and concentrated *in vacuo*. The residue was purified by column chromatography on silica gel (petroleum ether: EtOAc, 20:1). To a solution of NaH (364 mg, 60% in mineral oil, 9.0 mmol, 2.4 equiv) in THF (32 mL) was added $(MeO)_2P(O)CH_2CO_2Me$ (1.72 g, 9.5 mmol, 2.5 equiv). The reaction mixture was stirred at 25 °C for 30 min. Then above crude product in THF (13 mL) was added and the reaction was stirred at 25 °C for 1 h before being quenched with saturated aqueous $NaHCO_3$ solution. The separated aqueous phase was

extracted with EtOAc. The combined organic layers were washed with brine, dried over Na_2SO_4, filtered and concentrated *in vacuo*. The residue was purified by column chromatography on silica gel (petroleum ether: EtOAc, 20:1). The above crude product was dissolved in DCM (19 mL) followed by adding TFA (19 mL), The reaction mixture was stirred at 25 °C for 2 h. the reaction was quenched with saturated aqueous $NaHCO_3$ solution and extracted with DCM. The combined organic layers were washed with brine, dried over Na_2SO_4, filtered and concentrated *in vacuo*. The residue was purified by column chromatography on silica gel (petroleum ether: EtOAc, 10:1) to give **38**.

Yellow oil, isolated yield 72% for 3 steps (1.1 g); ^1H NMR (400 MHz, $CDCl_3$) δ 7.87 (s, 1H), 7.52 (d, J = 7.7 Hz, 1H), 7.33 (d, J = 8.0 Hz, 1H), 7.21-7.10 (m, 3H), 5.97 (d, J = 15.7 Hz, 1H), 5.88 (s, 1H), 5.61 (s, 1H), 3.87 (t, J = 6.7 Hz, 1H), 3.76 (s, 3H), 3.45 (s, 2H), 3.29-3.22 (m, 1H), 3.06 (ddd, J = 13.6, 5.2, 2.6 Hz, 1H), 2.90-2.82 (m, 1H), 2.80-2.60 (m, 3H); ^{13}C NMR (101 MHz, $CDCl_3$) δ 166.9, 146.4, 135.8, 133.5, 132.0, 127.0, 122.8, 121.8, 119.4, 118.5, 118.2, 110.9, 108.3, 61.6, 56.1, 51.5, 44.4, 37.6, 17.8; IR (neat): v 2926, 2351, 1756, 1659, 1264, 1020, 801, 671 cm^{-1}; HRMS (ESI): m/z $[M + H]^+$ calcd. For $C_{19}H_{22}BrN_2O_2$: 389.0859; found: 389.0854; $[\alpha]_D^{23}$ = +13.6 (c = 0.20, $CHCl_3$).

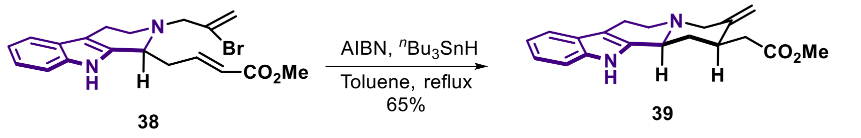

methyl 2-((2*R*, 12b*S*)-3-methylene-1,2,3,4,6,7,12,12b-octahydroindolo[2,3-*a*]quinolizin-2-yl)acetate (39)

A solution of **38** (1.08 g, 2.77 mmol) in THF (35 mL) was stirred at reflux while AIBN (22 mg, 0.14 mmol) was added, followed by the slow addition of a solution of $^nBu_3SnH$ (1.1 mL, 3.92 mmol) and AIBN (210 mg, 1.26 mmol) in THF (20 mL) by a syringe pump over a period of 4 h. Following the complete addition of the $^nBu_3SnH$ solution, the reaction mixture was maintained at reflux for a further 2 h, after the solution was cooled to 25 °C. The reaction mixture was concentrated in *vacuo*. The residue was purified by column chromatography on silica gel (petroleum ether: EtOAc, 2:1) to give **39**.

Yellow form, isolated yield 65% (405 mg); ^1H NMR (400 MHz, $CDCl_3$) δ 7.86 (s, 1H), 7.47 (d, J = 7.6 Hz, 1H), 7.31 (d, J = 7.8 Hz, 1H), 7.15 (t, J = 6.9 Hz, 1H), 7.10 (t, J = 6.9 Hz, 1H), 4.99 (s, 1H), 4.75 (s, 1H), 3.75 (s, 3H), 3.55 (d, J = 10.6 Hz, 1H), 3.50 (d, J = 11.9 Hz, 1H), 3.14-3.11 (m, 2H), 3.04-2.96 (m, 1H), 2.82-2.73 (m, 3H), 2.70 (td, J = 10.9, 4.4 Hz, 1H), 2.40-2.28 (m, 2H), 1.44 (q, J = 12.0 Hz, 1H); ^{13}C NMR (101 MHz, $CDCl_3$): δ 173.1, 145.2, 136.0, 133.9, 127.2, 121.4, 119.4, 118.2, 110.8, 108.5, 108.3, 62.8, 59.4, 52.3, 51.8, 37.4, 36.7, 36.6, 21.6; IR (neat): v 3478, 2934, 1731, 1652, 1460, 1151, 802 cm^{-1}; HRMS (ESI): m/z $[M + H]^+$ calcd. For $C_{19}H_{23}N_2O_2$: 311.1754; found: 311.1752; $[\alpha]_D^{23}$ = +12.2 (c = 0.2, $CHCl_3$).

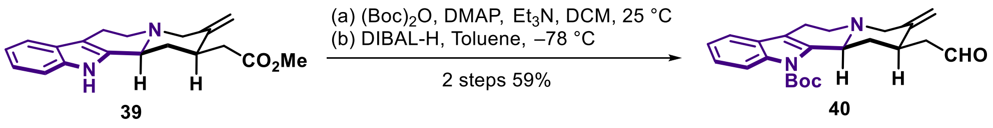

***tert*-butyl (2*R*, 12b*S*)-3-methylene-2-(2-oxoethyl)-1,3,4,6,7,12b-hexahydroindolo[2,3-*a*]quinolizine-12(2H)-carboxylate (40)**

To a solution of **39** (558 mg, 1.8 mmol, 1.0 equiv) in DCM (25 mL) was added Et_3N (0.75 mL, 5.4 mmol, 3.0 equiv), DMAP (22 mg, 0.18 mmol, 0.1 equiv) and $(Boc)_2O$ (0.78 g, 3.6 mmol, 2.0 equiv), The reaction was stirred at 25 °C overnight then was concentrated *in vacuo*. The residue was purified by column chromatography on silica gel (petroleum ether: EtOAc, 6:1). Under N_2, to a solution of the above ester (1.8 mmol, 1.0 equiv) in dry toluene (11 mL) was added DIBAL-H (2.0 mL, 1.0 M in Hexane, 1.98 mmol, 1.1 equiv) at −78 °C. After being stirred for 30 min, the reaction was quenched with addition of aqueous saturated Rochelle salt solution and extracted with EtOAc. The combined organic layers were washed with brine, dried over Na_2SO_4, filtered and concentrated *in vacuo*. The residue was purified by column chromatography on silica gel (petroleum ether: EtOAc, 5:1) to give **40**.

Yellow oil, isolated yield 59% for 2 steps (411 mg); ^{1}H NMR (400 MHz, $CDCl_3$): δ 9.83 (s, 1H), 8.10 (d, J = 8.0 Hz, 1H), 7.40 (d, J = 8.4 Hz, 1H), 7.28-7.19 (m, 2H), 4.92 (s, 1H), 4.72 (s, 1H), 4.66 (d, J = 10.4 Hz, 1H), 3.76 (d, J = 13.6 Hz, 1H), 3.48 (d, J = 13.6 Hz, 1H), 3.07-2.94 (m, 2H), 2.86-2.73 (m, 4H), 2.44 (ddd, J = 17.1, 6.9, 1.8 Hz, 1H), 2.30-2.25 (m, 1H), 1.70 (s, 9H), 1.54-1.45 (m, 1H); ^{13}C NMR (101 MHz, $CDCl_3$): δ 201.5, 150.1, 144.5, 136.3, 129.2, 123.9, 122.6, 117.9, 115.7, 115.0, 108.5, 83.9, 62.6, 57.2, 46.3, 44.6, 35.7, 35.0, 28.2, 21.9; IR (neat): ν 2933, 2857, 1379, 1151, 802, 751 cm^{-1}; HRMS (ESI): m/z $[M + H]^+$ calcd. For $C_{23}H_{29}N_2O_3$: 381.2173; found: 381.2168; $[\alpha]_D^{23}$ =−13.2 (c = 0.20, $CHCl_3$).

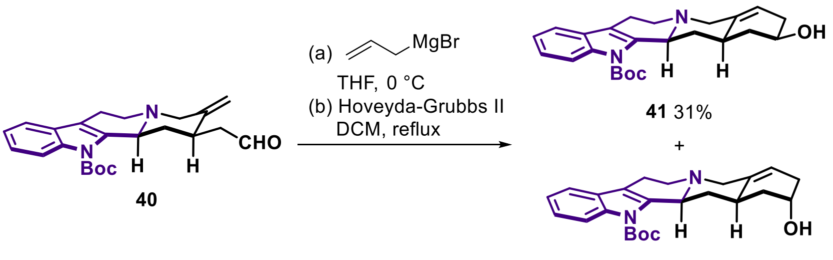

40 → **41** 31% + **42** 15%

***tert*-butyl (2*R*, 13b*S*, 14a*R*)-2-hydroxy-1,3,5,7,8,13b,14,14a-octahydroindolo[2',3':3,4]pyrido[1,2-*b*]isoquinoline-13(2*H*)-carboxylate (41) and *tert*-butyl (2*S*, 13b*S*, 14a*R*)-2-hydroxy-1,3,5,7,8,13b,14,14a-octahydroindolo[2',3':3,4]pyrido[1,2-*b*]isoquinoline-13(2*H*)-carboxylate (42)**

Under N_2, to a solution of **40** (400 mg, 1.05 mmol, 1.0 equiv) in dry THF (8.0 mL) was added allylmagnesium bromide (1.05 mL, 1.0 M in diethyl ether, 1.05 mmol, 1.0 equiv). The reaction mixture was stirred at 0 °C for 1 h before being quenched with saturated aqueous NH_4Cl solution and extracted with EtOAc. The combined organic layers were washed with brine, dried over Na_2SO_4, filtered and concentrated *in vacuo*. The residue was purified by column chromatography on silica gel (petroleum ether: EtOAc, 2:1) to give the desired product as inseparable mixture. To a solution of the above product in dry DCM (400 mL) was added Hoveyda-Grubbs II (94 mg, 0.15 mmol, 0.15 equiv). The reaction mixture was stirred at reflux for 48 h under N_2 atmosphere. The reaction solution was concentrated in *vacuo*. The residue was purified by column chromatography on silica gel (petroleum ether: EtOAc, 5:1-1:5) to give the **41** and **42**.

41: Brown form, isolated yield 31% (128 mg); ^{1}H NMR (400 MHz, $CDCl_3$) δ 8.08 (d, J = 7.7 Hz, 1H), 7.40 (d, J = 8.3 Hz, 1H), 7.26-7.19 (m, 2H), 5.44 (s, 1H), 4.30 (d, J = 10.6 Hz, 1H), 3.92-3.85 (m, 1H), 3.51 (d, J = 13.5 Hz, 1H), 3.39 (d, J = 13.7 Hz, 1H), 3.08-3.03 (m, 1H), 2.81-2.69 (m, 3H), 2.51 (s, 1H), 2.39 (d, J = 16.6 Hz, 1H), 2.29 (d, J = 18.0 Hz, 1H), 2.06-1.99 (m, 2H), 1.67 (s, 9H), 1.42-1.28 (m, 2H); ^{13}C NMR (101 MHz, $CDCl_3$): δ 150.2, 136.6, 136.4, 135.0, 129.2, 123.9, 122.6, 119.2, 117.9, 115.5, 115.4, 83.6, 67.3, 60.8, 57.8, 46.2, 39.6, 36.3, 36.1, 34.9, 28.2, 22.0; IR (neat): ν 2930, 2368, 1731, 1460,

1374, 1262, 1151, 802 cm^{-1}; HRMS (ESI): m/z [M + H]$^+$ calcd. For $C_{24}H_{31}N_2O_3$: 395.2329; found: 395.2325; $[\alpha]_D^{23}$ = −34.4 (*c* = 0.28, $CHCl_3$).

42: Brown form, isolated yield 15% (62 mg); ^{1}H NMR (400 MHz, $CDCl_3$) δ 8.09 (d, *J* = 8.0 Hz, 1H), 7.41 (d, *J* = 8.2 Hz, 1H), 7.28-7.19 (m, 2H), 5.47 (s, 1H), 4.40 (d, *J* = 10.7 Hz, 1H), 4.15-4.08 (m, 1H), 3.61 (d, *J* = 13.2 Hz, 1H), 3.38 (d, *J* = 13.5 Hz, 1H), 3.10-3.04 (m, 1H), 2.81-2.61 (m, 3H), 2.61 (s, 1H), 2.41 (d, *J* = 17.7 Hz, 1H), 2.26-2.22 (m, 1H), 2.11 (d, *J* = 17.9 Hz, 1H), 1.98-1.92 (m, 1H), 1.68 (s, 9H), 1.48-1.36 (m, 2H); ^{13}C NMR (101 MHz, $CDCl_3$): δ 150.2, 136.6, 136.5, 135.0, 129.2, 123.9, 122.6, 118.2, 117.9, 115.5, 115.3, 83.7, 64.7, 61.4, 58.2, 46.1, 36.6, 36.2, 33.7, 31.9, 28.3, 22.0; IR (neat): *v* 2368, 1735, 1450, 1374, 1262, 1152 cm^{-1}; HRMS (ESI): m/z [M + H]$^+$ calcd. For $C_{24}H_{31}N_2O_3$: 395.2329; found: 395.2325; $[\alpha]_D^{23}$ = −22.1 (*c* = 0.30, $CHCl_3$).

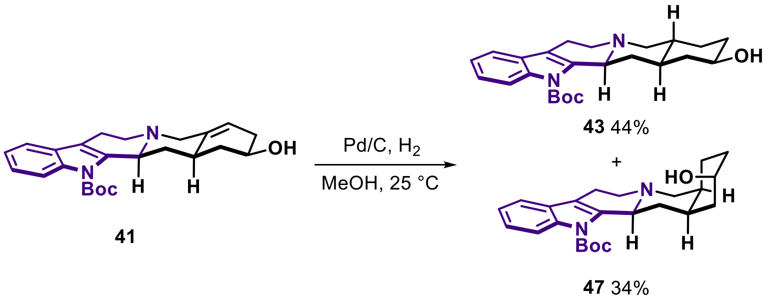

tert-butyl (2*R*, 4a*R*, 13b*S*, 14a*R*)-2-hydroxy-1,3,4,4a,5,7,8,13b,14,14a-decahydroindolo[2',3':3,4]pyrido[1,2-*b*]isoquinoline-13(2*H*)-carboxylate (43) and ***tert*-butyl (2*R*, 4a*S*, 13b*S*, 14a*R*)-2-hydroxy-1,3,4,4a,5,7,8,13b,14,14a-decahydroindolo[2',3':3,4]pyrido[1,2-*b*]isoquinoline-13(2*H*)-carboxylate (47)**

To a solution of **41** (110 mg, 0.28 mmol, 1.0 equiv) in MeOH (5.6 mL) was adde Pd/C (100 mg). The reaction mixture was stirred at 25 °C under an atmosphere H_2 for 5 h. The reaction solution was filtered through a pad of celite and concentrated in *vacuo*. The residue was purified by column chromatography on silica gel (petroleum ether: EtOAc, 4:1-1:1) to give **43** and **47**.

43: White form, isolated yield 44% (49 mg); ^{1}H NMR (400 MHz, $CDCl_3$): δ 8.06 (d, *J* = 7.4 Hz, 1H), 7.40 (d, *J* = 8.4 Hz, 1H), 7.26 (t, *J* = 7.2 Hz, 1H), 7.21 (t, *J* = 7.2 Hz, 1H), 4.08 (d, *J* = 10.2 Hz, 1H), 3.69-3.62 (m, 1H), 3.17-3.10 (m, 1H), 3.02 (dd, *J* = 12.4 Hz, *J* = 3.2 Hz, 1H), 2.83-2.69 (m, 3H), 2.56-2.50 (m, 1H), 2.05-2.02 (m, 2H), 1.92-1.89 (m, 1H), 1.68-1.58 (m, 12H), 1.35-1.27 (m, 2H), 1.10-0.96 (m, 2H); ^{13}C NMR (101 MHz, $CDCl_3$): δ 150.3, 136.9, 136.6, 129.2, 123.8, 122.6, 117.9, 115.7, 115.4, 83.6, 70.4, 61.1, 58.9, 47.3, 42.0, 40.4, 37.9, 35.4, 35.0, 28.2, 22.3 (2C); IR (neat): *v* 2930, 2860, 1737, 1380, 1263, 801 cm^{-1}; HRMS (ESI): m/z [M + H]$^+$ calcd. For $C_{24}H_{33}N_2O_3$: 397.2486; found: 397.2483; $[\alpha]_D^{23}$ = −9.6 (*c* = 0.27, $CHCl_3$).

47: White solid, isolated yield 34% (38 mg); m.p.: 164.7-165.2 °C; ^{1}H NMR (400 MHz, $CDCl_3$): δ 8.05 (d, *J* = 8.0 Hz, 1H), 7.39 (d, *J* = 7.0 Hz, 1H), 7.26-7.18 (m, 2H), 4.02-4.01 (m, 1H), 3.60 (s, 1H), 3.00 (s, 1H), 2.89-2.73 (m, 3H), 2.73-2.60 (m, 2H), 2.29 (qd, *J* = 13.5 Hz, *J* = 2.8 Hz, 1H), 2.18-2.09 (m, 1H), 2.09-1.99 (m, 2H), 1.74-1.68 (m, 4H), 1.65 (s, 9H), 1.52-1.46 (m, 1H), 1.30-1.28 (m, 1H).; ^{13}C NMR (101 MHz, $CDCl_3$): δ 150.4, 137.1, 129.2 (2C), 123.8, 122.5, 117.8, 116.6, 115.2, 83.4, 66.3, 61.9, 61.3, 50.8, 37.4, 36.5, 34.4, 34.1, 33.0, 28.2, 22.4, 21.3; IR (neat): *v* 2931, 1737, 1462, 1263, 1098, 802 cm^{-1};

HRMS (ESI): m/z $[M + H]^+$ calcd. For $C_{24}H_{33}N_2O_3$: 395.2329; found: 395.2325; $[\alpha]_D^{23}$ = −24.7 (*c* = 0.2, $CHCl_3$).

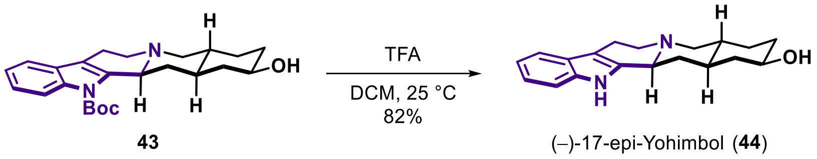

(−)-17-epi-Yohimbol (44)

To a solution of **43** (24 mg, 0.06 mmol, 1.0 equiv) in DCM (0.12 mL) was added TFA (0.06 mL), The reaction mixture was stirred at 25 °C for 1.5 h. the reaction was quenched with saturated aqueous $NaHCO_3$ solution and extracted with DCM. The combined organic layers were washed with brine, dried over Na_2SO_4, filtered and concentrated *in vacuo*. The residue was purified by column chromatography on silica gel (DCM: MeOH, 15:1) to give (−)-17-epi-yohimbol (**44**).

White form, isolated yield 82% (14 mg); ^{1}H NMR (400 MHz, $CDCl_3$:CD_3OD, 10:1 v/v): δ 7.42 (d, J = 7.4 Hz, 1H), 7.26 (d, J = 7.4 Hz, 1H), 7.10 (t, J = 7.1 Hz, 1H), 7.04 (t, J = 7.0 Hz, 1H), 3.60-3.52 (m, 1H), 3.28 (d, J = 11.5 Hz, 1H), 3.04-2.94 (m, 2H), 2.92 (dd, J = 11.4, 3.7 Hz, 1H), 2.72 (dd, J = 15.2 Hz, 4.4 Hz, 1H), 2.63 (td, J = 11.2, 4.5 Hz, 1H), 2.09-2.03 (m, 2H), 1.98-1.91 (m, 2H), 1.65-1.60 (m, 1H), 1.38-1.26 (m, 2H), 1.21-1.13 (m, 2H), 1.09-0.99 (m, 2H); ^{13}C NMR (151 MHz, $CDCl_3$:CD_3OD, 10:1 v/v): δ 136.0, 134.3, 127.0, 121.1, 119.0, 117.9, 110.8, 107.2, 70.0, 61.0, 60.0, 53.1, 41.2, 40.3, 39.5, 35.8, 34.7, 28.2, 21.4; IR (neat): ν 2925, 2832, 1648, 1263, 1096, 1024, 801 cm^{-1}; HRMS (ESI): m/z $[M + H]^+$ calcd. For $C_{19}H_{25}N_2O$: 297.1961; found: 297.1960; $[\alpha]_D^{23}$ = −78.1 (*c* = 0.10, MeOH), for (+)-enantiomer: lit[6]. $[\alpha]_D^{20}$ = +71.6 (*c* = 0.10, MeOH).

Supplementary Table 15 ^{1}H NMR data comparison of the synthetic (−)-17-epi-yohimbol

Lit[6]. Synthetic	This work	Δδ (Lit – Our synthetic)
7.77 (s, 1H)	-	-
7.47 (d, J = 7.8 Hz, 1H)	7.42 (d, J = 7.4 Hz, 1H)	0.05
7.31 (d, J = 8.4 Hz, 1H)	7.26 (d, J = 7.4 Hz, 1H)	0.05
7.14 (t, J = 7.8 Hz, 1H)	7.10 (t, J = 7.1 Hz, 1H)	0.04
7.09 (t, J = 7.8 Hz, 1H)	7.04 (t, J = 7.0 Hz, 1H)	0.05
3.70-3.66 (m, 1H)	3.60-3.52 (m, 1H)	-
3.48 (s, 1H)	-	-
3.27 (d, J = 10.2 Hz, 1H)	3.28 (d, J = 11.5 Hz, 1H)	−0.01
3.06 (q, J = 6.0 Hz, 1H)	3.04-2.94 (m, 2H)	-
3.00-2.94 (m, 1H)		
2.90 (dd, J = 11.4, 3.6 Hz, 1H)	2.92 (dd, J = 11.4, 3.7 Hz, 1H)	−0.02
2.70 (dd, J = 5.6, 4.8 Hz, 1H)	2.72 (dd, J = 15.2 Hz, 4.4 Hz, 1H)	−0.02
2.60 (td, J = 11.4, 4.2 Hz, 1H)	2.63 (td, J = 11.2, 4.5 Hz, 1H)	−0.03
2.08-2.03 (m, 2H)	2.09-2.03 (m, 2H)	-
2.00-1.93 (m, 2H)	1.98-1.91 (m, 2H)	-

1.65-1.63 (m, 1H)	1.65-1.60 (m, 1H)	-
1.39-1.33 (m, 2H)	1.38-1.26 (m, 2H)	-
1.27 (qd, *J* = 16.2, 3.6 Hz, 1H)	1.21-1.13 (m, 2H)	-
1.22-1.18 (m, 1H)		
1.08 (q, *J* = 12.0 Hz, 1H)	1.09-0.99 (m, 2H)	-
1.03 (qd, *J* = 13.2, 3.6 Hz, 1H)		

Supplementary Table 16 ^{13}C NMR data comparison of the synthetic (−)-17-epi-yohimbol

**Lit[6]. Synthetic**	**This work**	**Δδ** (Lit – Our synthetic)
136.0	136.0	0
134.3	134.3	0
127.1	127.0	0.1
121.2	121.1	0.1
119.2	119.0	0.2
118.0	117.9	0.1
110.8	110.8	0
107.6	107.2	0.4
70.2	70.0	0.2
61.1	61.0	0.1
60.0	60.0	0
53.1	53.1	0
41.1	41.2	−0.1
40.4	40.3	0.1
39.6	39.5	0.1
36.0	35.8	0.2
34.9	34.7	0.2
28.3	28.2	0.1
21.5	21.4	0.1

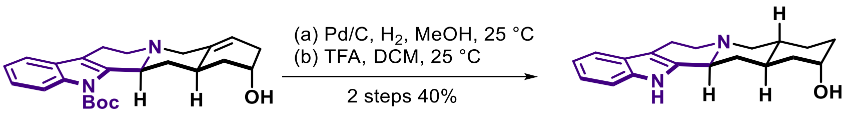

42 (−)-Yohimbol (**45**)

(−)-Yohimbol (45)

To a solution of **42** (26 mg, 0.07 mmol, 1.0 equiv) in MeOH (1.4 mL) was added Pd/C (26 mg), The reaction mixture was stirred at 35 °C under an atmosphere H_2 for 3 h. The reaction solution was filtered through a pad of celite and concentrated in *vacuo*. To a solution of the residue in DCM (13 mL) was added TFA (0.035 mL), The reaction mixture was stirred at 25 °C for 1.5 h. the reaction was quenched with saturated aqueous $NaHCO_3$ solution and extracted with DCM. The combined organic layers were

washed with brine, dried over Na_2SO_4, filtered and concentrated *in vacuo*. The residue was purified by column chromatography on silica gel (DCM: MeOH, 15:1-5:1) to give (−)-yohimbol (**45**).

White solid, isolated yield 40% for 2 steps (8 mg); m.p.: 256.2-256.8 ℃; ^{1}H NMR (600 MHz, $CDCl_3$:CD_3OD, 10:1 v/v) δ 7.38 (d, J = 7.8 Hz, 1H), 7.26 (d, J = 8.2 Hz, 1H), 7.05 (t, J = 7.1 Hz, 1H), 6.99 (t, J = 7.3 Hz, 1H), 4.07 (s, 1H), 3.40 (d, J = 10.6 Hz, 1H), 3.10-3.07 (m, 1H), 3.01-2.95 (m, 1H), 2.89 (d, J = 11.2 Hz, 1H), 2.71 (d, J = 15.3 Hz, 1H), 2.65-2.61 (m, 1H), 2.21 (t, J = 10.8 Hz, 1H), 2.03 (d, J = 12.8 Hz, 1H), 1.76 (t, J = 14.8 Hz, 2H), 1.66-1.61 (m, 1H), 1.54-1.35 (m, 4H), 1.30-1.24 (m, 2H); ^{13}C NMR (151 MHz, $CDCl_3$: CD_3OD, 10:1 v/v): δ 136.1, 133.9, 126.8, 121.0, 118.9, 117.8, 110.8, 106.8, 65.9, 61.1, 60.5, 52.9, 40.6, 38.4, 35.5, 34.6, 31.9, 23.7, 21.0; IR (neat): v 2925, 2856, 1263, 1095, 1024, 801 cm^{-1}; HRMS (ESI): m/z $[M + H]^+$ calcd. For $C_{19}H_{25}N_2O$: 297.1961; found: 297.1960; $[\alpha]_D^{23}$ = -62.4 (c = 0.10, MeOH).

Supplementary Table 17 ^{1}H NMR data comparison of the synthetic (−)-yohimbol

**Lit[11]. Natural ($CDCl_3$)**	**This work ($CDCl_3$:CD_3OD, 10:1 v/v)**	**Δδ** (Lit – Our synthetic)
7.38 (dd, J = 7.2 Hz, 1.1 Hz, 1H)	7.38 (d, J = 7.8 Hz, 1H)	0
7.26 (d, J = 7.2 Hz, 1.1 Hz, 1H)	7.26 (d, J = 8.2 Hz, 1H)	0
7.05 (td, J = 7.2 Hz, 1.1 Hz, 1H)	7.05 (t, J = 7.1 Hz, 1H)	0
6.98 (td, J = 7.2 Hz, 1.1 Hz, 1H)	6.99 (t, J = 7.3 Hz, 1H)	−0.01
4.19 (m, 1H)	4.07 (s, 1H)	0.12
3.57 (dd, J = 11.4 Hz, 1.7 Hz, 1H)	3.40 (d, J = 10.6 Hz, 1H)	0.17
3.25 (ddd, J = 12.2 Hz, 7.0, 2.3 Hz, 1H)	3.10-3.07 (m, 1H)	-
3.09 (m, 1H)	3.01-2.95 (m, 1H)	-
3.03 (dd, J = 11.5 Hz, 2.6 Hz, 1H)	2.89 (d, J = 11.2 Hz, 1H)	0.14
2.87-2.71 (m, 2H)	2.71 (d, J = 15.3 Hz, 1H)	-
	2.65-2.61 (m, 1H)	-
2.35 (dd, J = 11.5 Hz, 7.1, 1H)	2.21 (t, J = 10.8 Hz, 1H)	0.14
2.31 (dt, J = 12.9 Hz, 3.1 Hz, 1H)	2.03 (d, J = 12.8 Hz, 1H)	0.28
1.90 (m, 2H)	1.76 (t, J = 14.8 Hz, 2H)	0.14
1.85 (m, 1H)	1.66-1.61 (m, 1H)	-
1.7-1.1 (m, 6H)	1.54-1.35 (m, 4H)	-
	1.30-1.24 (m, 2H)	

Supplementary Table 18 ^{13}C NMR data comparison of the synthetic (−)-yohimbol

**Lit[11]. Natural ($CDCl_3$)**	**This work ($CDCl_3$:CD_3OD, 10:1 v/v)**	**Δδ** (Lit – Our synthetic)
138.1	136.1	2
135.2	133.9	1.3
128.3	126.8	1.5
122.1	121.0	1.1

119.9	118.9	1.0
118.6	117.8	0.8
112.0	110.8	1.2
107.5	106.8	0.7
67.1	65.9	1.2
62.3	61.1	1.2
62.2	60.5	1.7
54.3	52.9	1.4
42.2	40.6	1.6
39.8	38.4	1.4
36.8	35.5	1.3
36.0	34.6	1.4
33.1	31.9	1.2
25.1	23.7	1.4
22.2	21.0	1.2

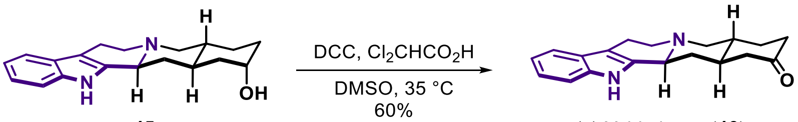

(−)-Yohimbone (46)

To a solution of **45** (8.0 mg, 0.02 mmol, 1.0 equiv) and DCC (21 mg, 0.1mmol, 5.0 equiv) in dry DMSO (0.5 mL) was added dichloroacetic acid (1.7 µL, 0.02 mmol, 1.0 equiv). After being stirred at 35 °C for 3 h, the mixture was diluted with EtOAc (0.4 mL). A solution of 16.3 mg of oxalic acid in MeOH (0.15 mL) was added. After 1 h, the suspension was diluted by H_2O (0.4 mL), filtrated and washed with EtOAc. The filtrate was diluted with H_2O (8 mL), basified with K_2CO_3 and extracted with DCM. The combined organic layers were dried over Na_2SO_4 and concentrated *in vacuo*. The residue was purified by column chromatography on silica gel (DCM: MeOH, 40:1) to give (−)-yohimbone (**46**).

White powder, isolated yield 60% (3.5 mg); m.p.: 253.2-253.8 °C; ^{1}H NMR (400 MHz, $CDCl_3$): δ 7.84 (s, 1H), 7.49 (d, J = 7.6 Hz, 1H), 7.32 (d, J = 7.8 Hz, 1H), 7.17 (t, J = 7.2 Hz, 1H), 7.11 (t, J = 7.1 Hz, 1H), 3.31 (d, J = 11.0 Hz, 1H), 3.12-3.08 (m, 2H), 3.06-2.97 (m, 1H), 2.77 (dd, J = 15.3, 4.3 Hz, 1H), 2.70 (td, J = 11.2, 4.3 Hz, 1H), 2.50-2.43 (m, 2H), 2.42 (td, J = 14.5, 6.7 Hz, 1H), 2.21 (t, J = 11.0 Hz, 2H), 2.11 (dt, J = 12.2, 3.0 Hz, 1H), 2.03-1.97 (m, 1H), 1.95-1.85 (m, 1H), 1.67-1.57 (m, 1H), 1.55-1.49 (m, 1H), 1.51 (qd, J = 12.7, 5.0 Hz, 1H); ^{13}C NMR (101 MHz, $CDCl_3$): δ 210.4, 136.0, 134.2, 127.3, 121.5, 119.5, 118.2, 110.8, 108.3, 60.7, 59.2, 53.1, 47.5, 41.5, 41.0, 40.3, 37.1, 29.9, 21.7; IR (neat): v 3322, 2927, 2857, 1696, 1459, 1265, 1095, 801, 736 cm^{-1}; HRMS (ESI): m/z $[M + H]^+$ calcd. For $C_{19}H_{23}N_2O$: 295.1805; found: 295.1803; $[\alpha]_D^{23}$ = −102.7 (c = 0.10, pyridine), for (+)-enantiomer: lit[6]. $[\alpha]_D^{20}$ = +96.7 (c = 0.07, pyridine).

Supplementary Table 19 ^{1}H NMR data comparison of the synthetic (−)-yohimbone

**Lit[6]. Synthetic**	**This work**	**Δδ** (Lit – Our synthetic)
10.44 (s, 1H)	7.84 (s, 1H)	2.6
7.30 (d, *J* = 8.0 Hz, 1H)	7.49 (d, *J* = 7.6 Hz, 1H)	−0.19
7.22 (d, *J* = 8.0 Hz, 1H)	7.32 (d, *J* = 7.8 Hz, 1H)	−0.1
6.95 (t, *J* = 7.6 Hz, 1H)	7.17 (t, *J* = 7.2 Hz, 1H)	−0.22
6.88 (t, *J* = 7.6 Hz, 1H)	7.11 (t, *J* = 7.1 Hz, 1H)	−0.23
3.24-3.20 (overlapped, 1H)	3.31 (d, *J* = 11.0 Hz, 1H)	-
3.01 (td, *J* = 10.8, 5.4 Hz, 2H)	3.12-3.08 (m, 2H)	-
2.88-2.80 (m, 1H)	3.06-2.97 (m, 1H)	-
2.64-2.54 (m, 2H)	2.77 (dd, *J* = 15.3, 4.3 Hz, 1H)<br>2.70 (td, *J* = 11.2, 4.3 Hz, 1H)	-
2.36 (td, *J* = 14.0, 6.0 Hz, 1H)<br>2.31-2.26 (m, 3H)<br>2.19-2.09 (m, 2H)	2.50-2.43 (m, 2H)<br>2.42 (td, *J* = 14.5, 6.7 Hz, 1H)<br>2.21 (t, *J* = 11.0 Hz, 2H)<br>2.11 (dt, *J* = 12.2, 3.0 Hz, 1H)	-
1.94-1.89 (m, 1H)	2.03-1.97 (m, 1H)	-
1.79 (q, *J* = 11.6 Hz, 1H)	1.95-1.85 (m, 1H)	-
1.56 (q, *J* = 11.6 Hz, 1H)	1.67-1.57 (m, 1H)	-
1.39 (td, *J* = 12.8, 4.8 Hz, 1H)	1.55-1.49 (m, 1H)	-
1.29 (q, *J* = 12.0 Hz, 1H)	1.51 (qd, *J* = 12.7, 5.0 Hz, 1H)	−0.22

Supplementary Table 20 ^{13}C NMR data comparison of the synthetic (−)-yohimbone

**Lit[6]. Synthetic**	**This work**	**Δδ** (Lit – Our synthetic)
209.0	210.4	−1.4
135.7	136.0	−0.3
134.7	134.2	0.5
126.3	127.3	−1.0
119.9	121.5	−1.6
117.9	119.5	−1.6
117.0	118.2	−1.2
110.5	110.8	−0.3
106.0	108.3	−2.3
60.0	60.7	−0.7
58.9	59.2	−0.3
52.4	53.1	−0.7
46.9	47.5	−0.6
40.8	41.5	−0.7
40.3	41.0	−0.7
overlapped	40.3	-
36.2	37.1	−0.9
29.2	29.9	−0.7
21.3	21.7	−0.4

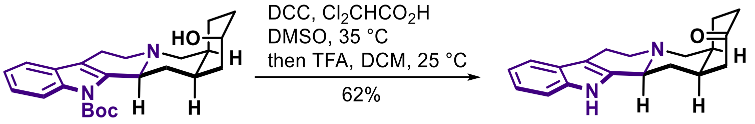

47

(−)-Alloyohimbone (**48**)

(−)-Alloyohimbone (48)

To a solution of **47** (31 mg, 0.08 mmol, 1.0 equiv) and DCC (84 mg, 0.4 mmol, 5.0 equiv) in dry DMSO (2 mL) was added dichloroacetic acid (7 μL, 0.08 mmol, 1.0 equiv). After being stirred at 35 °C for 3 h, the mixture was diluted with EtOAc (1.6 mL). A solution of 65 mg of oxalic acid in MeOH (0.6 mL) was added. After 1 h, the suspension was diluted by H_2O (1.6 mL), filtrated and washed with EtOAc. The filtrate was diluted with H_2O (30 mL), basified with K_2CO_3 and extracted with DCM. The combined organic layers were dried over Na_2SO_4 and concentrated in *vacuo*. The above crude product was dissolved in DCM (1.6 mL) followed by adding TFA (0.8 mL), The reaction mixture was stirred at 25 °C for 2 h. the reaction was quenched with saturated aqueous $NaHCO_3$ solution and extracted with DCM. The combined organic layers were washed with brine, dried over Na_2SO_4, filtered and concentrated *in vacuo*. The residue was purified by column chromatography on silica gel (DCM: MeOH, 20:1) to give (−)-alloyohimbone (**48**).

White solid, isolated yield 62% (15 mg); m.p.: 241.5-242.0 °C; ^1H NMR (400 MHz, $CDCl_3$): δ 7.87 (s, 1H), 7.47 (d, J = 7.6 Hz, 1H), 7.30 (d, J = 7.9 Hz, 1H), 7.14 (t, J = 7.1 Hz, 1H), 7.09 (t, J = 7.1 Hz, 1H), 3.25 (d, J = 10.6 Hz, 1H), 3.01-2.90 (m, 3H), 2.72-2.64 (m, 3H), 2.58-2.32 (m, 4H), 2.35-2.31 (m, 1H), 2.26 (d, J = 14.1 Hz, 2H), 1.92-1.85 (m, 2H), 1.67 (q, J = 12.7 Hz, 1H); ^{13}C NMR (101 MHz, $CDCl_3$): δ 211.3, 136.0, 134.4, 127.3, 121.4, 119.4, 118.1, 110.8, 108.3, 60.1, 59.9, 53.2, 46.8, 40.7, 37.9, 35.3, 31.6, 26.7, 21.7; IR (neat): ν 3354, 2857, 1633, 1438, 1280, 1027, 736 cm^{-1}; HRMS (ESI): m/z $[M + H]^+$ calcd. For $C_{19}H_{23}N_2O$: 295.1805; found: 295.1803; $[\alpha]_D^{23}$ = −130.6 (c = 0.30, EtOH), lit[12]. $[\alpha]_D^{23}$ = −133.7 (c = 1.01, EtOH).

Supplementary Table 21 ^1H NMR data comparison of the synthetic (−)-alloyohimbone

Lit[12]. Synthetic	This work	Δδ (Lit – Our synthetic)
7.95 (brs, 1H)	7.87 (s, 1H)	0.08
7.46 (d, J = 7.5 Hz, 1H)	7.47 (d, J = 7.6 Hz, 1H)	-0.01
7.30 (d, J = 7.5 Hz, 1H)	7.30 (d, J = 7.9 Hz, 1H)	0
7.12 (t, J = 7.5 Hz, 1H)	7.14 (t, J = 7.1 Hz, 1H)	-0.02
7.08 (t, J = 7.5 Hz, 1H)	7.09 (t, J = 7.1 Hz, 1H)	-0.01
3.21 (d, J = 11.5, 1H)	3.25 (d, J = 10.6 Hz, 1H)	-0.04
2.99-2.88 (m, 3H)	3.01-2.90 (m, 3H)	-
2.70-2.62 (m, 3H)	2.72-2.64 (m, 3H)	-
2.57-2.33 (m, 5H)	2.58-2.32 (m, 4H)	-
	2.35-2.31 (m, 1H)	
2.25-2.20 (m, 2H)	2.26 (d, J = 14.1 Hz, 2H)	-
1.89-1.82 (m, 2H)	1.92-1.85 (m, 2H)	-
1.60 (q, J = 13.0 Hz, 1H)	1.67 (q, J = 12.7 Hz, 1H)	-0.07

Supplementary Table 22 ^{13}C NMR data comparison of the synthetic (−)-alloyohimbone

Lit[12]. Synthetic	This work	Δδ (Lit – Our synthetic)
211.6	211.3	0.3
136.0	136.0	0
134.5	134.4	0.1
127.3	127.3	0
121.3	121.4	-0.1
119.4	119.4	0
118.0	118.1	-0.1
110.9	110.8	0.1
108.2	108.3	-0.1
60.2	60.1	0.1
59.9	59.9	0
53.2	53.2	0
46.8	46.8	0
40.7	40.7	0
37.9	37.9	0
35.3	35.3	0
31.6	31.6	0
26.8	26.7	0.1
21.8	21.7	0.1

2.10 Synthesis of (+)-arborescidine B and (+)-arborescidine C

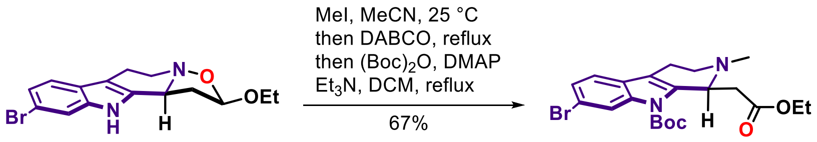

***tert*-butyl (*S*)-7-bromo-1-(2-ethoxy-2-oxoethyl)-2-methyl-1,2,3,4-tetrahydro-9*H*-pyrido[3,4-*b*]indole-9-carboxylate (49)**

To a solution of **3g** (296 mg, 0.88 mmol, 1.0 equiv) in MeCN (1.8 mL) was added MeI (187 mg, 1.32 mmol, 1.5 equiv). The reaction mixture was stirred at 25 °C for 6 h. Then DABCO (128 mg, 1.14 mmol, 1.3 equiv) was added and the reaction was refluxed for 1 h. The reaction solution was concentrated *in vacuo*. The above residue was dissolved in DCM (12 mL), and Et_3N (0.37 mL, 2.64 mmol, 3.0 equiv), DMAP (11 mg, 0.088 mmol, 0.1 equiv) and $(Boc)_2O$ (384 mg, 1.76 mmol, 2.0 equiv) were added. The reaction was stirred at 25 °C overnight then was concentrated *in vacuo*. The residue was purified by column chromatography on silica gel (petroleum ether: EtOAc, 2:1) to give **49**.

Pale yellow foam, isolated yield 67% (266 mg); ^{1}H NMR (400 MHz, $CDCl_3$) δ 8.35 (d, J = 1.2 Hz, 1H), 7.36 (dd, J = 8.3, 1.5 Hz, 1H), 7.29 (d, J = 8.3 Hz, 1H), 4.62 (dd, J = 10.5, 2.3 Hz, 1H), 4.27-4.13 (m, 2H), 3.24-3.16 (m, 1H), 2.98 (dd, J = 14.0, 6.0 Hz, 1H), 2.89-2.8 (m, 2H), 2.63 (dd, J = 15.5, 10.7 Hz, 1H), 2.50 (s, 3H), 2.48 (dd, J = 16.4, 5.0 Hz, 1H), 1.69 (s, 9H), 1.29 (t, J = 7.2 Hz, 3H); ^{13}C NMR (101

MHz, $CDCl_3$) δ 171.4, 149.8, 136.6, 135.2, 128.0, 125.8, 119.0, 118.9, 117.7, 114.4, 84.8, 60.3, 56.8, 43.0, 41.6, 39.5, 28.2, 16.2, 14.2; IR (neat): ν 2967, 2368, 1729, 1459, 1262, 1023 cm^{-1}; HRMS (ESI): m/z $[M + H]^+$ calcd. For $C_{21}H_{28}BrN_2O_4$: 451.1227; found: 451.1226; $[\alpha]_D^{23} = -8.7$ (c = 0.10, $CHCl_3$).

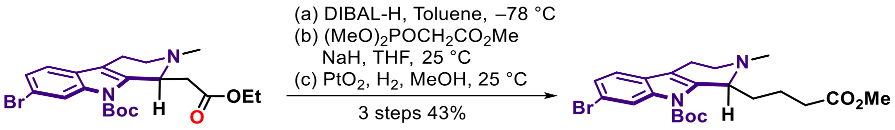

49 **50**

***tert*-butyl (*S*)-7-bromo-1-(4-methoxy-4-oxobutyl)-2-methyl-1,2,3,4-tetrahydro-9*H*-pyrido[3,4-*b*]indole-9-carboxylate (50)**

Under N_2, to a solution of **49** (126 mg, 0.28 mmol, 1.0 equiv) in dry toluene (1.4 mL) was added DIBAL-H (0.22 mL, 1.0 M in Hexane, 0.22 mmol, 0.8 equiv) at −78 °C. After being stirred for 30 min, the reaction was quenched with addition of aqueous saturated Rochelle salt solution and extracted with EtOAc. The combined organic layers were washed with brine, dried over Na_2SO_4, filtered and concentrated *in vacuo*. The residue was purified by column chromatography on silica gel (petroleum ether: EtOAc, 1:1) to the crude product. To a solution of NaH (12 mg, 60% in mineral oil, 0.5 mmol, 1.8 equiv) in THF (1.5 mL) was added $(MeO)_2POCH_2CO_2Me$ (102 mg, 0.56 mmol, 2.0 equiv). The reaction mixture was stirred at 25 °C for 30 min. Then a solution of the above crude product in THF (0.6 mL) was added and the reaction was stirred at 25 °C for 2 h before being quenched with aqueous saturated $NaHCO_3$ solution. The separated aqueous phase was extracted with EtOAc. The combined organic layers were washed with brine, dried over Na_2SO_4, filtered and concentrated *in vacuo*. The residue was purified by column chromatography on silica gel (petroleum ether: EtOAc, 4:1) to the crude product. PtO_2 (13 mg, 0.056 mmol, 0.2 equiv) was added to the MeOH (5 mL) solution of the above crude product. The reaction mixture was stirred at 25 °C under H_2 atmosphere for 1.5 h. The reaction solution was filtered through a pad of celite and concentrated *in vacuo*. The residue was purified by column chromatography on silica gel (petroleum ether: EtOAc, 4:1) to give **50**.

Yellow oil, isolated yield 43% for 3 steps (56 mg); ^1H NMR (400 MHz, $CDCl_3$) δ 8.26 (s, 1H), 7.34 (dd, J = 8.3, 1.6 Hz, 1H), 7.25 (d, J = 7.3 Hz, 1H), 4.07 (d, J = 9.3 Hz, 1H), 3.65 (s, 3H), 3.22-3.15 (m, 1H), 2.92-2.78 (m, 2H), 2.48 (s, 3H), 2.45-2.35 (m, 3H), 2.00-1.92 (m, 1H), 1.84-1.74 (m, 3H), 1.68 (s, 9H); ^{13}C NMR (101 MHz,$CDCl_3$) δ 174.2, 149.8, 137.5, 136.4, 128.2, 125.6, 118.9, 118.8, 117.2, 113.3, 84.3, 59.9, 51.4, 43.0, 41.9, 34.0, 33.5, 28.2, 22.3, 16.1; IR (neat): ν 2926, 2351, 1756, 1659, 1264, 1020, 801, 671 cm^{-1}; HRMS (ESI): m/z $[M + H]^+$ calcd. For $C_{22}H_{30}BrN_2O_4$: 465.1384; found: 465.1384; $[\alpha]_D^{23}$ = +10.0 (c = 0.10, $CHCl_3$).

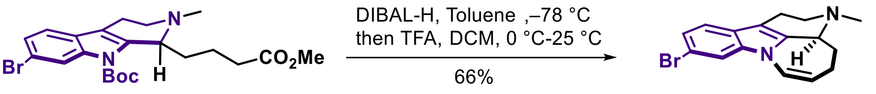

50 (+)-Arborescidine B (**51**)

(+)-Arborescidine B (51)

Under N_2, to a solution of **50** (19 mg, 0.04 mmol, 1.0 equiv) in dry toluene (1.4 mL) was added DIBAL-H (37 μL, 1.0 M in Hexane, 0.037 mmol, 0.9 equiv) at −78 °C. After being stirred for 30 min, the reaction

was quenched with addition of aqueous saturated Rochelle salt solution and extracted with EtOAc. The combined organic layers were washed with brine, dried over Na_2SO_4, filtered and concentrated *in vacuo*. The residue was purified by column chromatography on silica gel (petroleum ether: EtOAc, 1:1). The crude product was dissolved in DCM (2 mL) followed by adding TFA (0.8 mL, 0.05 M) at 0 °C. The reaction mixture was stirred at 25 °C for 2 h, then heated to 40 °C for 22 h. The reaction was quenched with aqueous saturated $NaHCO_3$ solution and extracted with DCM. The combined organic layers were washed with brine, dried over Na_2SO_4, filtered and concentrated *in vacuo*. The residue was purified by column chromatography on silica gel (DCM: MeOH, 20:1) to give (+)-arborescidine B (**51**).

Yellow oil, isolated yield 66% (8.4 mg); ^{1}H NMR (400 MHz, $CDCl_3$): δ 7.48 (d, J = 1.0 Hz, 1H), 7.33 (d, J = 8.3 Hz, 1H), 7.23 (d, J = 10.0 Hz, 1H), 6.83 (d, J = 9.8 Hz, 1H), 5.12-5.08 (m, 1H), 3.39 (d, J = 10.4 Hz, 1H), 3.16-3.10 (m, 1H), 2.94-2.85 (m, 1H), 2.75-2.66 (m, 2H), 2.56-2.51 (m, 4H), 2.46-2.32 (m, 2H), 1.92-1.85 (m, 1H); ^{13}C NMR (101 MHz, $CDCl_3$): δ 138.0, 136.9, 125.8, 123.2, 121.6, 119.3, 115.3, 112.3, 111.2, 109.3, 62.3, 52.6, 42.4, 29.8, 27.9, 20.6; IR (neat): v 2958, 2920, 1452, 1378, 1260, 1093, 1021 cm^{-1}; HRMS (ESI): m/z $[M + H]^+$ calcd. For $C_{16}H_{18}BrN_2$: 317.0648; found: 317.0649; $[\alpha]_D^{23}$ = +76.0 (c = 0.6, $CHCl_3$), for (−)-enantiomer: lit[13]. $[\alpha]_D$ = −71 (c = 0.6, $CHCl_3$).

Supplementary Table 23 ^{1}H NMR data comparison of the synthetic (+)-arborescidine B

**Lit[13]. Synthetic**	**This work**	**Δδ** (Lit – Our synthetic)
7.47 (d, J = 1.5 Hz, 1H)	7.48 (d, J = 1.0 Hz, 1H)	−0.01
7.31 (brd, J = 9.8 Hz, 1H)	7.33 (d, J = 8.3 Hz, 1H)	−0.02
7.21 (dd, J = 9.8, 1.8 Hz, 1H)	7.23 (d, J = 10.0 Hz, 1H)	−0.02
6.81 (dt, J = 9.8, 1.8 Hz, 1H)	6.83 (d, J = 9.8 Hz, 1H)	−0.02
5.09 (dt, J = 9.8, 4.1 Hz, 1H)	5.12-5.08 (m, 1H)	-
3.36 (d, J = 10.0 Hz, 1H)	3.39 (d, J = 10.4 Hz, 1H)	−0.03
3.12 (ddd, J = 15.0, 11.2, 2.4 Hz, 1H)	3.16-3.10 (m, 1H)	-
2.89 (dddd, J = 15.0, 11.2, 2.6, 2.4 Hz, 1H)	2.94-2.85 (m, 1H)	-
2.70 (dd, J = 11.2, 3.9 Hz, 1H)	2.75-2.66 (m, 2H)	-
2.68 (dd, J = 12.4, 4.1 Hz, 1H)		
2.54 (s, 3H)	2.56-2.51 (m, 4H)	-
2.57-2.49 (m, 1H)		
2.44-2.40 (m, 1H)	2.46-2.32 (m, 2H)	-
2.36-2.35 (m, 1H)		
1.86 (dq, J = 10.0, 4.3 Hz, 1H)	1.92-1.85 (m, 1H)	-

Supplementary Table 24 ^{13}C NMR data comparison of the synthetic (+)-arborescidine B

**Lit[13]. Synthetic**	**This work**	**Δδ** (Lit – Our synthetic)
138.0	138.0	0
136.9	136.9	0
125.8	125.8	0
123.3	123.2	0.1

121.7	121.6	0.1
119.3	119.3	0
115.3	115.3	0
112.4	112.3	0.1
111.2	111.2	0
109.3	109.3	0
62.4	62.3	0.1
52.6	52.6	0
42.4	42.4	0
29.9	29.8	0.1
27.9	27.9	0
20.6	20.6	0

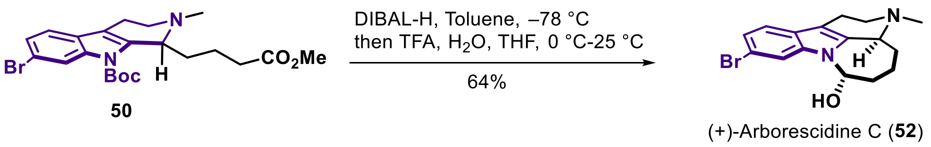

(+)-Arborescidine C (52)

Under N_2, to a solution of **50** (19 mg, 0.04 mmol, 1.0 equiv) in dry toluene (1.4 mL) was added DIBAL-H (37 µL, 1.0 M in Hexane, 0.037 mmol, 0.9 equiv) at −78 °C. After 30 min, the reaction was quenched with aqueous saturated Rochelle salt solution and extracted with with EtOAc. The combined organic layers were washed with brine, dried over Na_2SO_4, filtered and concentrated *in vacuo*. The residue was purified by column chromatography on silica gel (petroleum ether: EtOAc, 1:1) to give the crude product. The crude product was dissolved in DCM (2 mL) followed by adding TFA (0.8 mL, 0.05 M) at 0 °C. The reaction mixture was stirred at 25 °C for 1.5 h. The reaction solution was concentrated *in vacuo*. The above residue was dissolved in THF (0.34 mL) followed by adding H_2O (0.36 mL) and TFA (0.13 mL). The reaction mixture was stirred at 25 °C for 12 h before being quenched with aqueous saturated $NaHCO_3$ solution. The separated aqueous phase was extracted with DCM. The combined organic layers were washed with brine, dried over Na_2SO_4, filtered and concentrated *in vacuo*. The residue was purified by column chromatography on silica gel (DCM: MeOH, 20:1) to give (+)-arborescidine C (**52**).

White solid, isolated yield 64% (9.1 mg); m.p.: 171.2-172.0 °C; ^1H NMR (400 MHz, $CDCl_3$): δ 7.42 (s, 1H), 7.26 (d, J = 4.5 Hz, 1H), 7.16 (d, J = 8.3 Hz, 1H), 6.05 (d, J = 4.2 Hz, 1H), 3.59 (d, J = 10.7 Hz, 1H), 2.94-2.89 (m, 1H), 2.68-2.61 (m, 3H), 2.40 (s, 3H), 2.28 (d, J = 17.2 Hz, 1H), 2.18-2.03 (m, 2H), 1.77 (d, J = 15.9 Hz, 1H), 1.57 (t, J = 14.1 Hz, 1H), 1.40 (q, J = 15.5 Hz, 1H); ^{13}C NMR (101 MHz, $CDCl_3$): δ 138.2, 136.7, 125.5, 122.4, 119.4, 114.6, 111.6, 108.6, 76.5, 60.8, 50.0, 42.5, 34.2, 31.2, 20.1, 20.0; IR (neat): v 3350, 2920, 2876, 1458, 1021, 801, 739 cm^{-1}; HRMS (ESI): m/z $[M + H]^+$ calcd. For $C_{16}H_{20}BrN_2O$: 335.0754; found: 335.0754; $[\alpha]_D^{23}$ = +3.2 (c = 1.0, $CHCl_3$), for (−)-enantiomer: lit[14]. $[\alpha]_D$ = −3.1 (c = 1.0, $CHCl_3$).

Supplementary Table 25 ^1H NMR data comparison of the synthetic (+)-arborescidine C

**Lit[14]. Synthetic**	**This work**	**Δδ** (Lit – Our synthetic)

7.41 (s, 1H)	7.42 (s, 1H)	−0.01
7.22 (d, *J* = 10.2 Hz, 1H)	7.26 (d, *J* = 4.5 Hz, 1H)	−0.04
7.14 (d, *J* = 10.2 Hz, 1H)	7.16 (d, *J* = 8.3 Hz, 1H)	−0.02
6.00 (d, *J* = 2.9 Hz, 1H)	6.05 (d, *J* = 4.2 Hz, 1H)	−0.05
3.55 (d, *J* = 13.9 Hz, 1H)	3.59 (d, *J* = 10.7 Hz, 1H)	−0.04
2.89 (dd, *J* = 13.3, 5.9 Hz, 1H)	2.94-2.89 (m, 1H)	-
2.70-2.60 (m, 3H)	2.68-2.61 (m, 3H)	-
2.37 (s, 3H)	2.40 (s, 3H)	−0.03
2.23 (brd, *J* = 16.7 Hz, 1H)	2.28 (d, *J* = 17.2 Hz, 1H)	−0.05
2.13 (d, *J* = 14.5 Hz, 1H)	2.18-2.03 (m, 2H)	-
2.13 (q, *J* = 15.8 Hz, 1H)		
1.73 (d, *J* = 16.6 Hz, 1H)	1.77 (d, *J* = 15.9 Hz, 1H)	−0.04
1.52 (t, *J* = 16.6 Hz, 1H)	1.57 (t, *J* = 14.1 Hz, 1H)	−0.05
1.33 (q, *J* = 15.6 Hz, 1H)	1.40 (q, *J* = 15.5 Hz, 1H)	−0.07

Supplementary Table 26 ^{13}C NMR data comparison of the synthetic (+)-arborescidine C

**Lit[14]. Synthetic**	**This work**	**Δδ** (Lit – Our synthetic)
138.5	138.2	0.3
137.1	136.7	0.4
125.8	125.5	0.3
122.6	122.4	0.2
119.7	119.4	0.3
114.9	114.6	0.3
111.9	111.6	0.3
108.9	108.6	0.3
76.8	76.5	0.3
61.1	60.8	0.3
50.2	50.0	0.2
42.8	42.5	0.3
34.5	34.2	0.3
31.5	31.2	0.3
20.5	20.1	0.4
20.3	20.0	0.3

2.11 Synthesis of eburnamine family alkaloids

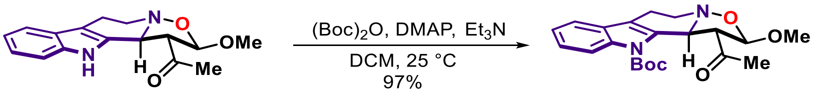

5a **53**

***tert*-butyl (1*R*, 2*R*, 11b*S*)-1-acetyl-2-methoxy-1,5,6,11b-tetrahydroisoxazolo[2',3':1,2]pyrido[3,4-*b*]indole-11(2*H*)-carboxylate (53)**

To a stirred solution of **5a** (2.42 g, 8.44 mmol, 1.0 equiv) in DCM (84.4 mL) was added Et_3N (2.56 g, 25.32 mmol, 3.0 equiv), DMAP (103 mg, 0.84 mmol, 0.1 equiv) and $(Boc)_2O$ (3.68 g, 16.88 mmol, 2.0 equiv). The reaction was stirred at 25 °C overnight then was concentrated *in vacuo*. The residue was purified by column chromatography on silica gel (petroleum ether: EtOAc, 40:1-20:1) to give **53**.

White oil, isolated yield 97% (3.16 g); ^{1}H NMR (400 MHz, $CDCl_3$): δ 7.96 (d, J = 7.8 Hz, 1H), 7.46 (d, J = 7.2 Hz, 1H), 7.31-7.23 (m, 2H), 5.34 (d, J = 5.5 Hz, 1H), 5.24 (d, J = 3.4 Hz, 1H), 3.80-3.75 (m, 1H), 3.62 (dd, J = 5.5, 3.4 Hz, 1H), 3.38 (s, 3H), 3.36-3.30 (m, 1H), 2.94-2.86 (m, 1H), 2.76-2.68 (m, 1H), 2.35 (s, 3H), 1.66 (s, 9H); ^{13}C NMR (101 MHz, $CDCl_3$): δ 205.0, 150.4, 135.3, 132.1, 128.6, 124.3, 122.8, 118.3, 116.1, 115.7, 108.4, 84.4, 67.4, 62.9, 56.5, 49.3, 30.0, 28.1, 18.3; IR (neat): v 2926, 1731, 1460, 1376, 1317, 1146, 1117, 749 cm^{-1}; HRMS (ESI): m/z $[M + H]^+$ calcd. for $C_{21}H_{27}N_2O_5$: 387.1915; found: 387.1910; $[\alpha]_D^{23}$ = −65.9 (c = 0.85, DCM); **ent-53**: $[\alpha]_D^{23}$ = +66.2 (c = 0.85, DCM).

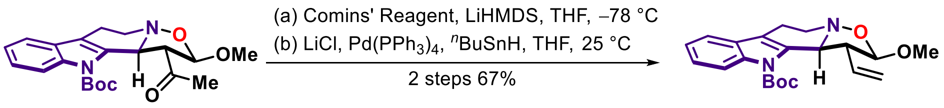

53 **54**

***tert*-butyl (1*R*, 2*R*, 11b*S*)-2-methoxy-1-vinyl-1,5,6,11*b*-tetrahydroisoxazolo[2',3':1,2]pyrido[3,4-b]indole-11(2*H*)-carboxylate (54)**

Under N_2, to a solution of **53** (3.16 g, 8.18 mmol, 1.0 equiv) and N-(5-Chloro-2-pyridyl)bis(trifluoromethanesulfonimide) (6.42 g, 16.36 mmol, 2.0 equiv) in dry THF (82 mL) was added LiHMDS (40.9 mL, 1.0 M in THF, 40.9 mmol, 5.0 equiv) dropwise at −78 ℃. The resulting mixture was stirred overnight. The reaction was quenched with saturated aqueous NH_4Cl and extracted with EtOAc. The combined organic layers were washed with brine, dried over Na_2SO_4 and concentrated in *vacuo*. The residue was purified by column chromatography on silica gel (petroleum ether: EtOAc, 20:1) to give the product (2.89 g). Under N_2, to a solution of the above product (2.89 g, 5.57 mmol, 1.0 equiv), LiCl (709 mg, 16.71 mmol, 3.0 equiv) and $Pd(PPh_3)_4$ (647 mg, 0.56 mmol, 0.1 equiv) in dry THF (111 mL) was added *n*-Bu_3SnH (2.43 g, 8.36 mmol, 1.5 equiv) at 25 ℃. The reaction was stirred at 25 ℃ for 2 h then was quenched with saturated aqueous NH_4Cl and extracted with EtOAc. The combined organic layers were washed with brine, dried over Na_2SO_4 and concentrated in *vacuo*. The residue was purified by column chromatography on silica gel (petroleum ether: EtOAc, 20:1) to give **54**.

White solid, isolated yield 67% for 2 steps (2.03 g); m.p.: 105.8-106.3 ℃; ^{1}H NMR (400 MHz, $CDCl_3$): δ 8.01 (d, J = 8.0 Hz, 1H), 7.47 (d, J = 7.1 Hz, 1H), 7.31 (t, J = 7.8 Hz, 1H), 7.26 (t, J = 7.7 Hz, 1H), 6.19-6.10 (m, 1H), 5.28-5.16 (m, 3H), 4.93 (d, J = 2.2 Hz, 1H), 3.82 (ddd, J = 12.7, 10.4, 5.2 Hz, 1H), 3.34-3.24 (m, 5H), 2.91-2.84 (m, 1H), 2.76-2.69 (m, 1H), 1.68 (s, 9H); ^{13}C NMR (101 MHz, $CDCl_3$): δ 150.3, 136.0, 135.9, 132.7, 128.6, 124.0, 122.5, 118.1, 117.1, 115.5, 109.4, 83.8, 63.4, 60.1, 56.0, 49.3, 28.2 (2C), 18.2; IR (neat): v 2927, 1723, 1461, 1373, 1179, 1069, 756, 663 cm^{-1}; HRMS (ESI): m/z $[M + H]^+$ calcd. for $C_{21}H_{27}N_2O_4$: 371.1965; found: 371.1961; $[\alpha]_D^{23}$ = −125.0 (c = 1.10, DCM); **ent-54**: $[\alpha]_D^{23}$ = +125.2 (c = 1.0, DCM).

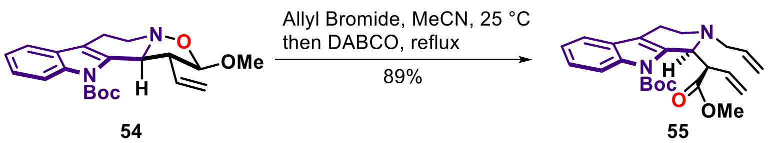

tert-butyl (*S*)-2-allyl-1-((*R*)-1-methoxy-1-oxobut-3-en-2-yl)-1,2,3,4-tetrahydro-9*H*-pyrido[3,4-*b*]indole-9-carboxylate (54)

To a solution of **54** (2.03 g, 5.48 mmol, 1.0 equiv) in MeCN (55 mL) was added allyl bromide (796 mg, 6.58 mmol, 1.2 equiv). The reaction mixture was stirred at 25 °C for 1 h. Then DABCO (738 mg, 6.58 mmol, 1.2 equiv) was added and the reaction was refluxed for 1 h. The reaction solution was concentrated *in vacuo*. The residue was purified by column chromatography on silica gel (petroleum ether: EtOAc, 20:1) to give **55**.

White oil, isolated yield 89% (2.00 g); ^{1}H NMR (400 MHz, $CDCl_3$): δ 8.12 (d, J = 8.2 Hz, 1H), 7.46-7.44 (m, 1H), 7.32 (td, J = 7.2, 1.5 Hz, 1H), 7.26 (td, J = 7.6, 1.3 Hz, 1H), 6.11 (ddd, J = 19.6, 17.2, 9.9 Hz, 1H), 5.91-5.81 (m, 1H), 5.14-5.08 (m, 2H), 5.04 (d, J = 10.3 Hz, 1H), 5.00 (dd, J = 10.1, 1.3 Hz, 1H), 4.88 (d, J = 17.1 Hz, 1H), 3.74 (s, 3H), 3.49-3.40 (m, 2H), 3.26-3.22 (m, 2H), 3.07 (dd, J = 14.6, 6.7 Hz, 1H), 2.90-2.81 (m, 1H), 2.58 (dd, J = 16.7, 5.5 Hz, 1H), 1.68 (s, 9H); ^{13}C NMR (101 MHz, $CDCl_3$): δ 172.6, 150.5, 136.6, 136.2, 134.1, 133.4, 129.0, 124.1, 122.4, 118.3, 117.9, 117.1, 115.5, 114.9, 83.8, 58.9, 57.8, 56.5, 51.8, 41.6, 28.2, 16.7; IR (neat): v 3864, 2957, 1739, 1460, 1371, 1324, 1143, 748 cm^{-1}; HRMS (ESI): m/z [M + H]$^+$ calcd. for $C_{24}H_{31}N_2O_4$: 411.2278; found: 411.2275; $[\alpha]_D^{23}$ = −16.0 (c = 0.50, DCM); **ent-55**: $[\alpha]_D^{23}$ = +16.0 (c = 0.50, DCM).

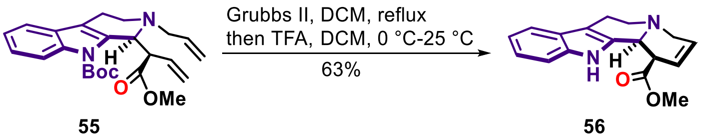

methyl (1*S*, 12b*S*)-1,4,6,7,12,12b-hexahydroindolo[2,3-*a*]quinolizine-1-carboxylate (56)

To a solution of **55** (2.00 g, 4.87 mmol, 1.0 equiv) in dry DCM (325 mL) was added Grubbs II catalyst (416 mg, 0.49 mmol, 0.1 equiv). The reaction mixture was stirred at 40 °C for 2 h under N_2 atmosphere. The reaction solution was concentrated *in vacuo*. The above residue was dissolved in DCM (97 mL), and TFA (48.5 mL) was added at 0 °C. The reaction was stirred at 25 °C 4 h. The reaction mixture was concentrated in *vacuo* and aqueous saturated Na_2CO_3 was added until pH > 7 was achieved. The aqueous layer was extracted with EtOAc. The combined organic layers were washed with brine, dried over Na_2SO_4 and concentrated in *vacuo*. The residue was purified by column chromatography on silica gel (petroleum ether: EtOAc, 4:1) to give **56**.

Yellow solid, isolated yield 63% (866 mg); m.p.: 139.5-140.4 °C; ^{1}H NMR (400 MHz, $CDCl_3$): δ 7.88 (s, 1H), 7.50 (d, J = 7.2 Hz, 1H), 7.33 (d, J = 7.7 Hz, 1H), 7.16-7.07 (m, 2H), 6.05-6.01 (m, 1H), 5.90-5.85 (m, 1H), 3.84-3.83 (m, 1H), 3.62-3.57 (m, 2H), 3.42 (s, 3H), 3.27 (dd, J = 11.2, 5.3 Hz, 1H), 3.09-2.97 (m, 2H), 2.75-2.71 (m, 1H), 2.65 (ddd, J = 15.3, 11.4, 3.9 Hz, 1H); ^{13}C NMR (101 MHz, $CDCl_3$): δ 171.1, 136.5, 131.8, 129.0, 127.0, 121.8, 121.5, 119.3, 118.1, 110.9, 110.3, 57.8, 54.3, 52.5, 52.1, 45.4, 21.0; IR (neat): v 3864, 1847, 1739, 1658, 1653, 1554, 1461, 670 cm^{-1}; HRMS (ESI): m/z [M + H]$^+$ calcd. for $C_{17}H_{19}N_2O_2$: 283.1441; found: 283.1437; $[\alpha]_D^{23}$ = −508.8 (c = 0.50, DCM); **ent-56**: $[\alpha]_D^{23}$ = +508.8 (c = 0.50, DCM).

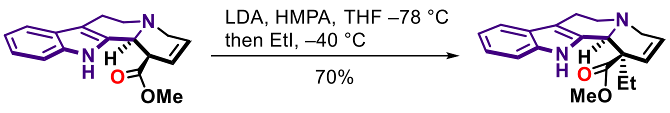

56 **57**

methyl (1*R*, 12b*S*)-1-ethyl-1,4,6,7,12,12b-hexahydroindolo[2,3-*a*]quinolizine-1-carboxylate (57)

Under N_2, the solution of LDA (5.11 mL, 1.32 M in THF, 6.75 mmol, 2.2 equiv) was added HMPA (1.10 g, 6.14 mmol, 2.0 equiv) at −78 ºC. After 30 min, the resulting mixture was added the solution of **56** (866 mg, 3.07 mmol, 1.0 equiv) in THF (15 mL) at −78 ºC and stirred for 20 min then warmed to −40 ºC and stirred for 20 min. The reaction was added EtI (507 mg, 3.25 mmol, 1.06 equiv) and stirred for 40 min at −40 ºC. The reaction was quenched with saturated aqueous NH_4Cl and extracted with EtOAc. The combined organic layers were washed with brine, dried over Na_2SO_4 and concentrated in *vacuo*. The residue was purified by column chromatography on silica gel (petroleum ether: EtOAc, 5:1) to give **57**.

Yellow solid, isolated yield 70% (667 mg); m.p.: 131.3-131.8; ^{1}H NMR (400 MHz, $CDCl_3$): δ 7.89 (s, 1H), 7.49 (d, J = 7.6 Hz, 1H), 7.34 (d, J = 7.9 Hz, 1H), 7.17-7.07 (m, 2H), 5.99-5.95 (m, 1H), 5.80 (d, J = 10.2 Hz, 1H), 3.84 (s, 1H), 3.50-3.44 (m, 4H), 3.22 (dd, J = 10.6, 4.9 Hz, 1H), 3.12 (d, J = 16.8 Hz, 1H), 2.99-2.92 (m, 1H), 2.71-2.68 (m, 2H), 2.25-2.17 (m, 1H), 2.13-2.04 (m, 1H), 1.11 (t, J = 7.4 Hz, 3H); ^{13}C NMR (101 MHz, $CDCl_3$): δ 174.3, 136.3, 132.3, 127.7, 127.0, 126.8, 121.6, 119.3, 118.0, 111.2, 110.9, 62.0, 53.6, 52.8, 52.3, 52.2, 30.4, 20.8, 8.8; IR (neat): ν 2927, 1738, 1722, 1563, 1544, 1451, 1229, 748, 670 cm^{-1}; HRMS (ESI): m/z [M + H]$^+$ calcd. for $C_{19}H_{23}N_2O_2$: 311.1754; found: 311.1752; $[\alpha]_D^{23}$ = −225.9 (c = 0.50, DCM); **ent-57**: $[\alpha]_D^{23}$ = +227.2 (c = 0.50, DCM).

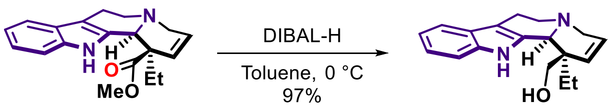

57 **58**

((1*R*, 12b*S*)-1-ethyl-1,4,6,7,12,12b-hexahydroindolo[2,3-*a*]quinolizin-1-yl)methanol (58)

Under N_2, to a solution of **57** (550 mg, 1.77 mmol, 1.0 equiv) in dry toluene (18 mL) at 0 °C was added dropwise DIBAL-H (3.89 mL, 1.0 M in Hexane, 3.89 mmol, 2.2 equiv) and the mixture was stirred for 30 min at 0 °C. The reaction was quenched with MeOH and warmed to 25 °C within 30 min. Rochelle salts were added and the mixture was stirred for 1 h. The aqueous layer was extracted with EtOAc. The combined organic layers were washed with brine, dried over Na_2SO_4 and concentrated in *vacuo*. The residue was purified by column chromatography on silica gel (petroleum ether: EtOAc, 1:1) to give **58**.

White solid, isolated yield 97% (485 mg); m.p.: 241.9-242.5 °C; ^{1}H NMR (400 MHz, $CDCl_3$): δ 7.80 (s, 1H), 7.52 (d, J = 7.6 Hz, 1H), 7.33 (d, J = 7.8 Hz, 1H), 7.19-7.10 (m, 2H), 6.28 (s, 1H), 6.10-6.07 (m, 1H), 5.55 (d, J = 10.0 Hz, 1H), 3.86 (s, 1H), 3.46-3.39 (m, 2H), 3.21-3.00 (m, 4H), 2.78 (d, J = 14.4 Hz, 1H), 2.62-2.56 (m, 1H), 1.78-1.69 (m, 1H), 1.52-1.43 (m, 1H), 1.14 (t, J = 7.5 Hz, 3H); ^{13}C NMR (101 MHz, $CDCl_3$): δ 136.3, 132.3, 131.1, 127.6, 126.7, 121.9, 119.5, 118.2, 112.1, 110.7, 67.8, 61.9, 54.7, 52.1, 44.7, 28.2, 21.5, 8.6; IR (neat): ν 3462, 1704, 1658, 1563, 1544, 1461, 670 cm^{-1}; HRMS (ESI): m/z [M + H]$^+$ calcd. for $C_{18}H_{23}N_2O$: 283.1805; found: 283.1801; $[\alpha]_D^{23}$ = −232.9 (c = 0.50, DCM); **ent-58**: $[\alpha]_D^{23}$ = +231.9 (c = 0.50, DCM).

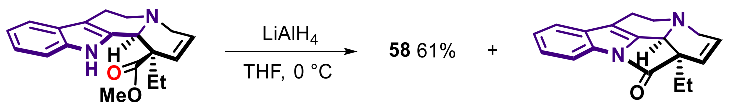

57 **59** 35%

(−)-14,15-Dihydro-17-nor-eburnamonine (59)

To a solution of **57** (93 mg, 0.30 mmol, 1.0 equiv) in THF (3.0 mL) was added $LiAlH_4$ (0.60 mL, 1.0 M in THF, 0.60 mmol, 2.0 equiv) at 0 °C. After being stirred at 0 °C for 1 h, the reaction mixture was quenched with H_2O. The aqueous layer was extracted with EtOAc. The combined organic layers were washed with brine, dried over Na_2SO_4 and concentrated in *vacuo*. The residue was purified by column chromatography on silica gel (petroleum ether: EtOAc, 1:1) to give **58** (61%) and **59** (35%).

Yellow solid, isolated yield 35% (29 mg); m.p.: 92.9-93.6 °C; ^{1}H NMR (400 MHz, $CDCl_3$): δ 7.90-7.87 (m, 1H), 7.47-7.45 (m, 1H), 7.31-7.28 (m, 2H), 5.80-5.76 (m, 1H), 5.60-5.56 (m, 1H), 4.34 (s, 1H), 3.42-3.39 (m, 2H), 2.98-2.87 (m, 2H), 2.58-2.50 (m, 2H), 2.06-1.99 (m, 2H), 1.12 (t, J = 7.4 Hz, 3H); ^{13}C NMR (101 MHz, $CDCl_3$): δ 172.6, 141.1, 134.5, 134.4, 128.2, 125.0, 124.2, 123.6, 119.3, 115.0, 109.2, 59.5, 55.0, 49.1, 42.2, 29.3, 17.6, 9.1; IR (neat): v 3865, 3445, 2930, 1747, 1659, 1446, 1325, 1291, 730 cm^{-1}; HRMS (ESI): m/z $[M + H]^+$ calcd. for $C_{18}H_{19}N_2O$: 279.1492; found: 279.1491; $[\alpha]_D^{23}$ = −10.3 (c = 0.20, $CHCl_3$).

Supplementary Table 27 ^{1}H NMR data comparison of the synthetic (−)-14,15-dihydro-17-nor-eburnamonine

**Lit[15]. Synthetic**	**This work**	**Δδ** (Lit – Our synthetic)
7.87 (m, 1H)	7.90-7.87 (m, 1H)	-
7.43 (m, 1H)	7.47-7.45 (m, 1H)	-
7.25 (m, 2H)	7.31-7.28 (m, 2H)	-
5.73 (ddd, J = 10.5, 6.0, 1.5 Hz, 1H)	5.80-5.76 (m, 1H)	-
5.55 (dd, J = 10.5, 2.0 Hz, 1H)	5.60-5.56 (m, 1H)	-
4.33 (s, 1H)	4.34 (s, 1H)	−0.01
3.40 (m, 2H)	3.42-3.39 (m, 2H)	-
2.90 (m, 2H)	2.98-2.87 (m, 2H)	-
2.53 (m, 2H)	2.58-2.50 (m, 2H)	-
2.02 (q, J = 7.5, 2H)	2.06-1.99 (m, 2H)	-
1.10 (t, J = 7.5 Hz, 3H)	1.12 (t, J = 7.4 Hz, 3H)	−0.02

Supplementary Table 28 ^{13}C NMR data comparison of the synthetic (−)-14,15-dihydro-17-nor-eburnamonine

**Lit[15]. Synthetic**	**This work**	**Δδ** (Lit – Our synthetic)
172.7	172.6	0.1
141.4	141.1	0.3
134.7 (2C)	134.5	-
	134.4	-

128.3	128.2	0.1
125.2	125.0	0.2
124.3, 123.8 (4C)	124.2	-
	123.6	-
	119.3	-
	115.0	-
Missing	109.2	-
59.7	59.5	0.2
55.3	55.0	0.3
49.4	49.1	0.3
42.5	42.2	0.3
29.9	29.3	0.6
17.8	17.6	0.2
9.2	9.1	0.1

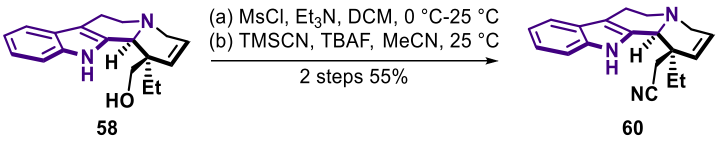

2-((1*R*, 12b*S*)-1-ethyl-1,4,6,7,12,12b-hexahydroindolo[2,3-*a*]quinolizin-1-yl)acetonitrile (60)

Under N_2, to a solution of **58** (379 mg, 1.34 mmol, 1.0 equiv) in dry DCM (13.4 mL) was added Et_3N (163 mg, 1.61 mmol, 1.2 equiv) and followed by addition of MsCl (185 mg, 1.61 mmol, 1.2 equiv). The reaction was stirred for 2 h, then was quenched by addition of saturated aqueous NH_4Cl and extracted with EtOAc. The combined organic layers were washed with brine, dried over Na_2SO_4 and concentrated in *vacuo*. The above residue was dissolved in MeCN (13.4 mL), and TMSCN (479 mg, 4.83 mmol, 3.0 equiv) and TBAF (4.83 mL, 1.0 M in THF, 4.83 mmol, 3.0 equiv) were added. The reaction was stirred at 25 °C for 5 d then was concentrated *in vacuo*. The residue was purified by column chromatography on silica gel (petroleum ether: EtOAc, 5:1) to give **60**.

Yellow solid, isolated yield 55% (215 mg); m.p.: 147.0-147.6 °C ; ^{1}H NMR (400 MHz, $CDCl_3$): δ 7.76 (s, 1H), 7.53 (d, J = 7.7 Hz, 1H), 7.38 (d, J = 8.0 Hz, 1H), 7.22 (t, J = 7.2 Hz, 1H), 7.16 (t, J = 7.8 Hz, 1H), 6.03 (ddd, J = 10.0, 4.6, 1.7 Hz, 1H), 5.67 (dt, J = 10.0, 1.9 Hz, 1H), 3.77 (s, 1H), 3.42 (ddd, J = 16.8, 4.6, 1.7 Hz, 1H), 3.16-3.08 (m, 2H), 2.97-2.89 (m, 2H), 2.74-2.61 (m, 2H), 2.06-1.95 (m, 3H), 1.21 (t, J = 7.6 Hz, 3H); ^{13}C NMR (101 MHz, $CDCl_3$): δ 136.3, 131.5, 130.6, 128.6, 126.5, 122.1, 119.7, 119.0, 118.1, 113.0, 110.9, 59.5, 54.9, 51.6, 42.4, 30.1, 26.2, 21.6, 8.6; IR (neat): ν 3864, 2097, 1777, 1739, 1658, 1563, 1544, 1461, 748, 670 cm^{-1}; HRMS (ESI): m/z $[M + H]^+$ calcd. for $C_{19}H_{22}N_3$: 292.1808; found: 292.1803; $[\alpha]_D^{23}$ = −185.7 (c = 0.20, $CHCl_3$); **ent-60**: $[\alpha]_D^{23}$ = +185.8 (c = 0.20, $CHCl_3$).

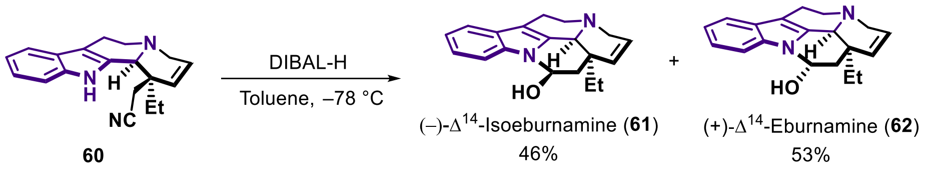

(−)-Δ^{14}-Isoeburnamine (61) and **(+)-Δ^{14}-Eburnamine (62)**

Under N_2, to a solution of **60** (100 mg, 0.34 mmol, 1.0 equiv) in dry toluene (3.4 mL) at -78 °C was added dropwise DIBAL-H (0.75 mL, 1.0 M in Hexane, 0.75 mmol, 2.2 equiv) and the mixture was stirred for 30 min at −78°C. The reaction was quenched with Rochelle salts and the mixture was stirred for 1 h. The aqueous layer was extracted with EtOAc. The combined organic layers were washed with brine, dried over Na_2SO_4 and concentrated in *vacuo*. The residue was purified by column chromatography on silica gel (DCM: MeOH, 200:1-100:1) to give **61** and **62**.

61: White solid, isolated yield 46% (46 mg); m.p.: 153.3-154.0 °C ; ^{1}H NMR (400 MHz, $CDCl_3$): δ 7.49-7.45 (m, 2H), 7.22 (t, J = 7.3 Hz, 1H), 7.16 (t, J = 7.6 Hz, 1H), 5.92 (d, J = 10.1 Hz, 1H), 5.81 (d, J = 10.3 Hz, 1H), 5.69 (dt, J = 10.2, 3.0 Hz, 1H), 4.05 (s, 1H), 3.47 (dd, J = 14.0, 7.2 Hz, 1H), 3.38-3.31 (m, 1H), 3.16-3.07 (m, 3H), 2.63 (ddd, J = 16.3, 6.4, 1.8 Hz, 1H), 2.42-2.36 (m, 2H), 2.19 (dd, J = 14.6, 3.7 Hz, 1H), 1.95-1.86 (m, 1H), 1.69-1.60 (m, 1H), 1.05 (t, J = 7.6 Hz, 3H); ^{13}C NMR (101 MHz, $CDCl_3$): δ 135.2, 130.7, 128.9, 127.4 (2C), 121.3, 120.1, 118.1, 110.6, 106.0, 75.1, 57.3, 49.7, 43.8, 39.7, 36.2, 34.7, 16.6, 8.5; IR (neat): v 3866, 3659, 1771, 1739, 1658, 1563, 1461, 670 cm^{-1}; HRMS (ESI): m/z [M + H]$^+$ calcd. for $C_{19}H_{23}N_2O$: 295.1805; found: 295.1803; $[\alpha]_D^{23}$ = −68.7 (c = 0.40, DCM); **ent-61**: $[\alpha]_D^{23}$ = +68.0 (c = 0.40, DCM).

62: White solid, isolated yield 53% (53 mg); m.p.: 142.1-143.0 °C ; ^{1}H NMR (400 MHz, $CDCl_3$): δ 7.75-7.73 (m, 1H), 7.48-7.45 (m, 1H), 7.19-7.12 (m, 2H), 5.43-5.35 (m, 2H), 5.16-5.13 (m, 1H), 3.67 (s, 1H), 3.36 (dd, J = 13.8, 7.2 Hz, 1H), 3.24-3.17 (m, 1H), 3.12-3.04 (m, 1H), 2.98 (d, J = 17.1 Hz, 1H), 2.78 (d, J = 17.5 Hz, 1H), 2.53 (dd, J = 16.0, 6.2 Hz, 1H), 2.21-2.17 (m, 1H), 1.83-1.74 (m, 1H), 1.50-1.39 (m, 2H), 0.95 (t, J = 7.6 Hz, 3H); ^{13}C NMR (101 MHz, $CDCl_3$): δ 136.9, 133.3, 128.7, 126.8, 126.6, 121.2, 120.0, 117.9, 112.5, 105.5, 77.4, 57.0, 49.4, 43.7, 42.8, 38.8, 34.0, 16.5, 8.4; IR (neat): v 3862, 3659, 1704, 1658, 1554, 1461, 1359, 670 cm^{-1}; HRMS (ESI): m/z [M + H]$^+$ calcd. for $C_{19}H_{23}N_2O$: 295.1805; found: 295.1803; $[\alpha]_D^{23}$ = +255.7 (c = 0.50, DCM).**; ent-62**: $[\alpha]_D^{23}$ = −255.0 (c = 0.50, DCM);

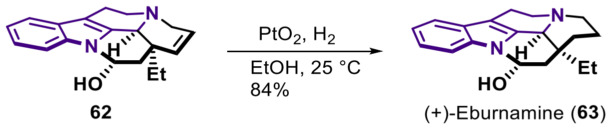

(+)-Eburnamine (63)

A mixture of **62** (35 mg, 0.12 mmol, 1.0 equiv) and PtO_2 (6 mg, 0.024 mmol, 0.2 equiv) in EtOH (1.2 mL) was stirred at 25 °C under an atmosphere of H_2 for 3 h. The mixture was filtered through a pad of celite and the filter cake was washed with MeOH. The filtrate was concentrated *in vacuo*. The residue was purified by column chromatography on silica gel (DCM: MeOH, 10:1) to give (+)-eburnamine (**63)**.

White solid, isolated yield 84% (30 mg); m.p.: 98.4-98.9 °C ; ^{1}H NMR (400 MHz, $CDCl_3$): δ 7.73 (d, J = 7.1 Hz, 1H), 7.48 (d, J = 6.8 Hz, 1H), 7.20-7.13 (m, 2H), 5.54 (dd, J = 9.5, 5.1 Hz, 1H), 3.72 (s, 1H), 3.27-3.12 (m, 2H), 2.96-2.87 (m, 1H), 2.51-2.46 (m, 2H), 2.33-2.23 (m, 2H), 2.07-1.97 (m, 1H), 1.72-1.61 (m, 1H), 1.51-1.24 (m, 4H), 0.90 (t, J = 7.5 Hz, 3H), 0.85-0.77 (m, 1H); ^{13}C NMR (101 MHz, $CDCl_3$): δ 136.6, 132.7, 128.6, 121.3, 120.1, 118.0, 112.2, 105.5, 76.5, 58.7, 50.7, 44.3, 43.4, 36.8, 28.5,

25.0, 20.4, 16.8, 7.5; IR (neat): v 3841, 3650, 1754, 1645, 1575, 1455, 1359, 1256, 679 cm^{-1}; HRMS (ESI): m/z $[M + H]^+$ calcd. for $C_{19}H_{25}N_2O$: 297.1961; found: 297.1957; $[\alpha]_D^{23}$ = +90.5 (c = 0.40, $CHCl_3$), for (–)-enantiomer: lit[6]. $[\alpha]_D^{20}$ = −93.3 (c = 0.47, $CHCl_3$); **ent-63**: $[\alpha]_D^{23}$ = −93.0 (c = 0.40, $CHCl_3$).

Supplementary Table 29 ^{1}H NMR data comparison of the synthetic (+)-eburnamine

**Lit[6]. Synthetic**	**This work**	**Δδ** (Lit – Our synthetic)
7.72 (d, J = 7.6 Hz, 1H)	7.73 (d, J = 7.1 Hz, 1H)	−0.01
7.47 (d, J = 6.8 Hz, 1H)	7.48 (d, J = 6.8 Hz, 1H)	−0.01
7.21-7.11 (m, 2H)	7.20-7.13 (m, 2H)	-
5.51 (dd, J = 9.2, 4.8 Hz, 1H)	5.54 (dd, J = 9.5, 5.1 Hz, 1H)	−0.03
3.63 (s, 1H)	3.72 (s, 1H)	−0.09
3.21 (dd, J = 13.6, 6.4 Hz, 1H)	3.27-3.12 (m, 2H)	-
3.16-3.08 (m, 1H)		
2.95-2.87 (m, 1H)	2.96-2.87 (m, 1H)	-
2.52-2.40 (m, 2H)	2.51-2.46 (m, 2H)	-
2.31-2.21 (m, 2H)	2.33-2.23 (m, 2H)	-
2.00 (dq, J = 15.0, 7.6 Hz, 1H)	2.07-1.97 (m, 1H)	-
1.69-1.60 (m, 1H)	1.72-1.61 (m, 1H)	-
1.56-1.24 (m, 4H)	1.51-1.24 (m, 4H)	-
0.86 (t, J = 7.6 Hz, 3H)	0.90 (t, J = 7.5 Hz, 3H)	−0.04
0.83-0.74 (m, 1H)	0.85-0.77 (m, 1H)	-

Supplementary Table 30 ^{13}C NMR data comparison of the synthetic (+)-eburnamine

**Lit[6]. Synthetic**	**This work**	**Δδ** (Lit – Our synthetic)
136.6	136.6	0
132.7	132.7	0
128.6	128.6	0
121.3	121.3	0
120.1	120.1	0
118.0	118.0	0
112.1	112.2	−0.1
105.7	105.5	0.2
76.6	76.5	0.1
58.7	58.7	0
50.8	50.7	0.1
44.3	44.3	0
43.6	43.4	0.2
36.8	36.8	0
28.6	28.5	0.1
25.1	25.0	0.1
20.5	20.4	0.1

16.8	16.8	0
7.6	7.5	0.1

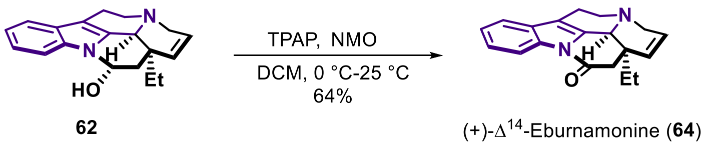

(+)-Δ[14]-Eburnamonine (64)

To a solution of **62** (40 mg, 0.14 mmol, 1.0 equiv), NMO (25 mg, 0.21 mmol, 1.5 equiv) and 4 Å molecular sieves (40 mg) in DCM (2.8 mL) was added TPAP (4.9 mg, 0.014 mmol, 0.1 equiv) at 25 ℃ and the reaction was stirred for 2 h. The mixture was filtered through a pad of celite and the filter cake was washed with DCM. The filtrate was concentrated *in vacuo*. The residue was purified by column chromatography on silica gel (DCM: MeOH, 20:1) to give (+)-Δ[14]-eburnamonine (**64**).

Yellow oil, isolated yield 64% (26 mg); ^{1}H NMR (400 MHz, $CDCl_3$): δ 8.33 (d, J = 7.2 Hz, 1H), 7.43 (d, J = 6.7 Hz, 1H), 7.32-7.25 (m, 2H), 5.56-5.52 (m, 1H), 5.49 (d, J = 10.4 Hz, 1H), 4.04 (s, 1H), 3.46 (dd, J = 14.0, 7.0 Hz, 1H), 3.35-3.27 (m, 1H), 3.17-3.11 (m, 1H), 3.07-2.99 (m, 1H), 2.93 (d, J = 17.7 Hz, 1H), 2.73 (s, 2H), 2.52 (dd, J = 17.0, 6.5 Hz, 1H), 1.94-1.85 (m, 1H), 1.72-1.63 (m, 1H), 1.02 (t, J = 7.6 Hz, 3H); ^{13}C NMR (101 MHz, $CDCl_3$): δ 167.0, 134.1, 132.8, 130.1, 127.0 (2C), 124.2, 123.7, 118.0, 116.1, 112.3, 56.3, 49.3, 43.9, 43.2, 40.8, 34.0, 16.3, 8.5; IR (neat): ν 3865, 3451, 2926, 1739, 1690, 1442, 1209, 805 cm^{-1}; HRMS (ESI): m/z $[M + H]^+$ calcd. for $C_{19}H_{21}N_2O$: 293.1648; found: 293.1645; $[\alpha]_D^{23}$ = +24.5 (c = 0.45, $CHCl_3$), lit[16]. $[\alpha]_D$ = +27.5 (c = 1.0, $CHCl_3$).

Supplementary Table 31 ^{1}H NMR data comparison of the synthetic (+)-Δ[14]-eburnamonine

**Lit[16]. Synthetic**	**This work**	**Δδ** (Lit – Our synthetic)
8.33 (m, 1H)	8.33 (d, J = 7.2 Hz, 1H)	-
7.41 (m, 1H)	7.43 (d, J = 6.7 Hz, 1H)	-
7.32-7.24 (m, 2H)	7.32-7.25 (m, 2H)	-
5.54 (dt, 1H)	5.56-5.52 (m, 1H)	-
4.98 (d, 1H)	5.49 (d, J = 10.4 Hz, 1H)	−0.51
4.04 (s, 1H)	4.04 (s, 1H)	0
3.43 (dd, 1H)	3.46 (dd, J = 14.0, 7.0 Hz, 1H)	−0.03
3.30 (ddd, 1H)	3.35-3.27 (m, 1H)	-
3.13 (dm, 1H)	3.17-3.11 (m, 1H)	-
3.04 (m ,1H)	3.07-2.99 (m, 1H)	-
2.91 (dm, 1H)	2.93 (d, J = 17.7 Hz, 1H)	−0.02
2.78 (d, 1H)	2.73 (s, 2H)	-
2.72 (d, 1H)		-
2.47 (ddd, 1H)	2.52 (dd, J = 17.0, 6.5 Hz, 1H)	−0.05
1.89 (dq, 1H)	1.94-1.85 (m, 1H)	-

1.68 (dq, 1H)	1.72-1.63 (m, 1H)	-
1.00 (t, 3H)	1.02 (t, J = 7.6 Hz, 3H)	−0.02

Supplementary Table 32 ^{13}C NMR data comparison of the synthetic (+)-Δ^{14}-eburnamonine

**Lit[16]. Synthetic**	**This work**	**Δδ** (Lit – Our synthetic)
167.0	167.0	0
134.2	134.1	0.1
132.9	132.8	0.1
130.1	130.1	0
127.0 (2C)	127.0 (2C)	0
124.2	124.2	0
123.7	123.7	0
118.0	118.0	0
116.1	116.1	0
112.3	112.3	0
56.4	56.3	0.1
49.4	49.3	0.1
43.9	43.9	0
43.3	43.2	0.1
40.9	40.8	0.1
34.1	34.0	0.1
16.3	16.3	0
8.5	8.5	0

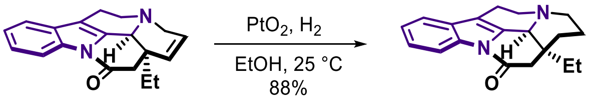

64

(–)-Eburnamonine (**65**)

(−)-Eburnamonine (65)

A mixture of **64** (23 mg, 0.08 mmol, 1.0 equiv) and PtO_2 (3.6 mg, 0.016 mmol, 0.2 equiv) in EtOH (0.8 mL) was stirred at 25 °C under an atmosphere of H_2 for 3 h. The mixture was filtered through a pad of celite and the filter cake was washed with MeOH. The filtrate was concentrated *in vacuo*. The residue was purified by column chromatography on silica gel (petroleum ether: EtOAc, 1:2) to give (−)-eburnamonine (**65**).

White solid, isolated yield 88% (20 mg); m.p.: 199.4-199.9 °C; ^{1}H NMR (400 MHz, $CDCl_3$): δ 8.38 (d, J = 7.2 Hz, 1H), 7.44 (d, J = 6.8 Hz, 1H), 7.33-7.26 (m, 2H), 3.97 (s, 1H), 3.36-3.21 (m, 2H), 2.95-2.86 (m, 1H), 2.68-2.56 (m, 3H), 2.51-2.38 (m, 2H), 2.09-2.00 (m, 1H), 1.81-1.61 (m, 2H), 1.51 (d, J = 13.6 Hz, 1H), 1.41-1.36 (m, 1H), 1.07-1.00 (m, 1H), 0.95 (t, J = 7.6 Hz, 3H); ^{13}C NMR (101 MHz, $CDCl_3$): δ 167.6, 134.2, 132.0, 130.1, 124.3, 123.8, 118.1, 116.2, 112.6, 57.7, 50.6, 44.4, 44.3, 38.4, 28.3, 26.9,

20.6, 16.5, 7.6; IR (neat): *v* 3434, 2927, 1707, 1632, 1455, 1379, 811 cm^{-1}; HRMS (ESI): m/z $[M + H]^+$ calcd. for $C_{19}H_{23}N_2O$: 295.1805; found: 295.1801; $[\alpha]_D^{23} = -80.7$ (c = 0.40, $CHCl_3$), for (+)-enantiomer: lit[6]. $[\alpha]_D^{20} = +83.1$ (c = 0.23, $CHCl_3$); **ent-65**: $[\alpha]_D^{23} = +80.0$ (c = 0.40, DCM).

Supplementary Table 33 ^{1}H NMR data comparison of the synthetic (+)-eburnamine

<table>
<tr><th>Lit[6]. Synthetic</th><th>This work</th><th>Δδ (Lit – Our synthetic)</th></tr>
<tr><td>8.37 (d, J = 7.6 Hz, 1H)</td><td>8.38 (d, J = 7.2 Hz, 1H)</td><td>−0.01</td></tr>
<tr><td>7.43 (d, J = 7.2 Hz, 1H)</td><td>7.44 (d, J = 6.8 Hz, 1H)</td><td>−0.01</td></tr>
<tr><td>7.36-7.20 (m, 2H)</td><td>7.33-7.26 (m, 2H)</td><td>-</td></tr>
<tr><td>3.99 (s, 1H)</td><td>3.97 (s, 1H)</td><td>0.02</td></tr>
<tr><td>3.34 (dd, J = 14.0, 6.4 Hz, 1H)</td><td rowspan="2">3.36-3.21 (m, 2H)</td><td>-</td></tr>
<tr><td>3.31-3.21 (m, 1H)</td><td>-</td></tr>
<tr><td>2.91 (dt, J = 16.8, 8.0 Hz, 1H)</td><td rowspan="3">2.95-2.86 (m, 1H)
2.68-2.56 (m, 3H)
2.51-2.38 (m, 2H)</td><td>-</td></tr>
<tr><td>2.68 and 2.59 (ABq, J 1= J 2 = 16.8 Hz, 2H)</td><td>-</td></tr>
<tr><td>2.61-2.35 (m, 3H)</td><td>-</td></tr>
<tr><td>2.08-2.00 (m, 1H)</td><td>2.09-2.00 (m, 1H)</td><td>-</td></tr>
<tr><td>1.78-1.61 (m, 2H)</td><td>1.81-1.61 (m, 2H)</td><td>-</td></tr>
<tr><td>1.49 (d, J = 13.6 Hz, 1H)</td><td>1.51 (d, J = 13.6 Hz, 1H)</td><td>−0.02</td></tr>
<tr><td>1.39 (d, J = 13.6 Hz, 1H)</td><td>1.41-1.36 (m, 1H)</td><td>-</td></tr>
<tr><td>1.04 (td, J = 13.6, 3.2 Hz, 1H)</td><td>1.07-1.00 (m, 1H)</td><td>-</td></tr>
<tr><td>0.93 (t, J = 7.6 Hz, 3H)</td><td>0.95 (t, J = 7.6 Hz, 3H)</td><td>−0.02</td></tr>
</table>

Supplementary Table 34 ^{13}C NMR data comparison of the synthetic (+)-eburnamine

Lit[6]. Synthetic	This work	Δδ (Lit – Our synthetic)
167.7	167.6	0.1
134.2	134.2	0
132.0	132.0	0
130.1	130.1	0
124.3	124.3	0
123.8	123.8	0
118.1	118.1	0
116.2	116.2	0
112.6	112.6	0
57.7	57.7	0
50.7	50.6	0.1
44.4	44.4	0
44.3	44.3	0
38.4	38.4	0
28.4	28.3	0.1

27.0	26.9	0.1
20.6	20.6	0
16.5	16.5	0
7.7	7.6	0.1

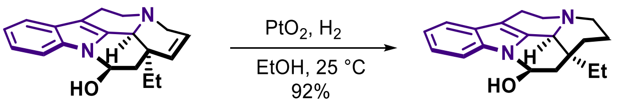

61

(–)-Isoeburnamine (**66**)

(−)-Isoeburnamine (66)

A mixture of **61** (27 mg, 0.092 mmol, 1.0 equiv) and PtO_2 (4 mg, 0.018 mmol, 0.2 equiv) in EtOH (0.92 mL) was stirred at 25 °C under an atmosphere of H_2 for 3 h. The mixture was filtered through a pad of celite and the filter cake was washed with MeOH. The filtrate was concentrated *in vacuo*. The residue was purified by column chromatography on silica gel (DCM: MeOH, 10:1) to give (−)-isoeburnamine (**66**).

White solid, isolated yield 92% (25 mg); m.p.: 174.9-175.3 °C ; ^{1}H NMR (400 MHz, $CDCl_3$): δ 7.52 (d, J = 7.2 Hz, 1H), 7.42 (d, J = 7.6 Hz, 1H), 7.22-7.13 (m, 2H), 6.07 (d, J = 4.1 Hz, 1H), 3.85 (s, 1H), 3.37 (dd, J = 13.6, 6.2 Hz, 1H), 3.29-3.21 (m, 1H), 3.04-2.95 (m, 1H), 2.67-2.52 (m, 3H), 2.23-2.14 (m, 2H), 2.03 (dd, J = 15.0, 4.7 Hz, 1H), 1.79-1.74 (m, 1H), 1.68-1.60 (m, 1H), 1.56-1.53 (m, 1H), 1.49-1.37 (m, 2H), 0.95 (t, J = 7.6 Hz, 3H); ^{13}C NMR (101 MHz, $CDCl_3$): δ 134.7, 131.2, 128.9, 121.2, 120.1, 118.5, 109.8, 105.6, 74.7, 59.3, 51.3, 44.9, 39.9, 34.6, 29.0, 26.6, 21.0, 16.8, 7.6; IR (neat): ν 3854, 3645, 1774, 1625, 1545, 1359, 1259, 670 cm^{-1}; HRMS (ESI): m/z [M + H]$^+$ calcd. for $C_{19}H_{25}N_2O$: 297.1961; found: 297.1957; $[\alpha]_D^{23}$ = −110.2 (c = 0.25, $CHCl_3$), for (+)-enantiomer: lit[6]. $[\alpha]_D^{20}$ = +109.2 (c = 0.15, $CHCl_3$); **ent-66**: $[\alpha]_D^{23}$ = +110.0 (c = 0.20, $CHCl_3$).

Supplementary Table 35 ^{1}H NMR data comparison of the synthetic (−)-isoeburnamine

**Lit[6]. Synthetic**	**This work**	**Δδ** (Lit – Our synthetic)
7.51 (d, J = 7.6 Hz, 1H)	7.52 (d, J = 7.2 Hz, 1H)	−0.01
7.41 (d, J = 8.0 Hz, 1H)	7.42 (d, J = 7.6 Hz, 1H)	−0.01
7.24-7.10 (m, 2H)	7.22-7.13 (m, 2H)	-
6.06 (d, J = 3.6 Hz, 1H)	6.07 (d, J = 4.1 Hz, 1H)	−0.01
3.87 (s, 1H)	3.85 (s, 1H)	0.02
3.35 (dd, J = 13.6, 6.0 Hz, 1H)	3.37 (dd, J = 13.6, 6.2 Hz, 1H)	−0.02
3.30-3.22 (m, 1H)	3.29-3.21 (m, 1H)	-
3.04-2.95 (m, 1H)	3.04-2.95 (m, 1H)	-
2.74-2.49 (m, 3H)	2.67-2.52 (m, 3H)	-
2.24-2.15 (m, 2H)	2.23-2.14 (m, 2H)	-
2.00 (dd, J = 14.8, 4.8 Hz, 1H)	2.03 (dd, J = 15.0, 4.7 Hz, 1H)	−0.03
1.85-1.70 (m, 1H)	1.79-1.74 (m, 1H)	-
1.70-1.60 (m, 2H)	1.68-1.60 (m, 1H)	-
	1.56-1.53 (m, 1H)	
1.59-1.35 (m, 2H)	1.49-1.37 (m, 2H)	-

0.93 (t, J = 7.6 Hz, 3H)	0.95 (t, *J* = 7.6 Hz, 3H)	−0.02

**Supplementary Table 36 ^{13}C NMR data comparison of the synthetic (−)-isoeburnamine**		
**Lit[6]. Synthetic**	**Lit[6]. Synthetic**	**Δδ** (Lit – Our synthetic)
134.7	134.7	0
131.0	131.2	−0.2
128.9	128.9	0
121.3	121.2	0.1
120.2	120.1	0.1
118.5	118.5	0
109.8	109.8	0
105.5	105.6	−0.1
74.7	74.7	0
59.3	59.3	0
51.3	51.3	0
44.9	44.9	0
39.9	39.9	0
34.7	34.6	0.1
29.0	29.0	0
26.5	26.6	−0.1
20.9	21.0	−0.1
16.7	16.8	−0.1
7.6	7.6	0

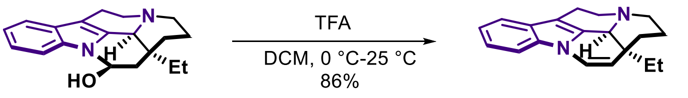

66 (−)-Eburnamenine (**67**)

(−)-Eburnamenine (67)

To a solution of **66** (20 mg, 0.07 mmol, 1.0 equiv) in DCM (2.3 mL) was added TFA (0.15 mL) at 0 °C. The reaction mixture was stirred at 25 °C for 4 h. The reaction mixture was quenched with saturated aqueous $NaHCO_3$. The aqueous layer was extracted with EtOAc. The combined organic layers were washed with brine, dried over Na_2SO_4 and concentrated in *vacuo*. The residue was purified by column chromatography on silica gel (petroleum ether: EtOAc, 2:1) to give (−)-eburnamenine (**67**).

White solid, isolated yield 86% (17 mg); m.p.: 44.7-45.0 °C ; ^{1}H NMR (400 MHz, $CDCl_3$): δ 7.49 (d, *J* = 7.7 Hz, 1H), 7.35 (d, *J* = 8.1 Hz, 1H), 7.21(td, *J* = 7.2, 1.0 Hz, 1H), 7.13 (td, *J* = 7.8, 1.0 Hz, 1H), 6.94 (d, *J* = 7.9 Hz, 1H), 5.09 (d, *J* = 7.9 Hz, 1H), 4.28 (s, 1H), 3.40 (dd, *J* = 13.7, 5.9 Hz, 1H), 3.32-3.24 (m, 1H), 3.08-2.99 (m, 1H), 2.78-2.67 (m, 2H), 2.55-2.49 (m, 1H), 2.04-1.95 (m, 1H), 1.76-1.66 (m, 2H), 1.50-1.41 (m, 2H), 1.19 (td, *J* = 13.8, 3.7 Hz, 1H), 1.02 (t, *J* = 7.5 Hz, 3H); ^{13}C NMR (101 MHz, $CDCl_3$):

δ 133.5, 130.2, 128.2, 121.5, 119.8, 119.7, 118.4, 116.7, 108.5, 107.0, 55.7, 52.1, 45.4, 37.3, 31.1, 27.5, 20.8, 16.5, 9.0; IR (neat): ν 3865, 3660, 3446, 1705, 1658, 1462, 1264, 739 cm^{-1}; HRMS (ESI): m/z [M + H]$^+$ calcd. for $C_{19}H_{23}N_2$: 279.1856; found: 279.1852; $[\alpha]_D^{23}$ = −167.3 (*c* = 0.21, $CHCl_3$), for (+)-enantiomer: lit[6]. $[\alpha]_D^{20}$ = +177.6 (*c* = 0.42, $CHCl_3$); **ent-67**: $[\alpha]_D^{23}$ = +168.1 (*c* = 0.20, $CHCl_3$).

Supplementary Table 37 ^{1}H NMR data comparison of the synthetic (−)-eburnamenine

Lit[6]. Synthetic	This work	Δδ (Lit – Our synthetic)
7.47 (d, *J* = 7.6 Hz, 1H)	7.49 (d, *J* = 7.7 Hz, 1H)	−0.02
7.34 (d, *J* = 8.0 Hz, 1H)	7.35 (d, *J* = 8.1 Hz, 1H)	−0.01
7.17 (d, *J* = 7.2 Hz, 1H)	7.21(td, *J* = 7.2, 1.0 Hz, 1H)	−0.04
7.11 (t, *J* = 7.2 Hz, 1H)	7.13 (td, *J* = 7.8, 1.0 Hz, 1H)	−0.02
6.93 (d, *J* = 8.0 Hz, 1H)	6.94 (d, *J* = 7.9 Hz, 1H)	−0.01
5.08 (d, *J* = 8.0 Hz, 1H)	5.09 (d, *J* = 7.9 Hz, 1H)	−0.01
4.27 (s, 1H)	4.28 (s, 1H)	−0.01
3.37 (dd, *J* = 13.6, 6.0 Hz, 1H)	3.40 (dd, *J* = 13.7, 5.9 Hz, 1H)	−0.03
3.33-3.22 (m ,1H)	3.32-3.24 (m, 1H)	-
3.11-2.97 (m ,1H)	3.08-2.99 (m, 1H)	-
2.80-2.64 (m ,2H)	2.78-2.67 (m, 2H)	-
2.52 (dd, *J* = 15.6, 4.4 Hz, 1H)	2.55-2.49 (m, 1H)	-
2.05-1.93 (m ,1H)	2.04-1.95 (m, 1H)	-
1.77-1.63 (m ,2H)	1.76-1.66 (m, 2H)	-
1.49-1.37 (m ,2H)	1.50-1.41 (m, 2H)	-
1.16 (td, *J* = 13.6, 3.6 Hz, 1H)	1.19 (td, *J* = 13.8, 3.7 Hz, 1H)	−0.03
1.00 (t, *J* = 7.6 Hz, 3H)	1.02 (t, *J* = 7.5 Hz, 3H)	−0.02

Supplementary Table 38 ^{13}C NMR data comparison of the synthetic (−)-eburnamenine

Lit[6]. Synthetic	This work	Δδ (Lit – Our synthetic)
133.5	133.5	0
130.3	130.2	0.1
128.2	128.2	0
121.5	121.5	0
119.8	119.8	0
119.7	119.7	0
118.4	118.4	0
116.7	116.7	0
108.5	108.5	0
107.0	107.0	0
55.8	55.7	0.1
52.1	52.1	0
45.4	45.5	−0.1
37.3	37.3	0
31.1	31.1	0
27.6	27.5	0.1

20.8	20.8	0
16.5	16.5	0
9.0	9.0	0

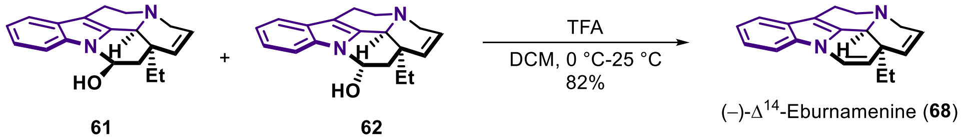

(−)-Δ^{14}-Eburnamenine (68)

To a solution of **61** and **62** (50 mg, 0.17 mmol, 1.0 equiv) in DCM (5.6 mL) was added TFA (0.37 mL) at 0 °C. The reaction mixture was stirred at 25 °C for 4 h. The reaction mixture was quenched with saturated aqueous $NaHCO_3$. The aqueous layer was extracted with EtOAc. The combined organic layers were washed with brine, dried over Na_2SO_4 and concentrated in *vacuo*. The residue was purified by column chromatography on silica gel (petroleum ether: EtOAc, 5:1) to give (−)-Δ^{14}-eburnamenine (**68**).

Yellow solid, isolated yield 82% (39 mg); m.p.: 70.0-70.4 °C ; ^{1}H NMR (400 MHz, $CDCl_3$): δ 7.48 (d, J = 7.7 Hz, 1H), 7.34 (d, J = 8.1 Hz, 1H), 7.21-7.17 (m, 1H), 7.13-7.09 (m, 1H), 6.97 (d, J = 8.0 Hz, 1H), 5.57-5.51 (m, 1H), 5.38-5.35 (m, 1H), 4.99 (d, J = 8.0 Hz, 1H), 4.38 (s, 1H), 3.52 (dd, J = 13.8, 6.4 Hz, 1H), 3.41-3.34 (m, 1H), 3.26-3.17 (m, 2H), 3.13-3.07 (m, 1H), 2.59 (ddd, J = 16.4, 5.6, 2.0 Hz, 1H), 1.83-1.72 (m, 2H), 1.07 (t, J = 7.5 Hz, 3H); ^{13}C NMR (101 MHz, $CDCl_3$): δ 133.7, 130.0, 128.3, 127.7, 122.5, 121.6, 119.9 (2C), 118.4, 112.9, 108.6, 107.2, 54.3, 50.3, 43.4, 38.8, 33.4, 16.6, 9.2; IR (neat): v 3864, 1739, 1658, 1563, 1544, 1461, 670 cm^{-1}; HRMS (ESI): m/z [M + H]$^+$ calcd. for $C_{19}H_{21}N_2$: 277.1699; found: 277.1697; $[\alpha]_D^{23}$ = −39.0 (c = 0.20, $CHCl_3$).

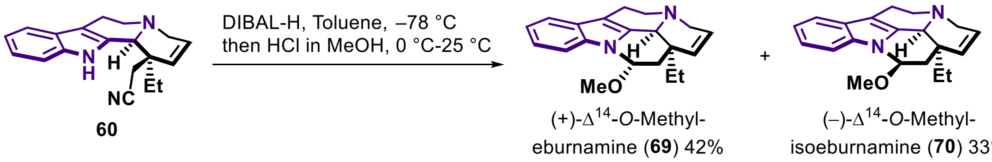

(+)-Δ^{14}-*O*-methyleburnamine (69) and **(−)-Δ^{14}-*O*-methylisoeburnamine (70)**

Under N_2, to a solution of **60** (50 mg, 0.17 mmol, 1.0 equiv) in dry toluene (1.7 mL) at −78°C was added dropwise DIBAL-H (0.37 mL, 1.0 M in Hexane, 0.37 mmol, 2.2 equiv) and the mixture was stirred for 30 min at −78 °C. The reaction was quenched with Rochelle salts and the mixture was stirred for 1 h. The aqueous layer was extracted with EtOAc. The combined organic layers were washed with brine, dried over Na_2SO_4 and concentrated in *vacuo*. The above residue was dissolved in dry THF (0.6 mL), and 1 M HCl in MeOH (under N_2, the solution of 2.4 mL MeOH was added the 0.17 mL AcCl at 0 °C and stirred for 5 min at 25 °C) was added. The reaction was stirred at 25 °C for 2 h then was quenched with saturated aqueous $NaHCO_3$. The aqueous layer was extracted with EtOAc. The combined organic layers were washed with brine, dried over Na_2SO_4 and concentrated in *vacuo*. The residue was purified by column chromatography on silica gel (DCM: MeOH, 20:1) to give **69** and **70**.

69: Yellow solid, isolated yield 42% (22 mg); m.p.: 80.1-80.7 °C ; ^{1}H NMR (400 MHz, $CDCl_3$): δ 7.62

(d, J = 7.8 Hz, 1H), 7.50 (d, J = 6.8 Hz, 1H), 7.20-7.12 (m, 2H), 5.48 (s, 2H), 5.25 (dd, J = 10.0, 4.4 Hz, 1H), 4.03 (s, 1H), 3.46-3.41 (m, 4H), 3.36-3.28 (m, 1H), 3.19-3.10 (m, 1H), 3.08 (d, J = 17.0 Hz, 1H), 2.87 (d, J = 17.4 Hz, 1H), 2.60 (ddd, J = 16.2, 6.4, 1.9 Hz, 1H), 2.22 (dd, J = 13.5, 4.5 Hz, 1H), 2.05 (dd, J = 13.4, 10.0 Hz, 1H), 1.96-1.87 (m, 1H), 1.73-1.64 (m, 1H), 1.05 (t, J = 7.6 Hz, 3H); ^{13}C NMR (101 MHz, $CDCl_3$): δ 136.8, 133.6, 128.8, 127.0, 126.7, 121.3, 119.9, 117.8, 112.2, 105.9, 83.3, 57.2, 50.9, 49.5, 43.8, 38.6, 36.3, 34.4, 16.6, 8.5; IR (neat): v 3864, 3755, 1734, 1658, 1563, 1544, 1461, 670 cm^{-1}; HRMS (ESI): m/z [M + H]$^+$ calcd. for $C_{20}H_{25}N_2O$: 309.1961; found: 309.1958; $[\alpha]_D^{23}$ = +227.9 (c = 0.11, $CHCl_3$); **ent-69**: $[\alpha]_D^{23}$ = −228.5 (c = 0.15, $CHCl_3$).

70: White solid, isolated yield 33% (17.5 mg); m.p.: 57.6-58.2 °C ; ^{1}H NMR (400 MHz, $CDCl_3$): δ 7.45 (d, J = 7.5 Hz, 1H), 7.27-7.26 (m, 1H), 7.17-7.08 (m, 2H), 5.63 (d, J = 10.3 Hz, 1H), 5.51-5.45 (m, 2H), 4.01 (s, 1H), 3.48-3.42 (m, 4H), 3.37-3.29 (m, 1H), 3.16-3.08 (m, 3H), 2.59 (ddd, J = 16.1, 6.3, 1.8 Hz, 1H), 2.43 (dd, J = 14.7, 1.8 Hz, 1H), 1.95-1.88 (m, 2H), 1.67-1.60 (m, 1H), 1.04 (t, J = 7.6 Hz, 3H); ^{13}C NMR (101 MHz, $CDCl_3$): δ 135.5, 131.6, 129.3, 128.8, 123.1, 121.0, 120.0, 118.1, 110.4, 105.5, 82.4, 57.4, 56.0, 49.9, 43.8, 36.1, 34.9, 34.8, 16.5, 8.5; IR (neat): v 3864, 1704, 1649, 1554, 1512, 1469, 1359, 670 cm^{-1}; HRMS (ESI): m/z [M + H]$^+$ calcd. for $C_{20}H_{25}N_2O$: 309.1961; found: 309.1958; $[\alpha]_D^{23}$ = -89.6 (c = 0.60, $CHCl_3$); **ent-70**: $[\alpha]_D^{23}$ = +91.2 (c = 0.20, $CHCl_3$).

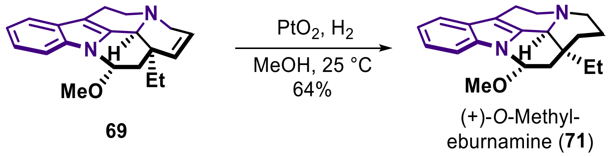

(+)-*O*-methyleburnamine (71)

A mixture of **69** (22 mg, 0.07 mmol, 1.0 equiv) and PtO_2 (3.2 mg, 0.014 mmol, 0.2 equiv) in MeOH (0.7 mL) was stirred at 25 °C under an atmosphere of H_2 for 3 h. The mixture was filtered through a pad of celite and the filter cake was washed with MeOH. The mixture was concentrated *in vacuo*. The residue was purified by column chromatography on silica gel (petroleum ether: EtOAc, 1:2) to give (+)-*O*-methyleburnamine (**71**).

White solid, isolated yield 64% (14 mg); m.p.: 70.4-70.8 °C ; ^{1}H NMR (400 MHz, $CDCl_3$): δ 7.60 (d, J = 7.3 Hz, 1H), 7.50 (d, J = 6.8 Hz, 1H), 7.21-7.13 (m, 2H), 5.56 (dd, J = 9.6, 5.4 Hz, 1H), 3.92 (s, 1H), 3.35-3.25 (m, 5H), 3.02-2.93 (m, 1H), 2.56-2.51 (m, 2H), 2.38-2.35 (m, 1H), 2.18-2.09 (m, 2H), 1.89 (dd, J = 13.8, 9.6 Hz, 1H), 1.79-1.67 (m, 1H), 1.65-1.55 (m, 1H), 1.41-1.38 (m, 1H), 1.33-1.29 (m, 1H), 0.96-0.86 (m, 4H); ^{13}C NMR (101 MHz, $CDCl_3$): δ 136.5, 133.1, 128.6, 121.3, 120.0, 117.9, 111.8, 105.8, 82.3, 58.8, 50.8, 50.5, 44.3, 36.5 (2C), 28.8, 25.2, 20.5, 16.8, 7.6; IR (neat): v 3864, 3659, 1739, 1658, 1563, 1544, 1511, 1461, 671 cm^{-1}; HRMS (ESI): m/z [M + H]$^+$ calcd. for $C_{20}H_{27}N_2O$: 311.2118; found: 311.2114; $[\alpha]_D^{23}$ = +75.3 (c = 0.10, $CHCl_3$), for (−)-enantiomer: lit[17]. $[\alpha]_D$ = −67.3 (c = 0.26, $CHCl_3$); **ent-71**: $[\alpha]_D^{23}$ = −75.0 (c = 0.10, $CHCl_3$).

Supplementary Table 39 ^{1}H NMR data comparison of the synthetic (+)-*O*-methyleburnamine

Lit[17]. Natural	This work	Δδ (Lit – Our synthetic)
7.62-7.43 (m, 2H)	7.60 (d, J = 7.3 Hz, 1H)	-
	7.50 (d, J = 6.8 Hz, 1H)	-

7.24-7.10 (m, 2H)	7.21-7.13 (m, 2H)	-
5.52 (dd, J = 9.2, 5.5 Hz, 1H)	5.56 (dd, J = 9.6, 5.4 Hz, 1H)	−0.04
3.90 (s, 1H)	3.92 (s, 1H)	−0.02
3.33 (s, 3H)<br>3.24-1.25 (m, 14H)<br>0.92 (t, J = 7.5 Hz, 3H)	3.35-3.25 (m, 5H)	-
	3.02-2.93 (m, 1H)	
	2.56-2.51 (m, 2H)	
	2.38-2.35 (m, 1H)	
	2.18-2.09 (m, 2H)	
	1.89 (dd, J = 13.8, 9.6 Hz, 1H)	
	1.79-1.67 (m, 1H)	
	1.65-1.55 (m, 1H)	
	1.41-1.38 (m, 1H)	
	1.33-1.29 (m, 1H)	
	0.96-0.86 (m, 4H)	

Supplementary Table 40 ^{13}C NMR data comparison of the synthetic (+)-*O*-methyleburnamine

**Lit[17]. Synthetic**	**This work**	**Δδ** (Lit – Our synthetic)
136.7	136.5	0.2
133.2	133.1	0.1
129.0	128.6	0.4
121.4	121.3	0.1
120.1	120.0	0.1
118.1	117.9	0.2
112.0	111.8	0.2
105.9	105.8	0.1
82.4	82.3	0.1
58.8	58.8	0
50.9	50.8	0.1
50.6	50.5	0.1
44.3	44.3	0
36.3 (2C)	36.5 (2C)	0
28.9	28.8	0.1
25.3	25.2	0.1
20.5	20.5	0
16.9	16.8	0.1
7.6	7.6	0

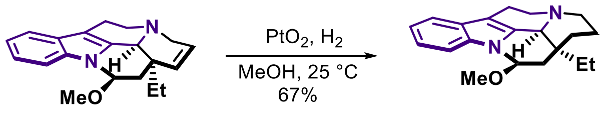

70

(–)-*O*-methyl-
isoeburnamine (**72**)

(−)-*O*-methylisoeburnamine (72)

A mixture of **70** (17.5 mg, 0.06 mmol, 1.0 equiv) and PtO_2 (2.7 mg, 0.012 mmol, 0.2 equiv) in MeOH (0.6 mL) was stirred at 25 °C under an atmosphere of H_2 for 3 h. The mixture was filtered through a pad of celite and the filter cake was washed with MeOH. The filtrate was concentrated *in vacuo*. The residue was purified by column chromatography on silica gel (petroleum ether: EtOAc, 1:2) to give (−)-*O*-methylisoeburnamine (**72**).

White solid, isolated yield 67% (12.5 mg); m.p.: 50.3-50.9 °C ; ^{1}H NMR (400 MHz, $CDCl_3$): δ 7.48 (d, J = 7.5 Hz, 1H), 7.29 (d, J = 8.0 Hz, 1H), 7.19-7.10 (m, 2H), 5.47 (dd, J = 4.0, 0.9 Hz, 1H), 3.86 (s, 1H), 3.51 (s, 3H), 3.36-3.21 (m, 2H), 3.03-2.94 (m, 1H), 2.61-2.51 (m, 3H), 2.28 (d, J = 14.9 Hz, 1H), 2.18-2.09 (m, 1H), 1.85-1.68 (m, 3H), 1.55-1.16 (m, 1H), 1.40-1.34 (m, 2H), 0.94 (t, J = 7.6 Hz, 3H); ^{13}C NMR (101 MHz, $CDCl_3$): δ 135.4, 131.1, 128.7, 121.1, 120.0, 118.2, 110.5, 105.4, 83.1, 59.2, 55.7, 51.3, 44.8, 35.0, 34.5, 29.0, 25.6, 20.9, 16.8, 7.7; IR (neat): v 3864, 1704, 1658, 1563, 1544, 1461, 670 cm^{-1}; HRMS (ESI): m/z $[M + H]^+$ calcd. for $C_{20}H_{27}N_2O$: 311.2118; found: 311.2114; $[\alpha]_D^{23}$ = −50.8 (c = 0.45, $CHCl_3$), for (+)-enantiomer: lit[17]. $[\alpha]_D$ = +72.7 (c = 0.22, $CHCl_3$); **ent-72**: $[\alpha]_D^{23}$ = +51.5 (c = 0.20, $CHCl_3$).

Supplementary Table 41 ^{1}H NMR data comparison of the synthetic (−)-*O*-methylisoeburnamine

**Lit[17]. Natural**	**This work**	**Δδ** (Lit – Our synthetic)
7.45-7.40 (m, 1H)	7.48 (d, J = 7.5 Hz, 1H)	-
7.25-7.07 (m, 3H)	7.29 (d, J = 8.0 Hz, 1H)	-
	7.19-7.10 (m, 2H)	-
5.45 (dd, J = 4.1, 1.1 Hz, 1H)	5.47 (dd, J = 4.0, 0.9 Hz, 1H)	−0.02
3.83 (s, 1H)	3.86 (s, 1H)	−0.03
3.50 (s, 3H)	3.51 (s, 3H)	−0.01
3.33-1.22 (m, 14H)	3.36-3.21 (m, 2H)	-
	3.03-2.94 (m, 1H)	
	2.61-2.51 (m, 3H)	
	2.28 (d, J = 14.9 Hz, 1H)	
	2.18-2.09 (m, 1H)	
	1.85-1.68 (m, 3H)	
	1.40-1.34 (m, 2H)	
0.92 (t, J = 7.5 Hz, 3H)	0.94 (t, J = 7.6 Hz, 3H)	−0.02

Supplementary Table 42 ^{13}C NMR data comparison of the synthetic (−)-*O*-methyleburnamine

**Lit[17]. Synthetic**	**This work**	**Δδ** (Lit – Our synthetic)
135.6	135.4	0.2
131.3	131.1	0.2
128.9	128.7	0.2
121.0	121.1	−0.1

119.9	120.0	−0.1
118.2	118.2	0
110.5	110.5	0
105.5	105.4	0.1
83.1	83.1	0
59.2	59.2	0
55.7	55.7	0
51.3	51.3	0
44.8	44.8	0
35.2	35.0	0.2
34.5	34.5	0
29.1	29.0	0.1
25.8	25.6	0.2
21.1	20.9	0.2
16.9	16.8	0.1
7.6	7.7	−0.1

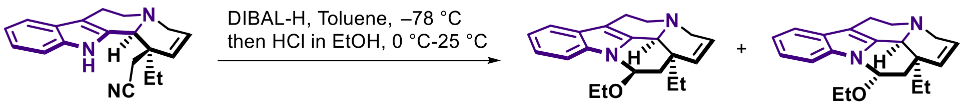

60 **73** 29% **74** 44%

(−)-Δ^{14}-*O*-ethylisoeburnamine (73) and **(+)-Δ^{14}-*O*-ethyleburnamine (74)**

Under N_2, to a solution of **60** (50 mg, 0.17 mmol, 1.0 equiv) in dry toluene (1.7 mL) at −78°C was added dropwise DIBAL-H (0.37 mL, 1.0 M in Hexane, 0.37 mmol, 2.2 equiv) and the mixture was stirred for 30 min at −78 °C. The reaction was quenched with Rochelle salts and the mixture was stirred for 1 h. The aqueous layer was extracted with EtOAc. The combined organic layers were washed with brine, dried over Na_2SO_4 and concentrated in *vacuo*. The above residue was dissolved in dry THF (1.2 mL), and 1 M HCl in EtOH (under N_2, the solution of 2.4 mL EtOH was added the 0.17 mL AcCl at 0 °C and stirred for 5 min at 25 °C) was added. The reaction was stirred at 25 °C for 2 h then was quenched with saturated aqueous $NaHCO_3$. The aqueous layer was extracted with EtOAc. The combined organic layers were washed with brine, dried over Na_2SO_4 and concentrated in *vacuo*. The residue was purified by column chromatography on silica gel (DCM: MeOH, 20:1) to give **73** and **74**.

73: Yellow solid, isolated yield 29% (16 mg); m.p.: 43.4-43.8 °C ; ^{1}H NMR (400 MHz, $CDCl_3$): δ 7.45 (d, J = 7.2 Hz, 1H), 7.27-7.25(m, 1H), 7.15-7.07 (m, 2H), 5.64-5.60 (m, 1H), 5.57 (dd, J = 3.5, 1.8 Hz, 1H), 5.48-5.44 (m, 1H), 4.01 (s, 1H), 3.73-3.61 (m, 2H), 3.47-3.42 (m, 1H), 3.37-3.30 (m, 1H), 3.16-3.07 (m, 3H), 2.58-2.53 (m, 1H), 2.42 (dd, J = 14.7, 1.8 Hz, 1H), 1.97-1.86 (m, 2H), 1.66-1.57 (m, 1H), 1.23 (t, J = 7.0 Hz, 3H), 1.03 (d, J = 7.6 Hz, 3H); ^{13}C NMR (101 MHz, $CDCl_3$): δ 135.5, 131.9, 129.5, 128.9, 123.1, 121.0, 119.9, 118.1, 110.4, 105.4, 80.8, 64.0, 57.5, 50.0, 44.0, 36.1, 36.0, 34.8, 16.6, 15.3, 8.5; IR (neat): v 3865, 2929, 1734, 1544, 1358, 1091, 801, 670 cm^{-1}; HRMS (ESI): m/z [M + H]$^+$ calcd. for $C_{21}H_{27}N_2O$: 323.2118; found: 323.2114; $[\alpha]_D^{23}$ = −118.3 (c = 0.20, $CHCl_3$); **ent-73**: $[\alpha]_D^{23}$ = +120.1 (c = 0.20, $CHCl_3$).

74: Yellow solid, isolated yield 44% (24 mg); m.p.: 122.6-123.5 °C ; ^{1}H NMR (400 MHz, $CDCl_3$): *δ* 7.63 (d, *J* = 7.2 Hz, 1H), 7.48 (d, *J* = 6.9 Hz, 1H), 7.18-7.11 (m, 2H), 5.47 (s, 2H), 5.27 (dd, *J* = 9.9, 4.4 Hz, 1H), 4.02 (s, 1H), 3.74-3.59 (m, 2H), 3.45 (dd, *J* = 13.9, 7.2 Hz, 1H), 3.35-3.27 (m, 1H), 3.18-3.02 (m, 2H), 2.87 (d, *J* = 17.3 Hz, 1H), 2.59 (ddd, *J* = 16.1, 6.4, 1.7 Hz, 1H), 2.25 (dd, *J* = 13.4, 4.4 Hz, 1H), 2.06-2.00 (m, 1H), 1.94-1.85 (m, 1H), 1.72-1.63 (m, 1H), 1.35 (t, *J* = 7.0 Hz, 3H), 1.04 (t, *J* = 7.6 Hz, 3H); ^{13}C NMR (101 MHz, $CDCl_3$): *δ* 136.9, 133.6, 128.9, 127.0, 126.7, 121.1, 119.8, 117.8, 112.2, 105.8, 82.8, 59.4, 57.3, 49.6, 43.9, 38.6, 37.4, 34.4, 16.6, 15.5, 8.5; IR (neat): *v* 3864, 2931, 1658, 1563, 1461, 1095, 746, 670 cm^{-1}; HRMS (ESI): m/z [M + H]$^+$ calcd. for $C_{21}H_{27}N_2O$: 323.2118; found: 323.2114; $[\alpha]_D^{23}$ = +268.0 (*c* = 0.20, $CHCl_3$); **ent-74**: $[\alpha]_D^{23}$ = −266.1 (*c* = 0.20, $CHCl_3$).

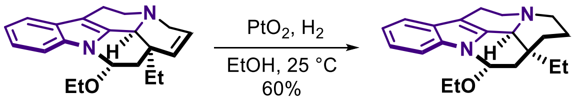

74

(+)-*O*-ethyl-
eburnamine (**75**)

(+)-*O*-ethyleburnamine (75)

A mixture of **74** (24 mg, 0.07 mmol, 1.0 equiv) and PtO_2 (3.2 mg, 0.014 mmol, 0.2 equiv) in EtOH (0.7 mL) was stirred at 25 °C under an atmosphere of H_2 for 3 h. The mixture was filtered through a pad of celite and the filter cake was washed with MeOH. The filtrate was concentrated *in vacuo*. The residue was purified by column chromatography on silica gel (DCM: MeOH, 20:1) to give (+)-*O*-ethyleburnamine (**75**).

White solid, isolated yield 60% (15 mg); m.p.: 98.0-98.5 °C ; ^{1}H NMR (400 MHz, $CDCl_3$): *δ* 7.61 (d, *J* = 7.8 Hz, 1H), 7.49 (d, *J* = 7.0 Hz, 1H), 7.20-7.11 (m, 2H), 5.56 (dd, *J* = 9.6, 5.4 Hz, 1H), 3.91 (s, 1H), 3.70-3.63 (m, 1H), 3.57-3.50 (m, 1H), 3.34-3.20 (m, 2H), 3.01-2.92 (m, 1H), 2.55-2.50 (m, 2H), 2.38-2.31 (m, 1H), 2.22 (dd, *J* = 13.8, 5.4 Hz, 1H), 2.13-2.05 (m, 1H), 1.90 (dd, *J* = 13.8, 9.6 Hz, 1H), 1.78-1.67 (m, 1H), 1.64-1.54 (m, 1H), 1.40-1.35 (m, 1H), 1.31-1.28 (m, 4H), 0.96-0.86 (m, 4H); ^{13}C NMR (101 MHz, $CDCl_3$): *δ* 136.6, 133.1, 128.7, 121.2, 120.0, 117.9, 111.9, 105.7, 81.9, 59.0, 58.8, 50.9, 44.3, 37.5, 36.5, 28.9, 25.3, 20.6, 16.8, 15.5, 7.6; IR (neat): *v* 3864, 1739, 1704, 1658, 1563, 1461, 1423, 670 cm^{-1}; HRMS (ESI): m/z [M + H]$^+$ calcd. for $C_{21}H_{29}N_2O$: 325.2274; found: 325.2269; $[\alpha]_D^{23}$ = +96.6 (*c* = 0.20, $CHCl_3$), lit[18]. $[\alpha]_D$ = +105 (*c* = 0.62, $CHCl_3$); **ent-75**: $[\alpha]_D^{23}$ = −97.8 (*c* = 0.20, $CHCl_3$).

Supplementary Table 43 ^{1}H NMR data comparison of the synthetic (+)-*O*-ethyleburnamine

**Lit[19]. Natural**	**This work**	**Δδ** (Lit – Our synthetic)
7.5 (d, *J* = 7 Hz, 1H)	7.61 (d, *J* = 7.8 Hz, 1H)	−0.11
7.4 (d, *J* = 7 Hz, 1H)	7.49 (d, *J* = 7.0 Hz, 1H)	−0.09
7.1 (t, *J* = 7 Hz, 2H)	7.20-7.11 (m, 2H)	-
5.5 (dd, *J* = 9, 5 Hz, 1H)	5.56 (dd, *J* = 9.6, 5.4 Hz, 1H)	−0.06
3.9 (s, 1H)	3.91 (s, 1H)	−0.01
3.7 (q, *J* = 7 Hz, 2H)	3.70-3.63 (m, 1H)	-
-	3.57-3.50 (m, 1H)	-
-	3.34-3.20 (m, 2H)	-
-	3.01-2.92 (m, 1H)	-

-	2.55-2.50 (m, 2H)	
-	2.38-2.31 (m, 1H)	-
-	2.22 (dd, J = 13.8, 5.4 Hz, 1H)	-
-	2.13-2.05 (m, 1H)	-
-	1.90 (dd, J = 13.8, 9.6 Hz, 1H)	-
-	1.78-1.67 (m, 1H)	-
-	1.64-1.54 (m, 1H)	-
-	1.40-1.35 (m, 1H)	-
1.3 (t, J = 7 Hz, 3H)	1.31-1.28 (m, 4H)	-
0.9 (t, J = 7 Hz, 3H)	0.96-0.86 (m, 4H)	-

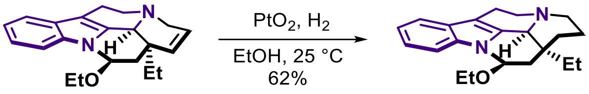

73 (–)-*O*-ethyl-isoeburnamine (**76**)

(−)-*O*-ethylisoeburnamine (76)

A mixture of **73** (16 mg, 0.05 mmol, 1.0 equiv) and PtO_2 (2.3 mg, 0.01 mmol, 0.2 equiv) in EtOH (0.5 mL) was stirred at 25 °C under an atmosphere of H_2 for 3 h. The mixture was filtered through a pad of celite and the filter cake was washed with MeOH. The filtrate was concentrated *in vacuo*. The residue was purified by column chromatography on silica gel (DCM: MeOH, 20:1) to give **76**.

White solid, isolated yield 62% (10 mg); m.p.: 93.7-94.5 °C ; ^{1}H NMR (400 MHz, $CDCl_3$): δ 7.48 (d, J = 7.3 Hz, 1H), 7.28 (d, J = 6.8 Hz, 1H), 7.18-7.10 (m, 2H), 5.57 (d, J = 3.2 Hz, 1H), 3.85 (s, 1H), 3.77-3.68 (m, 2H), 3.36-3.22 (m, 2H), 3.02-2.93 (m, 1H), 2.62-2.51 (m, 3H), 2.28-2.09 (m, 2H), 1.90-1.69 (m, 3H), 1.54-1.45 (m, 1H), 1.39-1.36 (m, 2H), 1.26 (t, J = 7.0 Hz, 3H), 0.93 (t, J = 7.6 Hz, 3H); ^{13}C NMR (101 MHz, $CDCl_3$): δ 135.3, 131.2, 128.7, 120.9, 119.9, 118.2, 110.5, 105.2, 81.6, 63.7, 59.3, 51.4, 44.8, 35.9, 34.4, 29.0, 25.6, 21.0, 16.9, 15.4, 7.7; IR (neat): v 3849, 1771, 1651, 1563, 1544, 1461, 1440, 670 cm^{-1}; HRMS (ESI): m/z [M + H]$^+$ calcd. for $C_{21}H_{29}N_2O$: 325.2274; found: 325.2269; $[\alpha]_D^{23}$ = −56.6 (c = 0.15, $CHCl_3$); **ent-76**: $[\alpha]_D^{23}$ = +57.0 (c = 0.10, $CHCl_3$).

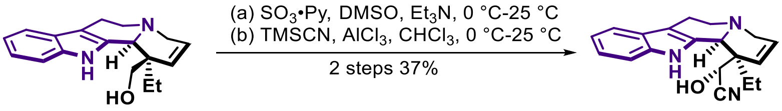

58 77

(*S*)-2-((1*R*,12b*S*)-1-ethyl-1,4,6,7,12,12b-hexahydroindolo[2,3-*a*]quinolizin-1-yl)-2-hydroxyacetonitrile (77)

Under N_2, to a solution of **58** (141 mg, 0.50 mmol, 1 equiv) in dry DMSO (2.5 mL) and Et_3N (1.3 mL) at 25 °C was added SO_3·Py (239 mg, 1.5 mmol, 3 equiv) and the mixture was stirred for 2 h at 25 °C. The reaction was quenched with H_2O and was extracted with EtOAc. The combined organic layers were washed with brine, dried over Na_2SO_4 and concentrated in *vacuo*. The residue was purified by column chromatography on a short silica gel (petroleum ether: EtOAc, 20:1) to give the product (154 mg). To a

solution of the product in dry $CHCl_3$ (11 mL) was added $AlCl_3$ (73 mg, 0.55 mmol, 1 equiv) in 0 °C and the mixture was stirred for 5 min. TMSCN (109 mg, 1.1 mmol, 2 equiv) was added dropwise. The mixture was allowed to warm to 25 °C and stirred for 1 h. The reaction mixture was quenched with saturated aqueous $NaHCO_3$. The aqueous layer was extracted with EtOAc. The combined organic layers were washed with brine, dried over Na_2SO_4 and concentrated *in vacuo*. The residue was purified by column chromatography on silica gel (petroleum ether: EtOAc, 10:1) to give **77**.

White solid, isolated yield 37% for 2 steps (58 mg); m.p.: 119.8-120.7 °C; ^{1}H NMR (400 MHz, $CDCl_3$): δ 7.74 (s, 1H), 7.53 (d, J = 7.8 Hz, 1H), 7.38 (d, J = 8.0 Hz, 1H), 7.23 (t, J = 8.0 Hz, 1H), 7.17 (t, J = 7.0 Hz, 1H), 6.29 (ddd, J = 10.1, 4.6, 1.6 Hz, 1H), 5.74 (dt, J = 10.1, 1.9 Hz, 1H), 4.23 (s, 1H), 3.99 (s, 1H), 3.52 (ddd, J = 16.9, 4.6, 1.6 Hz, 1H), 3.18-3.12 (m, 2H), 3.06-2.97 (m, 1H), 2.81-2.76 (m, 1H), 2.68 (td, J = 11.5, 3.5 Hz, 1H), 2.00-1.83 (m, 2H), 1.22 (t, J = 7.6 Hz, 3H); ^{13}C NMR (101 MHz, $CDCl_3$): δ 136.6, 130.5, 128.6, 128.6, 126.5, 122.7, 120.1, 118.5, 118.4, 113.1, 111.0, 68.2, 60.7, 54.3, 51.7, 45.3, 27.6, 21.1, 8.5; IR (neat): ν 3460, 2114, 1671, 1512, 1463, 670 cm^{-1}; HRMS (ESI): m/z [M + H]$^+$ calcd. for $C_{19}H_{22}N_3O$: 308.1757; found: 308.1761; $[\alpha]_D^{23}$ = −77.2 (c = 0.37, DCM).

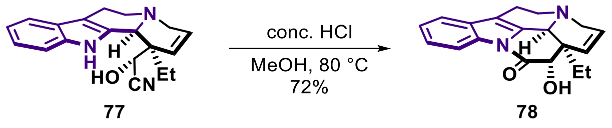

(4^1*S*, 13*S*, 13a*R*)-13a-ethyl-13-hydroxy-5,6,13,13a-tetrahydro-3*H*-indolo[3,2,1-*de*]pyrido[3,2,1-*ij*][1,5]naphthyridin-12(4^{1}H)-one (77)

To a solution of **77** (58 mg, 0.19 mmol, 1 equiv) in MeOH (1.9 mL) at 25 °C was added concentrated hydrochloric acid (3.8 mL) and the mixture was stirred for 4 h at 80 °C. The reaction was quenched with saturated aqueous K_2CO_3 and was extracted with EtOAc. The combined organic layers were washed with brine, dried over Na_2SO_4 and concentrated in *vacuo*. The residue was purified by column chromatography on silica gel (petroleum ether: EtOAc, 5:1) to give **78**.

White solid, isolated yield 72% (42 mg); m.p.: 129.5-130.1 °C; ^{1}H NMR (400 MHz, $CDCl_3$): δ 8.27-8.25 (m, 1H), 7.43-7.41 (m, 1H), 7.32-7.27 (m, 2H), 5.63 (dt, J = 10.2, 3.0 Hz, 1H), 5.48 (dt, J = 10.4, 2.7 Hz, 1H), 4.37 (s, 1H), 4.22 (s, 1H), 3.46-3.31 (m, 2H), 3.22-3.16 (m, 1H), 3.05-2.96 (m, 1H), 2.93 (dt, J = 17.9, 2.5 Hz, 1H), 2.55 (ddd, J = 17.0, 6.3, 2.2 Hz, 1H), 2.18-2.09 (m, 1H), 2.04-1.97 (m, 1H), 1.01 (t, J = 7.6 Hz, 3H); ^{13}C NMR (101 MHz, $CDCl_3$): δ 167.9, 134.4, 132.4, 130.6, 128.6, 124.3, 124.1, 123.7, 118.2, 116.2, 113.0, 74.9, 54.9, 49.4, 44.8, 44.0, 28.9, 16.5, 7.8; IR (neat): ν 3464, 1705, 1667, 1547, 1340, 671 cm^{-1}; HRMS (ESI): m/z [M + H]$^+$ calcd. for $C_{19}H_{21}N_2O_2$: 309.1598; found: 309.1594; $[\alpha]_D^{23}$ = −212.2 (c = 0.22, DCM).

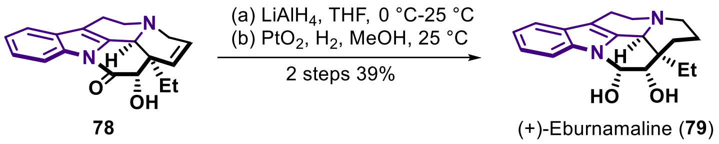

(+)-Eburnamaline (79)

To a solution of **78** (20 mg, 0.065 mmol, 1 equiv) in THF (3.3 mL) at 0 °C was added $LiAlH_4$ (7.4 mg, 0.195 mmol, 3 equiv) and the mixture was refluxed for 2 h. The reaction was quenched at 0 ºC by addition of H_2O (0.03 mL) followed by sequential addition of aq. NaOH (3 M, 0.03 mL), H_2O (0.08 mL) and Na_2SO_4 (30 mg). The mixture was stirred at 25 °C for 30 min then was filtrated through a pad of celite. The organic layers were concentrated in *vacuo*. A mixture of above residue and PtO_2 (1.5 mg, 0.0065 mmol, 0.1 equiv) in MeOH (1.3 mL) was stirred at 25 °C under an atmosphere of H_2 for 2 h. The mixture was filtered through a pad of celite and the filter cake was washed with MeOH. The filtrate was concentrated in *vacuo*. The residue was purified by column chromatography on silica gel (DCM: MeOH, 50:1) to give (+)-eburnamaline (**79**).

White solid, isolated yield 39% (8 mg); m.p.: 224.9-225.6 °C; ^{1}H NMR (400 MHz, $CDCl_3$): δ 7.82 (d, J = 7.7 Hz, 1H), 7.47 (d, J = 7.4 Hz, 1H), 7.21-7.12 (m, 2H), 5.60 (d, J = 3.2 Hz, 1H), 4.11 (s, 1H), 3.99 (d, J = 3.2 Hz, 1H), 3.28-3.15 (m, 2H), 2.93-2.85 (m, 1H), 2.60-2.56 (m, 1H), 2.44-2.30 (m, 3H), 1.86-1.72 (m, 2H), 1.44 (d, J = 13.1 Hz, 1H), 1.34 (d, J = 13.6 Hz, 1H), 0.94 (t, J = 7.6 Hz, 3H), 0.76 (td, J = 13.6, 3.2 Hz, 1H); ^{13}C NMR (101 MHz, $CDCl_3$): δ 137.3, 131.1, 128.6, 121.4, 120.2, 118.0, 112.4, 105.5, 77.5, 71.7, 56.0, 50.9, 44.7, 40.9, 22.8, 21.8, 19.9, 16.6, 6.9; IR (neat): ν 3470, 1711, 1647, 1572, 1516, 1468, 670 cm^{-1}; HRMS (ESI): m/z $[M + H]^+$ calcd. for $C_{19}H_{25}N_2O_2$: 313.1911; found: 313.1911; $[\alpha]_D^{23}$ = +47.2 (c = 0.11, $CHCl_3$), for (−)-enantiomer: lit[20]. $[\alpha]_D^{25}$ = −49 (c = 0.21, $CHCl_3$).

Supplementary Table 44 ^{1}H NMR data comparison of the synthetic (+)-eburnamaline

**Lit[20]. Natural**	**This work**	**Δδ** (Lit – Our synthetic)
7.79 (dd, J = 7, 1 Hz, 1H)	7.82 (d, J = 7.7 Hz, 1H)	−0.03
7.45 (dd, J = 7, 1 Hz, 1H)	7.47 (d, J = 7.4 Hz, 1H)	0.2
7.17 (td, J = 7, 1 Hz, 1H)	7.21-7.12 (m, 2H)	-
7.13 (td, J = 7, 1 Hz, 1H)		-
5.54 (d, J = 3 Hz, 1H)	5.60 (d, J = 3.2 Hz, 1H)	−0.06
4.02 (brs, 1H)	4.11 (s, 1H)	−0.09
3.90 (d, J = 3 Hz, 1H)	3.99 (d, J = 3.2 Hz, 1H)	−0.09
3.22 (dd, J = 14, 6 Hz, 1H)	3.28-3.15 (m, 2H)	-
3.14 (ddd, J = 14, 12, 6 Hz, 1H)		-
2.88 (dddd, J = 16, 12, 6, 2 Hz, 1H)	2.93-2.85 (m, 1H)	-
2.53 (brd, J = 13 Hz, 1H)	2.60-2.56 (m, 1H)	-
2.35 (m ,1H)	2.44-2.30 (m, 3H)	-
1.79 (dq, J = 14.5, 7.7 Hz, 1H)	1.86-1.72 (m, 2H)	-
1.70 (dt, J = 13, 3.6 Hz, 1H)		
1.37 (brd, J = 13 Hz, 1H)	1.44 (d, J = 13.1 Hz, 1H)	−0.07
1.29 (m ,1H)	1.34 (d, J = 13.6 Hz, 1H)	-
0.89 (t, J = 7.7 Hz, 3H)	0.94 (t, J = 7.6 Hz, 3H)	−0.05
0.66 (td, J = 13, 3.6 Hz, 3H)	0.76 (td, J = 13.6, 3.2 Hz, 1H)	−0.1

Supplementary Table 45 ^{13}C NMR data comparison of the synthetic (+)-eburnamaline

Lit[20]. Natural	This work	Δδ (Lit – Our synthetic)
137.2	137.3	−0.1
131.5	131.1	0.4
128.7	128.6	0.1
121.3	121.4	−0.1
120.2	120.2	0
118.0	118.0	0
112.3	112.4	−0.1
105.6	105.5	0.1
77.0	77.5	−0.5
71.7	71.7	0
55.8	56.0	−0.2
50.9	50.9	0
44.8	44.7	0.1
40.9	40.9	0
22.9	22.8	0.1
21.9	21.8	0.1
20.0	19.9	0.1
16.7	16.6	0.1
6.9	6.9	0

2.12 Synthesis of (−)-Arbornamine

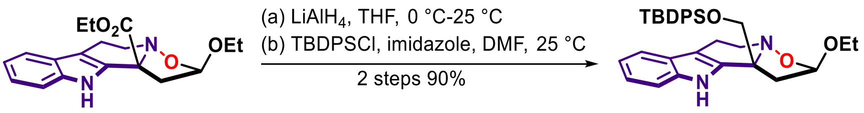

7b

80

(2*R*,11b*R*)-11b-(((*tert*-butyldiphenylsilyl)oxy)methyl)-2-ethoxy-1,2,5,6,11,11b-hexahydroisoxazolo[2',3':1,2]pyrido[3,4-*b*]indole (80)

To a solution of **7b** (165mg, 0.50 mmol, 1.0 equiv) in THF (5 mL) was added $LiAlH_4$ (23 mg, 0.60 mmol, 1.2 equiv) at 0 °C. After being stirred at 0 °C for 2 h, the reaction mixture was quenched with H_2O. The aqueous layer was extracted with EtOAc. The combined organic layers were washed with brine, dried over Na_2SO_4 and concentrated in *vacuo*. The above residue was dissolved in DMF (5 mL), and imidazole (41 mg, 0.60 mmol, 1.2 equiv) and TBDPSCl (165 mg, 0.60 mmol, 1.2 equiv) were added. The reaction was stirred at 25 °C for 24 h then was added H_2O. The aqueous layer was extracted with EtOAc. The combined organic layers were washed with brine, dried over Na_2SO_4 and concentrated in *vacuo*. The residue was purified by column chromatography on silica gel (petroleum ether: EtOAc, 40:1) to give **80**.

Yellow oil, isolated yield 90% for 2 steps (237 mg); ^{1}H NMR (400 MHz, $CDCl_3$): δ 8.16 (s, 1H), 7.56-7.51 (m, 5H), 7.44-7.40 (m, 2H), 7.33-7.29 (m, 4H), 7.23-7.12 (m, 3H), 5.15 (dd, J = 6.2, 2.0 Hz, 1H),

4.11 (d, J = 9.4 Hz, 1H), 3.92-3.82 (m, 2H), 3.69-3.63 (m, 1H), 3.49-3.42 (m, 1H), 3.33-3.26 (m, 1H), 3.05-2.97 (m, 1H), 2.81 (dd, J = 13.3, 6.3 Hz, 1H), 2.69-2.64 (m, 2H), 1.25 (t, J = 7.1 Hz, 3H), 1.10 (s, 9H); ^{13}C NMR (101 MHz, $CDCl_3$): δ 136.1, 135.7, 135.4, 134.2, 132.9, 132.3, 129.9, 129.8, 127.8, 127.8, 126.5, 121.7, 119.4, 118.4, 110.8, 108.1, 101.2, 70.2, 65.3, 63.9, 49.0, 46.1, 26.9, 19.3, 17.2, 15.1; IR (neat): ν 3863, 1739, 1704, 1658, 1563, 1511, 1461, 1391, 671 cm^{-1}; HRMS (ESI): m/z $[M + H]^+$ calcd. for $C_{32}H_{39}N_2O_3Si$: 527.2725; found: 527.2720; $[\alpha]_D^{23}$ = −14.4 (c = 1.0, DCM).

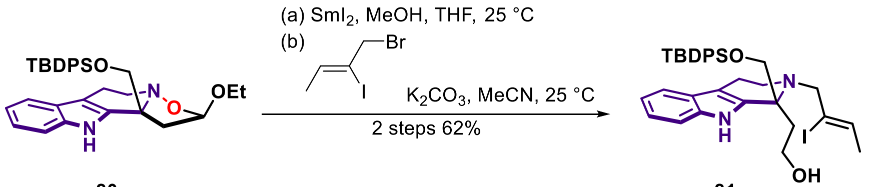

80 81

(R, Z)-2-(1-(((tert-butyldiphenylsilyl)oxy)methyl)-2-(2-iodobut-2-en-1-yl)-2,3,4,9-tetrahydro-1H-pyrido[3,4-b]indol-1-yl)ethan-1-ol (81)

To a stirred solution of **80** (237 mg, 0.45 mmol, 1.0 equiv) in MeOH (1 mL) was added SmI_2 (22.5 mL, 0.1 mmol in THF, 2.25 mmol, 5.0 equiv) at 25 °C. After stirring for 10 min at 25 °C, the reaction mixture was quenched with saturated aqueous $NaHCO_3$. The aqueous layer was extracted with EtOAc. The combined organic layers were washed with brine, dried over Na_2SO_4, and evaporated *in vacuo*. The above residue was dissolved in MeCN (4.5 mL), and K_2CO_3 (187 mg, 1.35 mmol, 3.0 equiv), 4 Å molecular sieves (237 mg) and (Z)-1-bromo-2-iodo-2-butene were added (352 mg, 1.35 mmol, 3.0 equiv). The reaction was stirred at 40 °C for 24 h then was concentrated in *vacuo*. The residue was purified by column chromatography on silica gel (petroleum ether: EtOAc, 4:1-2:1) to give **81**.

Yellow oil, isolated yield 62% for 2 steps (185 mg); ^{1}H NMR (400 MHz, $CDCl_3$): δ 8.21 (s, 1H), 7.57-7.53 (m, 3H), 7.50-7.47 (m, 2H), 7.45-7.41 (m, 2H), 7.37 (t, J = 7.6 Hz, 2H), 7.32 (t, J = 7.6 Hz, 2H), 7.19-7.11 (m, 3H), 5.96 (q, J = 6.2 Hz, 1H), 3.96-3.91 (m, 2H), 3.88-3.85 (m, 2H), 3.57-3.53 (m, 2H), 3.42 (d, J = 14.2 Hz, 1H), 3.16-3.11 (m, 1H), 2.96-2.90 (m, 1H), 2.87-2.80 (m, 1H), 2.76-2.72 (m, 1H), 2.35-2.29 (m, 1H), 2.08-2.01 (m, 1H), 1.85 (dd, J = 6.2, 0.7 Hz, 3H), 1.11 (s, 9H); ^{13}C NMR (101 MHz, $CDCl_3$): δ 136.1, 135.6, 135.4, 134.9, 132.5, 132.1, 131.7, 130.0, 129.9, 127.9, 127.8, 126.7, 121.7, 119.2, 118.2, 111.0, 110.8, 109.1, 67.1, 61.9, 60.0, 59.8, 44.0, 34.4, 26.9, 21.9, 20.8, 19.1; IR (neat): ν 3760, 1704, 1658, 1563, 1461, 1440, 671 cm^{-1}; HRMS (ESI): m/z $[M + H]^+$ calcd. for $C_{34}H_{42}N_2O_2ISi$: 665.2055; found: 665.2051; $[\alpha]_D^{23}$ = −15.9 (c = 0.30, DCM).

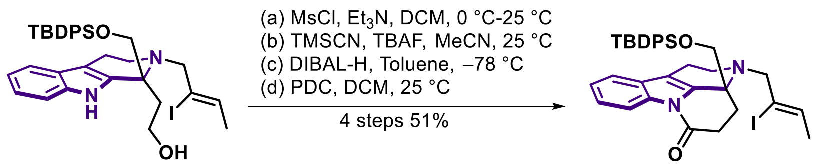

81 82

(R, Z)-3a-(((*tert*-butyldiphenylsilyl)oxy)methyl)-3-(2-iodobut-2-en-1-yl)-1,2,3,3a,4,5-hexahydro-6H-indolo[3,2,1-de][1,5]naphthyridin-6-one (82)

Under N_2, to a solution of **81** (160 mg, 0.24 mmol, 1.0 equiv) in dry DCM (2.4 mL) was added Et_3N (29 mg, 0.29 mmol, 1.2 equiv) and followed by addition of MsCl (33 mg, 0.29 mmol, 1.2 equiv). The reaction was stirred for 1 h at 25 °C, then was quenched by addition of saturated aqueous NH_4Cl (10 mL) and

extracted with EtOAc. The combined organic layers were washed with saturated brine, dried over Na_2SO_4 and concentrated in *vacuo*. The above residue was dissolved in MeCN (1.5 mL), and then a solution of TMSCN (24 mg, 0.24 mmol, 1.0 equiv) and TBAF (0.24 mL, 1.0 M in THF, 0.24 mmol, 1.0 equiv) in MeCN (1.5 mL) were added. The reaction was stirred at 25 °C for 24 h then was concentrated *in vacuo*. The residue was purified by column chromatography on silica gel (petroleum ether: EtOAc, 8:1) to give the cyanation product with minor inseparable impurities. Under N_2, to a solution of cyanation product in dry toluene (2.4 mL) at −78 °C was added dropwise DIBAL-H (0.48 mL, 1.0 M in Hexane, 0.48 mmol, 2.0 equiv) and the mixture was stirred for 15 min at −78°C. The reaction was quenched with Rochelle salts and the mixture was stirred for 1 h. The aqueous layer was extracted with EtOAc. The combined organic layers were washed with saturated brine, dried over Na_2SO_4 and concentrated in *vacuo*. The residue was dissolved in DCM (4.8 mL), and PDC (135 mg, 0.36 mmol, 1.5 equiv) was added. The reaction was stirred for 2 h at 25 °C and was directly charged to column chromatography on silica gel (petroleum ether: EtOAc, 18:1) to give **82**.

White solid, isolated yield 51% (82 mg); m.p.: 60.1-60.9 °C ; ^{1}H NMR (400 MHz, $CDCl_3$): δ 8.36 (d, J = 8.0 Hz, 1H), 7.65-7.63 (m, 2H), 7.57-7.55 (m, 2H), 7.48-7.34 (m, 7H), 7.32-7.24 (m, 2H), 5.95 (q, J = 6.2 Hz, 1H), 4.18 (d, J = 11.5 Hz, 1H), 4.04 (d, J = 15.0 Hz, 1H), 3.70 (d, J = 11.5 Hz, 1H), 3.24 (d, J = 15.0 Hz, 1H), 3.00 (dd, J = 12.3, 6.8 Hz, 1H), 2.88-2.84 (m, 1H), 2.78-2.61 (m, 4H), 2.56 (dd, J = 15.8, 4.3 Hz, 1H), 2.07-1.99 (m, 1H), 1.83 (d, J = 6.3 Hz, 3H), 1.08 (s, 9H); ^{13}C NMR (101 MHz, $CDCl_3$): δ 168.4, 135.7, 135.7, 135.2, 135.1, 132.5, 132.3, 130.6, 130.1, 130.0, 129.2, 127.9, 127.8, 124.6, 123.8, 118.1, 116.3, 114.6, 112.0, 61.8, 61.7, 56.6, 42.4, 31.0, 28.6, 26.9, 21.7, 20.9, 19.1; IR (neat): v 3862, 2925, 1704, 1643, 1563, 1264, 1029, 670 cm^{-1}; HRMS (ESI): m/z [M + H]$^+$ calcd. for $C_{35}H_{40}N_2O_2ISi$: 6675.1898; found: 675.1894; $[\alpha]_D^{23}$ = +42.3 (c = 0.10, DCM).

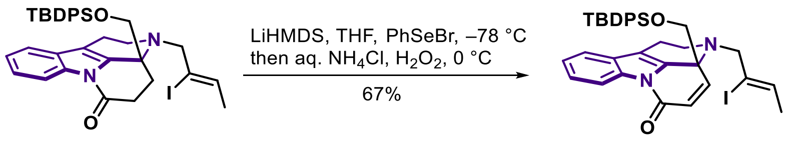

(*R*, *Z*)-3a-(((*tert*-butyldiphenylsilyl)oxy)methyl)-3-(2-iodobut-2-en-1-yl)-1,2,3,3a-tetrahydro-6*H*-indolo[3,2,1-*de*][1,5]naphthyridin-6-one (83)

Under N_2, to a solution of **82** (67 mg, 0.1 mmol, 1.0 equiv) in THF (1 mL) at −78 °C was added dropwise LiHMDS (0.5 mL, 1.0 M in THF, 0.5 mmol, 5.0 equiv). The resulting solution was stirred at −78 °C for 3 h, and then a solution of PhSeBr (35 mg, 0.15 mmol, 1.5 equiv) in THF (0.2 mL) was added. The reaction was stirred at −78 °C for 1 h and was warmed up to 25 °C for 30 min, then was quenched saturated aqueous NH_4Cl (10 mL) and extracted with EtOAc. The combined organic layers were washed with brine, dried over Na_2SO_4 and concentrated in *vacuo*. The residue was dissolved in DCM (1 mL), and then saturated aqueous NH_4Cl (0.1 mL) and H_2O_2 (50 µL) were added at 0 °C. The resulting solution was stirred at 0 °C for 1 h before quenching with saturated aqueous $Na_2S_2O_3$ (5 mL) and extracting with EtOAc. The combined organic layers were washed with brine, dried over Na_2SO_4 and concentrated in *vacuo*. The residue was purified by column chromatography on silica gel (petroleum ether: EtOAc, 12:1) to give **83**.

White oil, isolated yield 67% (45 mg); ^{1}H NMR (400 MHz, $CDCl_3$): δ 8.40 (d, J = 7.4 Hz, 1H), 7.62-7.60 (m, 2H), 7.47-7.29 (m, 9H), 7.16-7.12 (m, 2H), 7.02 (d, J = 10.0 Hz, 1H), 6.35 (d, J = 10.0 Hz, 1H),

5.89 (q, $J = 6.4$ Hz, 1H), 3.94 (d, $J = 9.8$ Hz, 1H), 3.87 (d, $J = 9.8$ Hz, 1H), 3.27-3.02 (m, 4H), 2.81-2.73 (m, 1H), 2.37 (dd, $J = 17.1$, 6.1 Hz, 1H), 1.80 (d, $J = 6.4$ Hz, 3H), 0.95 (s, 9H); ^{13}C NMR (101 MHz, $CDCl_3$): δ 161.1, 146.6, 135.7, 135.6, 135.5, 134.5, 132.9, 132.5, 132.4, 129.8, 129.6, 129.0, 127.7, 127.5, 126.4, 124.5, 123.7, 118.3, 116.1, 113.8, 109.5, 69.7, 62.2, 61.4, 41.3, 26.6, 21.7, 19.0, 16.3; IR (neat): v 3864, 1739, 1658, 1563, 1461, 671 cm^{-1}; HRMS (ESI): m/z $[M + H]^+$ calcd. for $C_{35}H_{38}N_2O_2ISi$: 673.1742; found: 673.1740; $[\alpha]_D^{23} = +115.8$ (c = 0.10, DCM).

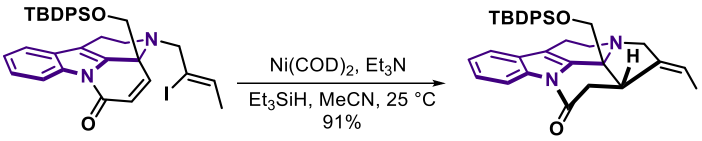

83 84

(3¹*S*, 12a*R*, *E*)-31-(((*tert*-butyldiphenylsilyl)oxy)methyl)-1-ethylidene-1,2,4,5,12,12a-hexahydroindolo[3,2,1-*de*]pyrrolo[3,2,1-*ij*][1,5]naphthyridin-11(3¹*H*)-one (84)

At 25 °C, to a suspension of $Ni(COD)_2$ (50 mg, 0.18 mmol, 3.0 equiv) in MeCN (0.6 mL) was added a solution of **83** (41 mg, 0.06 mmol, 1.0 equiv) and Et_3N (16 mg, 0.15 mmol, 2.5 equiv) in MeCN (0.6 mL) in the glovebox. The resulting solution was stirred for 20 min before adding Et_3SiH (21 mg, 0.18 mmol, 3.0 equiv). The reaction was stirred at 25 °C for 1 h, and was quenched by saturated aqueous $NaHCO_3$ solution and extracted with EtOAc. The combined organic layers were washed with brine, dried over Na_2SO_4 and concentrated *in vacuo*. The residue was purified by column chromatography on silica gel (petroleum: EtOAc, 1.2:1) to give **84**.

White solid, isolated yield 91% (30 mg); m.p.: 48.7-49.2 °C ; ^{1}H NMR (400 MHz, $CDCl_3$): δ 8.40 (d, J = 7.4 Hz, 1H), 7.54-7.52 (m, 2H), 7.47 (d, $J = 7.0$ Hz, 1H), 7.42-7.29 (m, 8H), 7.17 (t, $J = 7.6$ Hz, 2H), 5.34-5.29 (m, 1H), 4.02 (d, $J = 10.3$ Hz, 1H), 3.97 (d, $J = 10.2$ Hz, 1H), 3.46-3.09 (m, 7H), 2.87-2.78 (m, 1H), 2.45 (dd, $J = 17.2$, 7.3 Hz, 1H), 1.67 (d, $J = 7.1$ Hz, 3H), 0.95 (s, 9H); ^{13}C NMR (101 MHz, $CDCl_3$): δ 167.8, 137.4, 135.5, 135.5, 133.4, 132.6, 132.4, 130.1, 129.8, 129.7, 127.7 (2C), 127.6, 124.5, 123.8, 118.6, 118.2, 116.4, 113.8, 69.5, 62.7, 55.4, 43.5, 41.2, 35.1, 26.6, 19.0, 15.9, 13.9; IR (neat): v 3863, 1739, 1658, 1563, 1554, 1474, 670 cm^{-1}; HRMS (ESI): m/z $[M + H]^+$ calcd. for $C_{35}H_{39}N_2O_2Si$: 547.2775; found: 547.2773; $[\alpha]_D^{23} = -88.1$ (c = 0.05, DCM).

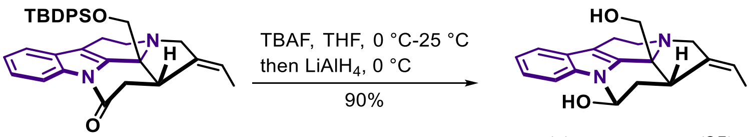

84 (−)-Arbornamine (**85**)

(−)-Arbornamine (85)

To a solution of **84** (27 mg, 0.05 mmol, 1.0 equiv) in THF (1 mL) at 0 °C was added TBAF (0.1 mL, 1.0 M in THF, 0.1 mmol, 2.0 equiv). The solution was warmed up to 25 °C for 2 h and then was cooled to 0 °C again. $LiAlH_4$ (0.2 mL, 1.0 M in THF, 0.2 mmol, 4.0 equiv) was added dropwise and the resulting solution was stirred at 0 °C for 20 min. The reaction was quenched by addition of saturated aqueous H_2O and extracted three times with DCM. The combined organic layers were washed with brine, dried over Na_2SO_4 and concentrated in *vacuo*. The residue was purified by column chromatography on silica gel

(DCM: MeOH, 20:1) to give (−)-arbornamine (**85**).

White solid, isolated yield 90% (14 mg); m.p.: 181.7-182.5 °C ; ^{1}H NMR (400 MHz, $CDCl_3$): *δ* 7.50 (d, *J* = 7.6 Hz, 1H), 7.42 (d, *J* = 8.1 Hz, 1H), 7.22 (t, *J* = 7.2 Hz, 1H), 7.15 (t, *J* = 7.9 Hz, 1H), 5.77-5.76 (m, 1H), 5.34 (q, *J* = 6.7 Hz, 1H), 4.02 (d, *J* = 10.3 Hz, 1H), 3.69 (d, *J* = 10.3 Hz, 1H), 3.47 (t, *J* = 9.0 Hz, 1H), 3.35-3.25 (m, 2H), 3.18-3.10 (m, 2H), 2.89-2.81 (m, 1H), 2.64-2.55 (m, 2H), 1.63 (d, *J* = 6.4 Hz, 3H), 1.04-0.97 (m, 1H); ^{13}C NMR (101 MHz, $CDCl_3$): *δ* 140.6, 137.8, 134.1, 128.0, 121.9, 120.1, 118.7, 116.3, 109.9, 107.7, 75.9, 67.3, 63.7, 53.9, 41.2, 37.1, 36.1, 16.5, 13.9; IR (neat): *v* 3863, 3760, 1704, 1658, 1563, 1544, 1461, 671 cm^{-1}; HRMS (ESI): m/z $[M + H]^+$ calcd. for $C_{19}H_{23}N_2O_2$: 311.1754; found: 311.1752; $[\alpha]_D^{23} = -43.2$ (*c* = 0.03, $CHCl_3$), lit[21]. $[\alpha]_D^{25} = -40$ (*c* = 0.07, $CHCl_3$).

Supplementary Table 46 ^{1}H NMR data comparison of the synthetic (−)-arbornamine

**Lit[21]. Natural**	**This work**	**Δδ** (Lit – Our synthetic)
7.52 (d, *J* = 8 Hz, 1H)	7.50 (d, *J* = 7.6 Hz, 1H)	0.02
7.44 (d, *J* = 8 Hz, 1H)	7.42 (d, *J* = 8.1 Hz, 1H)	0.02
7.23 (t, *J* = 8 Hz, 1H)	7.22 (t, *J* = 7.2 Hz, 1H)	0.01
7.15 (t, *J* = 8 Hz, 1H)	7.15 (t, *J* = 7.9 Hz, 1H)	0
5.86 (t, *J* = 3 Hz, 1H)	5.77-5.76 (m, 1H)	-
5.36 (q, *J* = 6 Hz, 1H)	5.34 (q, *J* = 6.7 Hz, 1H)	0.02
4.13 (d, *J* = 11 Hz, 1H)	4.02 (d, *J* = 10.3 Hz, 1H)	0.11
3.80 (d, *J* = 11 Hz, 1H)	3.69 (d, *J* = 10.3 Hz, 1H)	0.11
3.56 (dd, *J* = 10, 8 Hz, 1H)	3.47 (t, *J* = 9.0 Hz, 1H)	0.09
3.43 (m, 1H)	3.35-3.25 (m, 2H)	-
3.35 (d, *J* = 11 Hz, 1H)		
3.30 (d, *J* = 11 Hz, 1H)	3.18-3.10 (m, 2H)	-
3.21 (dd, *J* = 15, 6 Hz, 1H)		
2.91 (ddd, *J* = 16, 11, 6 Hz, 1H)	2.89-2.81 (m, 1H)	-
2.68 (ddd, *J* = 14, 8, 3 Hz, 1H)	2.64-2.55 (m, 2H)	-
2.64 (dd, *J* = 16, 6 Hz, 1H)		
1.65 (d, *J* = 6 Hz, 3H)	1.63 (d, *J* = 6.4 Hz, 3H)	0.02
1.09 (ddd, *J* = 14, 10, 3 Hz, 1H)	1.04-0.97 (m, 1H)	-

Supplementary Table 47 ^{13}C NMR data comparison of the synthetic (−)-arbornamine

**Lit[21]. Natural**	**This work**	**Δδ** (Lit – Our synthetic)
140.5	140.6	−0.1
138.0	137.8	0.2
134.0	134.1	−0.1
128.2	128.0	0.2
122.2	121.9	0.3
120.3	120.1	0.2
118.8	118.7	0.1
116.8	116.3	0.5

110.1	109.9	0.2
107.8	107.7	0.1
76.0	75.9	0.1
67.1	67.3	−0.2
64.3	63.7	0.6
54.2	53.9	−0.3
41.4	41.2	0.2
37.1	37.1	0
36.1	36.1	0
16.6	16.5	0.1
14.1	13.9	0.2

3. Crystallographic data

Crystallographic data have been deposited with the Cambridge Crystallographic Data Centre as supplementary publication nos. CCDC-2165190 (**3g**), CCDC-2165189 (**5d**), CCDC-2165191 (**7b**), CCDC-2289028 (**7h**). Copies of these data can be obtained free of charge from the Cambridge Crystallographic Data Centre via www.ccdc.cam.ac.uk/data_request/cif.

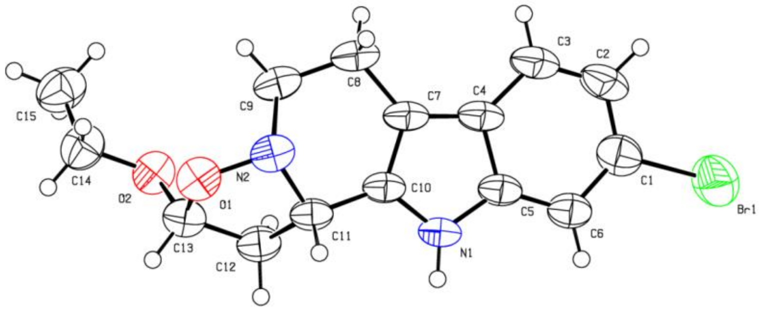

Supplementary Table 47 Crystal data and structure refinement for 3g

Identification code	**3g**
Empirical formula	$C_{15}H_{17}BrN_2O_2$
Formula weight	337.21
Temperature/K	293(2)
Crystal system	monoclinic
Space group	$P2_1$
a/Å	8.69468(17)
b/Å	7.17028(16)
c/Å	12.4524(3)
α/°	90
β/°	96.9129(18)
γ/°	90
Volume/Å^3	770.68(3)
Z	2
ρ_{calc}g/cm^3	1.453
μ/mm^{-1}	3.663

F(000)	344.0
Crystal size/mm^3	0.3 × 0.15 × 0.15
Radiation	Cu Kα (λ = 1.54184)
2Θ range for data collection/°	7.15 to 143.416
Index ranges	$-10 \le h \le 10$, $-8 \le k \le 8$, $-15 \le l \le 15$
Reflections collected	9808
Independent reflections	2974 [R_{int} = 0.0288, R_{sigma} = 0.0243]
Data/restraints/parameters	2974/1/182
Goodness-of-fit on F^2	1.079
Final R indexes [I>=2σ (I)]	R_1 = 0.0386, wR_2 = 0.1039
Final R indexes [all data]	R_1 = 0.0399, wR_2 = 0.1061
Largest diff. peak/hole / e Å^{-3}	0.31/-0.82
Flack parameter	-0.037(14)

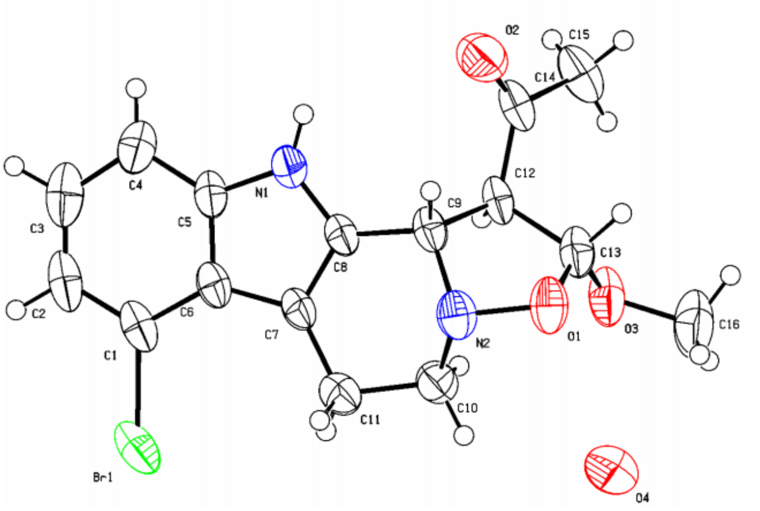

Supplementary Table 48 Crystal data and structure refinement for 5d

Identification code	**5d**
Empirical formula	$C_{16}H_{17}BrN_2O_3$
Formula weight	746.45
Temperature/K	293(2)
Crystal system	monoclinic
Space group	C2
a/Å	17.6425(3)
b/Å	9.20040(10)
c/Å	11.3595(2)
α/°	90
β/°	119.783(2)
γ/°	90
Volume/Å^3	1600.30(5)
Z	2
ρ_{calc}g/cm^3	1.549
μ/mm^{-1}	3.664
F(000)	760.0
Crystal size/mm^3	0.35 × 0.25 × 0.1

Radiation	CuKα (λ = 1.54178)
2Θ range for data collection/°	8.97 to 133.108
Index ranges	-20 ≤ h ≤ 20, -10 ≤ k ≤ 10, -13 ≤ l ≤ 12
Reflections collected	8599
Independent reflections	2833 [R_{int} = 0.0579, R_{sigma} = 0.0405]
Data/restraints/parameters	2833/1/206
Goodness-of-fit on F^2	1.041
Final R indexes [I>=2σ (I)]	R_1 = 0.0721, wR_2 = 0.1878
Final R indexes [all data]	R_1 = 0.0723, wR_2 = 0.1882
Largest diff. peak/hole / e $Å^{-3}$	0.47/-1.09
Flack parameter	-0.02(5)

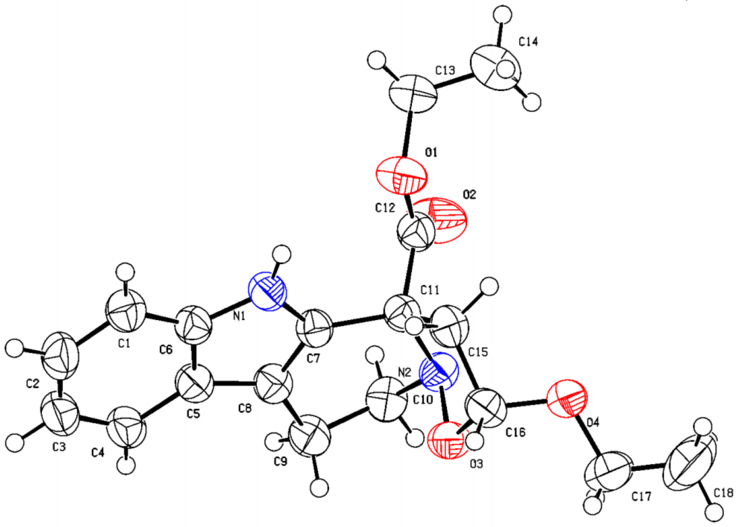

Supplementary Table 49 Crystal data and structure refinement for 7b

Identification code	**7b**
Empirical formula	$C_{18}H_{22}N_2O_4$
Formula weight	330.37
Temperature/K	293(2)
Crystal system	orthorhombic
Space group	$P2_12_12_1$
a/Å	9.75440(10)
b/Å	11.27700(10)
c/Å	15.7176(2)
α/°	90
β/°	90
γ/°	90
Volume/Å^3	1728.94(3)
Z	4
ρ_{calc}g/cm^3	1.269
μ/mm^{-1}	0.739
F(000)	704.0

Crystal size/mm^3	0.3 × 0.2 × 0.2
Radiation	Cu Kα (λ = 1.54184)
2Θ range for data collection/°	9.652 to 143.618
Index ranges	$-11 \leq h \leq 12$, $-13 \leq k \leq 13$, $-19 \leq l \leq 19$
Reflections collected	23964
Independent reflections	3376 [R_{int} = 0.0259, R_{sigma} = 0.0129]
Data/restraints/parameters	3376/0/219
Goodness-of-fit on F^2	1.070
Final R indexes [I>=2σ (I)]	R_1 = 0.0329, wR_2 = 0.0916
Final R indexes [all data]	R_1 = 0.0340, wR_2 = 0.0930
Largest diff. peak/hole / e $Å^{-3}$	0.21/-0.14
Flack parameter	0.08(6)

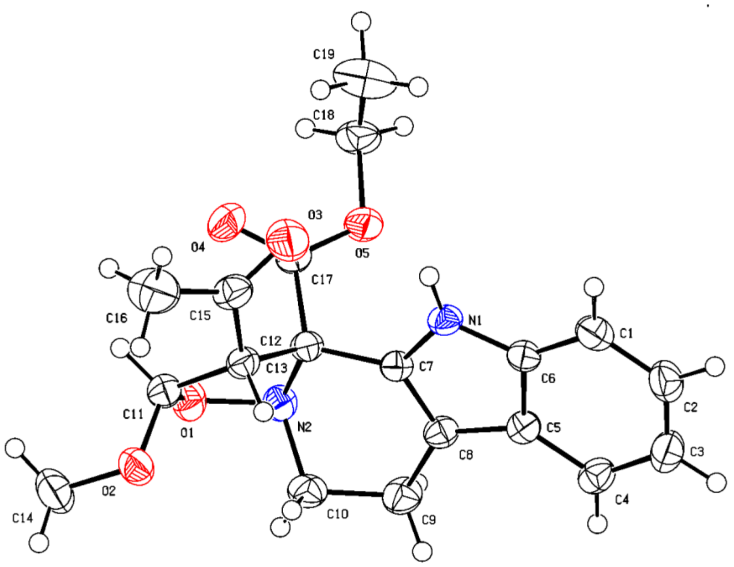

Supplementary Table 50 Crystal data and structure refinement for 7h

Identification code	**7h**
Empirical formula	$C_{19}H_{22}N_2O_5$
Formula weight	358.38
Temperature/K	291.2(3)
Crystal system	monoclinic
Space group	P21/n
a/Å	9.8848(3)
b/Å	18.9131(5)
c/Å	10.6328(4)
α/°	90
β/°	115.789(4)
γ/°	90
Volume/Å3	1789.84(11)
Z	4
ρcalcg/cm3	1.330
μ/mm-1	0.802
F(000)	760.0
Crystal size/mm3	0.4 × 0.4 × 0.4

Radiation	Cu Kα (λ = 1.54184)
2Θ range for data collection/°	9.352 to 133.146
Index ranges	$-11 \leq h \leq 11$, $-22 \leq k \leq 22$, $-12 \leq l \leq 12$
Reflections collected	20283
Independent reflections	3158 [Rint = 0.0626, Rsigma = 0.0265]
Data/restraints/parameters	3158/0/239
Goodness-of-fit on F2	1.051
Final R indexes [I>=2σ (I)]	R1 = 0.0403, wR2 = 0.1067
Final R indexes [all data]	R1 = 0.0425, wR2 = 0.1088
Largest diff. peak/hole / e Å-3	0.22/-0.18

4. Computational details

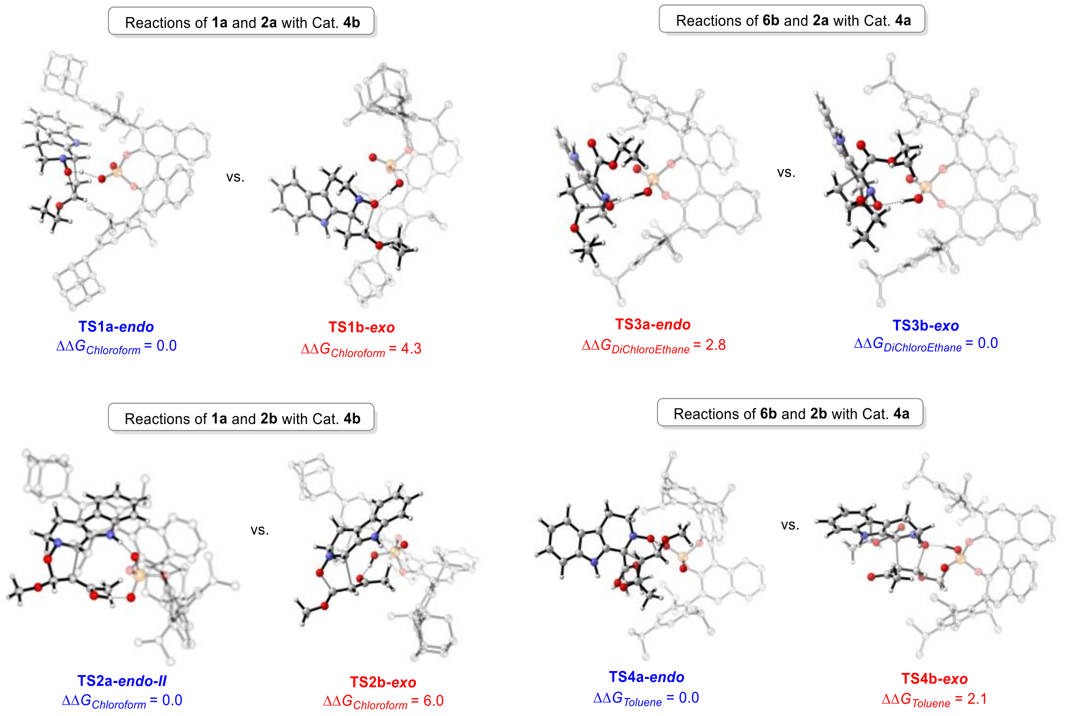

Supplementary Figure 6. The structures of the optimal transition states in 1,3-dipolar cycloaddition reactions.

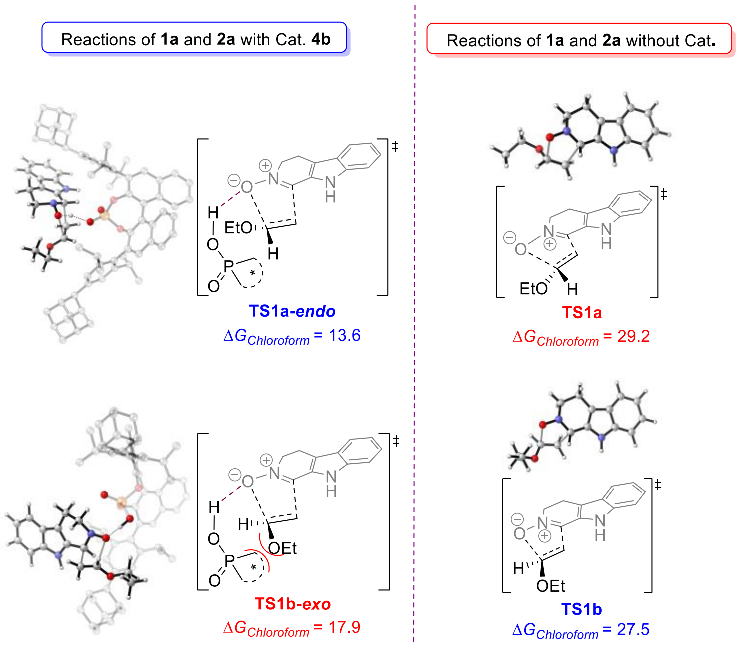

Supplementary Figure 7. The key transition states for 1,3-dipolar cycloaddition reactions of substrates **1a** and **2a** with/without catalyst **4b**. Gibbs free energy (in kcal/mol) obtained at the level of SMD(solvent = chloroform)-B3LYP-D3/def2TZVP//B3LYP-D3/6-31G*.

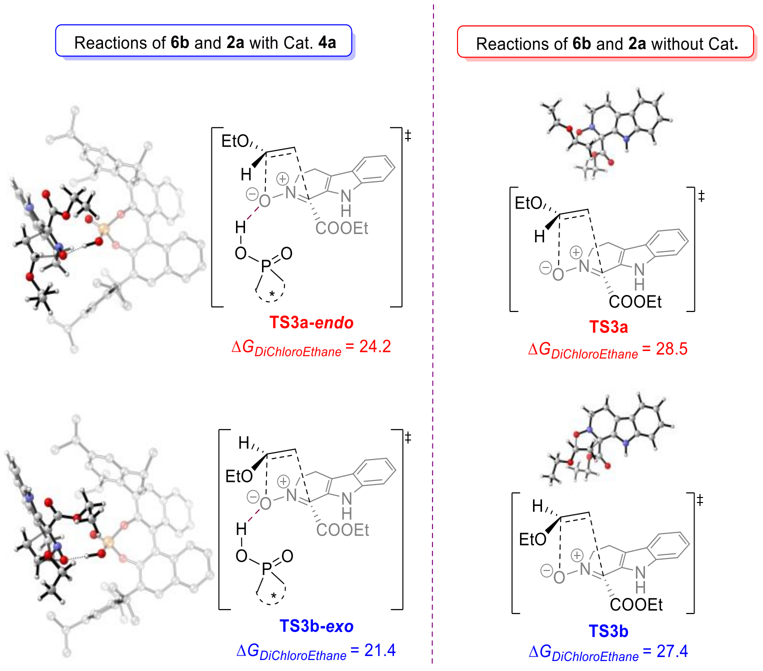

Supplementary Figure 8. The key transition states for 1,3-dipolar cycloaddition reactions of substrates **6b** and **2a** with/without catalyst **4a**. Gibbs free energy (in kcal/mol) obtained at the level of SMD(solvent = chloroform)-B3LYP-D3/def2TZVP//B3LYP-D3/6-31G*.

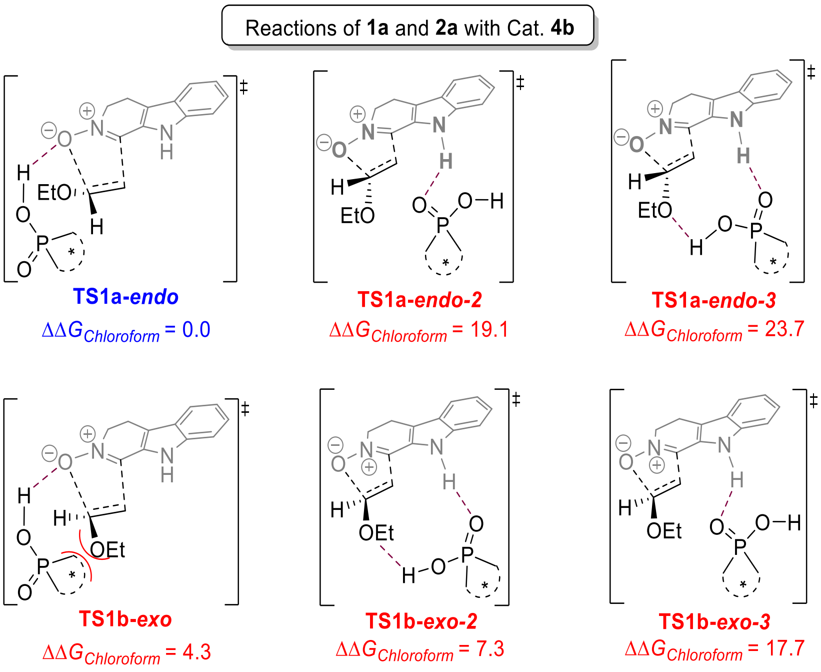

Supplementary Figure 9. The complete transition state of substrate **1a** and **2a** undergoing 1,3-dipolar cycloaddition reaction under the action of catalyst **4b**. Gibbs free energy (in kcal/mol) obtained at the level of SMD(solvent = chloroform)-B3LYP-D3/def2TZVP//B3LYP-D3/6-31G*.

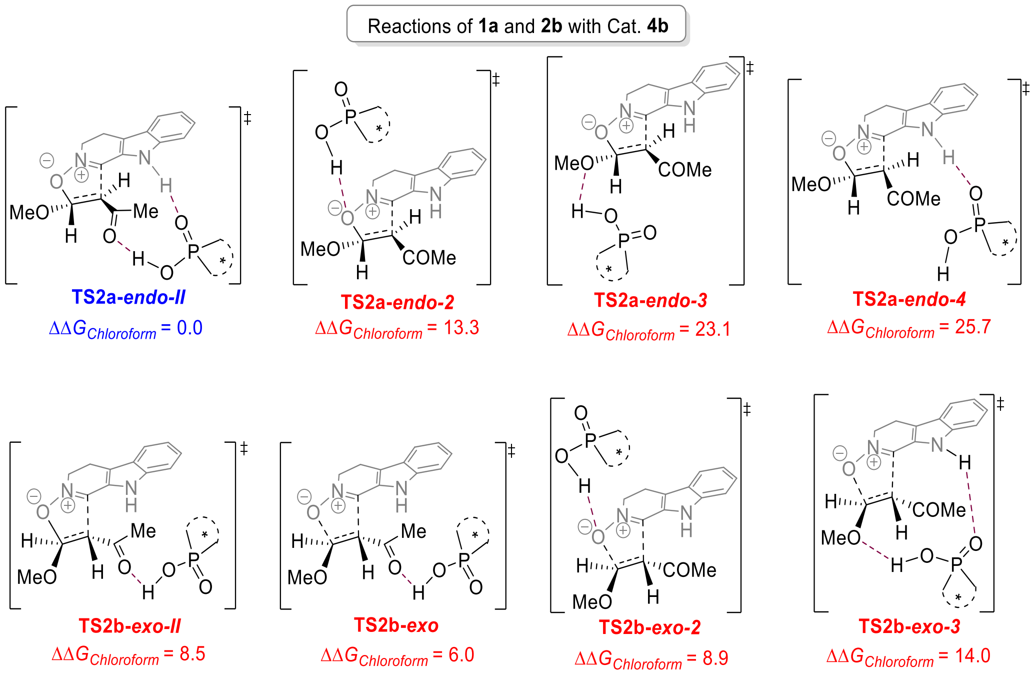

Supplementary Figure 10. The complete transition state of substrate **1a** and **2b** undergoing 1,3-dipolar cycloaddition reaction under the action of catalyst **4b**. Gibbs free energy (in kcal/mol) obtained at the level of SMD(solvent = chloroform)-B3LYP-D3/def2TZVP//B3LYP-D3/6-31G*.

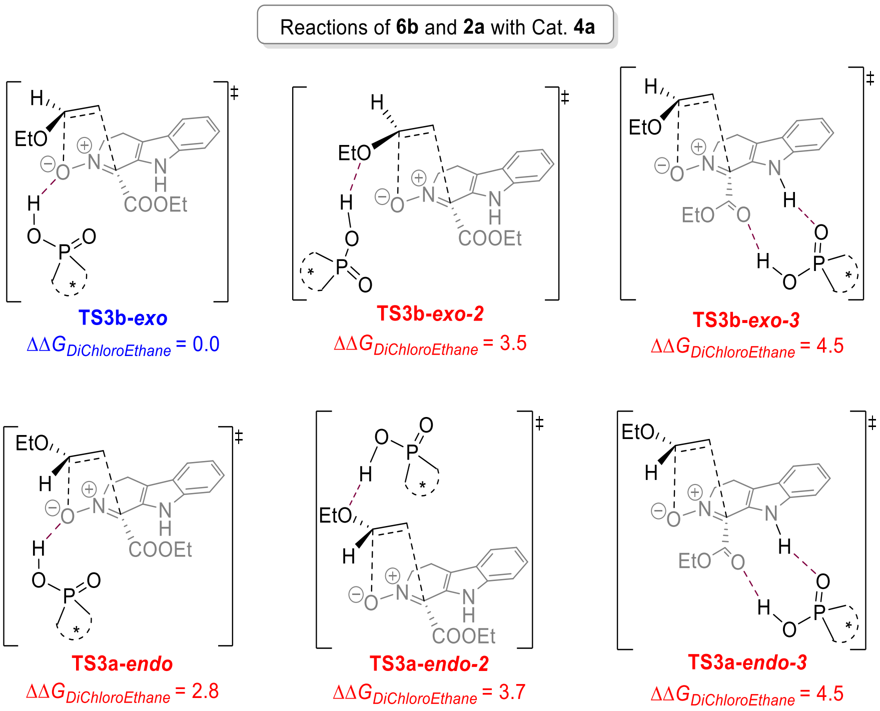

Supplementary Figure 11. The complete transition state of substrate **6b** and **2a** undergoing 1,3-dipolar cycloaddition reaction under the action of catalyst **4a**. Gibbs free energy (in kcal/mol) obtained at the level of SMD(solvent = dichloroethane)-B3LYP-D3/def2TZVP//B3LYP-D3/6-31G*.

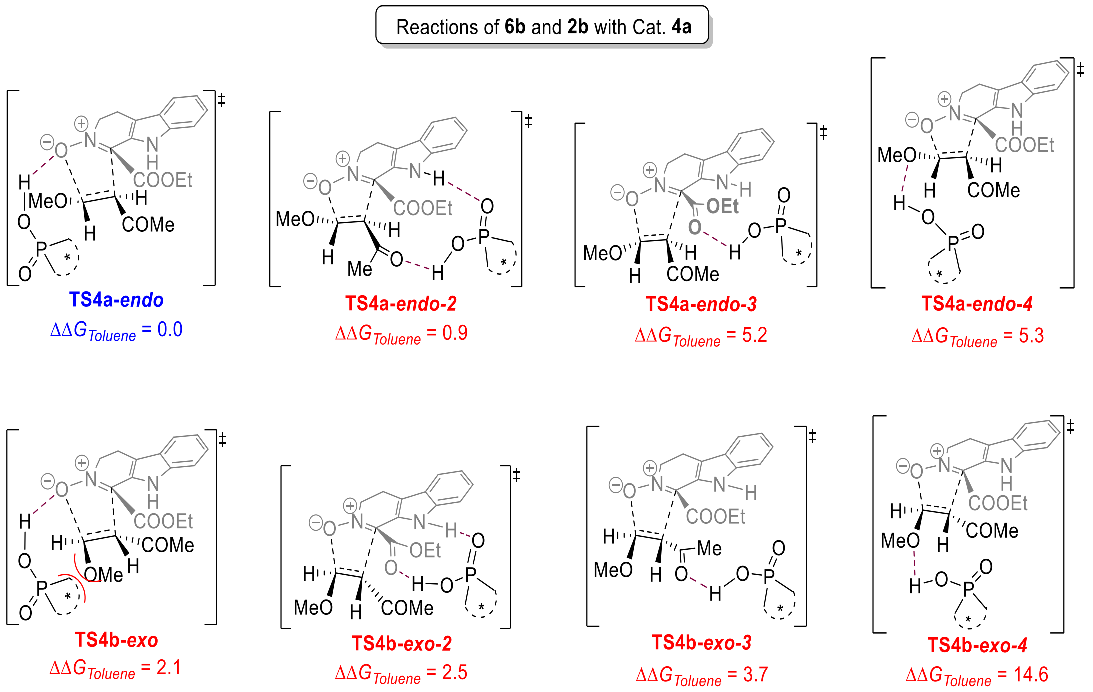

Supplementary Figure 12. The complete transition state of substrate **6b** and **2b** undergoing 1,3-dipolar cycloaddition reaction under the action of catalyst **4a**. Gibbs free energy (in kcal/mol) obtained at the

level of SMD(solvent = toluene)-B3LYP-D3/def2TZVP//B3LYP-D3/6-31G*.

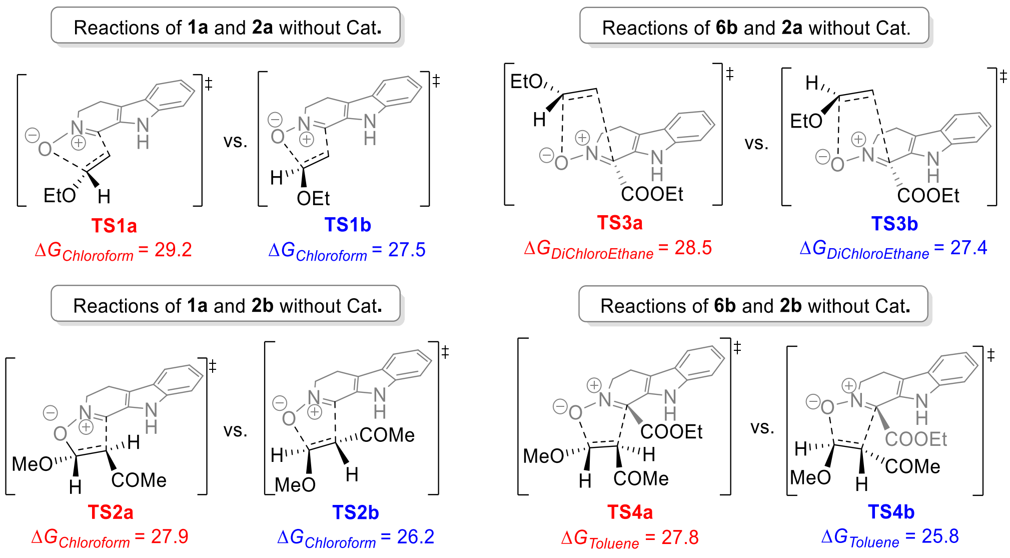

Supplementary Figure 13. The key transition states of uncatalyzed 1,3-dipolar cycloaddition reactions. Gibbs free energy (in kcal/mol) obtained at the level of SMD(solvent)-B3LYP-D3/def2TZVP//B3LYP-D3/6-31G*. In accordance with experimental conditions, chloroform was utilized as the solvent for reactions involving **1a** and **2a/2b**. Dichloroethane was employed as the solvent for reactions of **6b** and **2a**. Additionally, toluene was the chosen solvent for reactions of **6b** and **2b**.

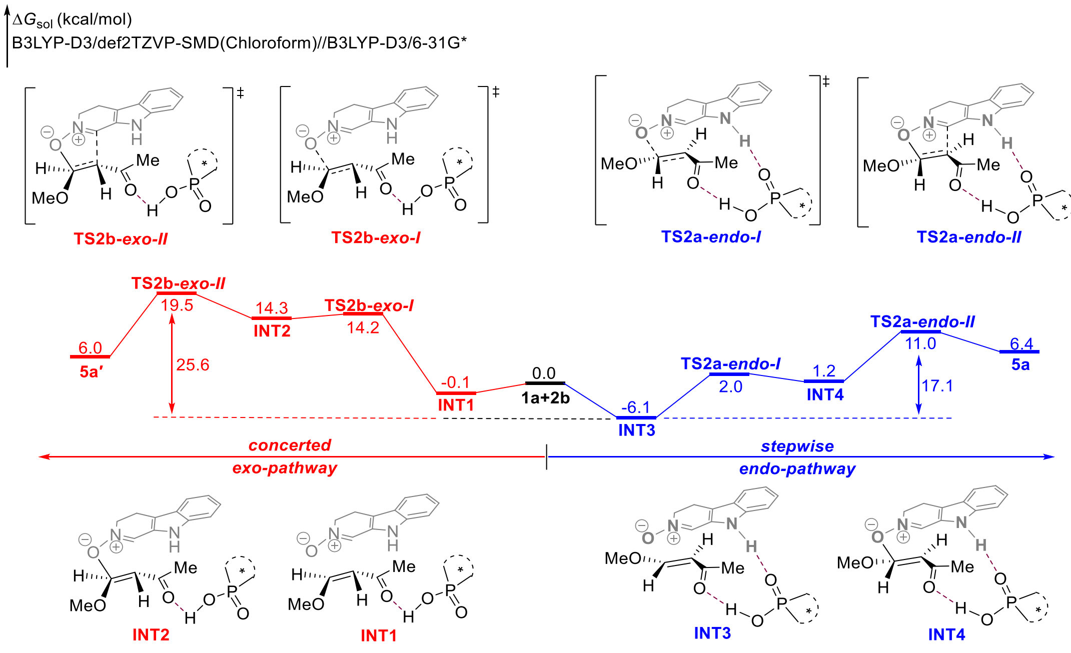

Supplementary Figure 14. Gibbs free energy profiles of the stepwise 1,3-dipolar cycloaddition reaction of substrates **1a** and **2b** catalyzed by catalyst **4b**. Gibbs free energy (in kcal/mol) obtained at the level of SMD(solvent = chloroform)-B3LYP-D3/def2TZVP//B3LYP-D3/6-31G*.

5. Copies of NMR spectra and HPLC spectra

Supplementary Figure 15: ^{1}H NMR of 1a (400 MHz, DMSO-d$_6$)

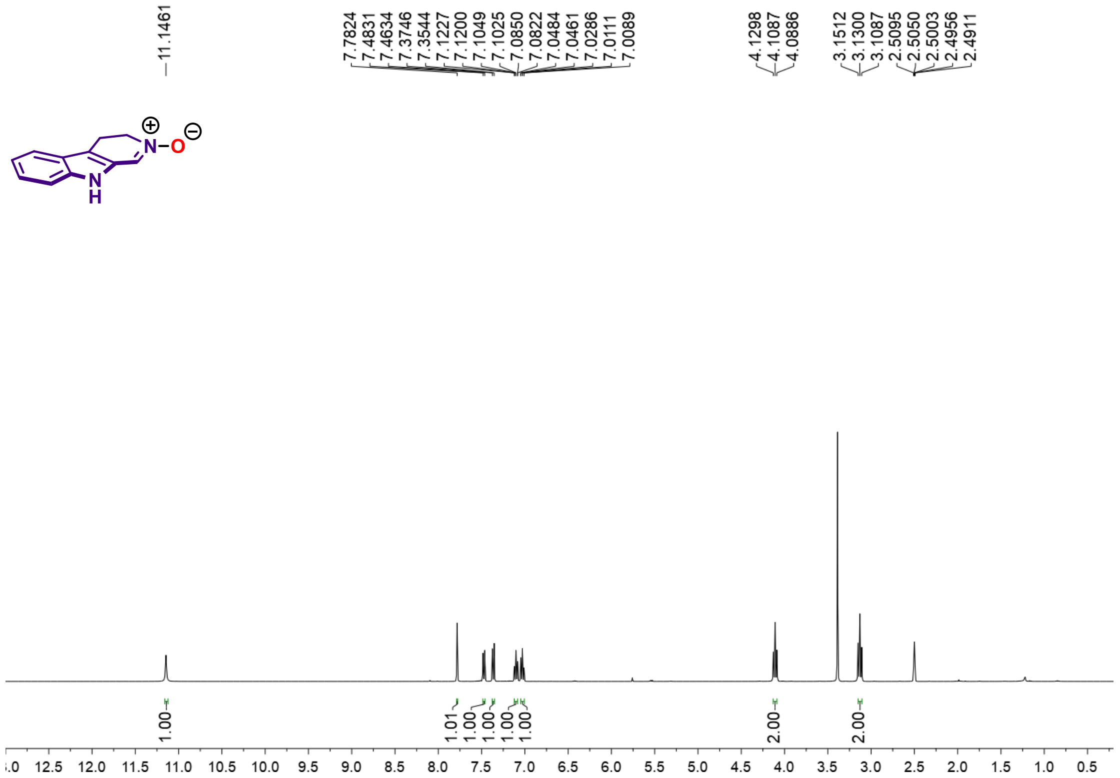

Supplementary Figure 16: ^{13}C NMR of 1a (101 MHz, DMSO-d$_6$)

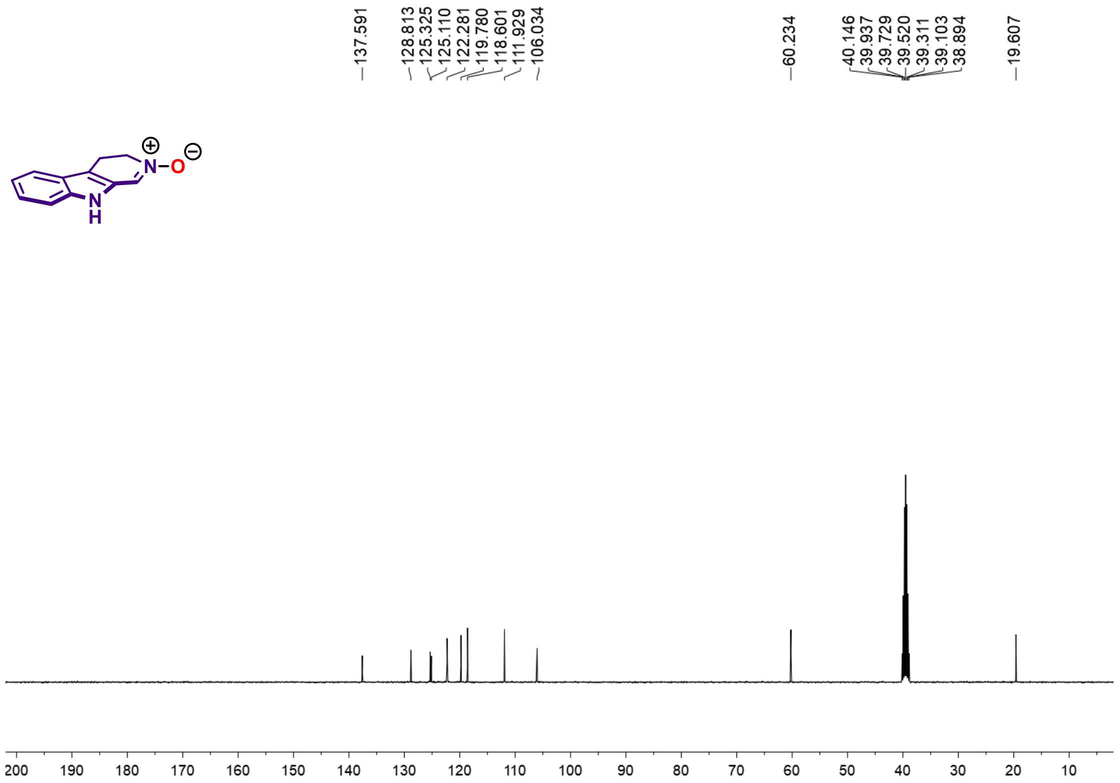

Supplementary Figure 17: ^{1}H NMR of 1b (400 MHz, DMSO-d_6)

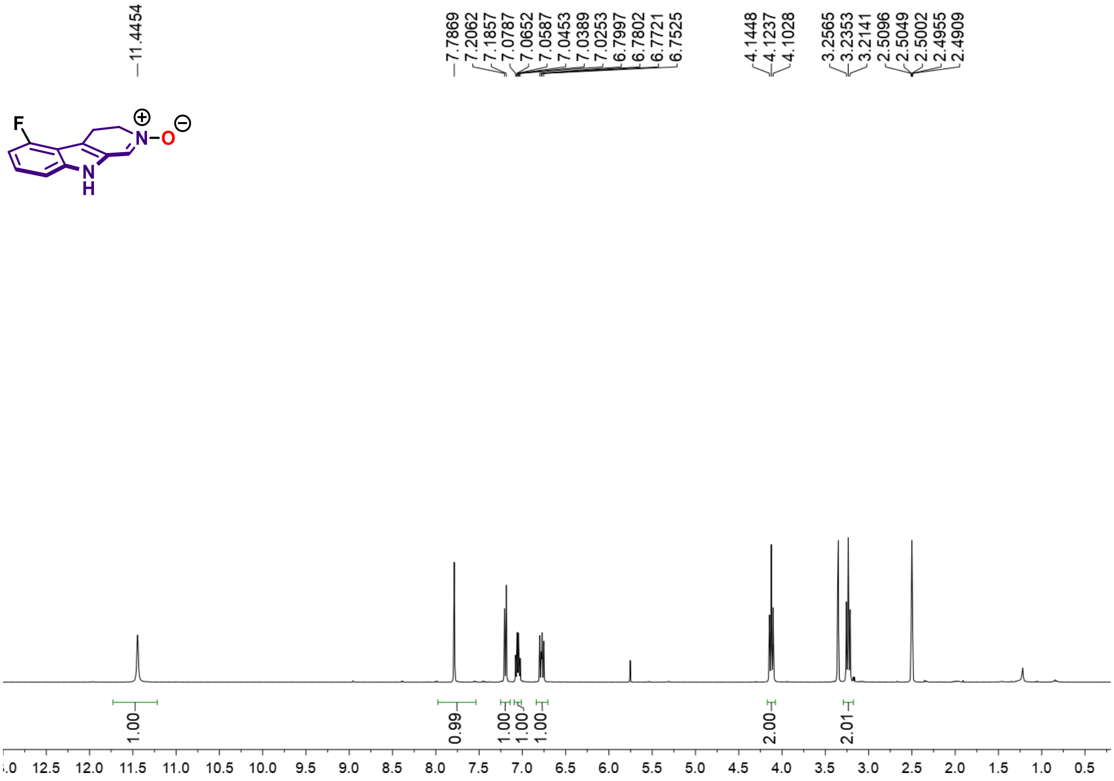

Supplementary Figure 18: ^{13}C NMR of 1a (101 MHz, DMSO-d_6)

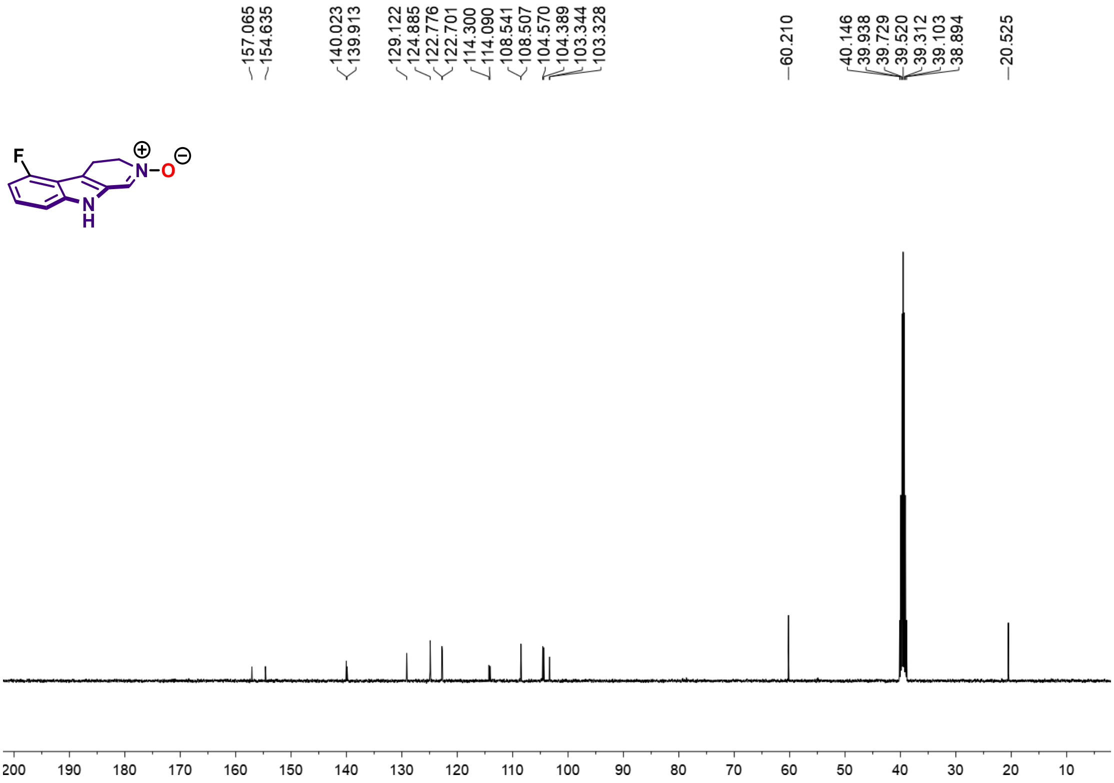

Supplementary Figure 19: ^{1}H NMR of 1c (400 MHz, DMSO-d$_6$)

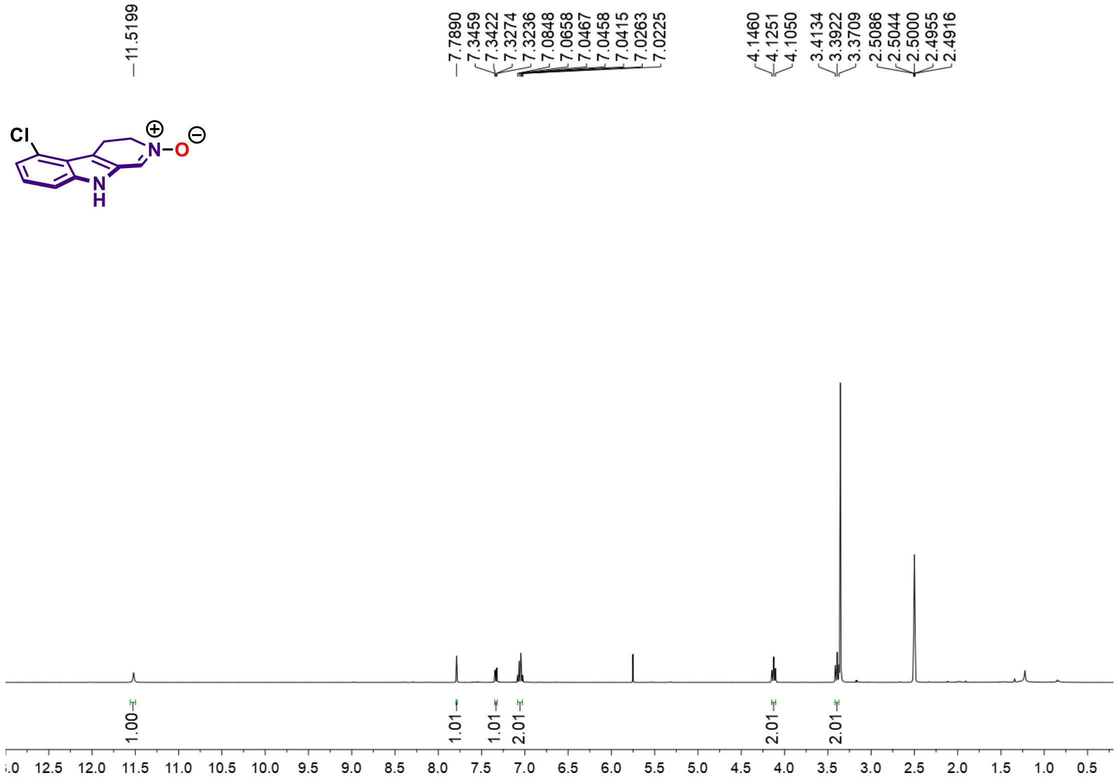

Supplementary Figure 20: ^{13}C NMR of 1c (101 MHz, DMSO-d$_6$)

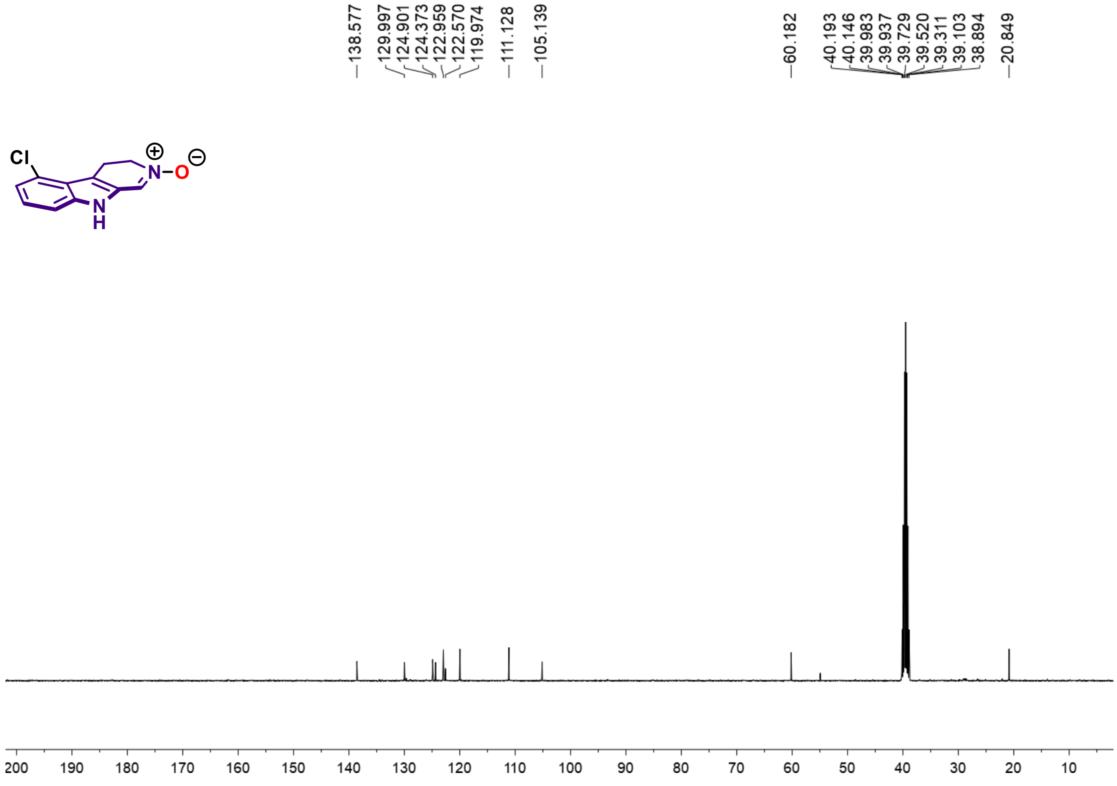

Supplementary Figure 21: ^{1}H NMR of 1d (400 MHz, DMSO-d$_6$)

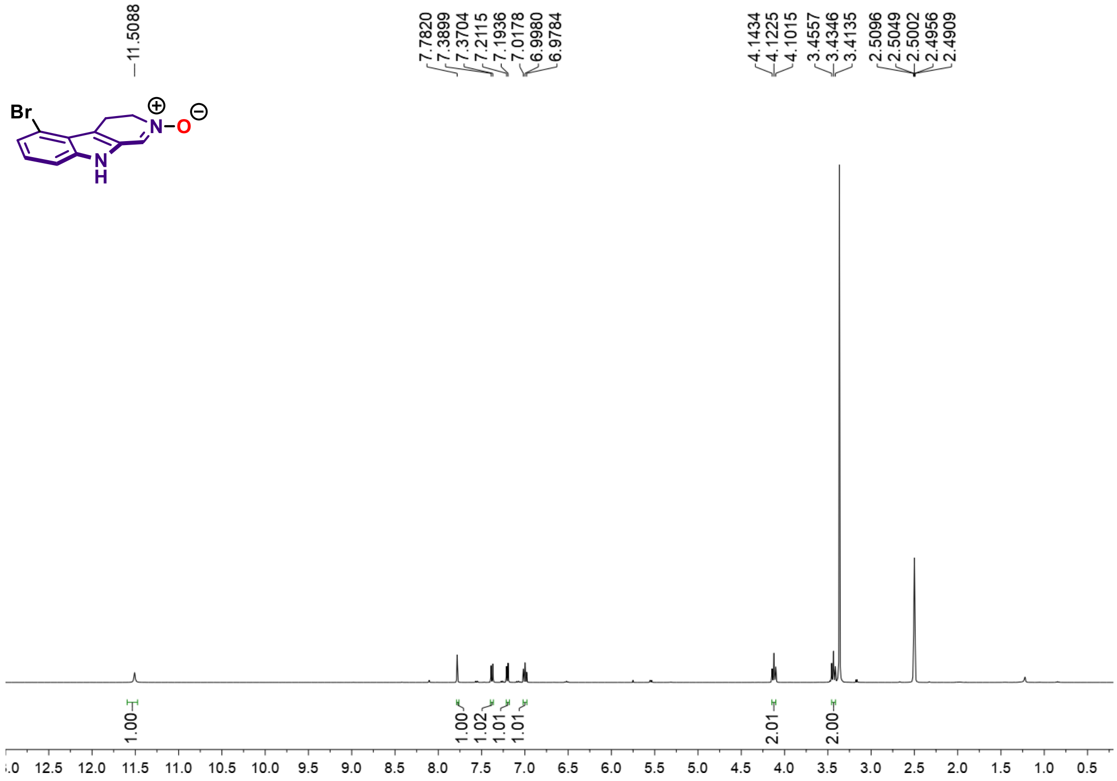

Supplementary Figure 22: ^{13}C NMR of 1d (101 MHz, DMSO-d$_6$)

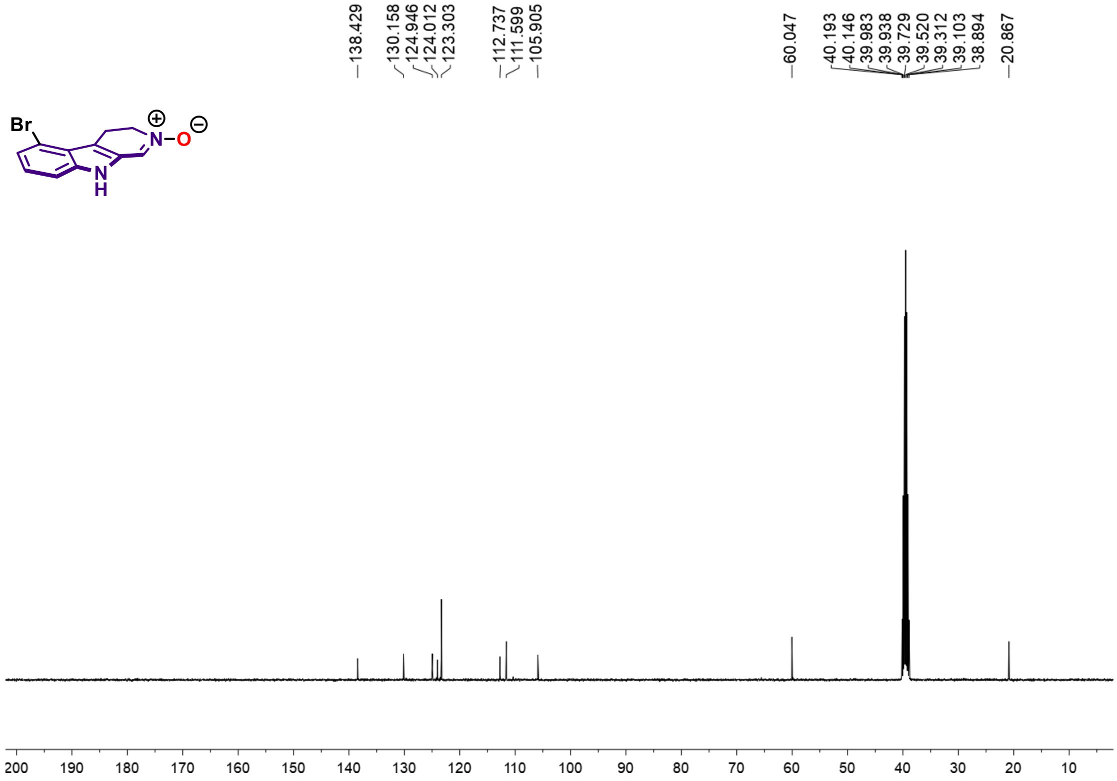

Supplementary Figure 23: ^{1}H NMR of 1e (400 MHz, DMSO-d₆)

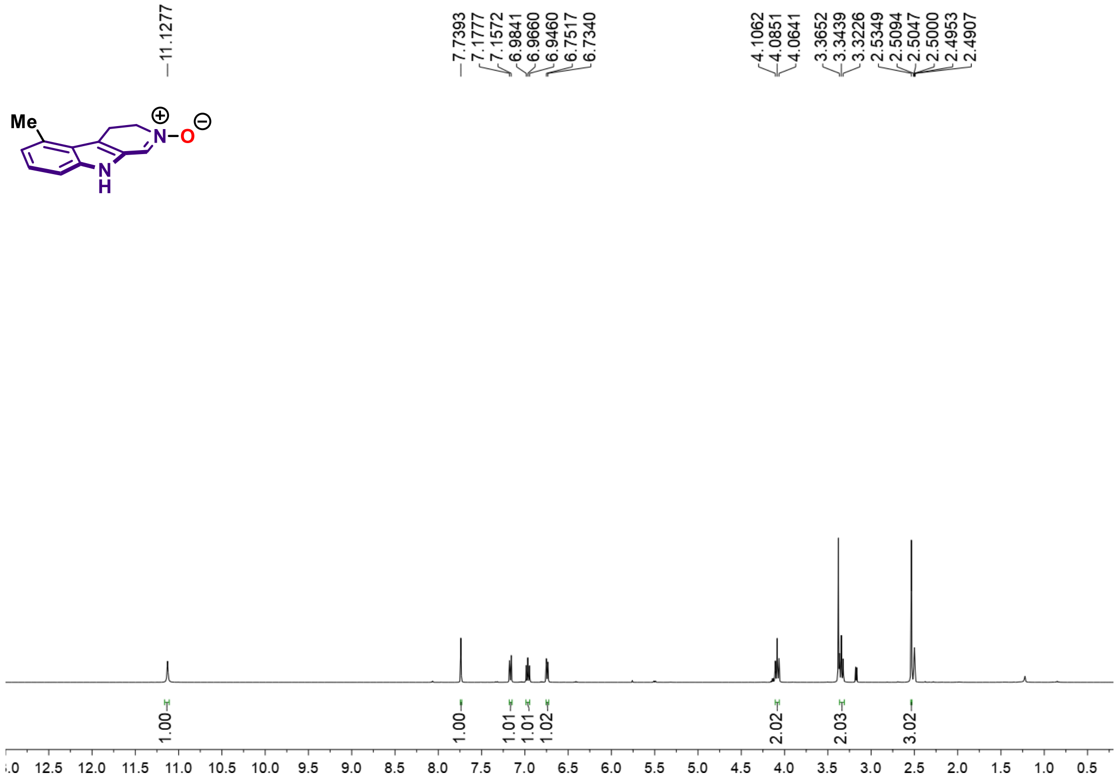

Supplementary Figure 24: ^{13}C NMR of 1e (101 MHz, DMSO-d₆)

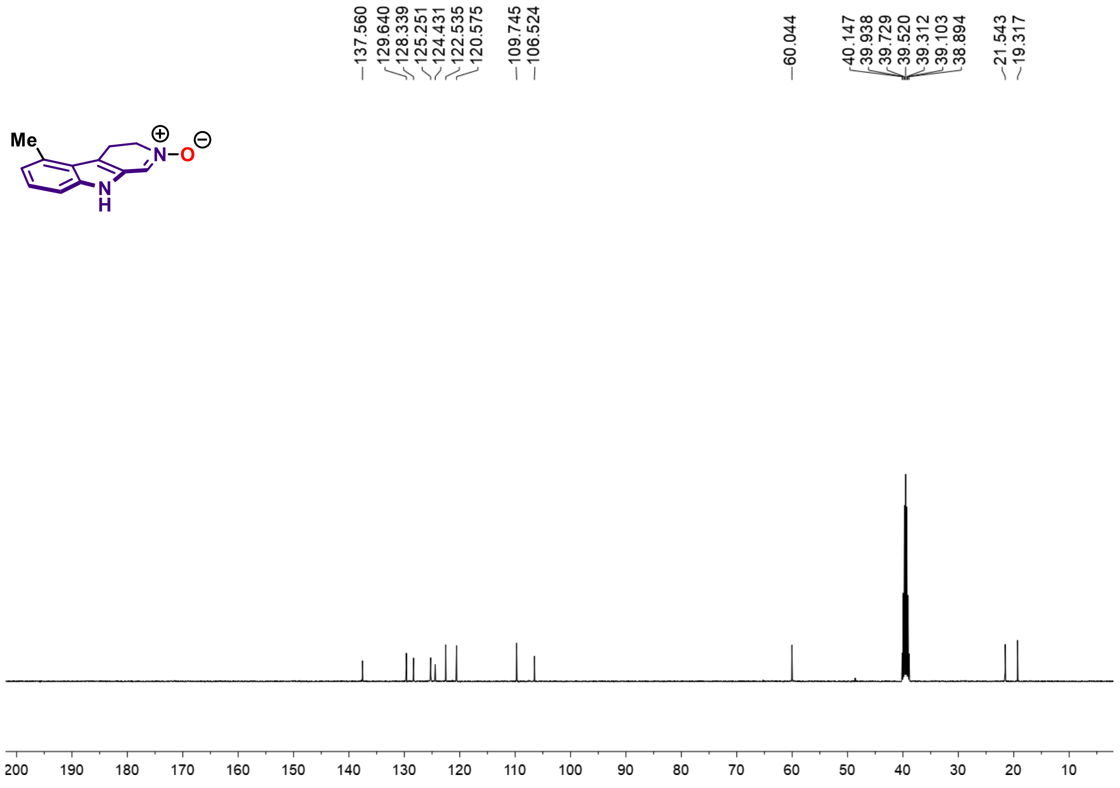

Supplementary Figure 25: ^{1}H NMR of 1f (400 MHz, DMSO-d$_6$)

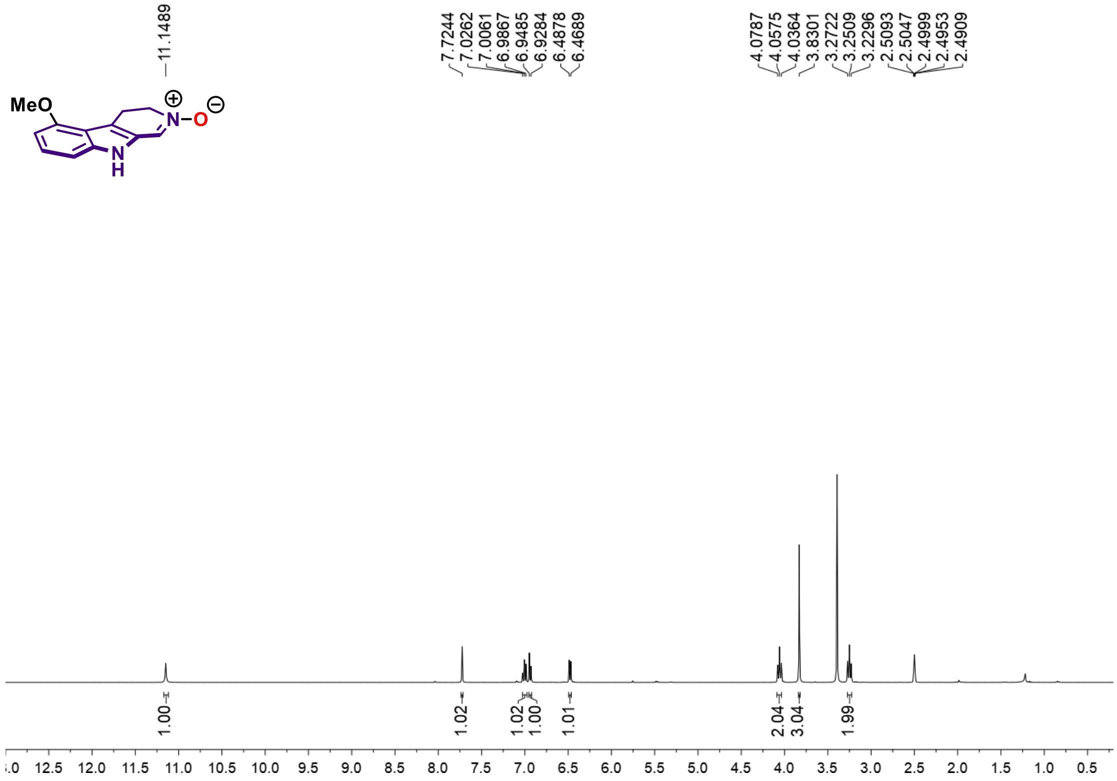

Supplementary Figure 26: ^{13}C NMR of 1f (101 MHz, DMSO-d$_6$)

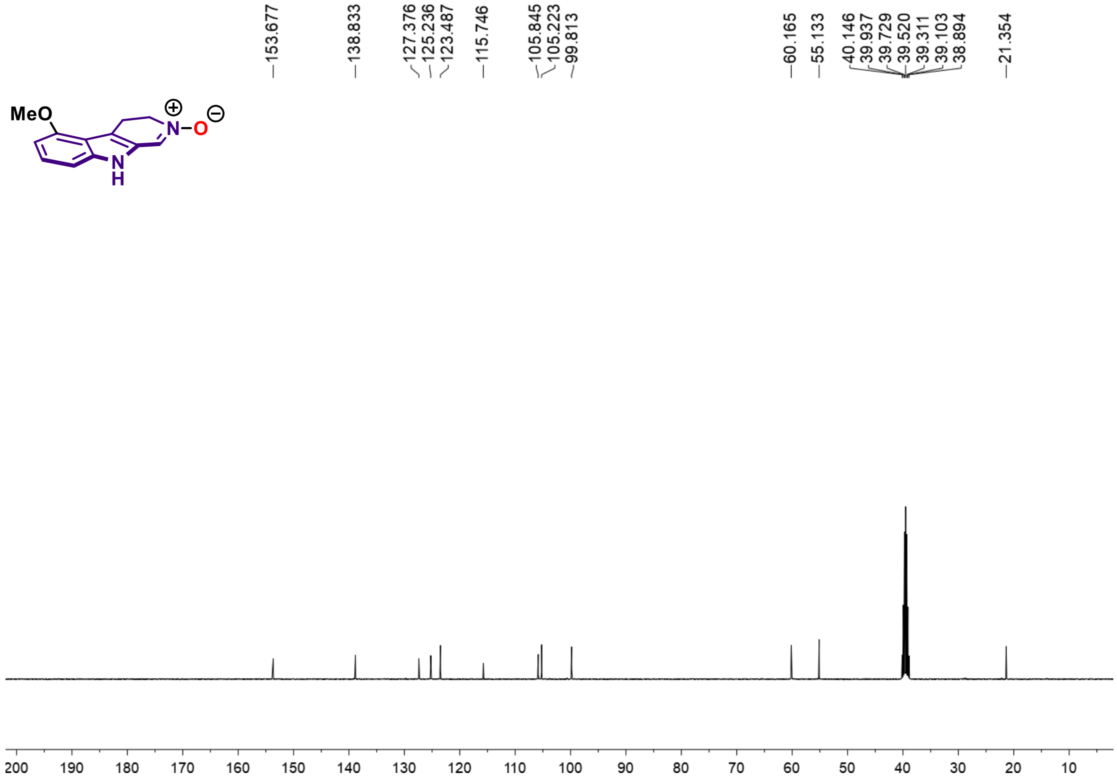

Supplementary Figure 27: ^{1}H NMR of 1g (400 MHz, DMSO-d$_6$)

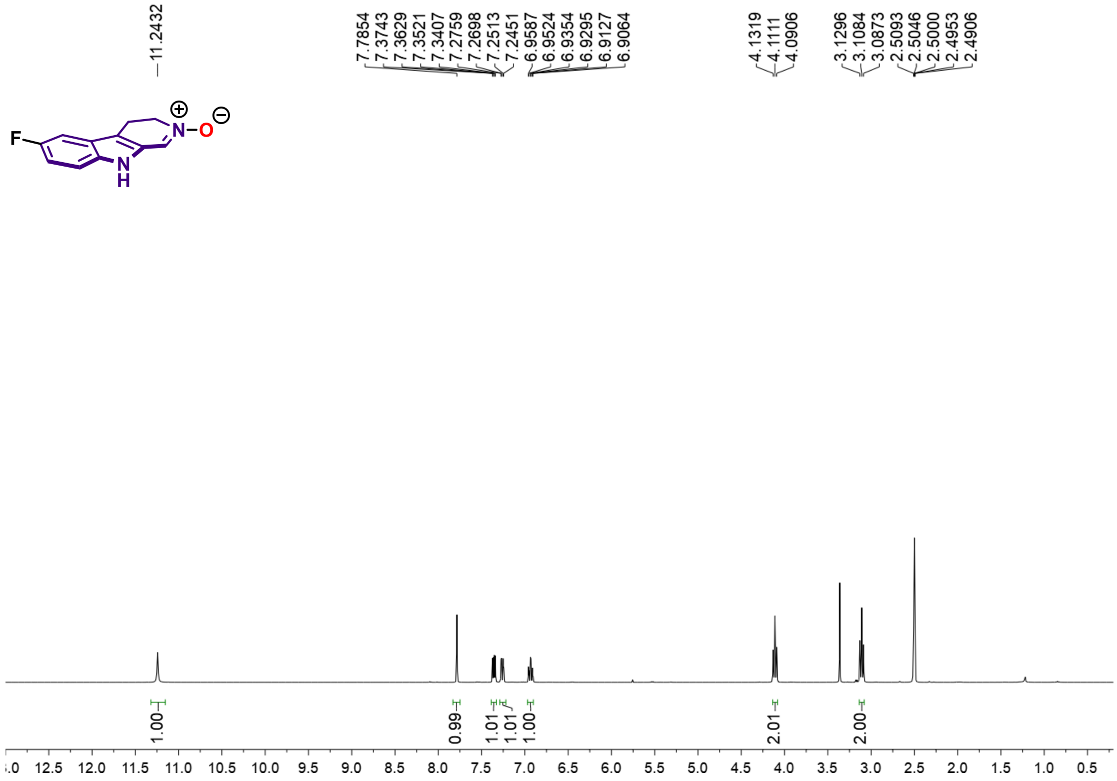

Supplementary Figure 28: ^{13}C NMR of 1g (101 MHz, DMSO-d$_6$)

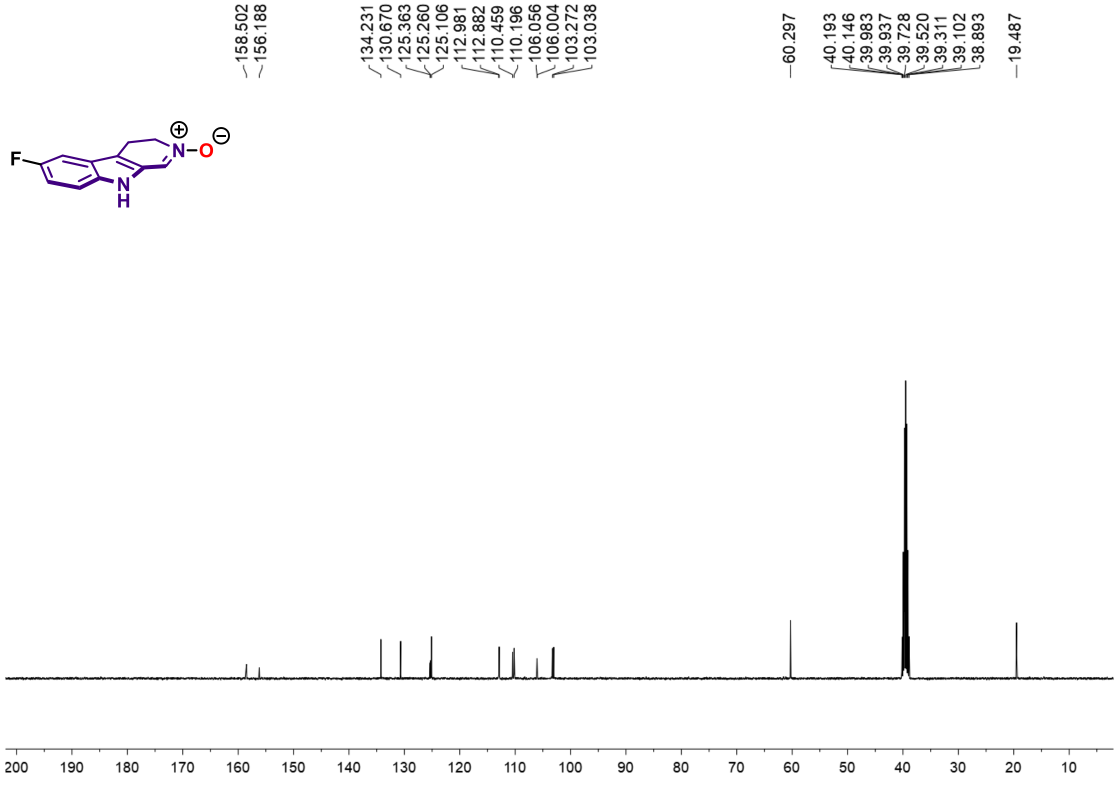

Supplementary Figure 29: ^{1}H NMR of 1h (400 MHz, DMSO-d$_6$)

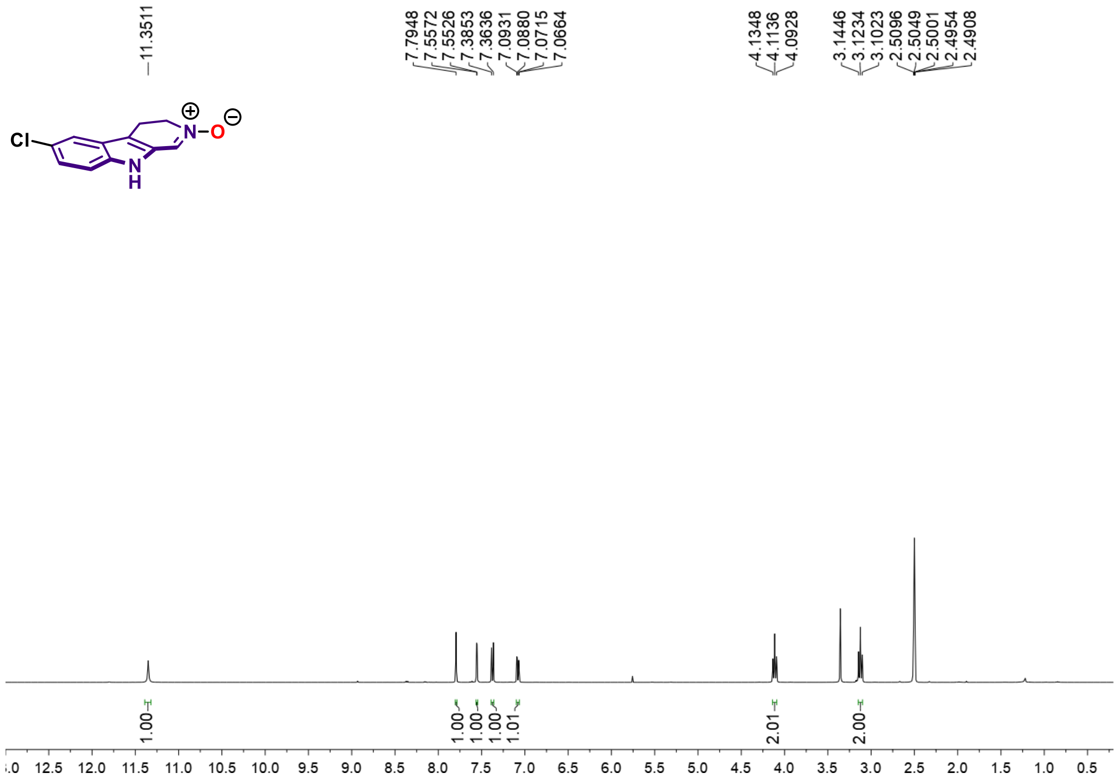

Supplementary Figure 30: ^{13}C NMR of 1h (101 MHz, DMSO-d$_6$)

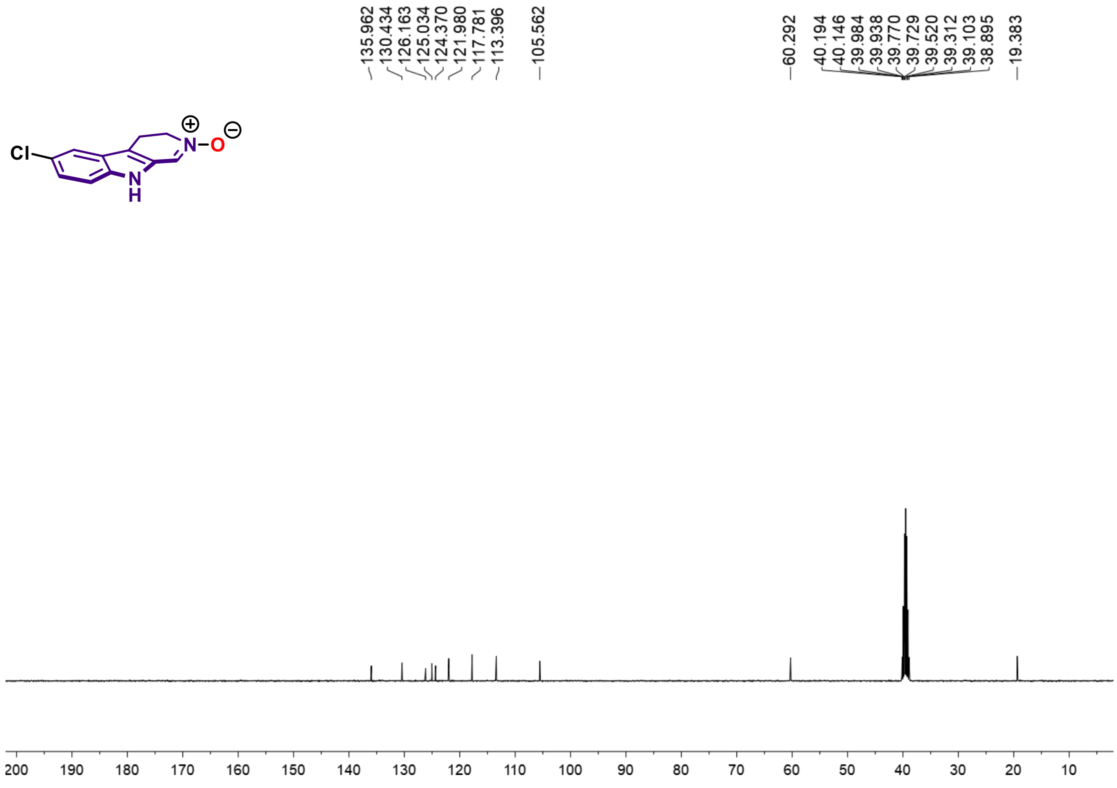

Supplementary Figure 31: ^{1}H NMR of 1i (400 MHz, DMSO-d$_6$)

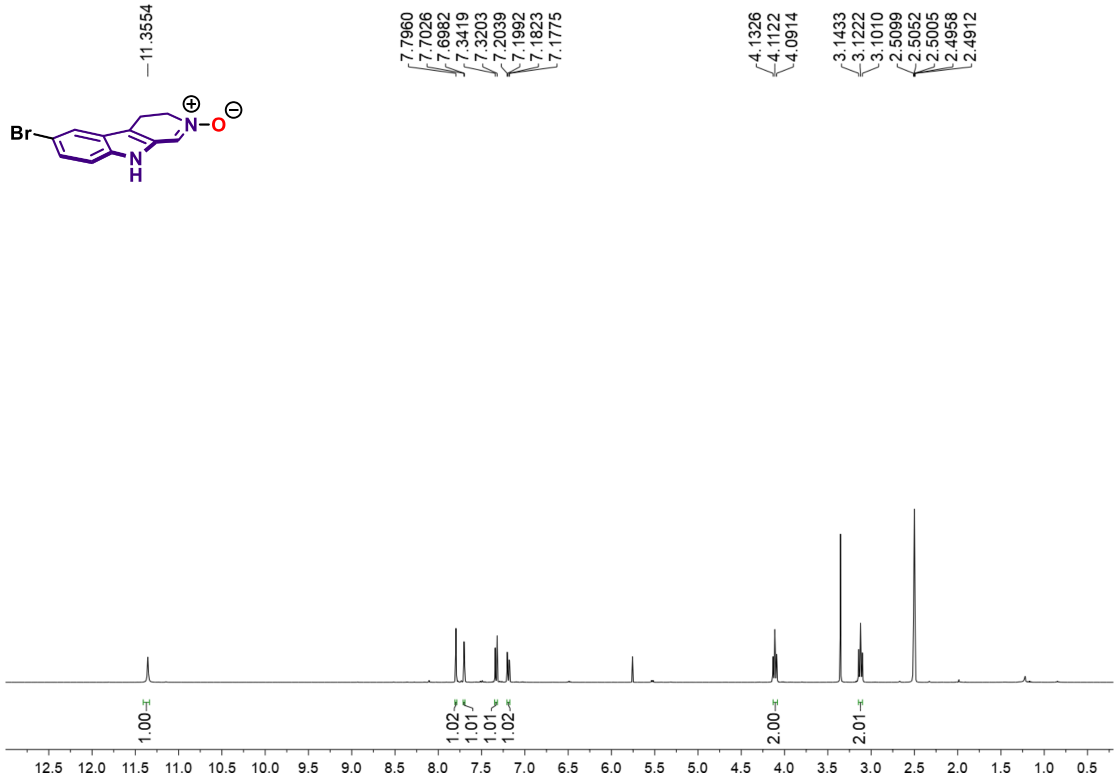

Supplementary Figure 32: ^{13}C NMR of 1i (101 MHz, DMSO-d$_6$)

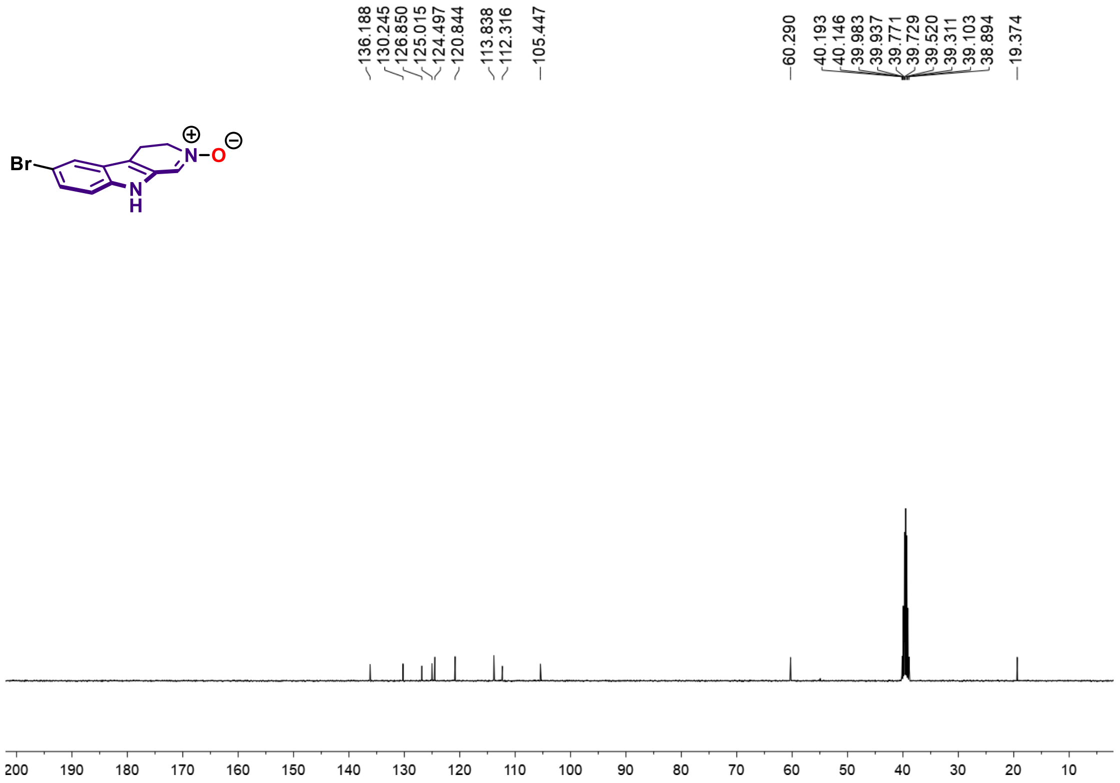

Supplementary Figure 33: ^{1}H NMR of 1j (400 MHz, DMSO-d$_6$)

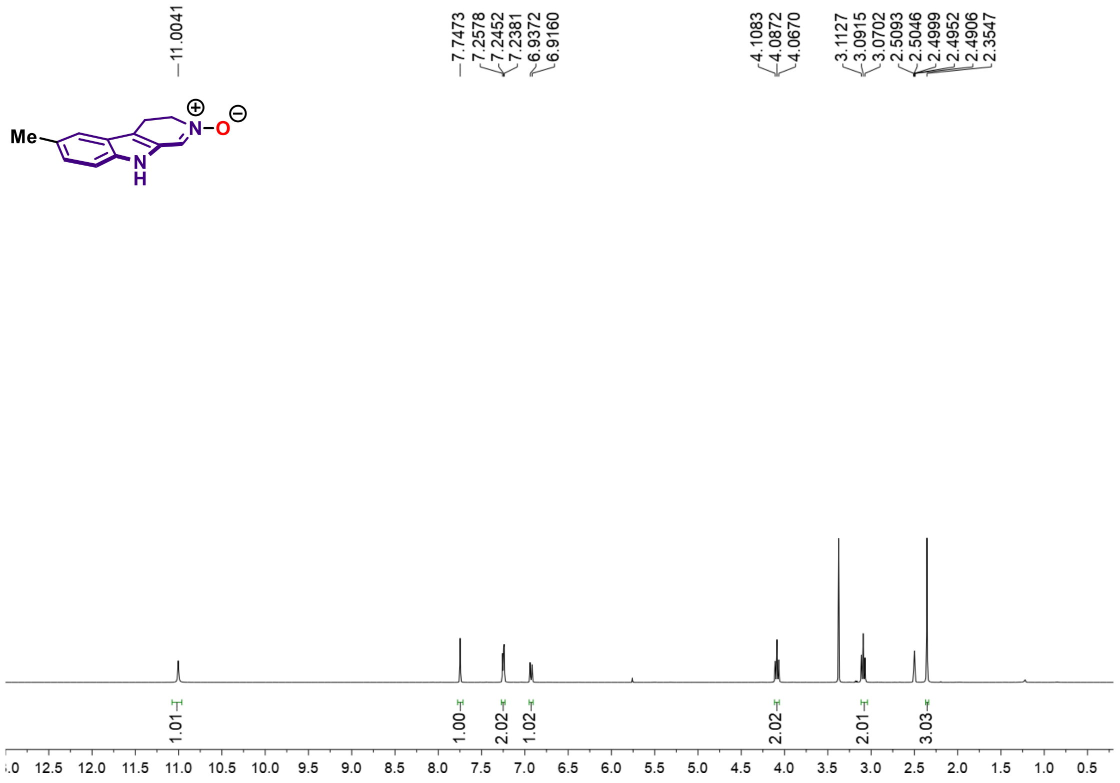

Supplementary Figure 34: ^{13}C NMR of 1j (101 MHz, DMSO-d$_6$)

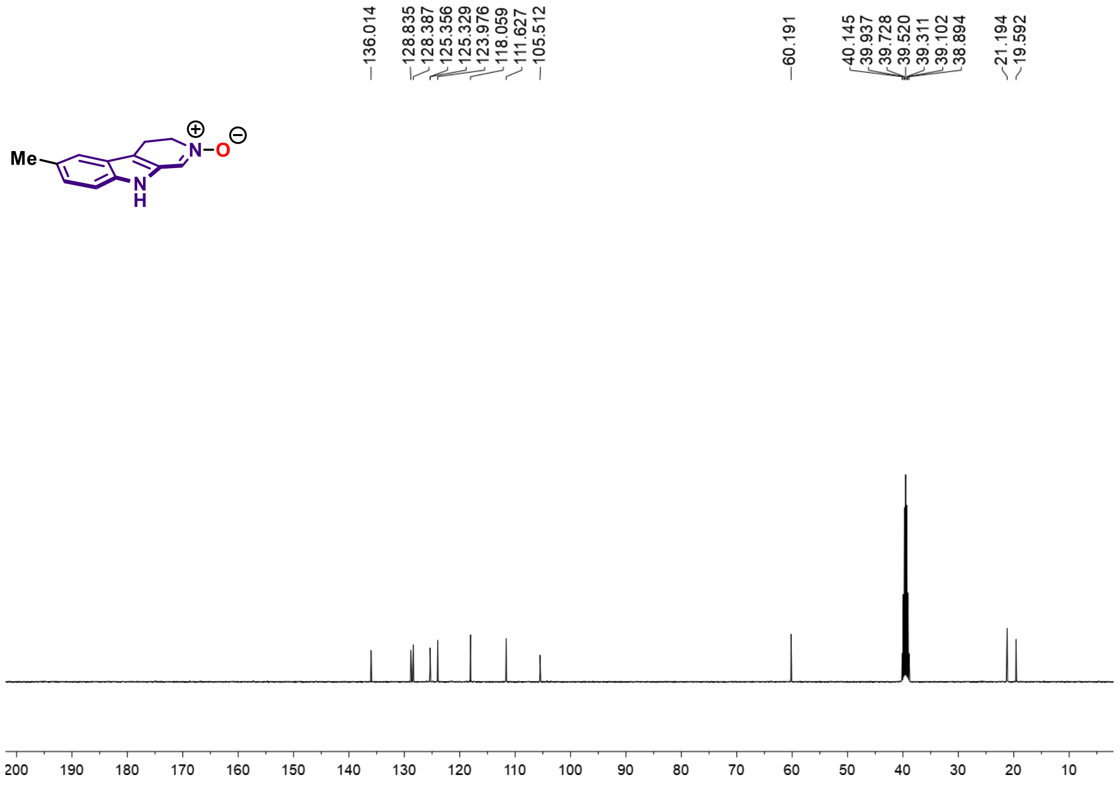

Supplementary Figure 35: ¹H NMR of 1k (400 MHz, DMSO-d₆)

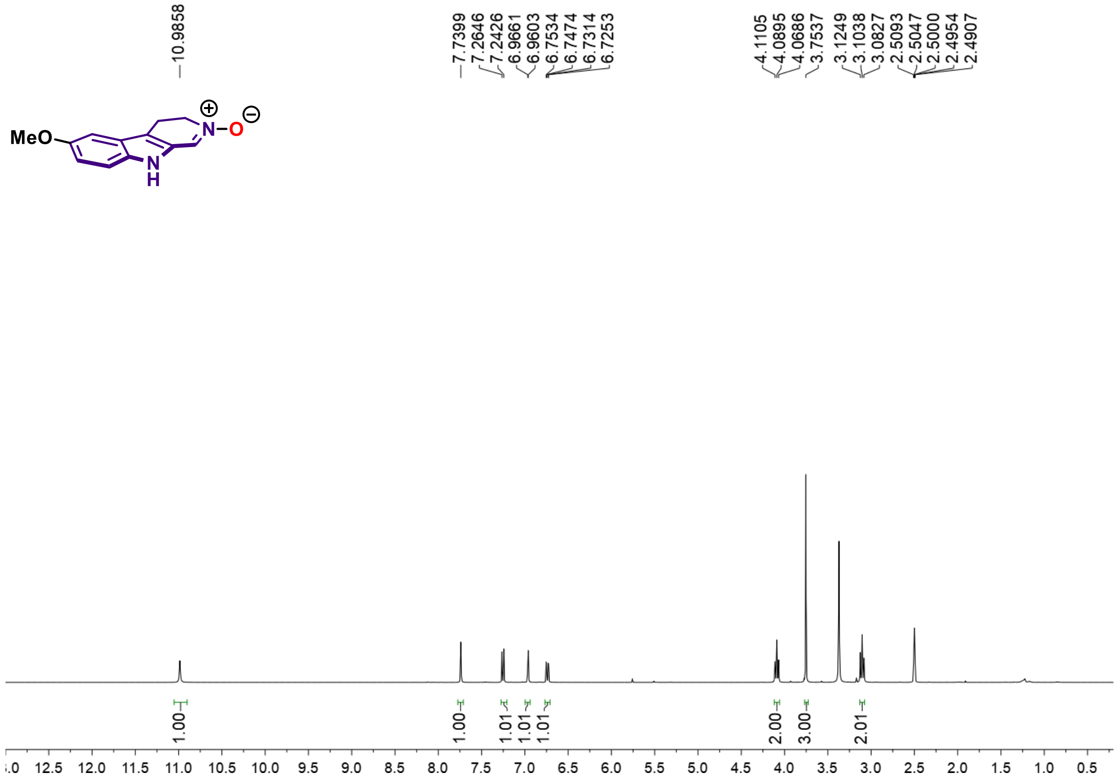

Supplementary Figure 36: ¹³C NMR of 1k (101 MHz, DMSO-d₆)

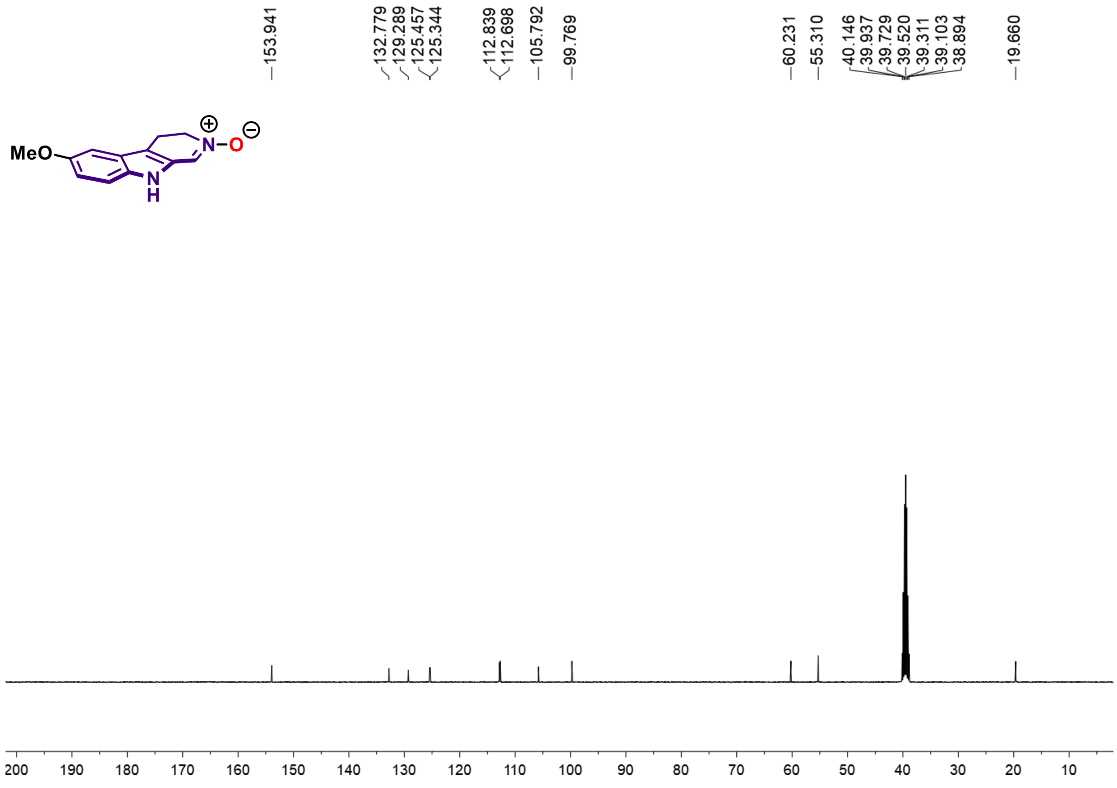

Supplementary Figure 37: ^{1}H NMR of 1l (400 MHz, DMSO-d$_6$)

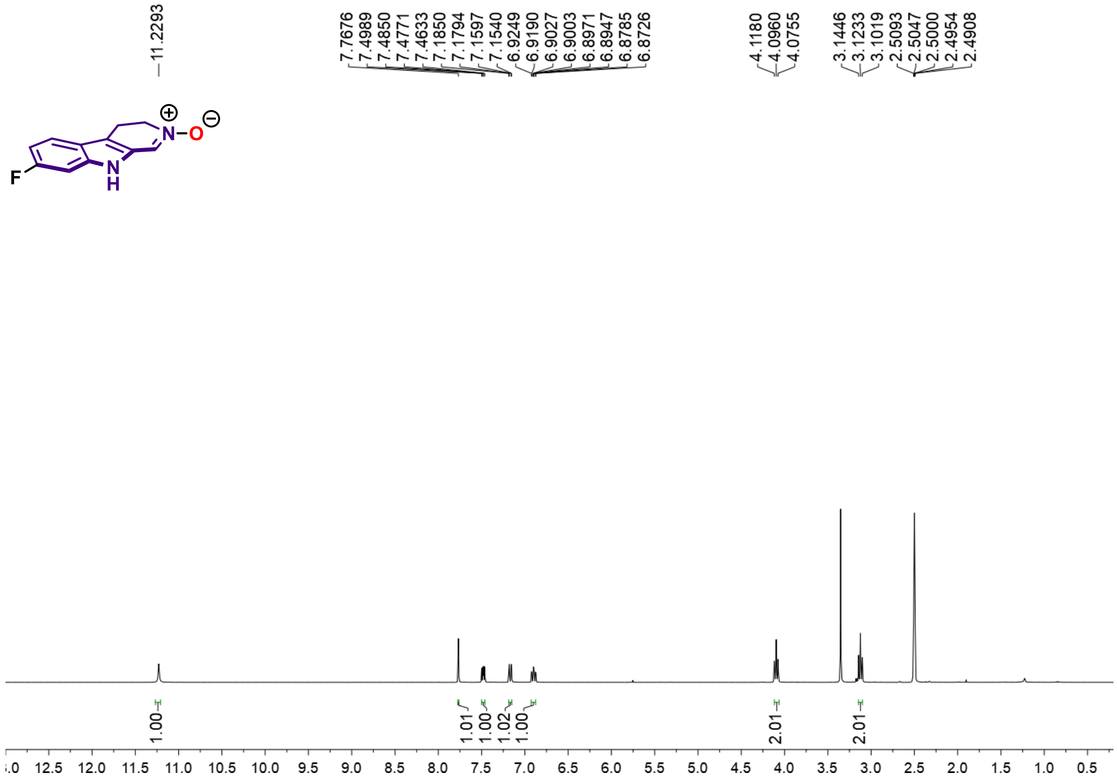

Supplementary Figure 38: ^{13}C NMR of 1l (101 MHz, DMSO-d$_6$)

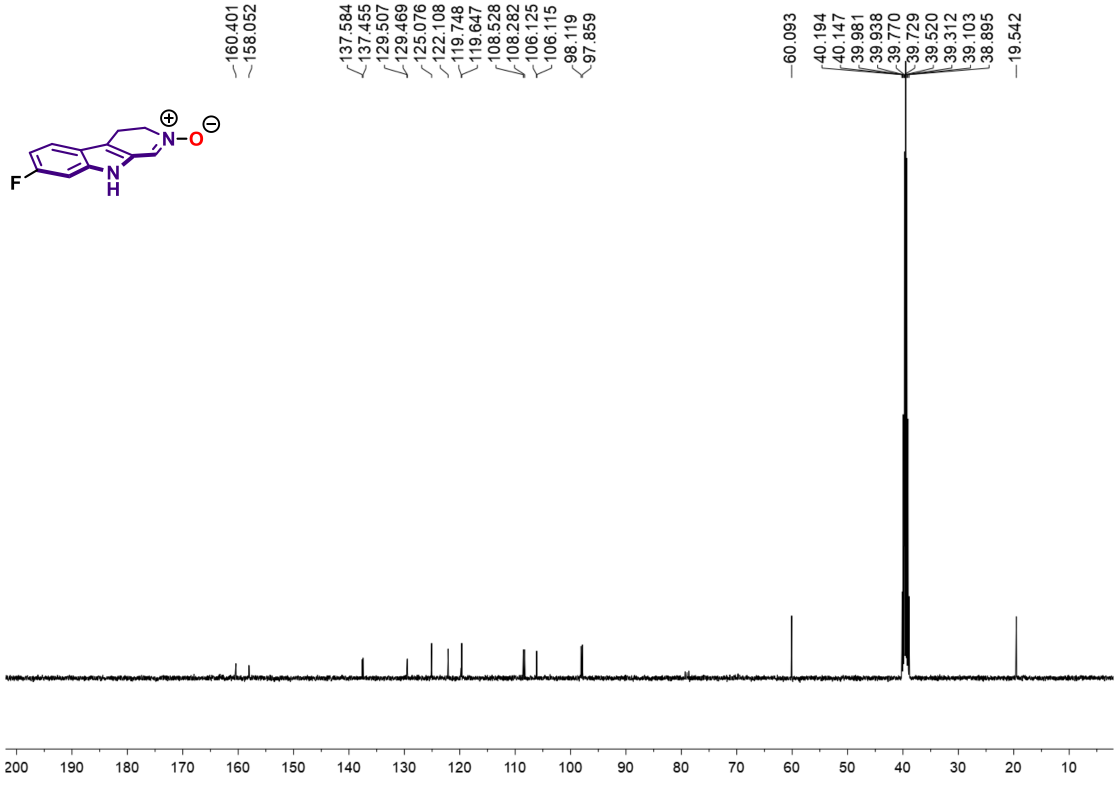

Supplementary Figure 39: ^{1}H NMR of 1m (400 MHz, DMSO-d$_6$)

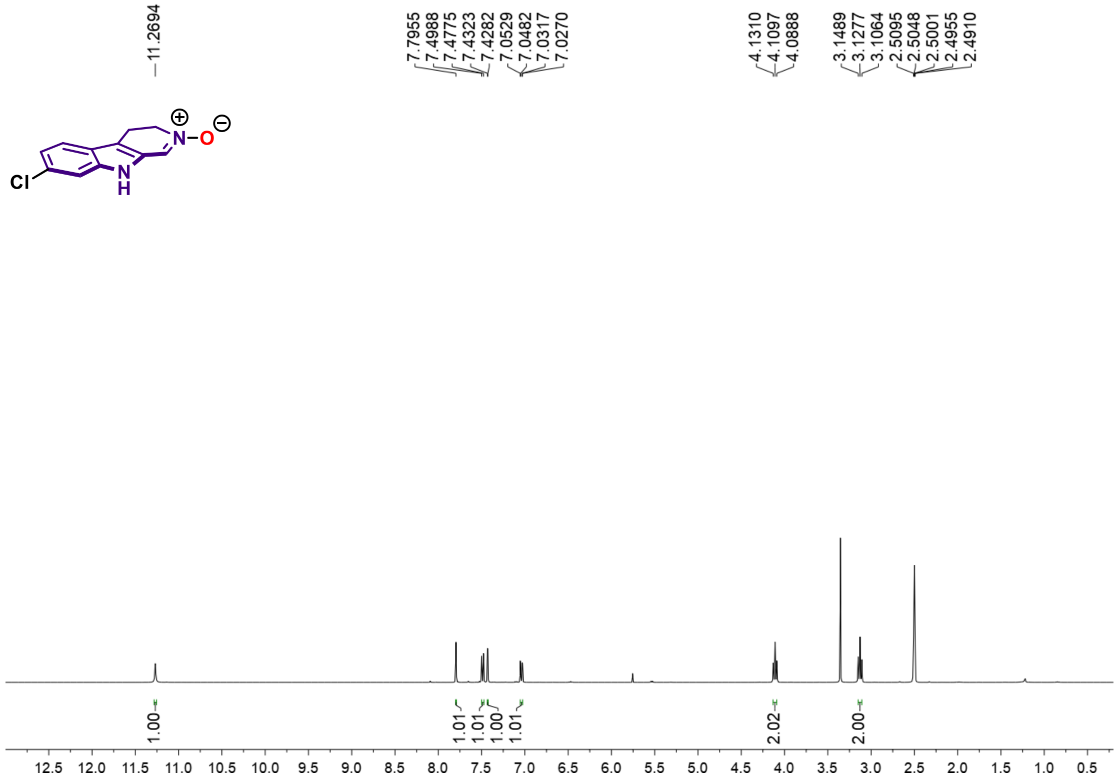

Supplementary Figure 40: ^{13}C NMR of 1m (101 MHz, DMSO-d$_6$)

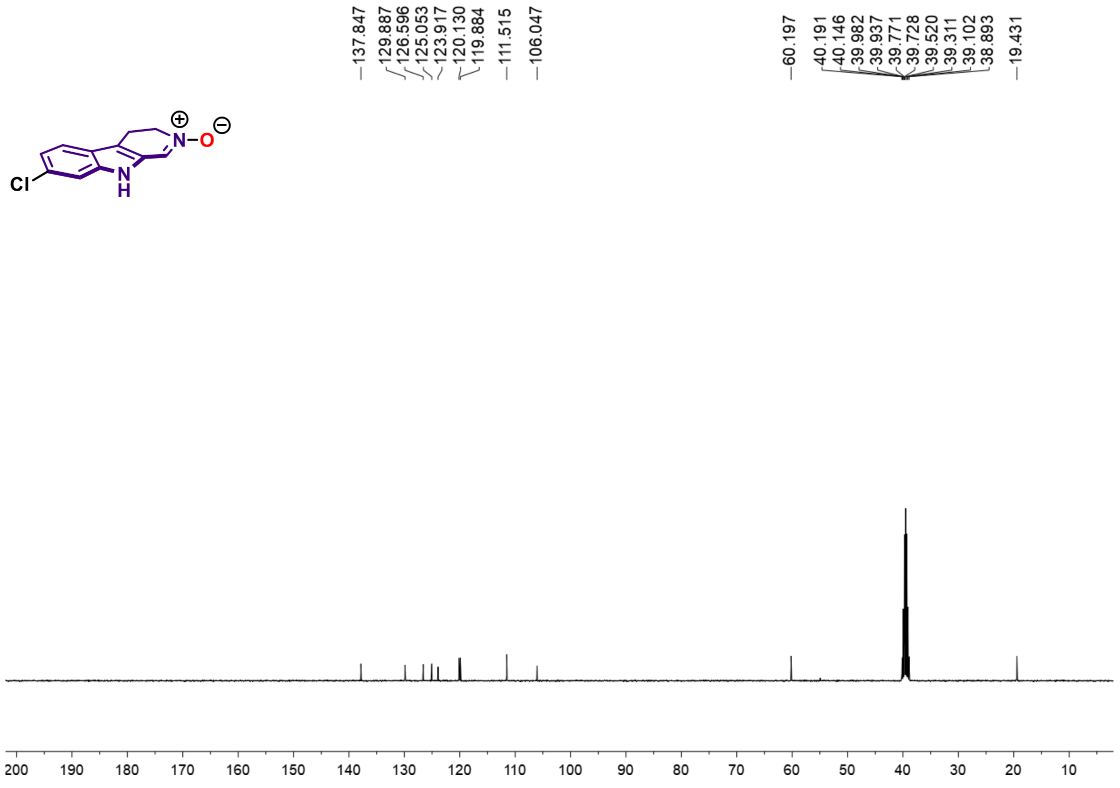

Supplementary Figure 41: ^{1}H NMR of 1n (400 MHz, DMSO-d_6)

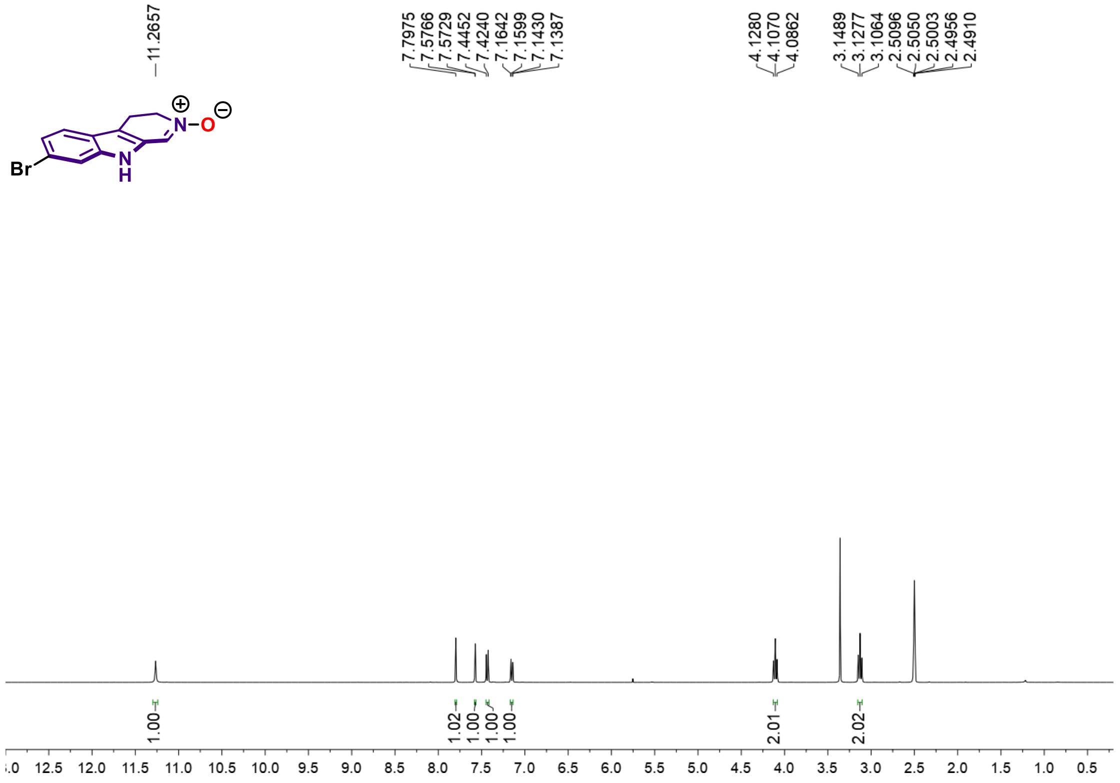

Supplementary Figure 42: ^{13}C NMR of 1n (101 MHz, DMSO-d_6)

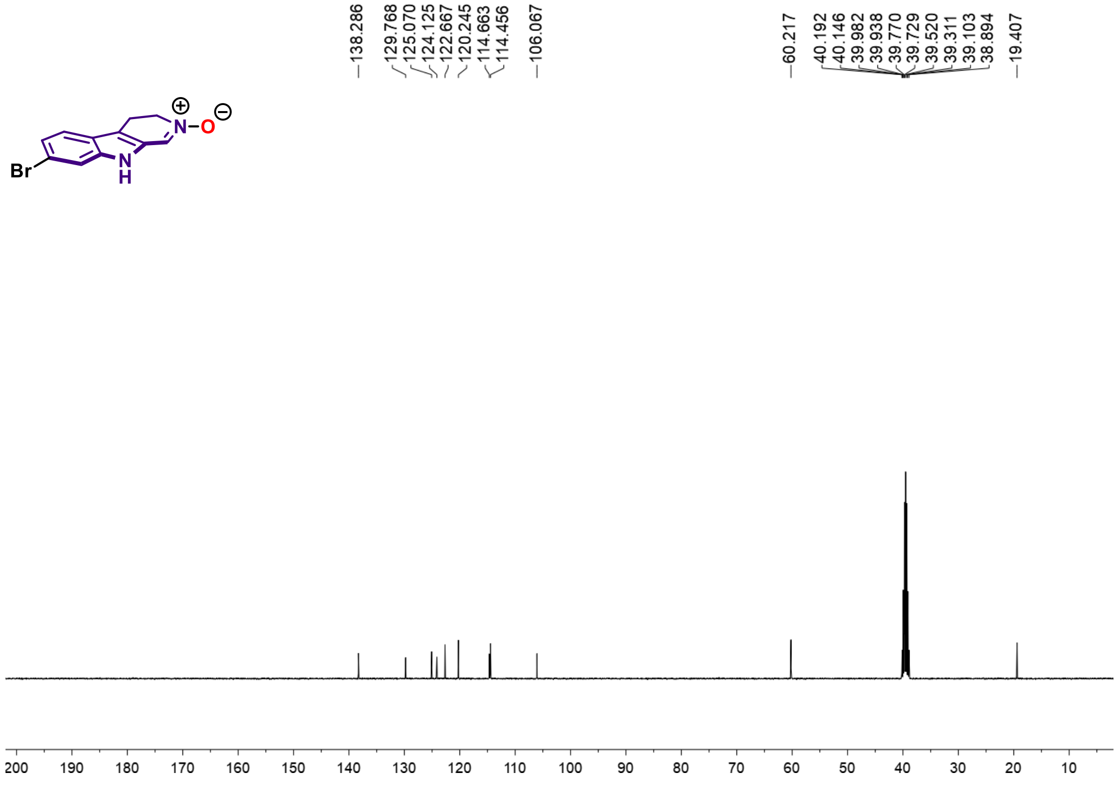

Supplementary Figure 43: ^{1}H NMR of 1o (400 MHz, DMSO-d$_6$)

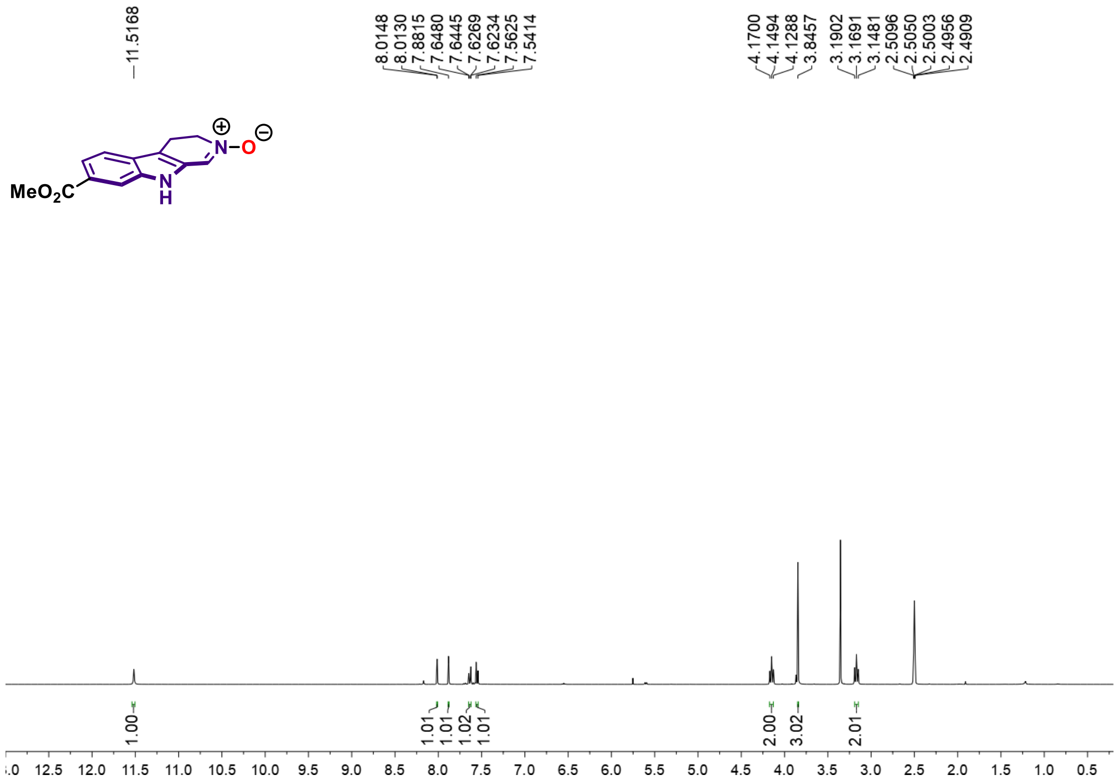

Supplementary Figure 44: ^{13}C NMR of 1o (101 MHz, DMSO-d$_6$)

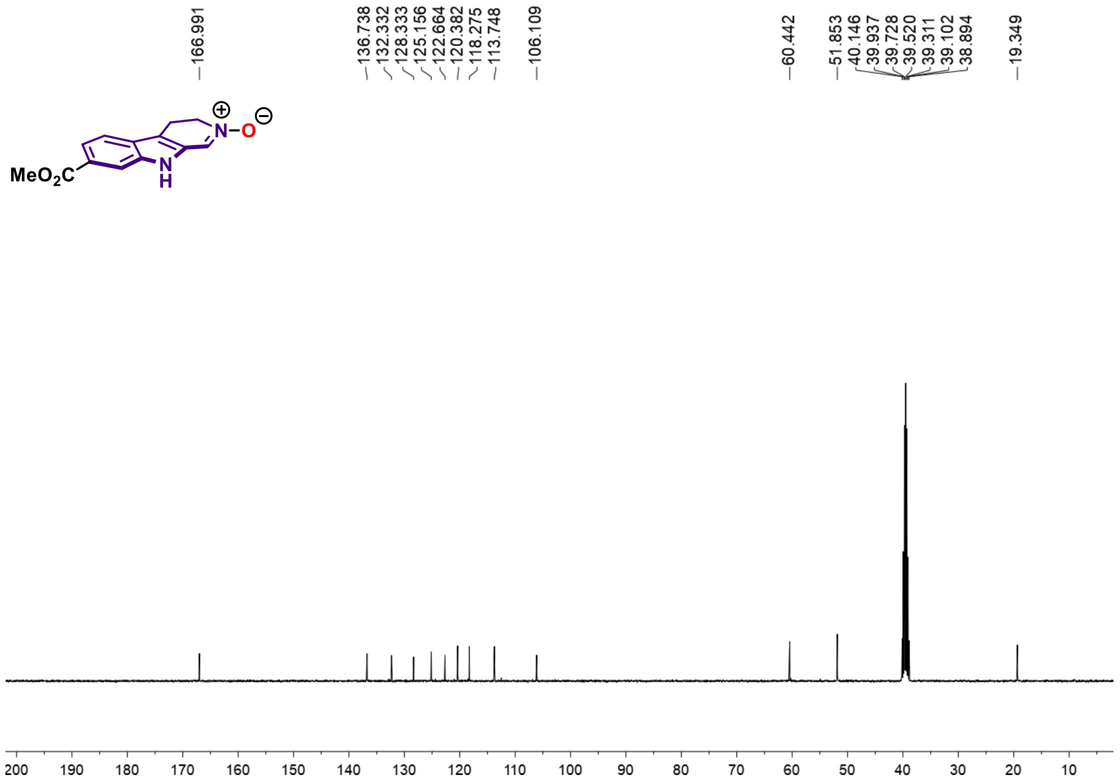

Supplementary Figure 45: ^{1}H NMR of 1p (400 MHz, DMSO-d$_6$)

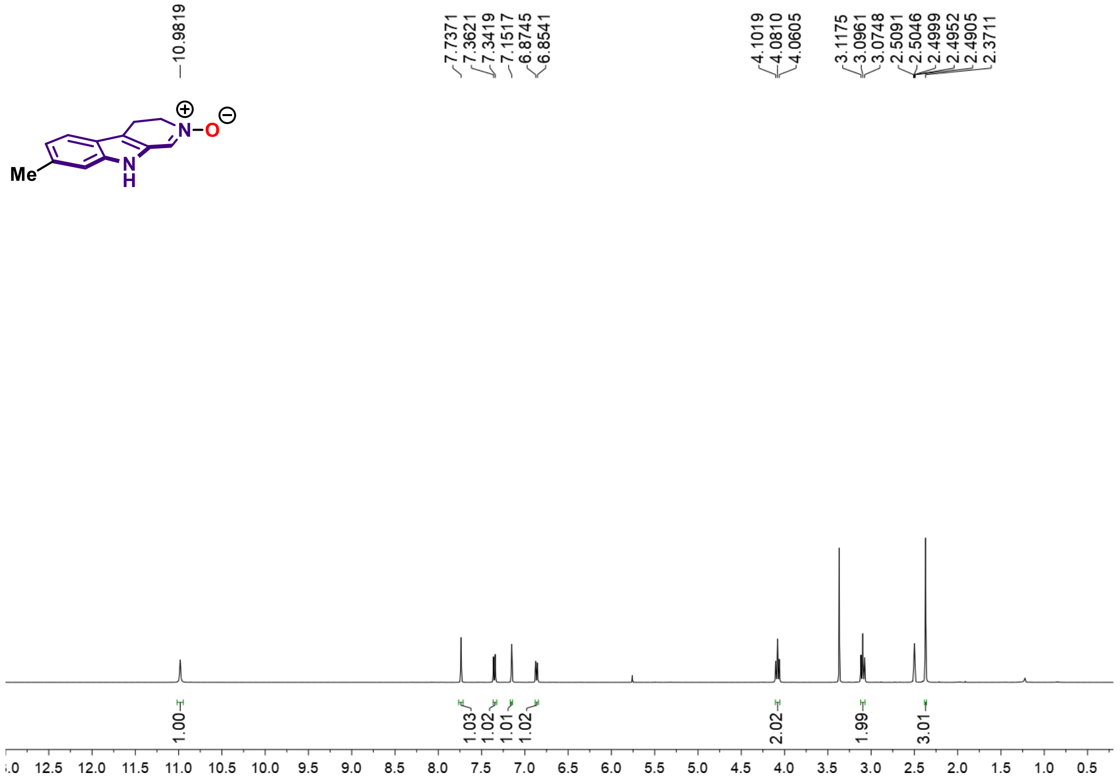

Supplementary Figure 46: ^{13}C NMR of 1p (101 MHz, DMSO-d$_6$)

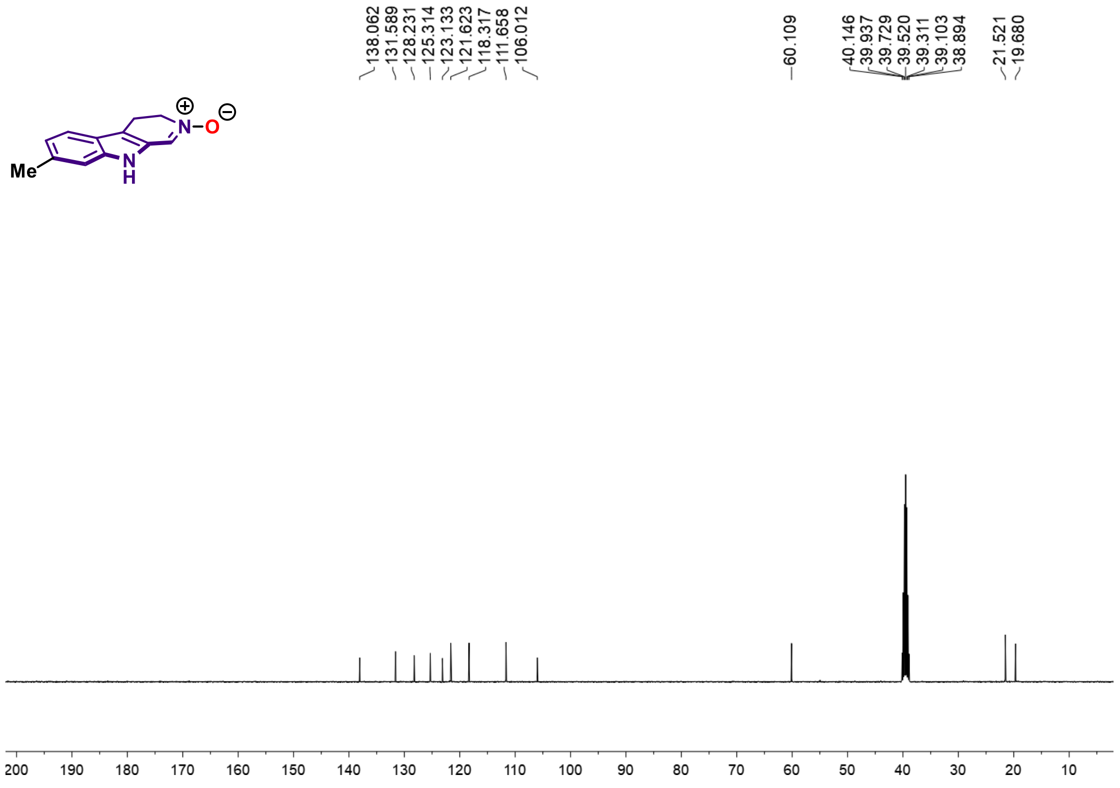

Supplementary Figure 47: ^{1}H NMR of 1q (400 MHz, DMSO-d$_6$)

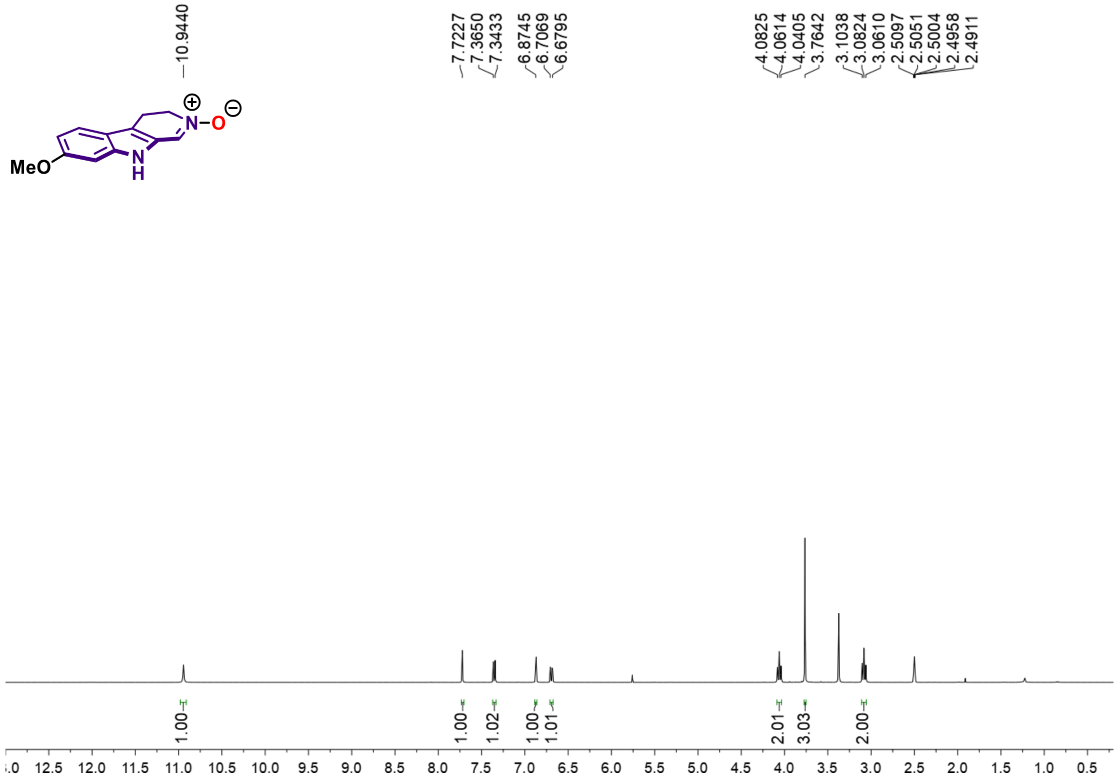

Supplementary Figure 48: ^{13}C NMR of 1q (101 MHz, DMSO-d$_6$)

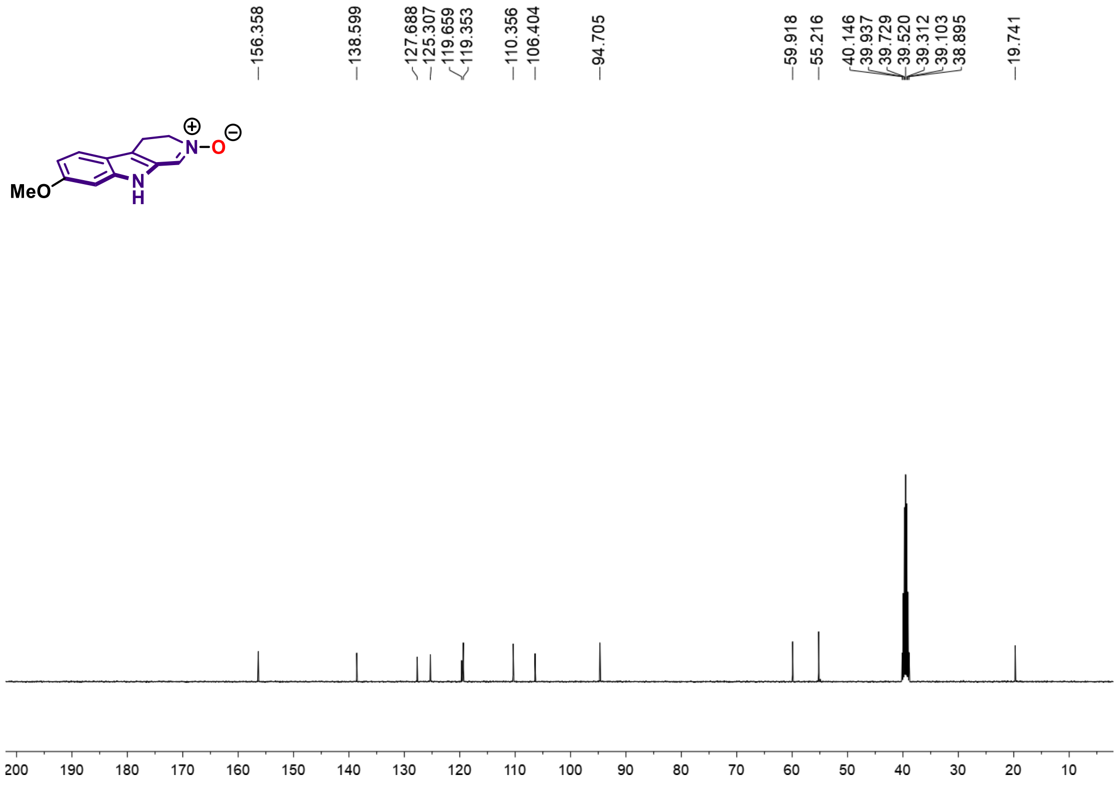

Supplementary Figure 49: ^{1}H NMR of 1r (400 MHz, DMSO-d$_6$)

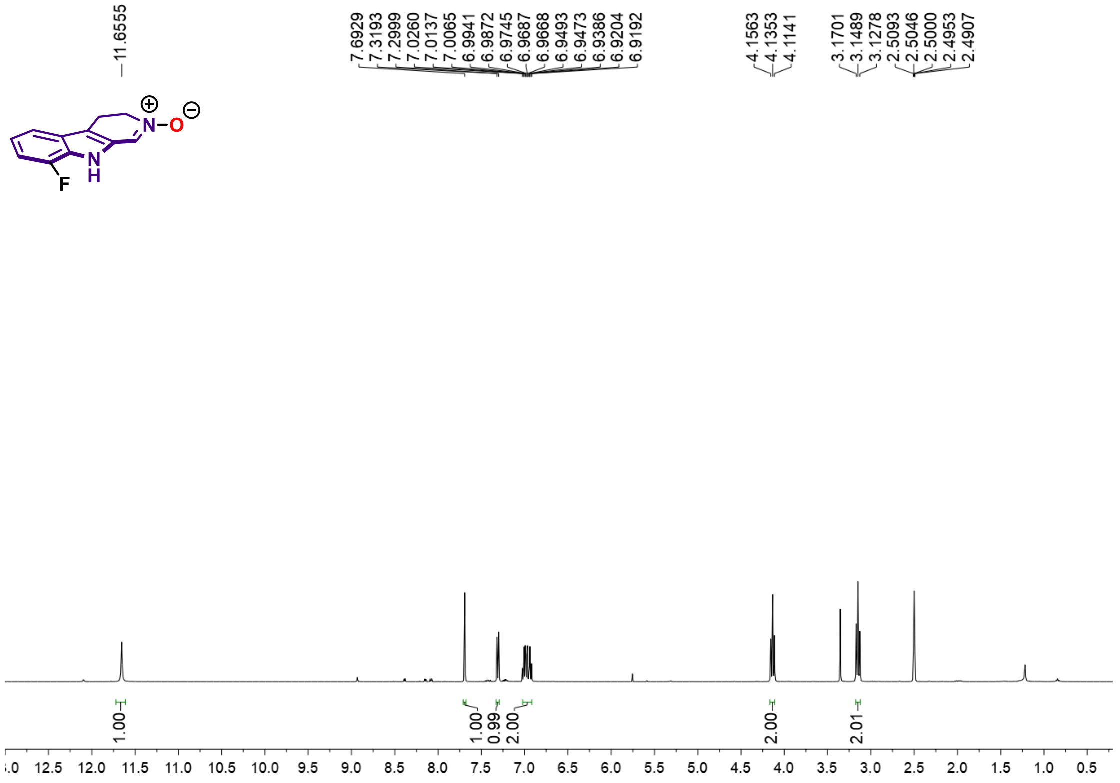

Supplementary Figure 50: ^{13}C NMR of 1r (101 MHz, DMSO-d$_6$)

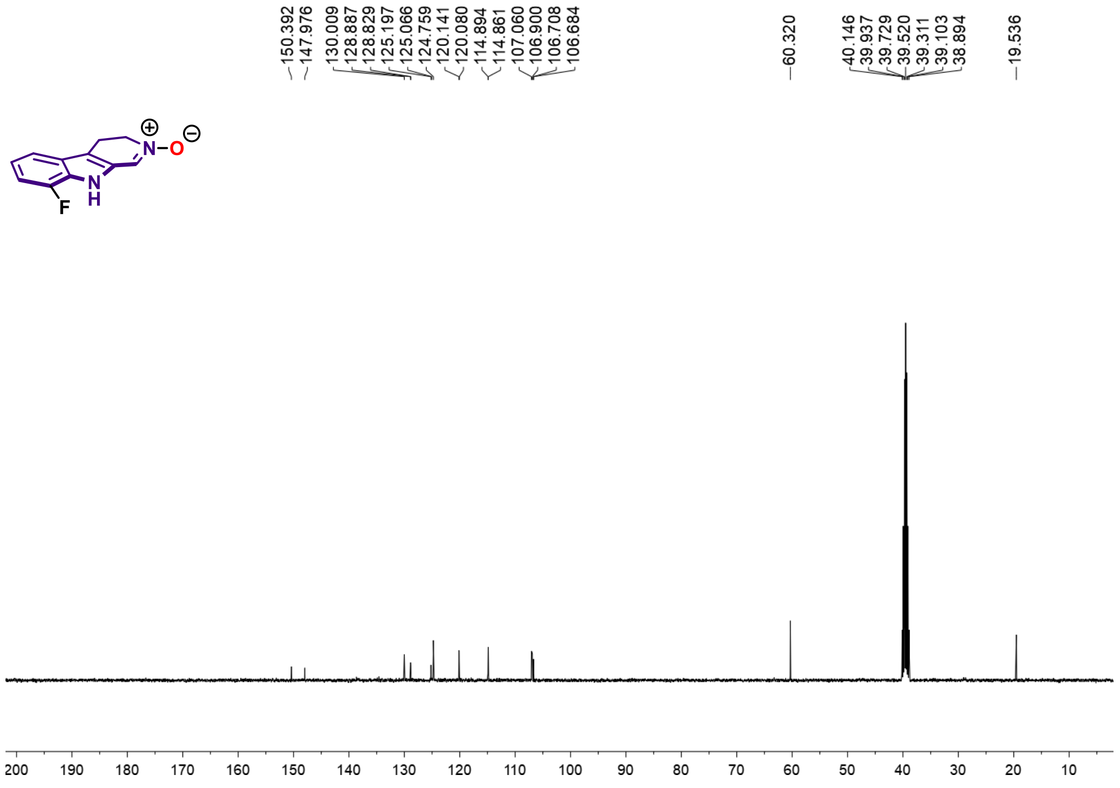

Supplementary Figure 51: ^{1}H NMR of 1s (400 MHz, DMSO-d$_6$)

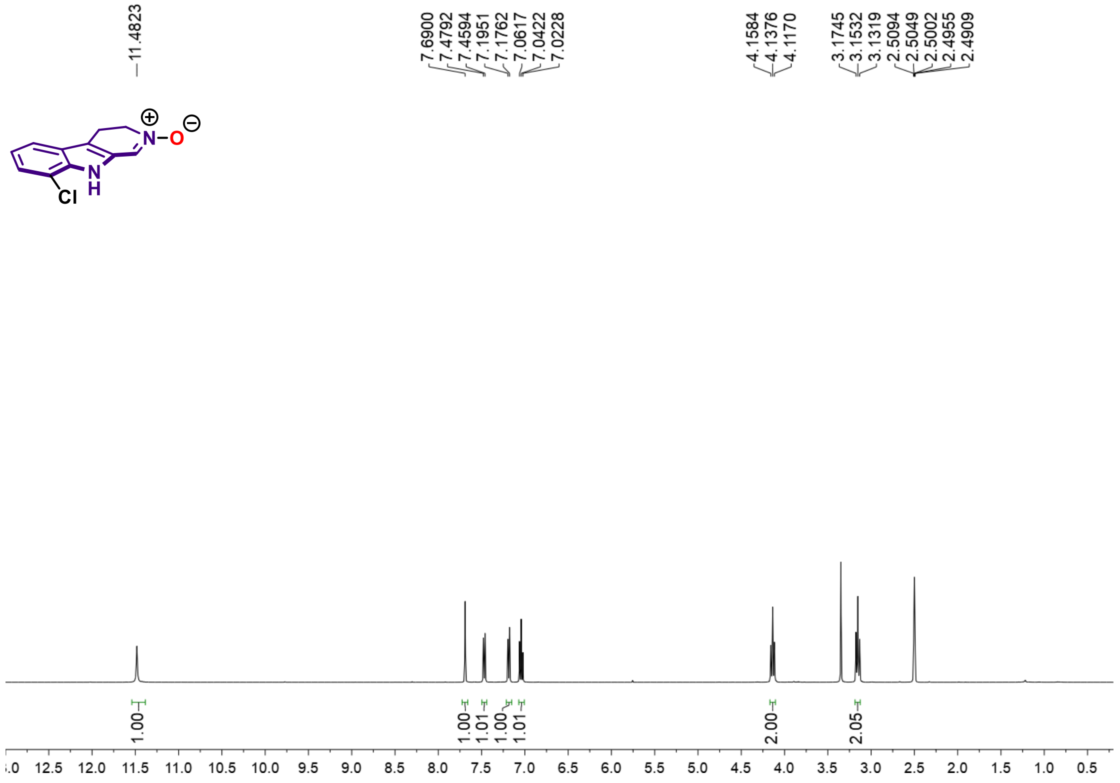

Supplementary Figure 52: ^{13}C NMR of 1s (101 MHz, DMSO-d$_6$)

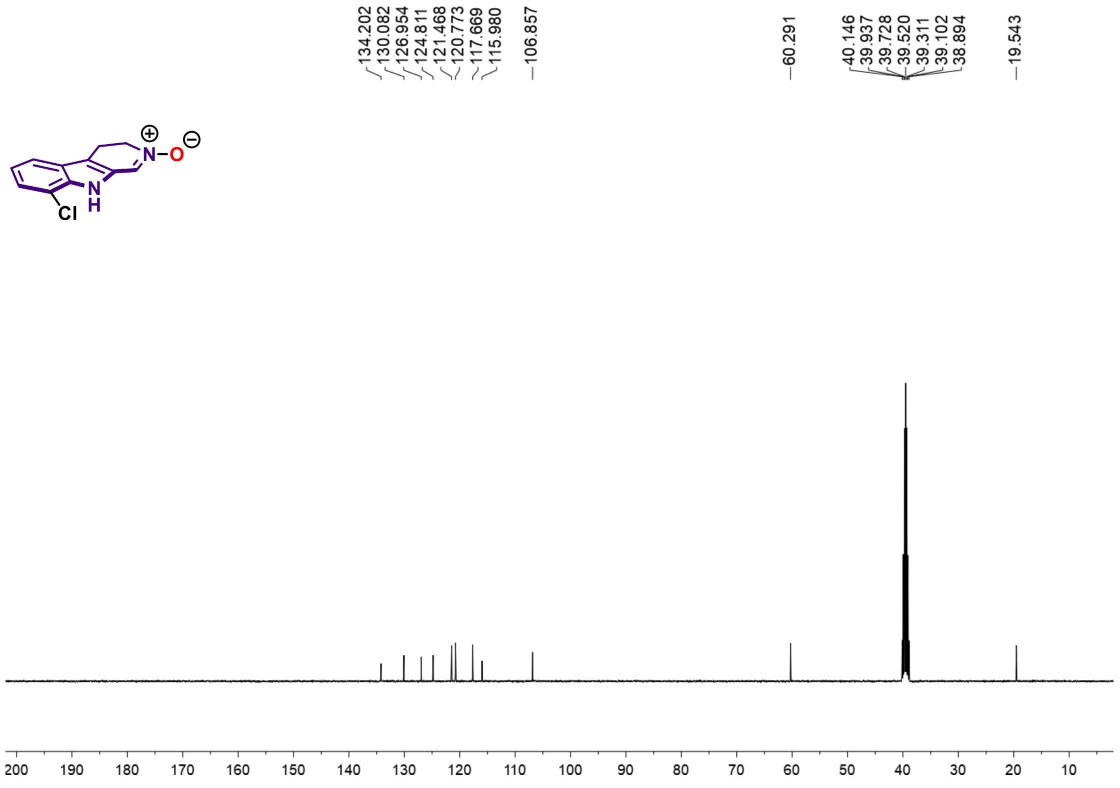

Supplementary Figure 53: ^{1}H NMR of 1t (400 MHz, DMSO-d$_6$)

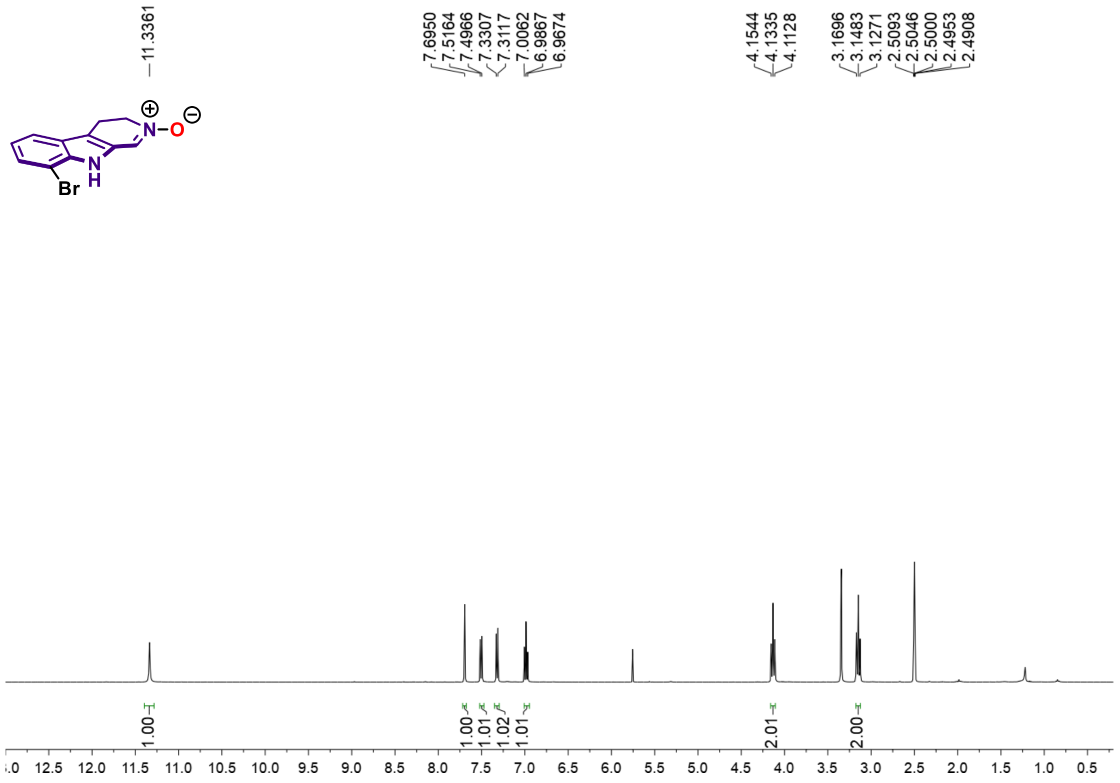

Supplementary Figure 54: ^{13}C NMR of 1t (101 MHz, DMSO-d$_6$)

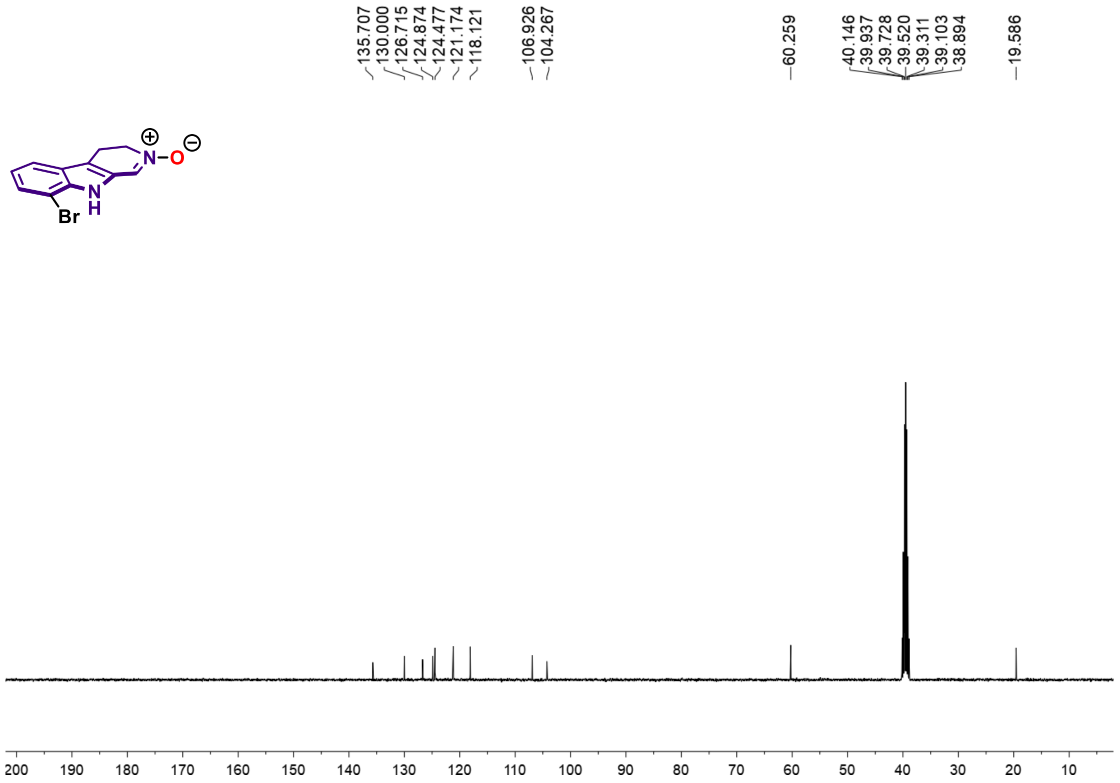

Supplementary Figure 55: ^{1}H NMR of 1u (400 MHz, DMSO-d6)

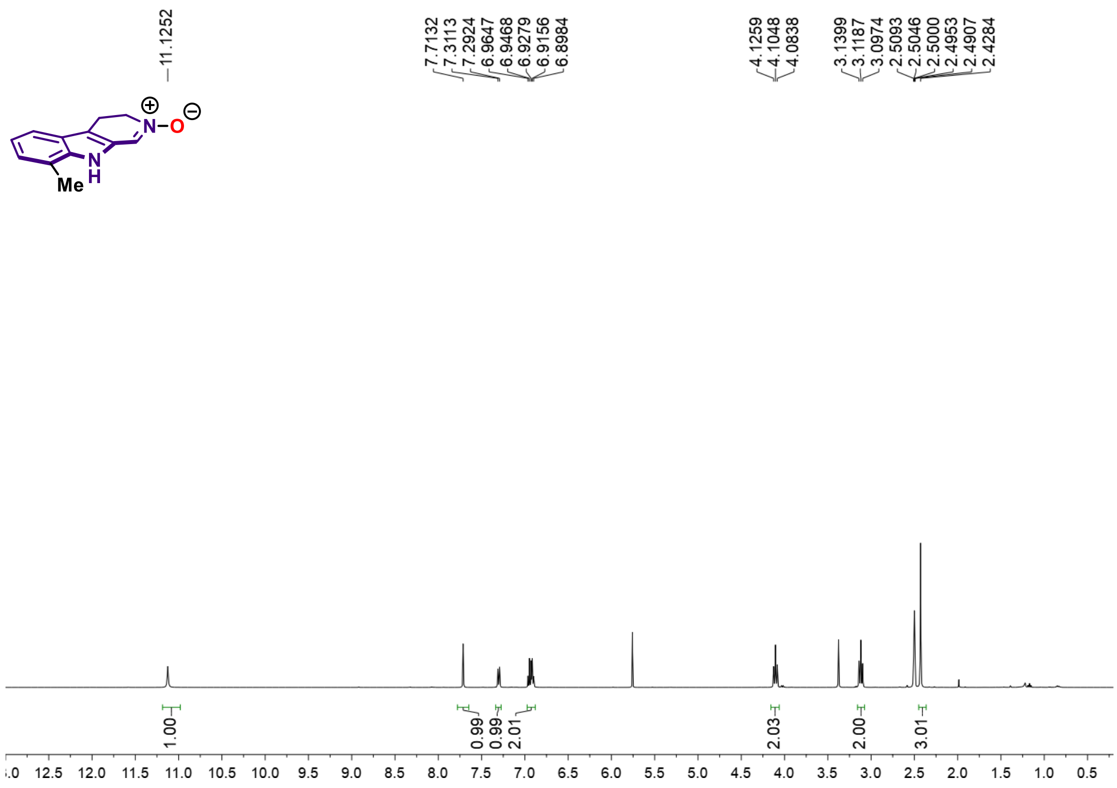

Supplementary Figure 56: ^{13}C NMR of 1u (101 MHz, DMSO-d6)

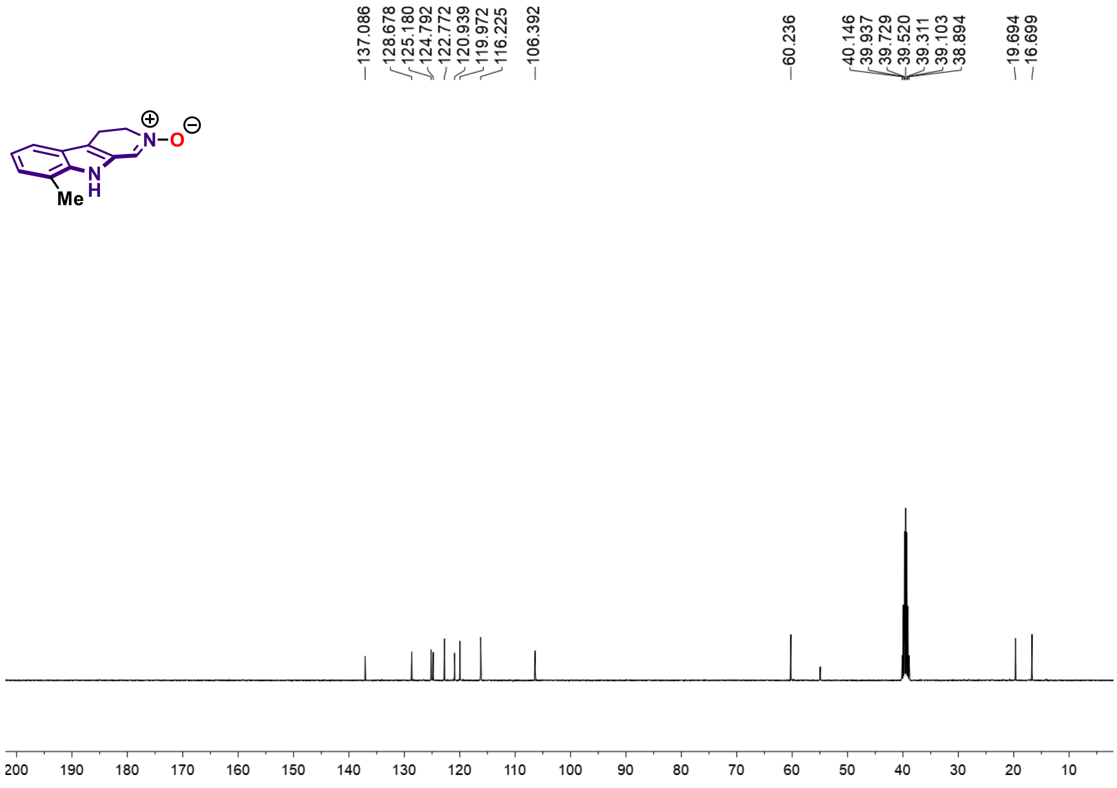

Supplementary Figure 57: ^{1}H NMR of 1v (400 MHz, DMSO-d$_6$)

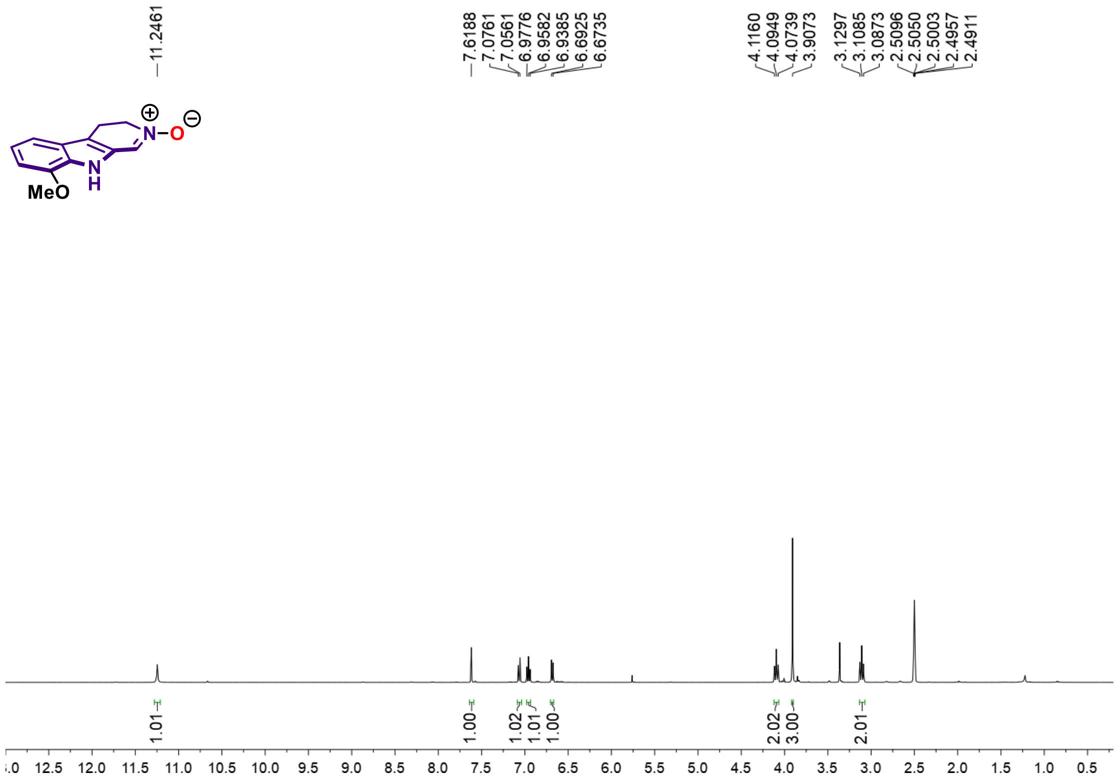

Supplementary Figure 58: ^{13}C NMR of 1v (101 MHz, DMSO-d$_6$)

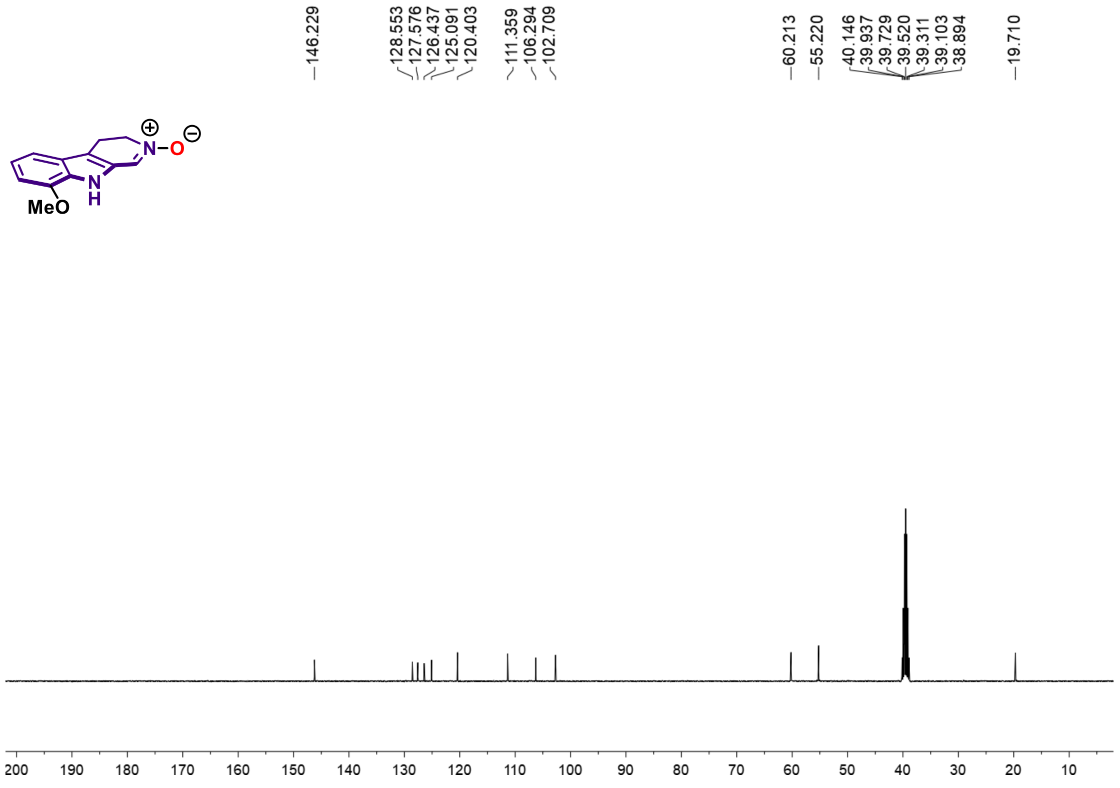

Supplementary Figure 59: ^{1}H NMR of 1w (400 MHz, DMSO-d6)

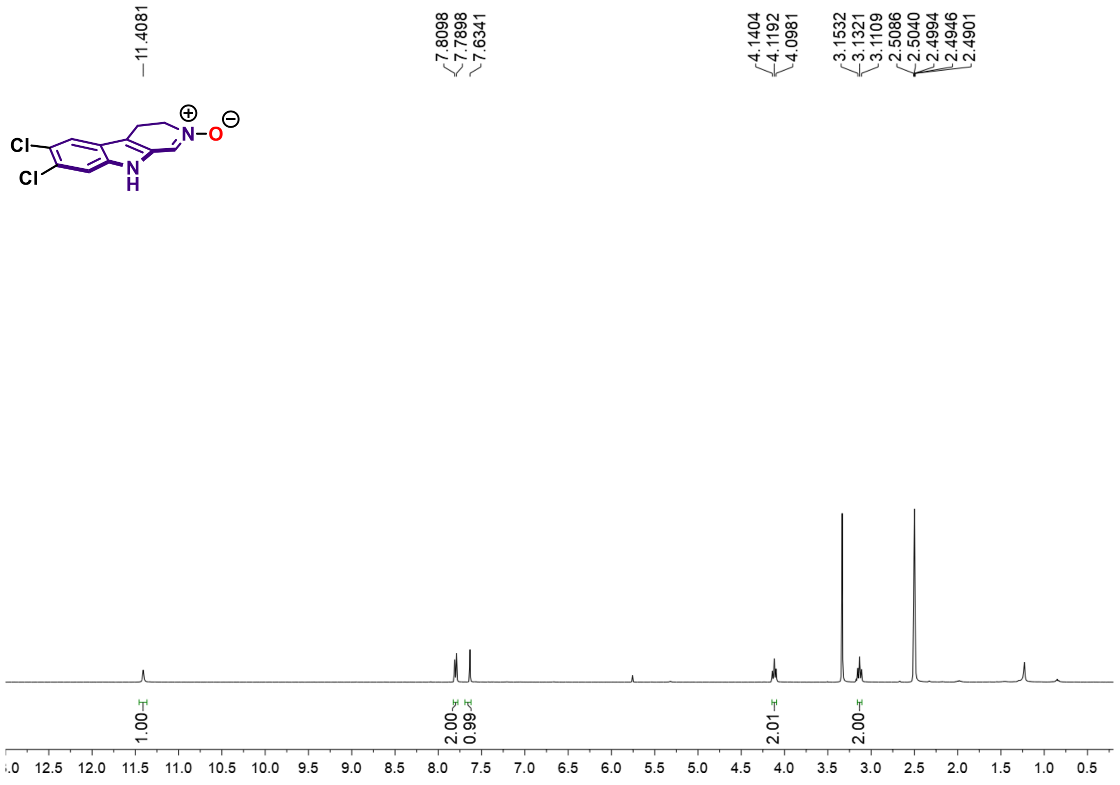

Supplementary Figure 60: ^{13}C NMR of 1w (101 MHz, DMSO-d6)

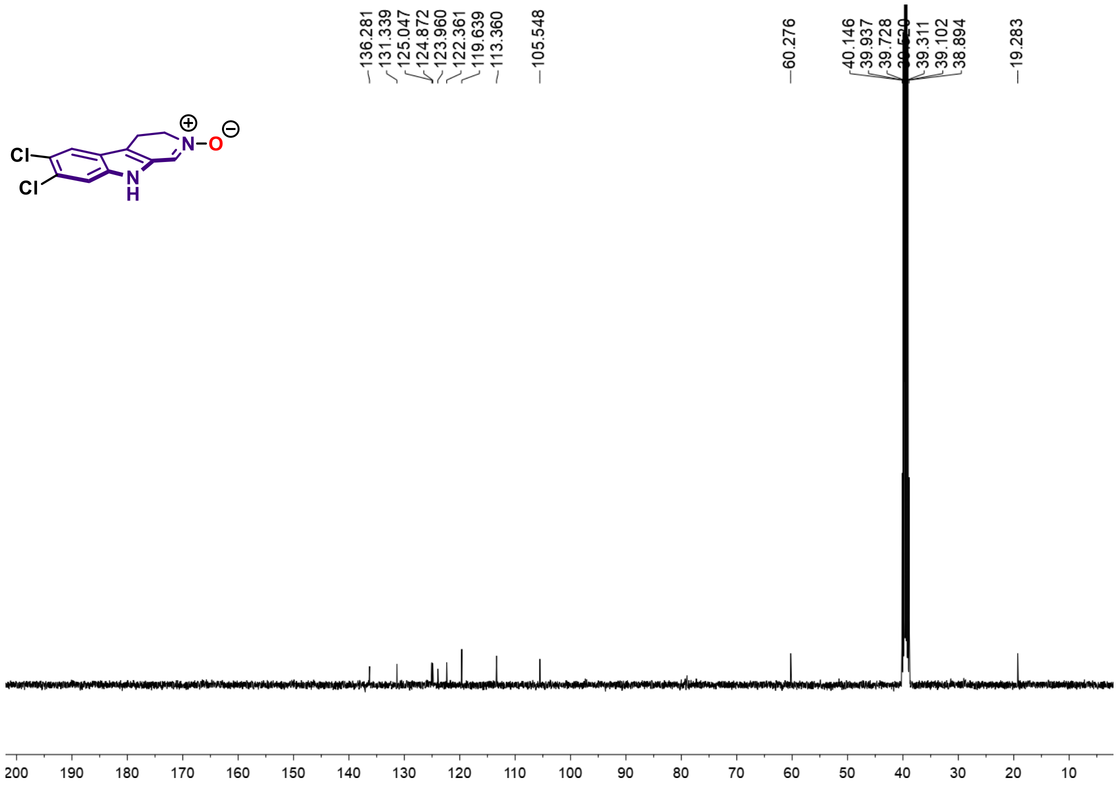

Supplementary Figure 61: ^{1}H NMR of 6a (400 MHz, $CDCl_3$)

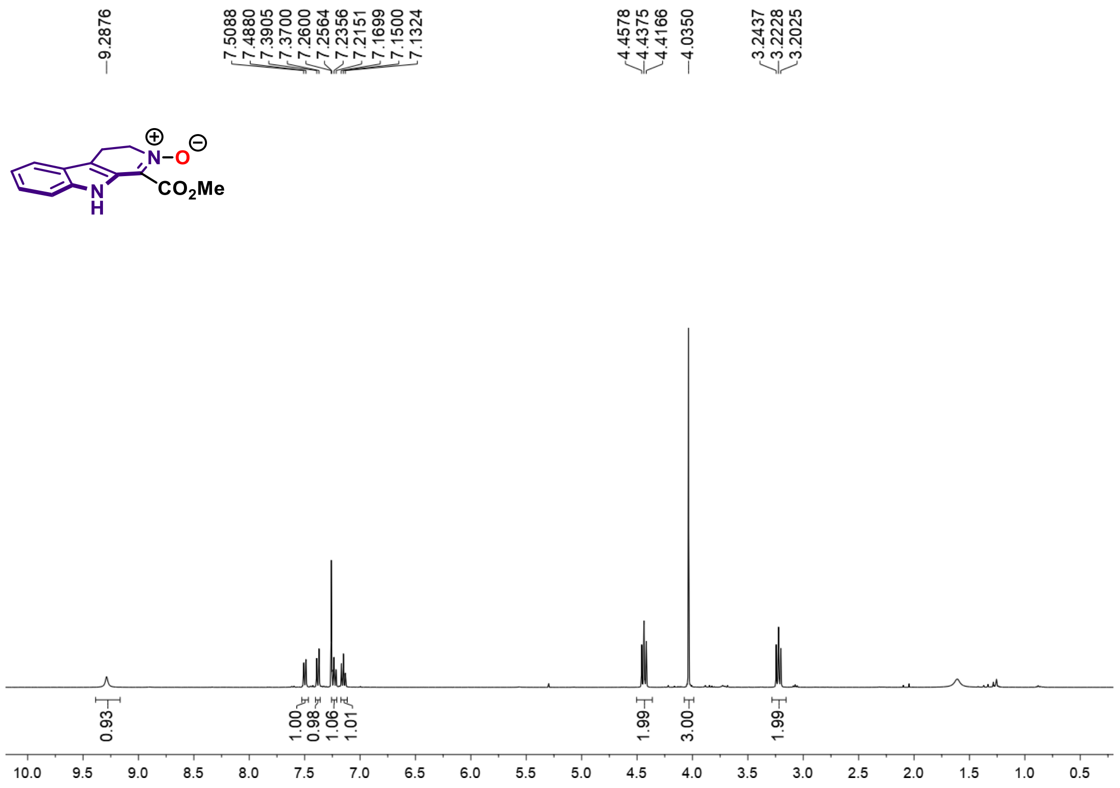

Supplementary Figure 62: ^{13}C NMR of 6a (101 MHz, $CDCl_3$)

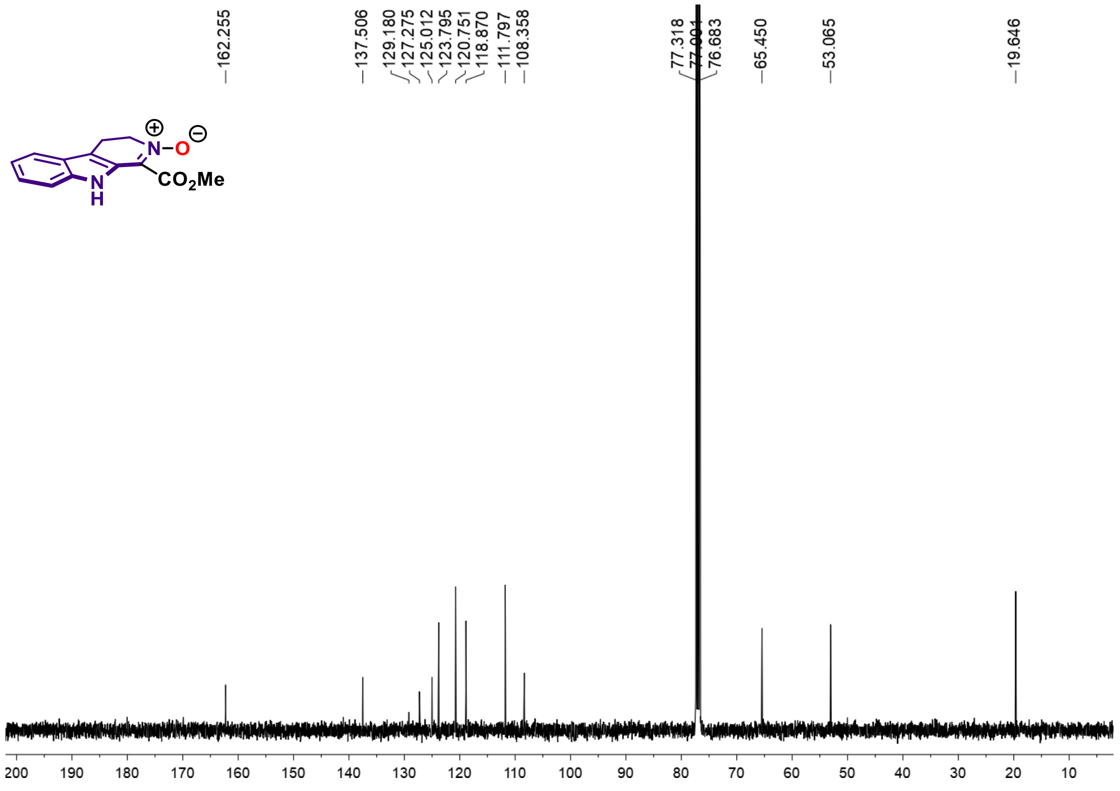

Supplementary Figure 63: ^{1}H NMR of 6b (400 MHz, CDCl3)

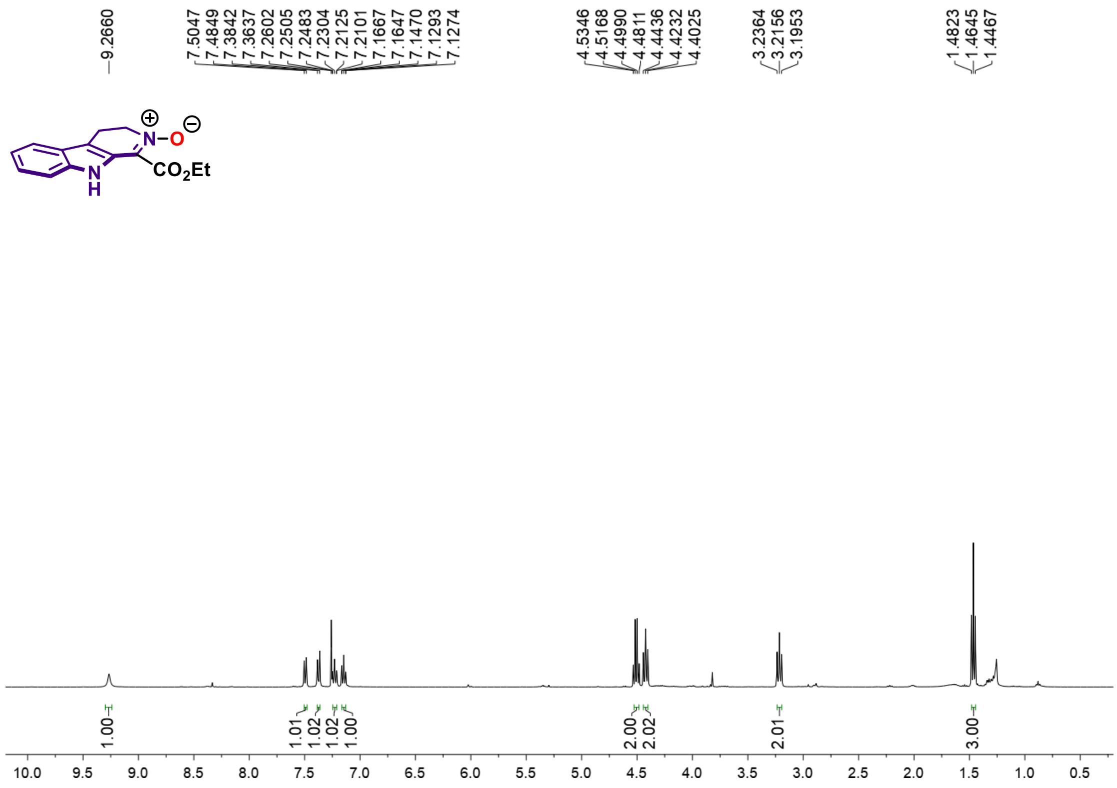

Supplementary Figure 64: ^{13}C NMR of 6b (101 MHz, CDCl3)

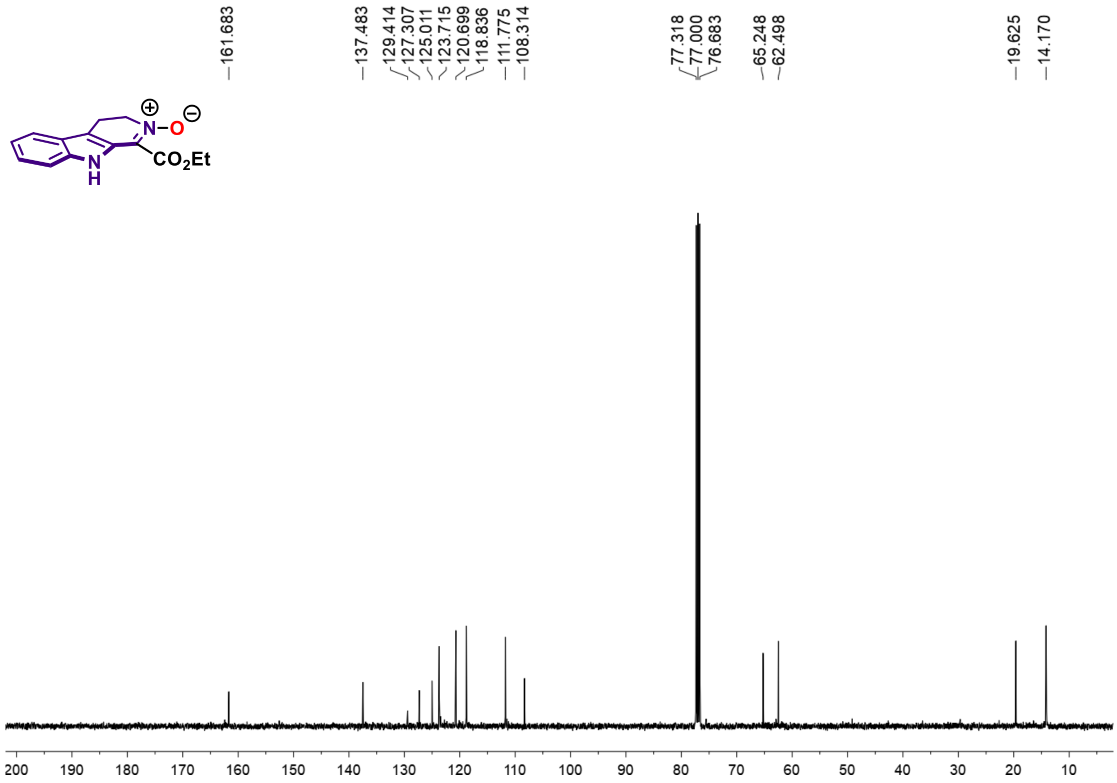

Supplementary Figure 65: ^{1}H NMR of 6c (400 MHz, $CDCl_3$)

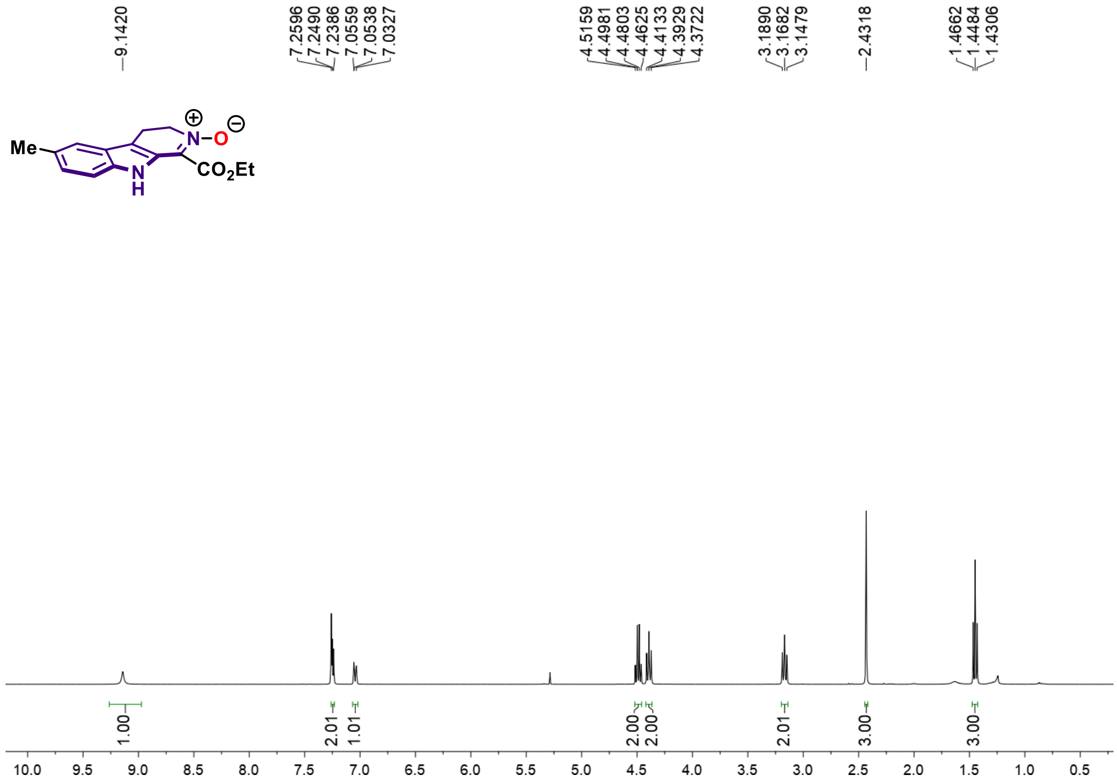

Supplementary Figure 66: ^{13}C NMR of 6c (101 MHz, $CDCl_3$)

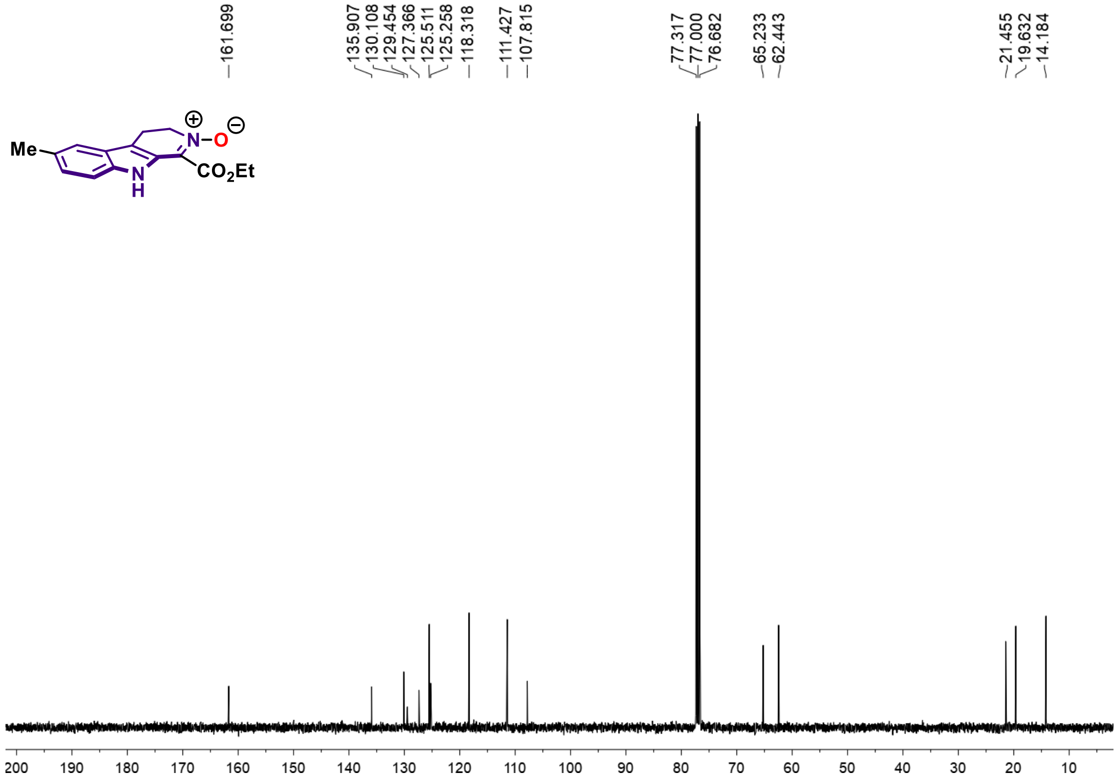

Supplementary Figure 67: ^{1}H NMR of 6d (400 MHz, $CDCl_3$)

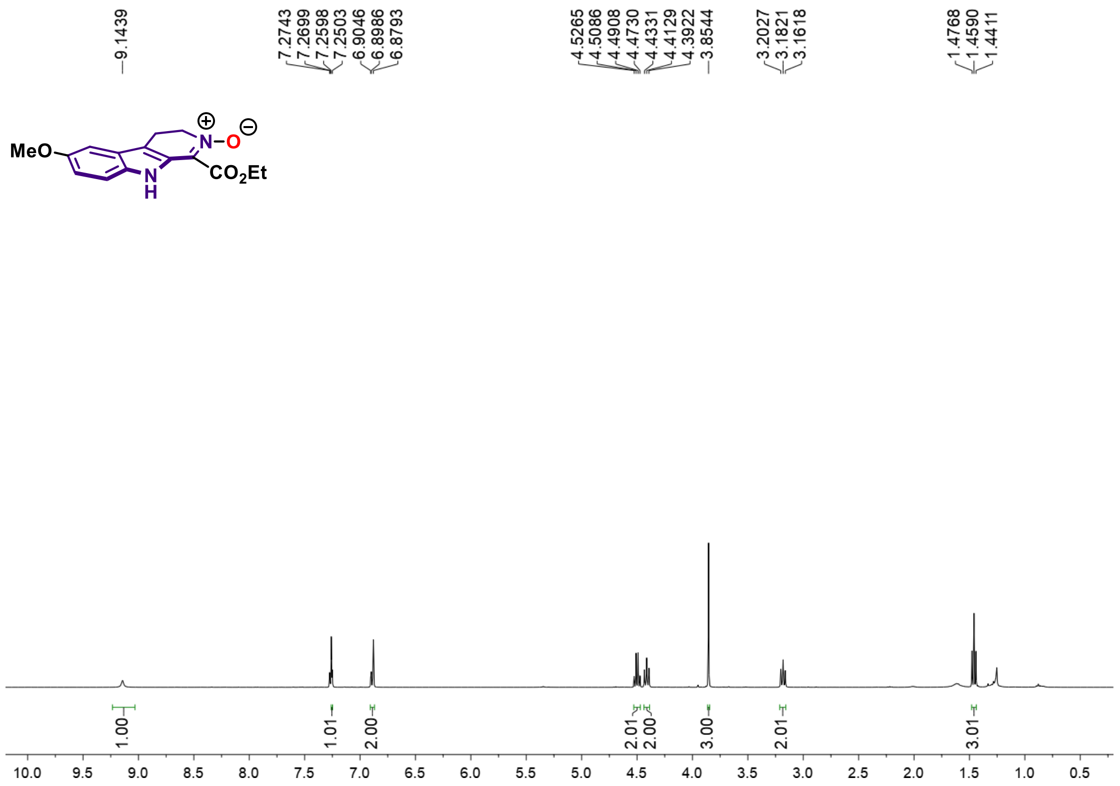

Supplementary Figure 68: ^{13}C NMR of 6d (101 MHz, $CDCl_3$)

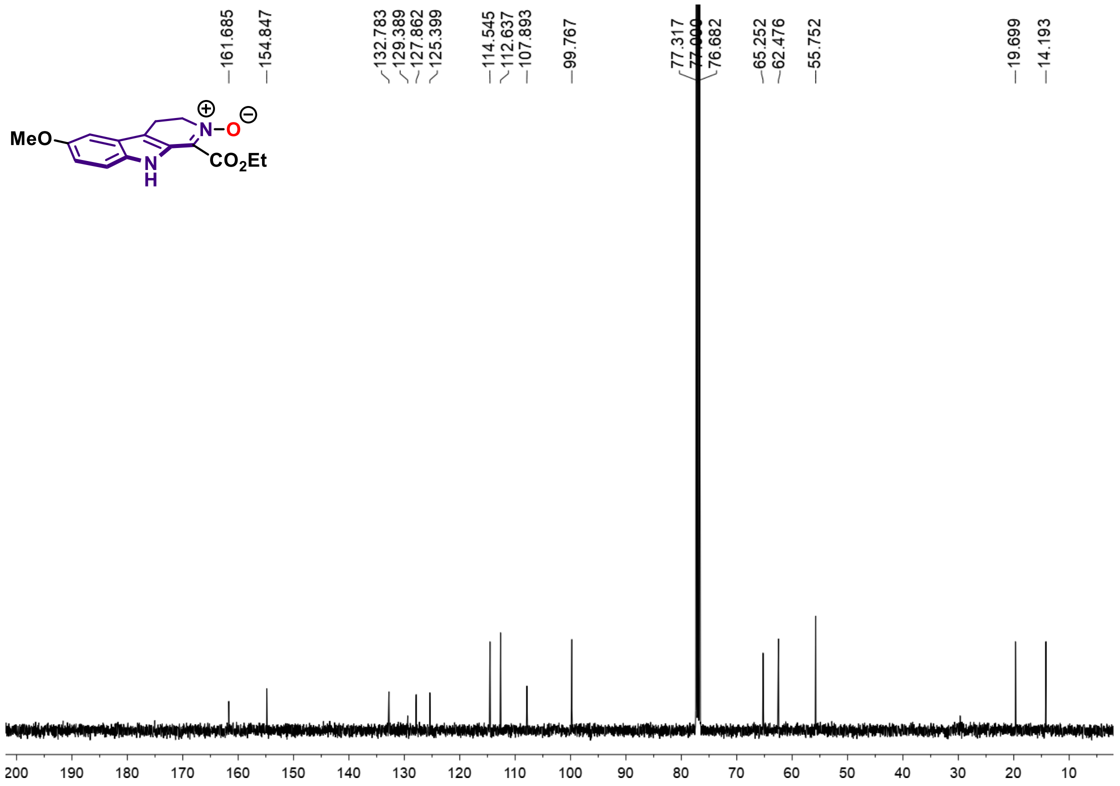

Supplementary Figure 69: ^{1}H NMR of 6e (400 MHz, $CDCl_3$)

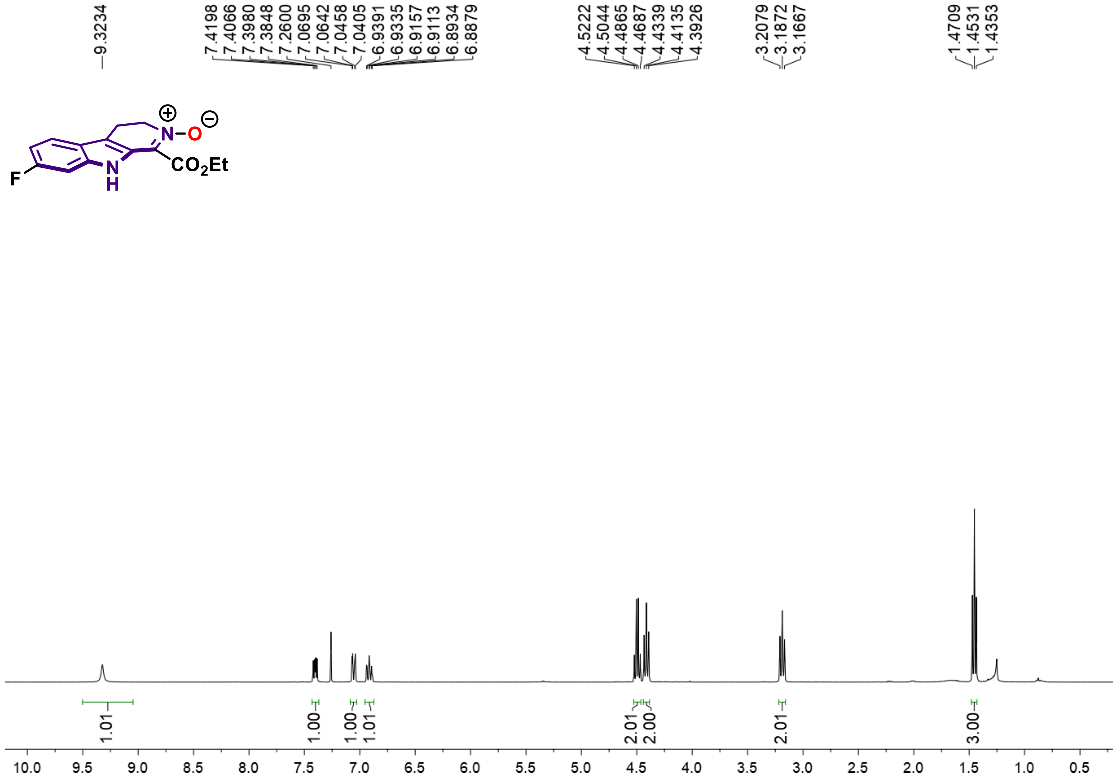

Supplementary Figure 70: ^{13}C NMR of 6e (101 MHz, $CDCl_3$)

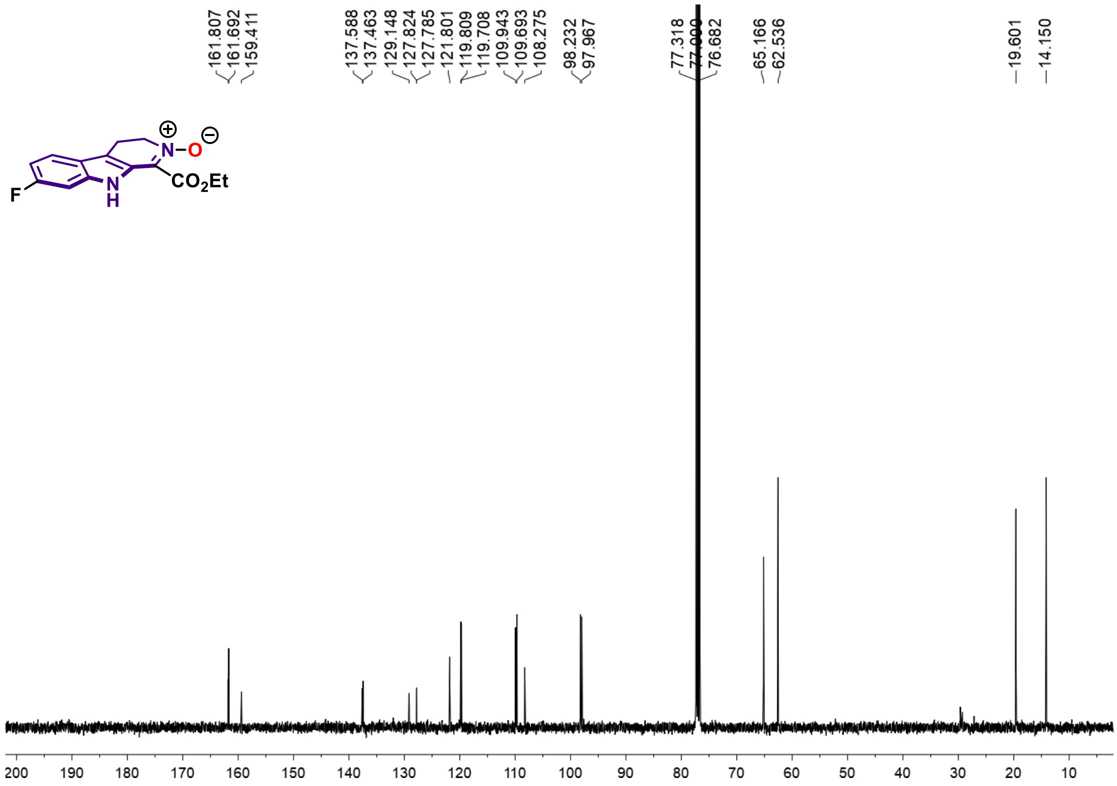

Supplementary Figure 71: ^{1}H NMR of 6f (400 MHz, $CDCl_3$)

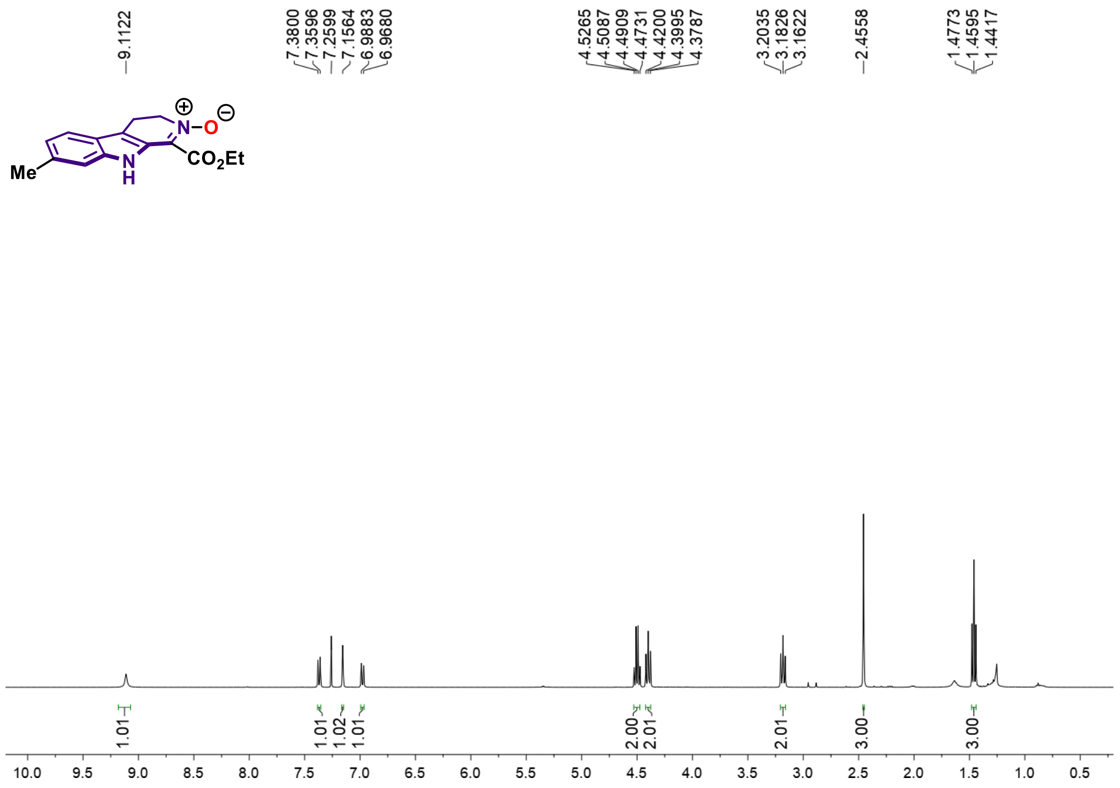

Supplementary Figure 72: ^{13}C NMR of 6f (101 MHz, $CDCl_3$)

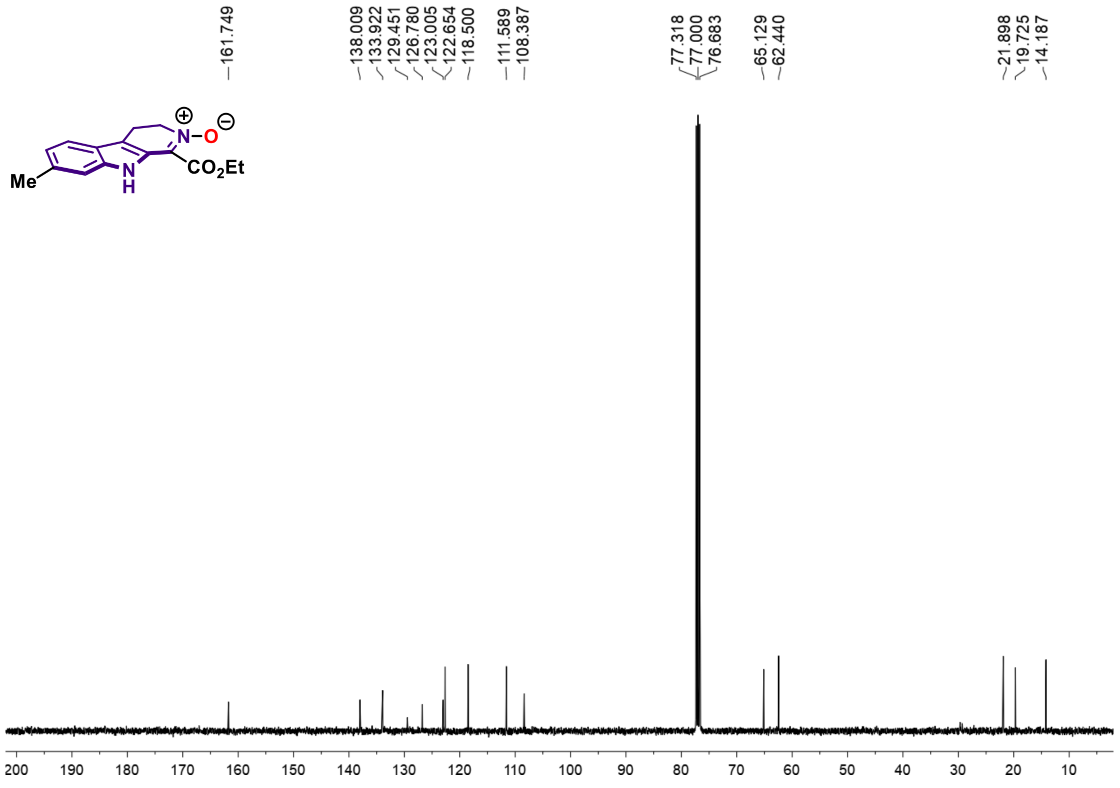

Supplementary Figure 73: ^{1}H NMR of 6g (400 MHz, $CDCl_3$)

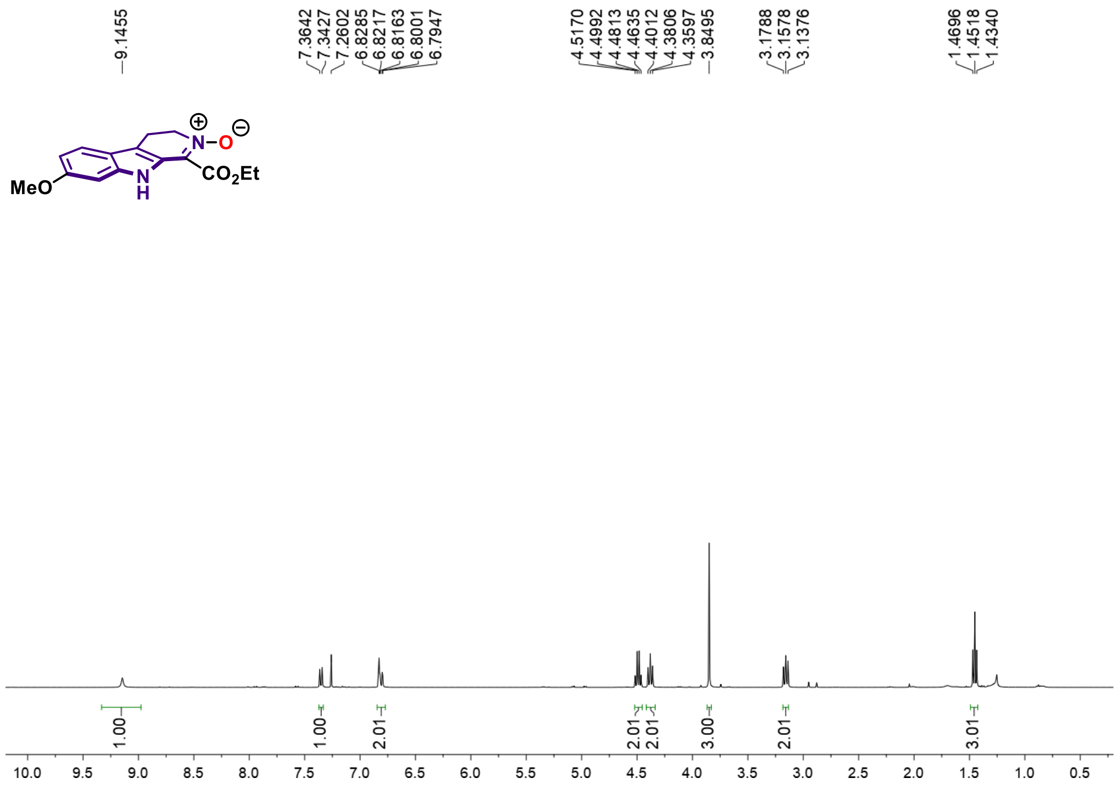

Supplementary Figure 74: ^{13}C NMR of 6g (101 MHz, $CDCl_3$)

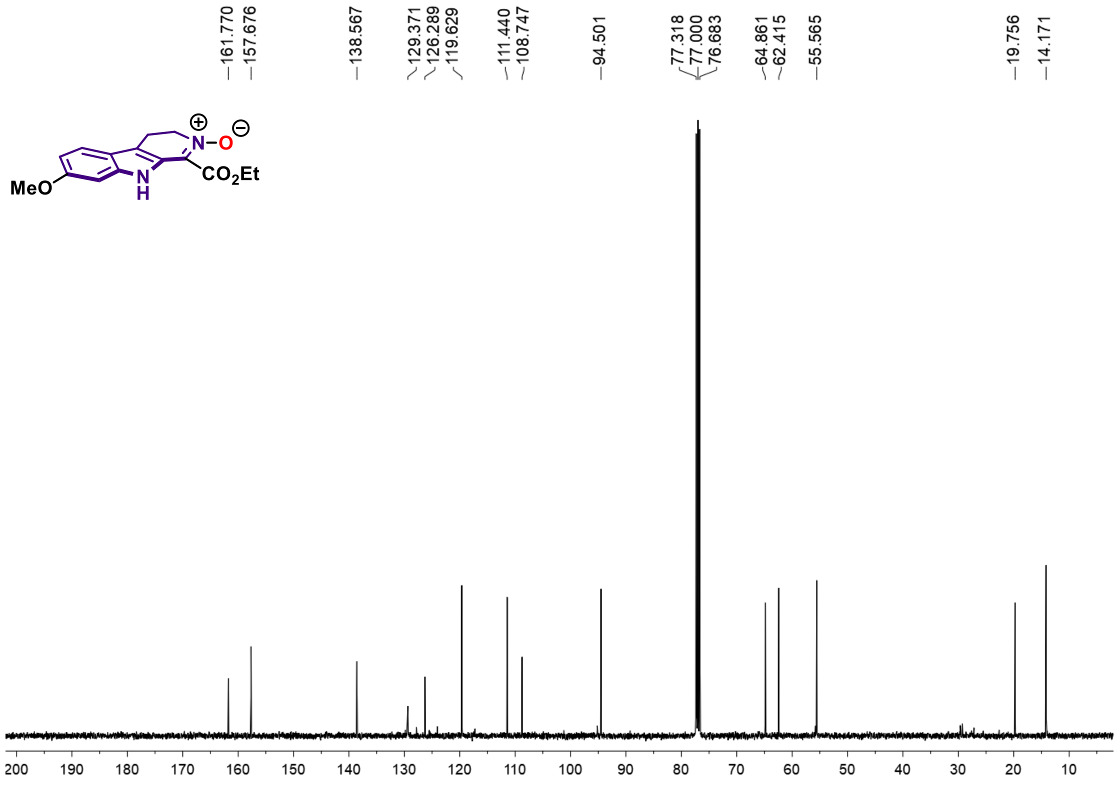

Supplementary Figure 75: ^{1}H NMR of 3a (400 MHz, $CDCl_3$)

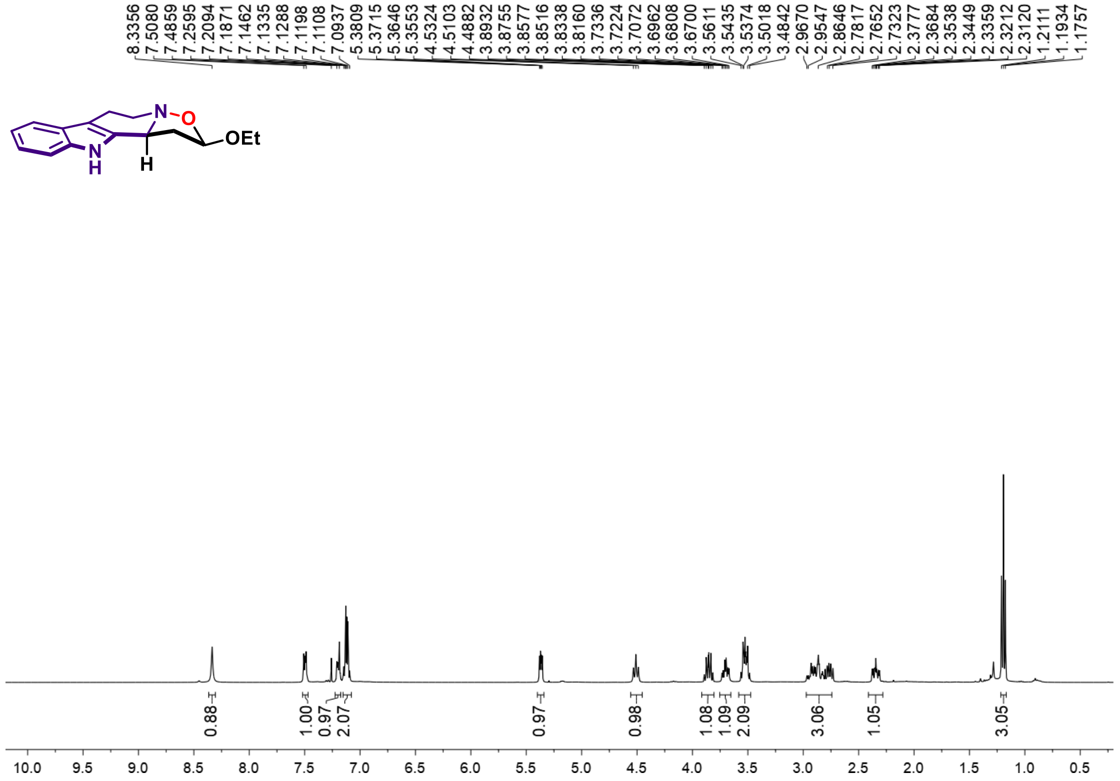

Supplementary Figure 76: ^{13}C NMR of 3a (101 MHz, $CDCl_3$)

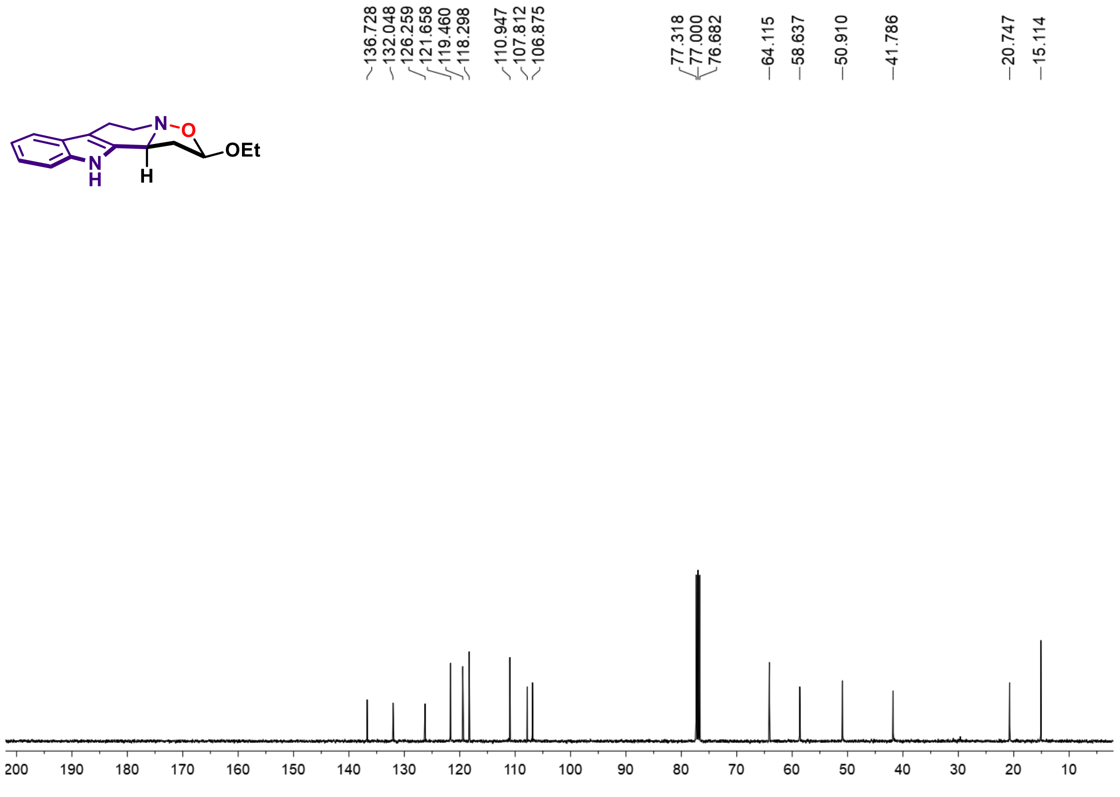

Supplementary Figure 77: HPLC spectrum of 3a

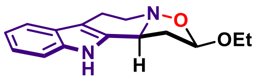

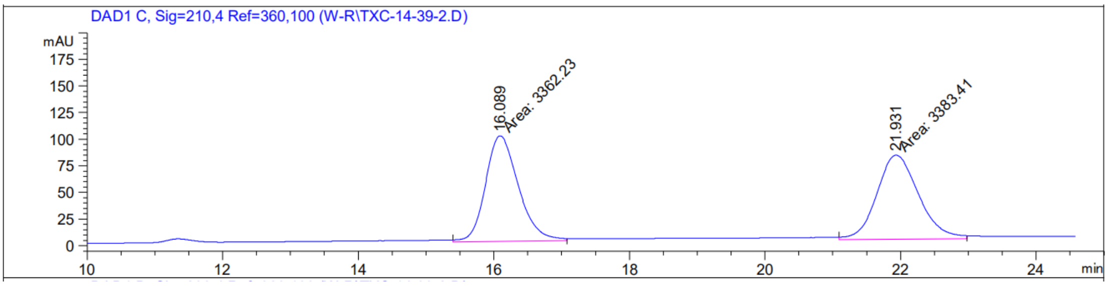

Signal 2: DAD1 C, Sig=210,4 Ref=360,100

Peak #	RetTime [min]	Type	Width [min]	Area [mAU*s]	Height [mAU]	Area %
1	16.089	MM	0.5649	3362.22510	99.20403	49.8430
2	21.931	MM	0.7154	3383.41235	78.82857	50.1570

Totals : 6745.63745 178.03260

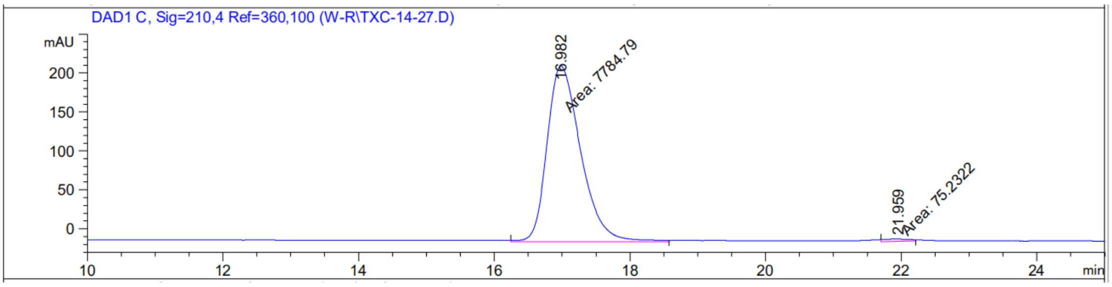

Signal 2: DAD1 C, Sig=210,4 Ref=360,100

Peak #	RetTime [min]	Type	Width [min]	Area [mAU*s]	Height [mAU]	Area %
1	16.982	MM	0.5749	7784.79492	225.69627	99.0429
2	21.959	MM	0.3393	75.23219	2.72005	0.9571

Totals : 7860.02711 228.41632

Supplementary Figure 78: ^{1}H NMR of 3b (400 MHz, CDCl$_3$)

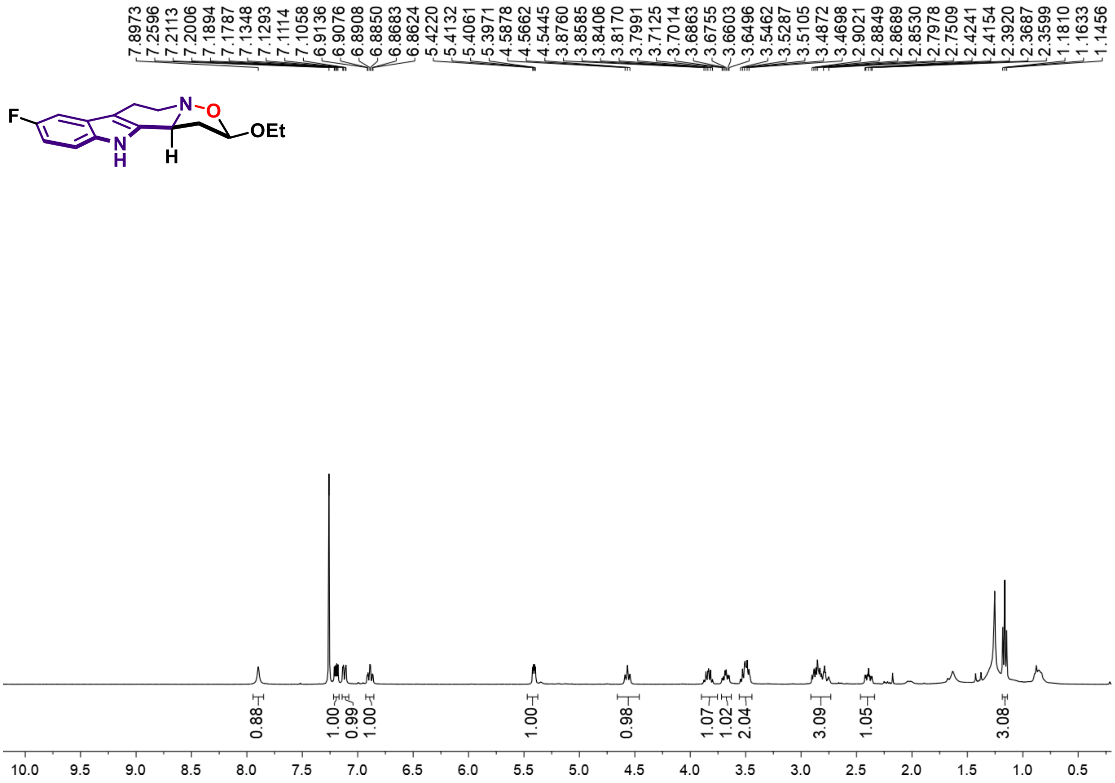

Supplementary Figure 79: ^{13}C NMR of 3b (101 MHz, CDCl$_3$)

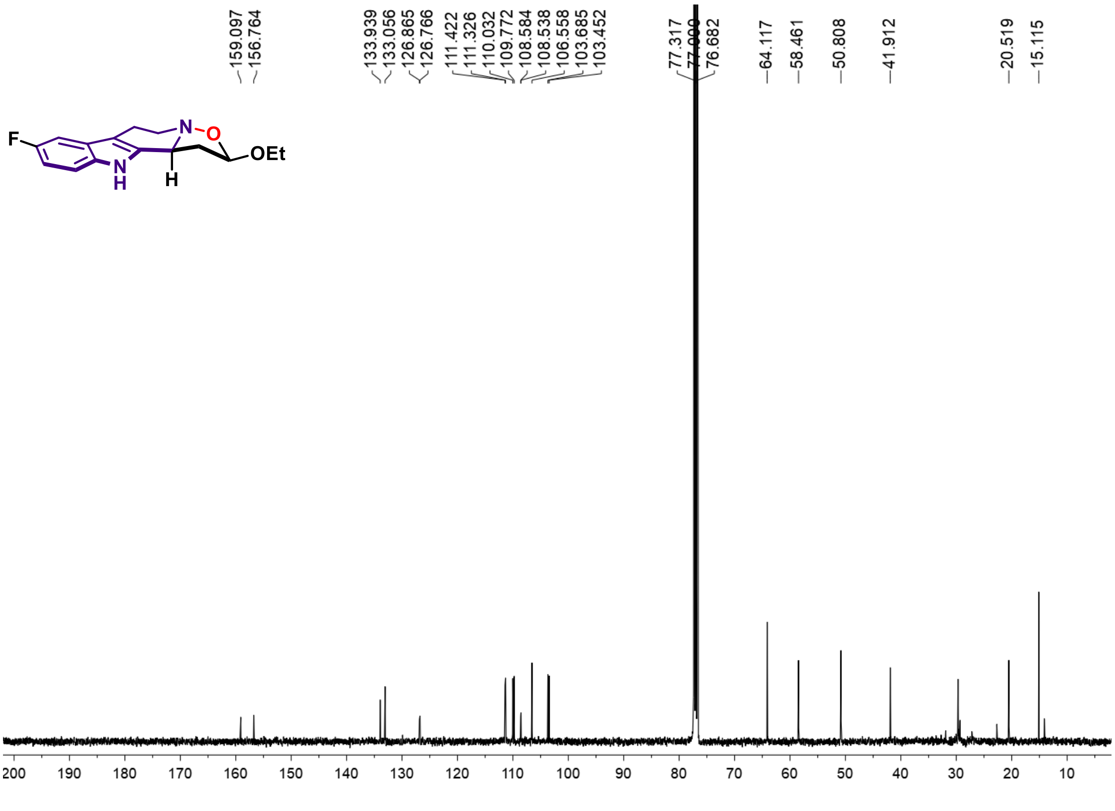

Supplementary Figure 80: HPLC spectrum of 3b

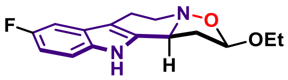

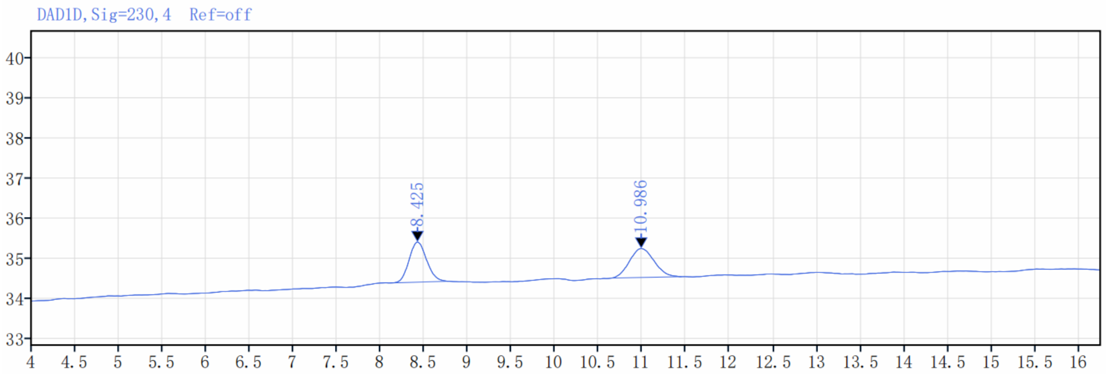

Signal : DAD1D, Sig=230, 4 Ref=off

RetTime [min]	Type	Width [min]	Area [mAU*s]	Height [mAU]	Area%
8.425	MM m	0.22	14.00	1.00	49.69
10.986	MM m	0.29	14.18	0.72	50.31
	Totals	0.50	28.18		

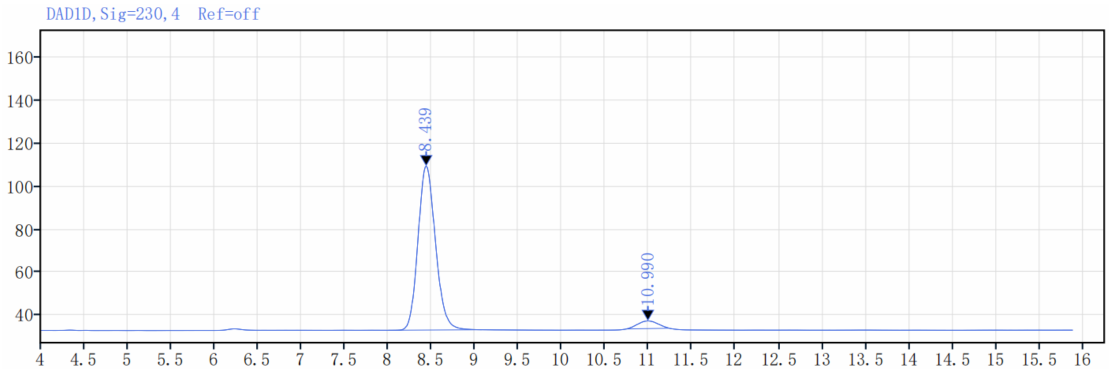

Signal : DAD1D, Sig=230, 4 Ref=off

RetTime [min]	Type	Width [min]	Area [mAU*s]	Height [mAU]	Area%
8.439	MM m	0.22	1070.69	76.36	94.92
10.990	MM m	0.26	57.34	3.57	5.08
	Totals	0.48	1128.03		

Supplementary Figure 81: ^{1}H NMR of 3c (400 MHz, $CDCl_3$)

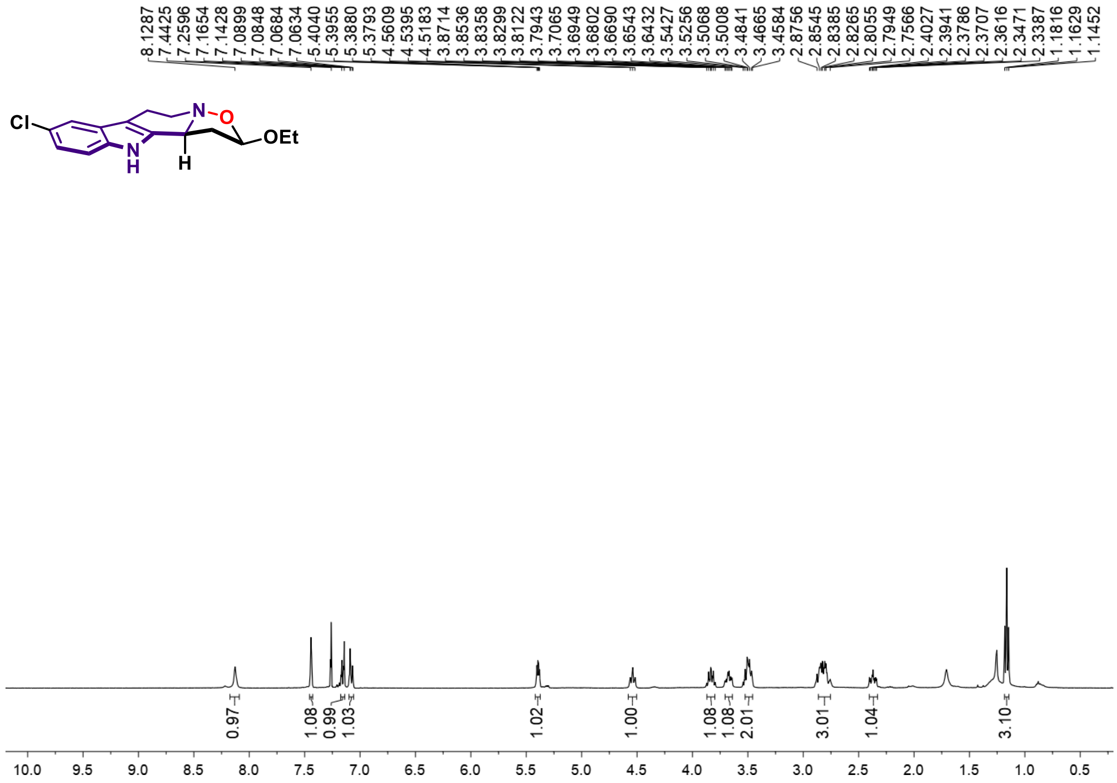

Supplementary Figure 82: ^{13}C NMR of 3c (101 MHz, $CDCl_3$)

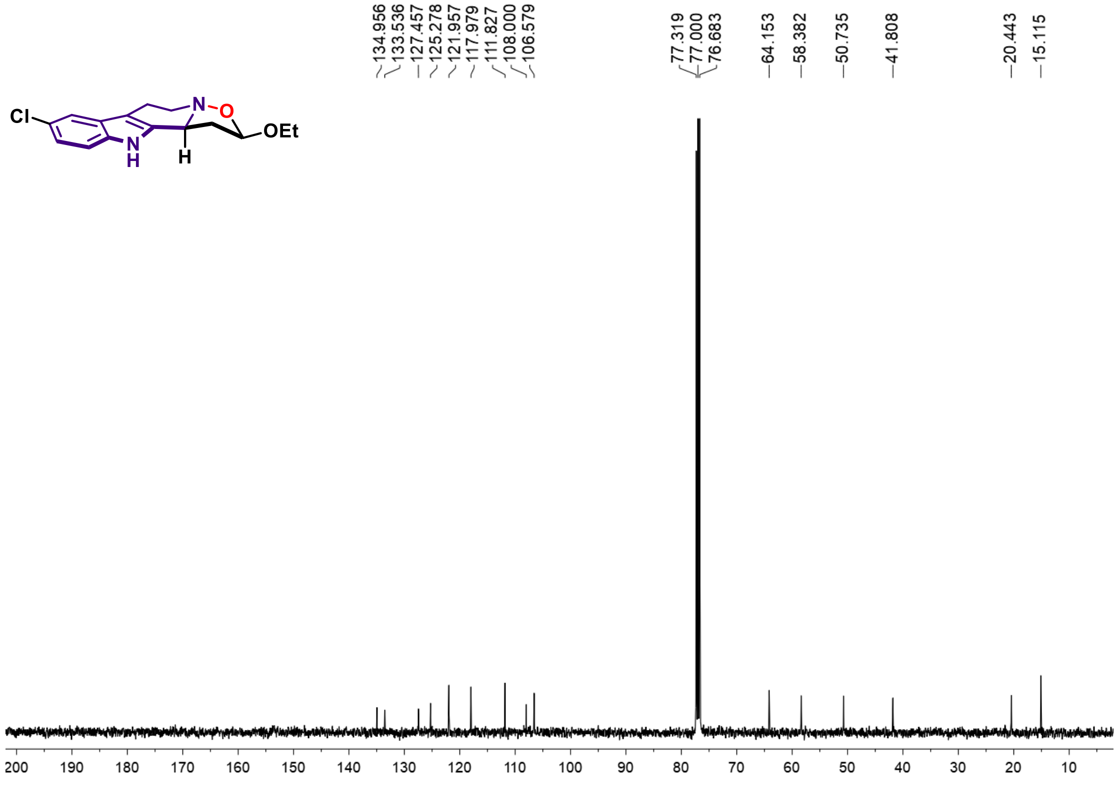

Supplementary Figure 83: HPLC spectrum of 3c

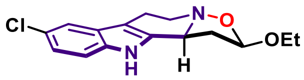

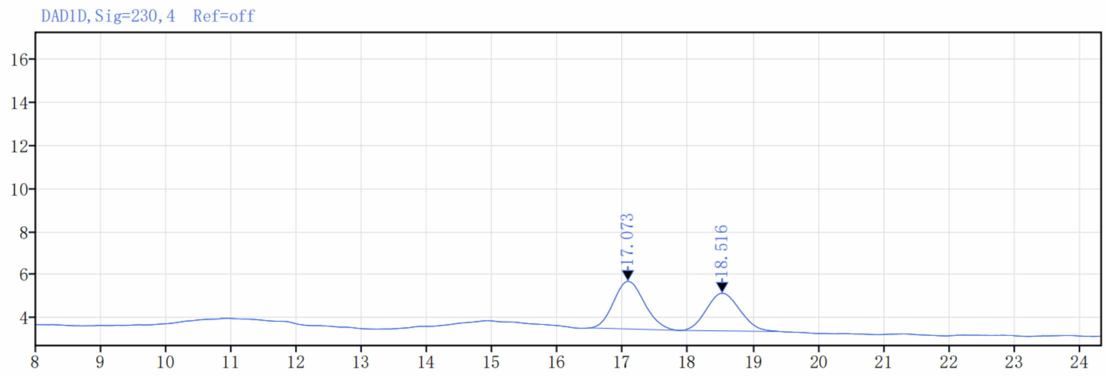

Signal : DAD1D, Sig=230, 4 Ref=off

RetTime [min]	Type	Width [min]	Area [mAU*s]	Height [mAU]	Area%
17.073	MM m	0.47	72.88	2.22	53.88
18.516	MM m	0.50	62.37	1.75	46.12
	Totals	0.97	135.25		

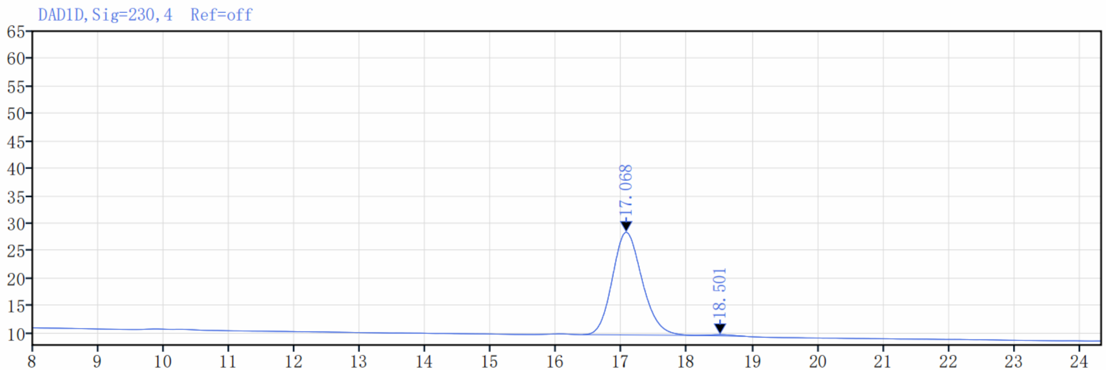

Signal : DAD1D, Sig=230, 4 Ref=off

RetTime [min]	Type	Width [min]	Area [mAU*s]	Height [mAU]	Area%
17.068	MM m	0.48	590.23	18.68	99.08
18.501	MM m	0.30	5.47	0.22	0.92
	Totals	0.78	595.70		

Supplementary Figure 84: ^{1}H NMR of 3d (400 MHz, $CDCl_3$)

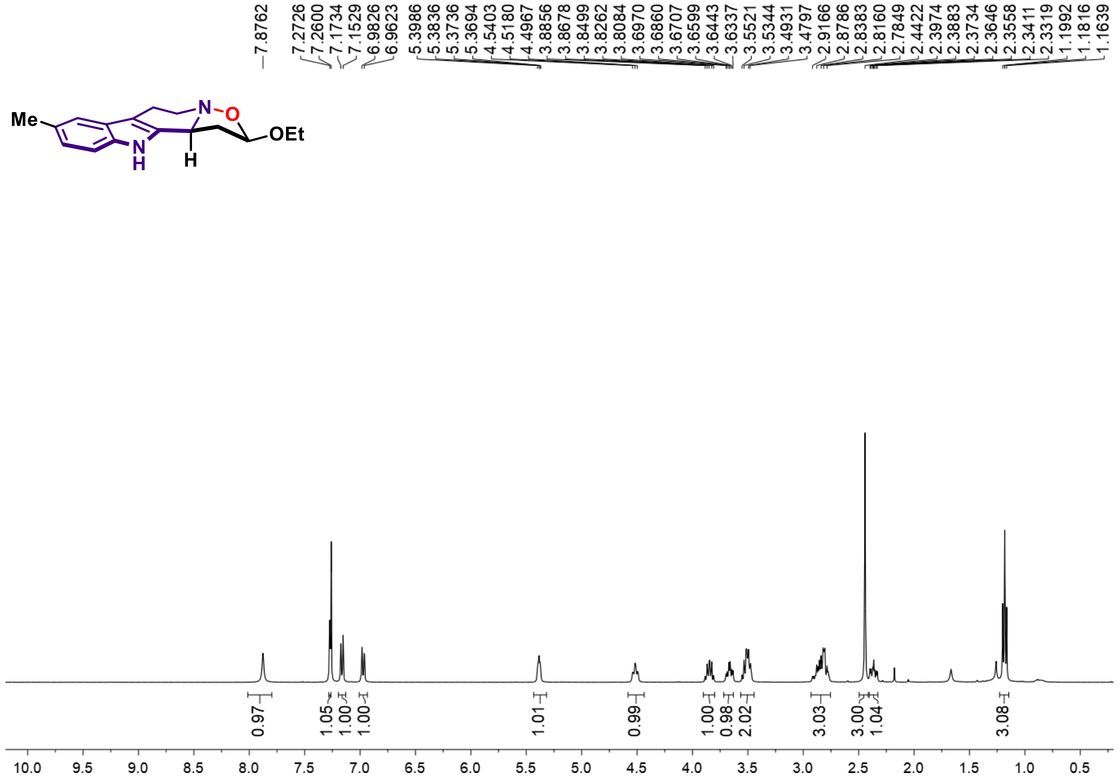

Supplementary Figure 85: ^{13}C NMR of 3d (101 MHz, $CDCl_3$)

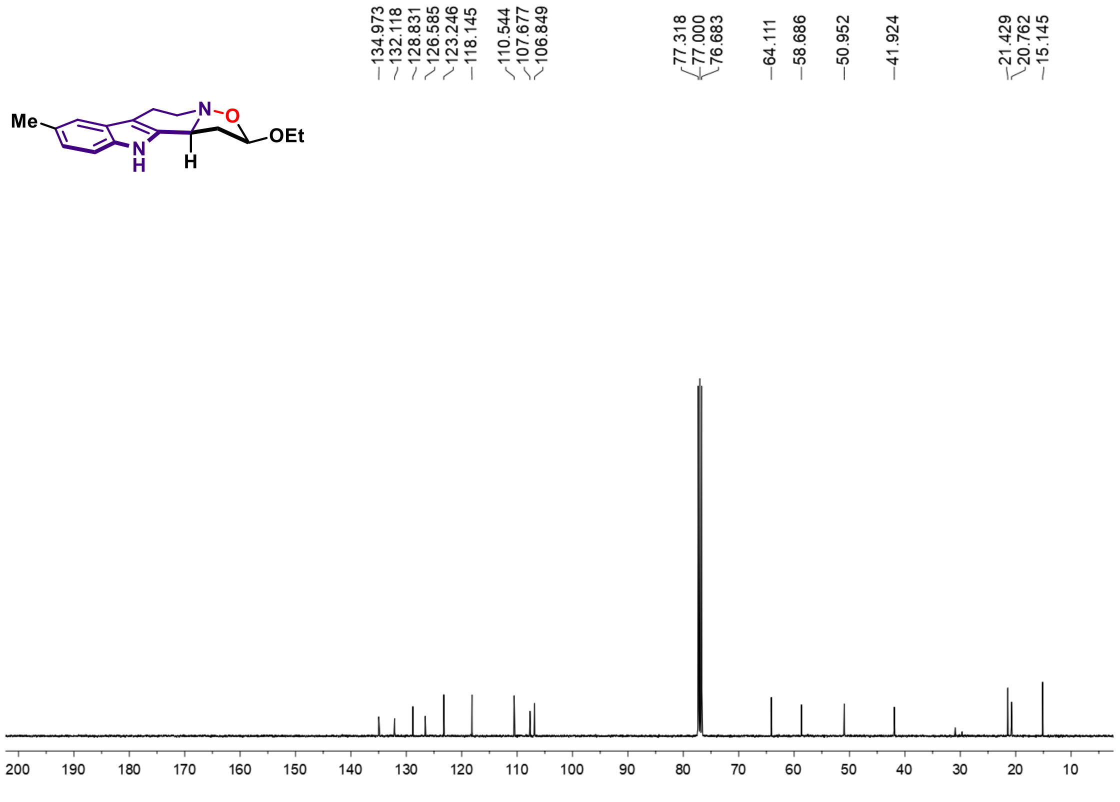

Supplementary Figure 86: HPLC spectrum of 3d

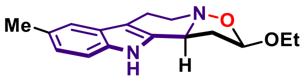

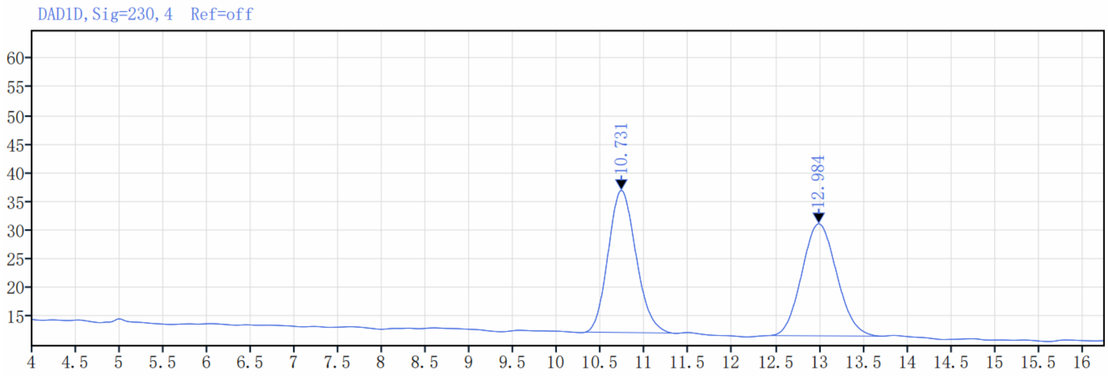

Signal : DAD1D, Sig=230, 4 Ref=off

RetTime [min]	Type	Width [min]	Area [mAU*s]	Height [mAU]	Area%
10.731	MM m	0.34	548.42	24.74	50.33
12.984	MM m	0.43	541.28	19.52	49.67
	Totals	0.78	1089.70		

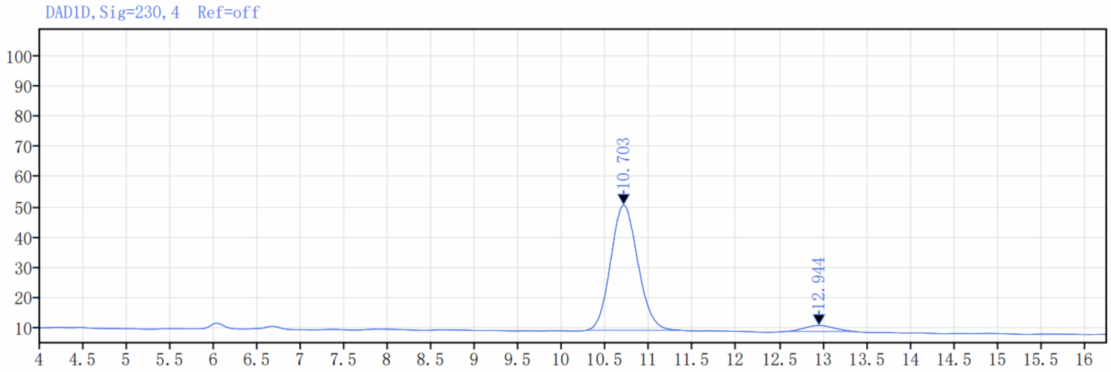

Signal : DAD1D, Sig=230, 4 Ref=off

RetTime [min]	Type	Width [min]	Area [mAU*s]	Height [mAU]	Area%
10.703	MM m	0.34	899.51	41.56	95.02
12.944	MM m	0.35	47.12	2.01	4.98
	Totals	0.68	946.64		

Supplementary Figure 87: ^{1}H NMR of 3e (400 MHz, CDCl$_3$)

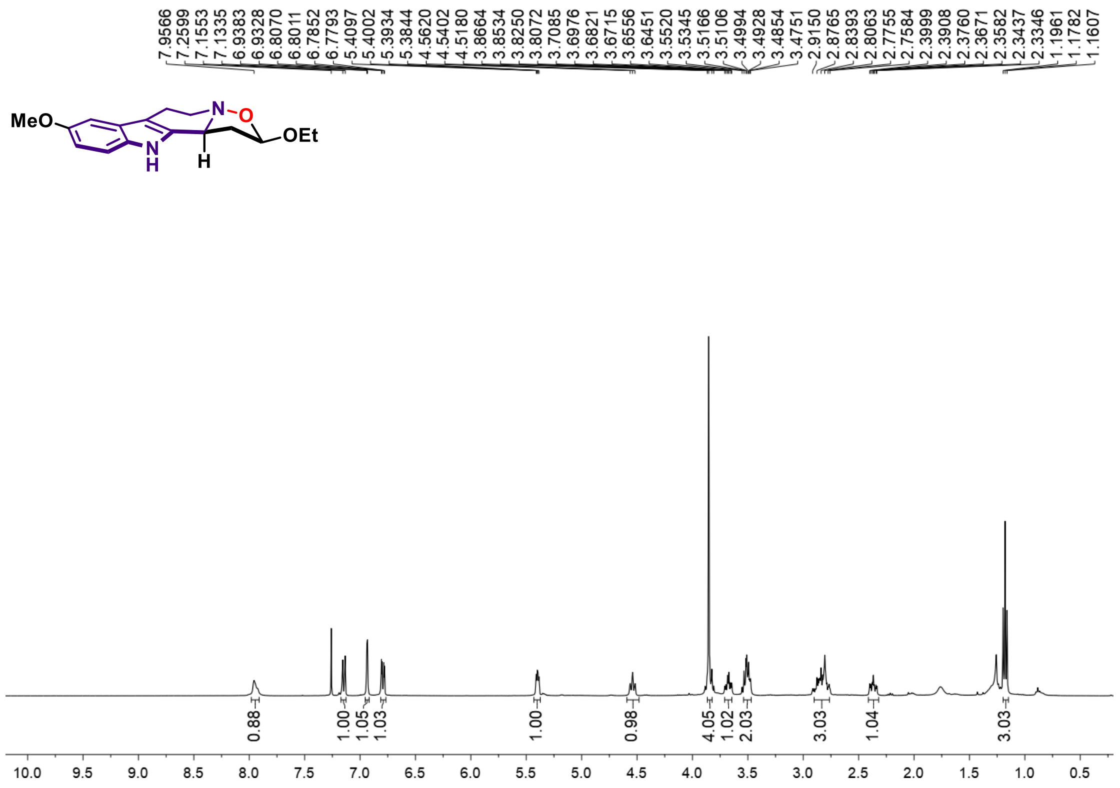

Supplementary Figure 88: ^{13}C NMR of 3e (101 MHz, CDCl$_3$)

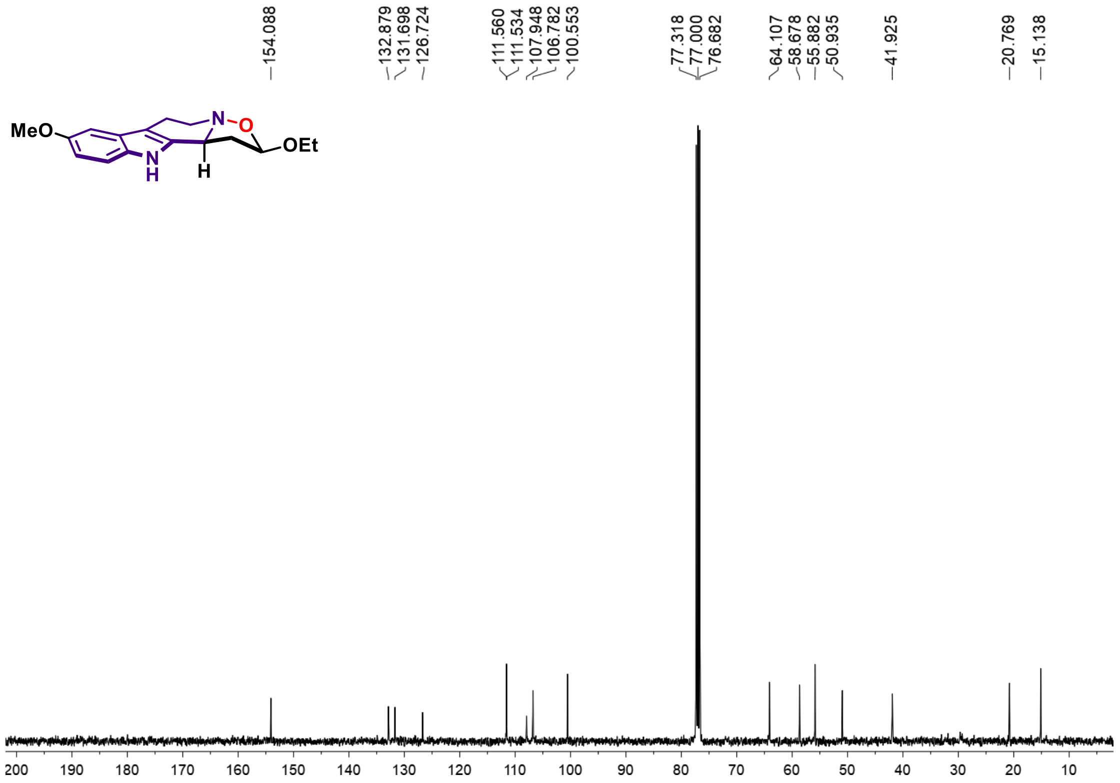

Supplementary Figure 89: HPLC spectrum of 3e

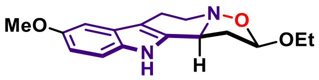

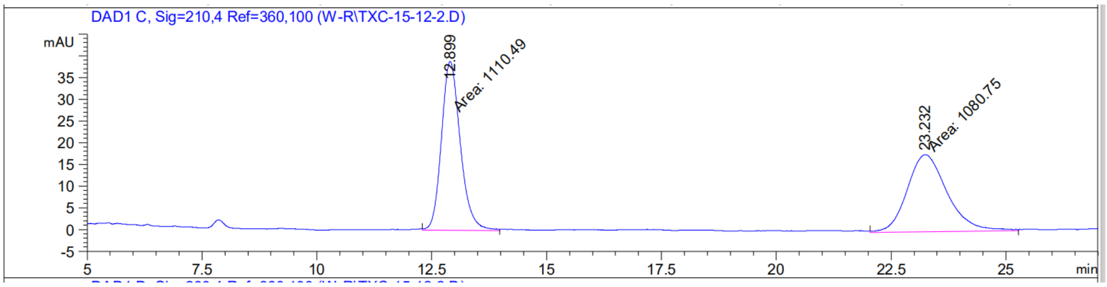

Signal 2: DAD1 C, Sig=210,4 Ref=360,100

Peak #	RetTime [min]	Type	Width [min]	Area [mAU*s]	Height [mAU]	Area %
1	12.899	MM	0.4758	1110.49036	38.89892	50.6787
2	23.232	MM	1.0162	1080.74683	17.72470	49.3213

Totals : 2191.23718 56.62362

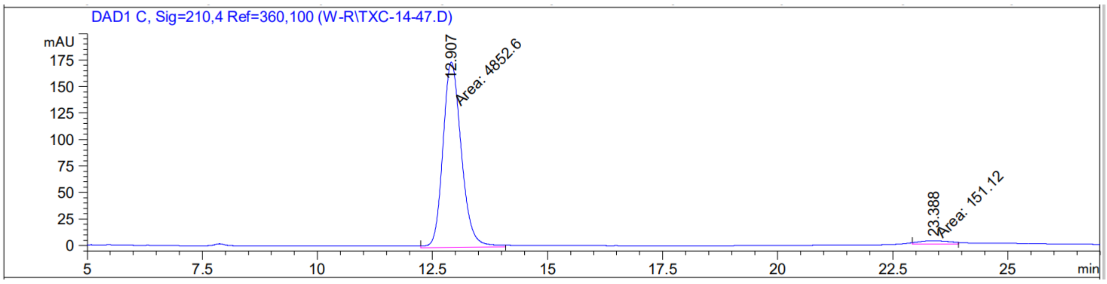

Signal 2: DAD1 C, Sig=210,4 Ref=360,100

Peak #	RetTime [min]	Type	Width [min]	Area [mAU*s]	Height [mAU]	Area %
1	12.907	MM	0.4629	4852.59717	174.71666	96.9798
2	23.388	MM	0.7985	151.12033	3.15434	3.0202

Totals : 5003.71750 177.87100

Supplementary Figure 90: ^{1}H NMR of 3f (400 MHz, $CDCl_3$)

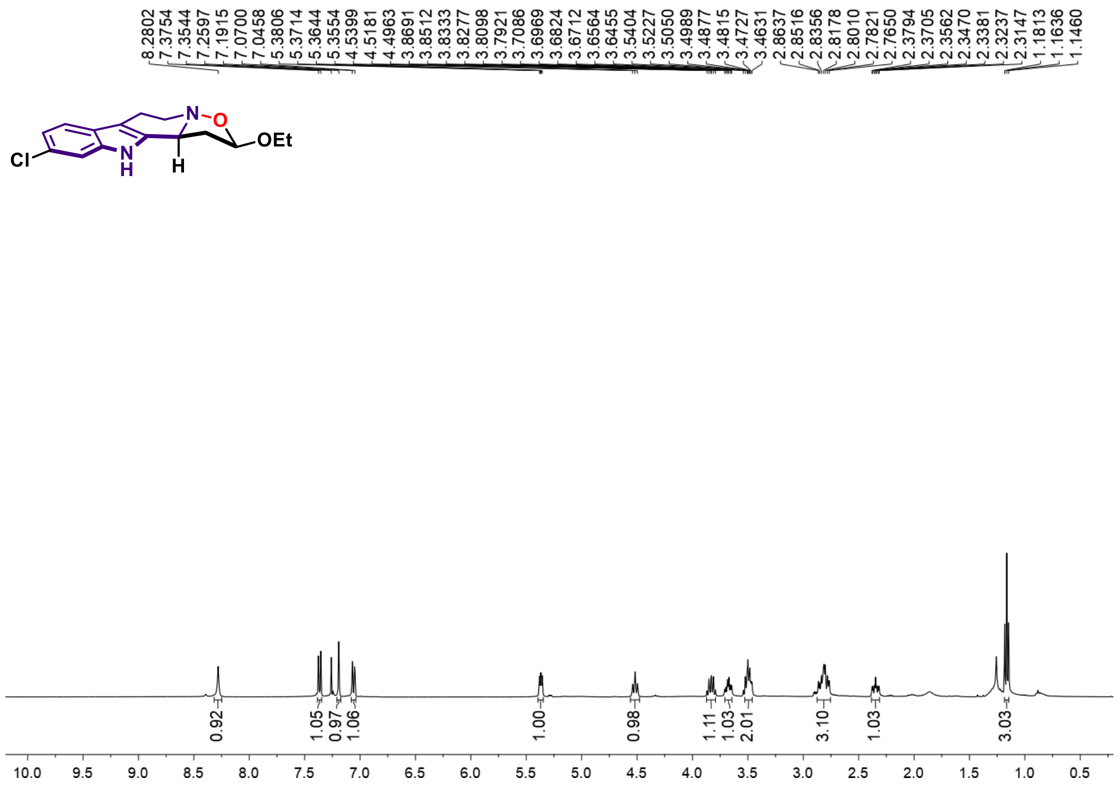

Supplementary Figure 91: ^{13}C NMR of 3f (101 MHz, $CDCl_3$)

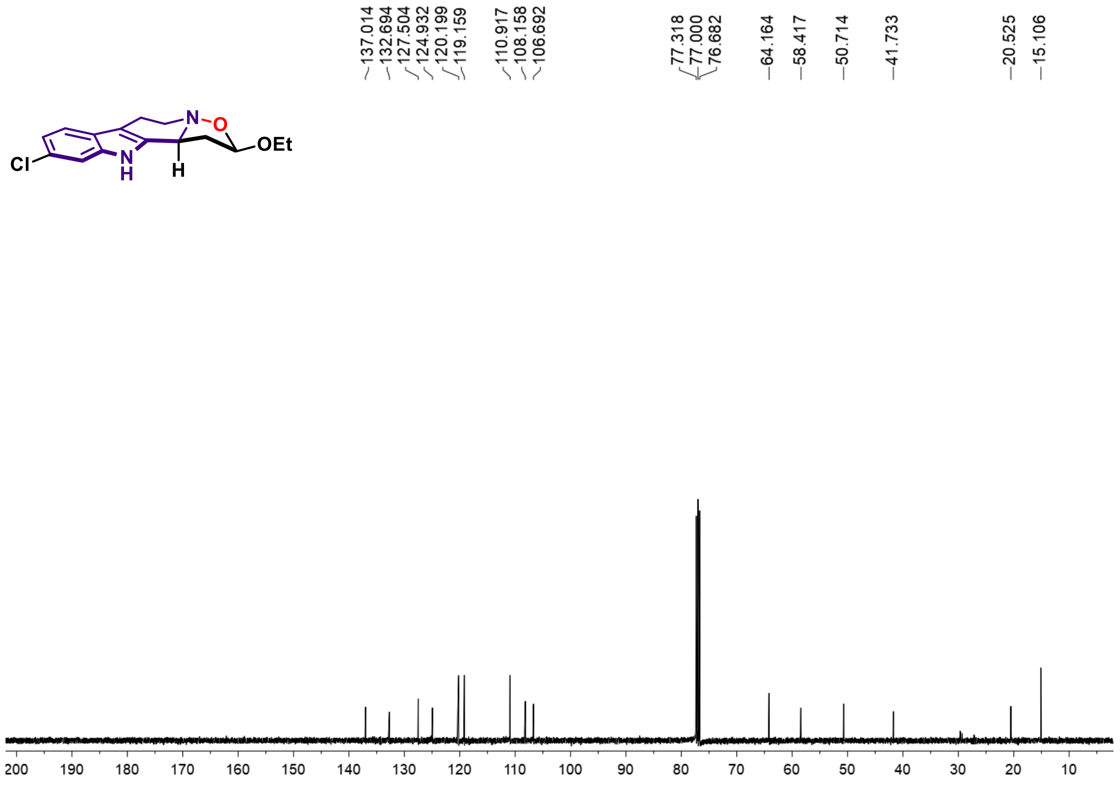

Supplementary Figure 92: HPLC spectrum of 3f

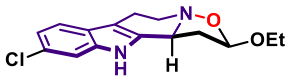

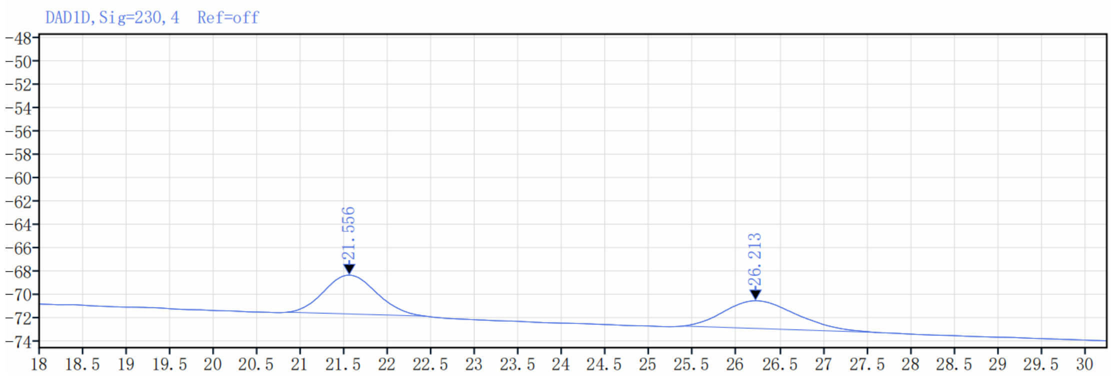

Signal :	DAD1D, Sig=230, 4 Ref=off				
RetTime [min]	Type	Width [min]	Area [mAU*s]	Height [mAU]	Area%
21.556	MM m	0.61	139.62	3.33	50.37
26.213	MM m	0.69	137.56	2.37	49.63
	Totals	1.30	277.18		

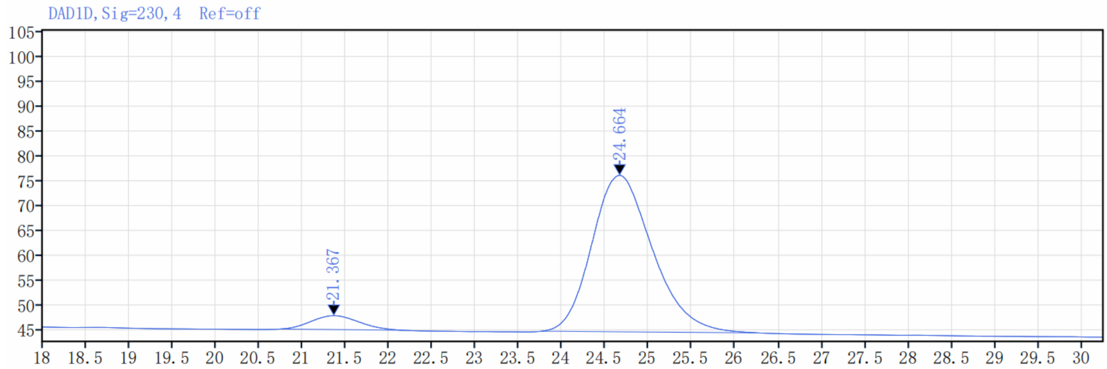

Signal :	DAD1D, Sig=230, 4 Ref=off				
RetTime [min]	Type	Width [min]	Area [mAU*s]	Height [mAU]	Area%
21.367	MM m	0.52	106.93	2.79	6.52
24.664	MM m	0.74	1533.20	31.49	93.48
	Totals	1.26	1640.13		

Supplementary Figure 93: ^{1}H NMR of 3g (400 MHz, CDCl$_3$)

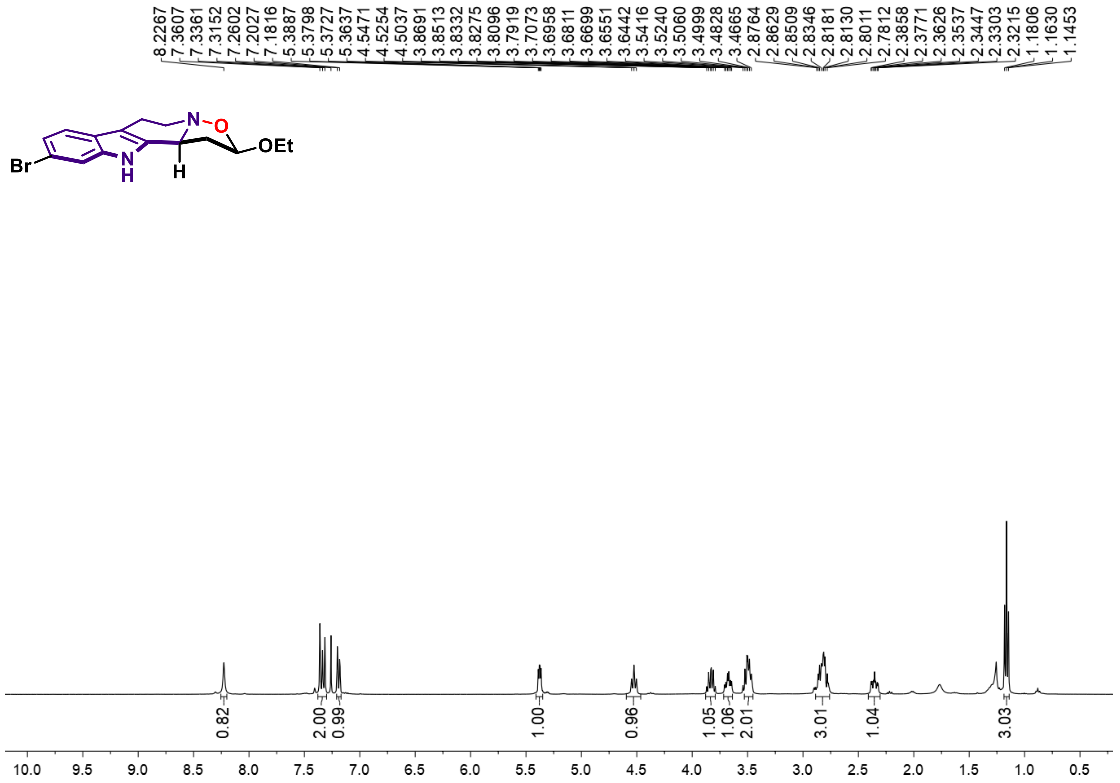

Supplementary Figure 94: ^{13}C NMR of 3g (101 MHz, CDCl$_3$)

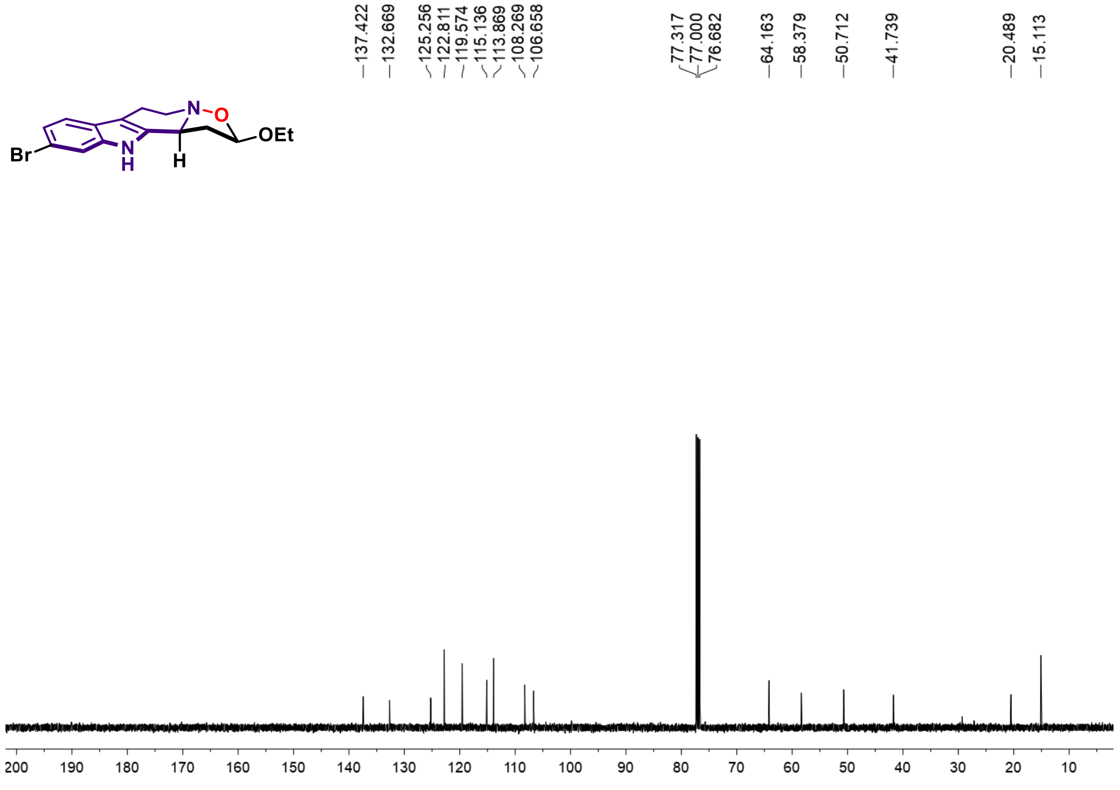

Supplementary Figure 95: HPLC spectrum of 3g

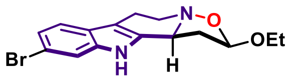

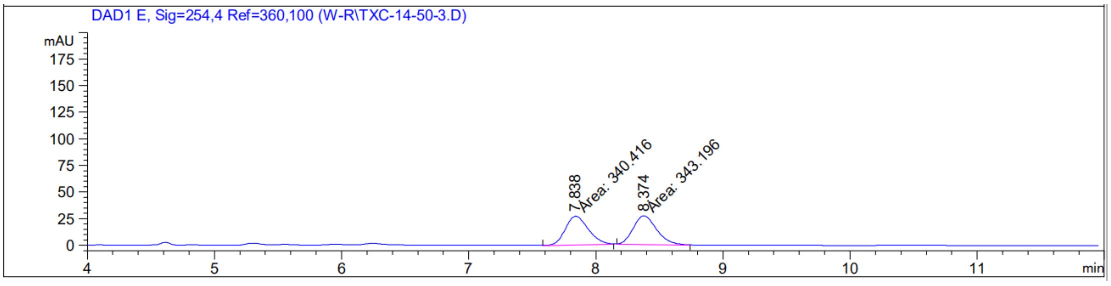

Signal 4: DAD1 E, Sig=254,4 Ref=360,100

Peak #	RetTime [min]	Type	Width [min]	Area [mAU*s]	Height [mAU]	Area %
1	7.838	MM	0.2096	340.41577	27.06720	49.7967
2	8.374	MM	0.2131	343.19574	26.84167	50.2033

Totals : 683.61151 53.90887

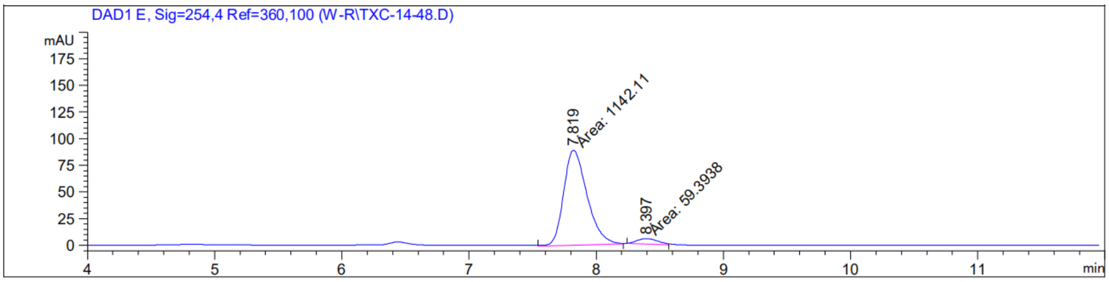

Signal 2: DAD1 C, Sig=210,4 Ref=360,100

Peak #	RetTime [min]	Type	Width [min]	Area [mAU*s]	Height [mAU]	Area %
1	7.819	MM	0.2137	6529.80127	509.35782	94.9615
2	8.390	MM	0.1935	346.45752	29.83509	5.0385

Totals : 6876.25879 539.19291

Supplementary Figure 96: ^{1}H NMR of 3h (400 MHz, $CDCl_3$)

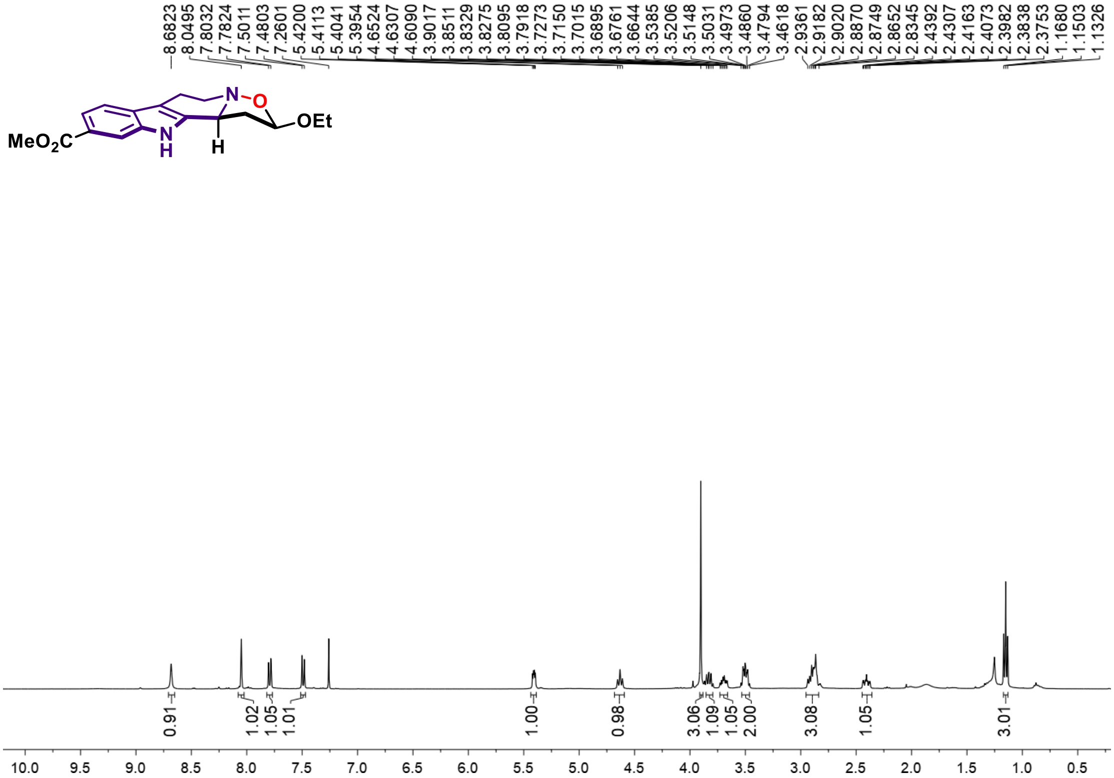

Supplementary Figure 97: ^{13}C NMR of 3h (101 MHz, $CDCl_3$)

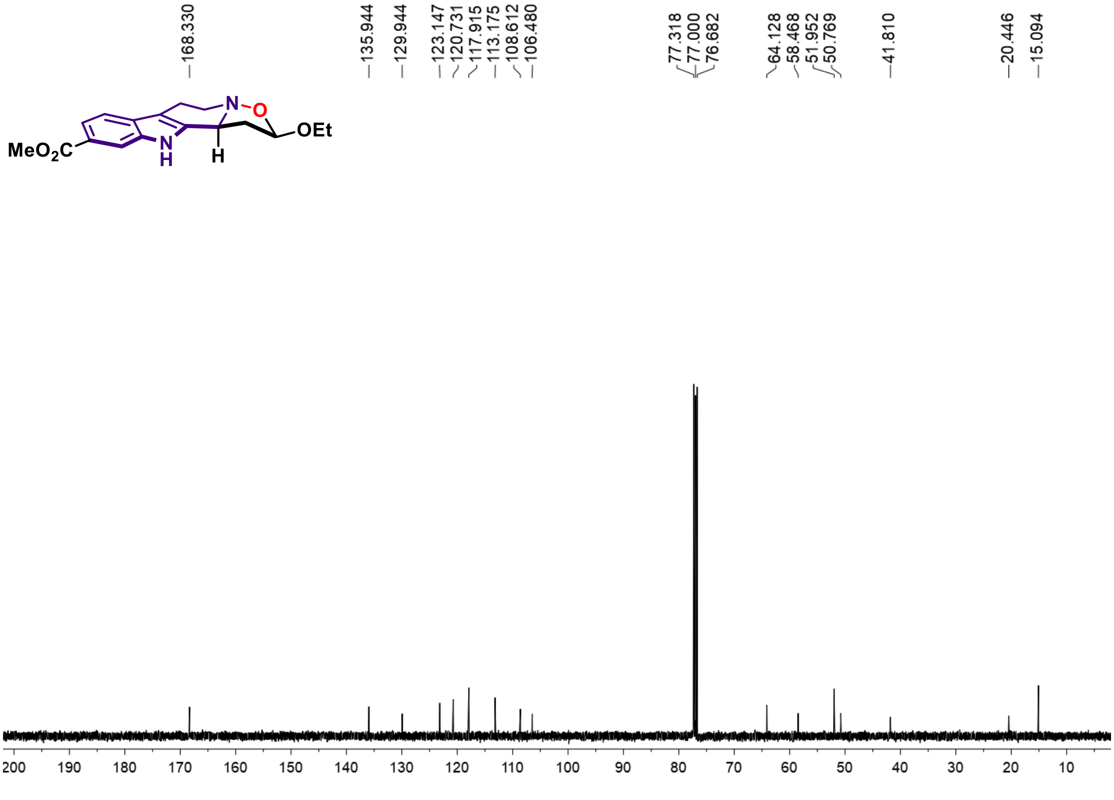

Supplementary Figure 98: HPLC spectrum of 3h

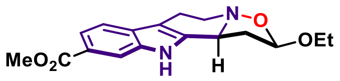

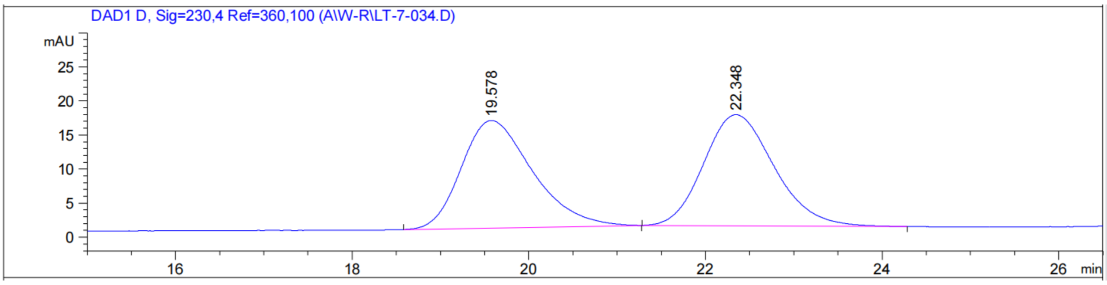

Signal 3: DAD1 D, Sig=230,4 Ref=360,100

Peak #	RetTime [min]	Type	Width [min]	Area [mAU*s]	Height [mAU]	Area %
1	19.578	BB	0.7805	884.91156	15.77486	49.4137
2	22.348	BB	0.7698	905.91052	16.31432	50.5863

Totals : 1790.82208 32.08919

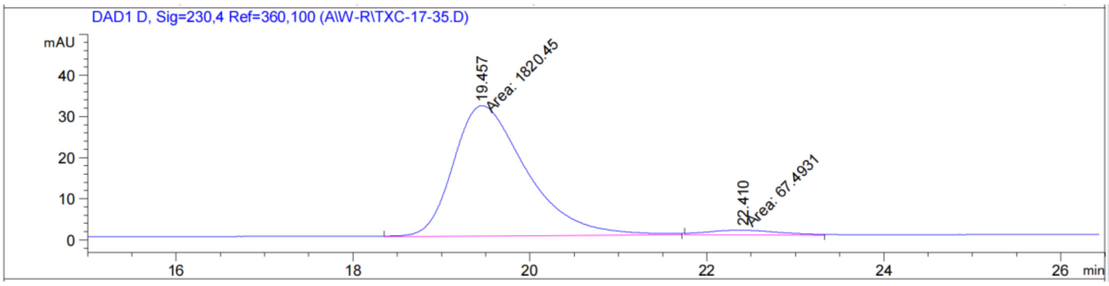

Signal 3: DAD1 D, Sig=230,4 Ref=360,100

Peak #	RetTime [min]	Type	Width [min]	Area [mAU*s]	Height [mAU]	Area %
1	19.457	MM	0.9583	1820.45203	31.66154	96.4251
2	22.410	MM	0.9616	67.49309	1.16977	3.5749

Totals : 1887.94511 32.83131

Supplementary Figure 99: ^{1}H NMR of 3i (400 MHz, CDCl$_3$)

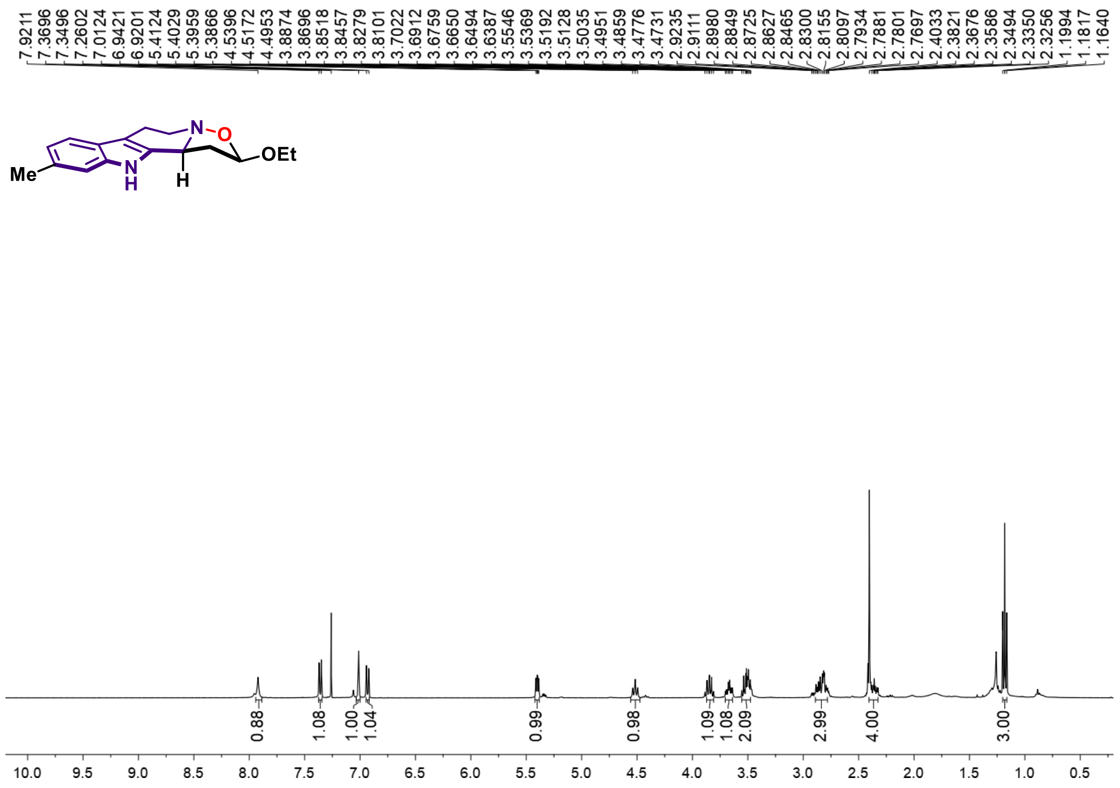

Supplementary Figure 100: ^{13}C NMR of 3i (101 MHz, CDCl$_3$)

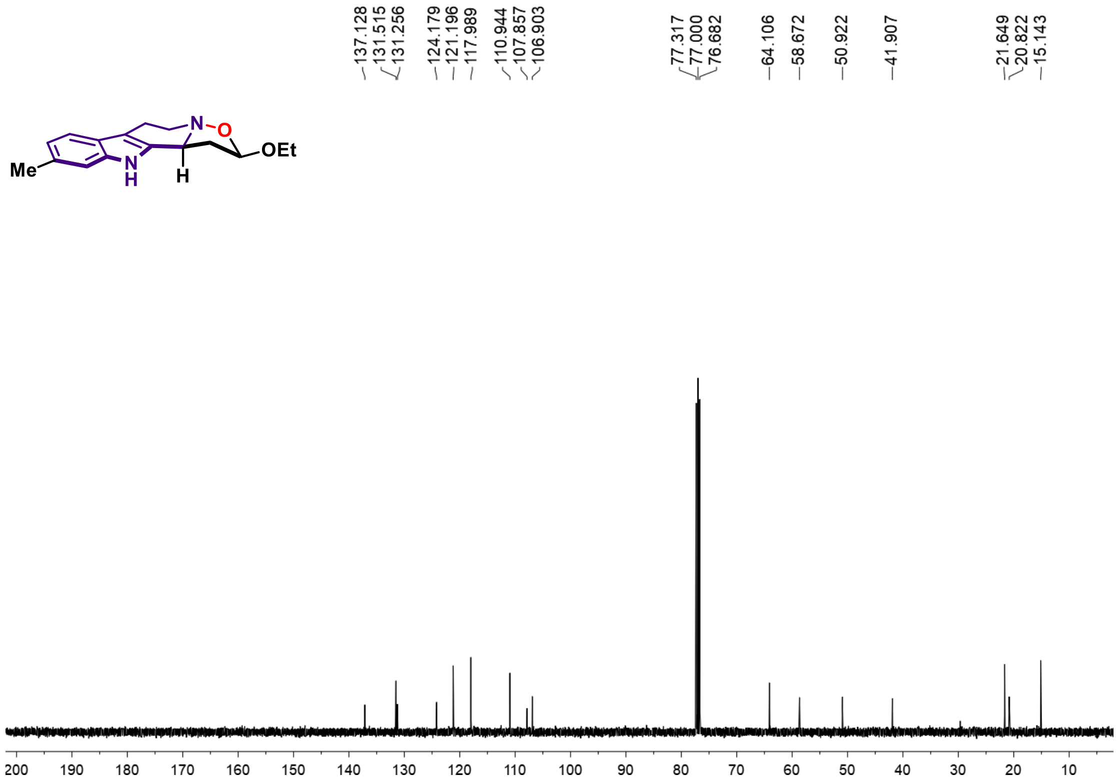

Supplementary Figure 101: HPLC spectrum of 3i

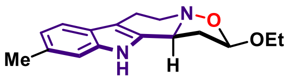

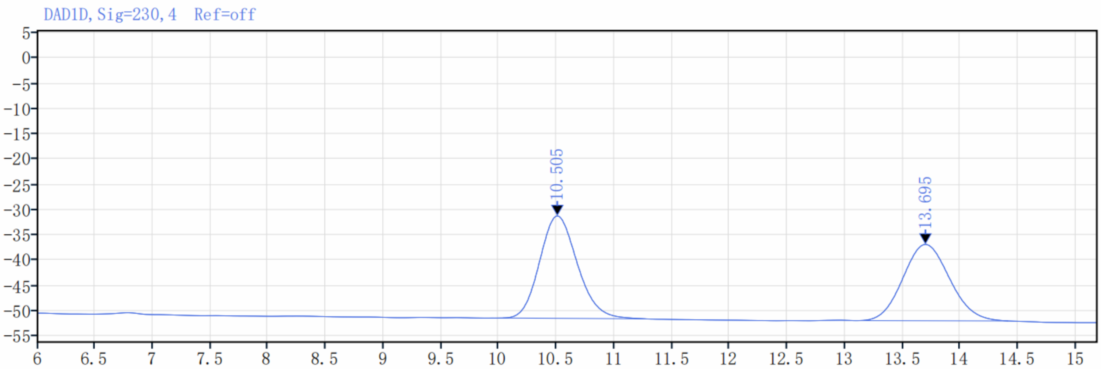

Signal : DAD1D, Sig=230, 4 Ref=off

RetTime [min]	Type	Width [min]	Area [mAU*s]	Height [mAU]	Area%
10.505	MM m	0.35	459.97	20.26	52.01
13.695	MM m	0.44	424.38	15.10	47.99
	Totals	0.79	884.34		

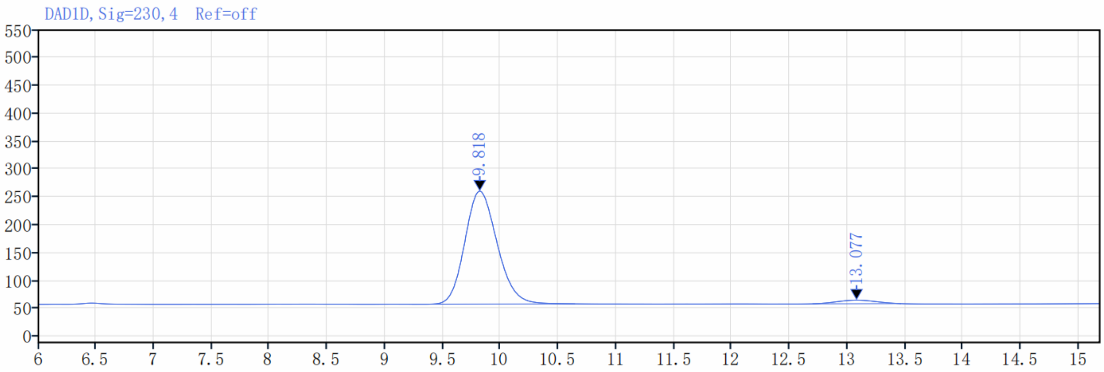

Signal : DAD1D, Sig=230, 4 Ref=off

RetTime [min]	Type	Width [min]	Area [mAU*s]	Height [mAU]	Area%
9.818	MM m	0.30	3937.03	202.50	96.36
13.077	MM m	0.37	148.57	6.43	3.64
	Totals	0.67	4085.60		

Supplementary Figure 102: ^{1}H NMR of 3j (400 MHz, $CDCl_3$)

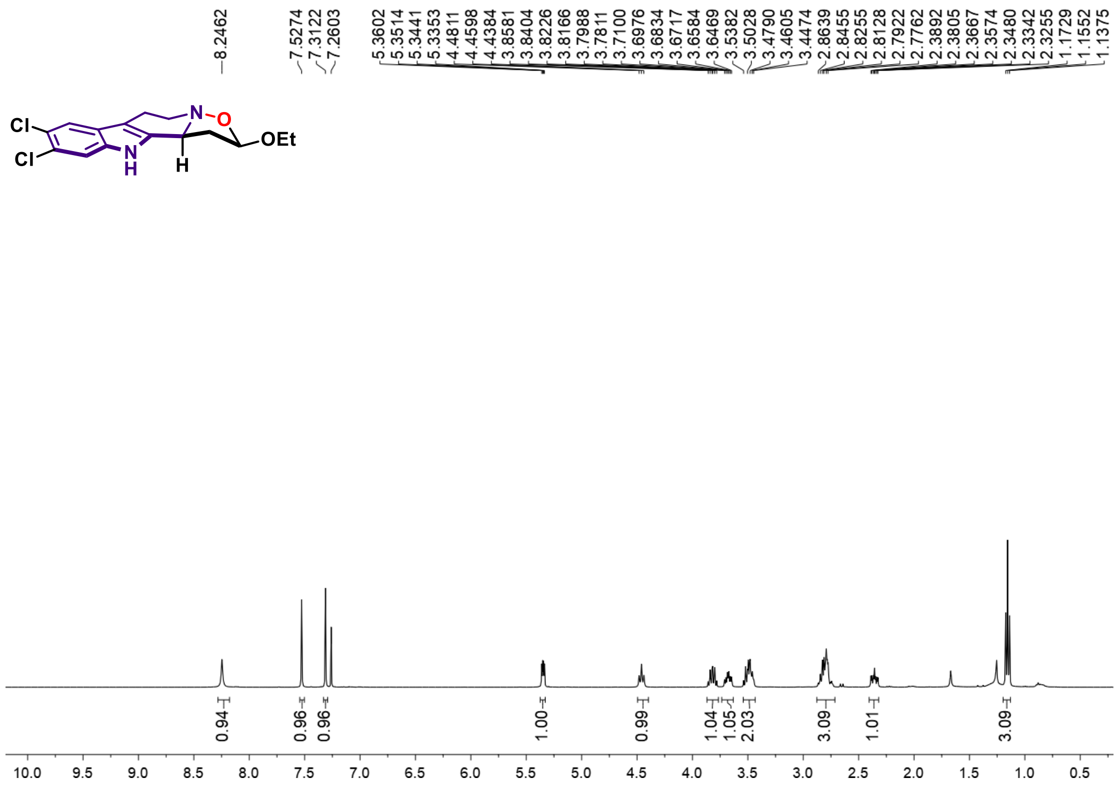

Supplementary Figure 103: ^{13}C NMR of 3j (101 MHz, $CDCl_3$)

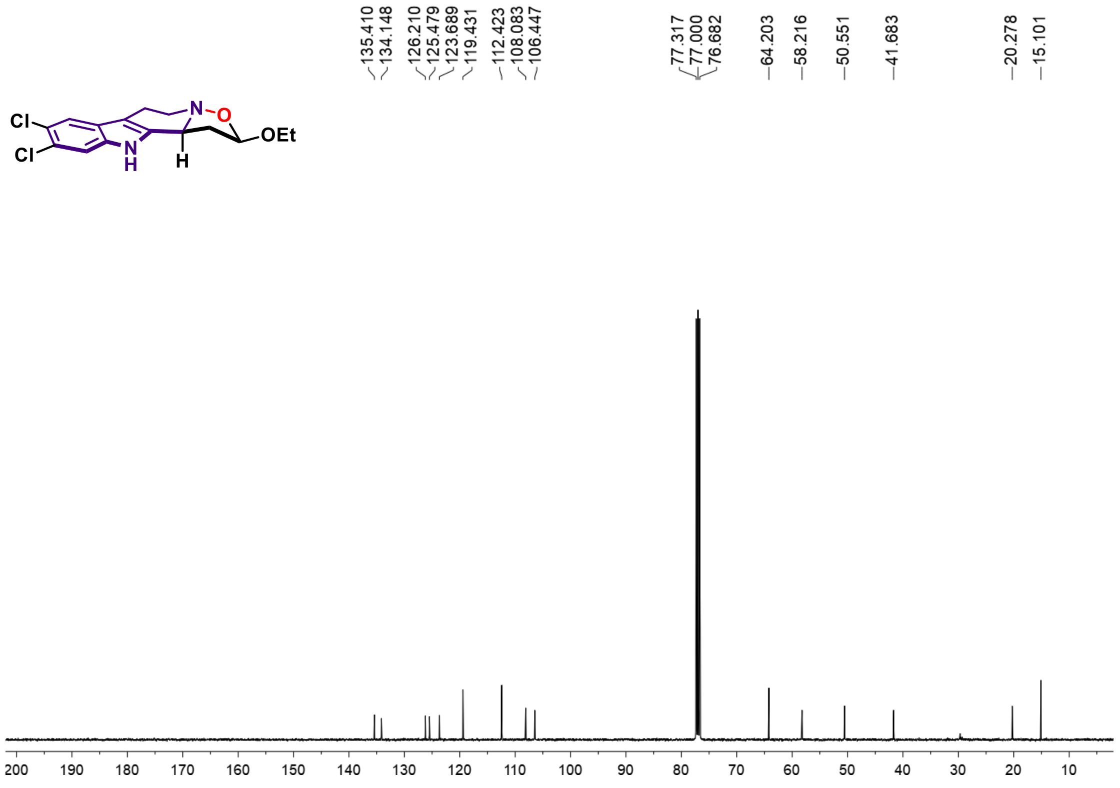

Supplementary Figure 104: HPLC spectrum of 3j

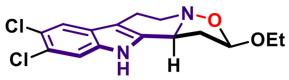

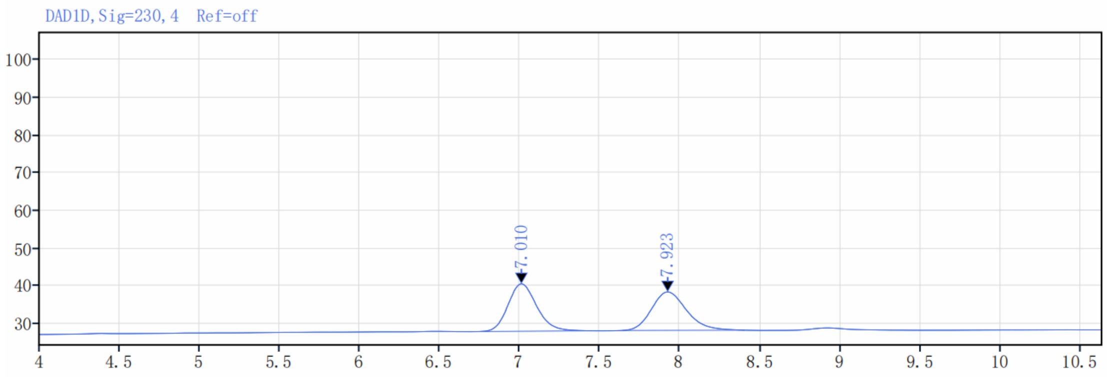

Signal : DAD1D, Sig=230, 4 Ref=off

RetTime [min]	Type	Width [min]	Area [mAU*s]	Height [mAU]	Area%
7.010	MM m	0.19	156.50	12.58	50.35
7.923	MM m	0.23	154.33	10.19	49.65
	Totals	0.42	310.82		

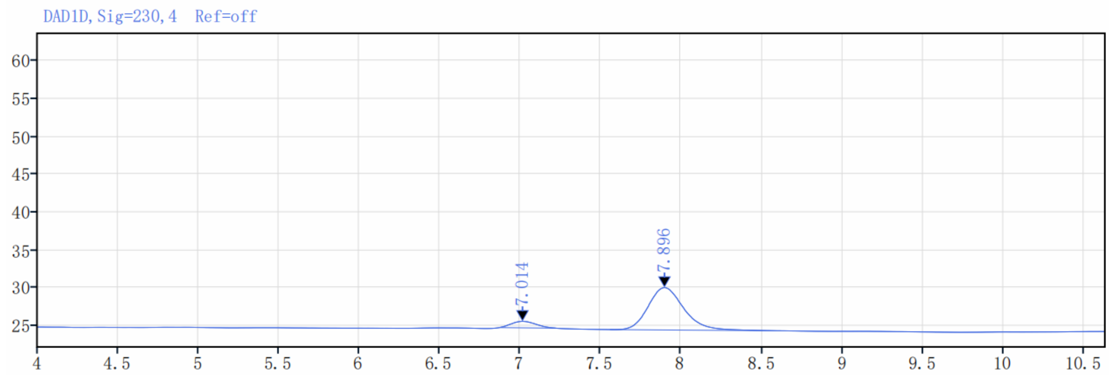

Signal : DAD1D, Sig=230, 4 Ref=off

RetTime [min]	Type	Width [min]	Area [mAU*s]	Height [mAU]	Area%
7.014	MM m	0.17	9.07	0.85	9.53
7.896	MM m	0.24	86.10	5.60	90.47
	Totals	0.40	95.17		

Supplementary Figure 105: ^{1}H NMR of 3k (400 MHz, $CDCl_3$)

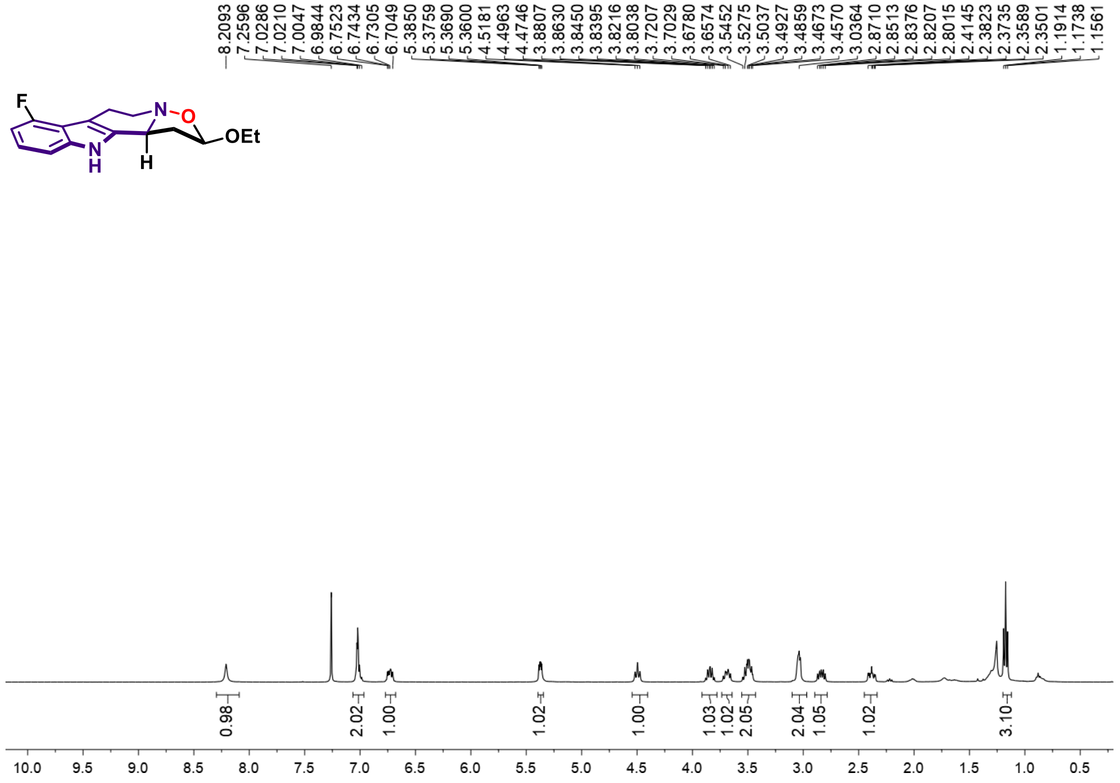

Supplementary Figure 106: ^{13}C NMR of 3k (101 MHz, $CDCl_3$)

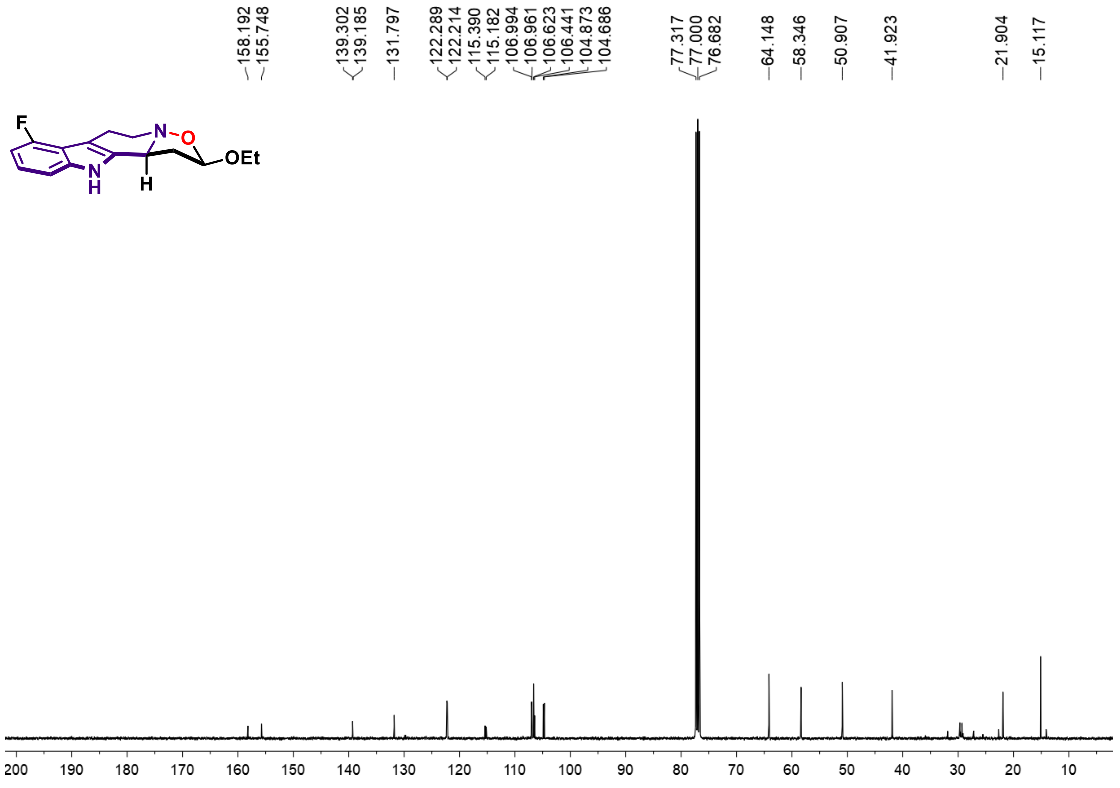

Supplementary Figure 107: HPLC spectrum of 3k

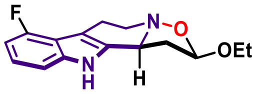

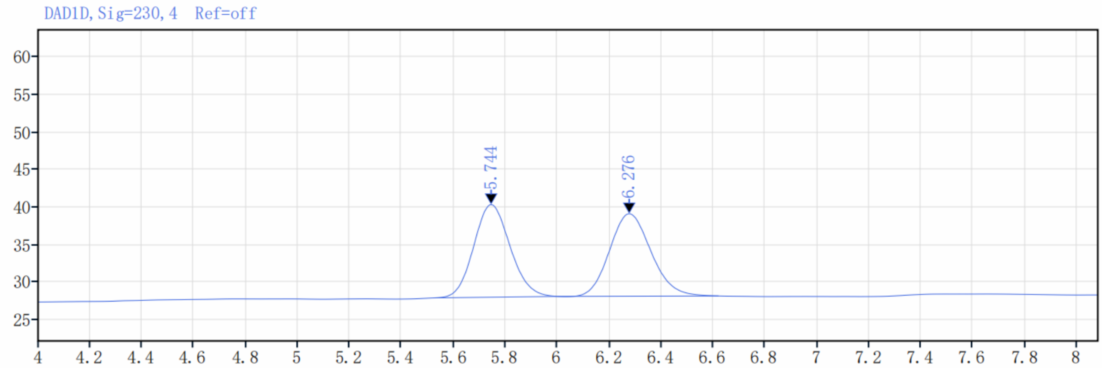

Signal : DAD1D, Sig=230, 4 Ref=off

RetTime [min]	Type	Width [min]	Area [mAU*s]	Height [mAU]	Area%
5.744	MM m	0.15	120.06	12.34	49.66
6.276	MM m	0.17	121.72	10.99	50.34
	Totals	0.32	241.77		

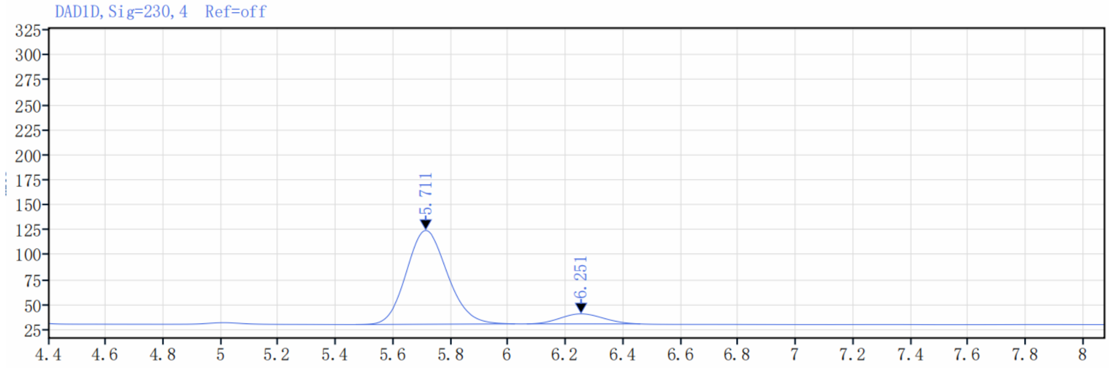

Signal : DAD1D, Sig=230, 4 Ref=off

RetTime [min]	Type	Width [min]	Area [mAU*s]	Height [mAU]	Area%
5.711	MM m	0.15	929.94	93.93	89.80
6.251	MM m	0.16	105.58	10.10	10.20
	Totals	0.32	1035.52		

Supplementary Figure 108: ^{1}H NMR of 3l (400 MHz, CDCl$_3$)

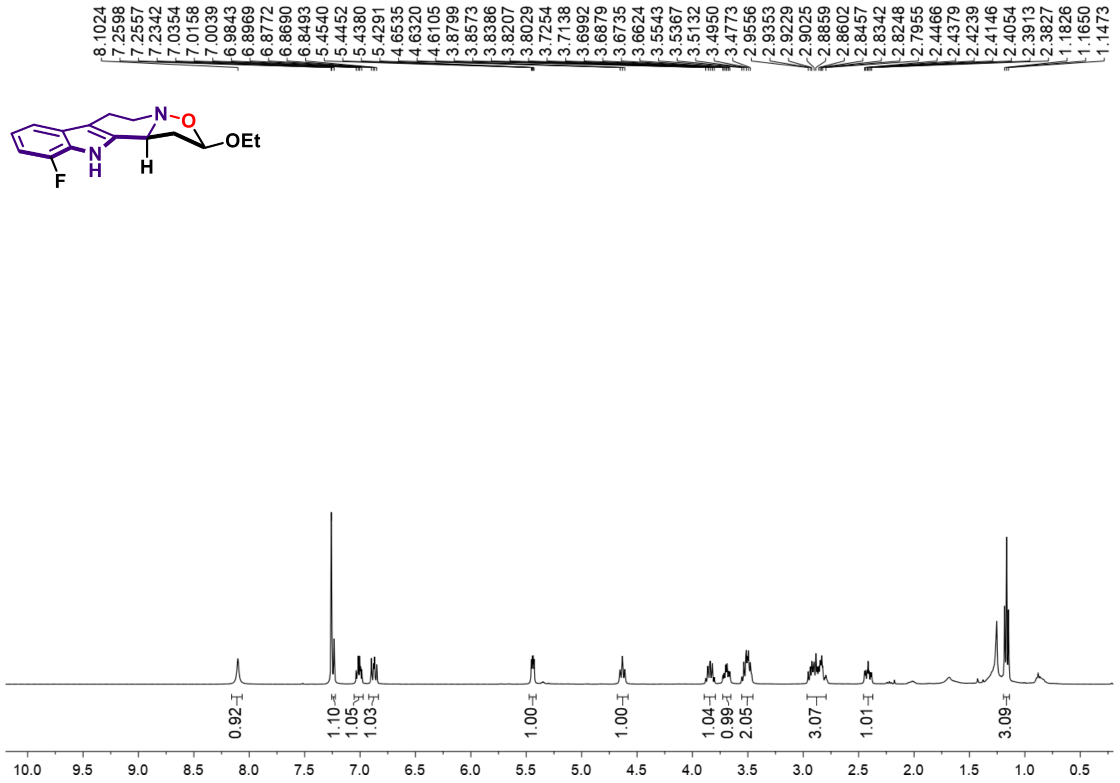

Supplementary Figure 109: ^{13}C NMR of 3l (101 MHz, CDCl$_3$)

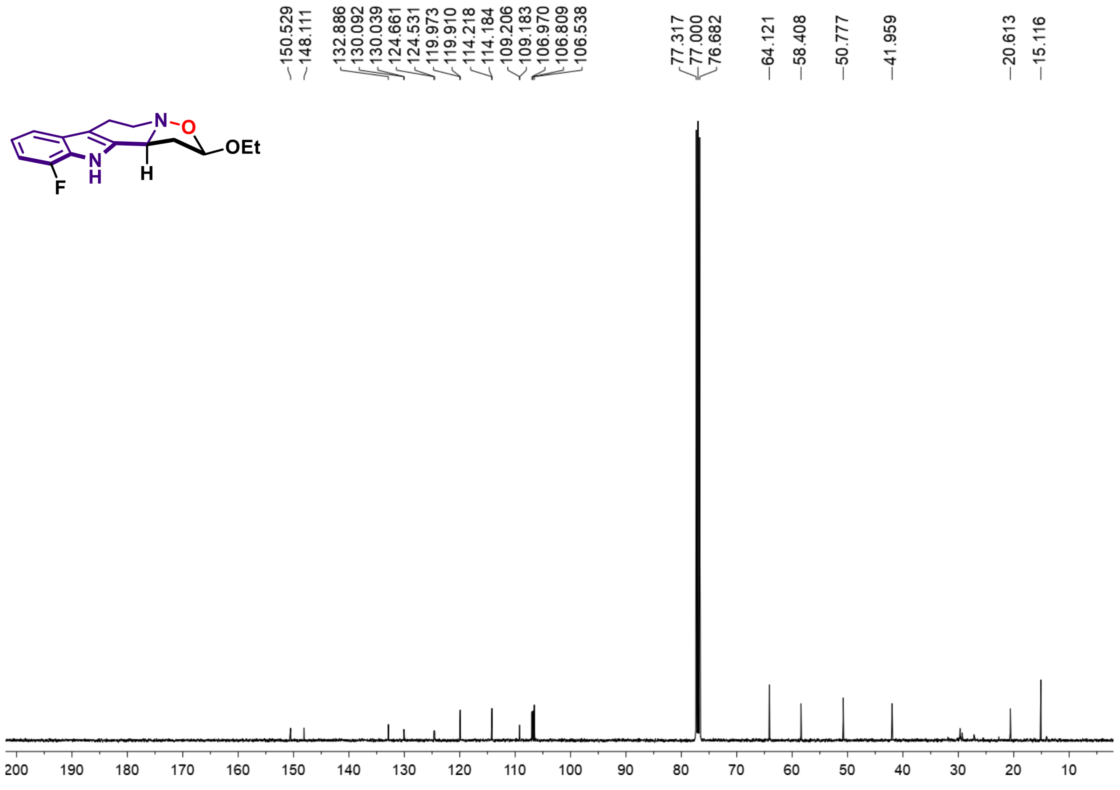

Supplementary Figure 110: HPLC spectrum of 3l

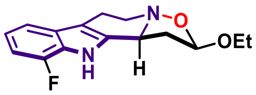

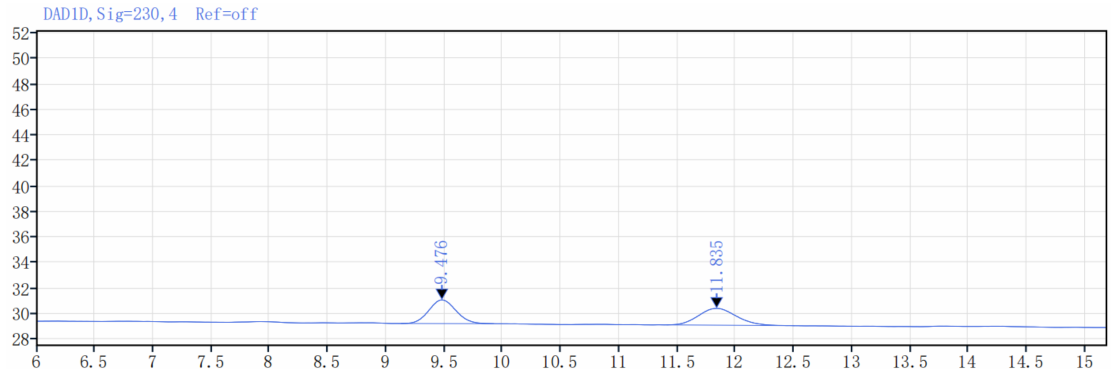

Signal : DAD1D, Sig=230, 4 Ref=off

RetTime [min]	Type	Width [min]	Area [mAU*s]	Height [mAU]	Area%
9.451	MM m	0.24	44.53	3.02	10.25
11.732	MM m	0.34	390.00	17.69	89.75
	Totals	0.58	434.53		

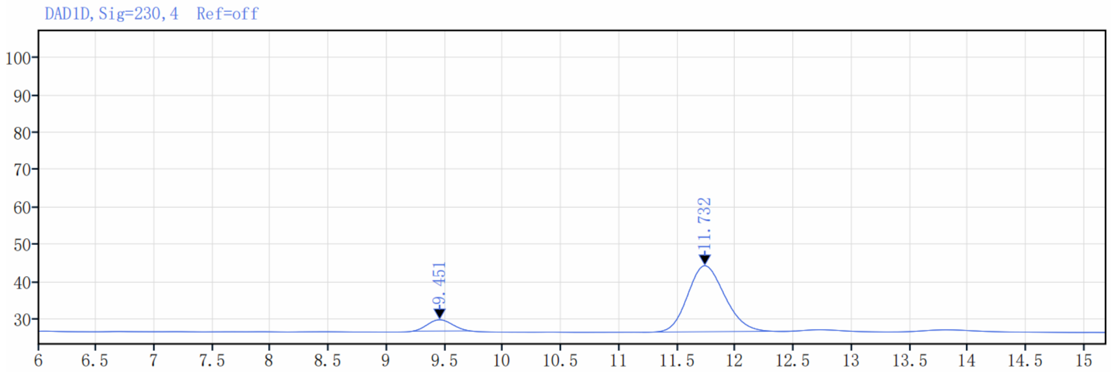

Signal : DAD1D, Sig=230, 4 Ref=off

RetTime [min]	Type	Width [min]	Area [mAU*s]	Height [mAU]	Area%
9.451	MM m	0.24	44.53	3.02	10.25
11.732	MM m	0.34	390.00	17.69	89.75
	Totals	0.58	434.53		

Supplementary Figure 111: ^{1}H NMR of 3m (400 MHz, $CDCl_3$)

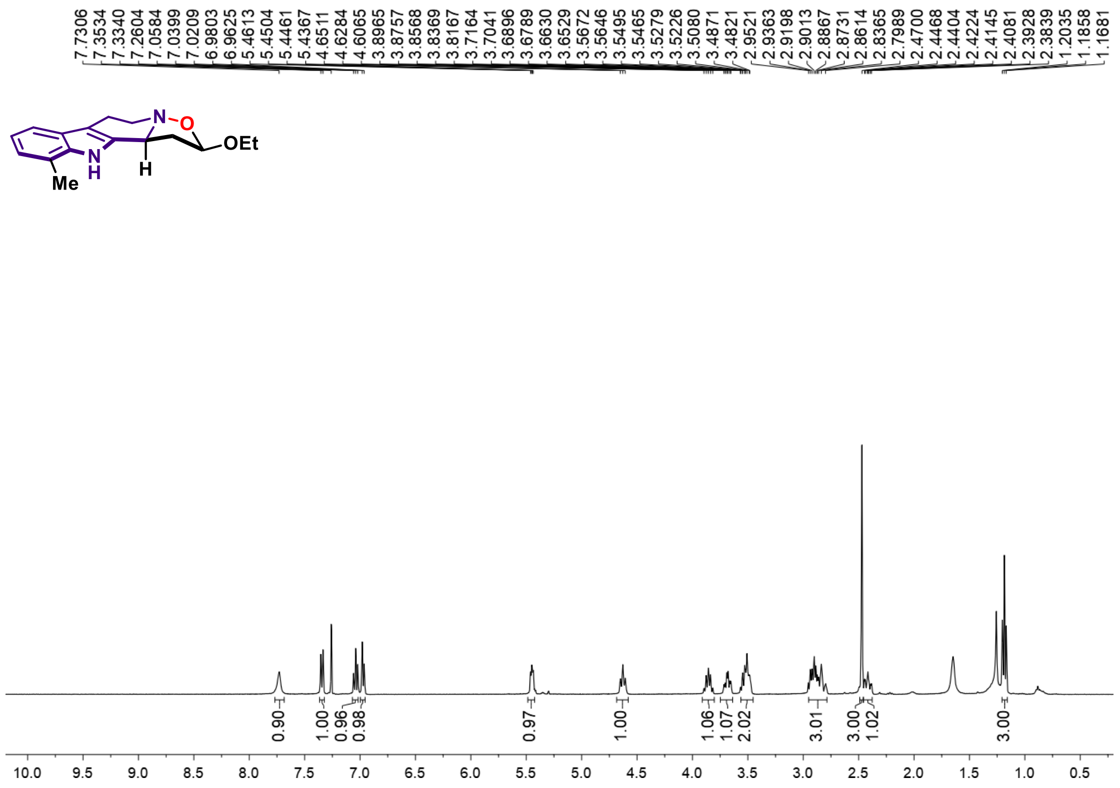

Supplementary Figure 112: ^{13}C NMR of 3m (101 MHz, $CDCl_3$)

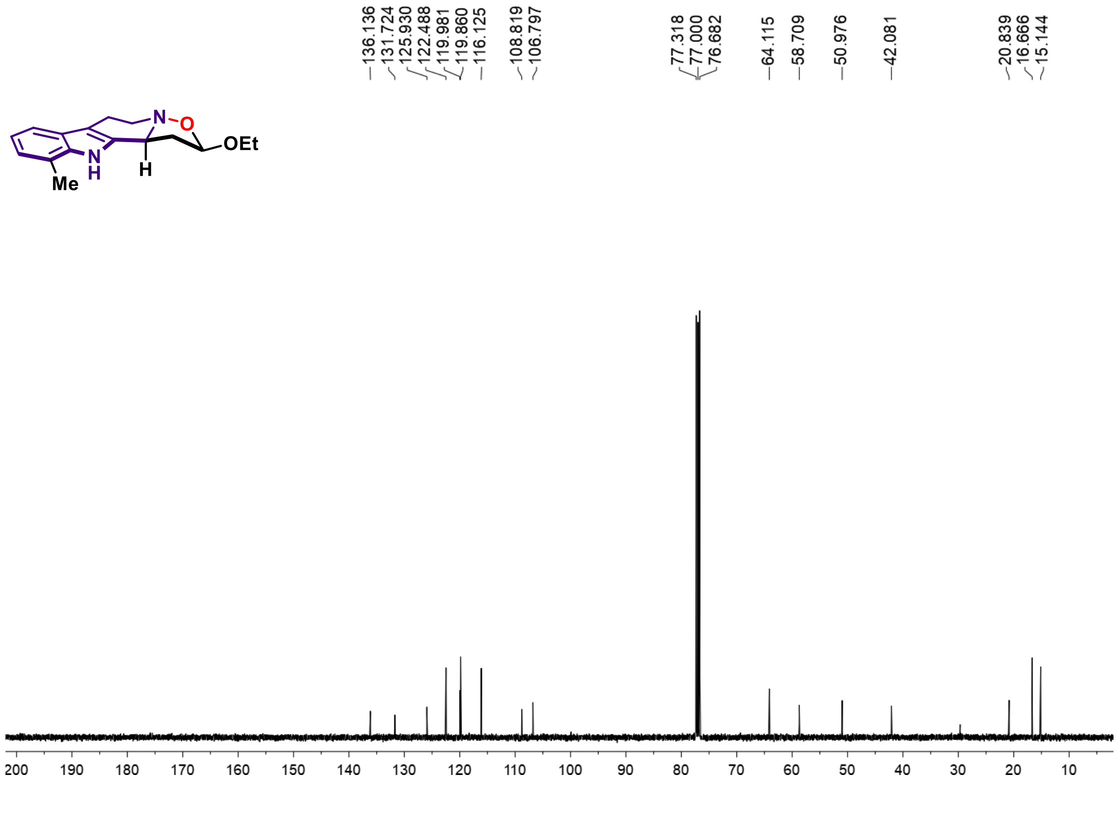

Supplementary Figure 113: HPLC spectrum of 3m

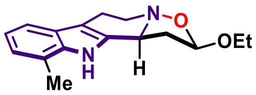

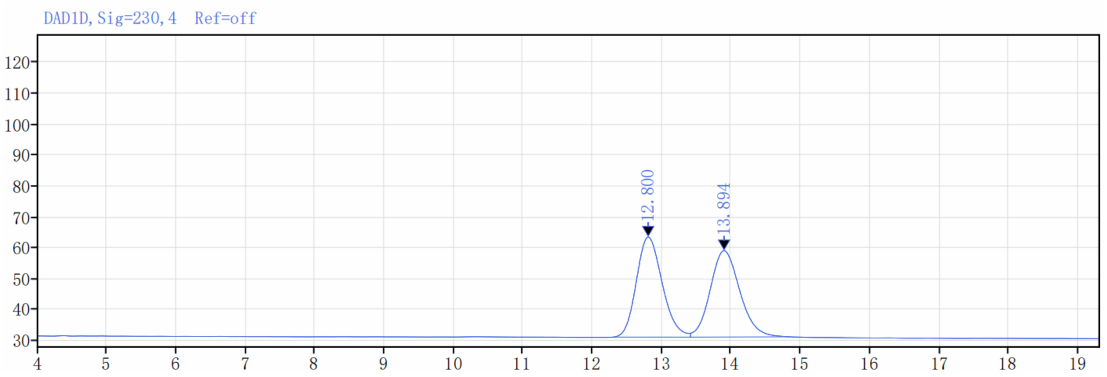

Signal : DAD1D, Sig=230, 4 Ref=off

RetTime [min]	Type	Width [min]	Area [mAU*s]	Height [mAU]	Area%
12.800	MM m	0.40	835.07	32.32	50.27
13.894	MM m	0.46	826.17	27.88	49.73
	Totals	0.86	1661.23		

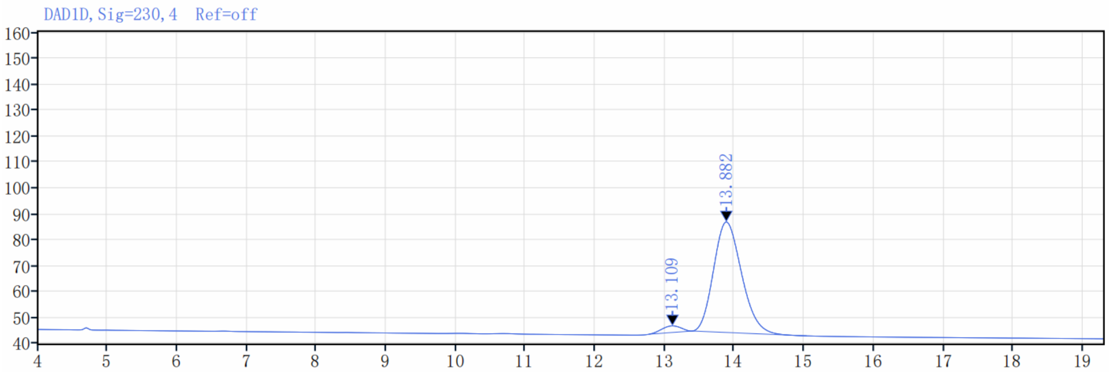

Signal : DAD1D, Sig=230, 4 Ref=off

RetTime [min]	Type	Width [min]	Area [mAU*s]	Height [mAU]	Area%
13.109	MM m	0.32	48.88	2.54	3.94
13.882	MM m	0.43	1192.57	42.68	96.06
	Totals	0.75	1241.45		

Supplementary Figure 114: ^{1}H NMR of 3n (400 MHz, $CDCl_3$)

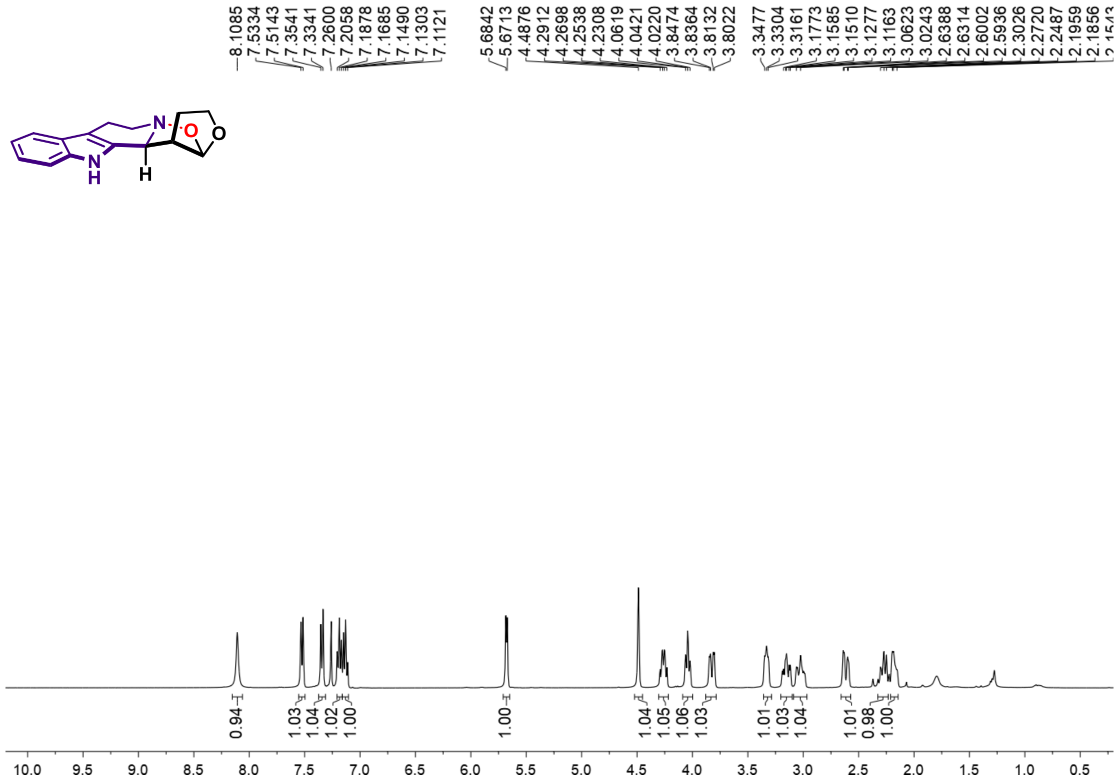

Supplementary Figure 115: ^{13}C NMR of 3n (101 MHz, $CDCl_3$)

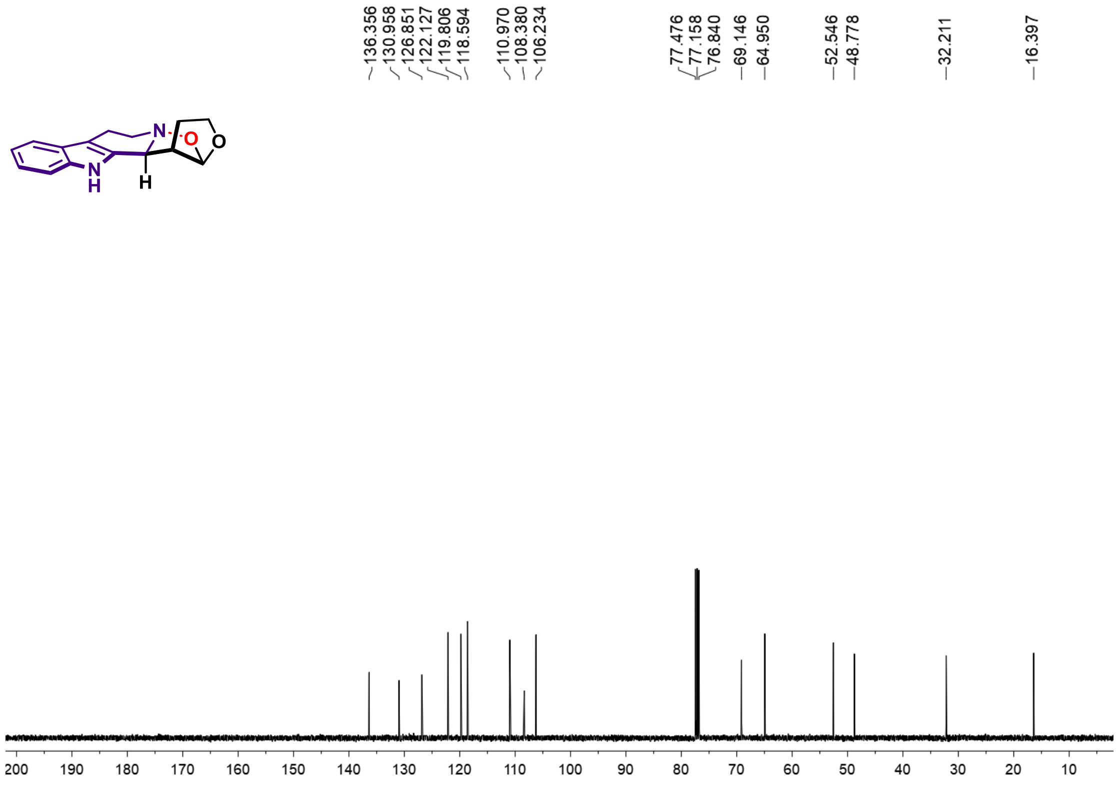

Supplementary Figure 116: HPLC spectrum of 3n

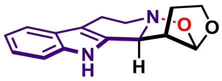

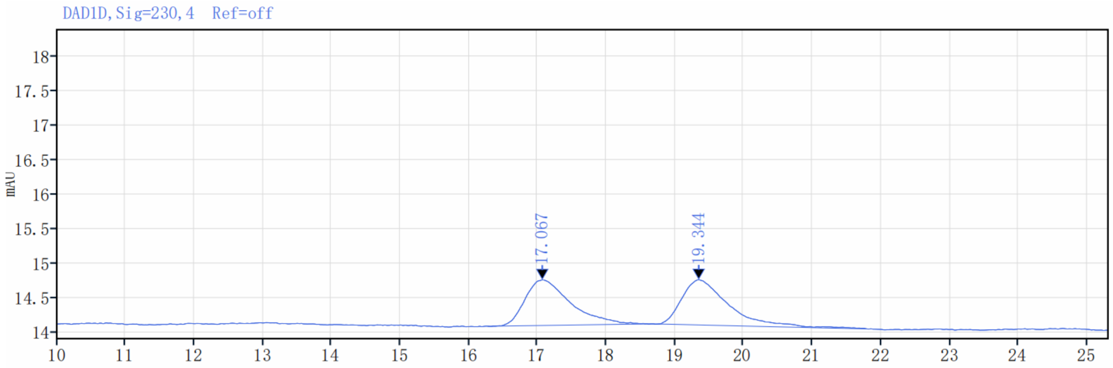

Signal : DAD1D, Sig=230, 4 Ref=off

RetTime [min]	Type	Width [min]	Area [mAU*s]	Height [mAU]	Area%
17.067	MM m	0.54	30.46	0.66	49.50
19.344	MM m	0.56	31.08	0.66	50.50
	Totals	1.11	61.54		

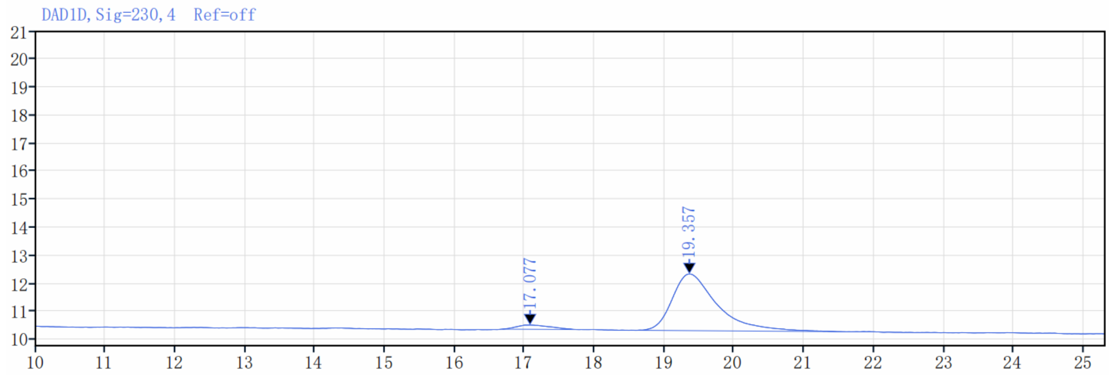

Signal : DAD1D, Sig=230, 4 Ref=off

RetTime [min]	Type	Width [min]	Area [mAU*s]	Height [mAU]	Area%
17.077	MM m	0.40	5.08	0.15	5.32
19.357	MM m	0.61	90.41	2.02	94.68
	Totals	1.01	95.49		

Supplementary Figure 117: ^{1}H NMR of 5a (400 MHz, $CDCl_3$)

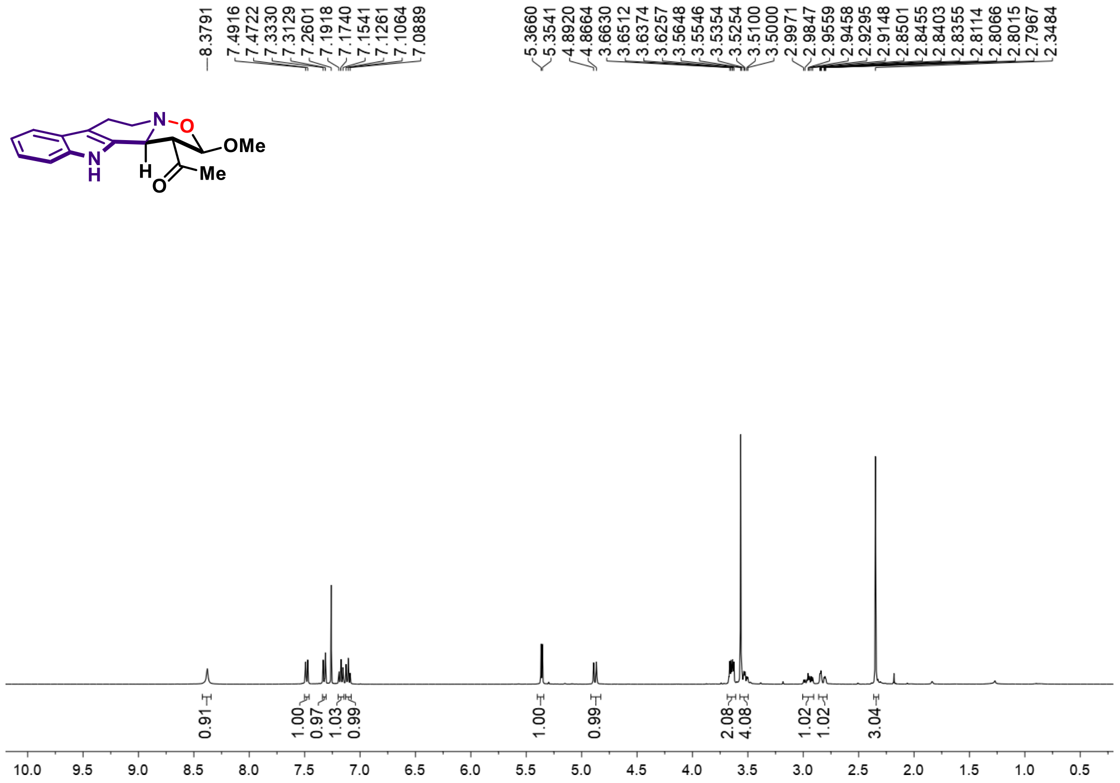

Supplementary Figure 118: ^{13}C NMR of 5a (101 MHz, $CDCl_3$)

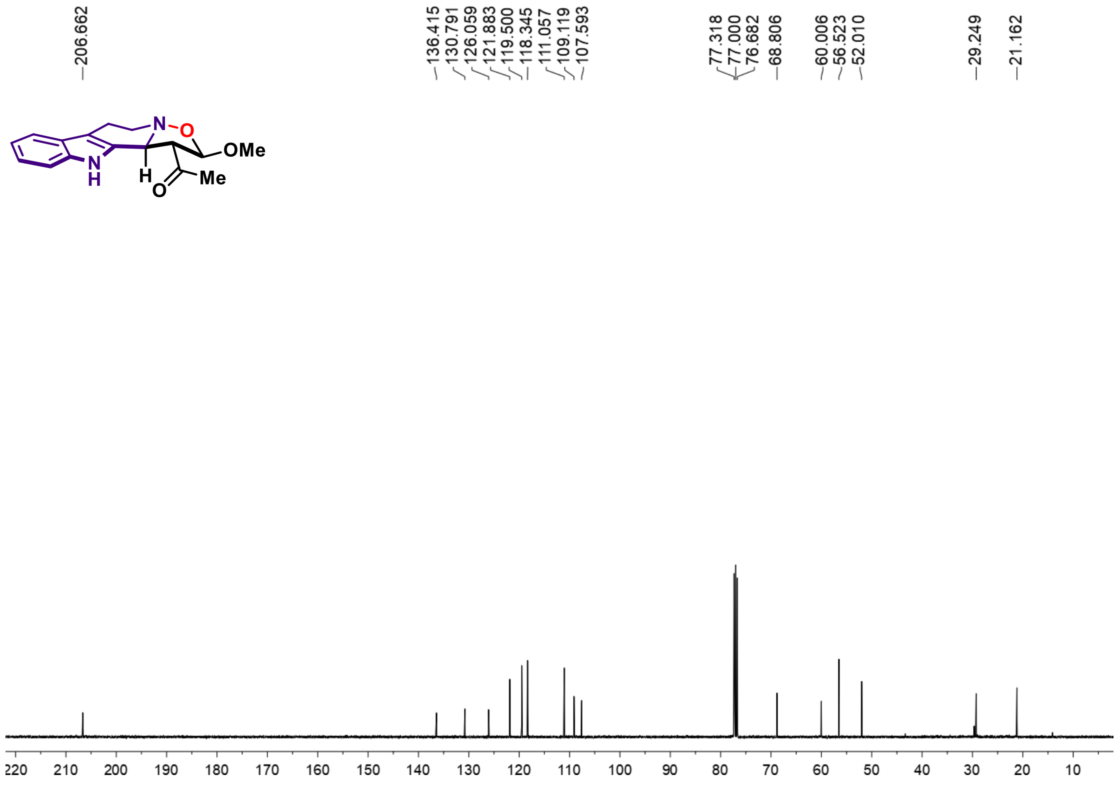

Supplementary Figure 119: HPLC spectrum of 5a

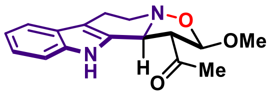

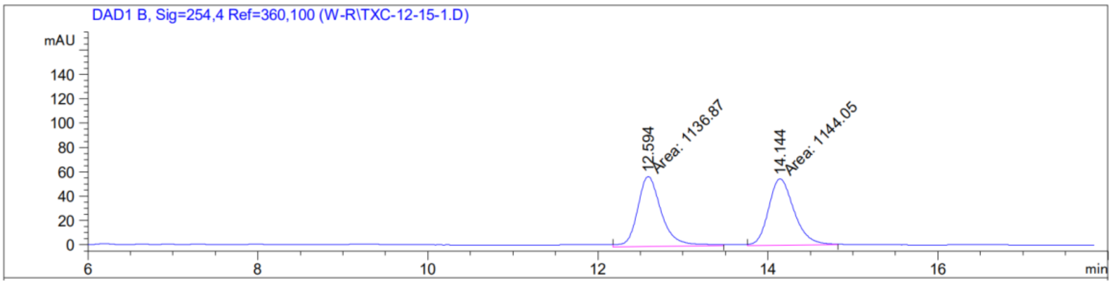

Signal 1: DAD1 B, Sig=254,4 Ref=360,100

Peak #	RetTime [min]	Type	Width [min]	Area [mAU*s]	Height [mAU]	Area %
1	12.594	MM	0.3300	1136.86731	57.42200	49.8426
2	14.144	MM	0.3485	1144.04688	54.72019	50.1574

Totals : 2280.91418 112.14219

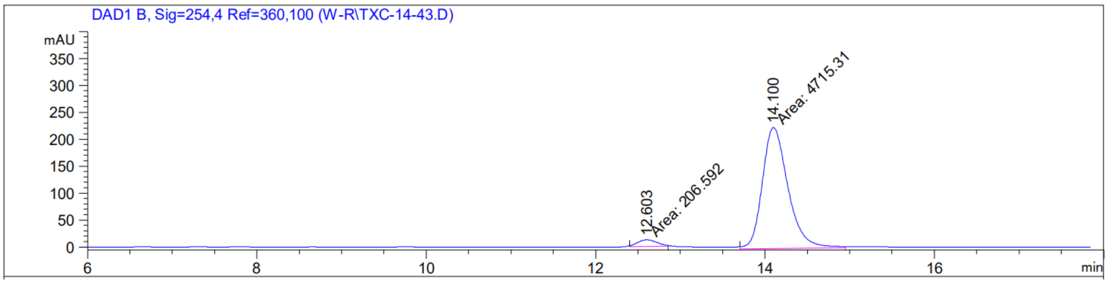

Signal 1: DAD1 B, Sig=254,4 Ref=360,100

Peak #	RetTime [min]	Type	Width [min]	Area [mAU*s]	Height [mAU]	Area %
1	12.603	MM	0.2724	206.59172	12.64013	4.1974
2	14.100	MM	0.3501	4715.31299	224.47800	95.8026

Totals : 4921.90471 237.11813

Supplementary Figure 120: HPLC spectrum of ent-5a

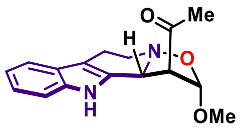

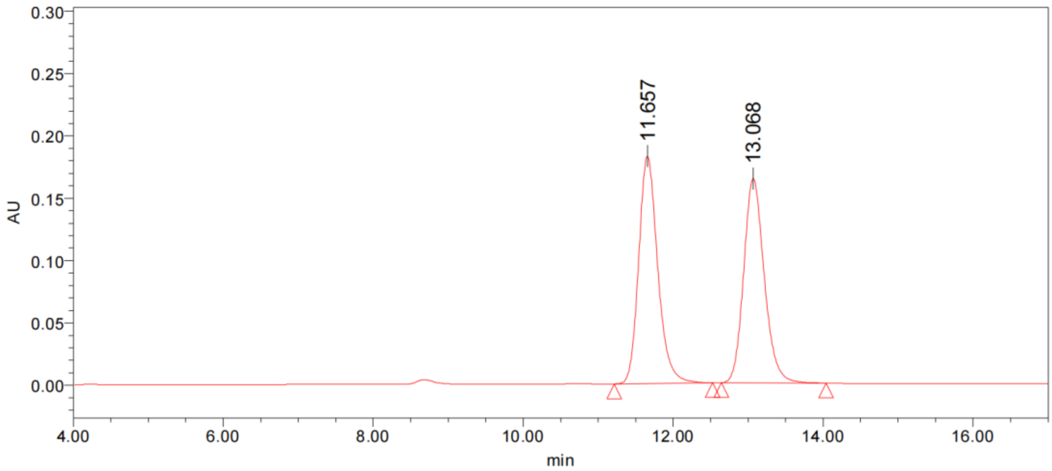

	RetTime [min]	Area [mAU*s]	Area%
1	11.657	3210335	50.62
2	13.068	3132060	49.38

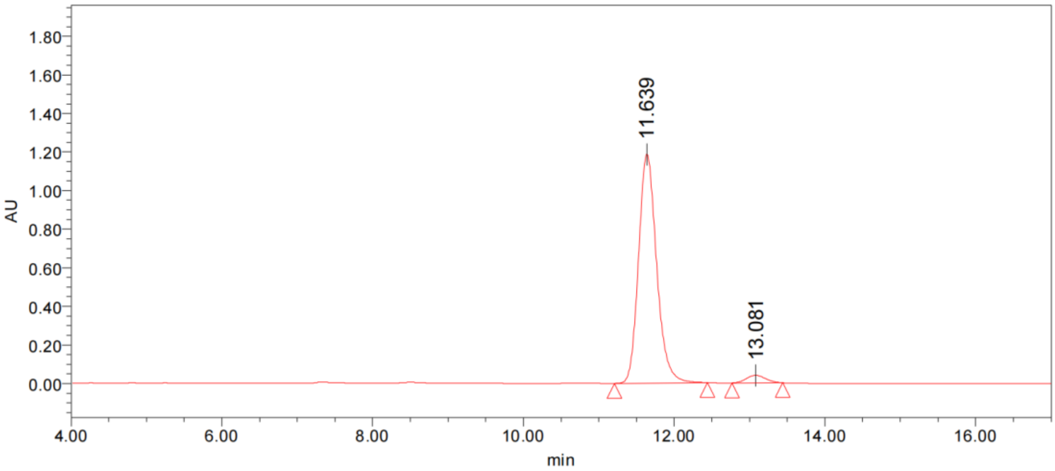

	RetTime [min]	Area [mAU*s]	Area%
1	11.639	19727274	96.60
2	13.081	694036	3.40

Supplementary Figure 121: ^{1}H NMR of 5b (400 MHz, $CDCl_3$)

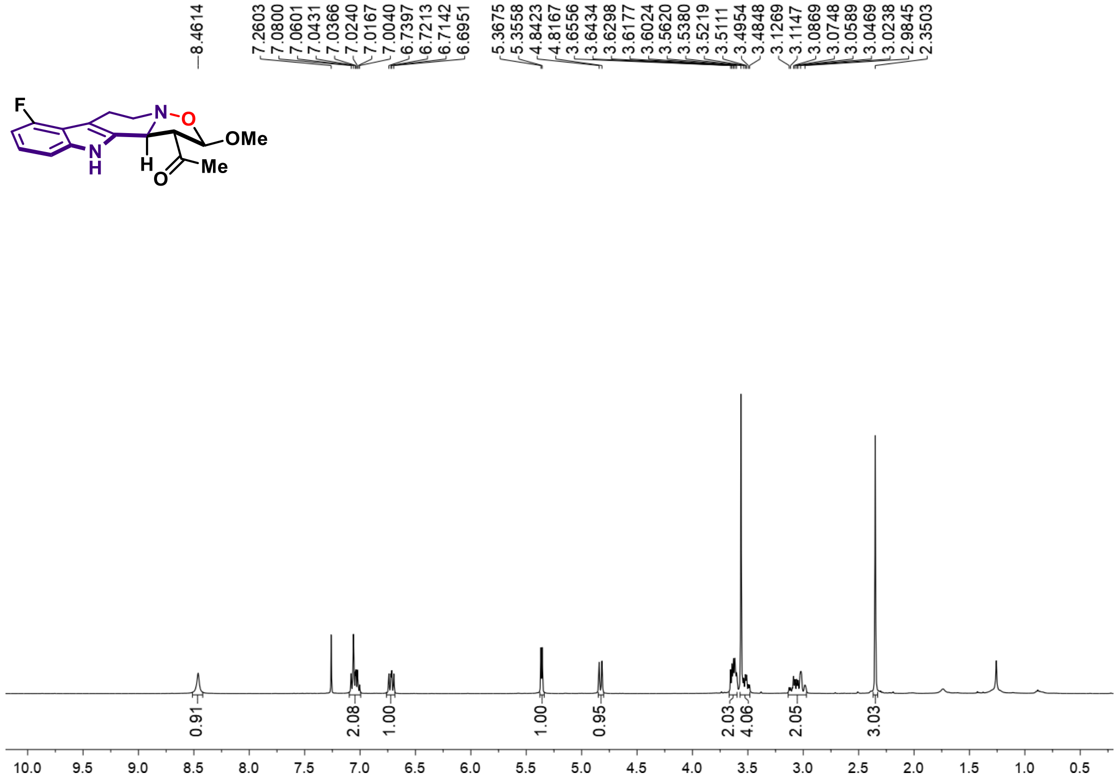

Supplementary Figure 122: ^{13}C NMR of 5b (101 MHz, $CDCl_3$)

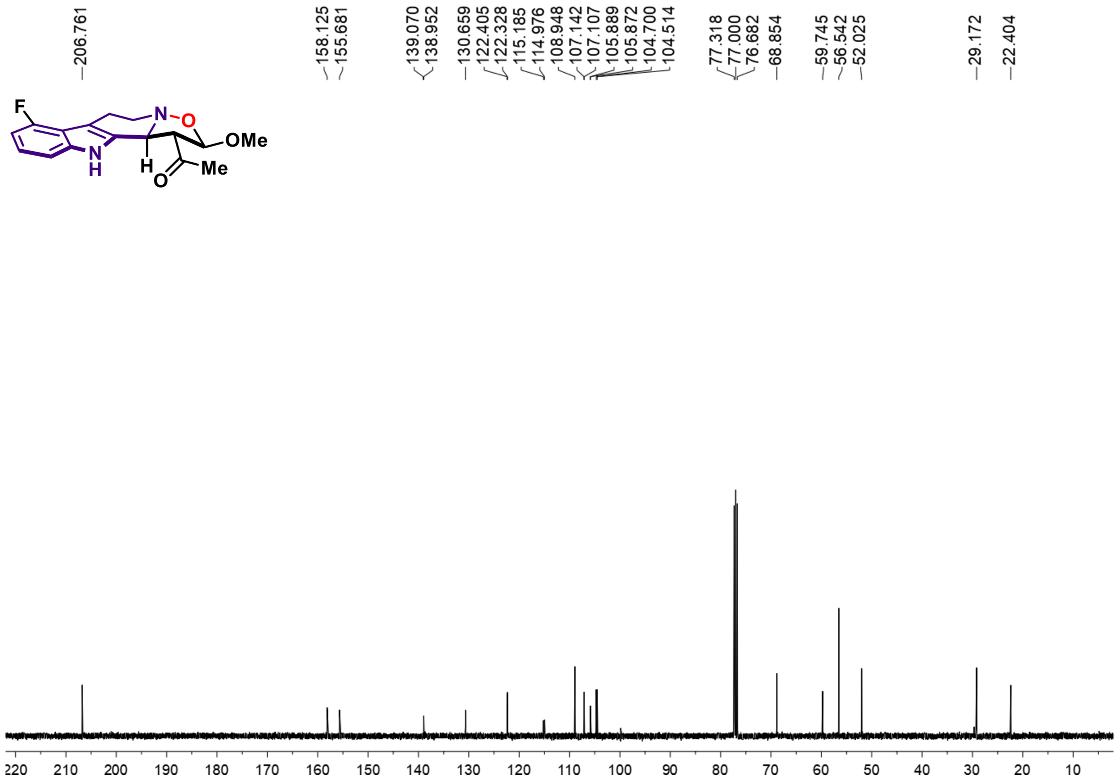

Supplementary Figure 123: HPLC spectrum of 5b

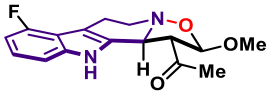

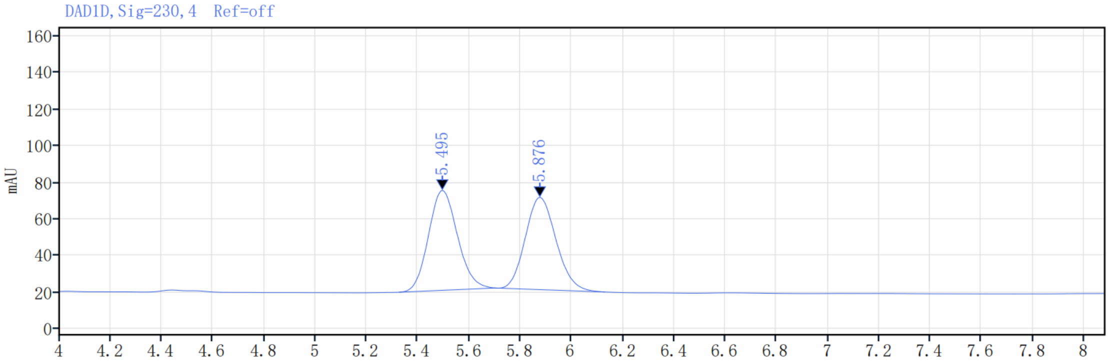

Signal : DAD1D, Sig=230, 4 Ref=off

RetTime [min]	Type	Width [min]	Area [mAU*s]	Height [mAU]	Area%
5.495	MM m	0.12	428.61	54.71	50.48
5.876	MM m	0.13	420.53	50.47	49.52
	Totals	0.25	849.14		

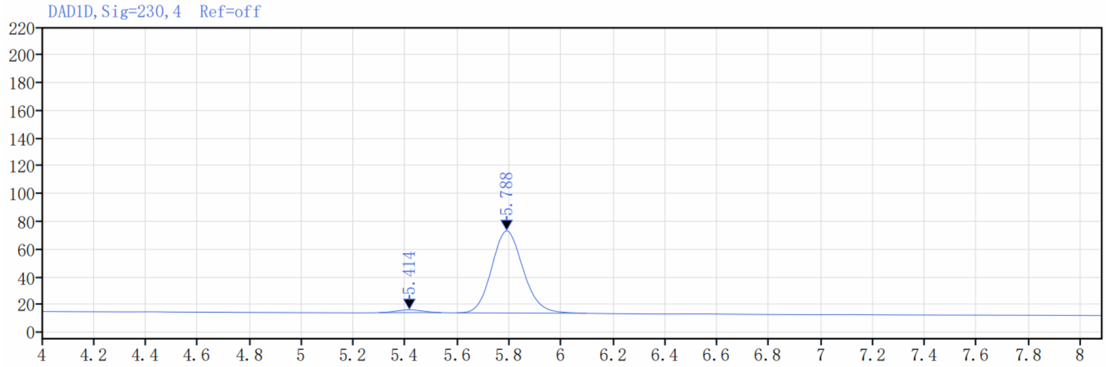

Signal : DAD1D, Sig=230, 4 Ref=off

RetTime [min]	Type	Width [min]	Area [mAU*s]	Height [mAU]	Area%
5.414	MM m	0.11	14.27	2.03	2.79
5.788	MM m	0.13	497.37	59.20	97.21
	Totals	0.24	511.64		

Supplementary Figure 124: ^{1}H NMR of 5c (400 MHz, $CDCl_3$)

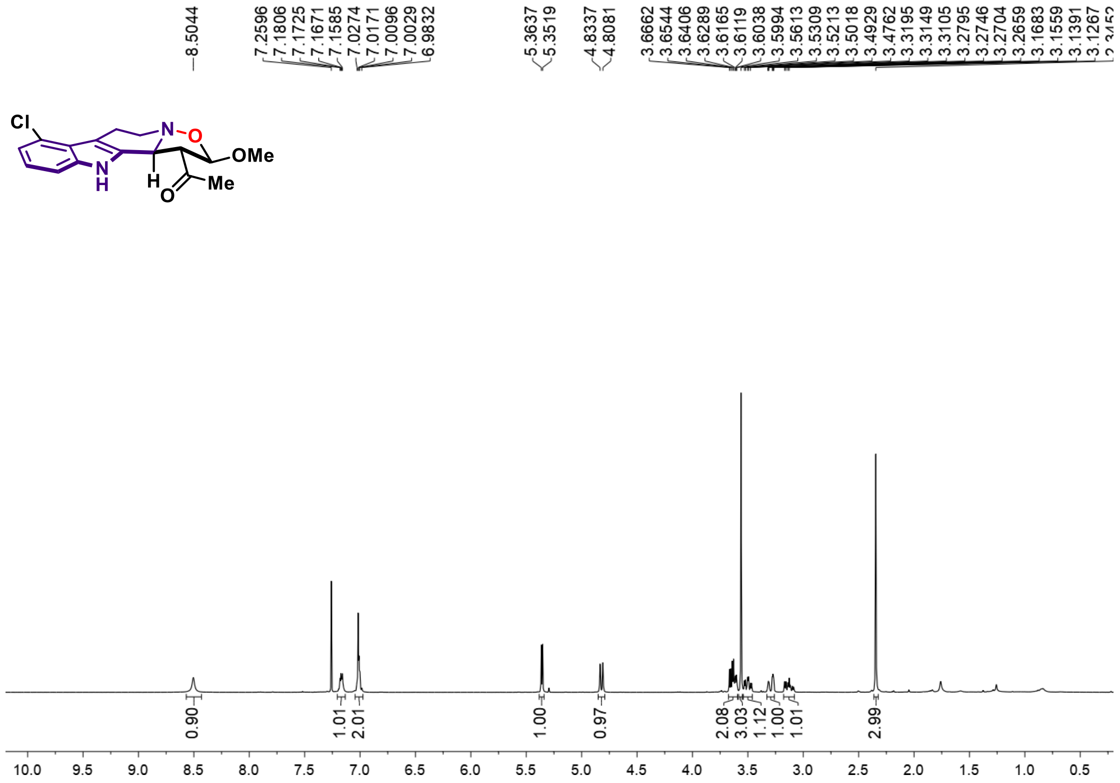

Supplementary Figure 125: ^{13}C NMR of 5c (101 MHz, $CDCl_3$)

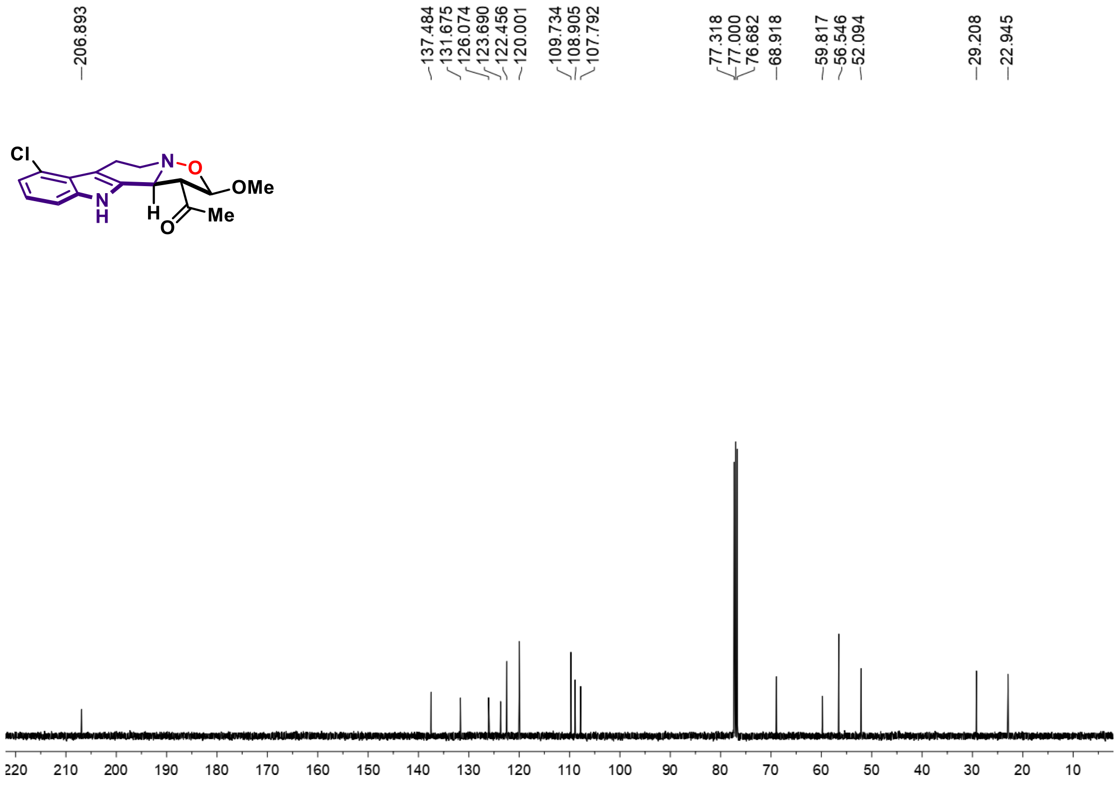

Supplementary Figure 126: HPLC spectrum of 5c

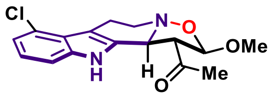

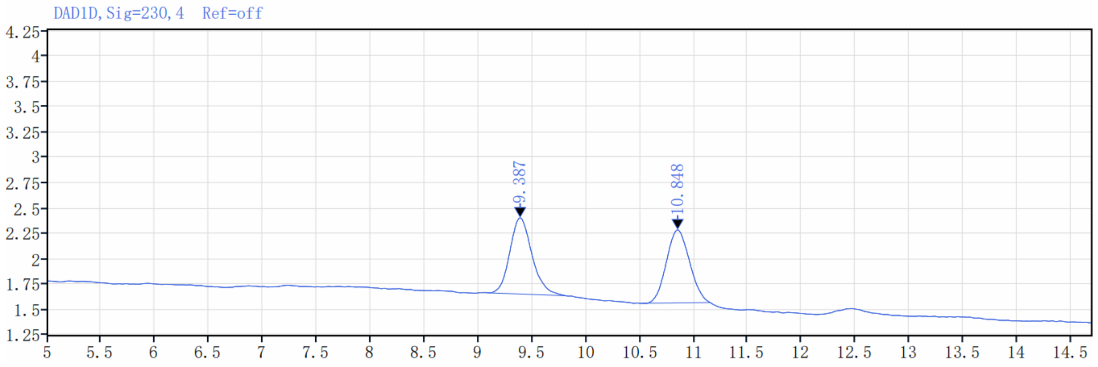

Signal : DAD1D, Sig=230, 4 Ref=off

RetTime [min]	Type	Width [min]	Area [mAU*s]	Height [mAU]	Area%
9.387	MM m	0.22	10.78	0.75	50.02
10.848	MM m	0.23	10.77	0.72	49.98
	Totals	0.45	21.55		

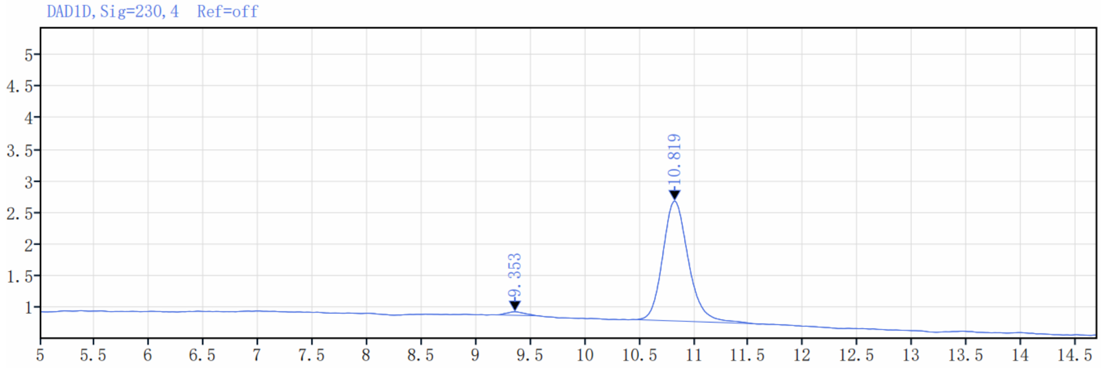

Signal : DAD1D, Sig=230, 4 Ref=off

RetTime [min]	Type	Width [min]	Area [mAU*s]	Height [mAU]	Area%
9.353	MM m	0.14	0.64	0.06	2.00
10.819	MM m	0.25	31.19	1.90	98.00
	Totals	0.39	31.83		

Supplementary Figure 127: ^{1}H NMR of 5d (400 MHz, $CDCl_3$)

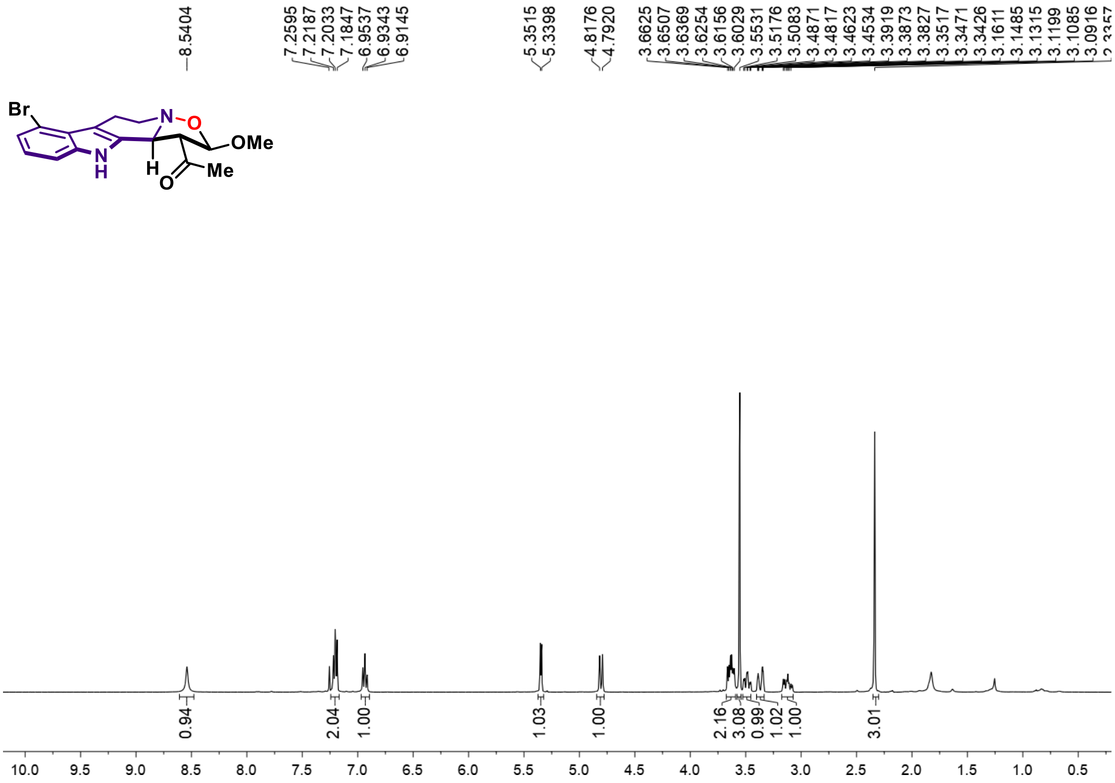

Supplementary Figure 128: ^{13}C NMR of 5d (101 MHz, $CDCl_3$)

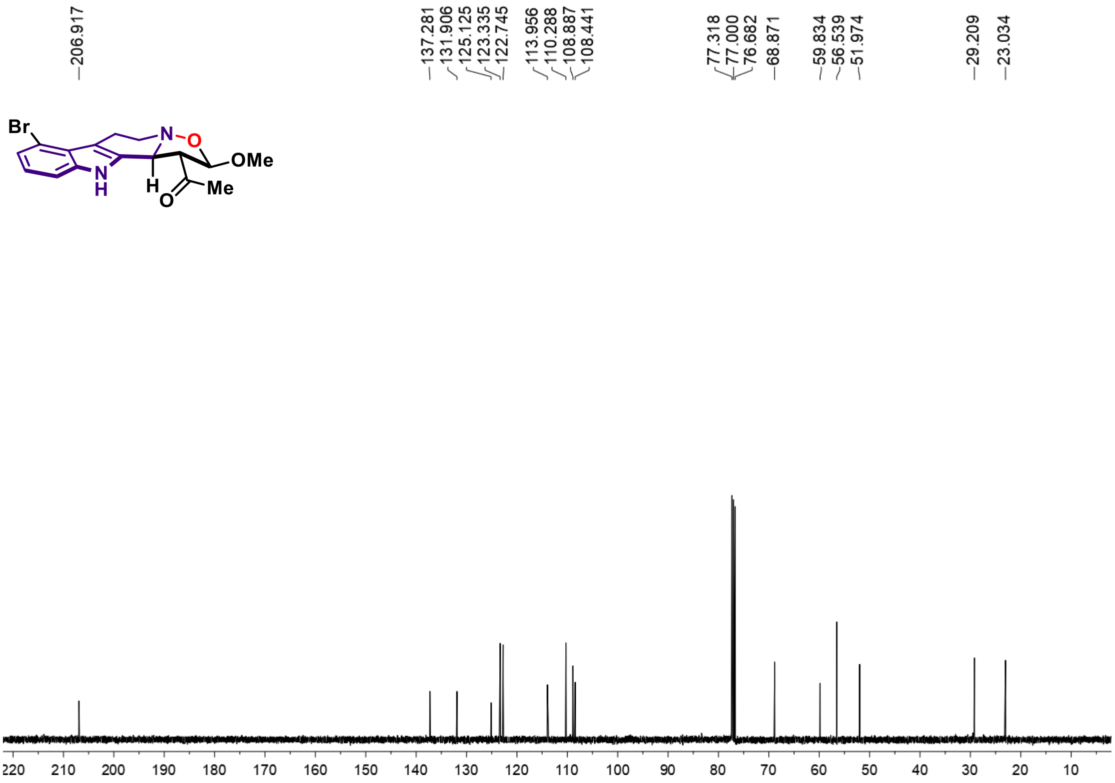

Supplementary Figure 129: HPLC spectrum of 5d

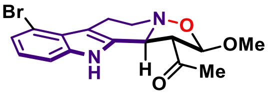

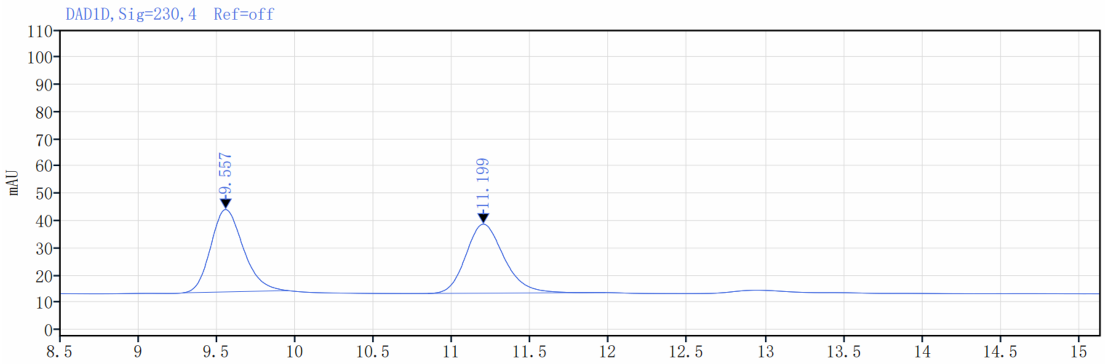

Signal : DAD1D, Sig=230, 4 Ref=off

RetTime [min]	Type	Width [min]	Area [mAU*s]	Height [mAU]	Area%
9.557	MM m	0.22	435.79	30.27	50.46
11.199	MM m	0.26	427.79	25.34	49.54
	Totals	0.48	863.58		

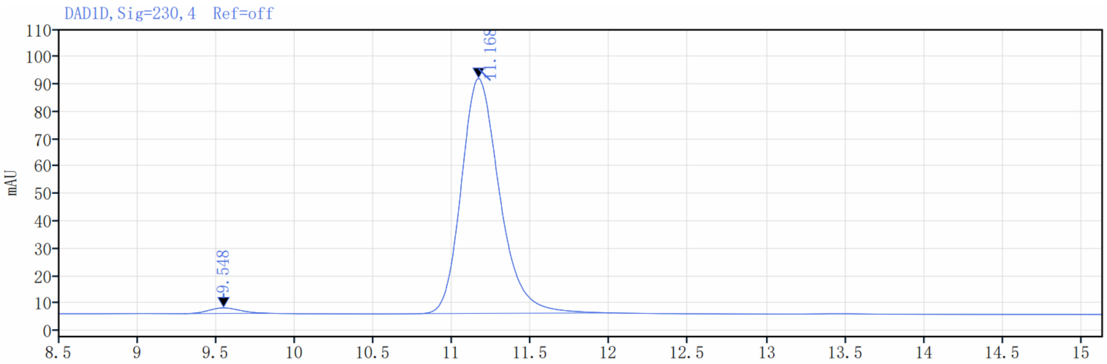

Signal : DAD1D, Sig=230, 4 Ref=off

RetTime [min]	Type	Width [min]	Area [mAU*s]	Height [mAU]	Area%
9.548	MM m	0.21	27.75	2.02	1.88
11.168	MM m	0.26	1447.69	85.76	98.12
	Totals	0.47	1475.44		

Supplementary Figure 130: ^{1}H NMR of 5e (400 MHz, $CDCl_3$)

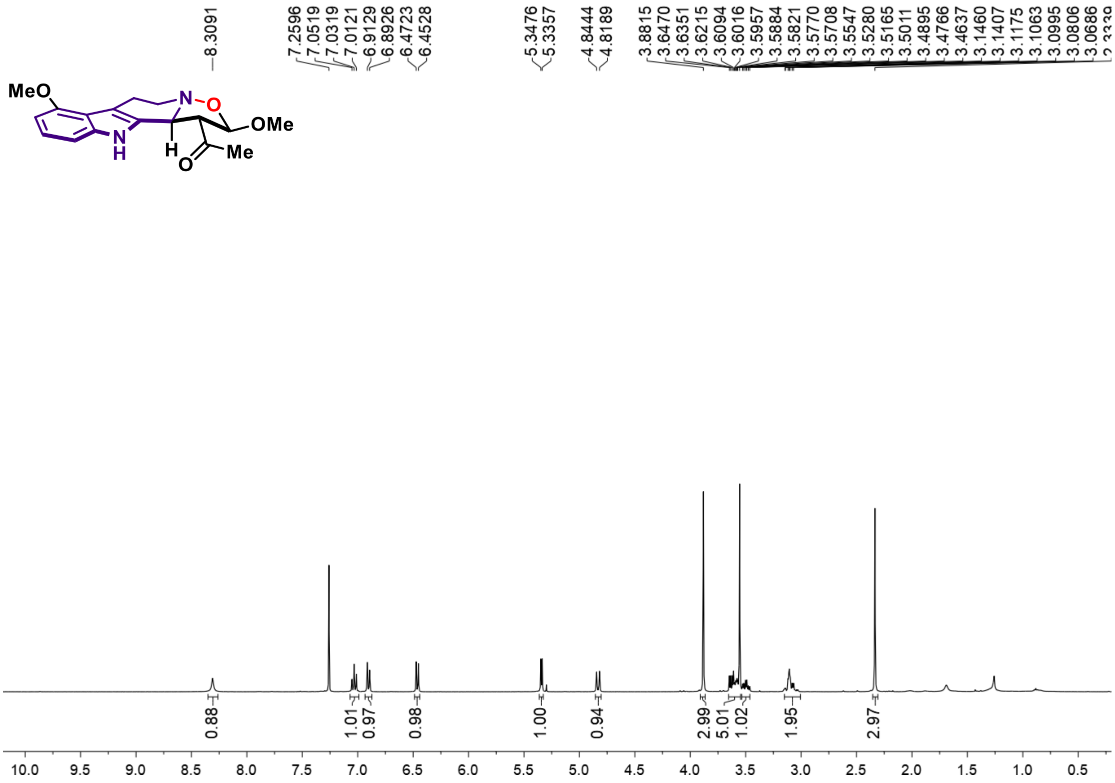

Supplementary Figure 131: ^{13}C NMR of 5e (101 MHz, $CDCl_3$)

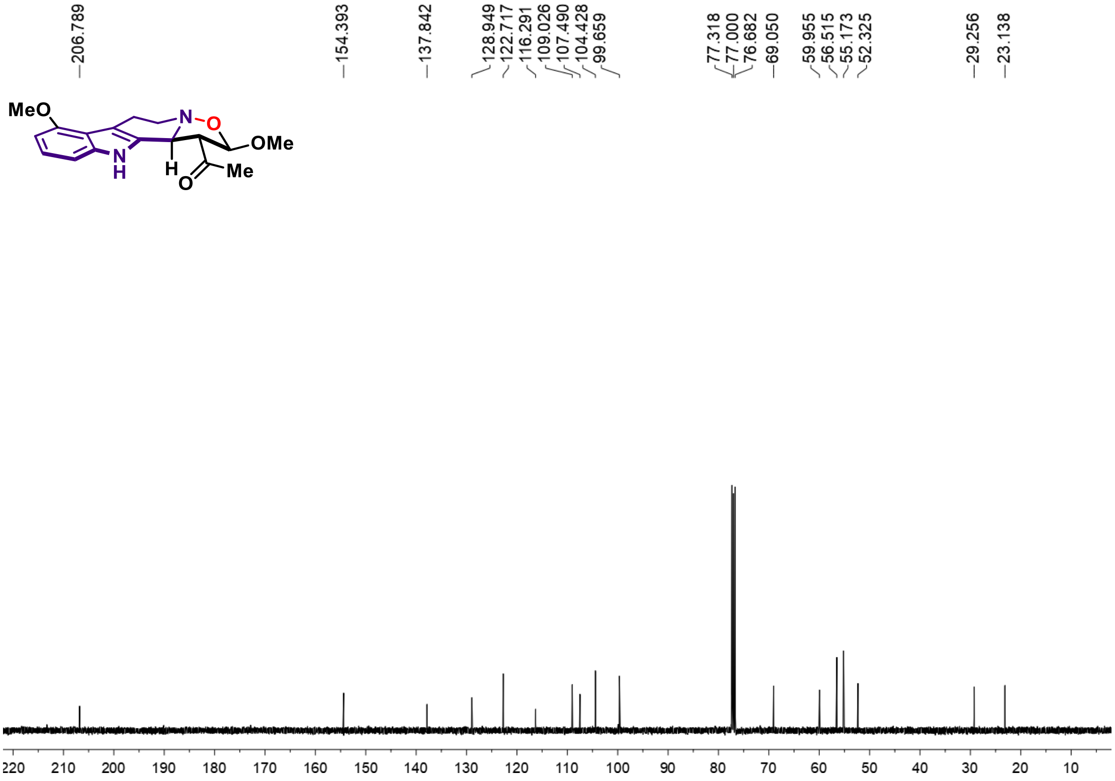

Supplementary Figure 132: HPLC spectrum of 5e

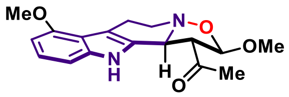

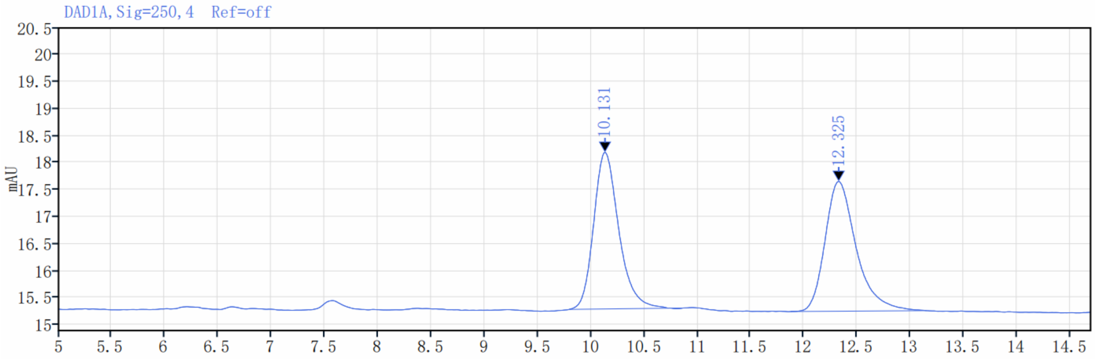

Signal : DAD1A, Sig=250, 4 Ref=off

RetTime [min]	Type	Width [min]	Area [mAU*s]	Height [mAU]	Area%
10.131	MM m	0.25	47.49	2.90	49.28
12.325	MM m	0.31	48.87	2.40	50.72
	Totals	0.55	96.36		

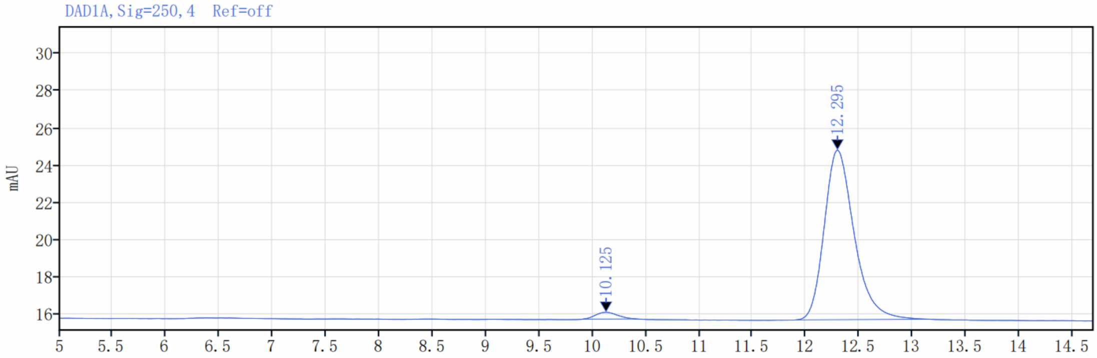

Signal : DAD1A, Sig=250, 4 Ref=off

RetTime [min]	Type	Width [min]	Area [mAU*s]	Height [mAU]	Area%
10.125	MM m	0.21	5.23	0.36	2.87
12.295	MM m	0.30	177.13	9.08	97.13
	Totals	0.50	182.36		

Supplementary Figure 133: ^{1}H NMR of 5f (400 MHz, $CDCl_3$)

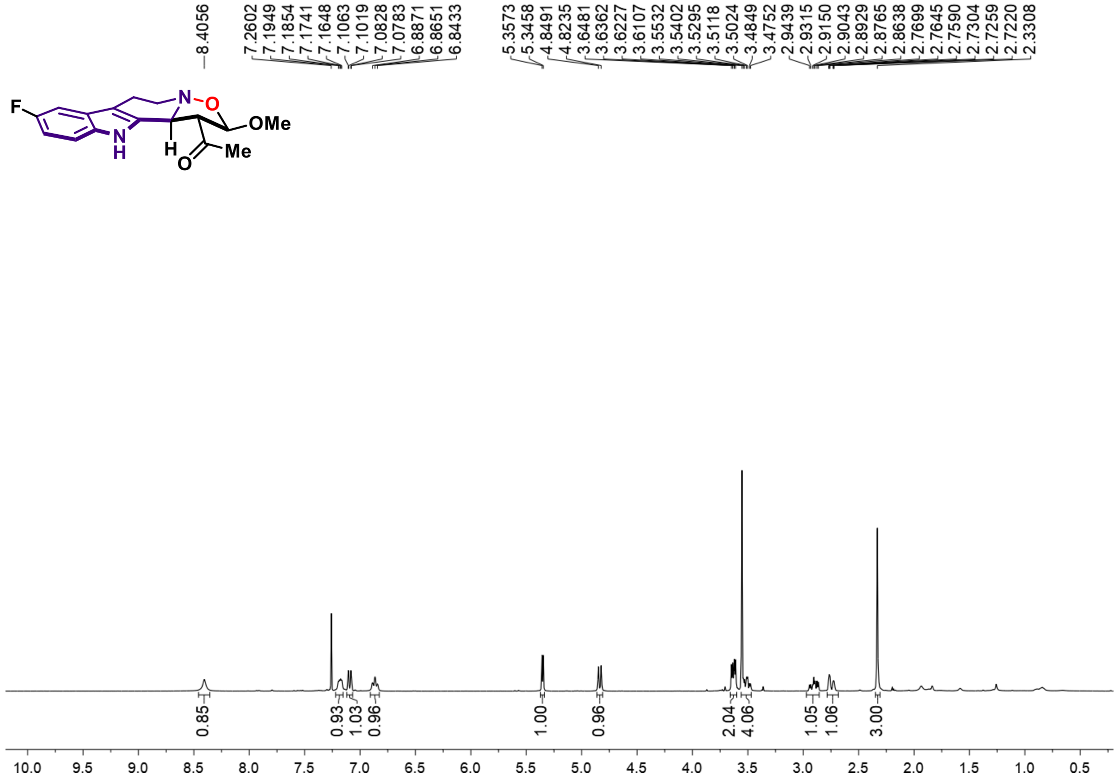

Supplementary Figure 134: ^{13}C NMR of 5f (101 MHz, $CDCl_3$)

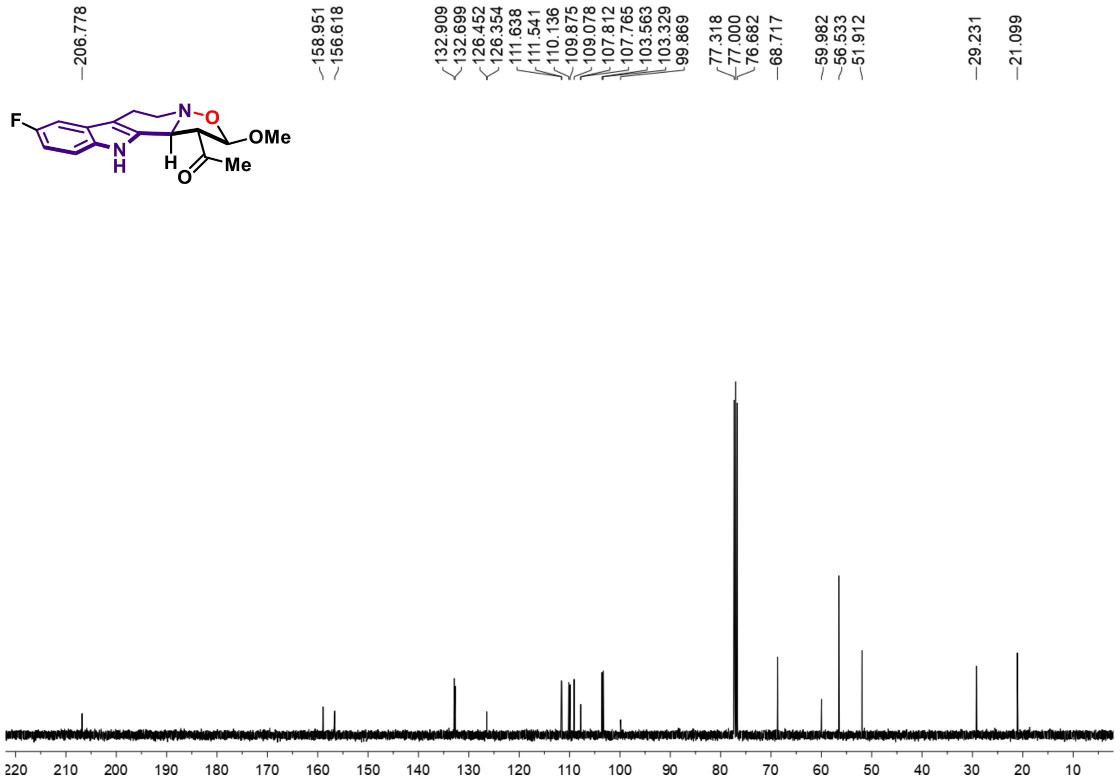

Supplementary Figure 135: HPLC spectrum of 5f

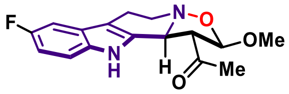

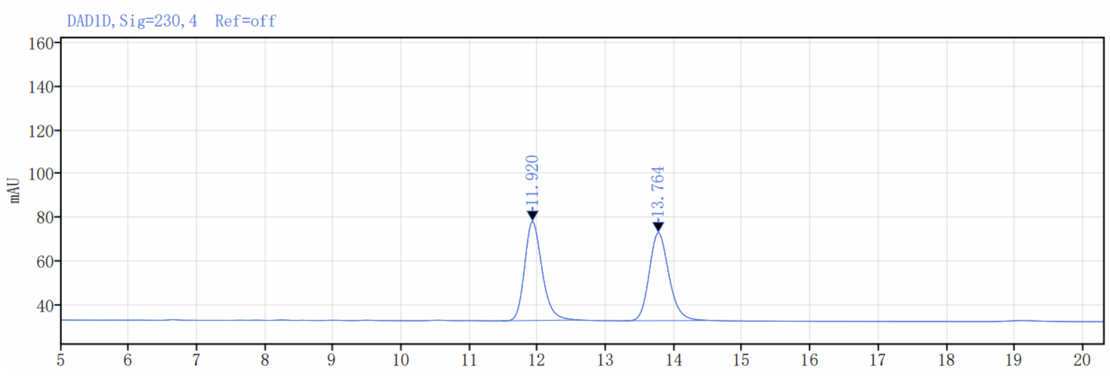

Signal : DAD1D, Sig=230, 4 Ref=off

RetTime [min]	Type	Width [min]	Area [mAU*s]	Height [mAU]	Area%
11.920	MM m	0.27	802.16	45.20	49.99
13.764	MM m	0.31	802.53	40.11	50.01
	Totals	0.58	1604.69		

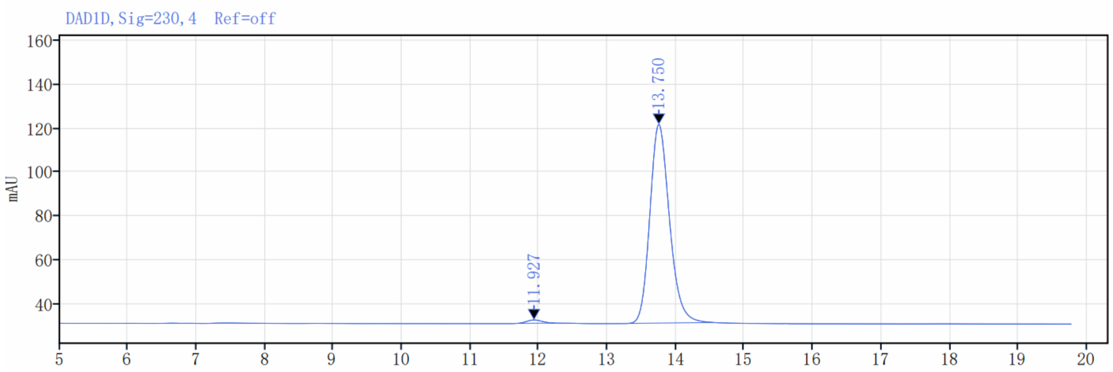

Signal : DAD1D, Sig=230, 4 Ref=off

RetTime [min]	Type	Width [min]	Area [mAU*s]	Height [mAU]	Area%
11.927	MM m	0.25	23.36	1.50	1.28
13.750	MM m	0.31	1798.07	90.30	98.72
	Totals	0.55	1821.43		

Supplementary Figure 136: ^{1}H NMR of 5g (400 MHz, $CDCl_3$)

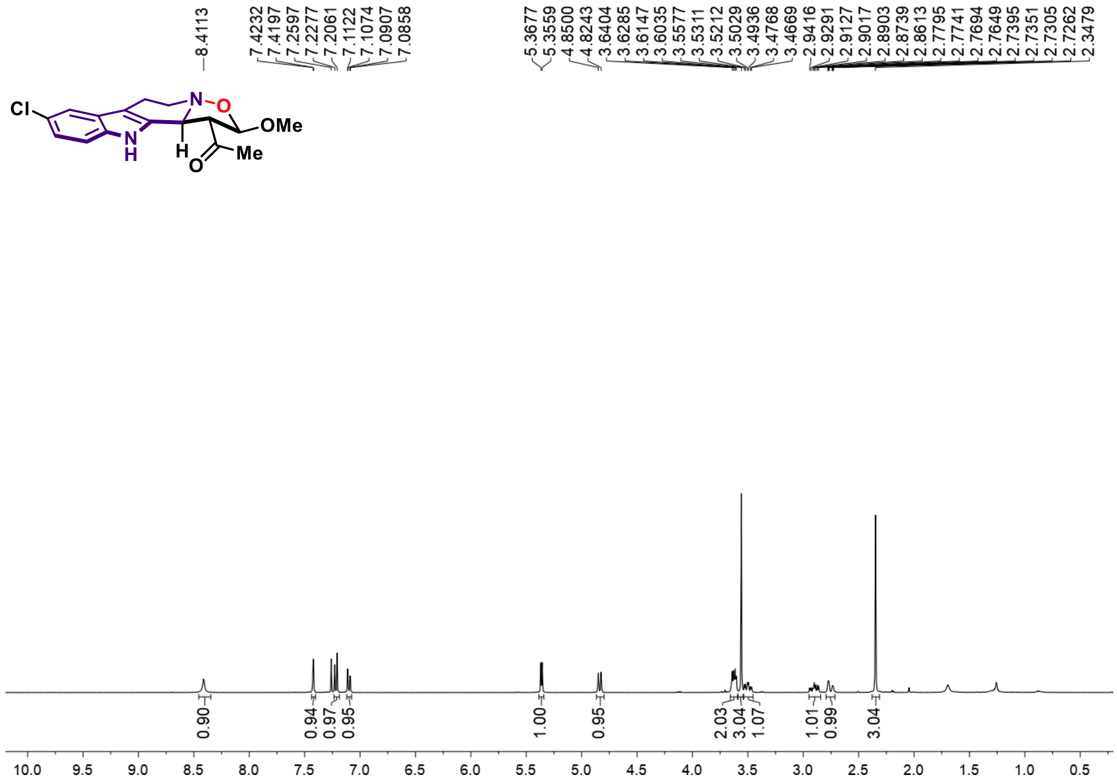

Supplementary Figure 137: ^{13}C NMR of 5g (101 MHz, $CDCl_3$)

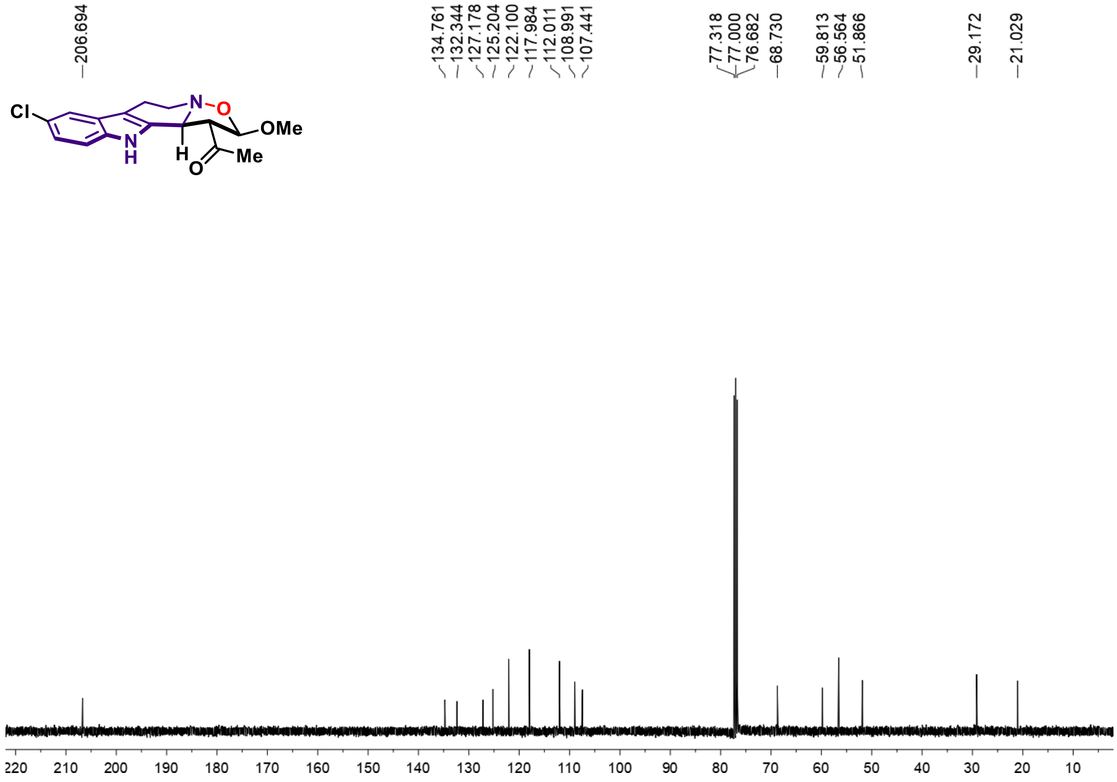

Supplementary Figure 138: HPLC spectrum of

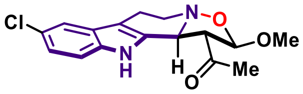

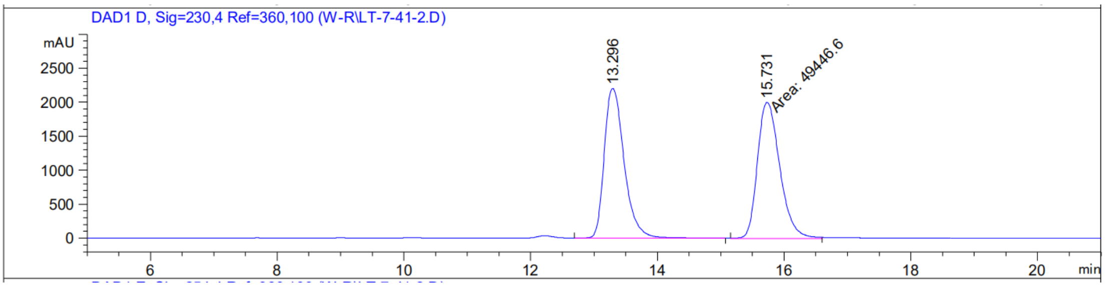

Signal 3: DAD1 D, Sig=230,4 Ref=360,100

Peak #	RetTime [min]	Type	Width [min]	Area [mAU*s]	Height [mAU]	Area %
1	13.296	BB	0.3359	4.78818e4	2200.58203	49.1961
2	15.731	MM	0.4107	4.94466e4	2006.36304	50.8039

Totals : 9.73285e4 4206.94507

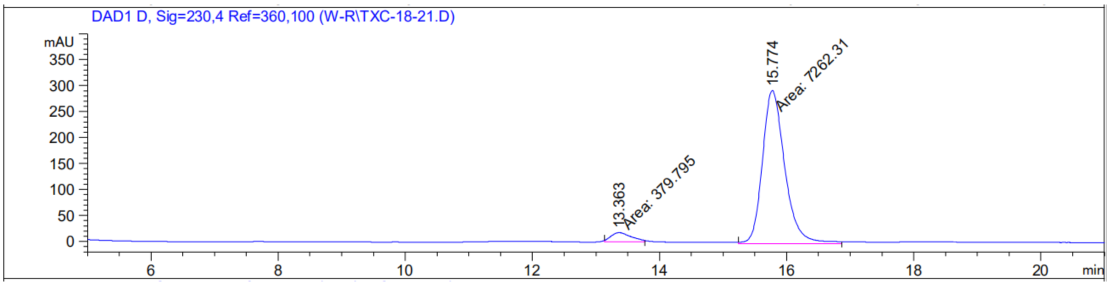

Signal 3: DAD1 D, Sig=230,4 Ref=360,100

Peak #	RetTime [min]	Type	Width [min]	Area [mAU*s]	Height [mAU]	Area %
1	13.363	MM	0.3650	379.79468	17.34216	4.9698
2	15.774	MM	0.4104	7262.30566	294.91293	95.0302

Totals : 7642.10034 312.25510

Supplementary Figure 139: ^{1}H NMR of 5h (400 MHz, $CDCl_3$)

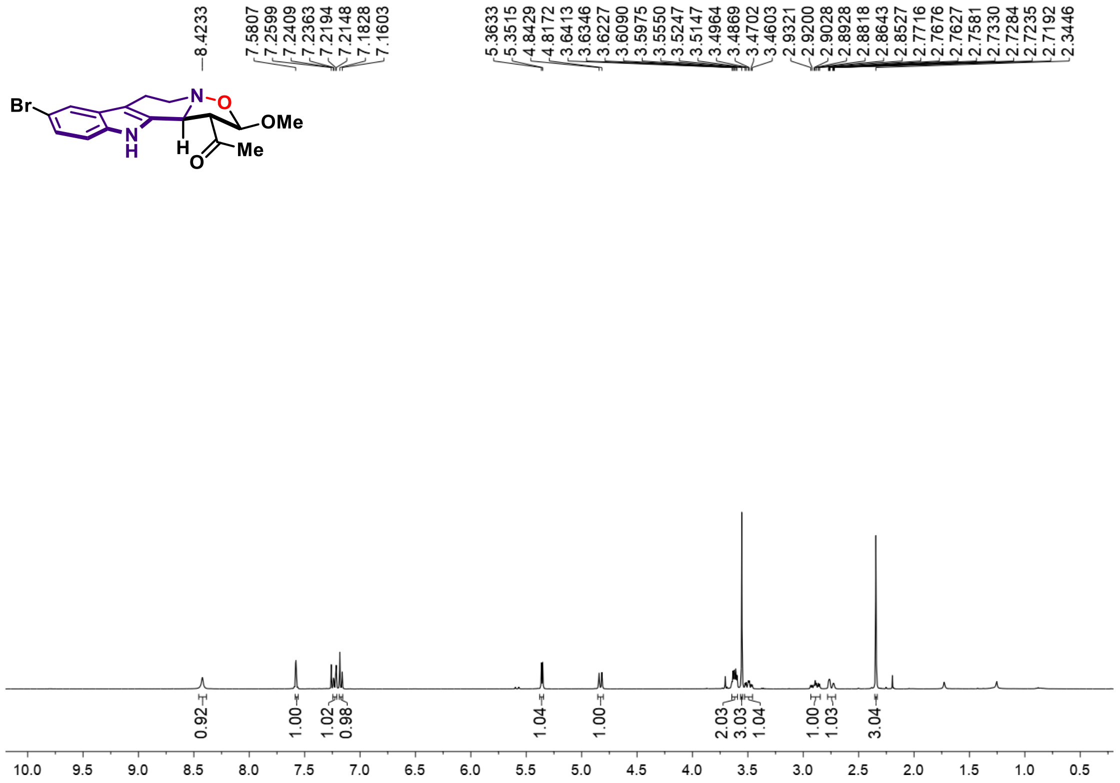

Supplementary Figure 140: ^{13}C NMR of 5h (101 MHz, $CDCl_3$)

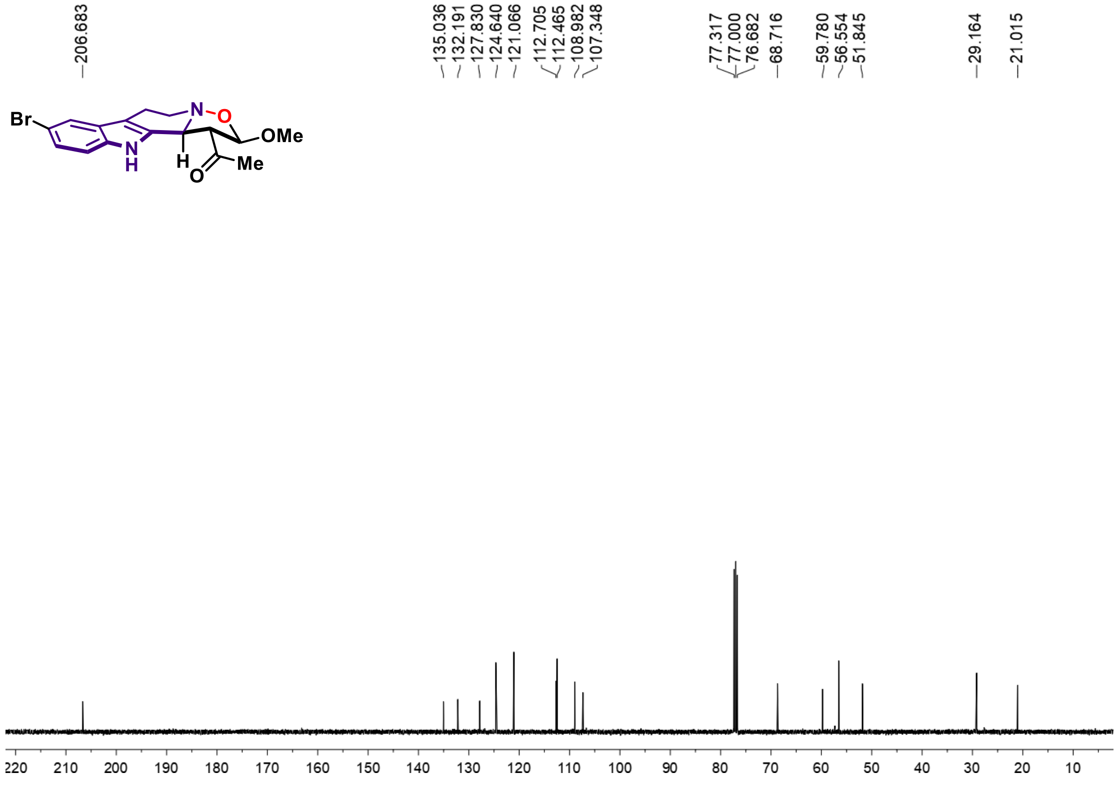

Supplementary Figure 141: HPLC spectrum of 5h

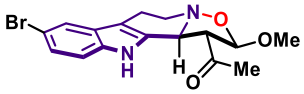

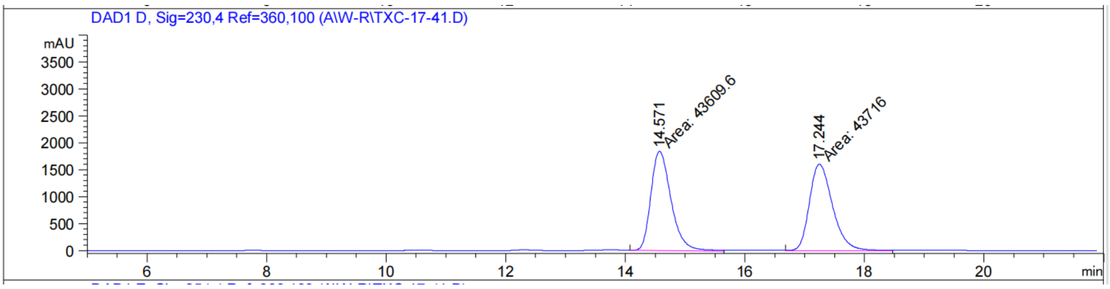

Signal 3: DAD1 D, Sig=230,4 Ref=360,100

Peak #	RetTime [min]	Type	Width [min]	Area [mAU*s]	Height [mAU]	Area %
1	14.571	MM	0.3944	4.36096e4	1842.75513	49.9391
2	17.244	MM	0.4531	4.37160e4	1608.03137	50.0609
Totals :				8.73256e4	3450.78650	

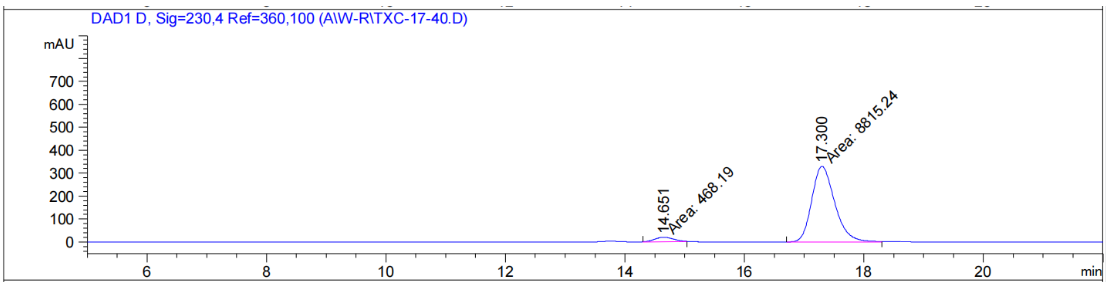

Signal 3: DAD1 D, Sig=230,4 Ref=360,100

Peak #	RetTime [min]	Type	Width [min]	Area [mAU*s]	Height [mAU]	Area %
1	14.651	MM	0.3784	468.18997	20.62062	5.0433
2	17.300	MM	0.4459	8815.24414	329.49054	94.9567
Totals :				9283.43411	350.11116	

Supplementary Figure 142: ^{1}H NMR of 5i (400 MHz, $CDCl_3$)

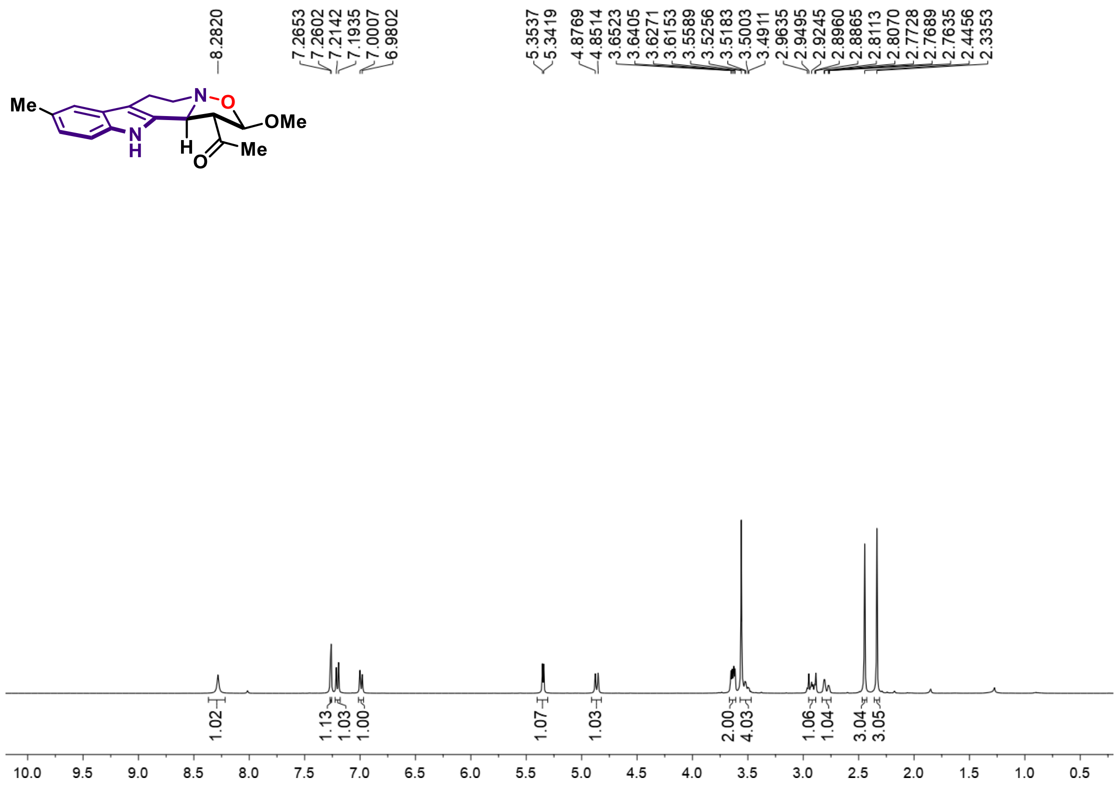

Supplementary Figure 143: ^{13}C NMR of 5i (101 MHz, $CDCl_3$)

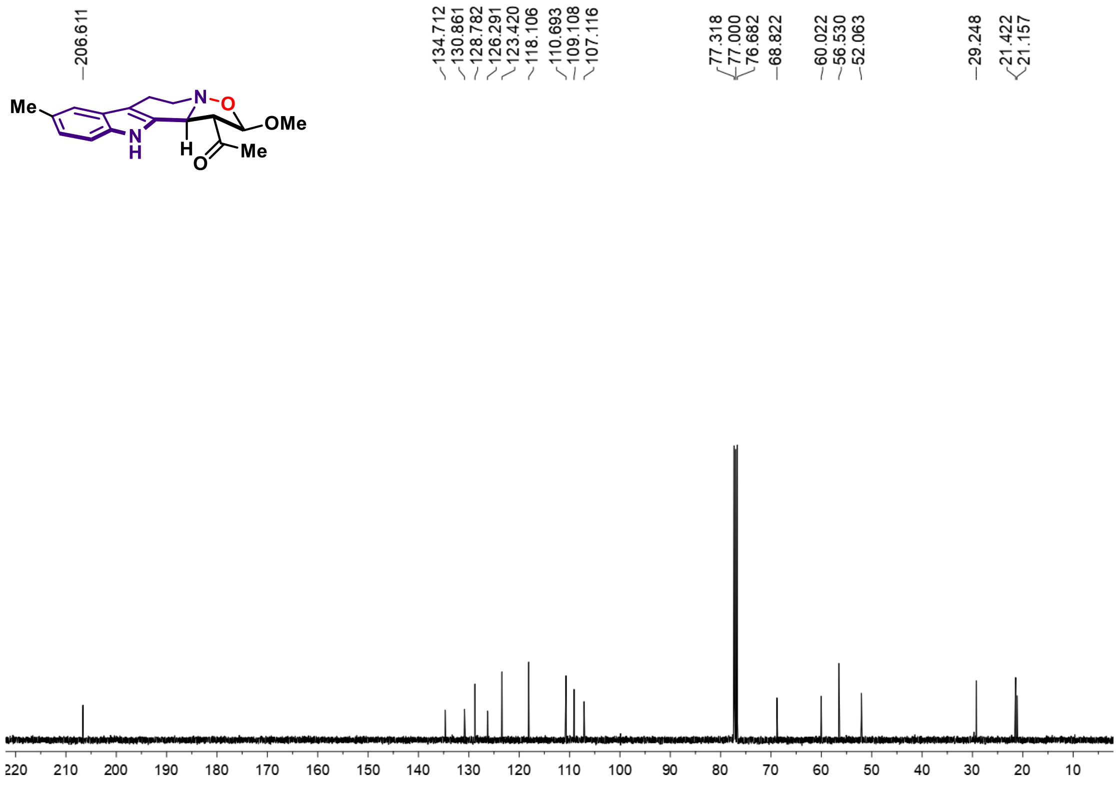

Supplementary Figure 144: HPLC spectrum of

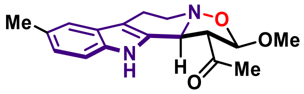

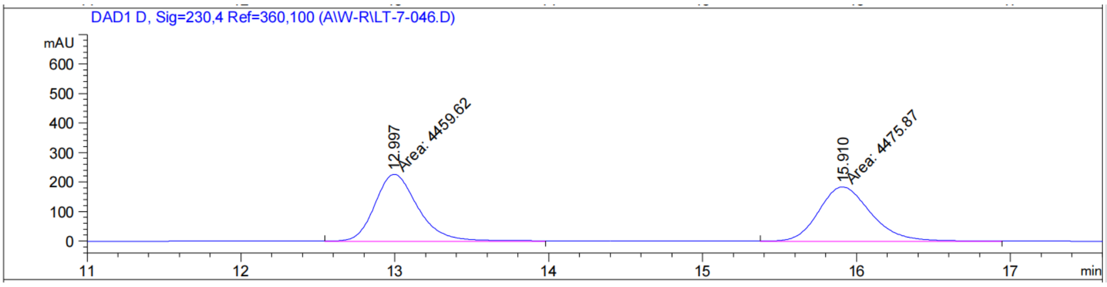

Signal 3: DAD1 D, Sig=230,4 Ref=360,100

Peak #	RetTime [min]	Type	Width [min]	Area [mAU*s]	Height [mAU]	Area %
1	12.997	MM	0.3273	4459.62061	227.08838	49.9091
2	15.910	MM	0.4022	4475.86914	185.46182	50.0909

Totals : 8935.48975 412.55020

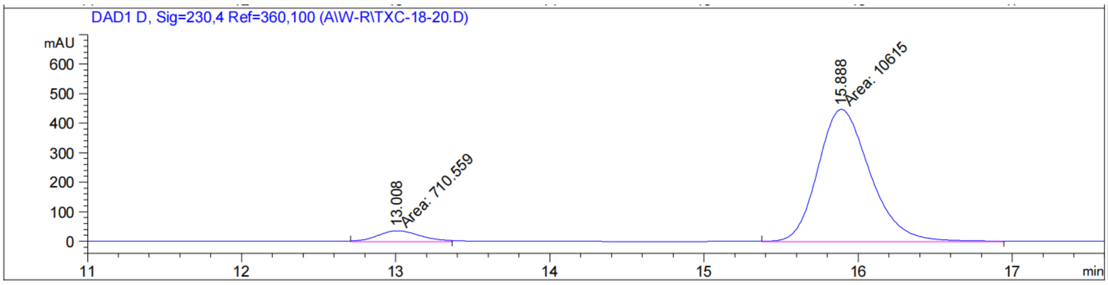

Signal 3: DAD1 D, Sig=230,4 Ref=360,100

Peak #	RetTime [min]	Type	Width [min]	Area [mAU*s]	Height [mAU]	Area %
1	13.008	MM	0.3229	710.55902	36.67924	6.2739
2	15.888	MM	0.3944	1.06150e4	448.55014	93.7261

Totals : 1.13256e4 485.22939

Supplementary Figure 145: ^{1}H NMR of 5j (400 MHz, $CDCl_3$)

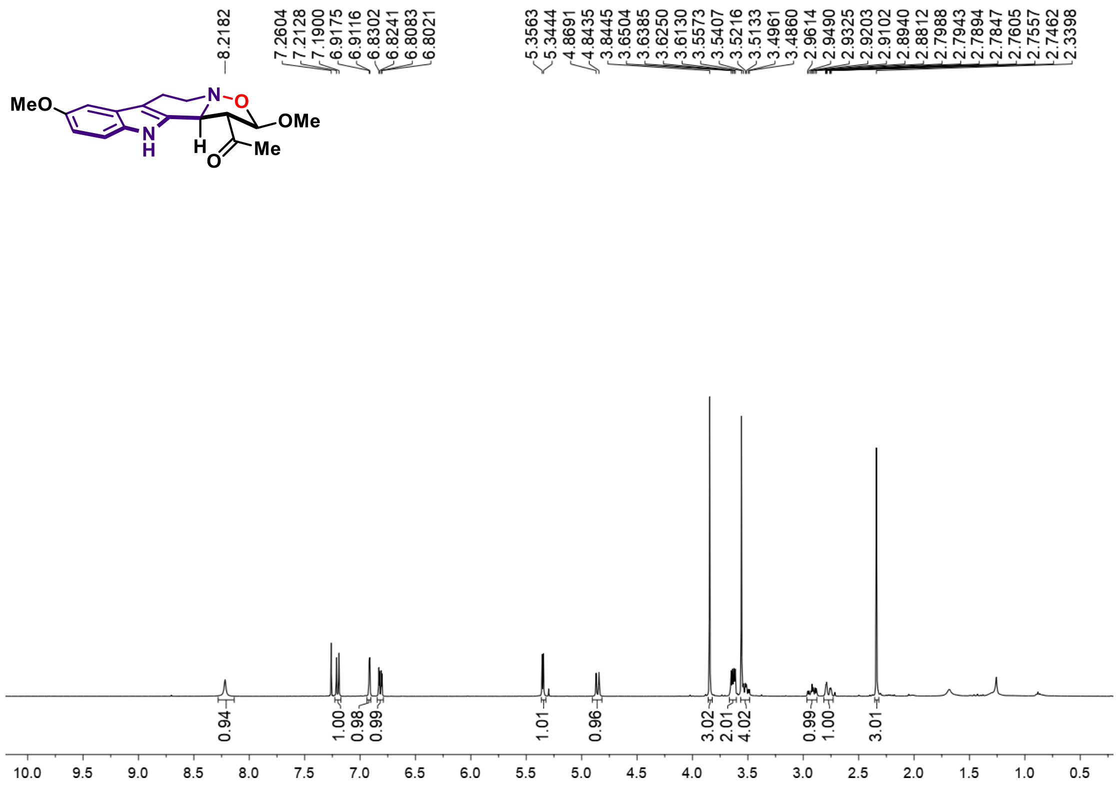

Supplementary Figure 146: ^{13}C NMR of 5j (101 MHz, $CDCl_3$)

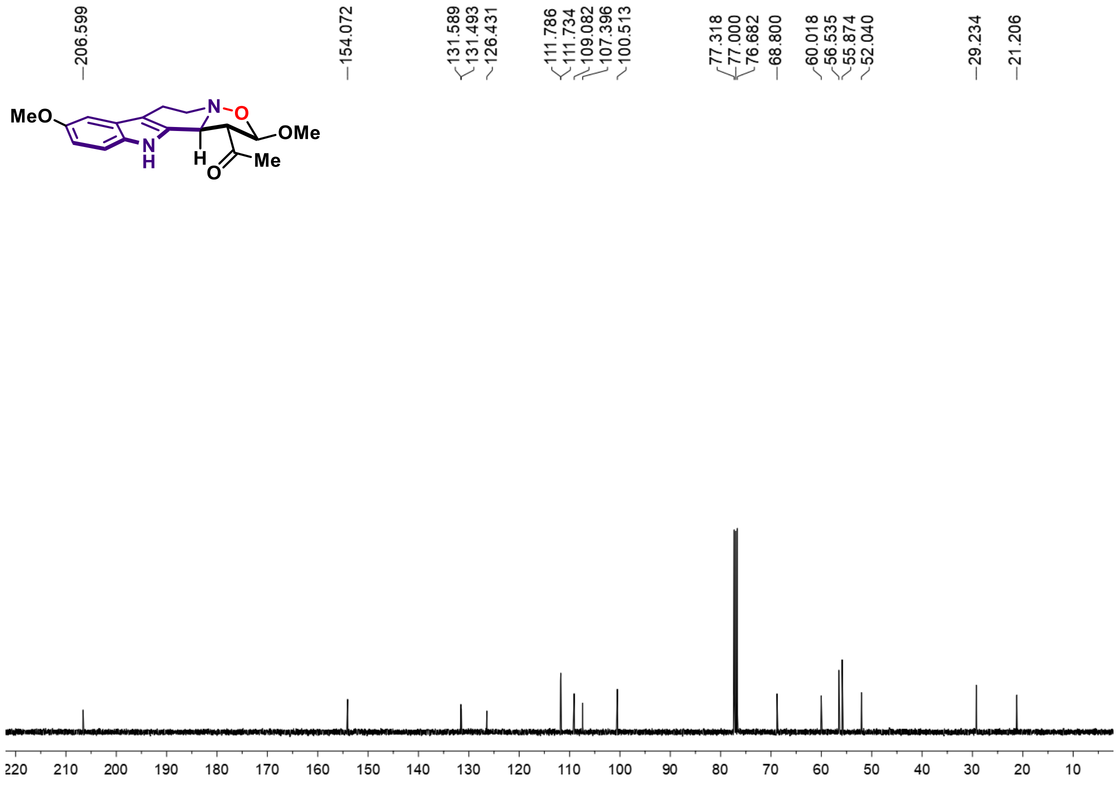

Supplementary Figure 147: HPLC spectrum of 5j

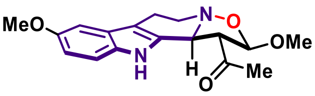

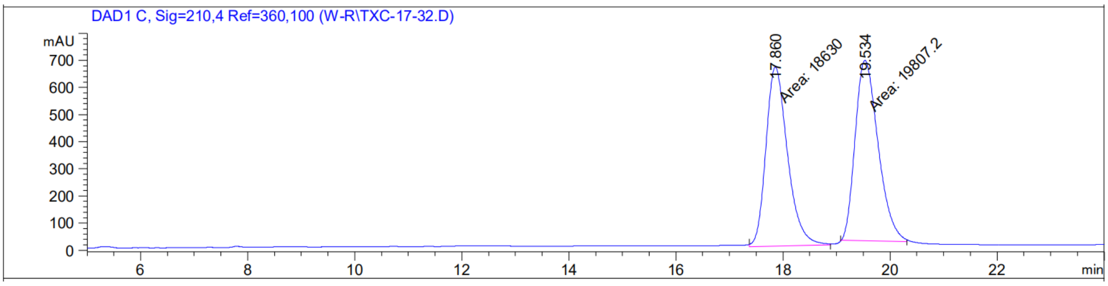

Signal 2: DAD1 C, Sig=210,4 Ref=360,100

Peak #	RetTime [min]	Type	Width [min]	Area [mAU*s]	Height [mAU]	Area %
1	17.860	MM	0.4698	1.86300e4	660.97681	48.4687
2	19.534	MM	0.4964	1.98072e4	664.99475	51.5313

Totals : 3.84372e4 1325.97156

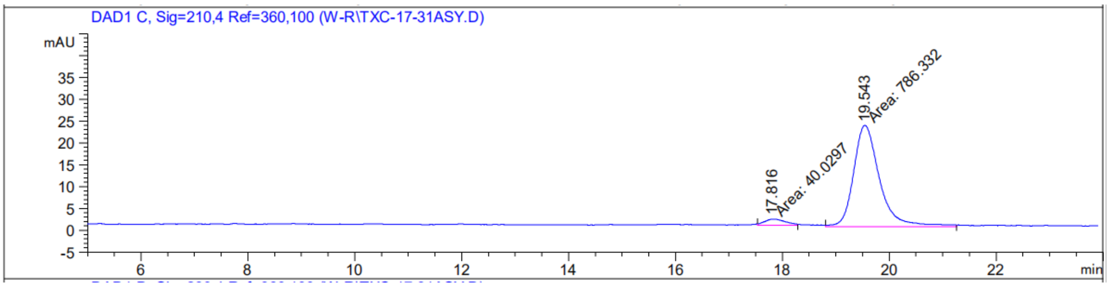

Signal 2: DAD1 C, Sig=210,4 Ref=360,100

Peak #	RetTime [min]	Type	Width [min]	Area [mAU*s]	Height [mAU]	Area %
1	17.816	MM	0.4661	40.02971	1.43134	4.8441
2	19.543	MM	0.5649	786.33154	23.19845	95.1559

Totals : 826.36125 24.62979

Supplementary Figure 148: ^{1}H NMR of 5k (400 MHz, CDCl$_3$)

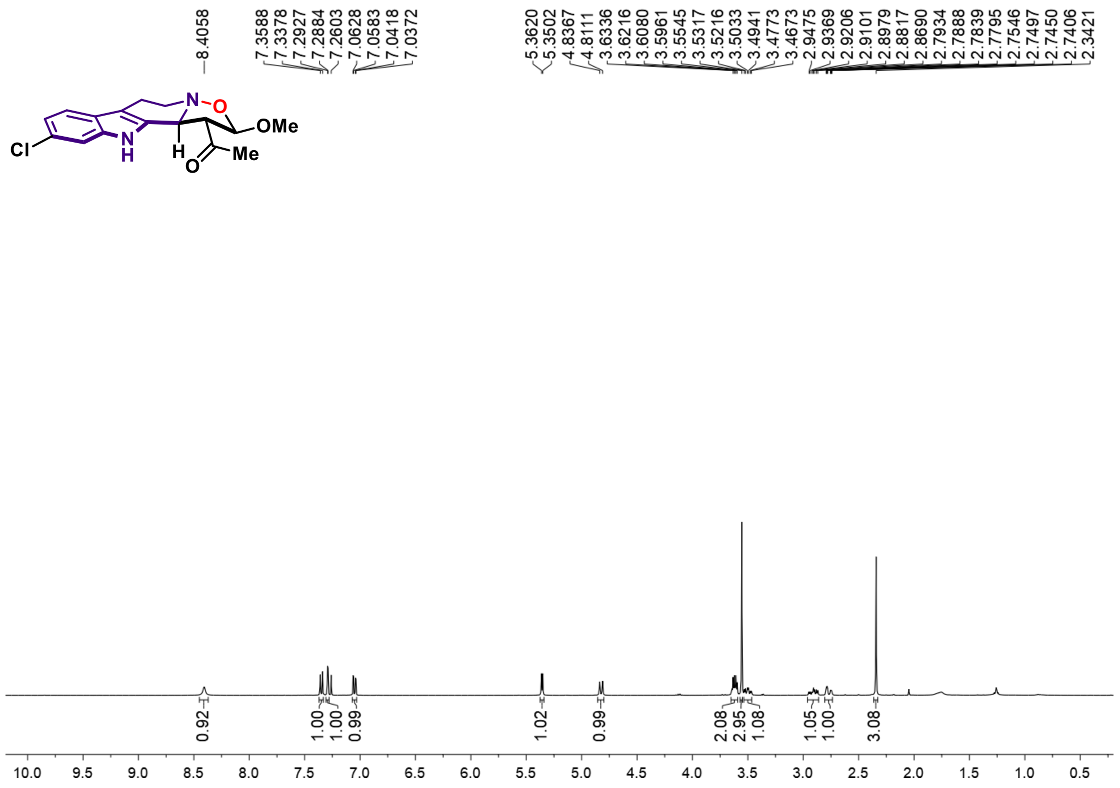

Supplementary Figure 149: ^{13}C NMR of 5k (101 MHz, CDCl$_3$)

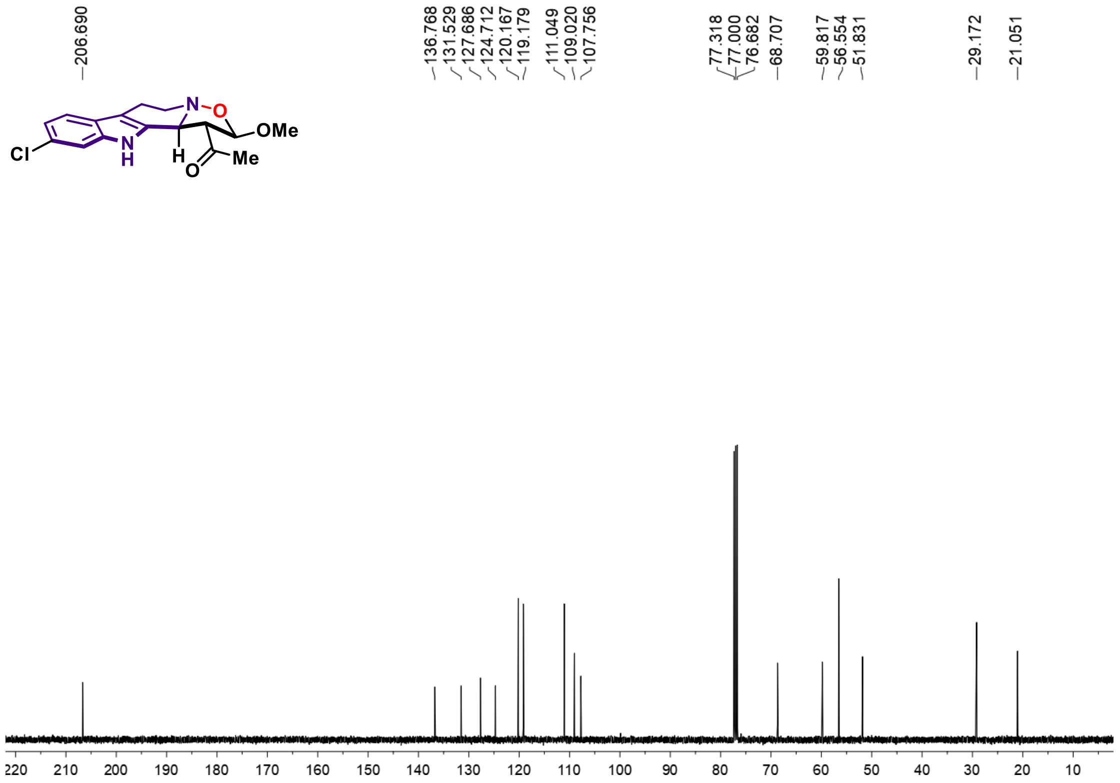

Supplementary Figure 150: HPLC spectrum of 5k

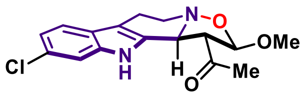

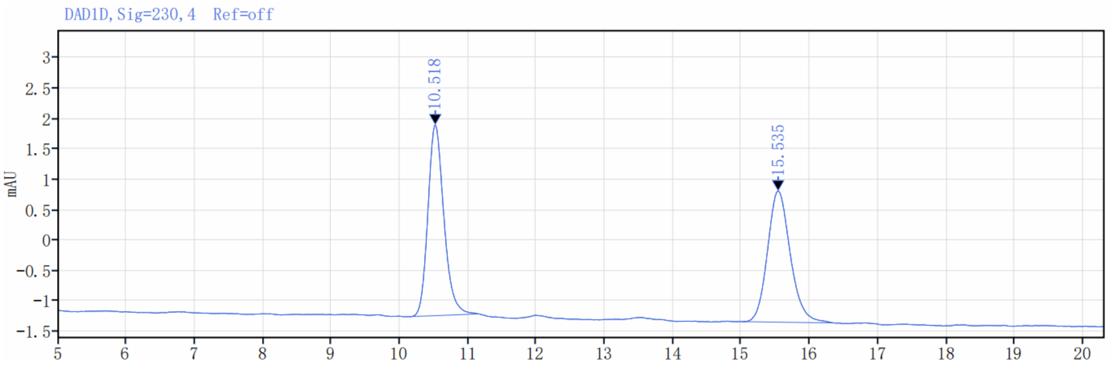

Signal : DAD1D, Sig=230, 4 Ref=off

RetTime [min]	Type	Width [min]	Area [mAU*s]	Height [mAU]	Area%
10. 518	MM m	0. 25	50. 79	3. 13	50. 47
15. 535	MM m	0. 34	49. 84	2. 15	49. 53
	Totals	0. 59	100. 62		

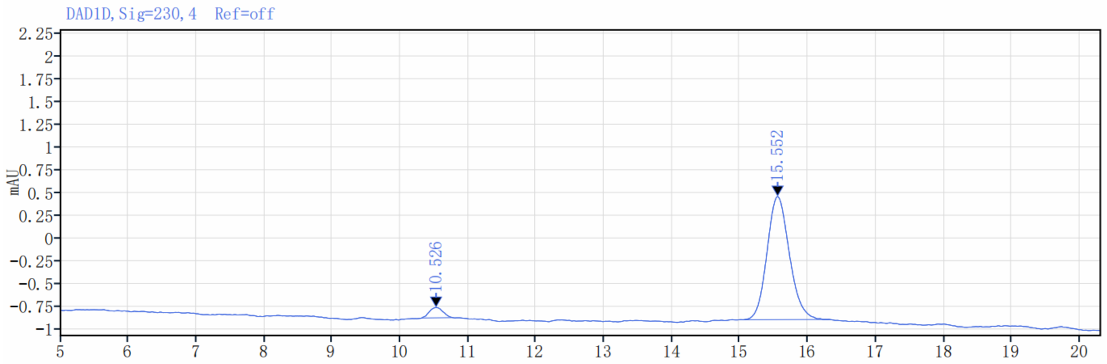

Signal : DAD1D, Sig=230, 4 Ref=off

RetTime [min]	Type	Width [min]	Area [mAU*s]	Height [mAU]	Area%
10. 526	MM m	0. 17	1. 57	0. 11	4. 85
15. 552	MM m	0. 34	30. 93	1. 35	95. 15
	Totals	0. 51	32. 50		

Supplementary Figure 151: ^{1}H NMR of 5l (400 MHz, CDCl₃)

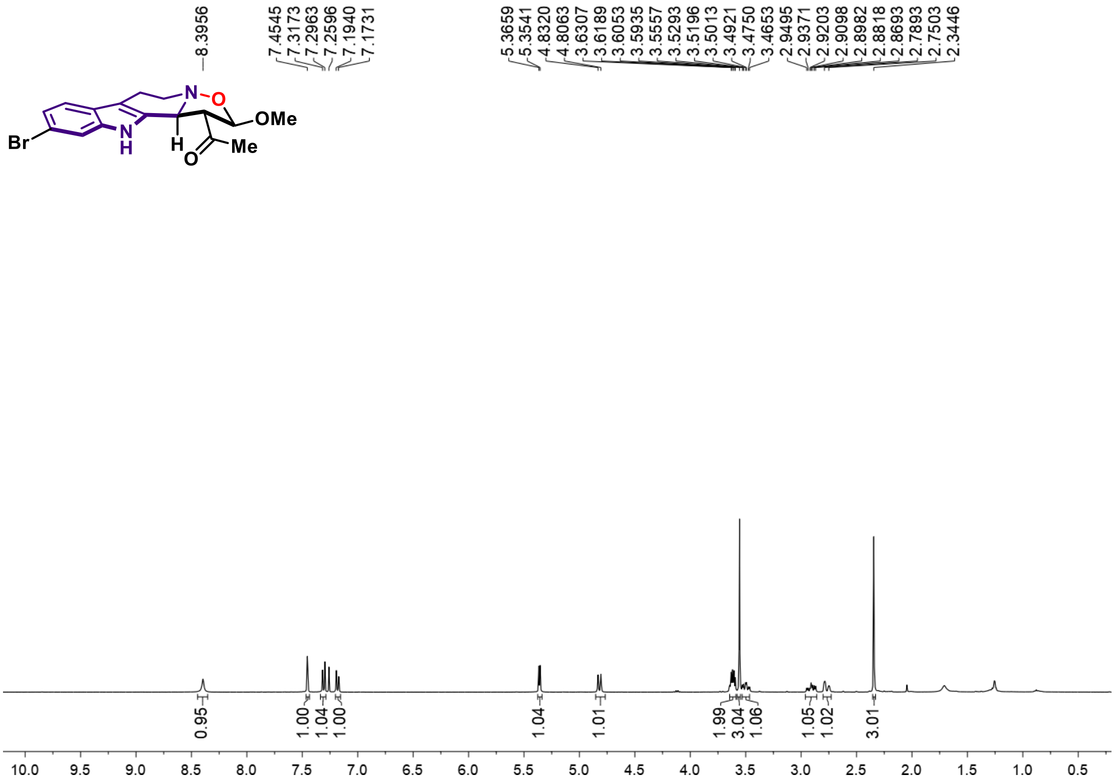

Supplementary Figure 152: ^{13}C NMR of 5l (101 MHz, CDCl₃)

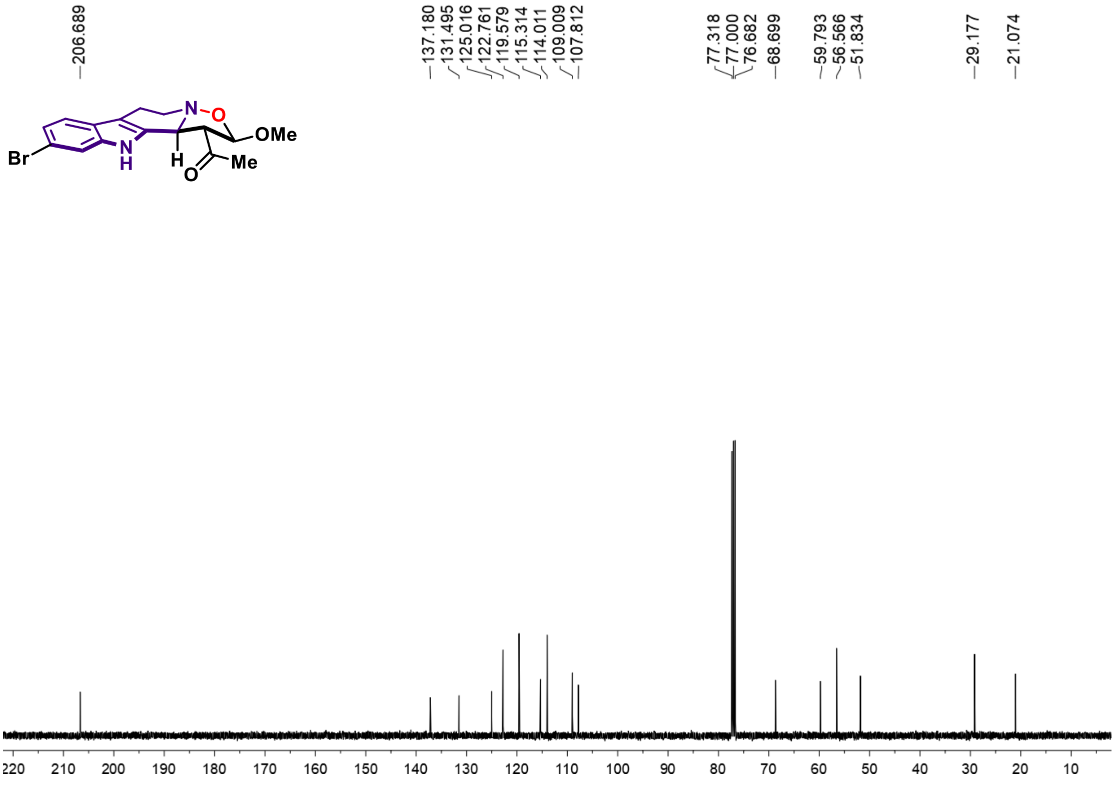

Supplementary Figure 153: HPLC spectrum of 5l

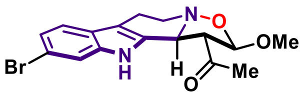

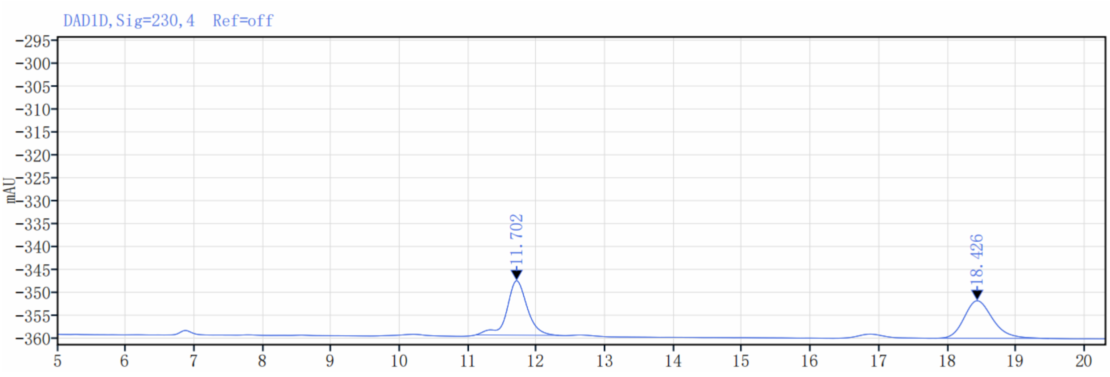

Signal : DAD1D, Sig=230, 4 Ref=off

RetTime [min]	Type	Width [min]	Area [mAU*s]	Height [mAU]	Area%
11. 702	MM m	0. 31	244. 98	11. 86	50. 77
18. 426	MM m	0. 44	237. 54	8. 21	49. 23
	Totals	0. 75	482. 52		

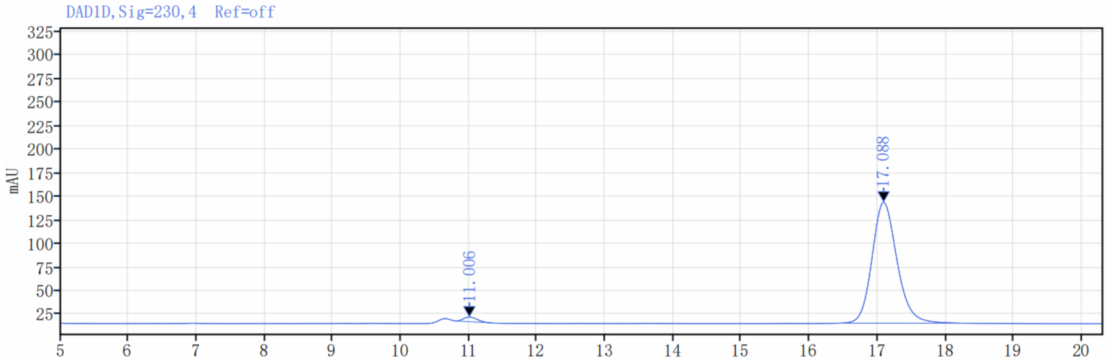

Signal : DAD1D, Sig=230, 4 Ref=off

RetTime [min]	Type	Width [min]	Area [mAU*s]	Height [mAU]	Area%
11. 006	MM m	0. 22	66. 80	4. 83	2. 01
17. 088	MM m	0. 39	3264. 21	128. 22	97. 99
	Totals	0. 61	3331. 01		

Supplementary Figure 154: ^{1}H NMR of 5m (400 MHz, $CDCl_3$)

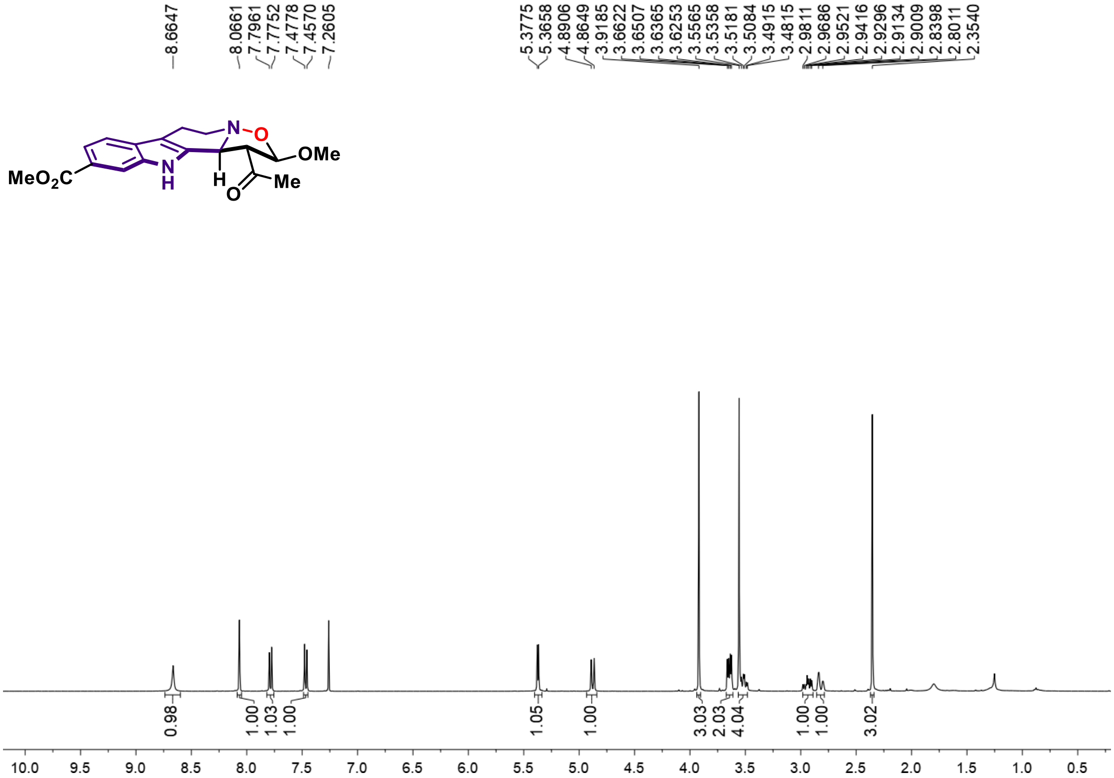

Supplementary Figure 155: ^{13}C NMR of 5m (101 MHz, $CDCl_3$)

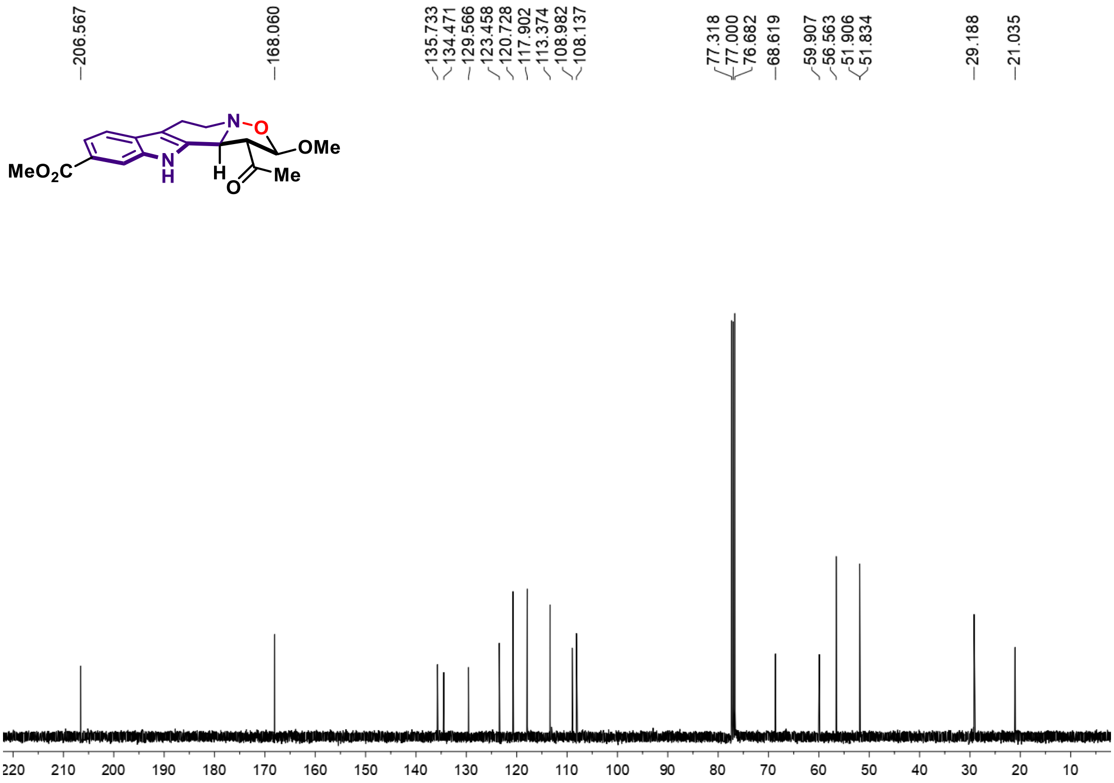

Supplementary Figure 156: HPLC spectrum of 5m

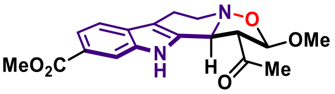

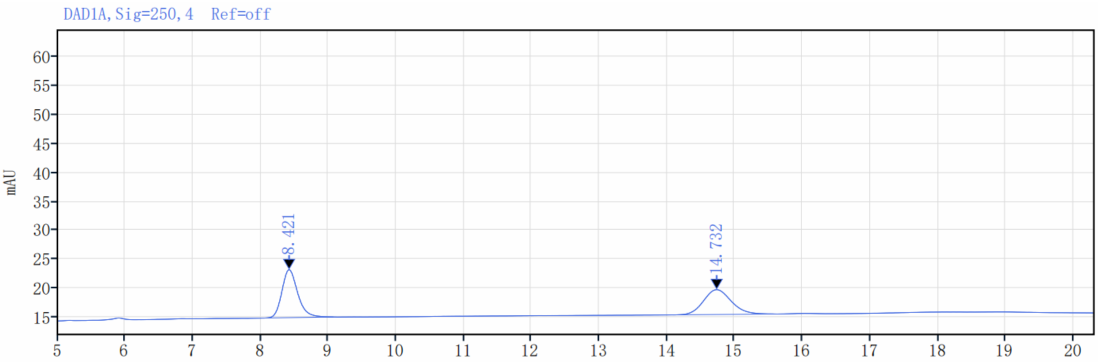

Signal : DAD1A, Sig=250, 4 Ref=off

RetTime [min]	Type	Width [min]	Area [mAU*s]	Height [mAU]	Area%
8.421	MM m	0.25	134.65	8.29	52.49
14.732	MM m	0.43	121.87	4.29	47.51
	Totals	0.68	256.52		

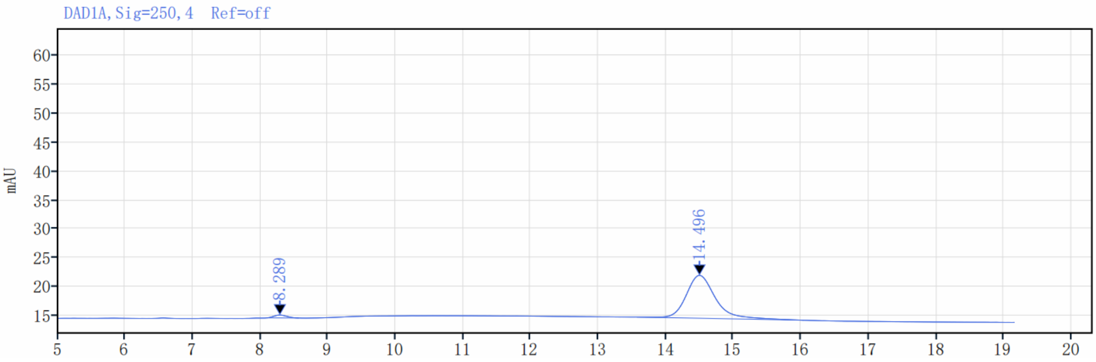

Signal : DAD1A, Sig=250, 4 Ref=off

RetTime [min]	Type	Width [min]	Area [mAU*s]	Height [mAU]	Area%
8.289	MM m	0.20	6.51	0.50	2.83
14.496	MM m	0.45	223.24	7.42	97.17
	Totals	0.65	229.75		

Supplementary Figure 157: ^{1}H NMR of 5n (400 MHz, CDCl$_3$)

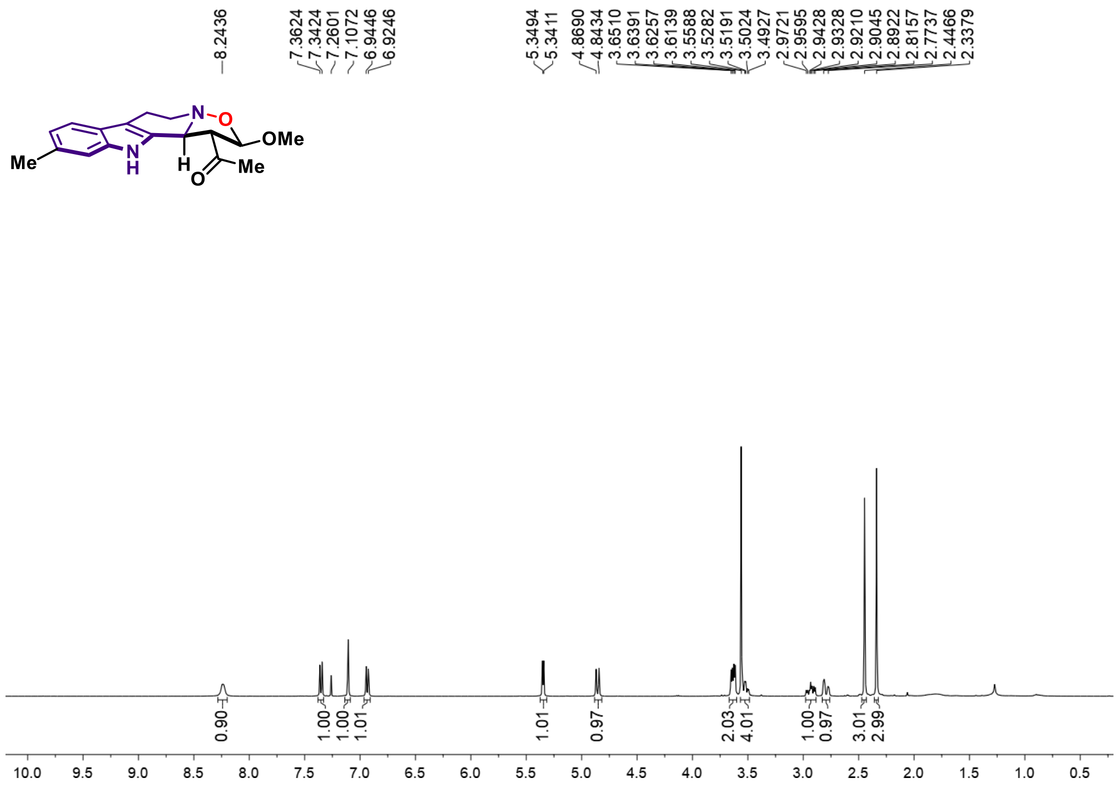

Supplementary Figure 158: ^{13}C NMR of 5n (101 MHz, CDCl$_3$)

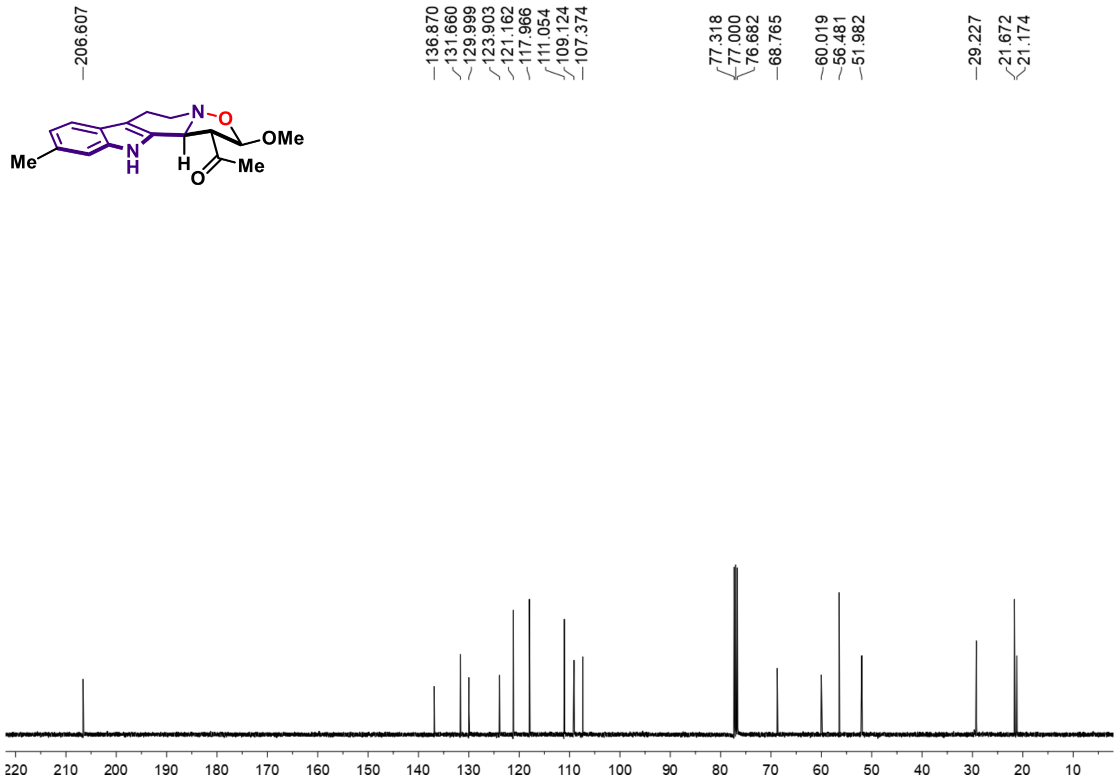

Supplementary Figure 159: HPLC spectrum of 5n

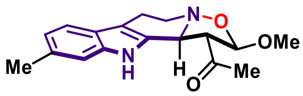

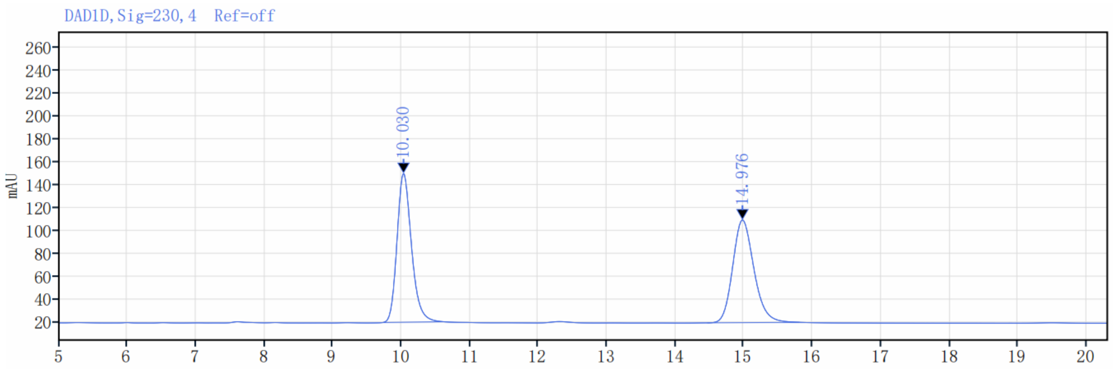

Signal : DAD1D, Sig=230, 4 Ref=off

RetTime [min]	Type	Width [min]	Area [mAU*s]	Height [mAU]	Area%
10.030	MM m	0.23	1907.26	129.87	49.71
14.976	MM m	0.33	1929.79	89.65	50.29
	Totals	0.56	3837.06		

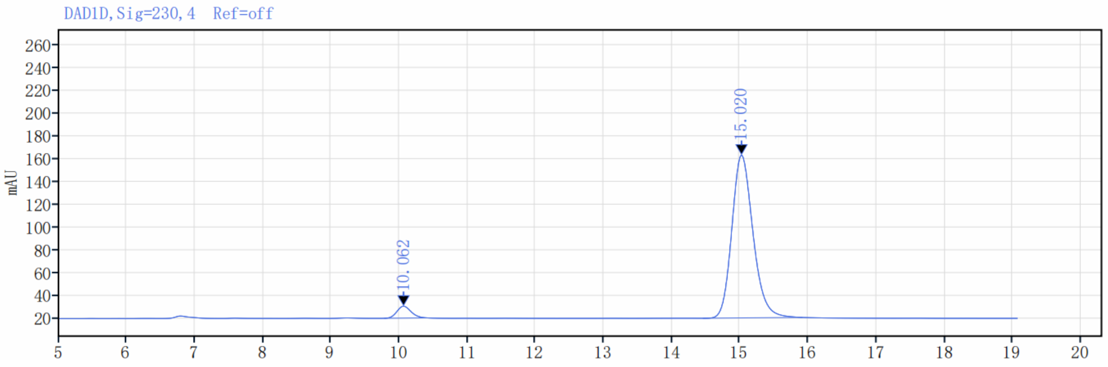

Signal : DAD1D, Sig=230, 4 Ref=off

RetTime [min]	Type	Width [min]	Area [mAU*s]	Height [mAU]	Area%
10.062	MM m	0.22	150.60	10.45	4.66
15.020	MM m	0.33	3082.58	142.91	95.34
	Totals	0.55	3233.17		

Supplementary Figure 160: ^{1}H NMR of 5o (400 MHz, $CDCl_3$)

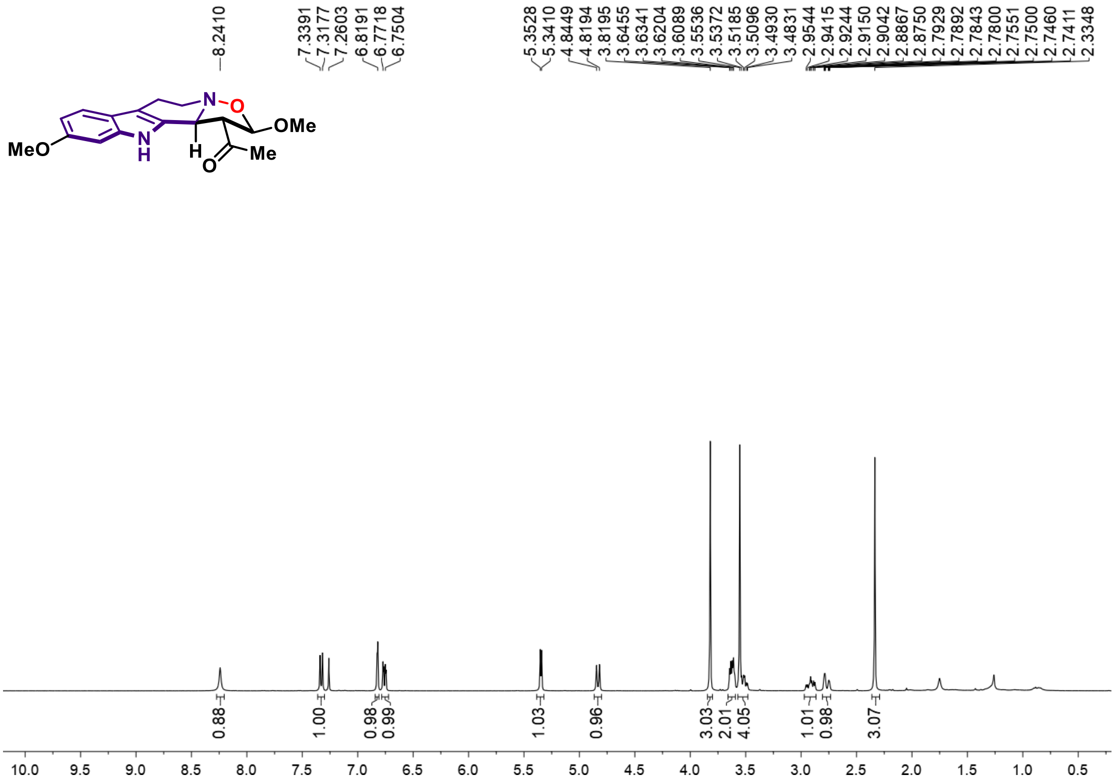

Supplementary Figure 161: ^{13}C NMR of 5o (101 MHz, $CDCl_3$)

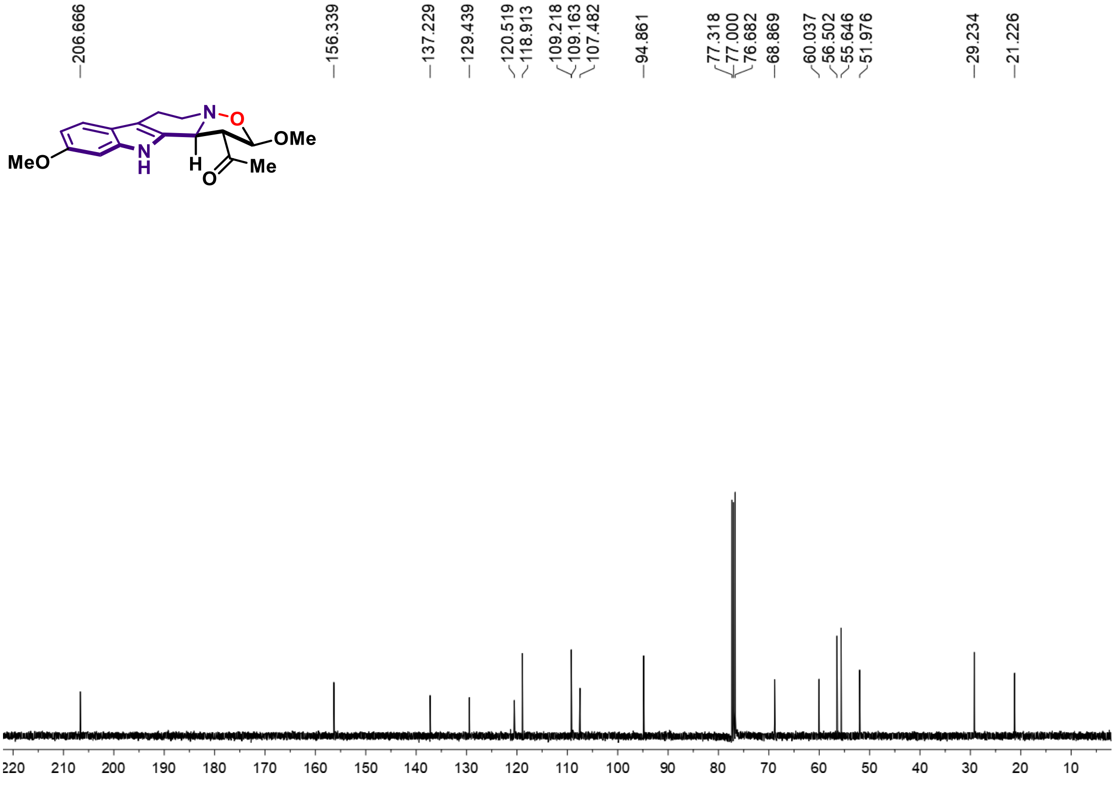

Supplementary Figure 162: HPLC spectrum of 5o

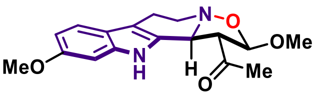

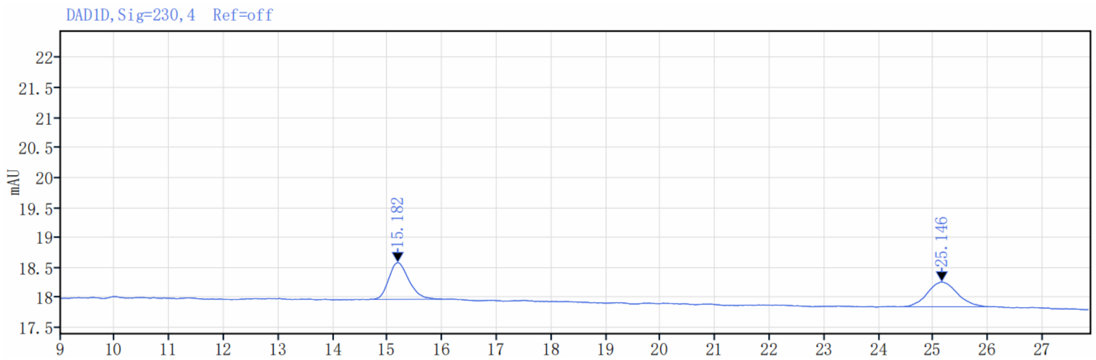

Signal : DAD1D, Sig=230, 4 Ref=off

RetTime [min]	Type	Width [min]	Area [mAU*s]	Height [mAU]	Area%
15.182	MM m	0.31	15.63	0.61	50.07
25.146	MM m	0.45	15.59	0.41	49.93
	Totals	0.77	31.22		

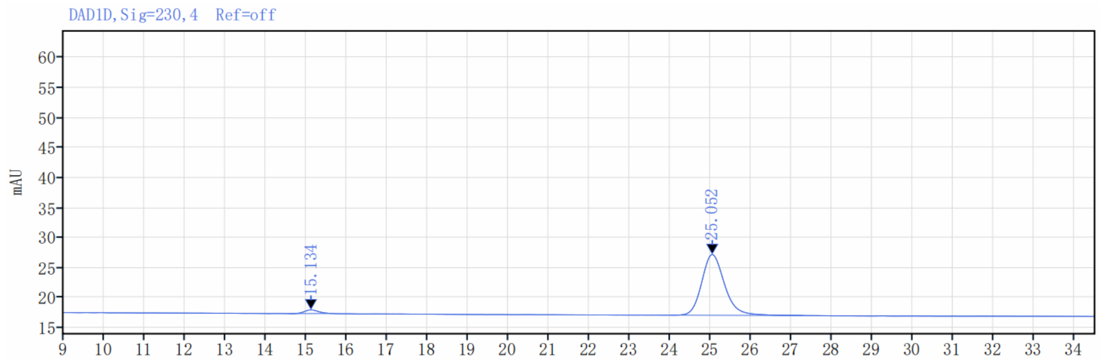

Signal : DAD1D, Sig=230, 4 Ref=off

RetTime [min]	Type	Width [min]	Area [mAU*s]	Height [mAU]	Area%
15.134	MM m	0.34	14.72	0.61	3.58
25.052	MM m	0.60	395.84	10.10	96.42
	Totals	0.93	410.56		

Supplementary Figure 163: ^{1}H NMR of 5p (400 MHz, $CDCl_3$)

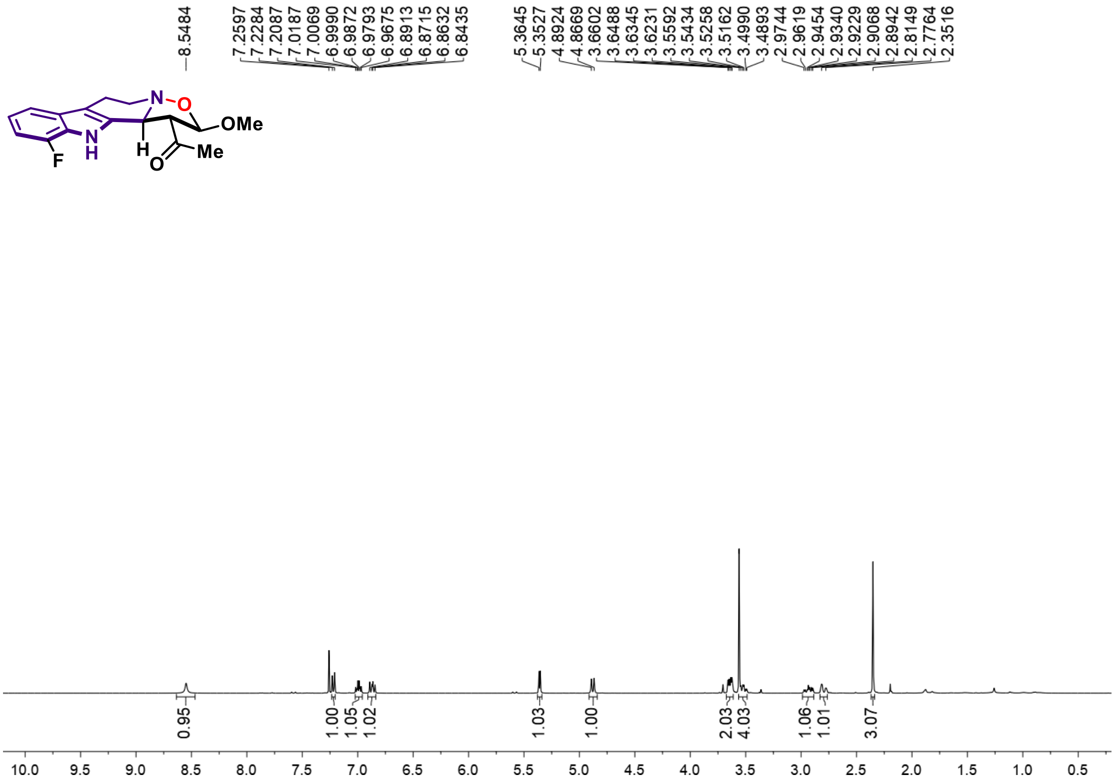

Supplementary Figure 164: ^{13}C NMR of 5p (101 MHz, $CDCl_3$)

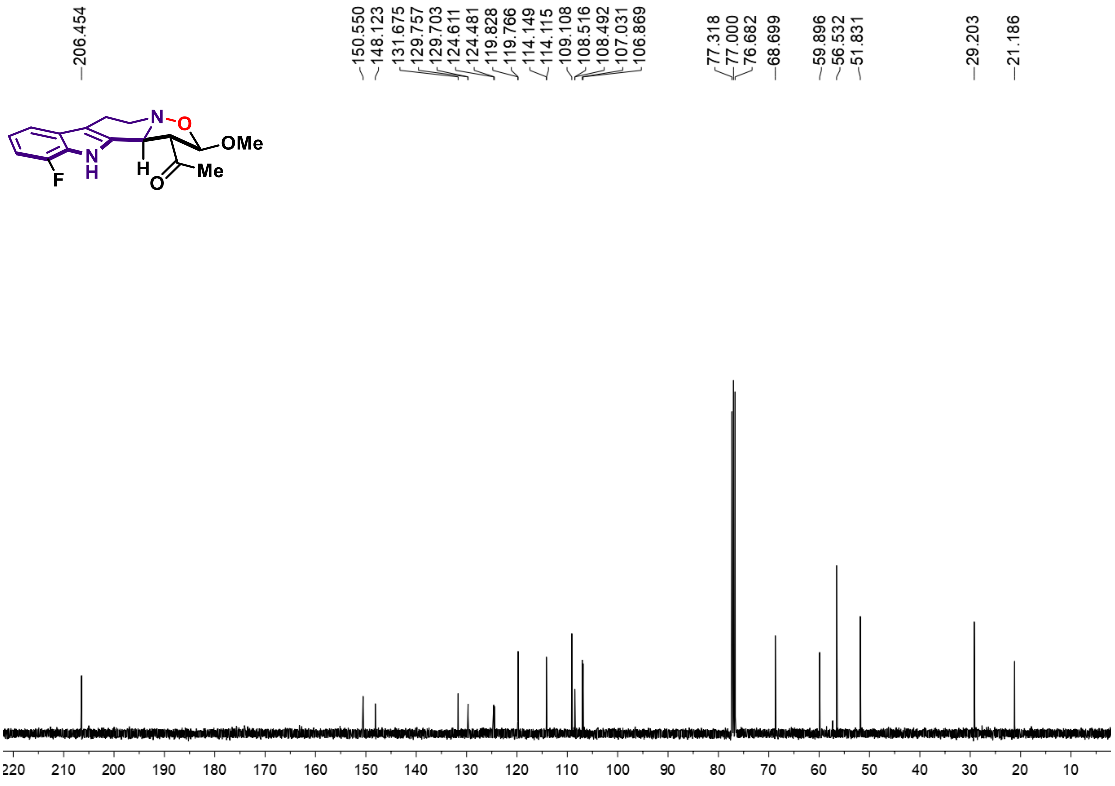

Supplementary Figure 165: HPLC spectrum of 5p

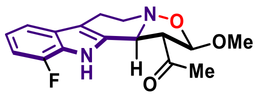

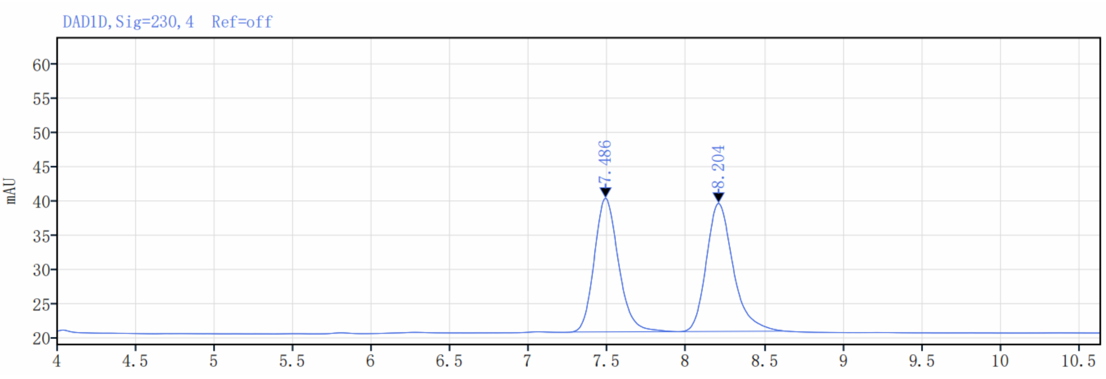

Signal : DAD1D, Sig=230, 4 Ref=off

RetTime [min]	Type	Width [min]	Area [mAU*s]	Height [mAU]	Area%
7.486	MM m	0.16	208.81	19.52	48.49
8.204	MM m	0.18	221.79	18.71	51.51
	Totals	0.35	430.60		

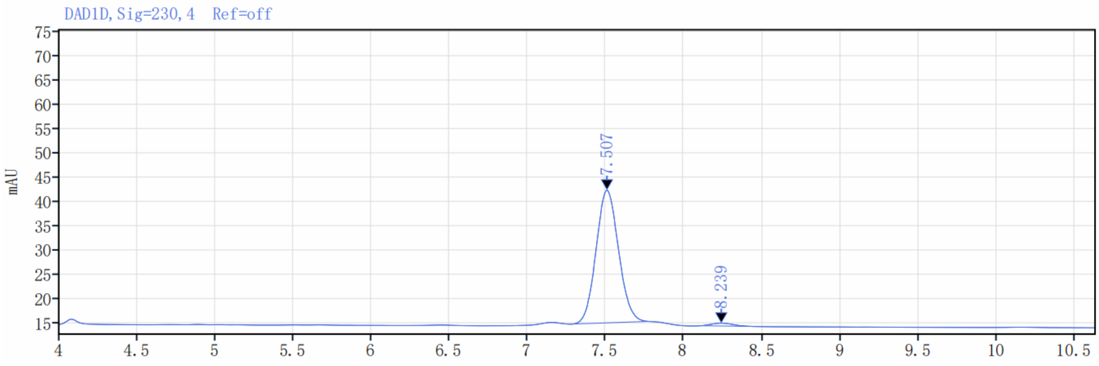

Signal : DAD1D, Sig=230, 4 Ref=off

RetTime [min]	Type	Width [min]	Area [mAU*s]	Height [mAU]	Area%
7.507	MM m	0.15	272.94	27.35	98.05
8.239	MM m	0.15	5.42	0.57	1.95
	Totals	0.30	278.36		

Supplementary Figure 166: ^{1}H NMR of 5q (400 MHz, $CDCl_3$)

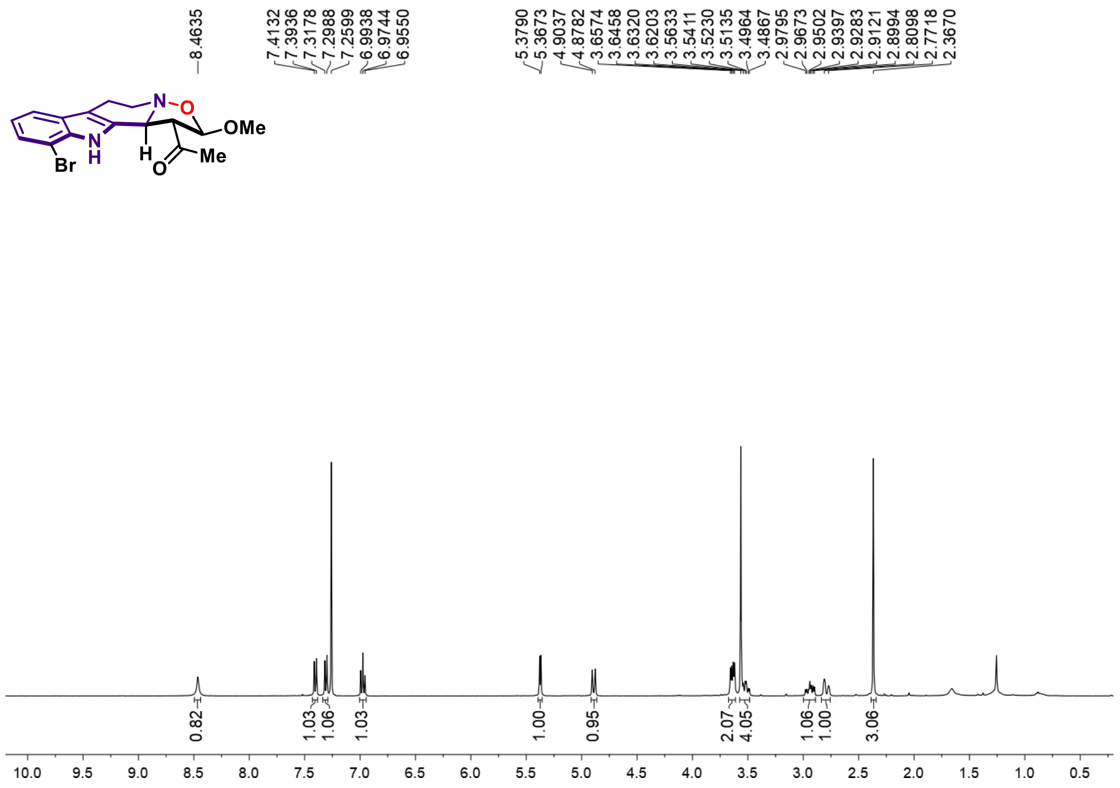

Supplementary Figure 167: ^{13}C NMR of 5q (101 MHz, $CDCl_3$)

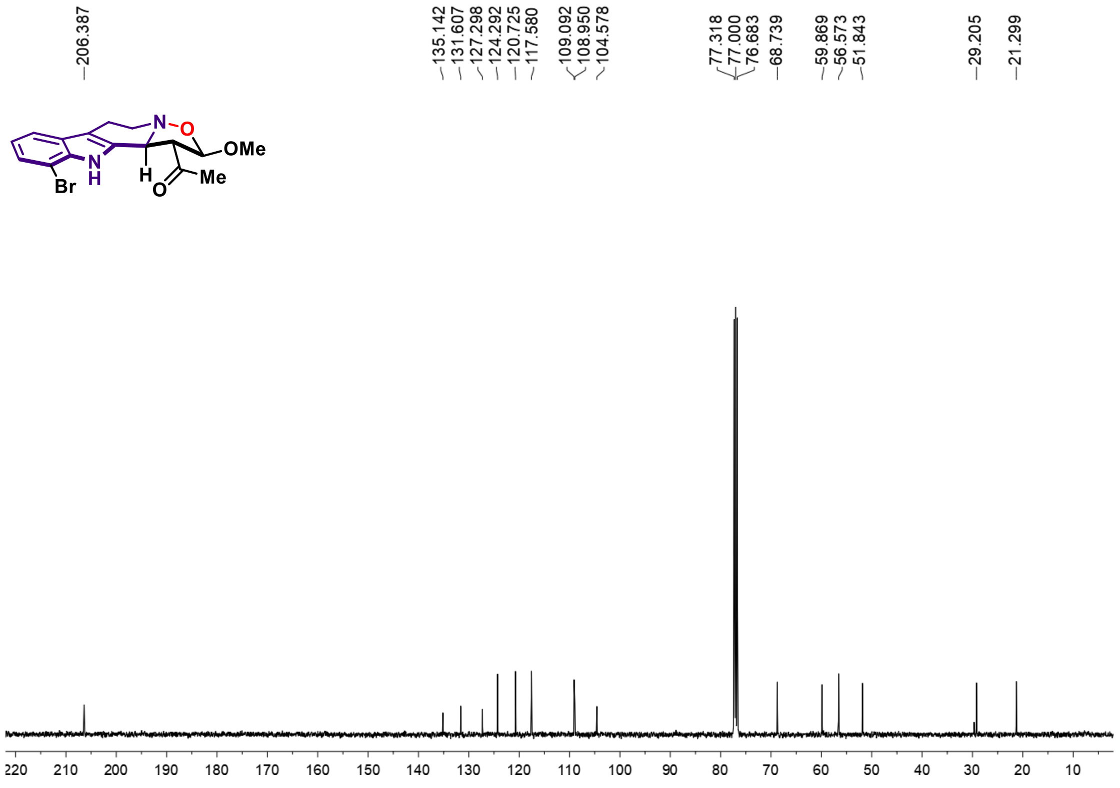

Supplementary Figure 168: HPLC spectrum of 5q

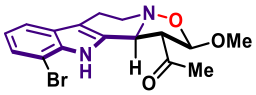

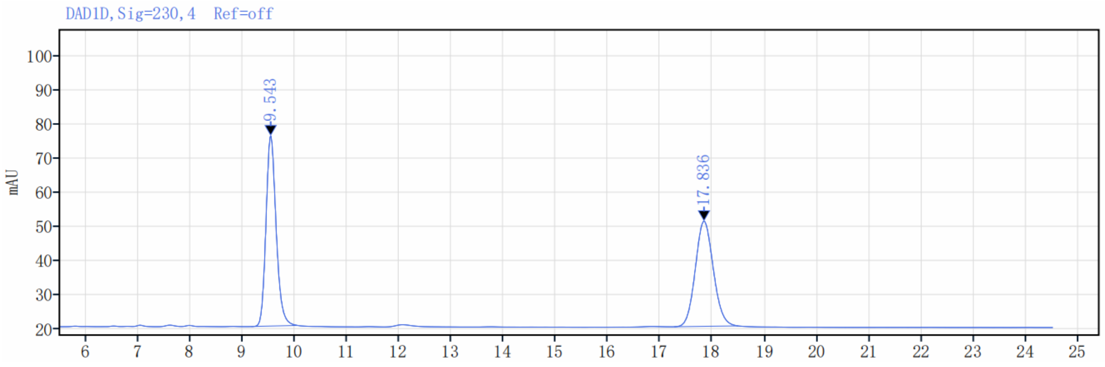

Signal : DAD1D, Sig=230, 4 Ref=off

RetTime [min]	Type	Width [min]	Area [mAU*s]	Height [mAU]	Area%
9.543	MM m	0.21	752.07	55.82	50.42
17.836	MM m	0.37	739.67	30.86	49.58
	Totals	0.58	1491.74		

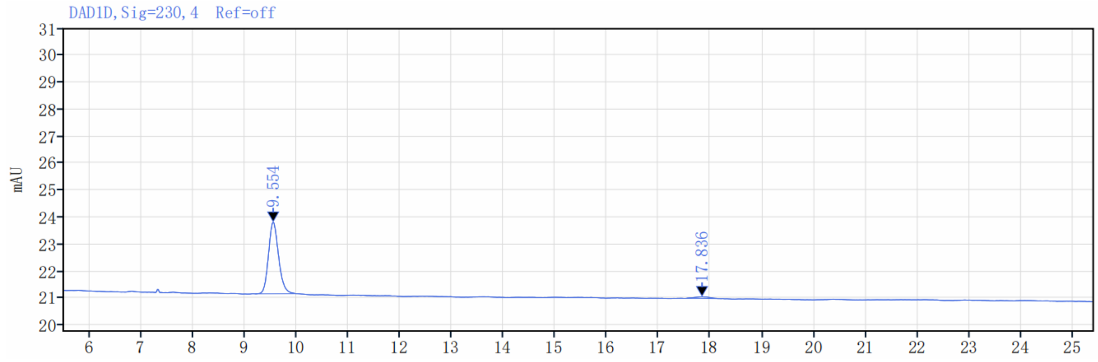

Signal : DAD1D, Sig=230, 4 Ref=off

RetTime [min]	Type	Width [min]	Area [mAU*s]	Height [mAU]	Area%
9.554	MM m	0.21	35.82	2.65	96.81
17.836	MM m	0.24	1.18	0.06	3.19
	Totals	0.45	37.00		

Supplementary Figure 169: ^{1}H NMR of 5r (400 MHz, $CDCl_3$)

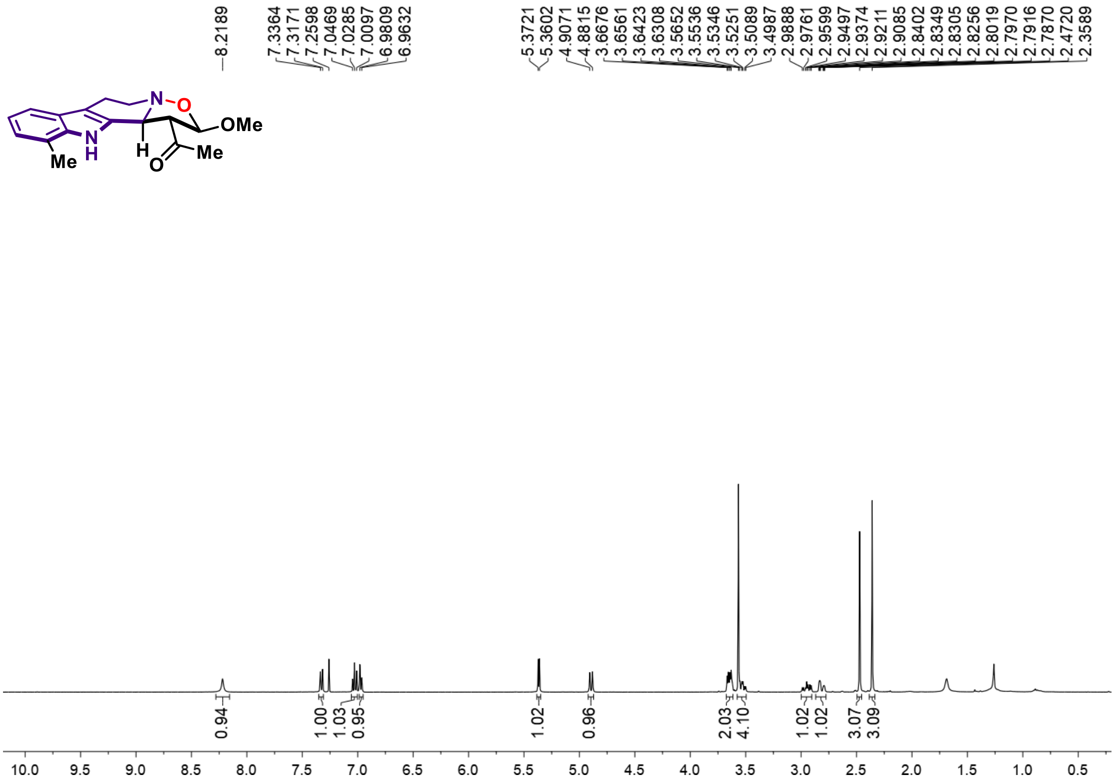

Supplementary Figure 170: ^{13}C NMR of 5r (101 MHz, $CDCl_3$)

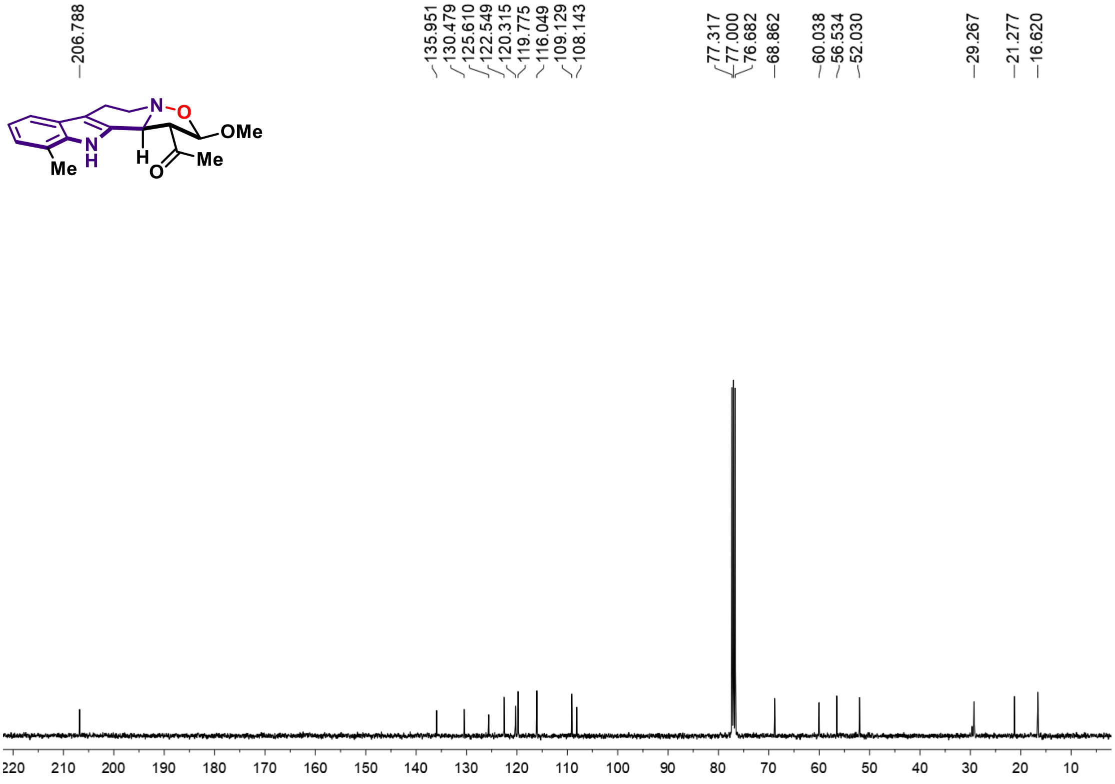

Supplementary Figure 171: HPLC spectrum of 5r

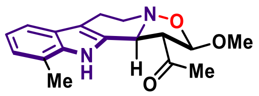

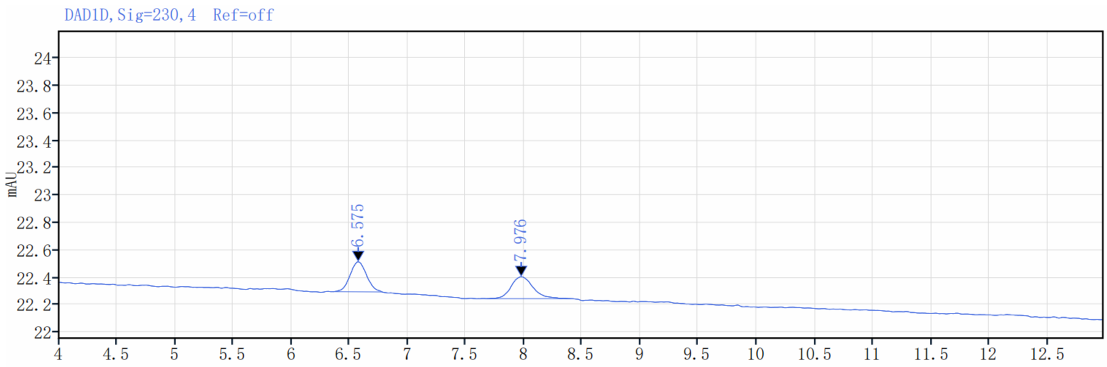

Signal : DAD1D, Sig=230, 4 Ref=off

RetTime [min]	Type	Width [min]	Area [mAU*s]	Height [mAU]	Area%
6.575	MM m	0.15	2.16	0.22	50.63
7.976	MM m	0.17	2.11	0.16	49.37
	Totals	0.32	4.27		

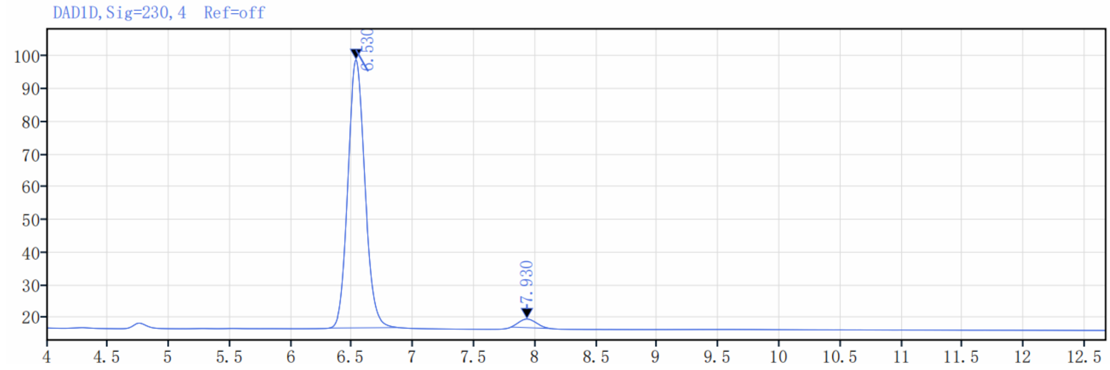

Signal : DAD1D, Sig=230, 4 Ref=off

RetTime [min]	Type	Width [min]	Area [mAU*s]	Height [mAU]	Area%
6.530	MM m	0.15	794.42	82.02	96.83
7.930	MM m	0.16	26.03	2.64	3.17
	Totals	0.31	820.45		

Supplementary Figure 172: ^{1}H NMR of 5s (400 MHz, $CDCl_3$)

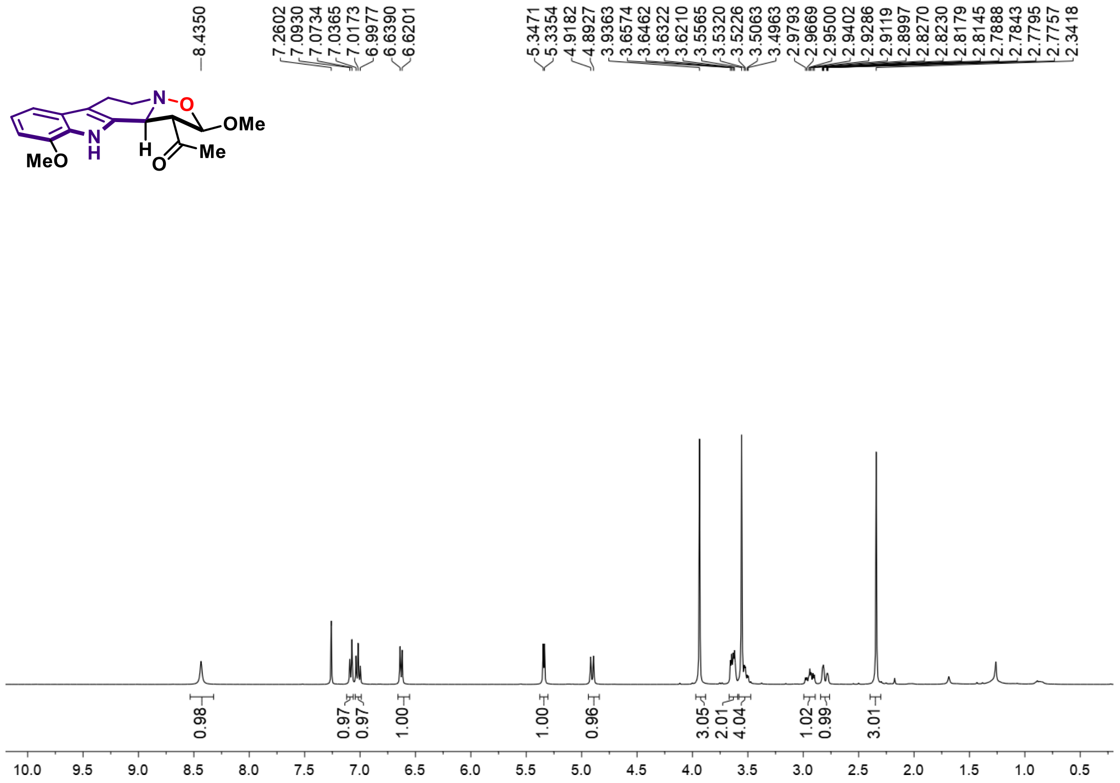

Supplementary Figure 173: ^{13}C NMR of 5s (101 MHz, $CDCl_3$)

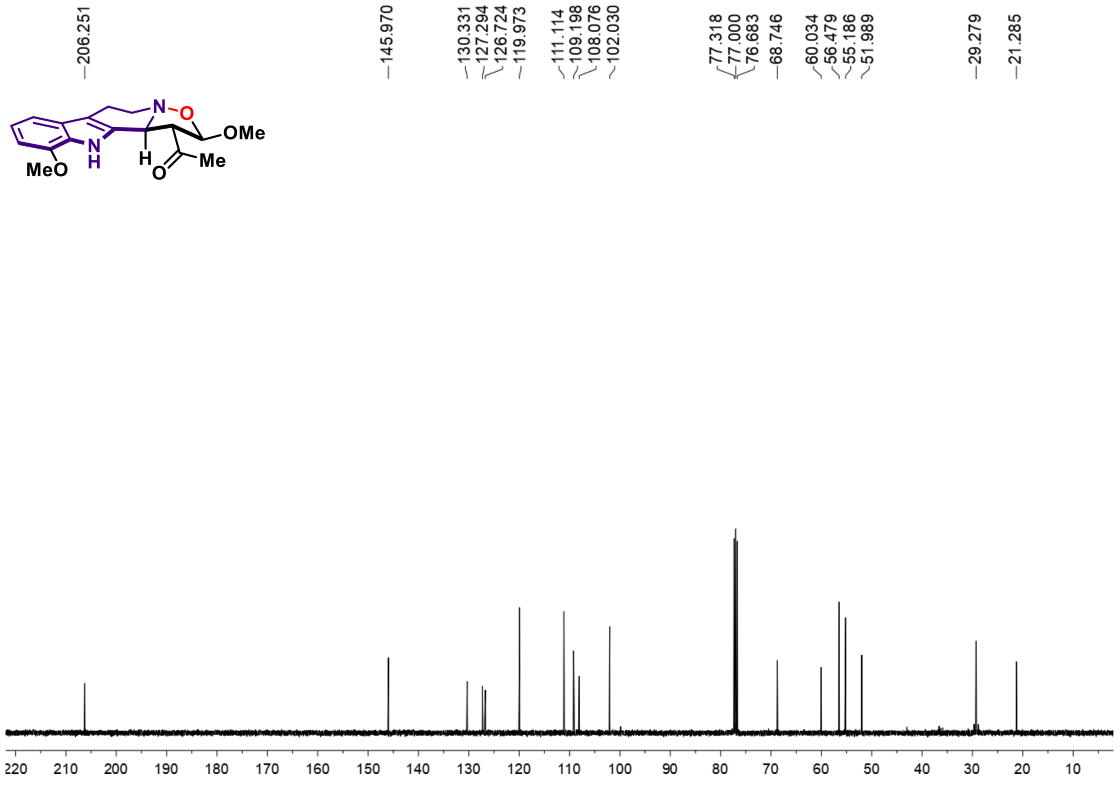

Supplementary Figure 174: HPLC spectrum of 5s

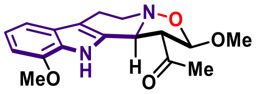

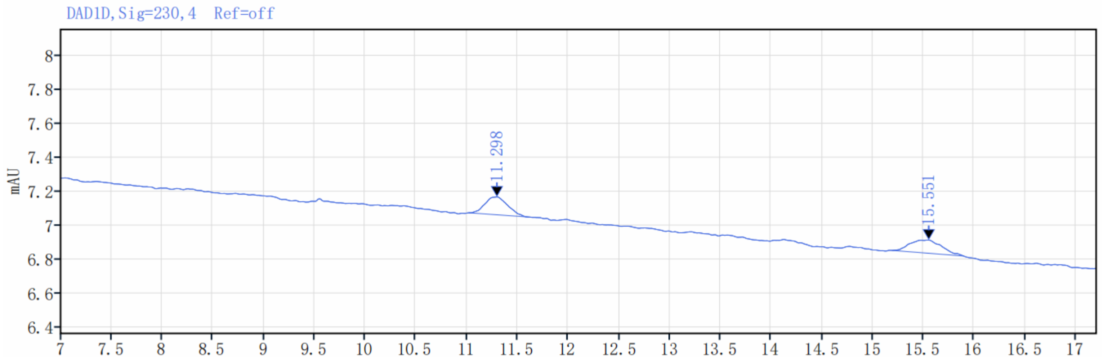

Signal : DAD1D, Sig=230, 4 Ref=off

RetTime [min]	Type	Width [min]	Area [mAU*s]	Height [mAU]	Area%
11. 298	MM m	0. 19	1. 64	0. 11	49. 57
15. 551	MM m	0. 26	1. 67	0. 08	50. 43
	Totals	0. 45	3. 30		

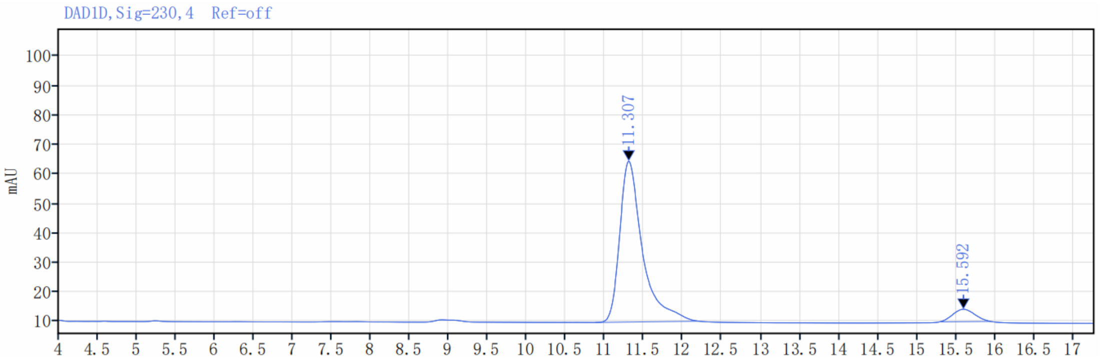

Signal : DAD1D, Sig=230, 4 Ref=off

RetTime [min]	Type	Width [min]	Area [mAU*s]	Height [mAU]	Area%
11. 307	MM m	0. 30	1104. 59	54. 53	93. 10
15. 592	MM m	0. 31	81. 88	4. 20	6. 90
	Totals	0. 61	1186. 47		

Supplementary Figure 175: ^{1}H NMR of 5t (400 MHz, $CDCl_3$)

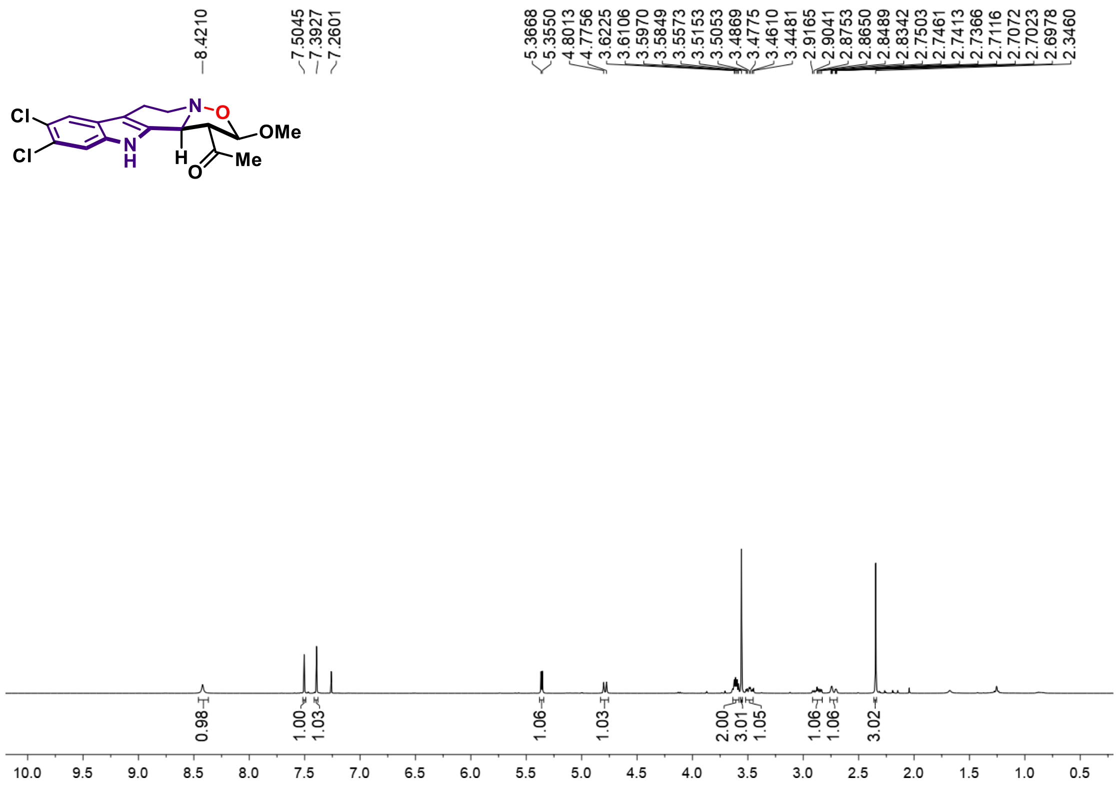

Supplementary Figure 176: ^{13}C NMR of 5t (101 MHz, $CDCl_3$)

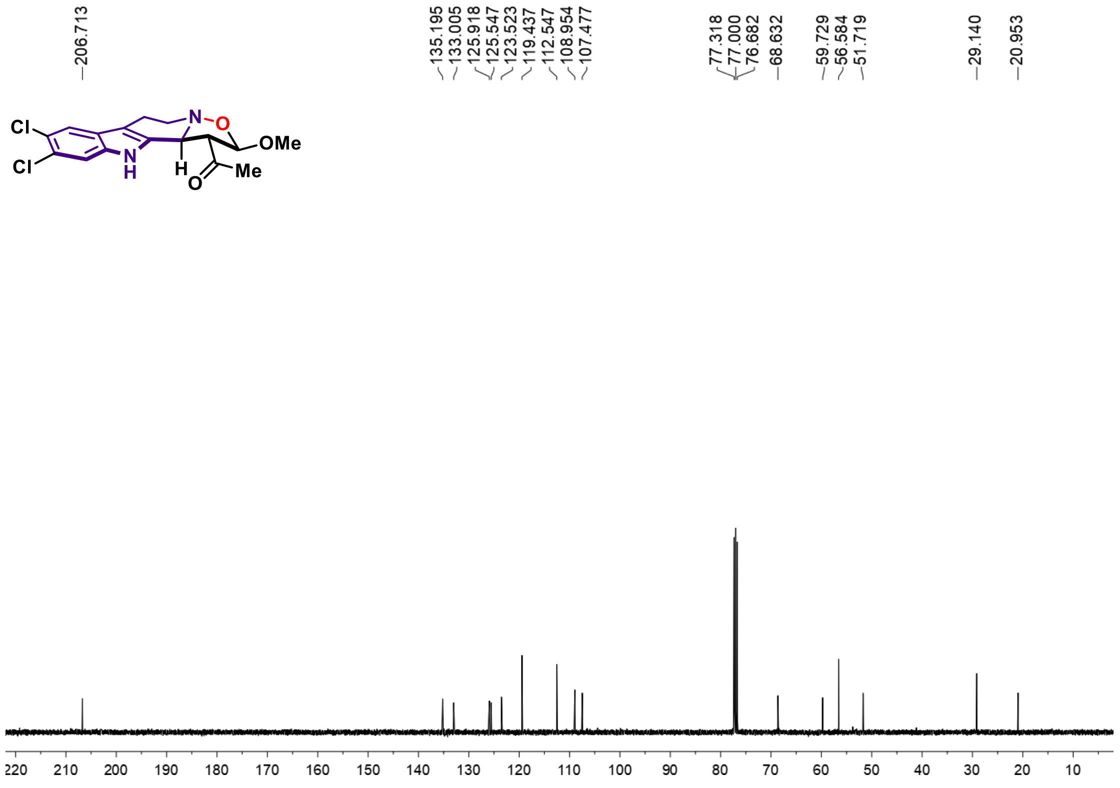

Supplementary Figure 177: HPLC spectrum of 5t

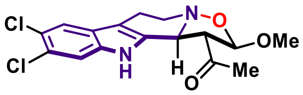

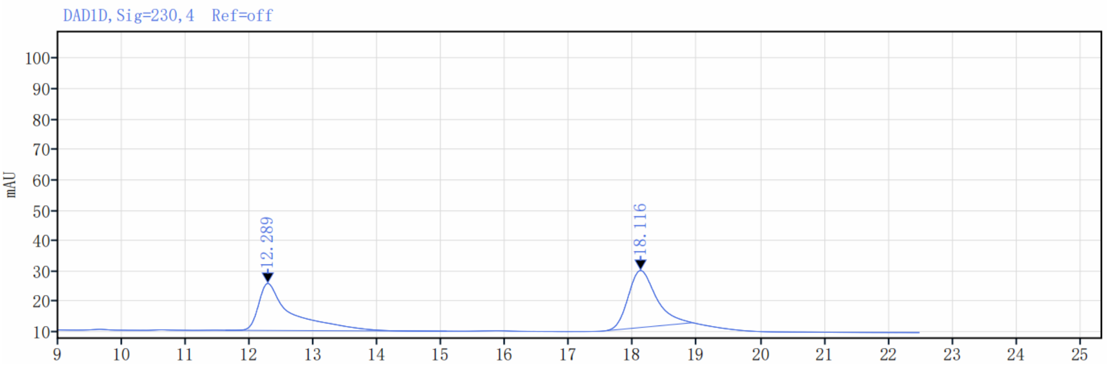

Signal : DAD1D, Sig=230, 4 Ref=off

RetTime [min]	Type	Width [min]	Area [mAU*s]	Height [mAU]	Area%
12.289	MM m	0.50	559.15	15.46	49.36
18.116	MM m	0.46	573.61	18.75	50.64
	Totals	0.96	1132.76		

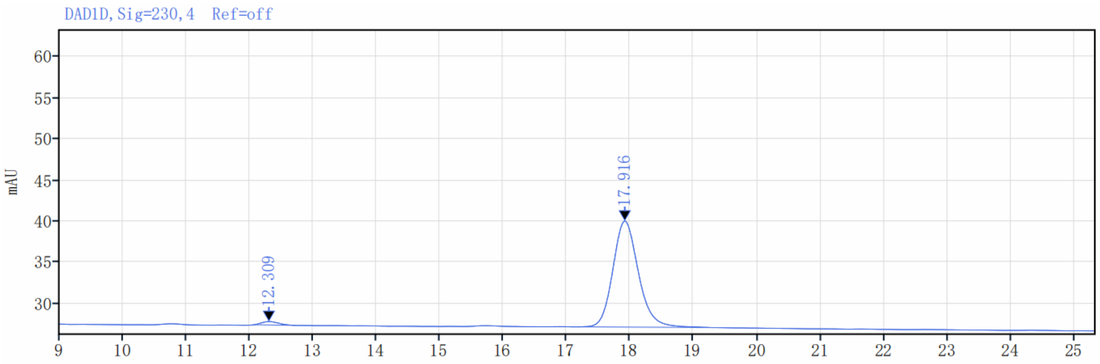

Signal : DAD1D, Sig=230, 4 Ref=off

RetTime [min]	Type	Width [min]	Area [mAU*s]	Height [mAU]	Area%
12.309	MM m	0.26	7.57	0.41	2.06
17.916	MM m	0.43	359.73	12.88	97.94
	Totals	0.68	367.30		

Supplementary Figure 178: ^{1}H NMR of 7a (400 MHz, CDCl$_3$)

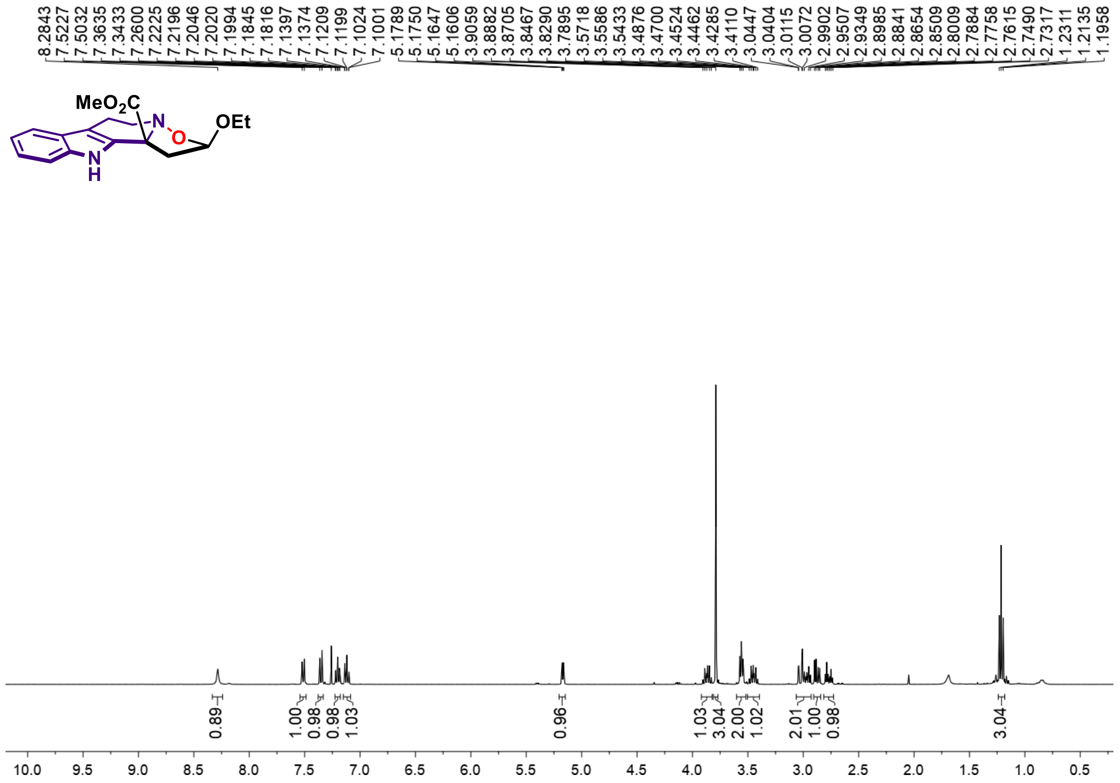

Supplementary Figure 179: ^{13}C NMR of 7a (101 MHz, CDCl$_3$)

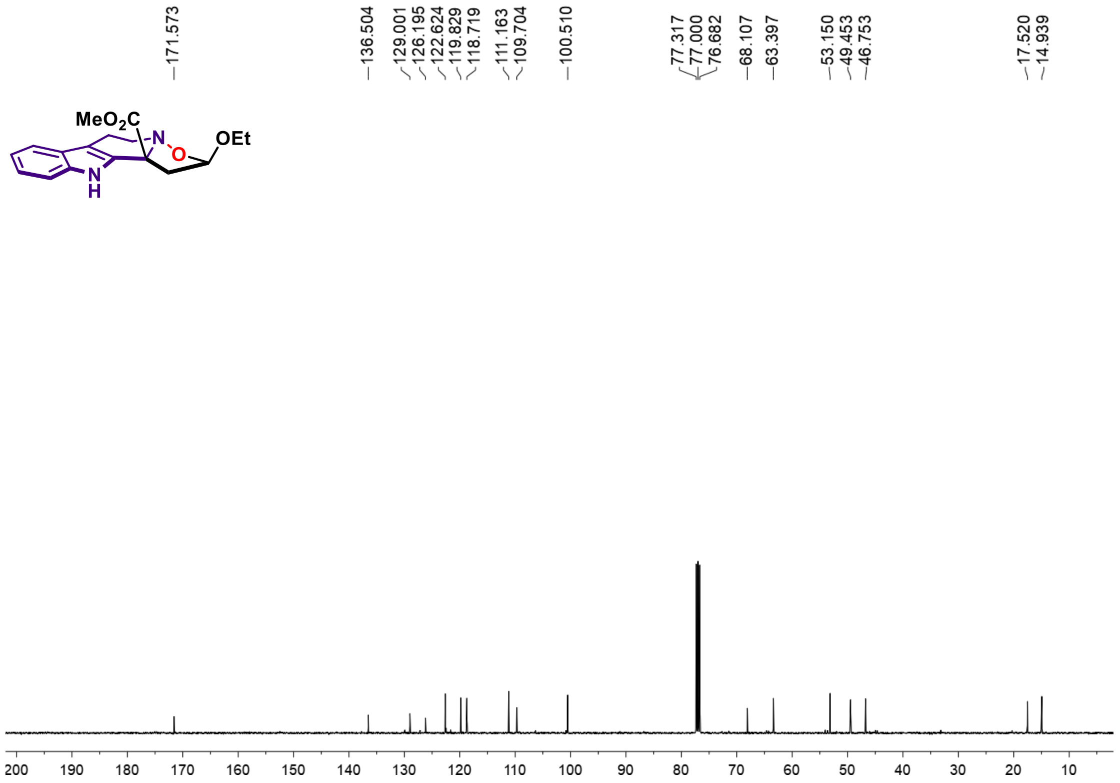

Supplementary Figure 180: HPLC spectrum of 7a

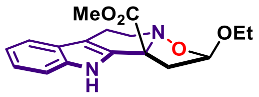

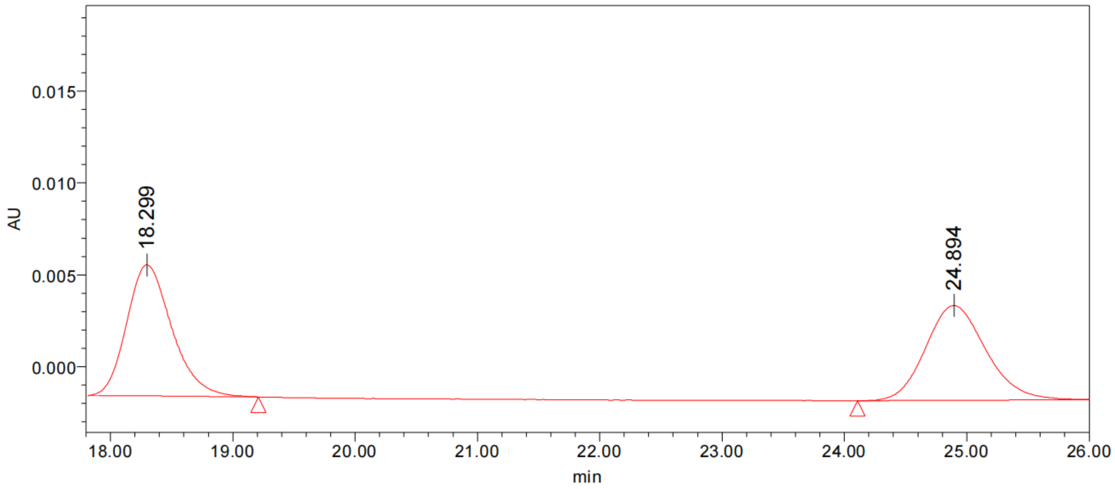

	RetTime [min]	Area [mAU*s]	Area%
1	18.299	182647	50.77
2	24.894	177112	49.23

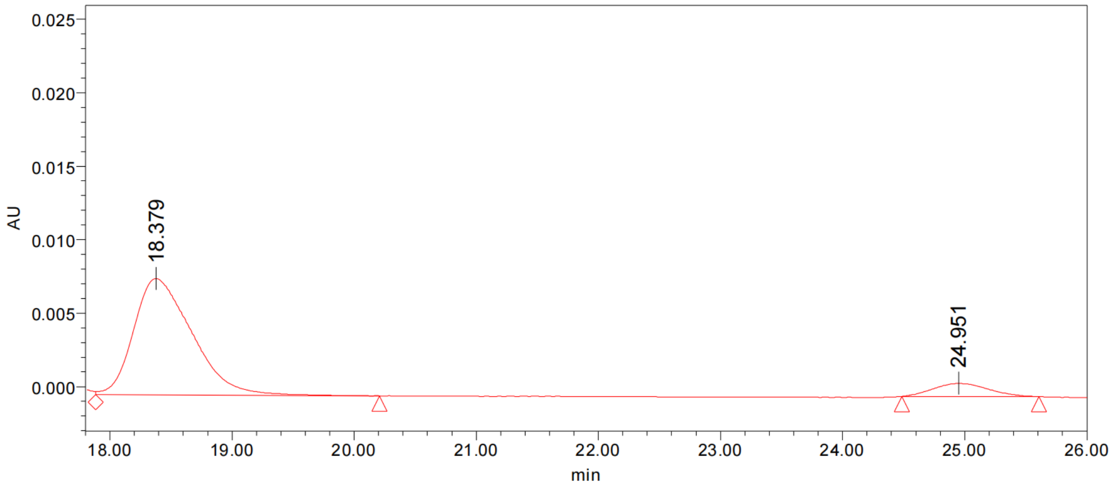

	RetTime [min]	Area [mAU*s]	Area%
1	18.379	260352	90.25
2	24.951	28118	9.75

Supplementary Figure 181: ^{1}H NMR of 7b (400 MHz, $CDCl_3$)

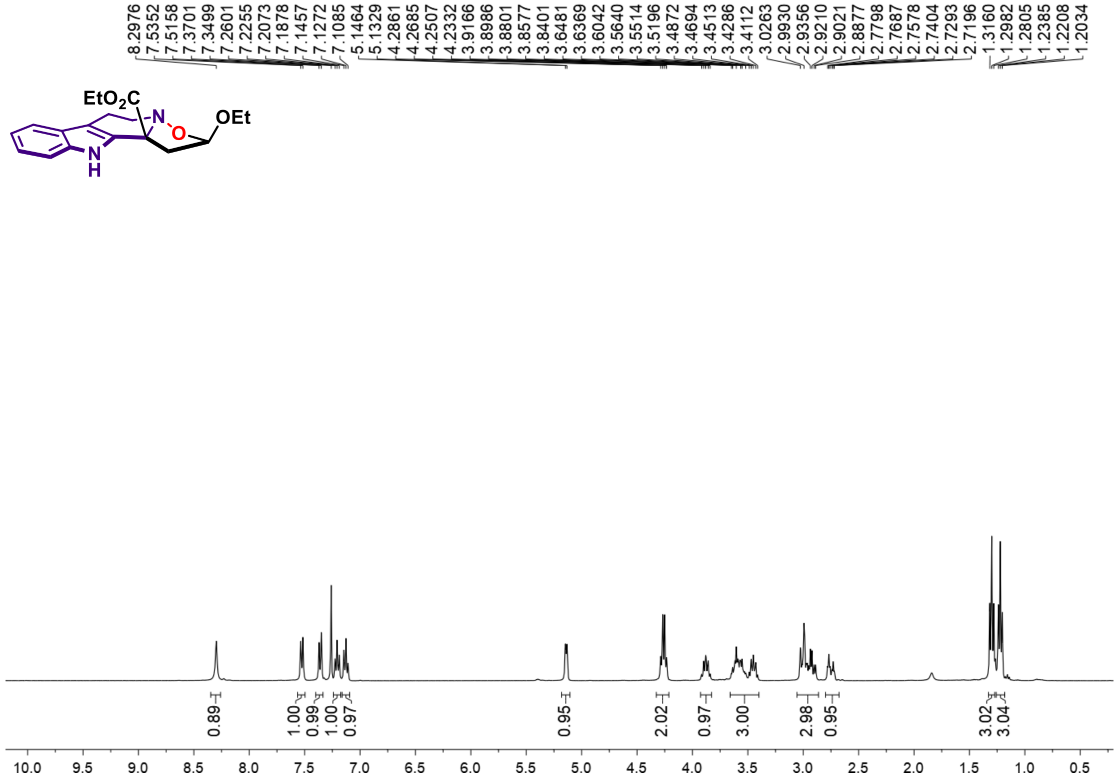

Supplementary Figure 182: ^{13}C NMR of 7b (101 MHz, $CDCl_3$)

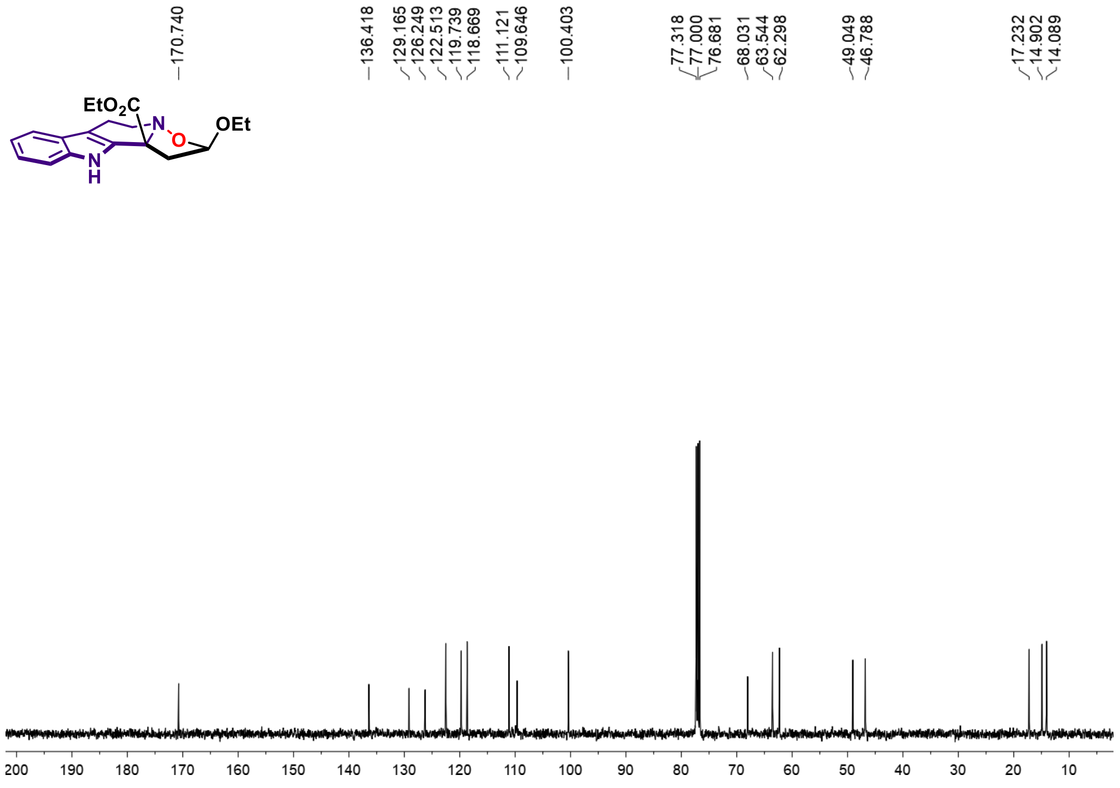

Supplementary Figure 183: HPLC spectrum of 7b

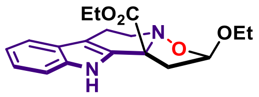

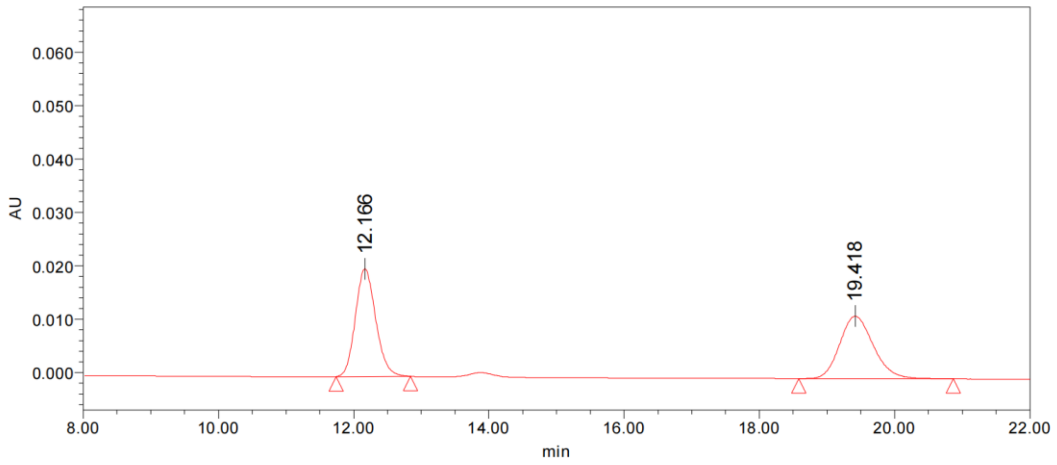

	RetTime [min]	Area [mAU*s]	Area%
1	12.166	421506	50.97
2	19.418	405541	49.03

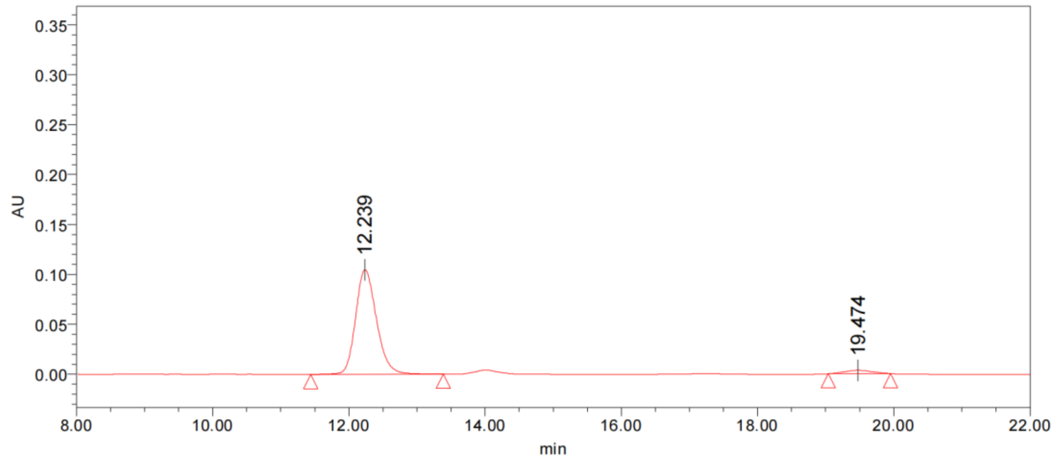

	RetTime [min]	Area [mAU*s]	Area%
1	12.239	2222646	95.93
2	19.474	94259	4.07

Supplementary Figure 184: ^{1}H NMR of 7c (400 MHz, $CDCl_3$)

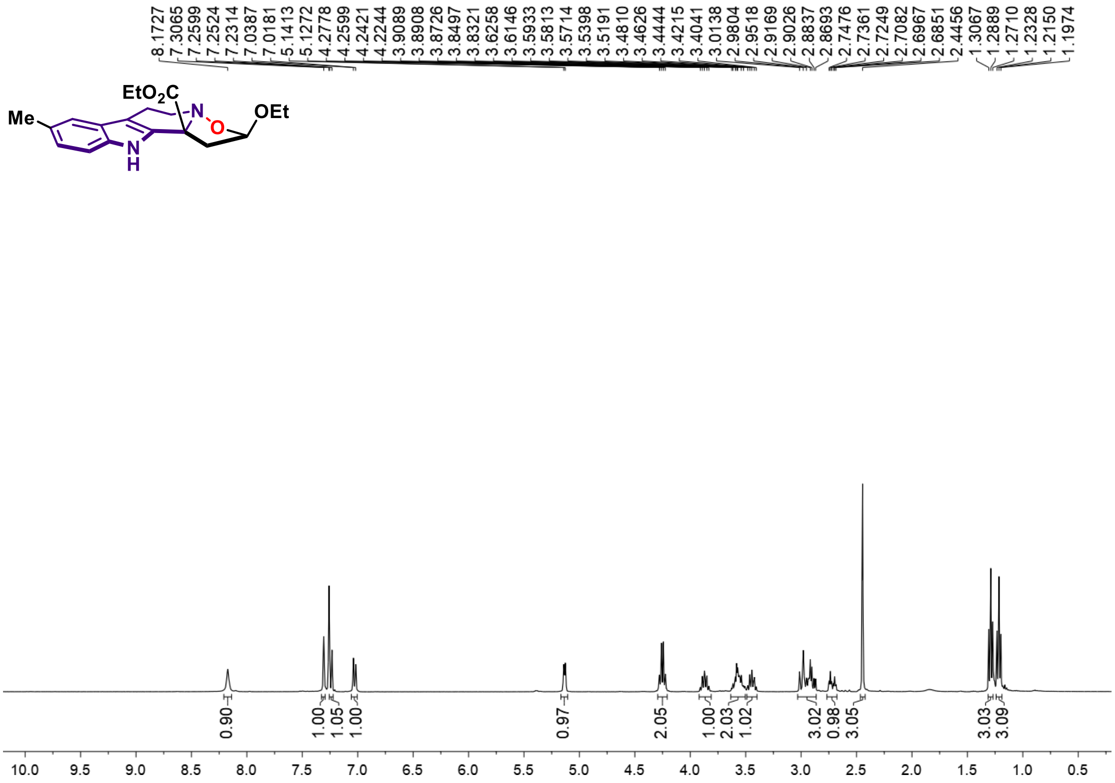

Supplementary Figure 185: ^{13}C NMR of 7c (101 MHz, $CDCl_3$)

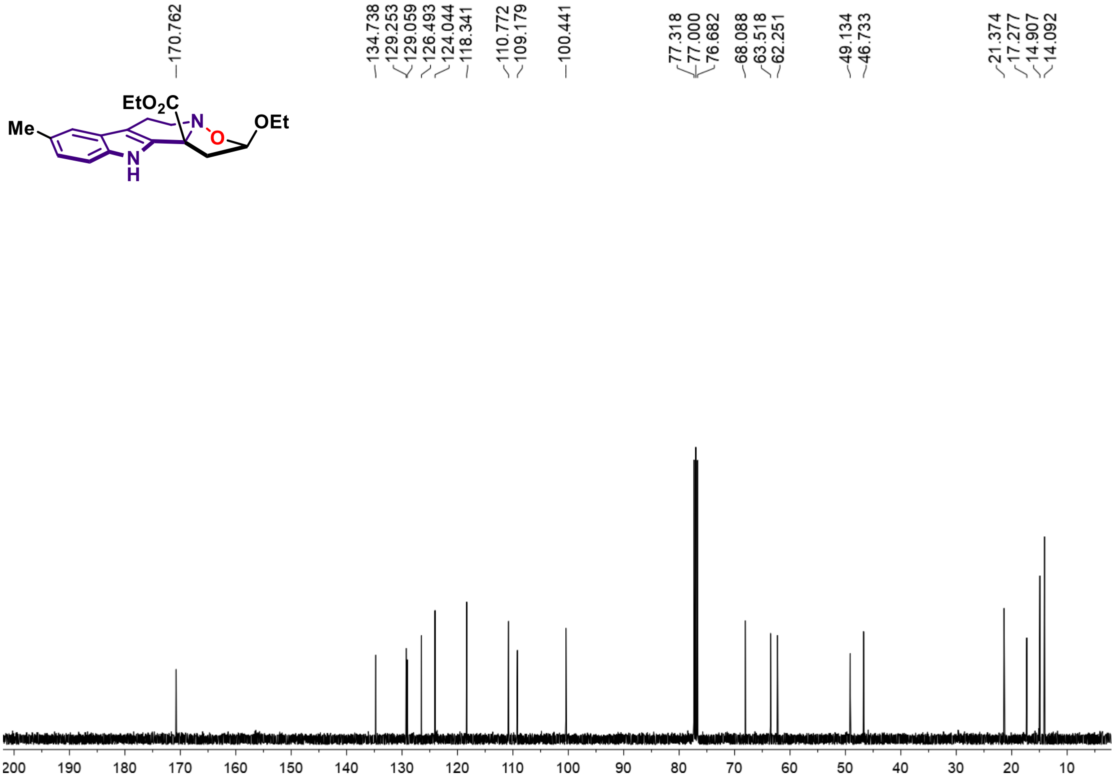

Supplementary Figure 186: HPLC spectrum of 7c

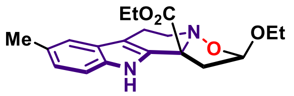

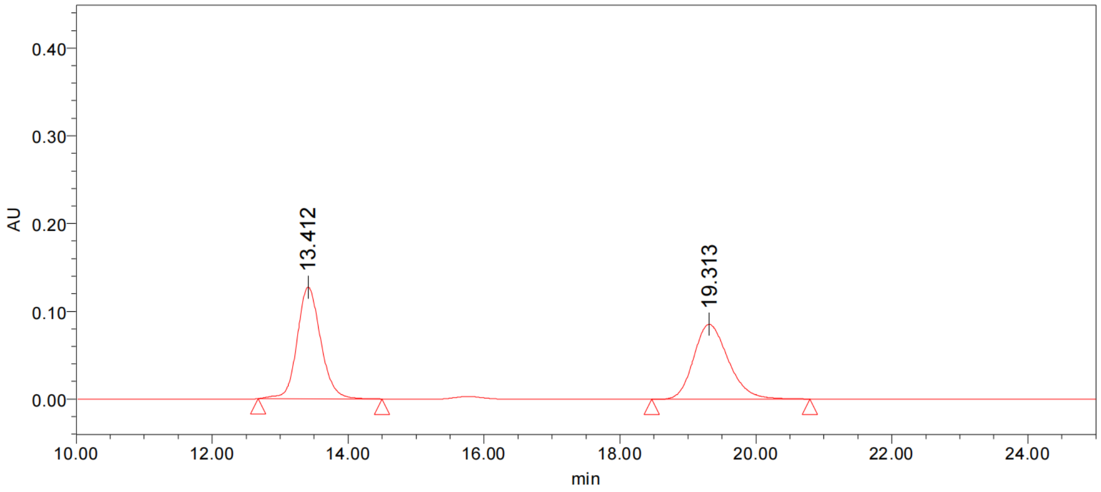

	RetTime [min]	Area [mAU*s]	Area%
1	13.412	3064881	50.42
2	19.313	3014236	49.58

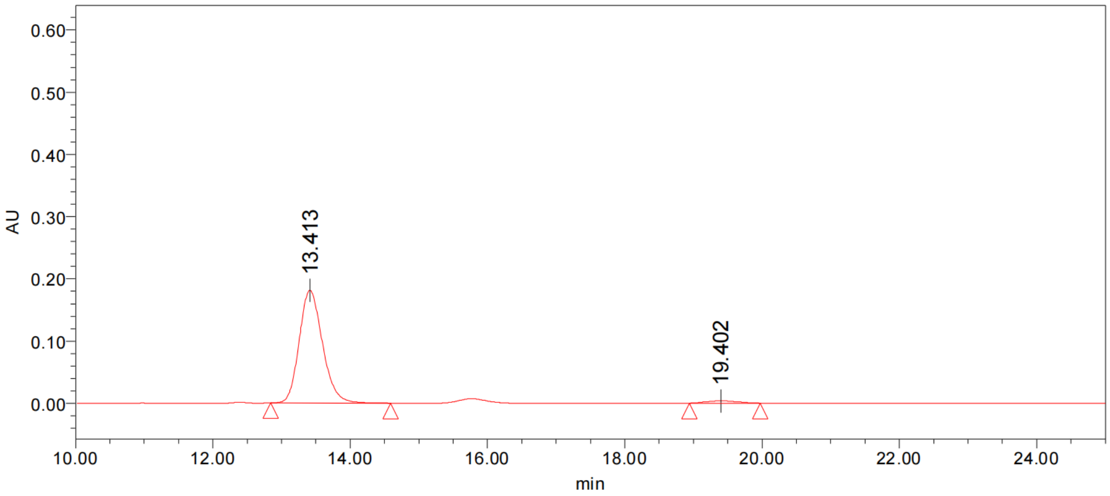

	RetTime [min]	Area [mAU*s]	Area%
1	13.413	4261782	97.40
2	19.402	113857	2.60

Supplementary Figure 187: ^{1}H NMR of 7d (400 MHz, $CDCl_3$)

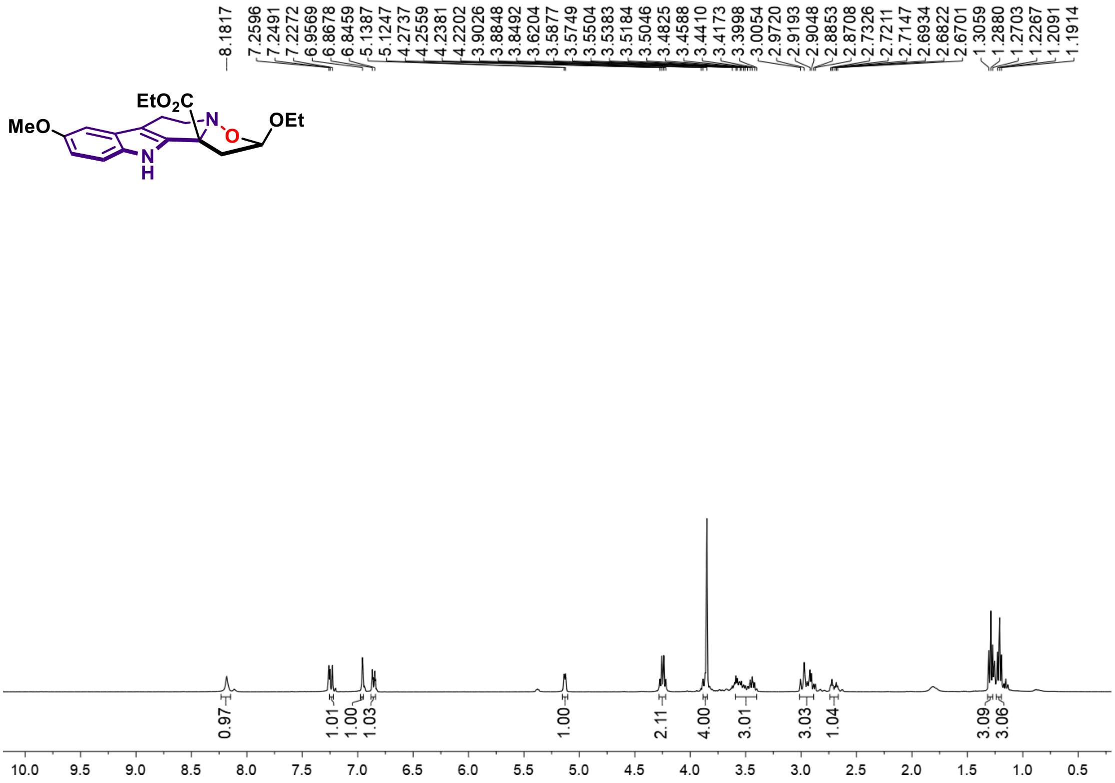

Supplementary Figure 188: ^{13}C NMR of 7d (101 MHz, $CDCl_3$)

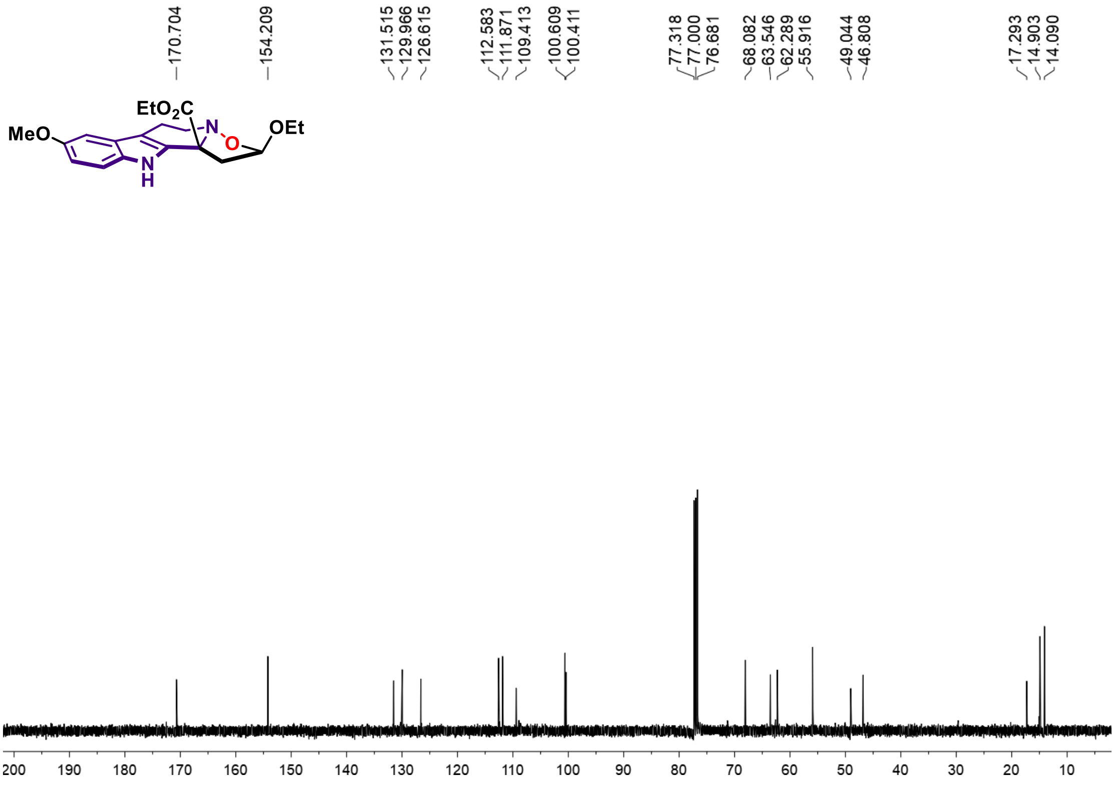

Supplementary Figure 189: HPLC spectrum of 7d

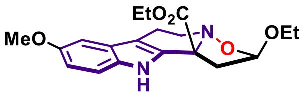

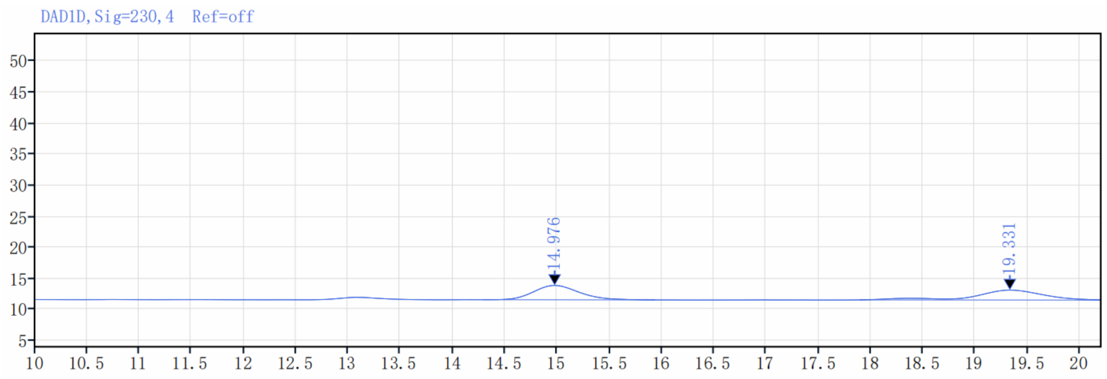

Signal : DAD1D, Sig=230, 4 Ref=off

RetTime [min]	Type	Width [min]	Area [mAU*s]	Height [mAU]	Area%
14.976	MM m	0.45	70.16	2.31	49.05
19.331	MM m	0.60	72.88	1.60	50.95
	Totals	1.05	143.05		

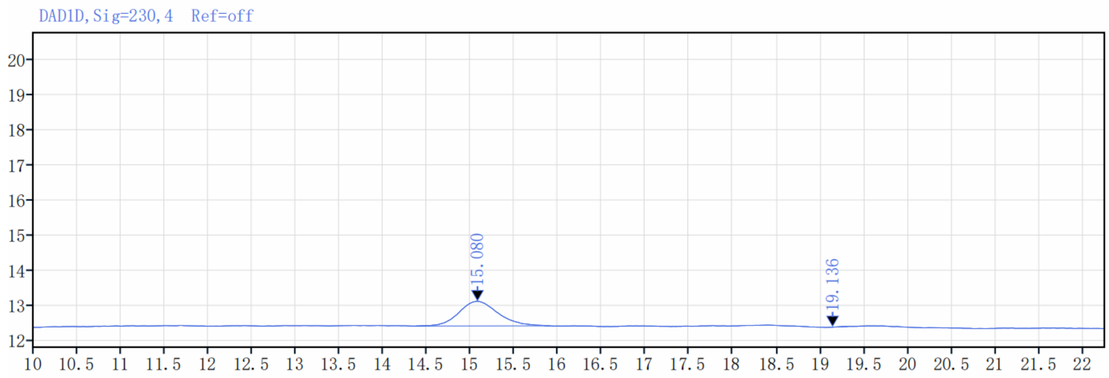

Signal : DAD1D, Sig=230, 4 Ref=off

RetTime [min]	Type	Width [min]	Area [mAU*s]	Height [mAU]	Area%
15.080	MM m	0.38	21.77	0.70	99.99
19.136	MM n	0.02	0.00	0.00	0.01
	Totals	0.40	21.77		

Supplementary Figure 190: ^{1}H NMR of 7e (400 MHz, $CDCl_3$)

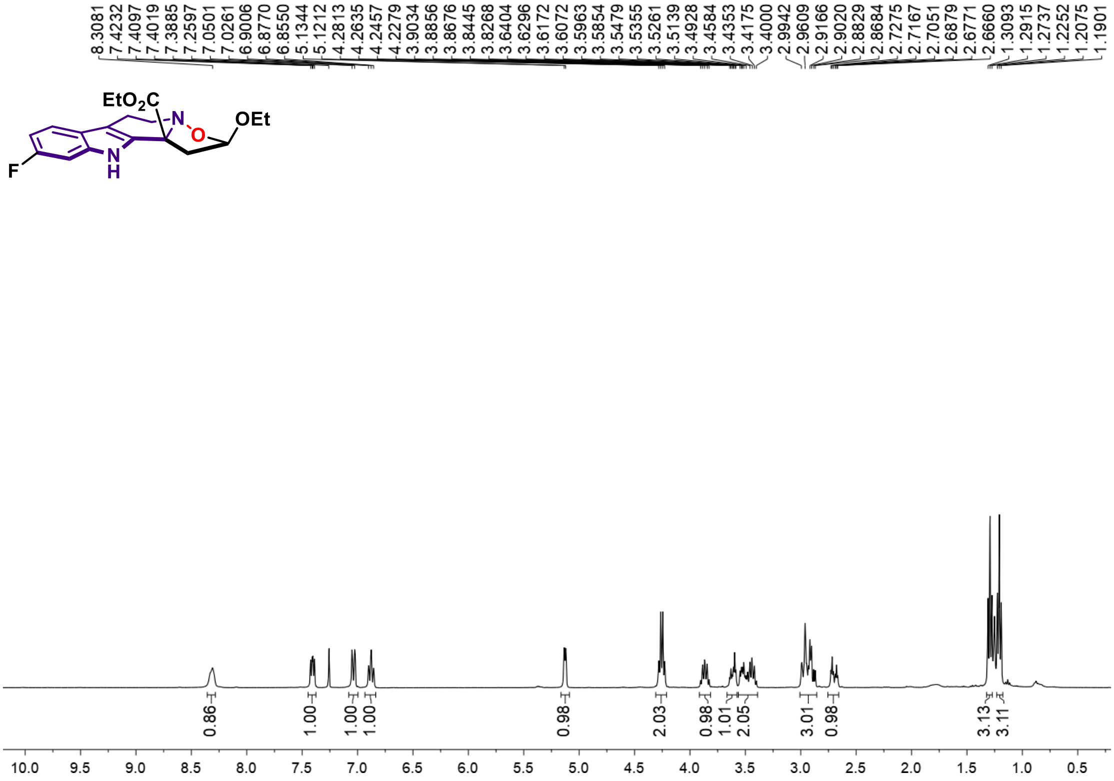

Supplementary Figure 191: ^{13}C NMR of 7e (101 MHz, $CDCl_3$)

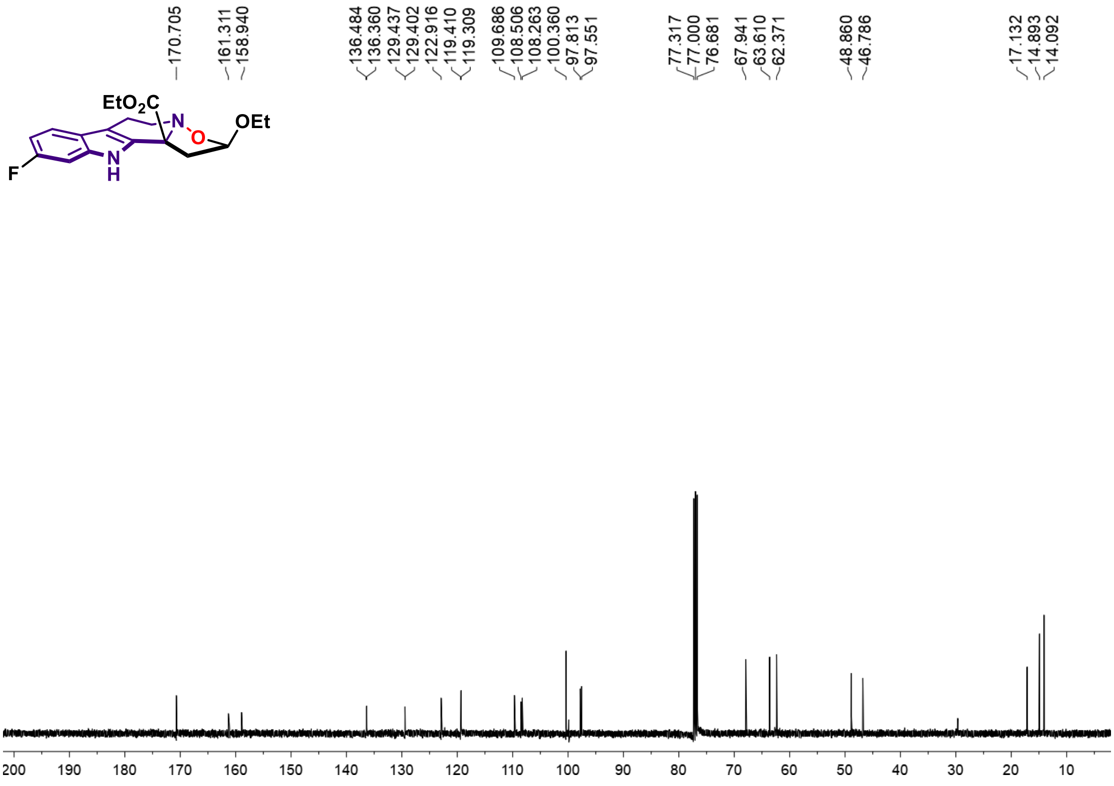

Supplementary Figure 192: HPLC spectrum of 7e

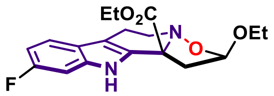

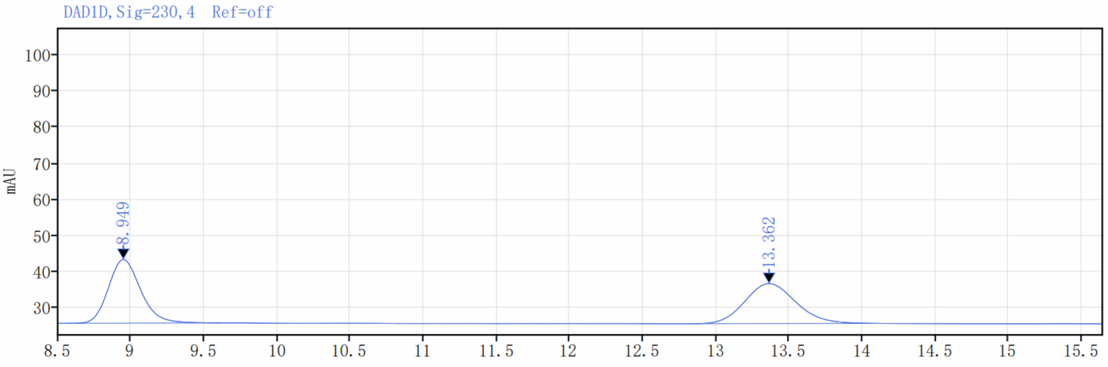

Signal : DAD1D, Sig=230, 4 Ref=off

RetTime [min]	Type	Width [min]	Area [mAU*s]	Height [mAU]	Area%
8. 949	MM m	0. 24	271. 23	17. 68	50. 24
13. 362	MM m	0. 37	268. 66	11. 10	49. 76
	Totals	0. 61	539. 90		

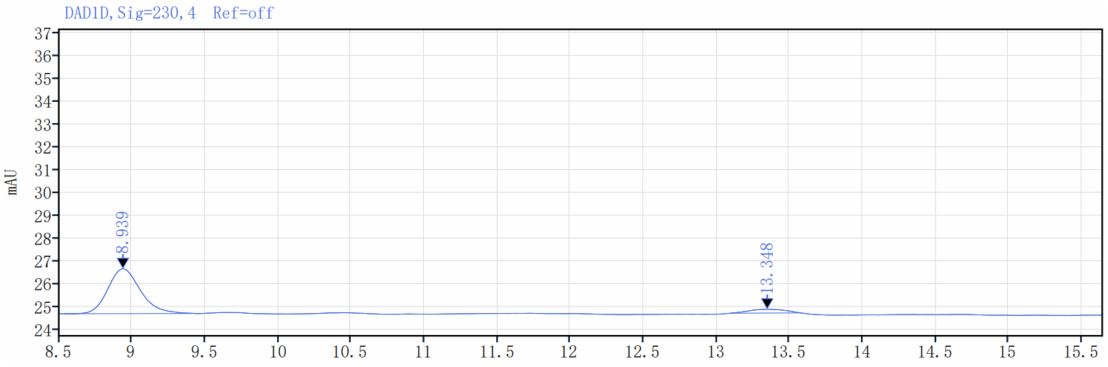

Signal : DAD1D, Sig=230, 4 Ref=off

RetTime [min]	Type	Width [min]	Area [mAU*s]	Height [mAU]	Area%
8. 939	MM m	0. 24	30. 47	1. 97	91. 45
13. 348	MM m	0. 21	2. 85	0. 17	8. 55
	Totals	0. 44	33. 32		

Supplementary Figure 193: ^{1}H NMR of 7f (400 MHz, $CDCl_3$)

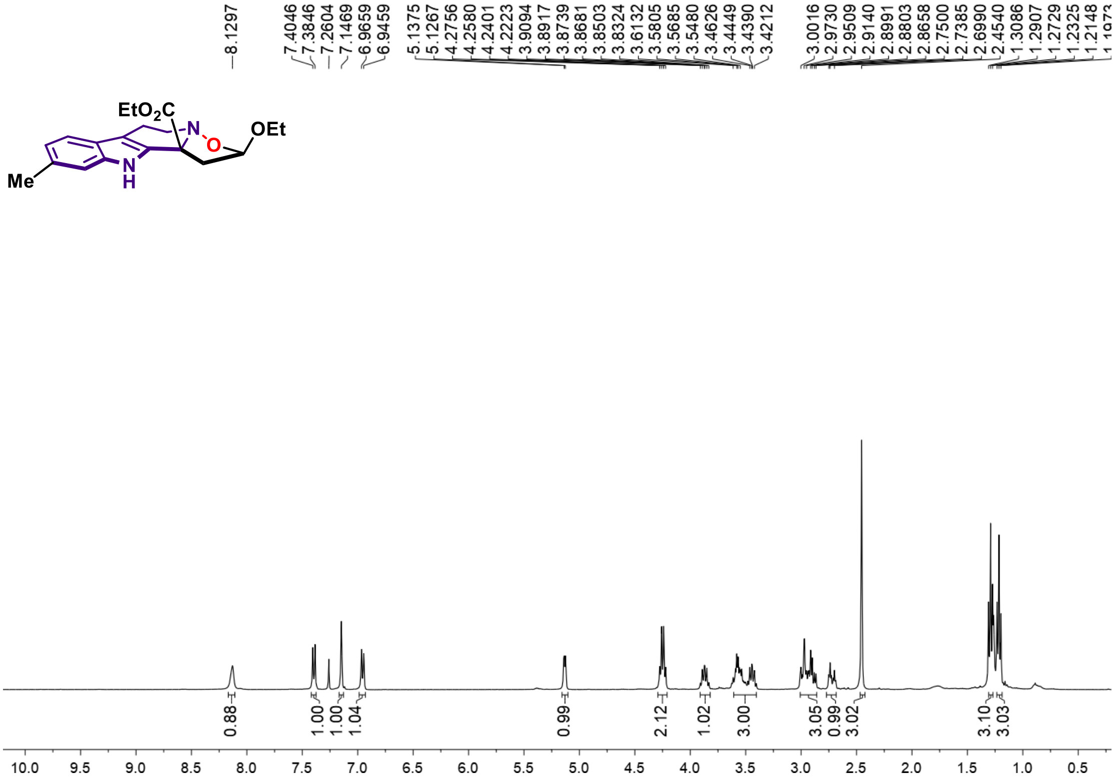

Supplementary Figure 194: ^{13}C NMR of 7f (101 MHz, $CDCl_3$)

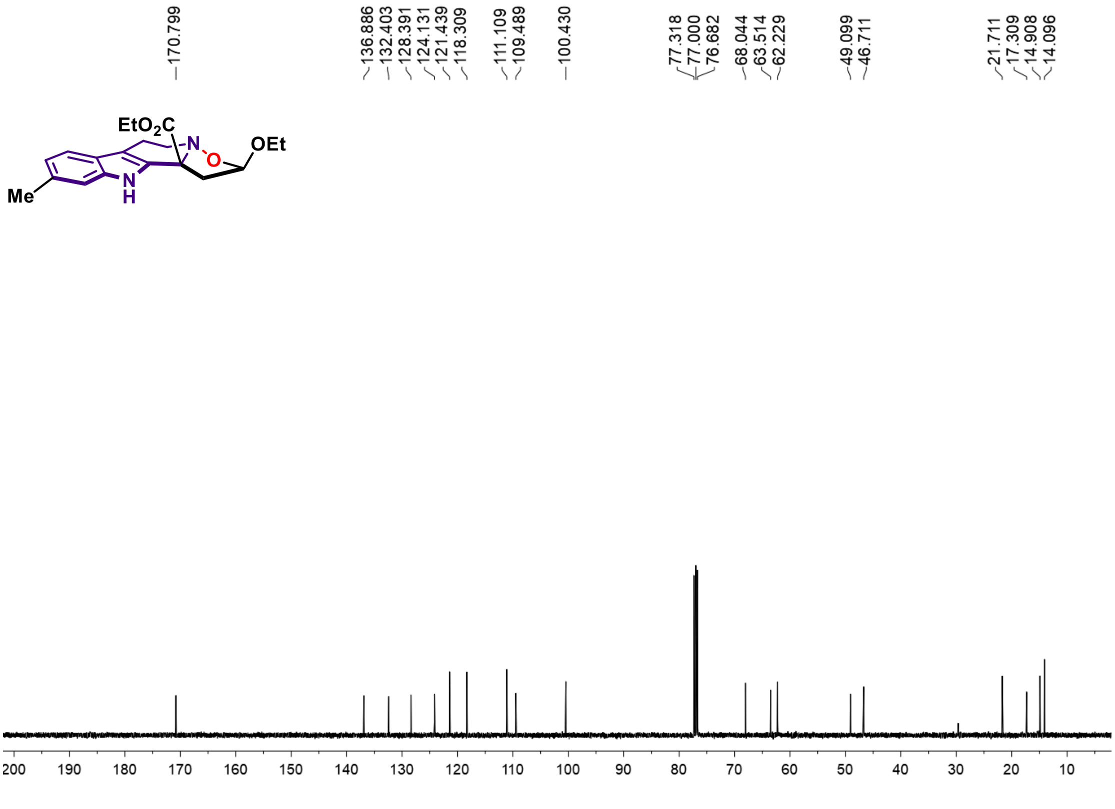

Supplementary Figure 195: HPLC spectrum of 7f

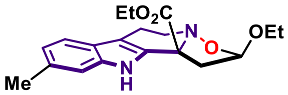

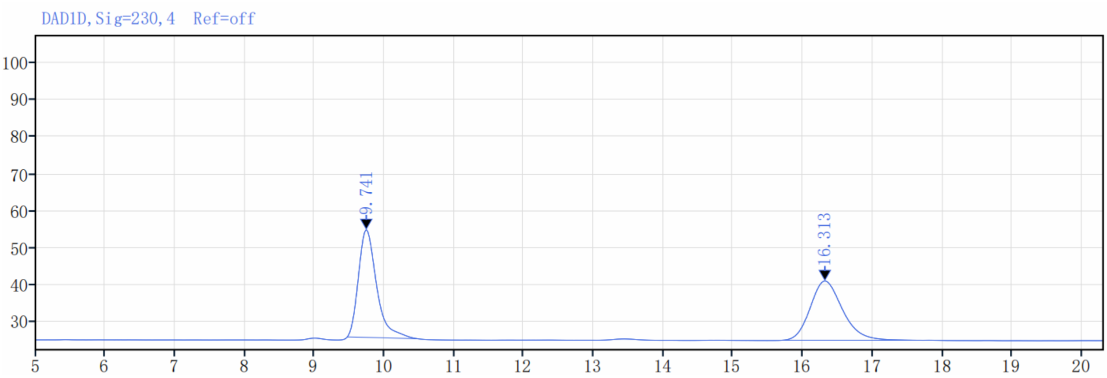

Signal : DAD1D, Sig=230, 4 Ref=off

RetTime [min]	Type	Width [min]	Area [mAU*s]	Height [mAU]	Area%
9.741	MM m	0.28	527.94	29.13	50.58
16.313	MM m	0.49	515.94	16.05	49.42
	Totals	0.77	1043.88		

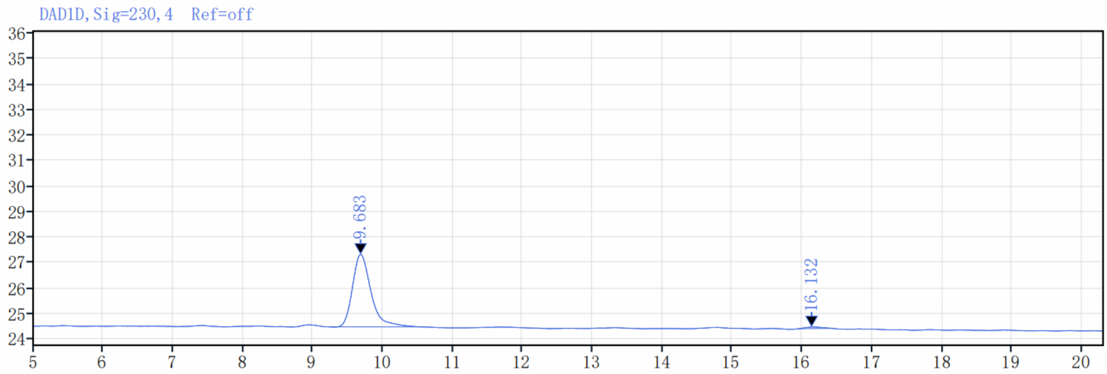

Signal : DAD1D, Sig=230, 4 Ref=off

RetTime [min]	Type	Width [min]	Area [mAU*s]	Height [mAU]	Area%
9.683	MM m	0.28	52.08	2.84	98.04
16.132	MM m	0.20	1.04	0.06	1.96
	Totals	0.48	53.12		

Supplementary Figure 196: ^{1}H NMR of 7g (400 MHz, $CDCl_3$)

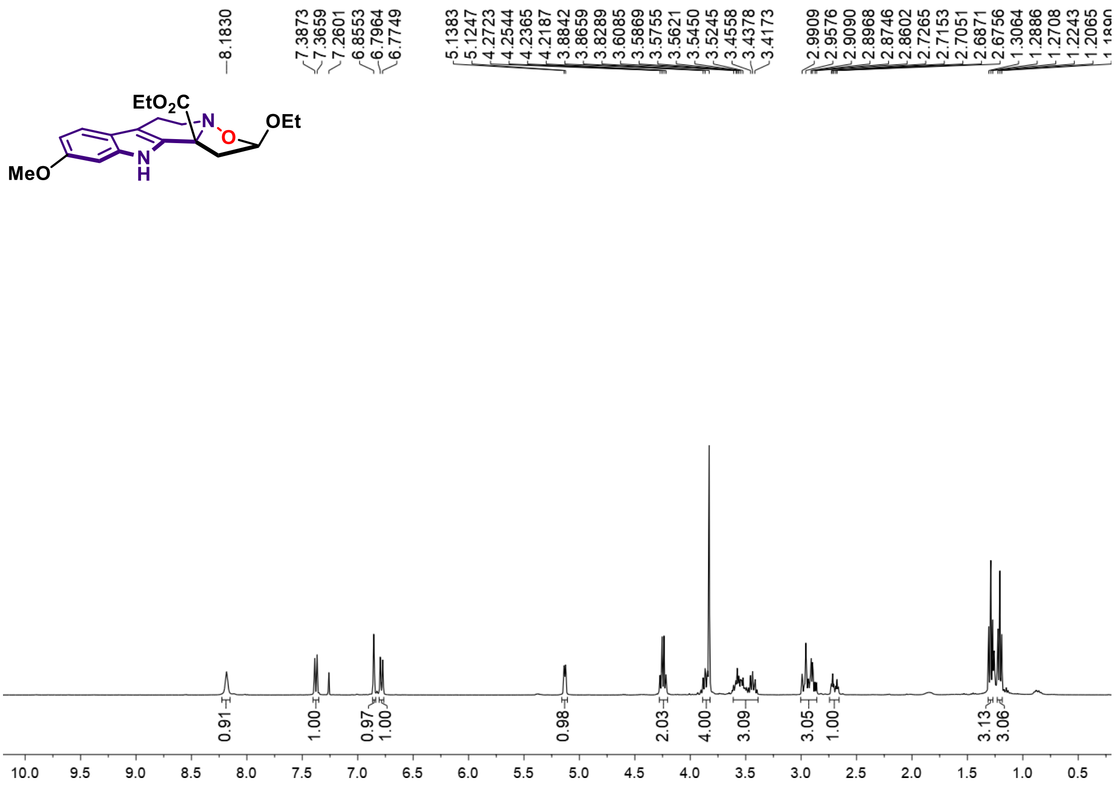

Supplementary Figure 197: ^{13}C NMR of 7g (101 MHz, $CDCl_3$)

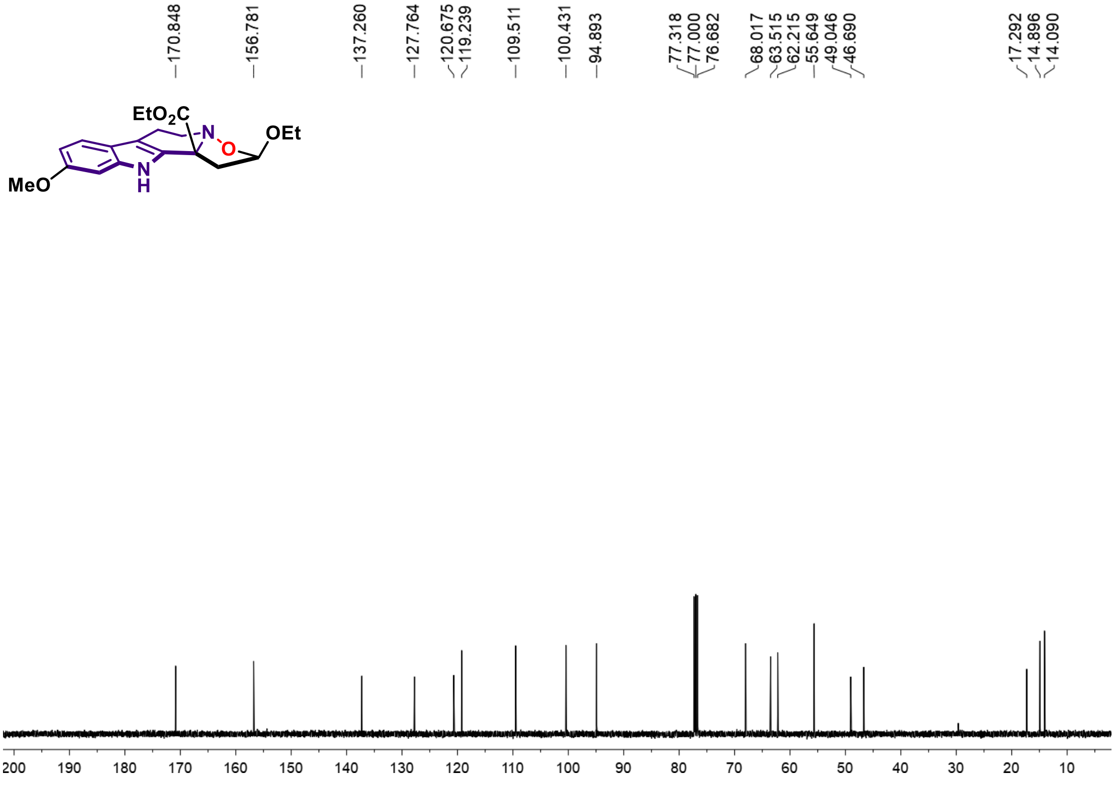

Supplementary Figure 198: HPLC spectrum of 7g

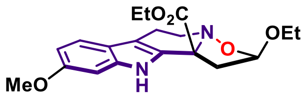

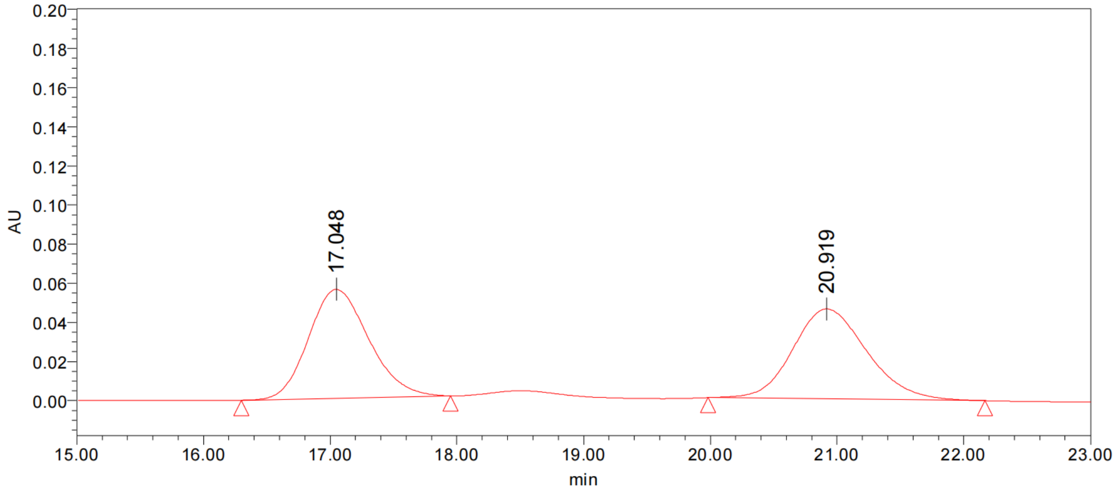

	RetTime [min]	Area [mAU*s]	Area%
1	17.048	1835158	49.58
2	20.919	1866218	50.42

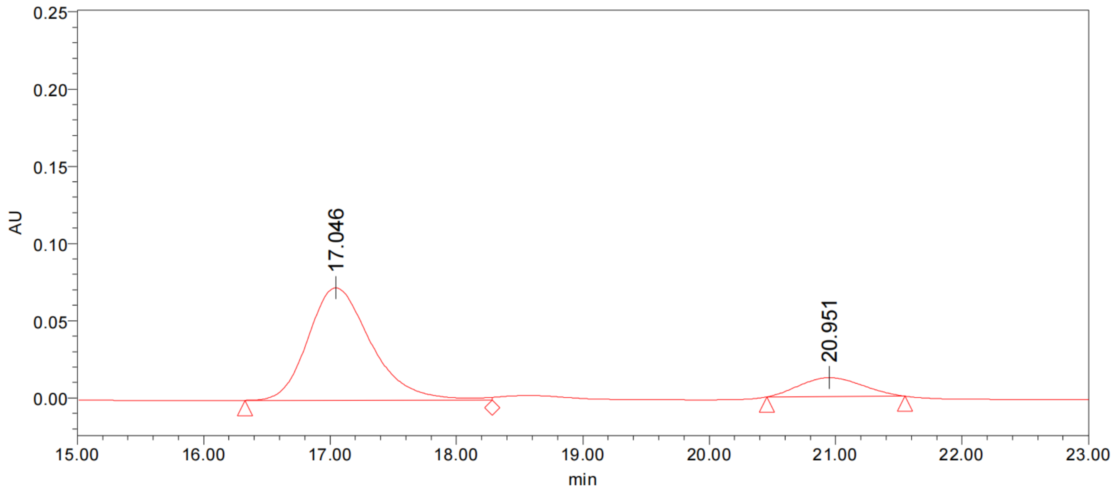

	RetTime [min]	Area [mAU*s]	Area%
1	17.046	2517342	85.73
2	20.951	419157	14.27

Supplementary Figure 199: ^{1}H NMR of 7h (400 MHz, $CDCl_3$)

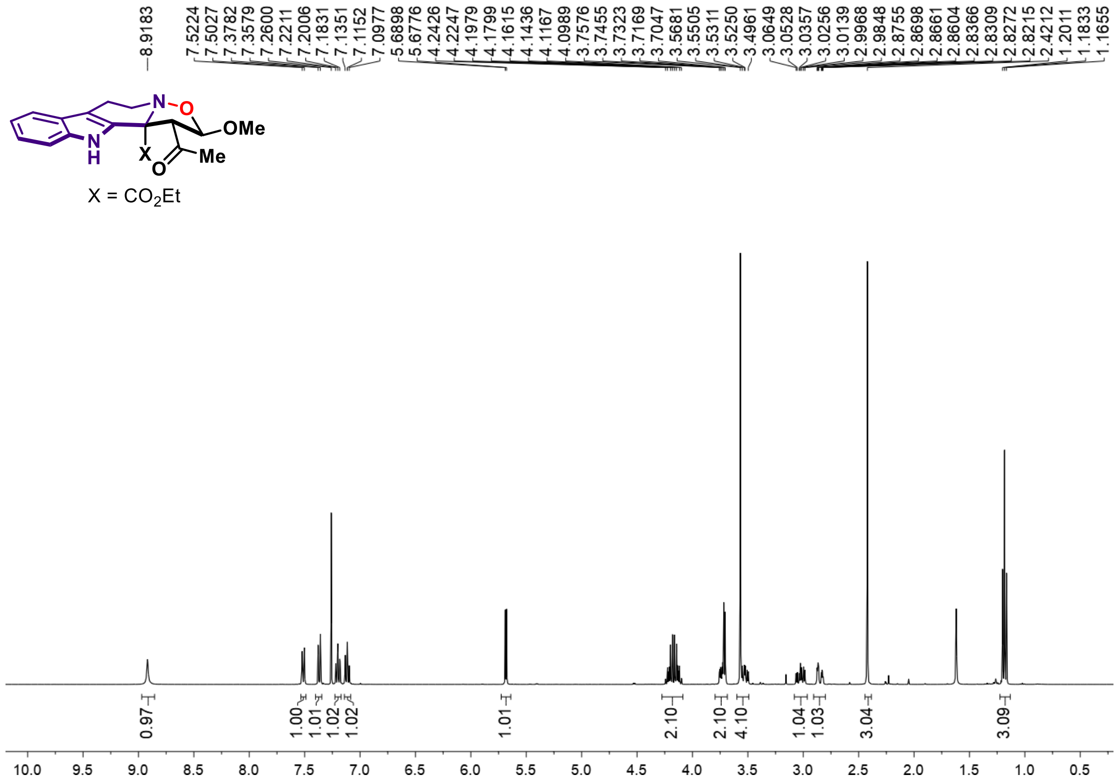

Supplementary Figure 200: ^{13}C NMR of 7h (101 MHz, $CDCl_3$)

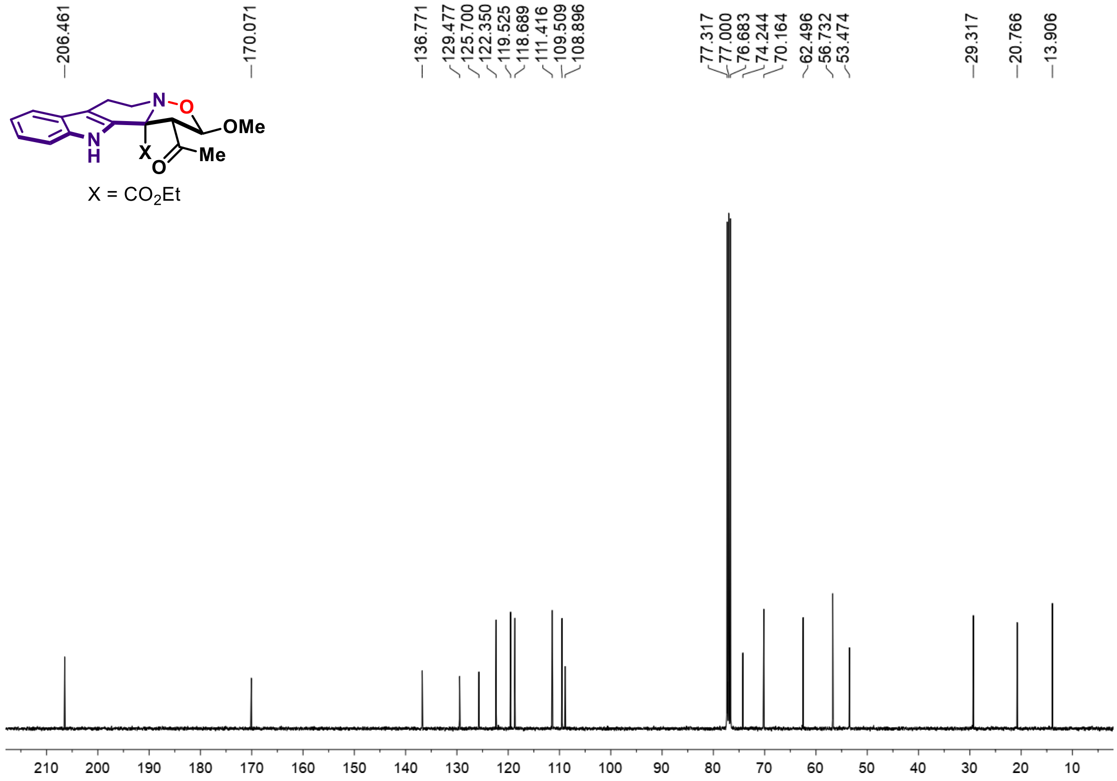

Supplementary Figure 201: HPLC spectrum of 7h

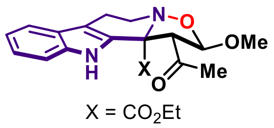

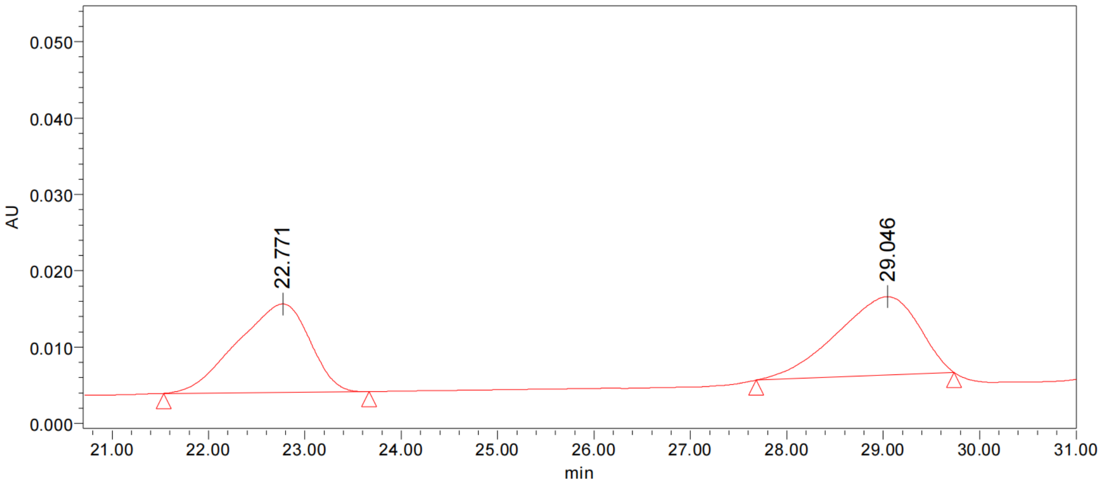

	RetTime [min]	Area [mAU*s]	Area%
1	22.771	603904	49.98
2	29.046	604488	50.02

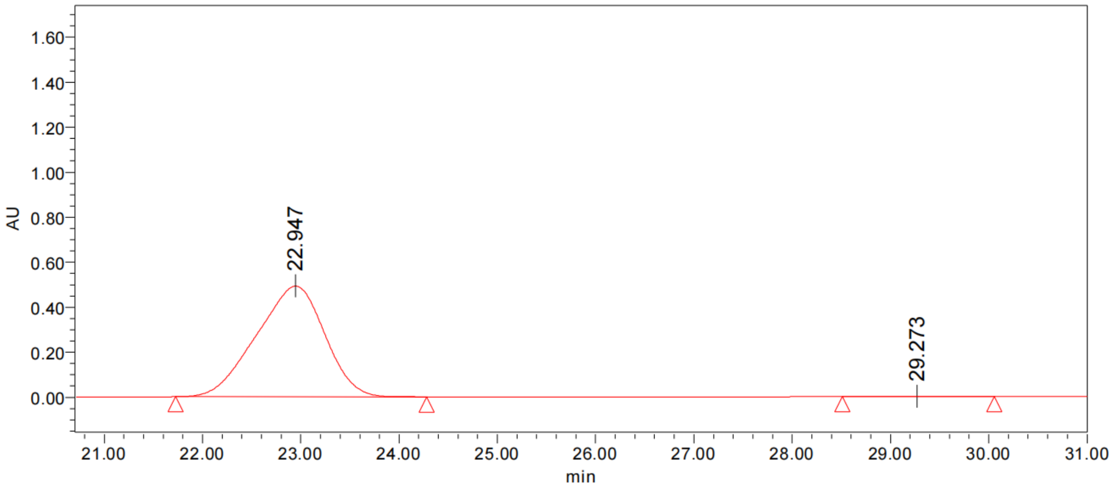

	RetTime [min]	Area [mAU*s]	Area%
1	22.947	23803101	99.69
2	29.273	74306	0.31

Supplementary Figure 202: ^{1}H NMR of 8a (400 MHz, $CDCl_3$)

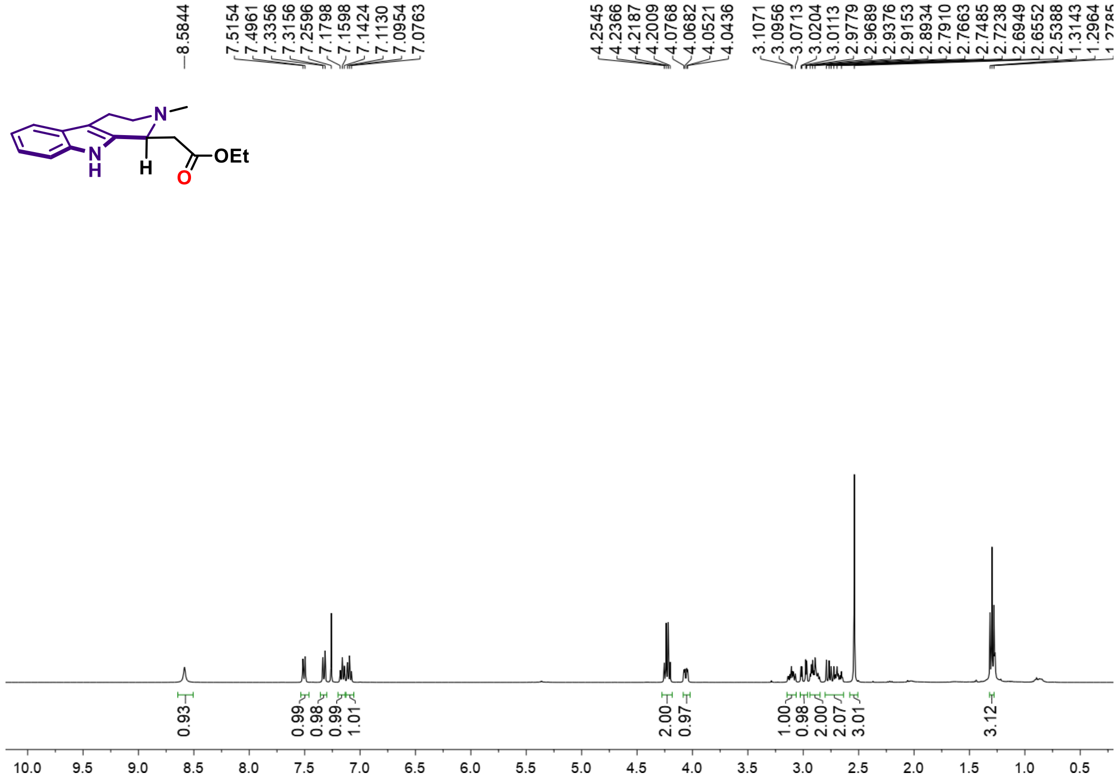

Supplementary Figure 203: ^{13}C NMR of 8a (101 MHz, $CDCl_3$)

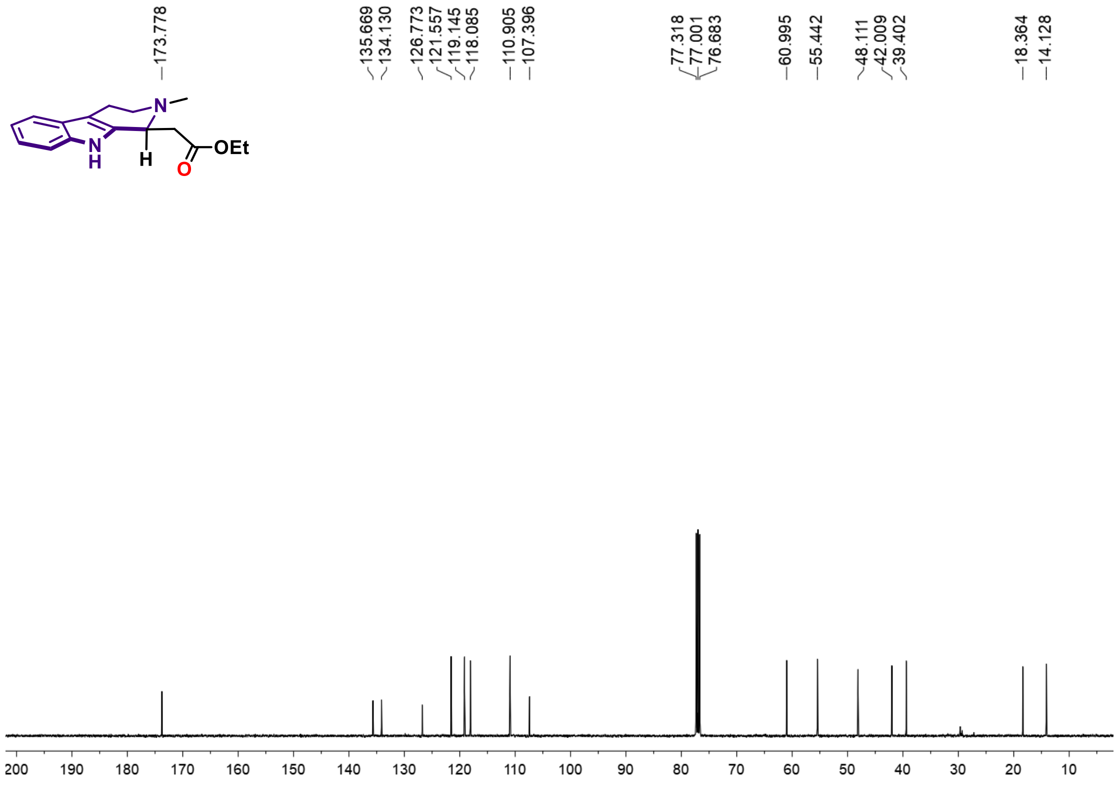

Supplementary Figure 204: HPLC spectrum of 8a

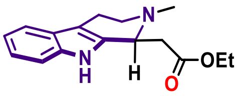

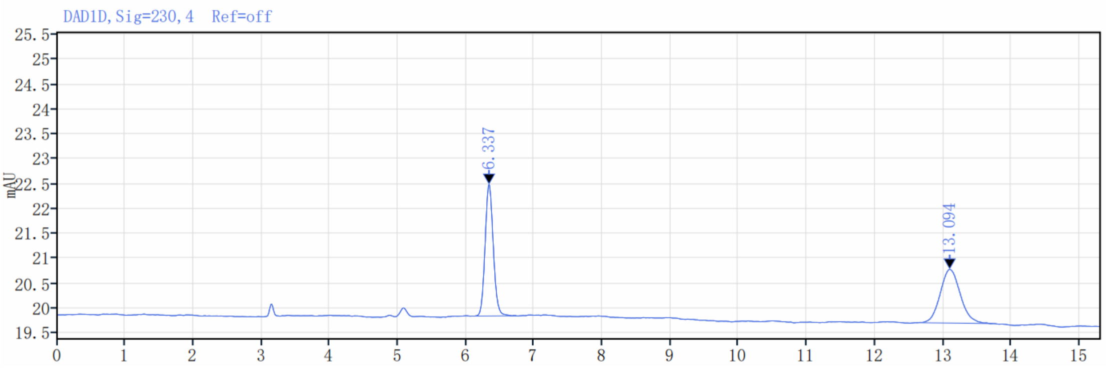

Signal : DAD1D, Sig=230, 4 Ref=off

RetTime [min]	Type	Width [min]	Area [mAU*s]	Height [mAU]	Area%
6.337	MM m	0.13	22.03	2.64	49.89
13.094	MM m	0.30	22.13	1.08	50.11
	Totals	0.43	44.16		

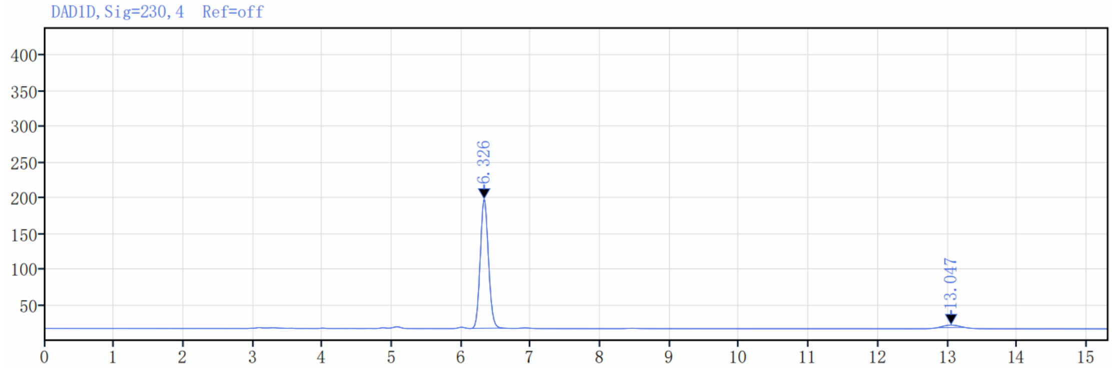

Signal : DAD1D, Sig=230, 4 Ref=off

RetTime [min]	Type	Width [min]	Area [mAU*s]	Height [mAU]	Area%
6.326	MM m	0.13	1475.30	180.27	96.92
13.047	MM m	0.23	46.83	3.41	3.08
	Totals	0.36	1522.13		

Supplementary Figure 205: ^{1}H NMR of 8b (400 MHz, CDCl$_3$)

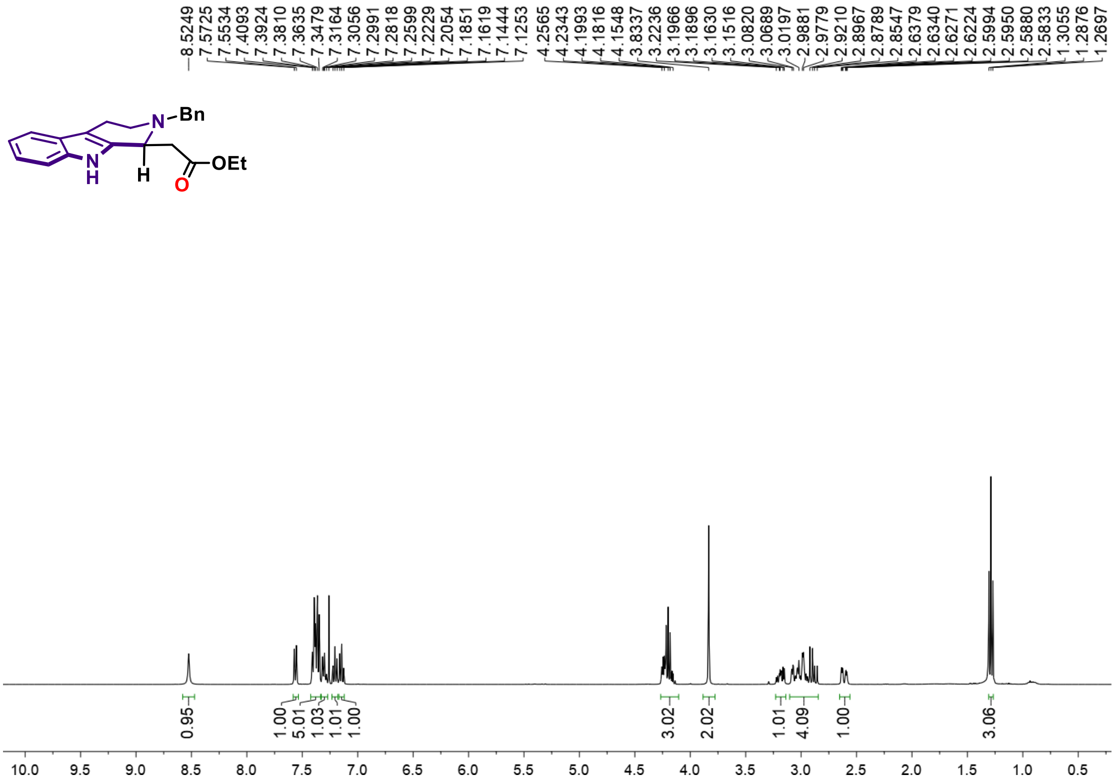

Supplementary Figure 206: ^{13}C NMR of 8b (101 MHz, CDCl$_3$)

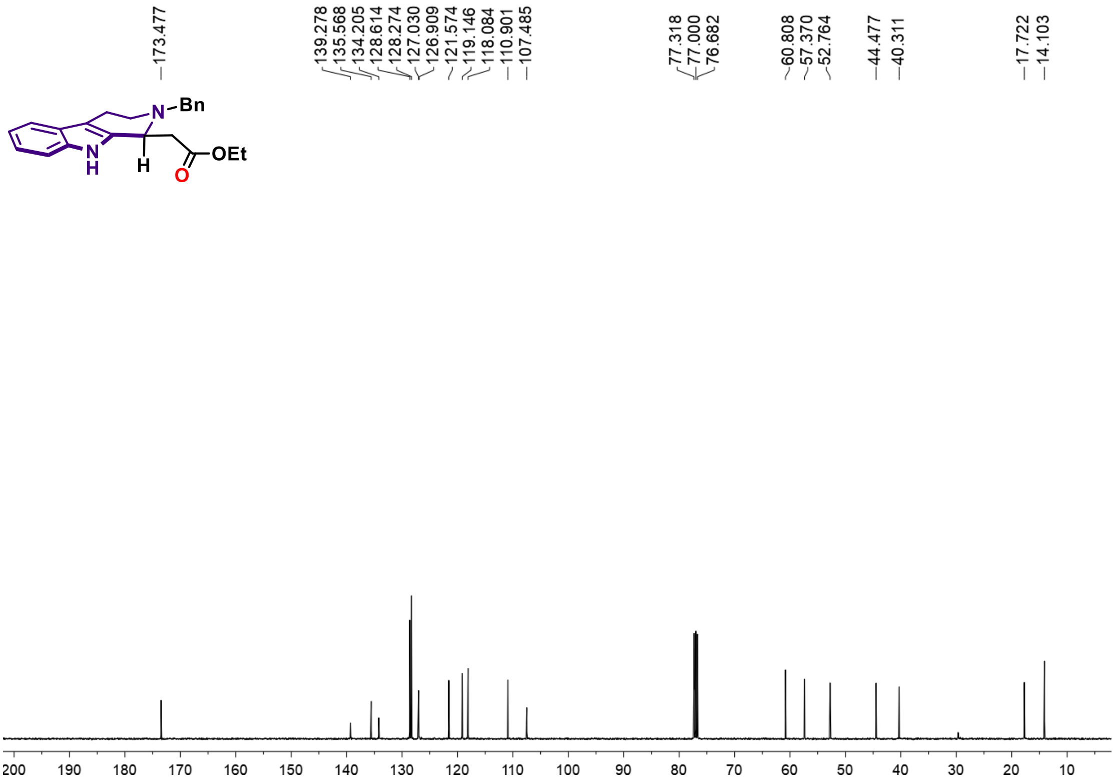

Supplementary Figure 207: HPLC spectrum of 8b

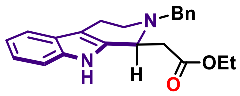

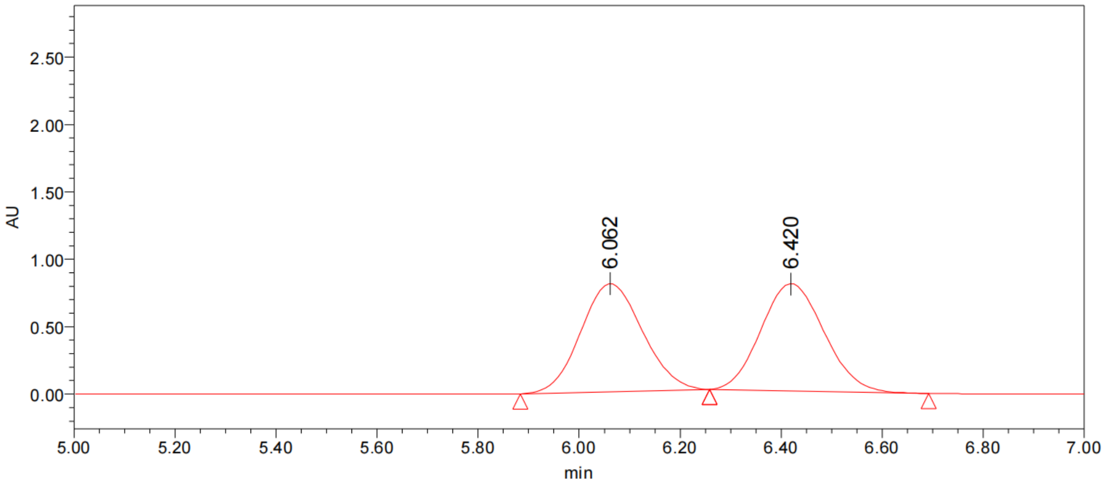

	RetTime [min]	Area [mAU*s]	Area%
1	6.062	6790705	49.62
2	6.420	6895286	50.38

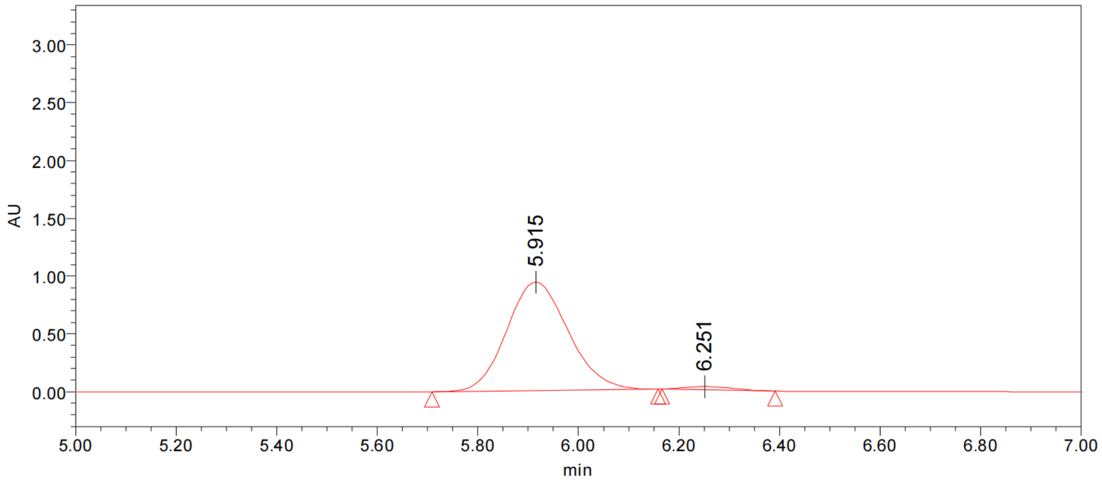

	RetTime [min]	Area [mAU*s]	Area%
1	5.915	7987114	97.69
2	6.251	188990	2.31

Supplementary Figure 208: ^{1}H NMR of 8c (400 MHz, $CDCl_3$)

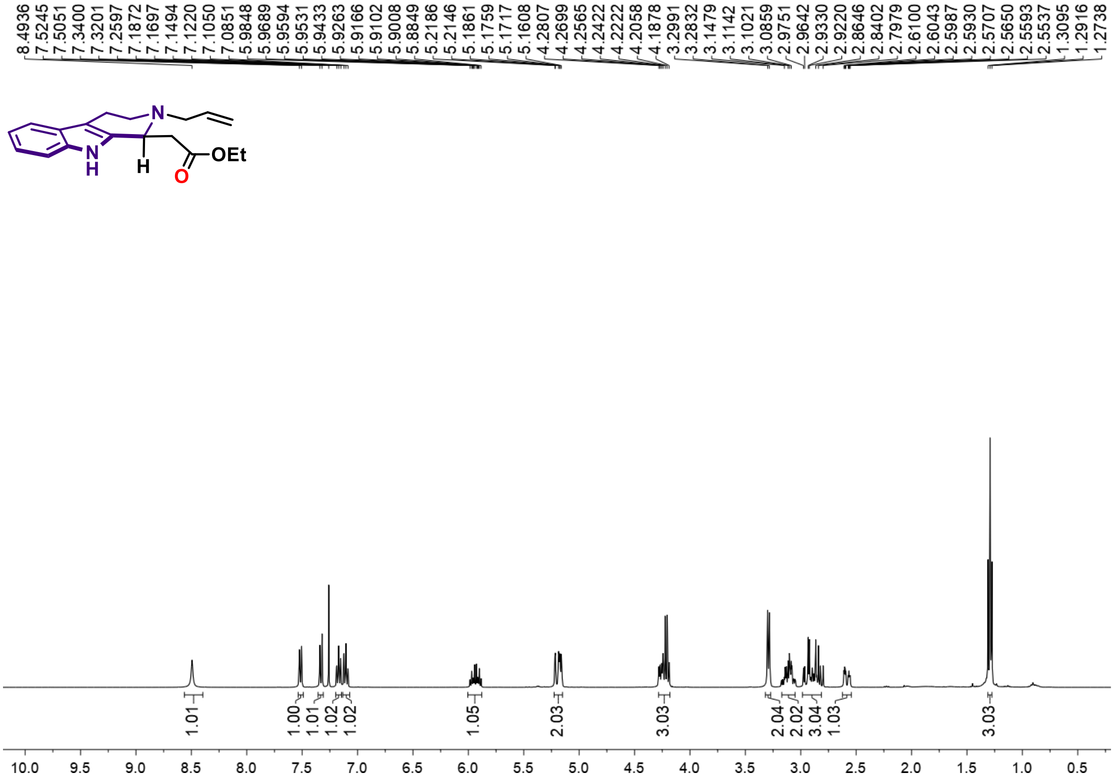

Supplementary Figure 209: ^{13}C NMR of 8c (101 MHz, $CDCl_3$)

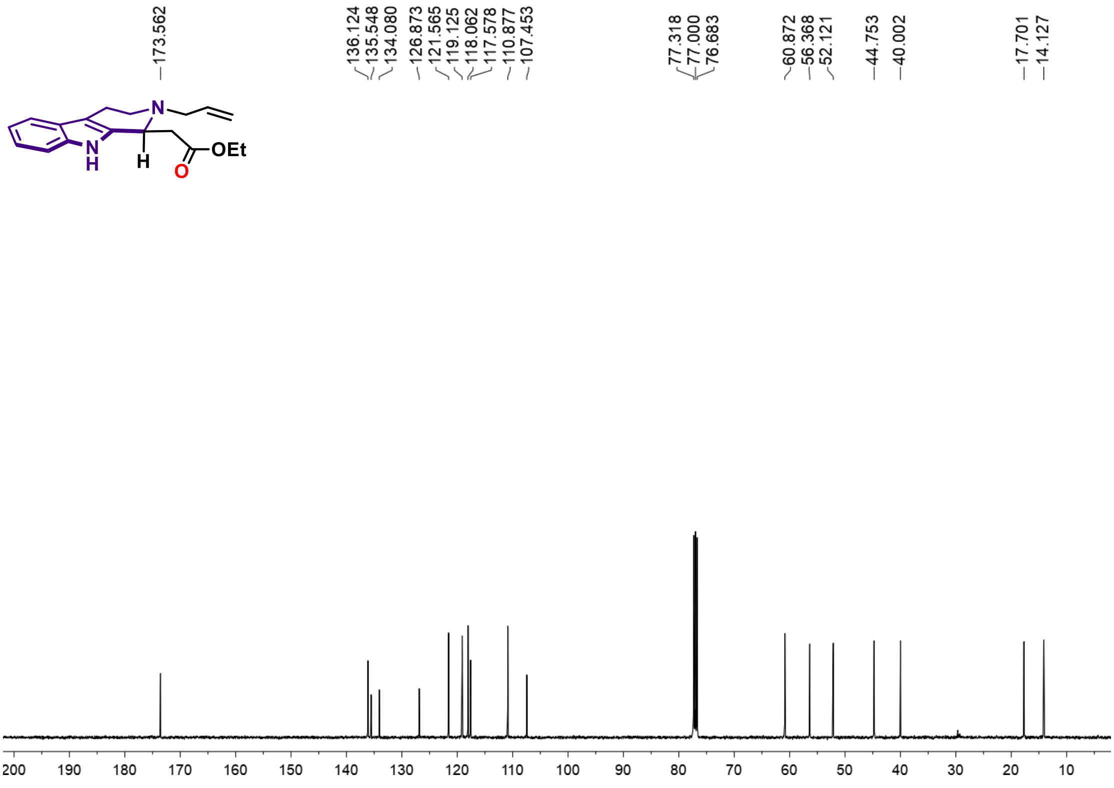

Supplementary Figure 210: HPLC spectrum of 8c

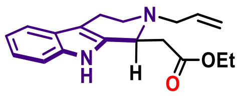

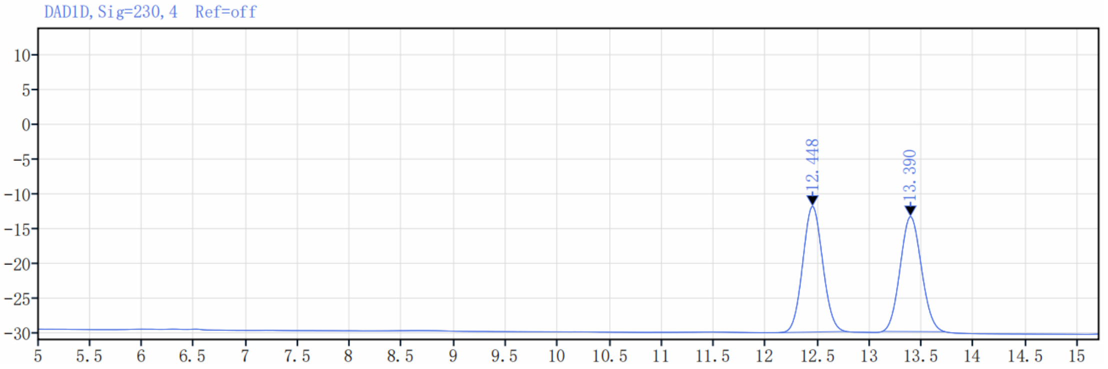

Signal : DAD1D, Sig=230, 4 Ref=off

RetTime [min]	Type	Width [min]	Area [mAU*s]	Height [mAU]	Area%
12. 448	MM m	0. 21	241. 37	18. 09	50. 63
13. 390	MM m	0. 22	235. 36	16. 54	49. 37
	Totals	0. 43	476. 73		

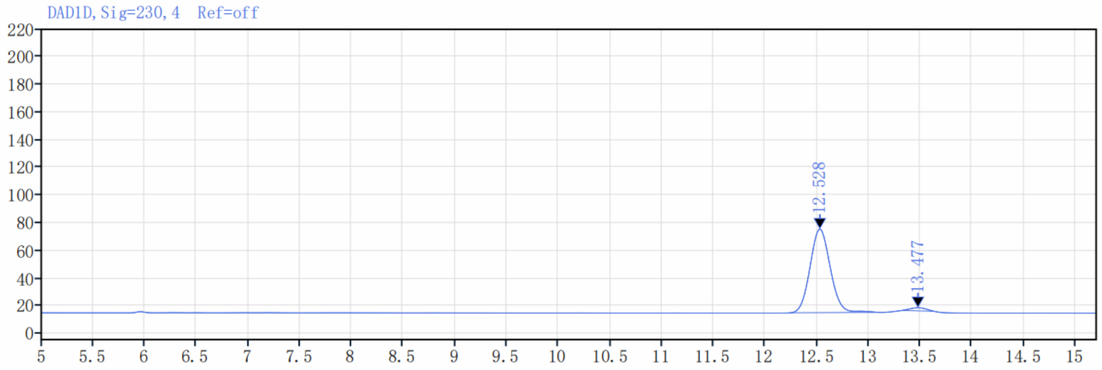

Signal : DAD1D, Sig=230, 4 Ref=off

RetTime [min]	Type	Width [min]	Area [mAU*s]	Height [mAU]	Area%
12. 528	MM m	0. 21	820. 07	60. 30	97. 21
13. 477	MM m	0. 18	23. 52	2. 21	2. 79
	Totals	0. 39	843. 59		

Supplementary Figure 211: ^{1}H NMR of 8d (400 MHz, $CDCl_3$)

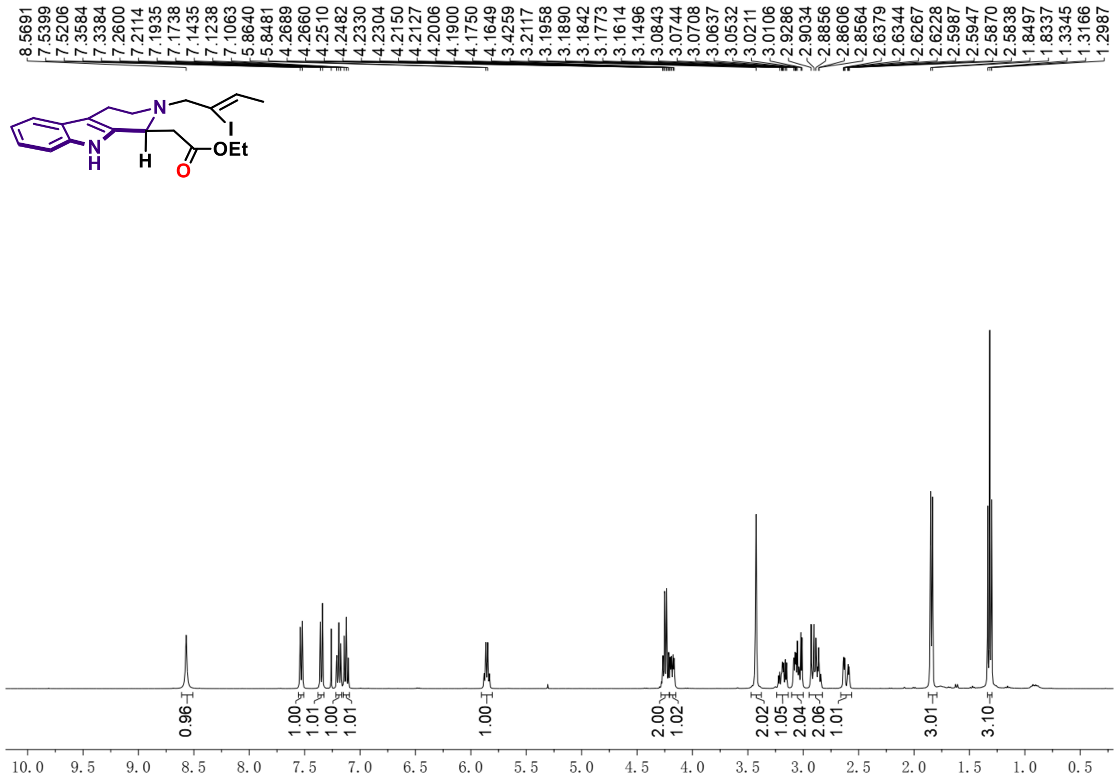

Supplementary Figure 212: ^{13}C NMR of 8d (101 MHz, $CDCl_3$)

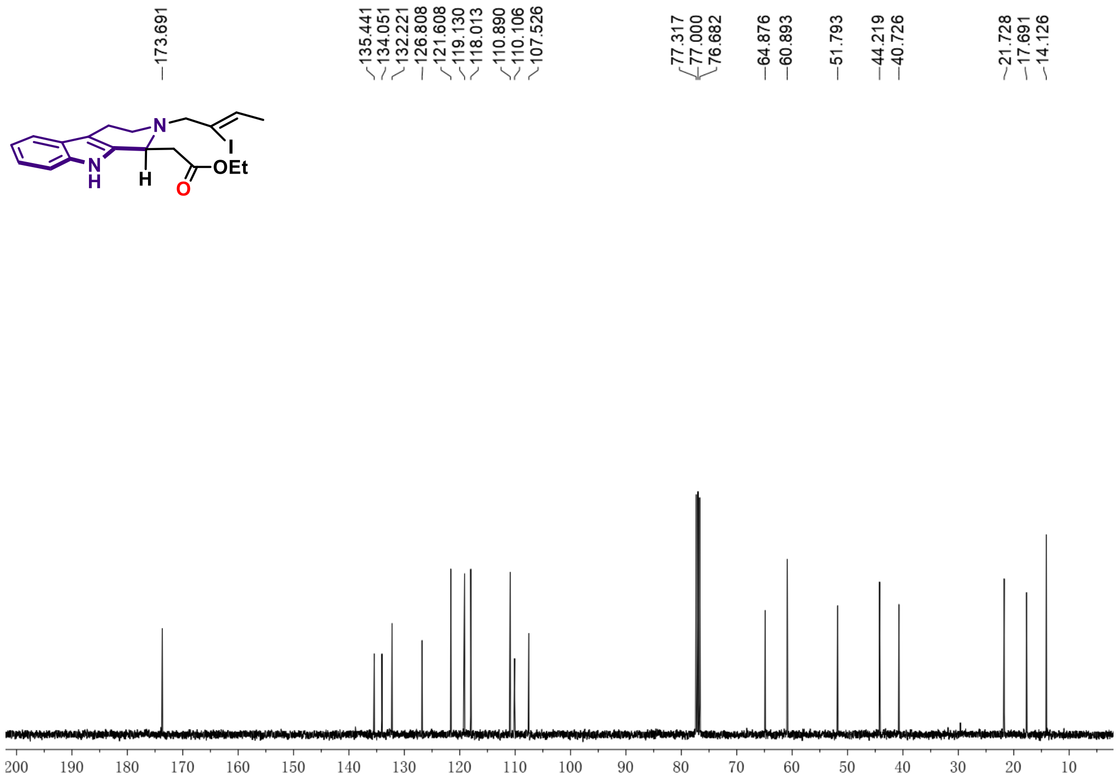

Supplementary Figure 213: HPLC spectrum of 8d

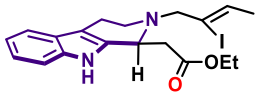

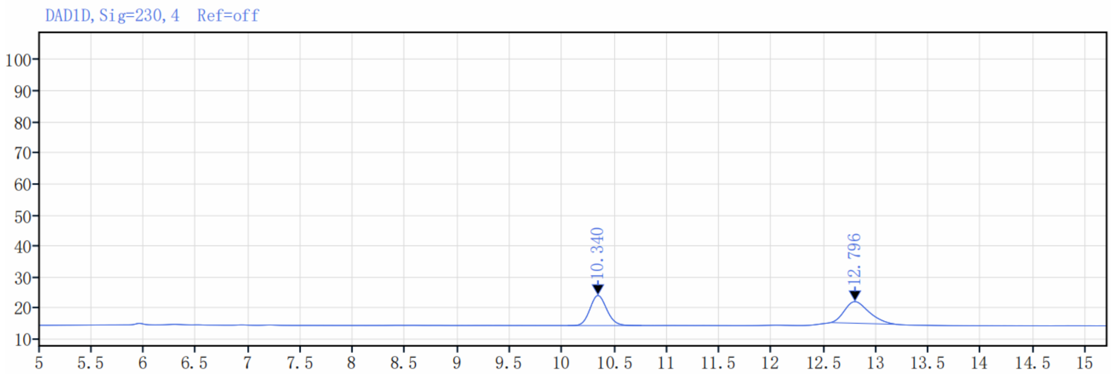

Signal : DAD1D, Sig=230, 4 Ref=off

RetTime [min]	Type	Width [min]	Area [mAU*s]	Height [mAU]	Area%
10. 340	MM m	0. 17	108. 21	9. 68	48. 76
12. 796	MM m	0. 25	113. 74	6. 96	51. 24
	Totals	0. 42	221. 95		

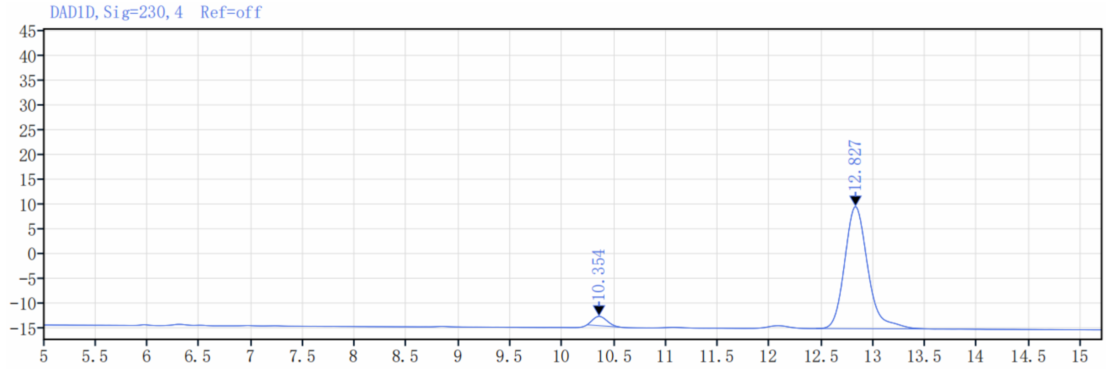

Signal : DAD1D, Sig=230, 4 Ref=off

RetTime [min]	Type	Width [min]	Area [mAU*s]	Height [mAU]	Area%
10. 354	MM m	0. 15	17. 85	1. 89	4. 45
12. 827	MM m	0. 24	383. 45	24. 66	95. 55
	Totals	0. 39	401. 31		

Supplementary Figure 214: ^{1}H NMR of 9 (400 MHz, $CDCl_3$)

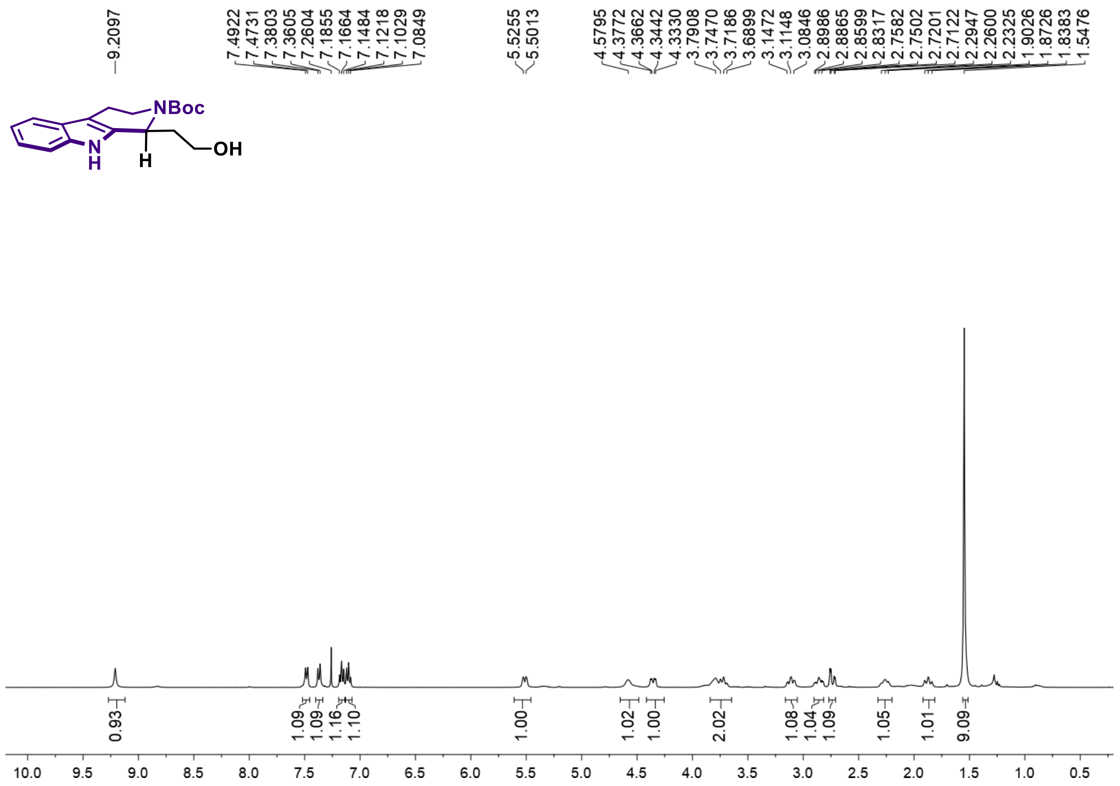

Supplementary Figure 215: ^{13}C NMR of 9 (101 MHz, $CDCl_3$)

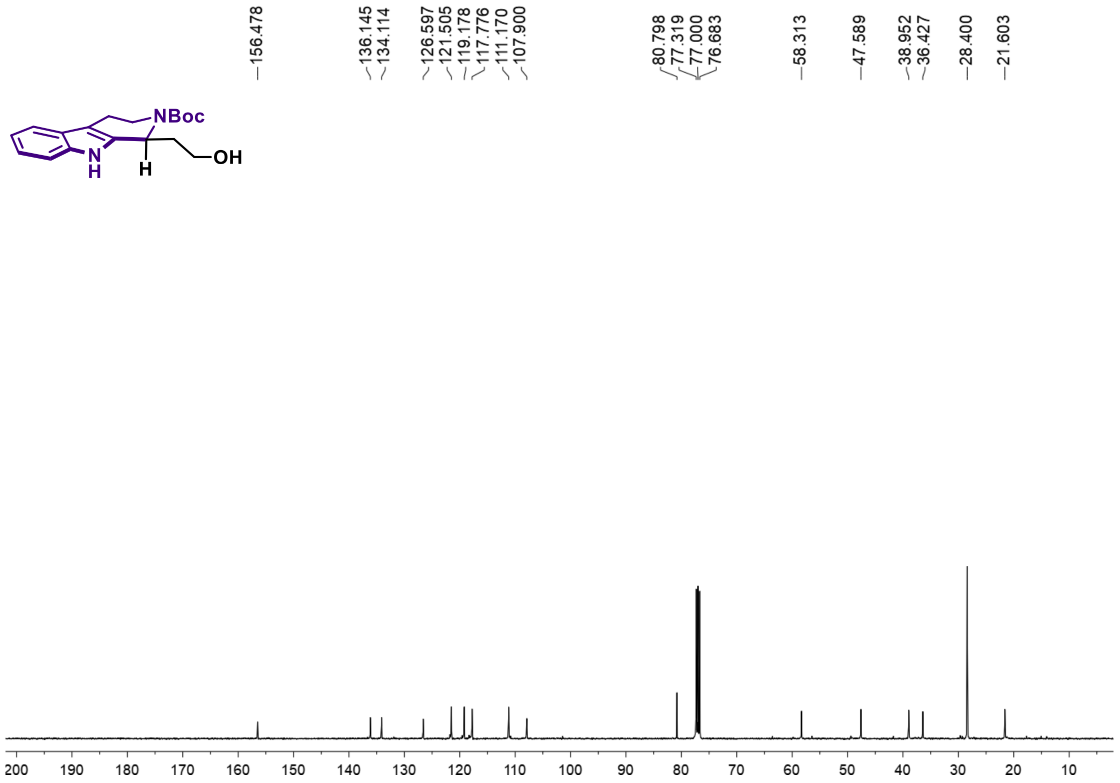

Supplementary Figure 216: HPLC spectrum of 9

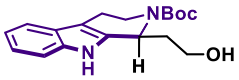

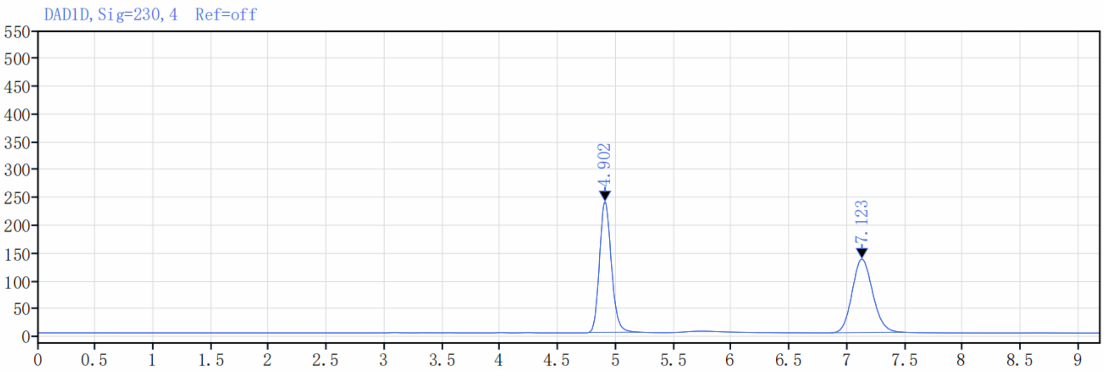

Signal : DAD1D, Sig=230, 4 Ref=off

RetTime [min]	Type	Width [min]	Area [mAU*s]	Height [mAU]	Area%
4.902	MM m	0.11	1670.27	234.56	50.66
7.123	MM m	0.19	1627.02	132.00	49.34
	Totals	0.30	3297.28		

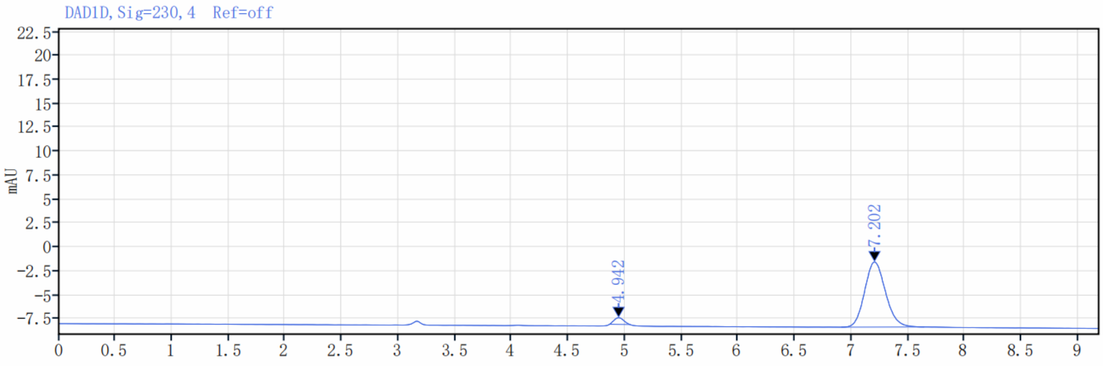

Signal : DAD1D, Sig=230, 4 Ref=off

RetTime [min]	Type	Width [min]	Area [mAU*s]	Height [mAU]	Area%
4.942	MM m	0.10	4.11	0.70	4.51
7.202	MM m	0.20	87.02	6.80	95.49
	Totals	0.29	91.12		

Supplementary Figure 217: ^{1}H NMR of 10 (400 MHz, $CDCl_3$)

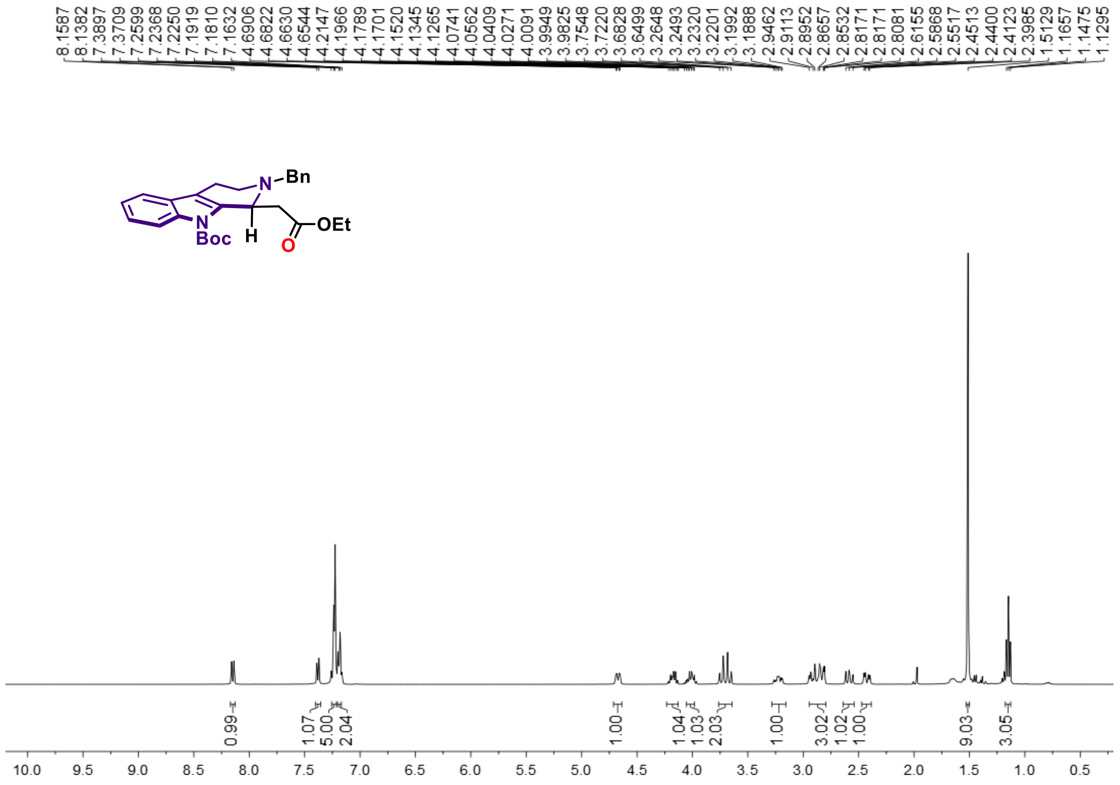

Supplementary Figure 218: ^{13}C NMR of 10 (101 MHz, $CDCl_3$)

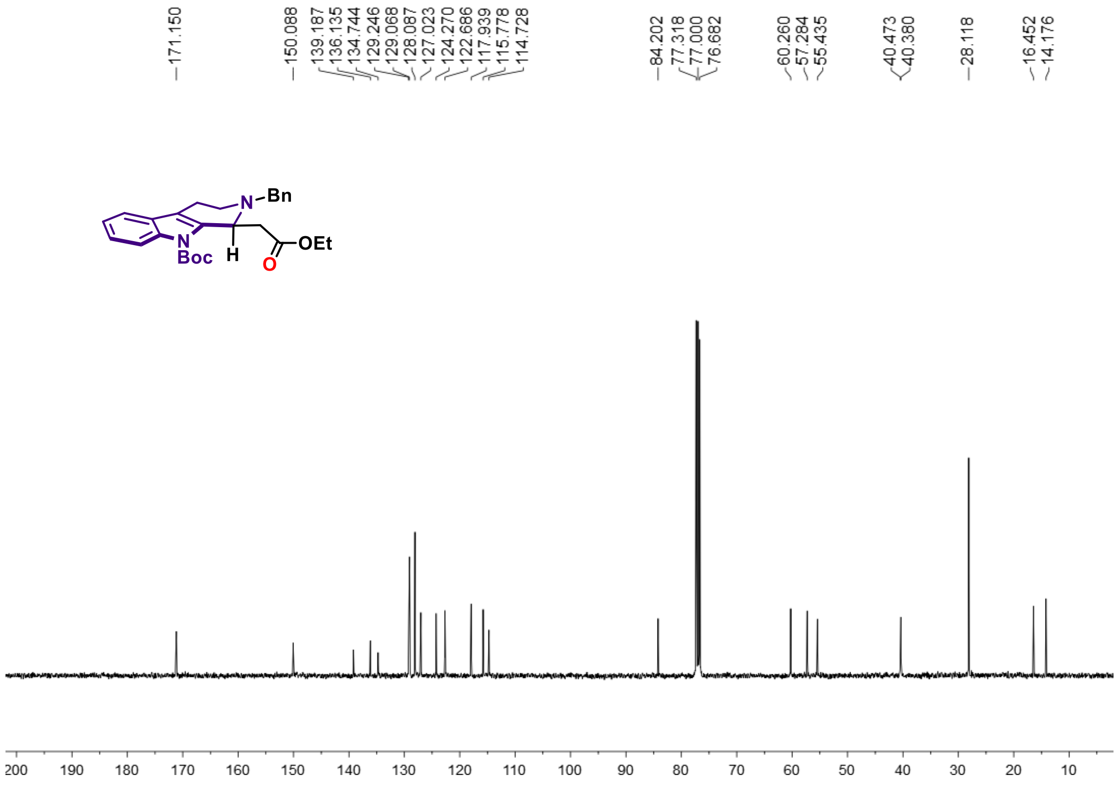

Supplementary Figure 219: ^{1}H NMR of 11 (400 MHz, $CDCl_3$)

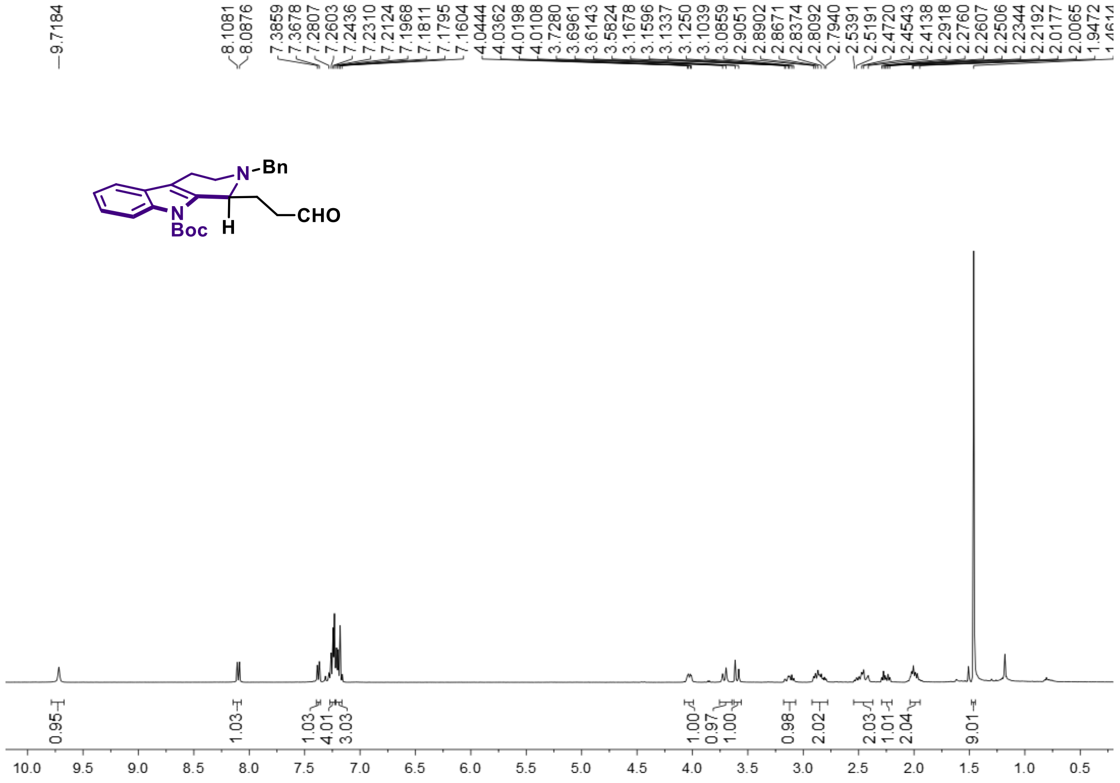

Supplementary Figure 220: ^{13}C NMR of 11 (101 MHz, $CDCl_3$)

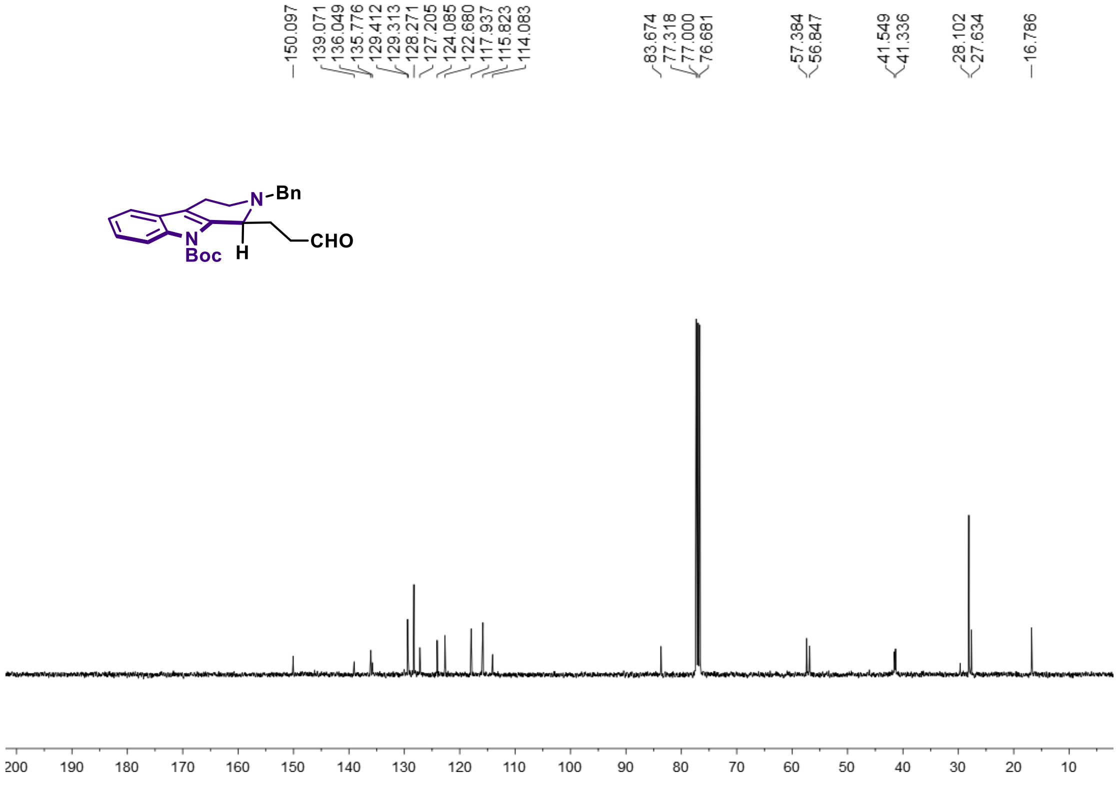

Supplementary Figure 221: ^{1}H NMR of 12 (400 MHz, $CDCl_3$)

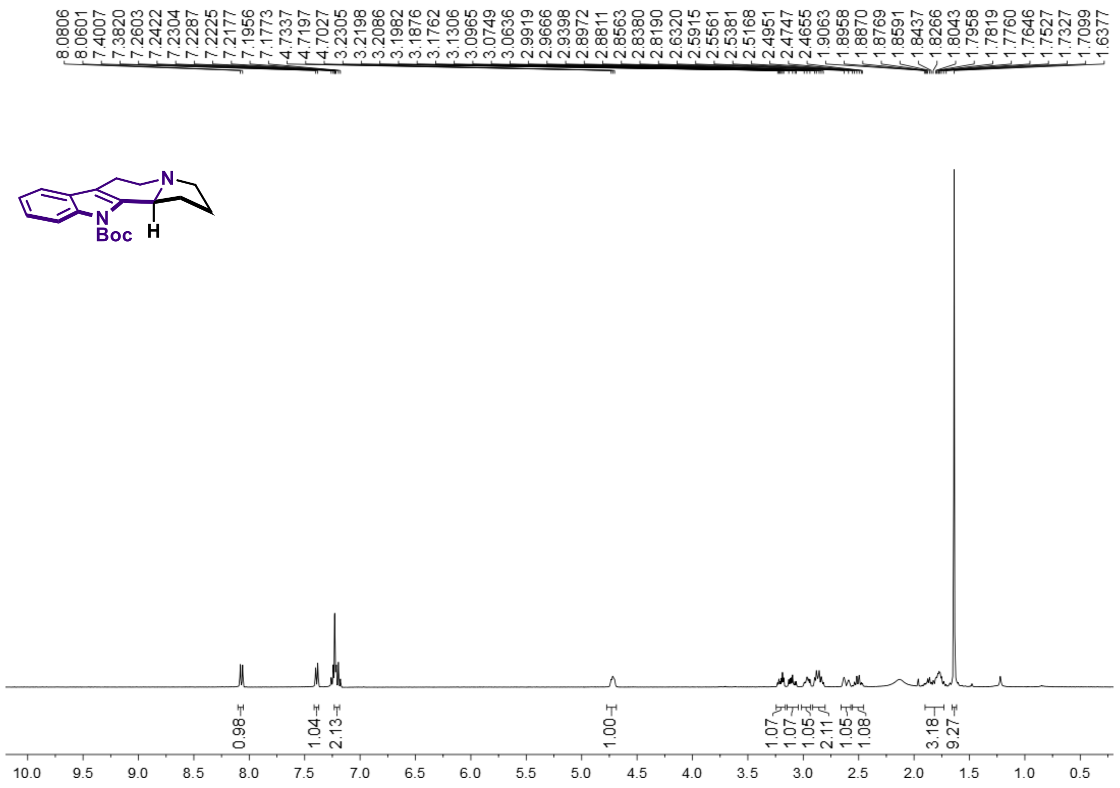

Supplementary Figure 222: ^{13}C NMR of 12 (101 MHz, $CDCl_3$)

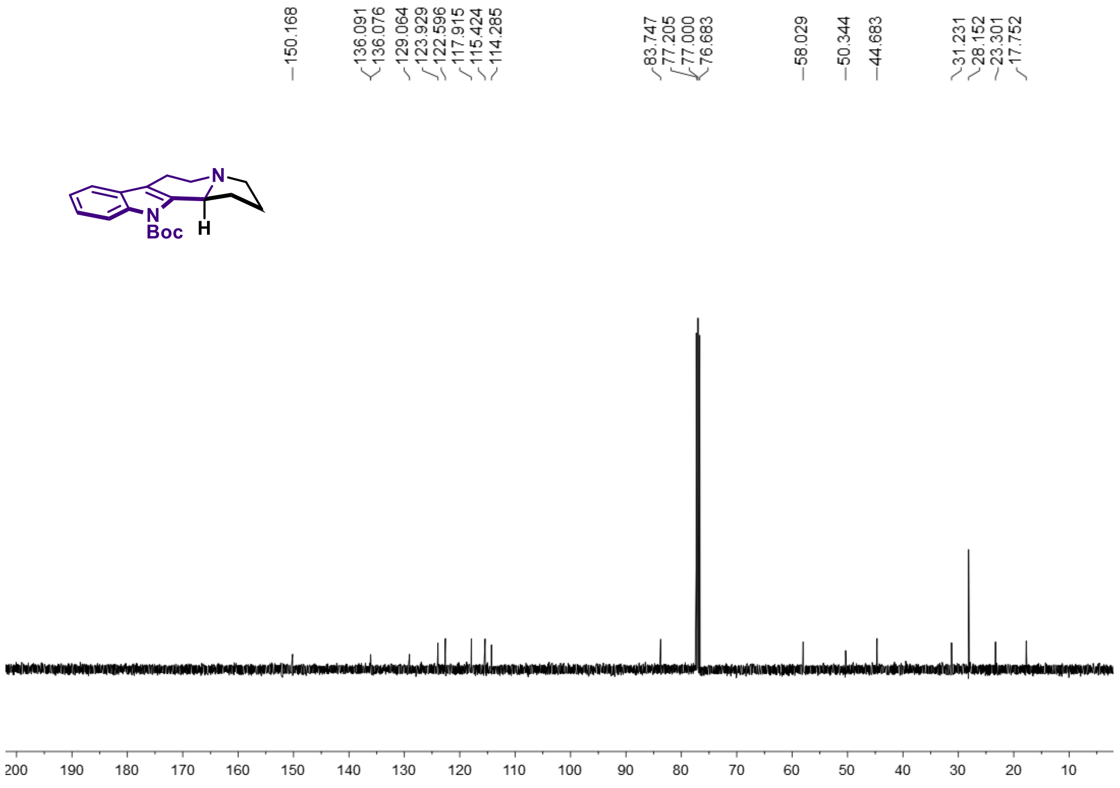

Supplementary Figure 223: ^{1}H NMR of 13 (400 MHz, CDCl$_3$)

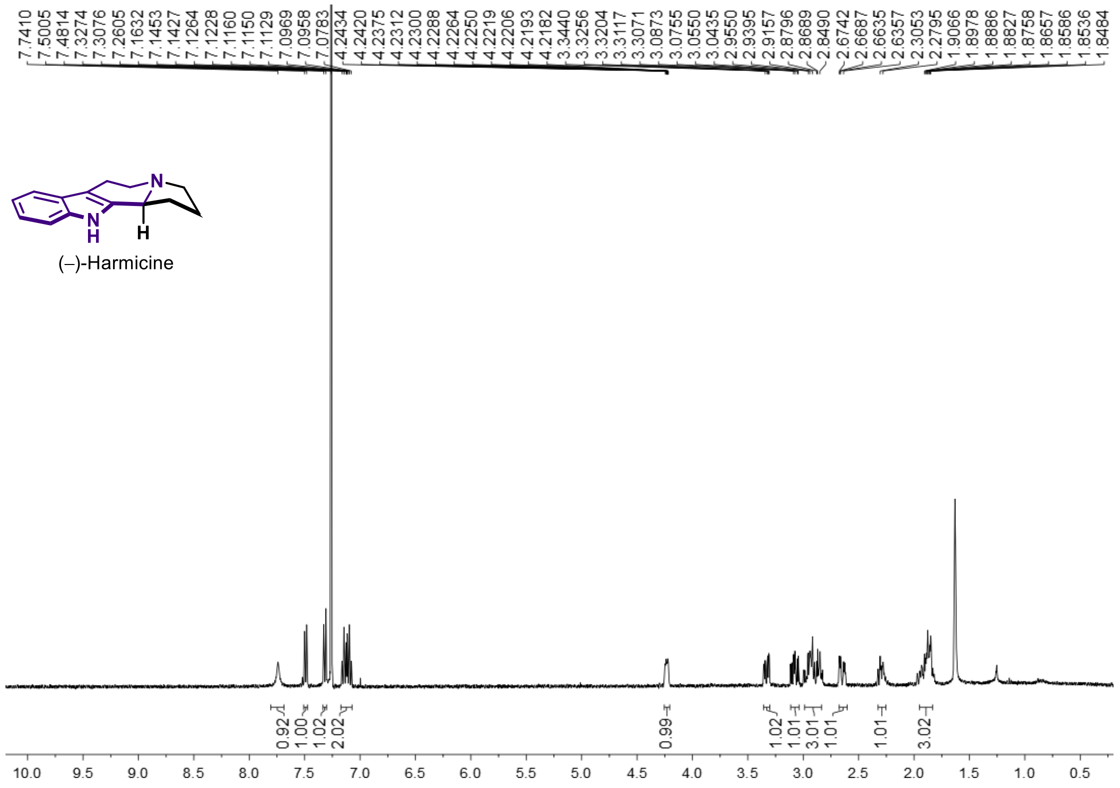

Supplementary Figure 224: ^{13}C NMR of 13 (101 MHz, CDCl$_3$)

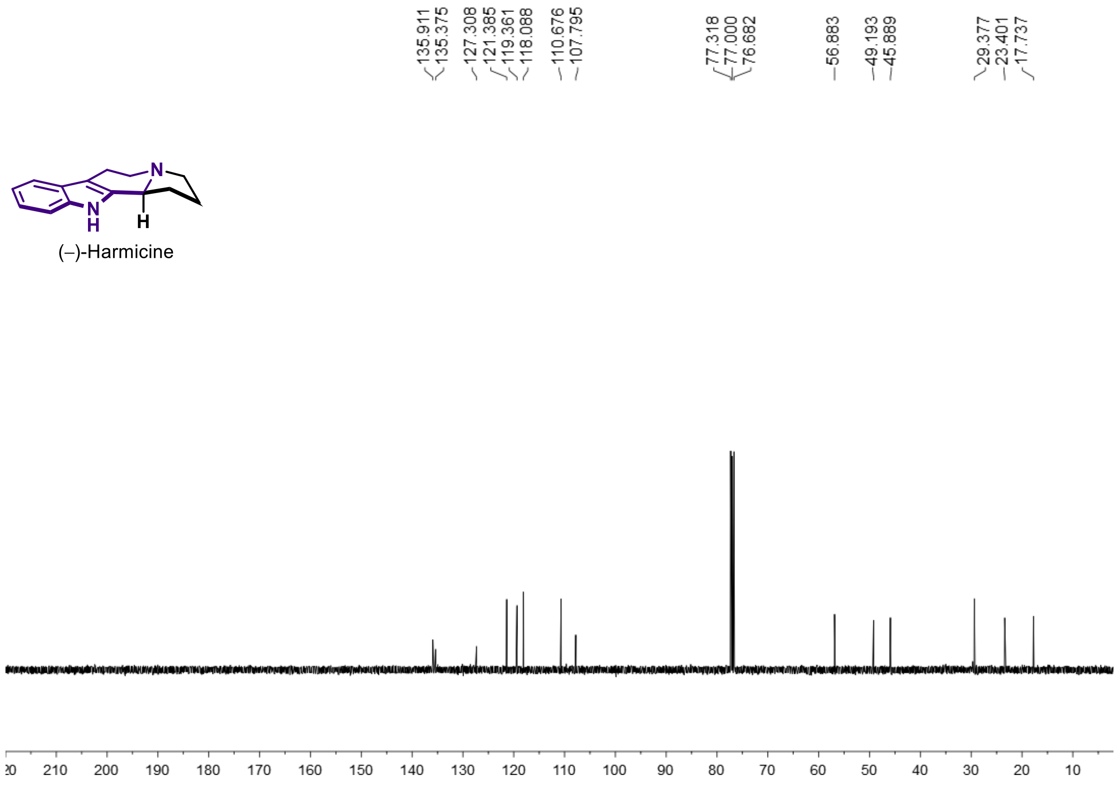

Supplementary Figure 225: ^{1}H NMR of 14 (400 MHz, $CDCl_3$)

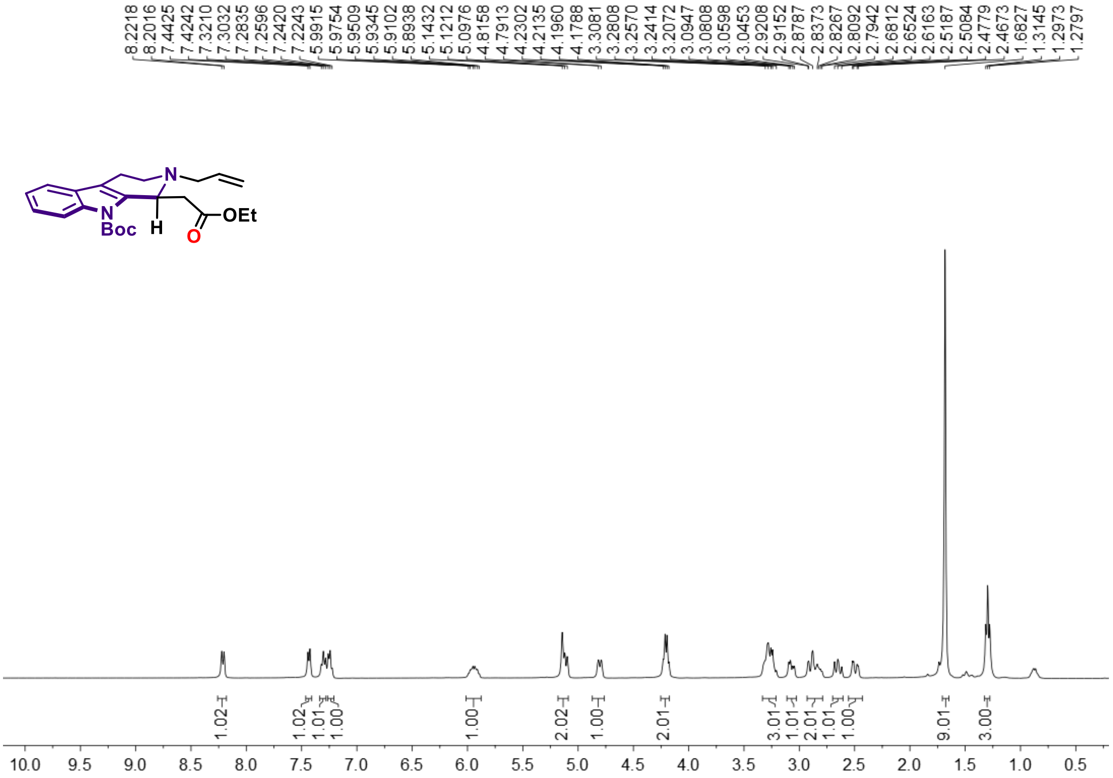

Supplementary Figure 226: ^{13}C NMR of 14 (101 MHz, $CDCl_3$)

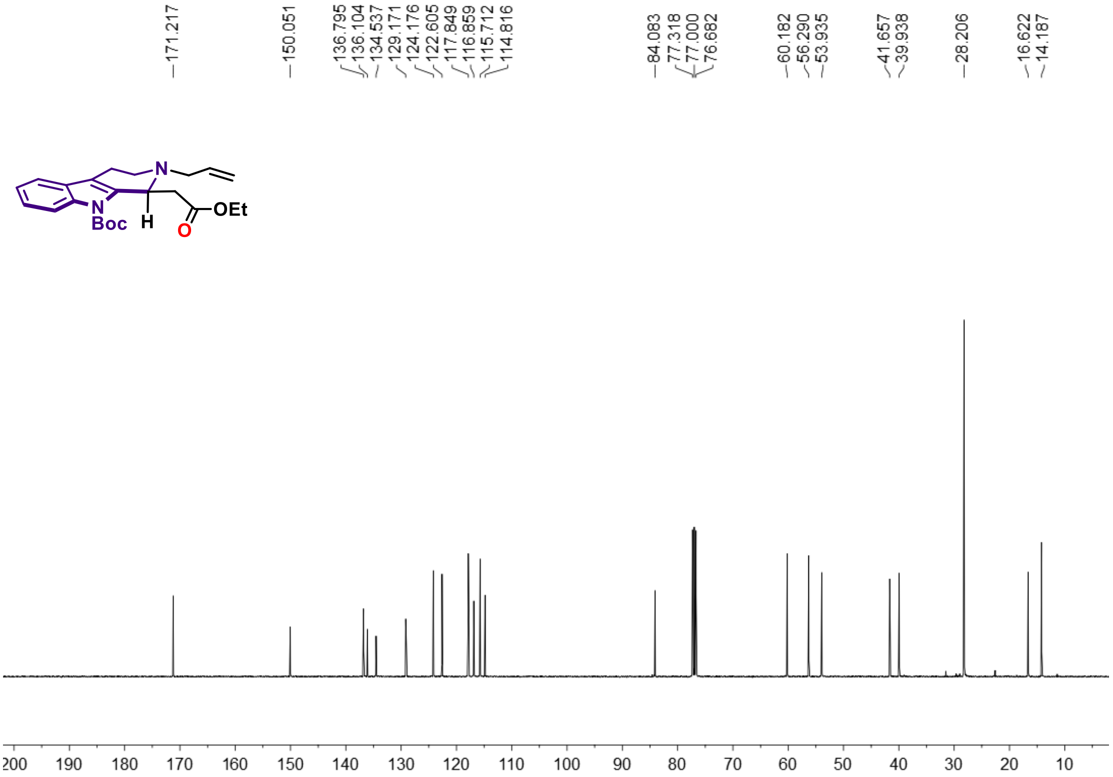

Supplementary Figure 227: ^{1}H NMR of 15 (400 MHz, $CDCl_3$)

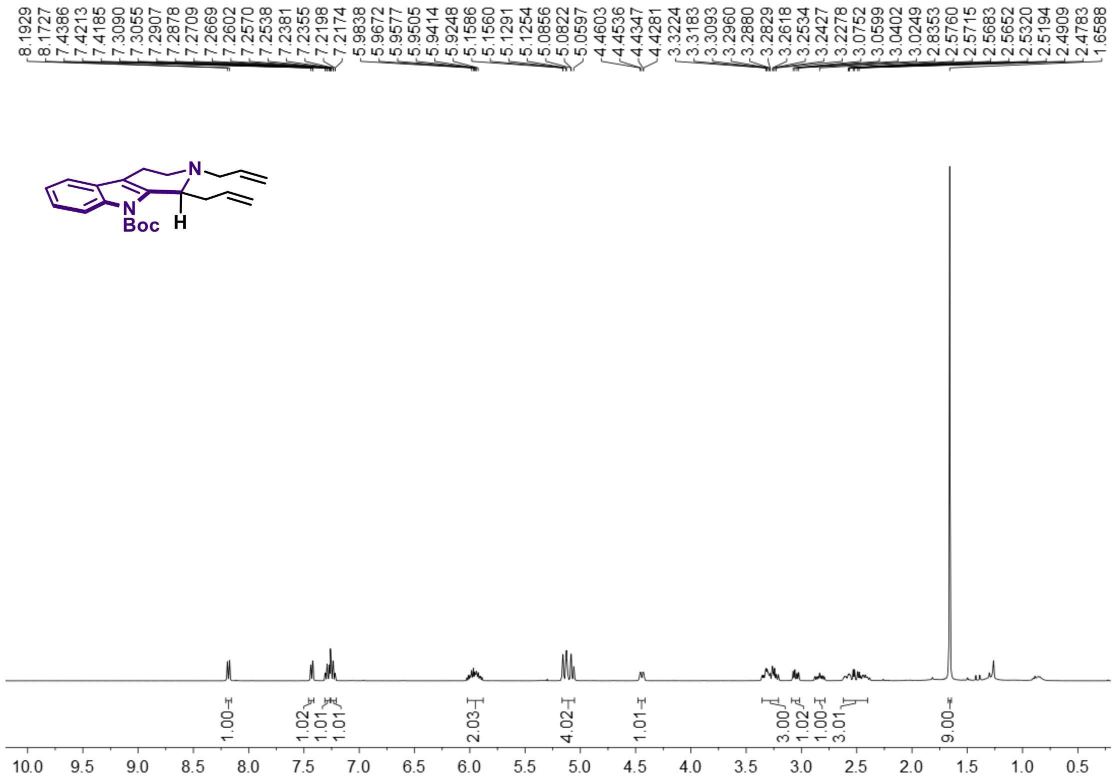

Supplementary Figure 228: ^{13}C NMR of 15 (101 MHz, $CDCl_3$)

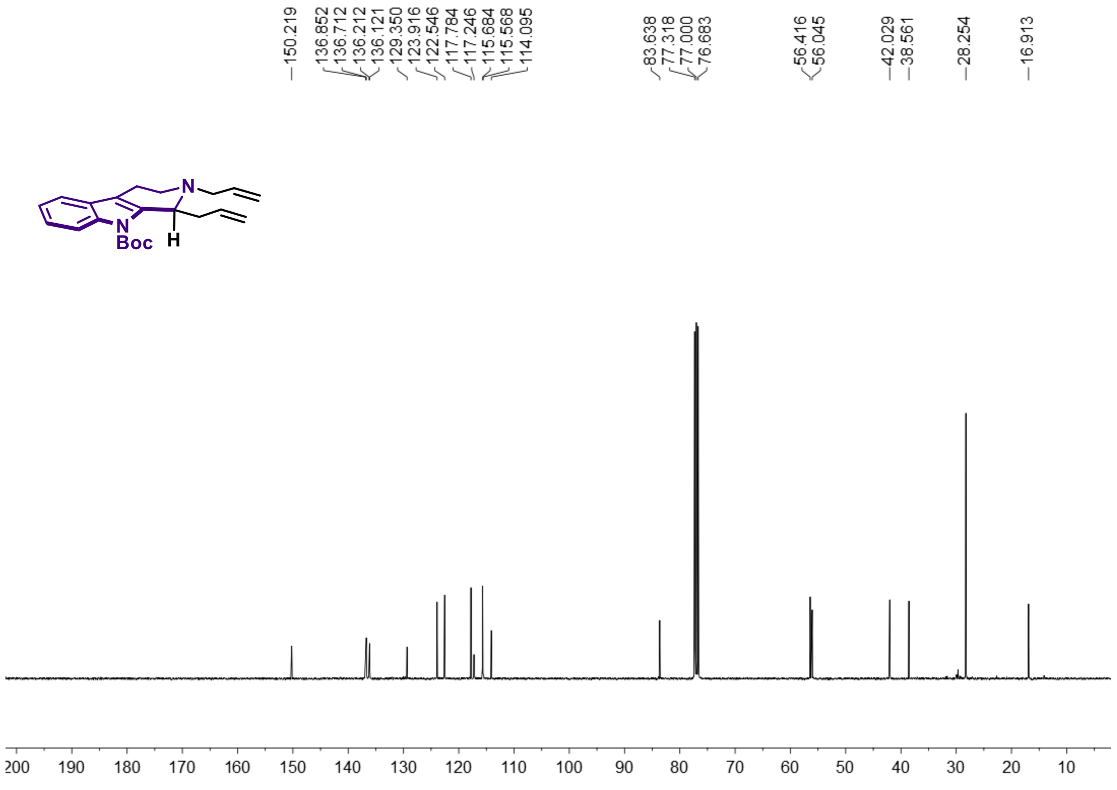

Supplementary Figure 229: ^{1}H NMR of 16 (400 MHz, $CDCl_3$)

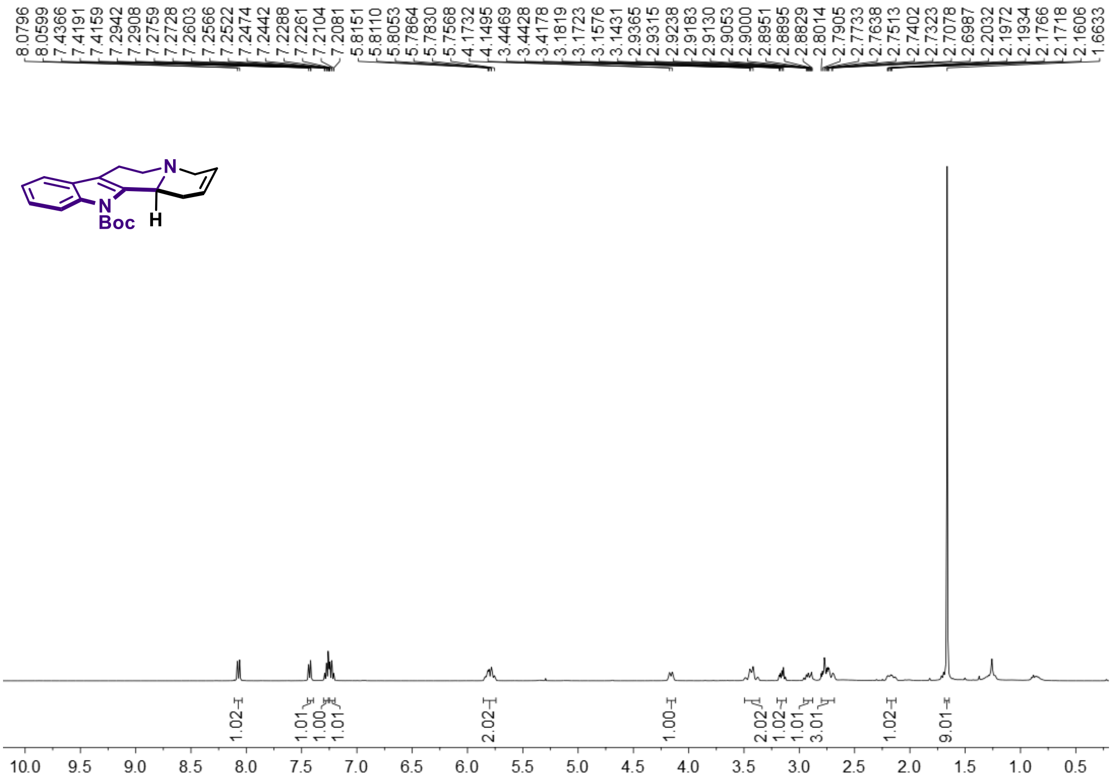

Supplementary Figure 230: ^{13}C NMR of 16 (101 MHz, $CDCl_3$)

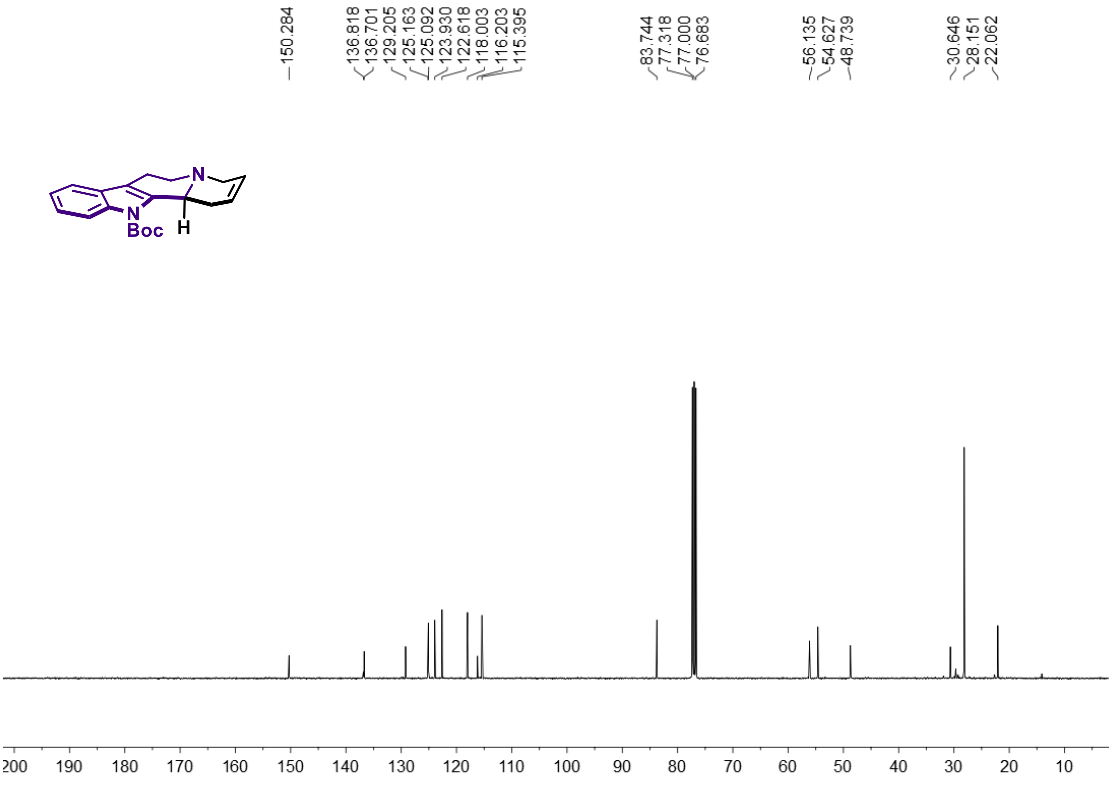

Supplementary Figure 231: ^{1}H NMR of 17 (400 MHz, $CDCl_3$)

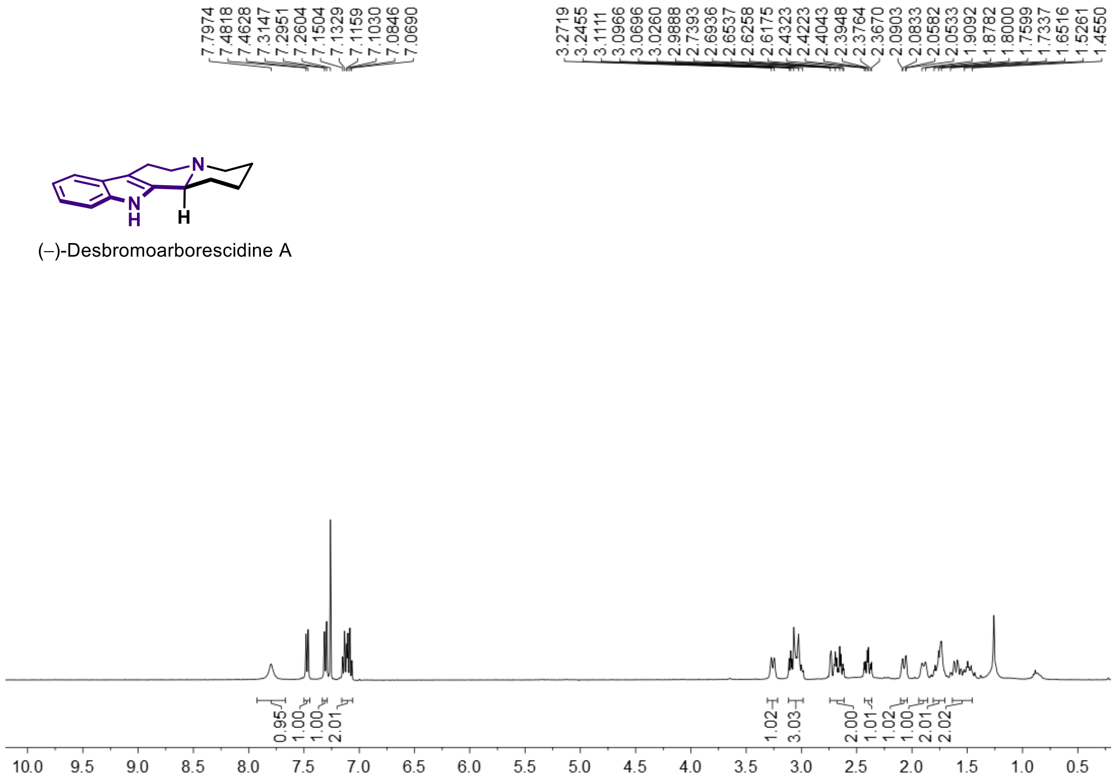

Supplementary Figure 232: ^{13}C NMR of 17 (101 MHz, $CDCl_3$)

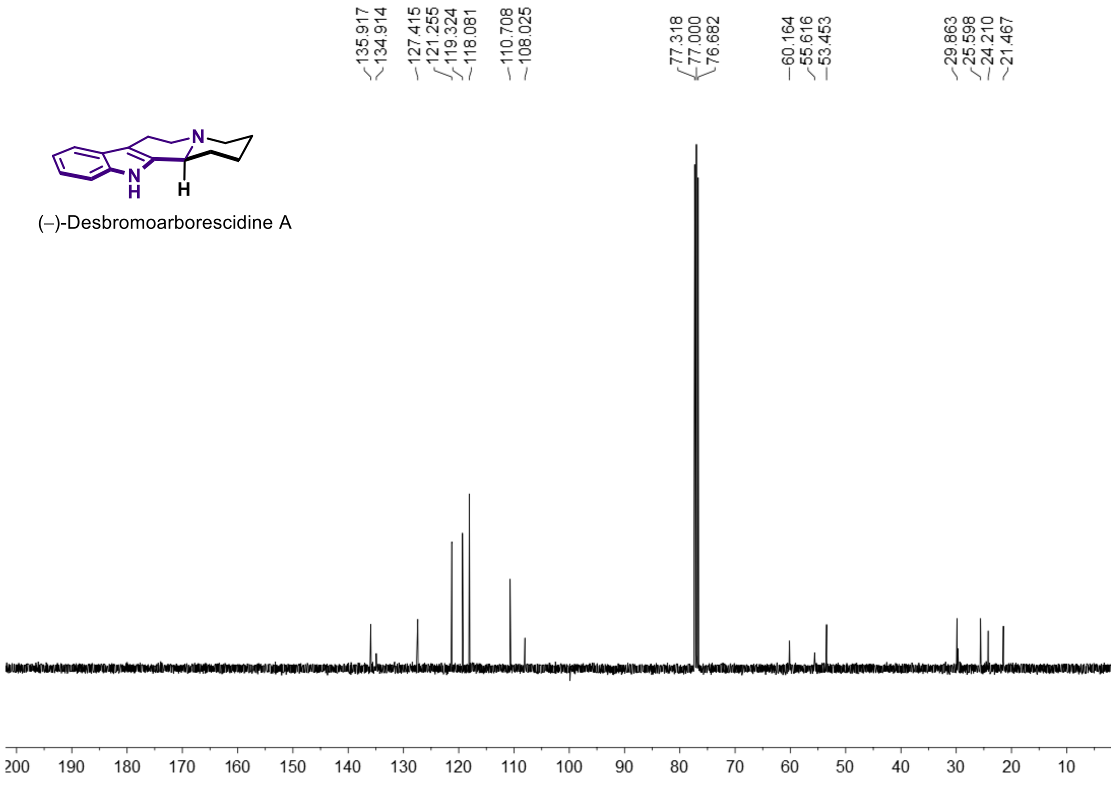

Supplementary Figure 233: ^{1}H NMR of 18 (400 MHz, CDCl$_3$)

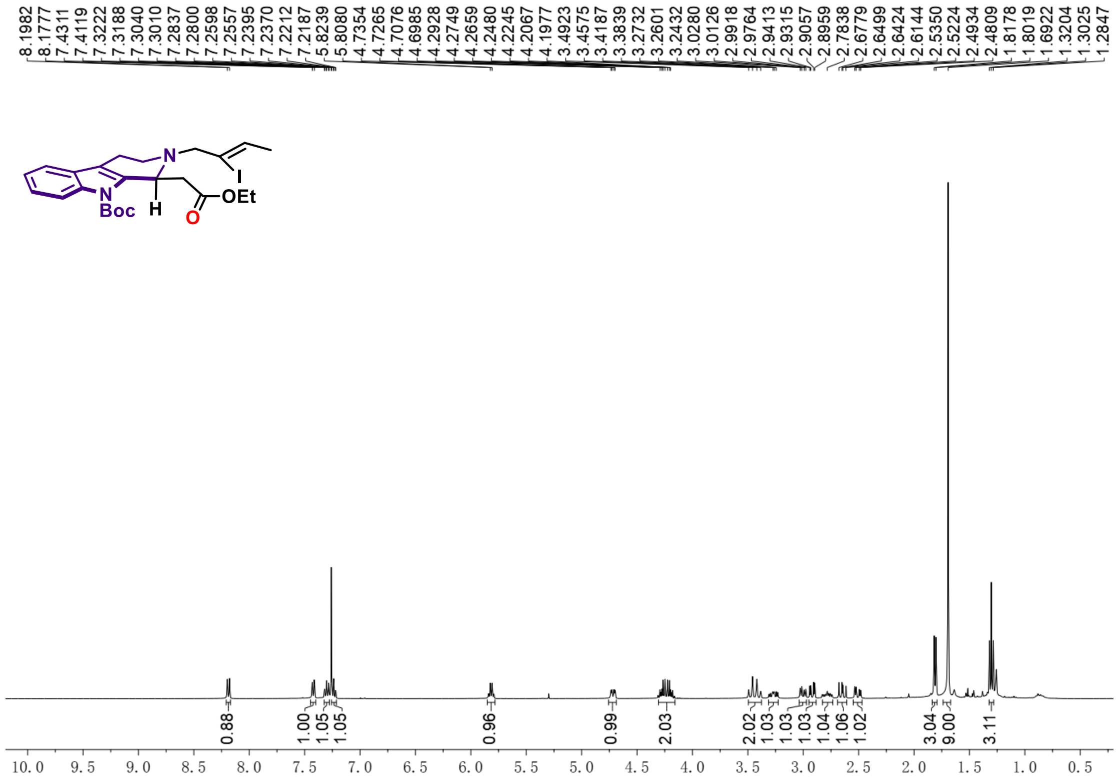

Supplementary Figure 234: ^{13}C NMR of 18 (101 MHz, CDCl$_3$)

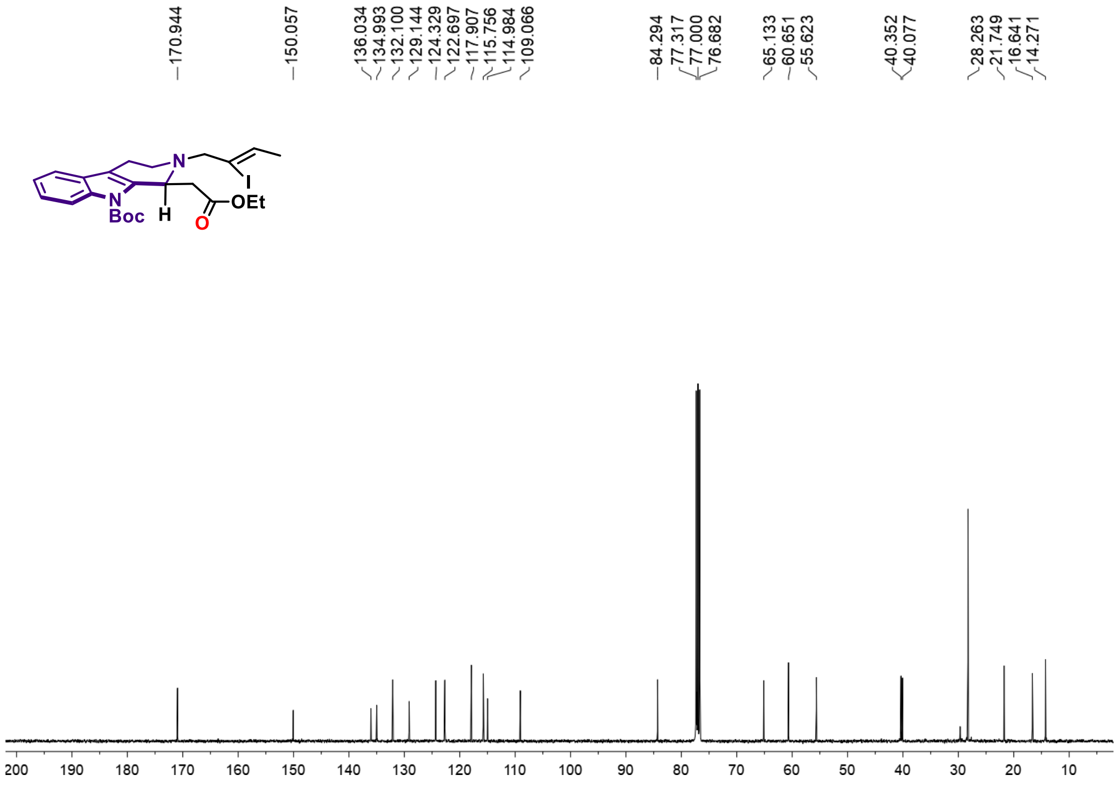

Supplementary Figure 235: ^{1}H NMR of 19 (400 MHz, $CDCl_3$)

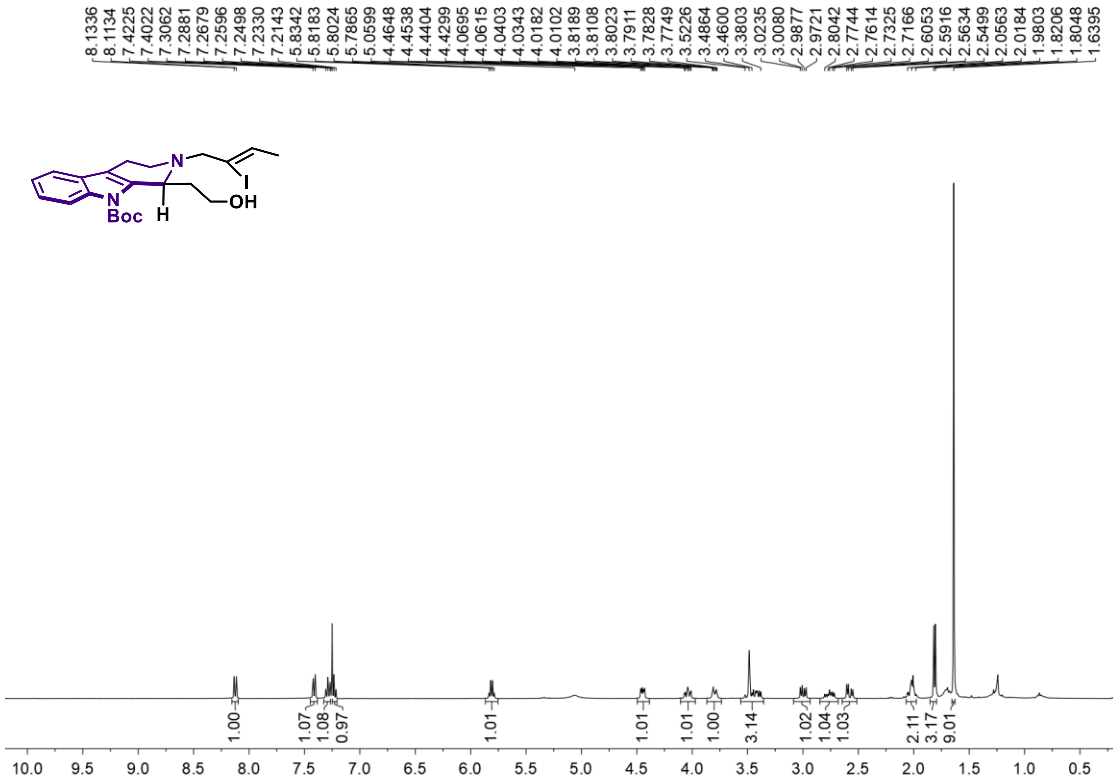

Supplementary Figure 236: ^{13}C NMR of 19 (101 MHz, $CDCl_3$)

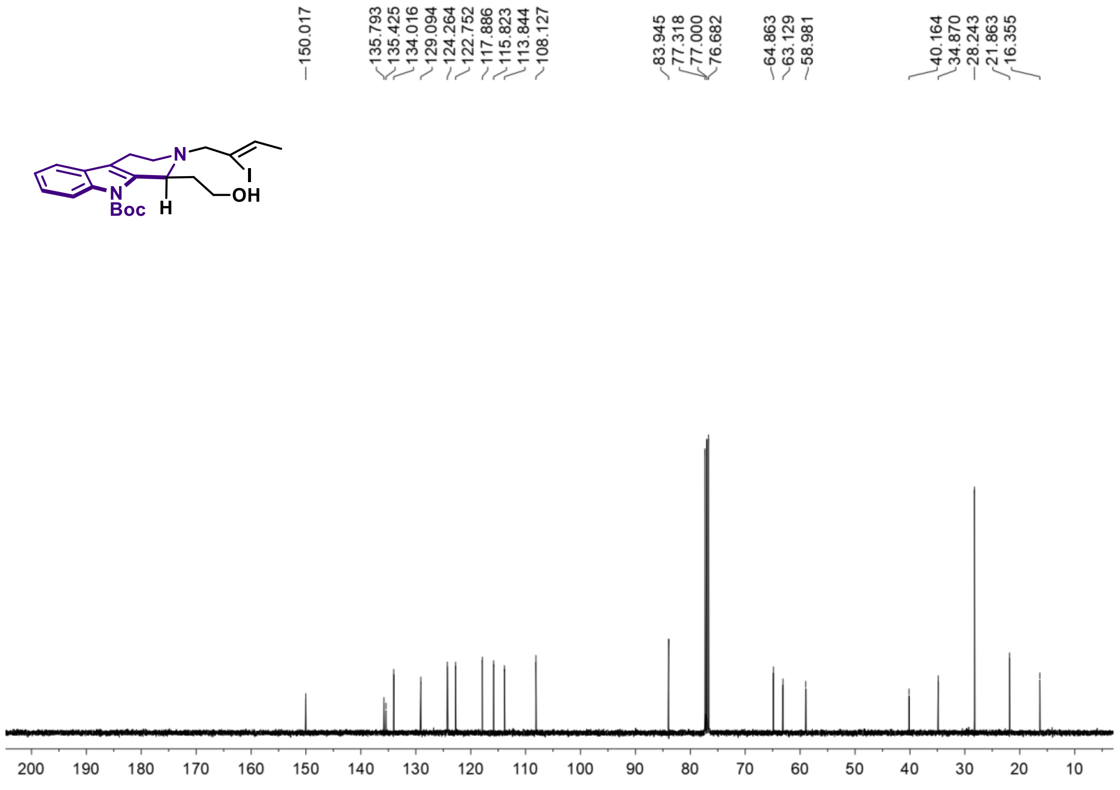

Supplementary Figure 237: ^{1}H NMR of 20 (400 MHz, $CDCl_3$)

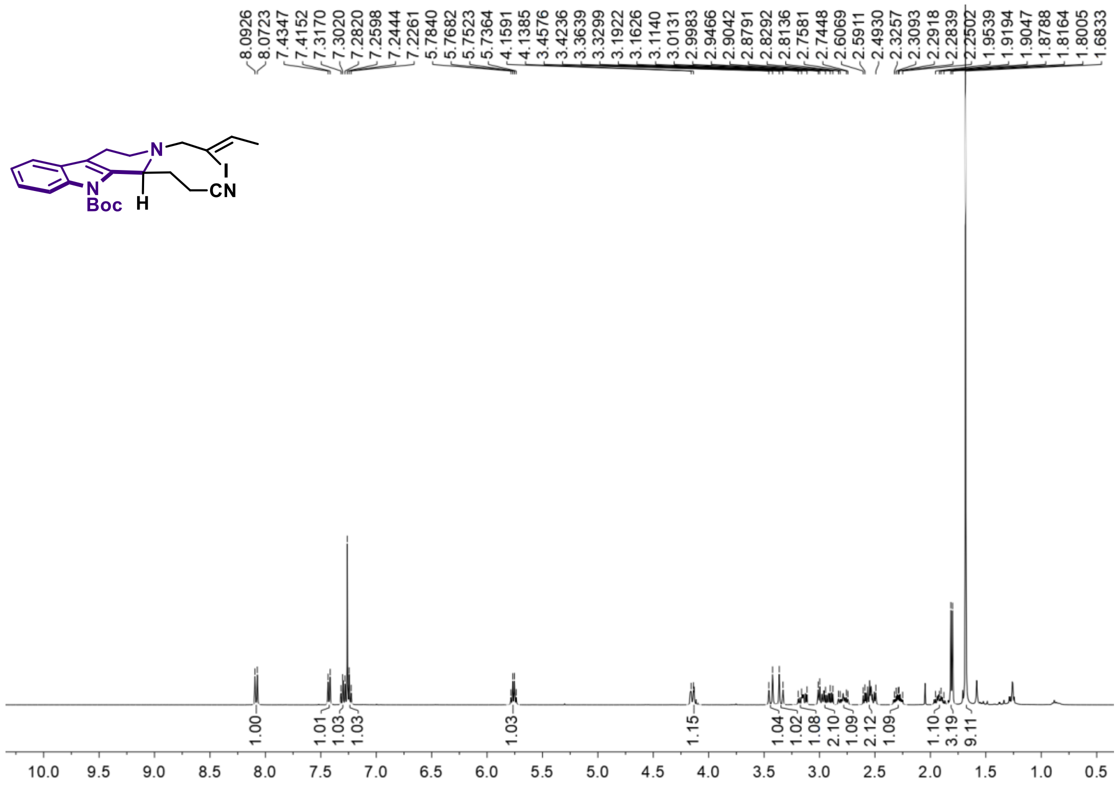

Supplementary Figure 238: ^{13}C NMR of 20 (101 MHz, $CDCl_3$)

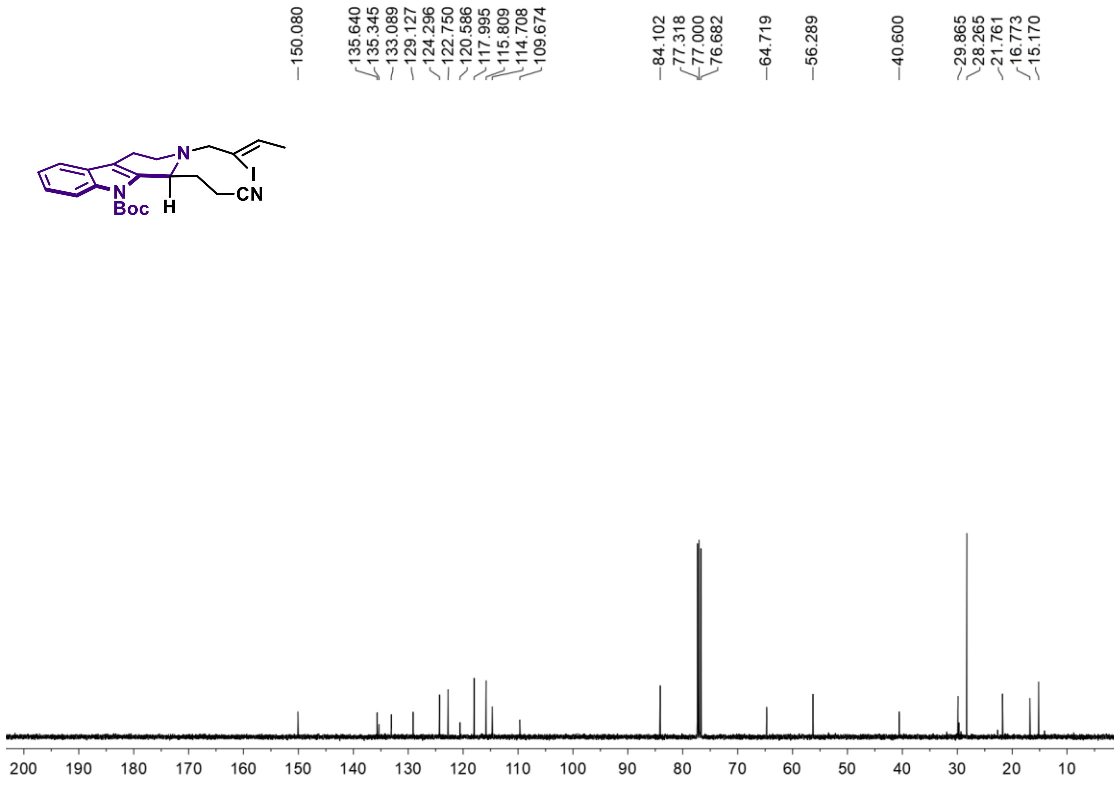

Supplementary Figure 239: ^{1}H NMR of 21 (400 MHz, $CDCl_3$)

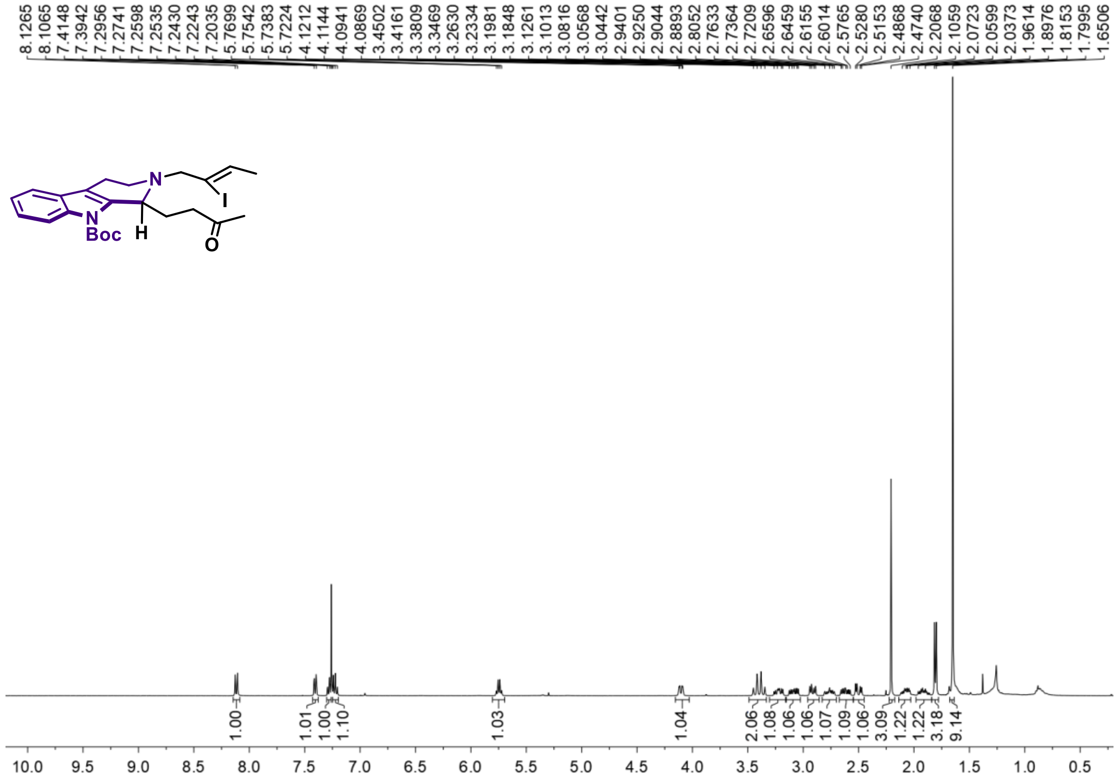

Supplementary Figure 240: ^{13}C NMR of 21 (101 MHz, $CDCl_3$)

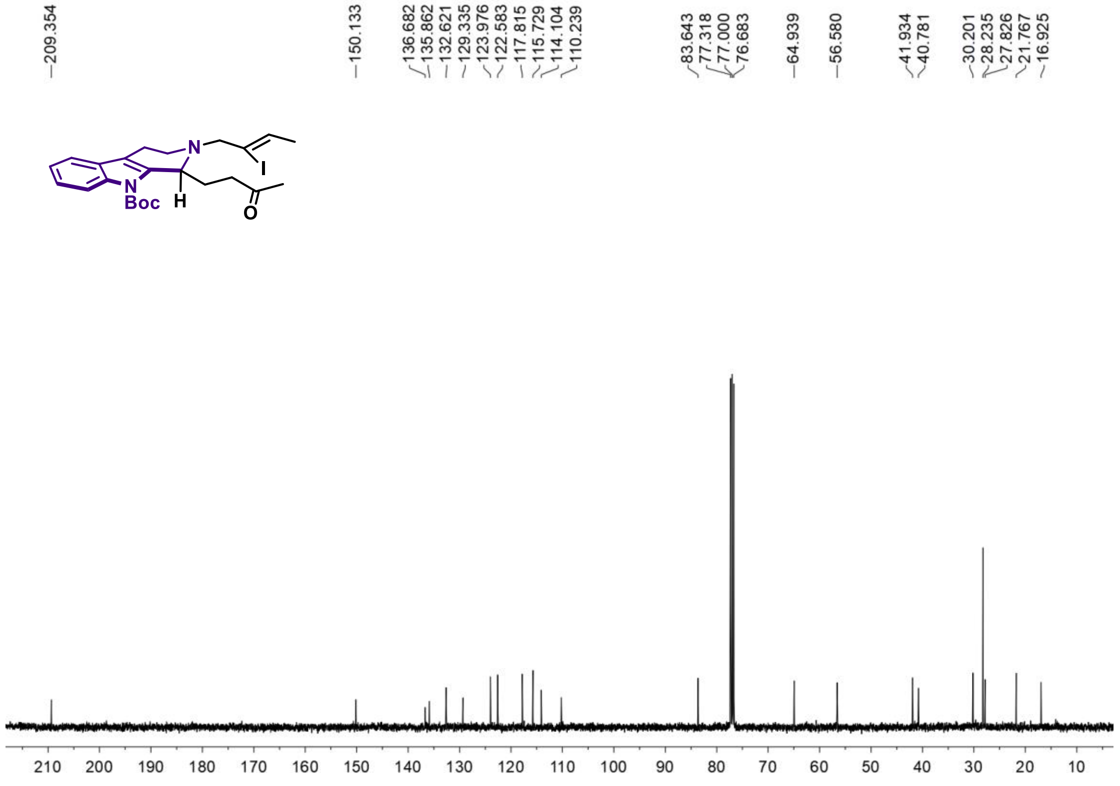

Supplementary Figure 241: ^{1}H NMR of 22 (400 MHz, $CDCl_3$)

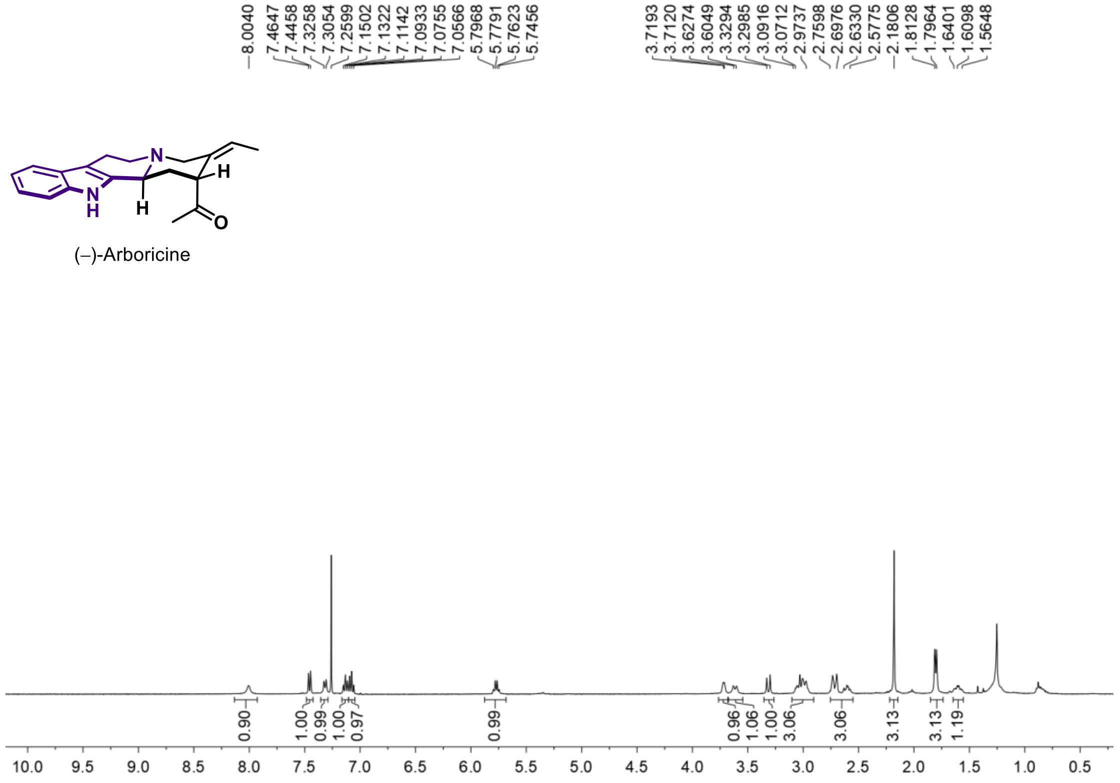

Supplementary Figure 242: ^{13}C NMR of 22 (101 MHz, $CDCl_3$)

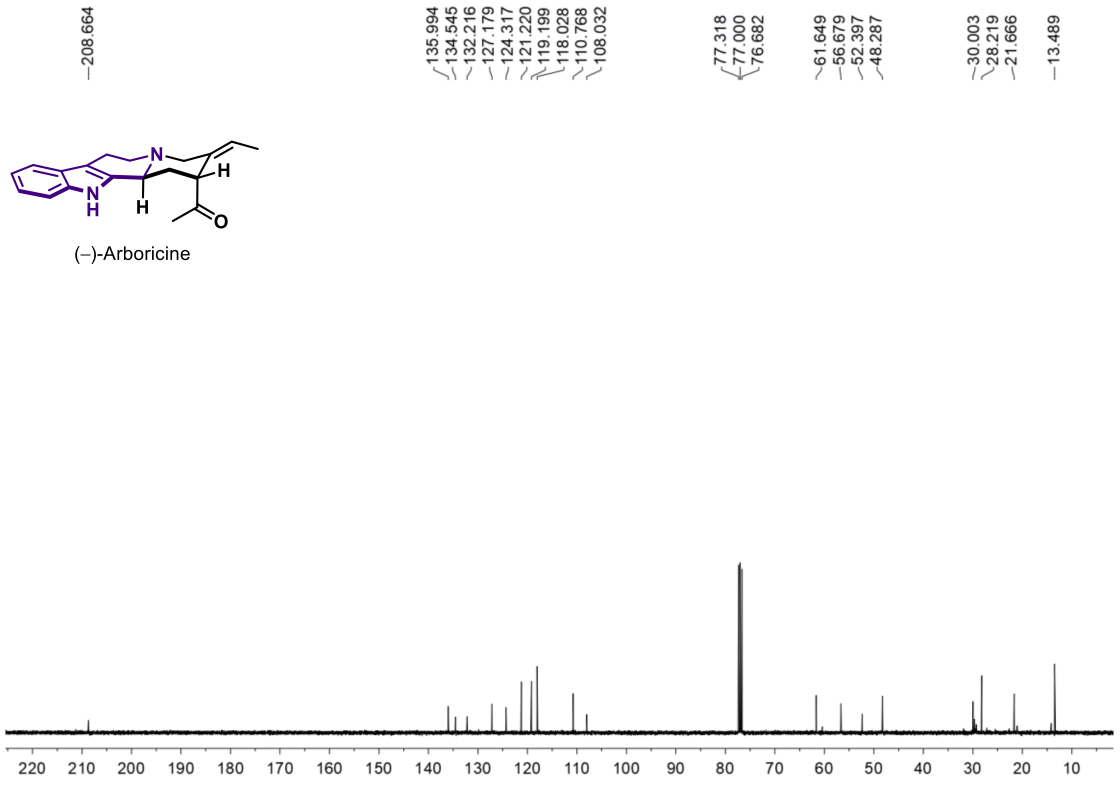

Supplementary Figure 243: ^{1}H NMR of 23 (400 MHz, $CDCl_3$)

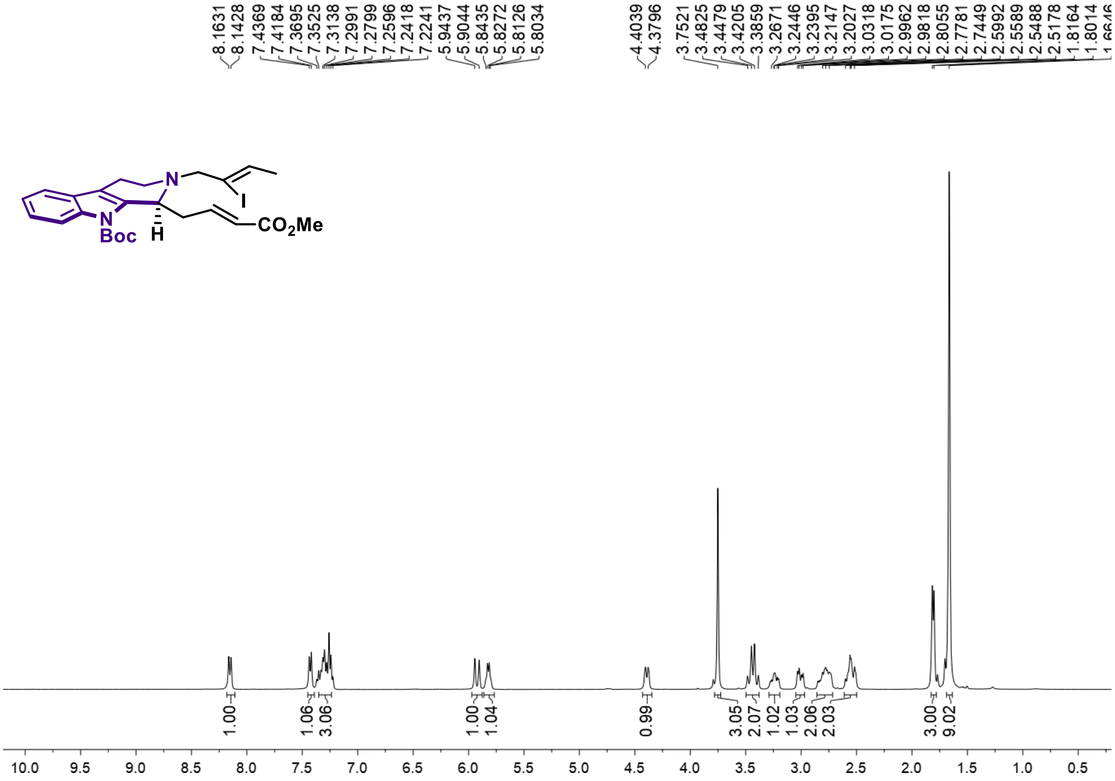

Supplementary Figure 244: ^{13}C NMR of 23 (101 MHz, $CDCl_3$)

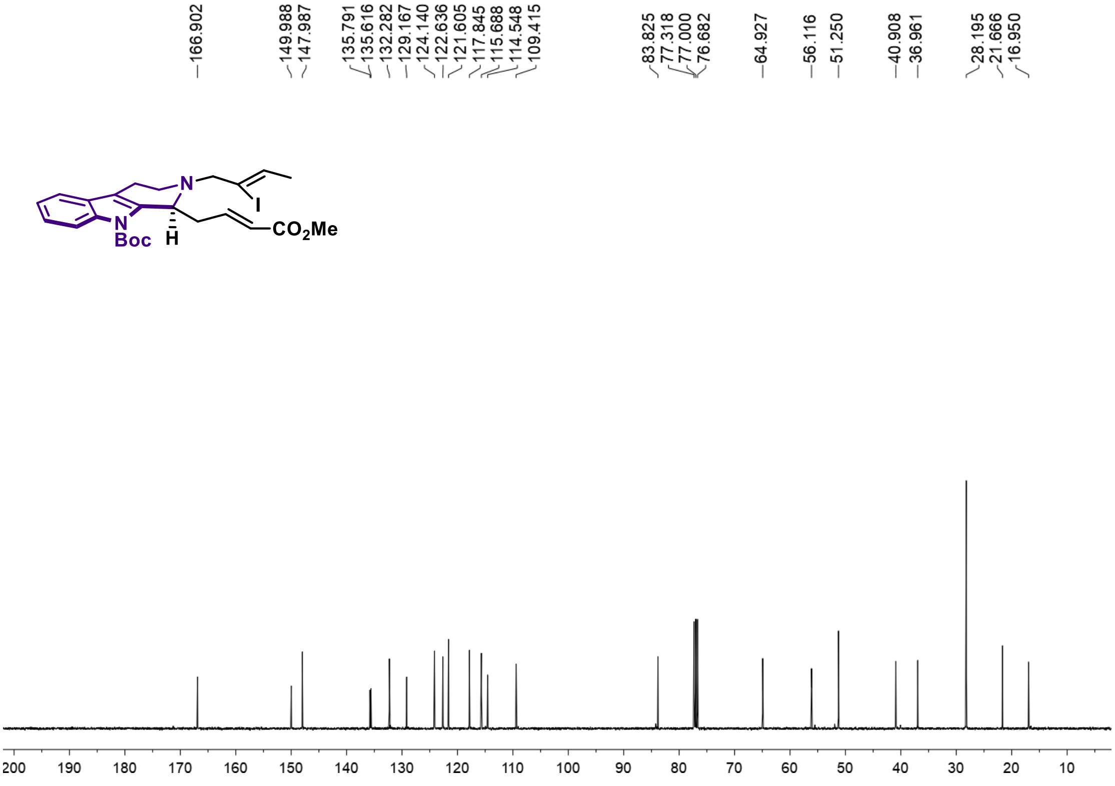

Supplementary Figure 245: ^{1}H NMR of 24 (400 MHz, $CDCl_3$)

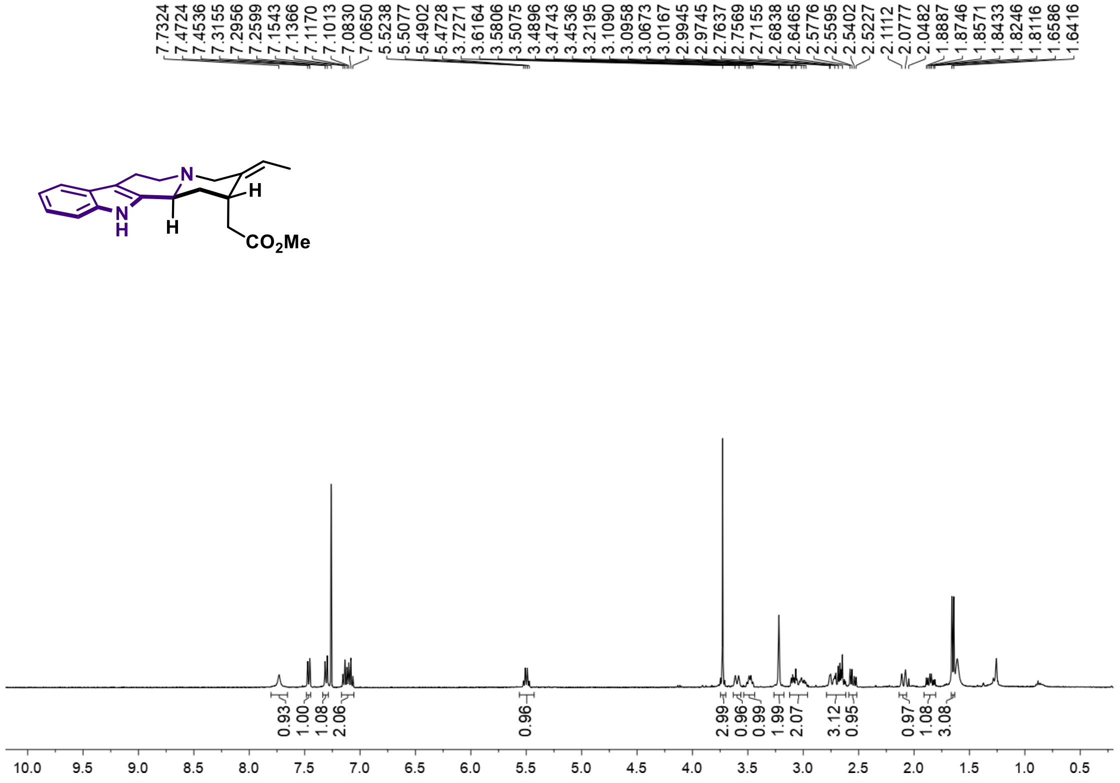

Supplementary Figure 246: ^{13}C NMR of 24 (101 MHz, $CDCl_3$)

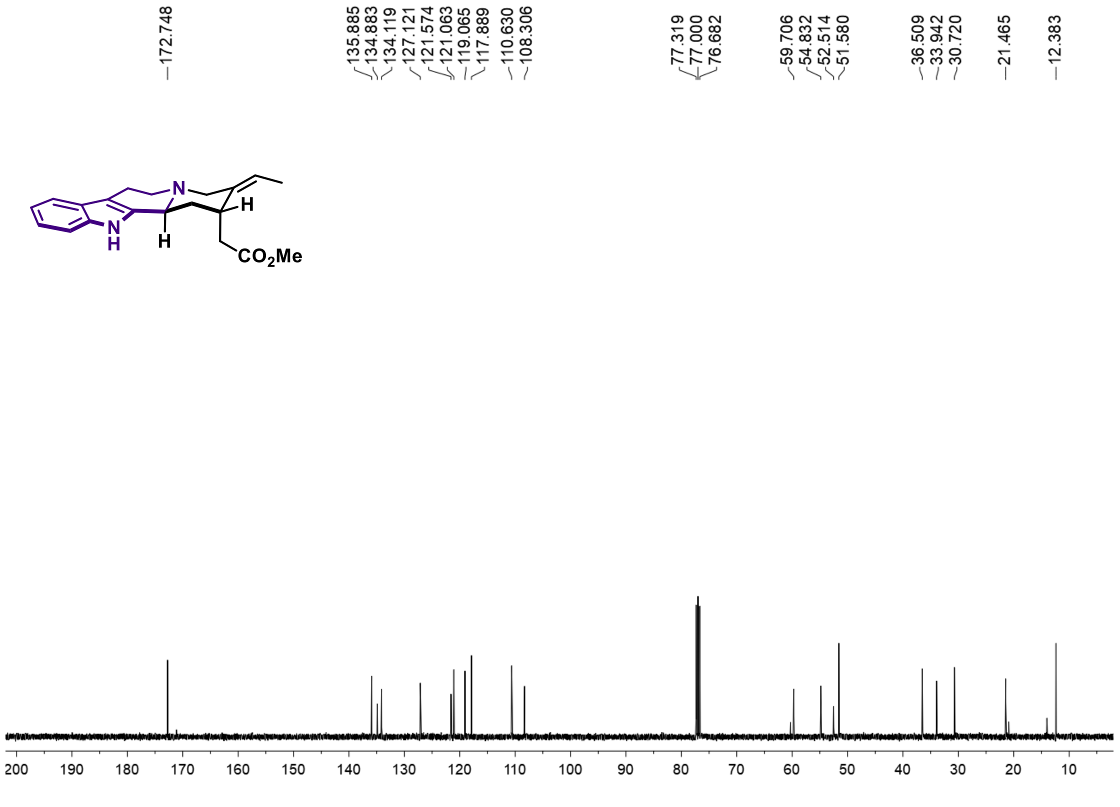

Supplementary Figure 247: ^{1}H NMR of 25 (400 MHz, $CDCl_3$)

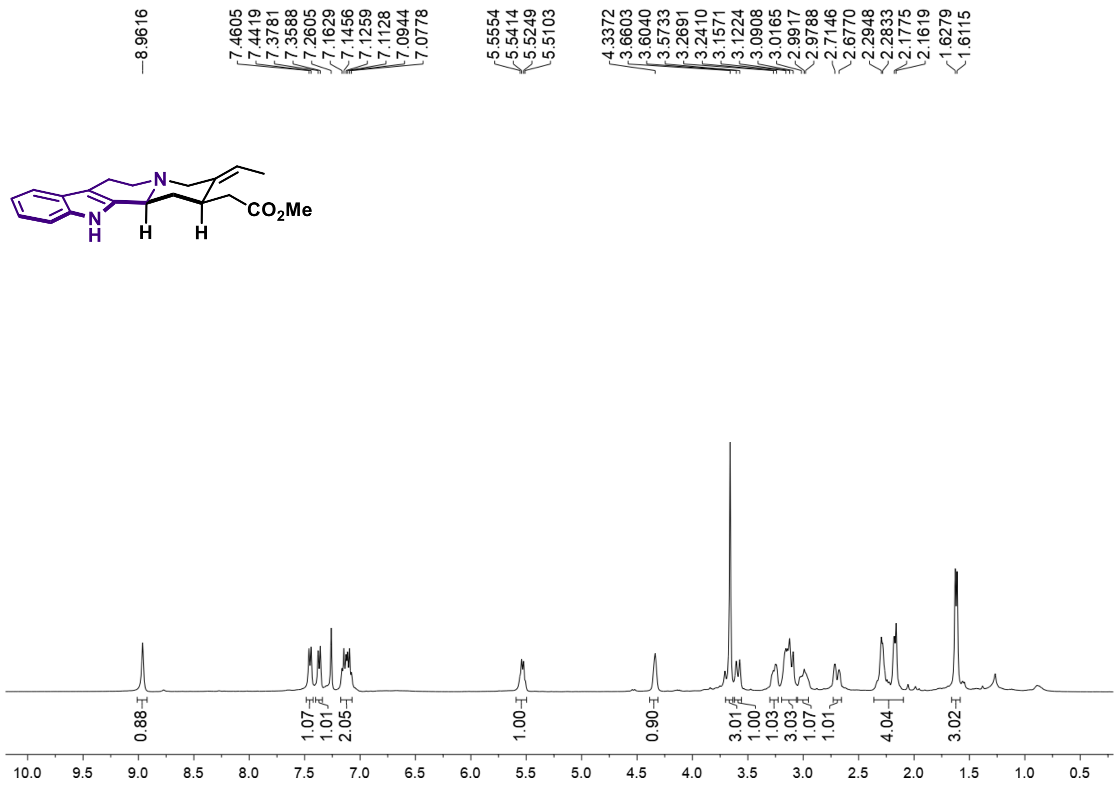

Supplementary Figure 248: ^{13}C NMR of 25 (101 MHz, $CDCl_3$)

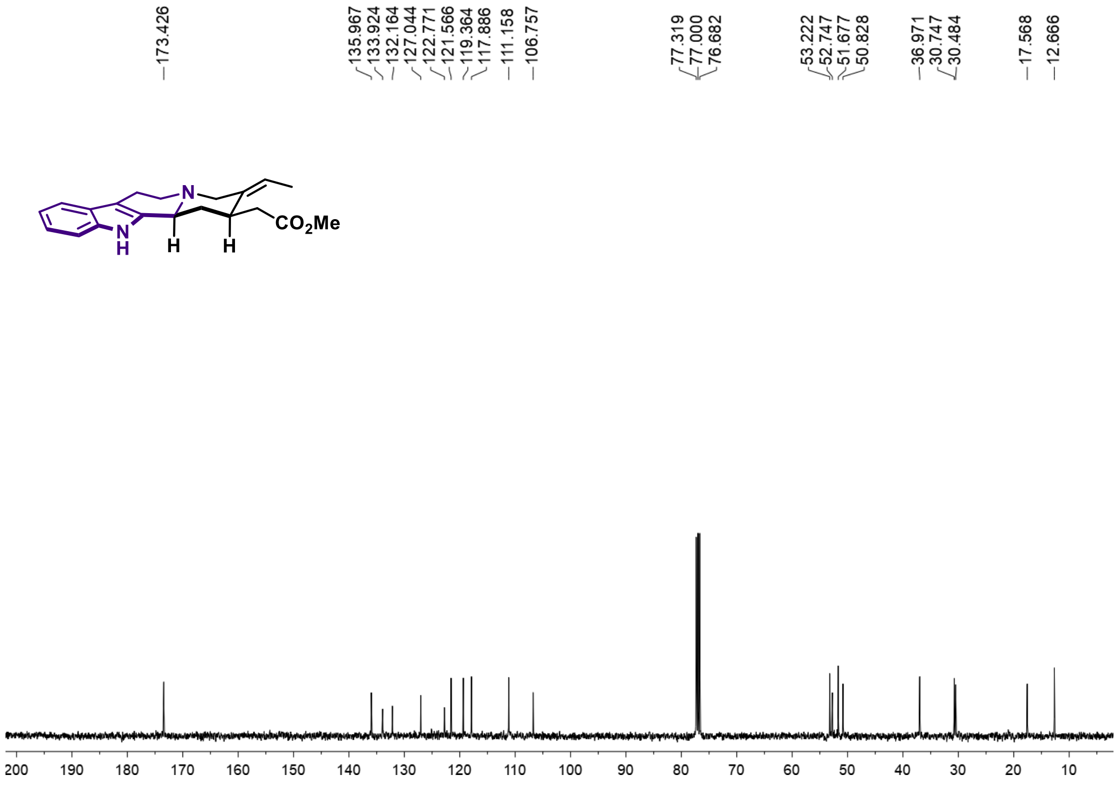

Supplementary Figure 249: ^{1}H NMR of 26 (400 MHz, CDCl$_3$)

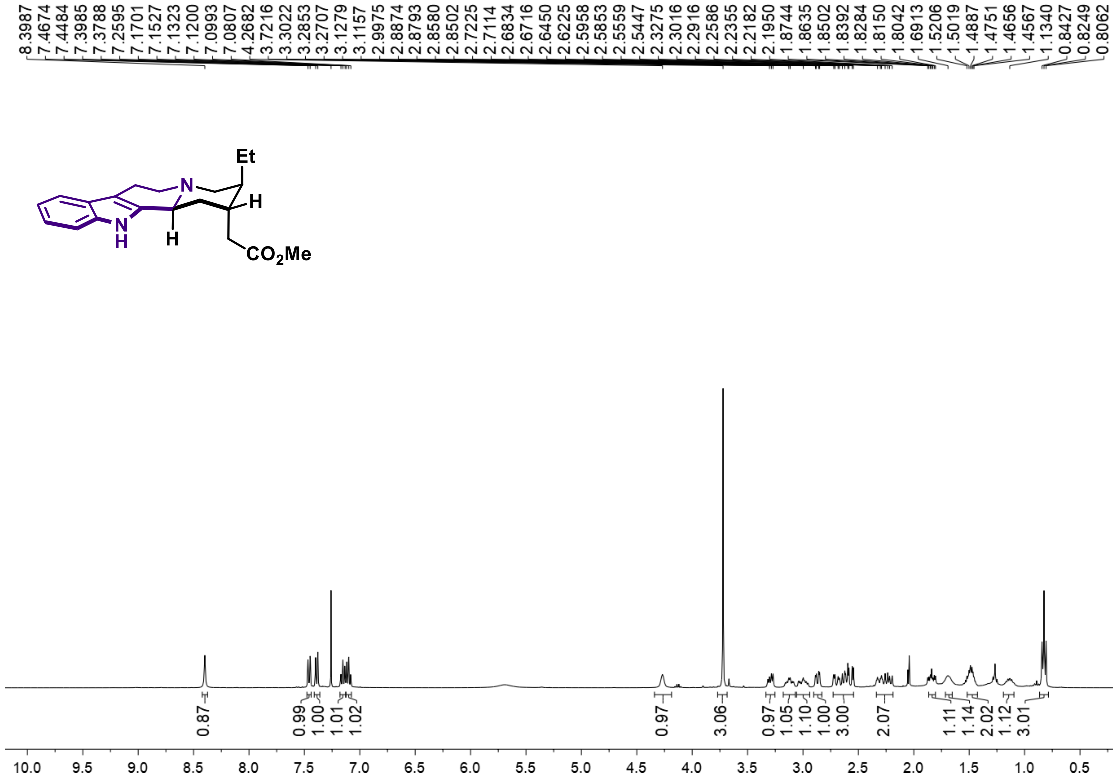

Supplementary Figure 250: ^{13}C NMR of 26 (101 MHz, CDCl$_3$)

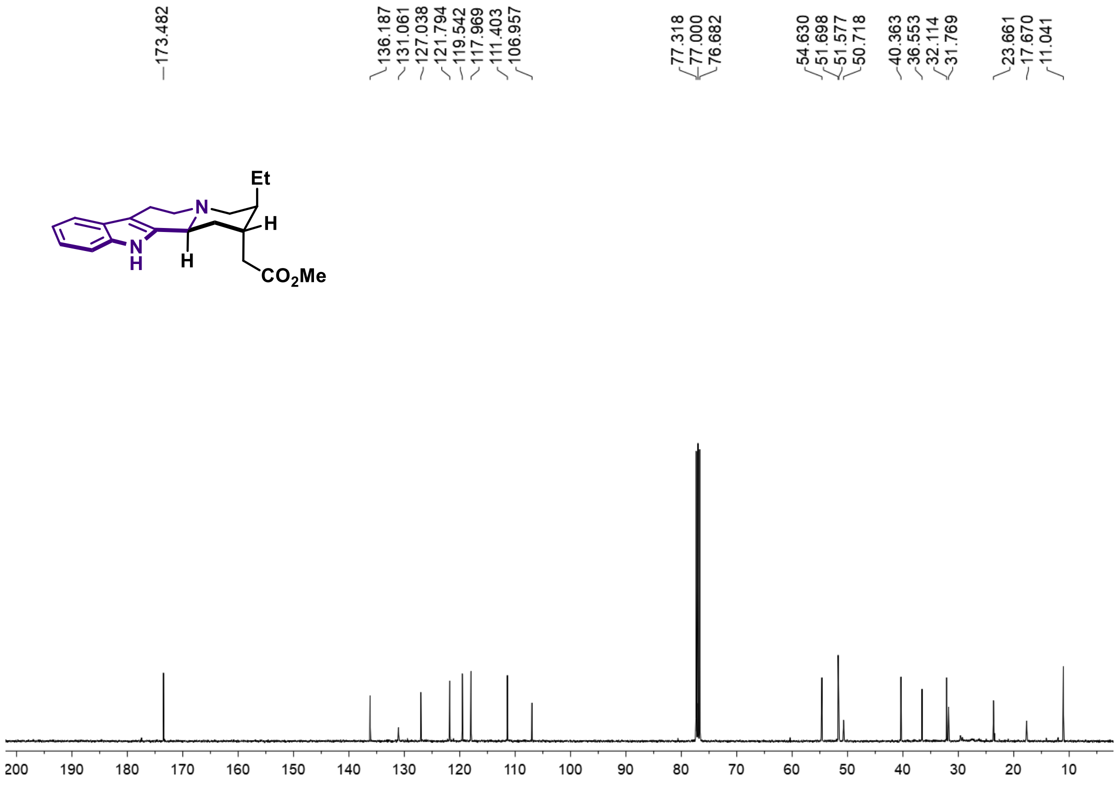

Supplementary Figure 251: ^{1}H NMR of 27 (400 MHz, $CDCl_3$)

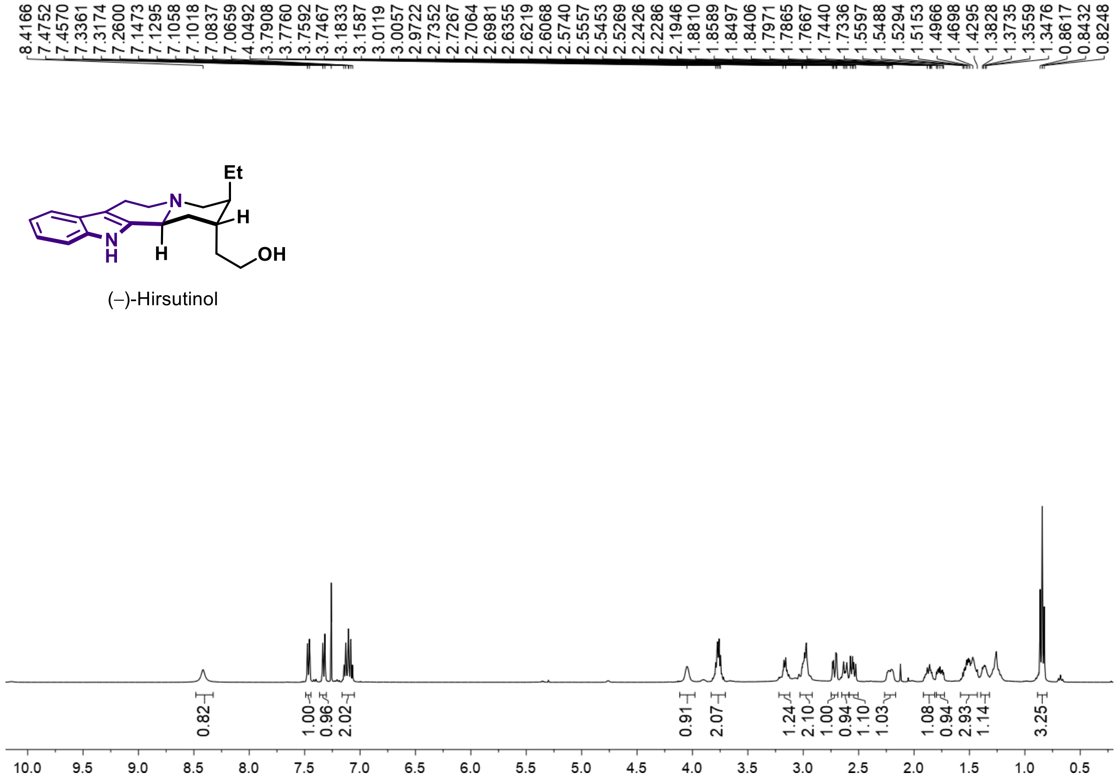

Supplementary Figure 252: ^{13}C NMR of 27 (101 MHz, $CDCl_3$)

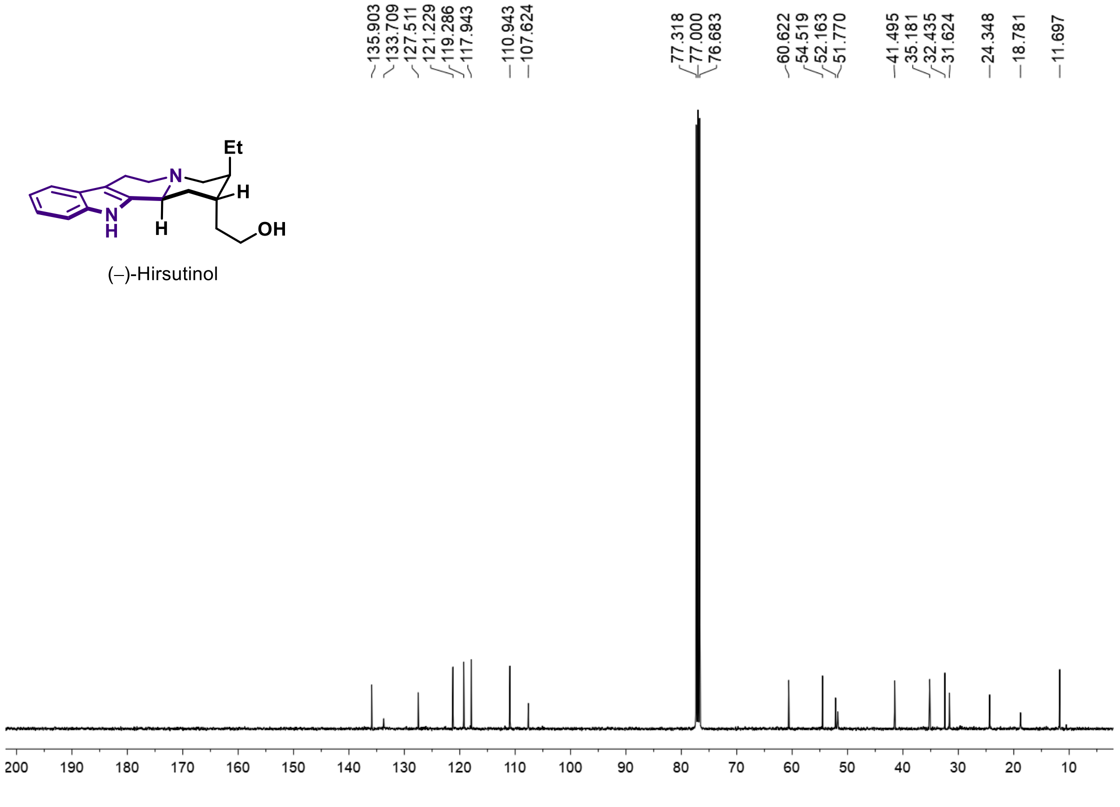

Supplementary Figure 253: ^{1}H NMR of 29 (400 MHz, $CDCl_3$)

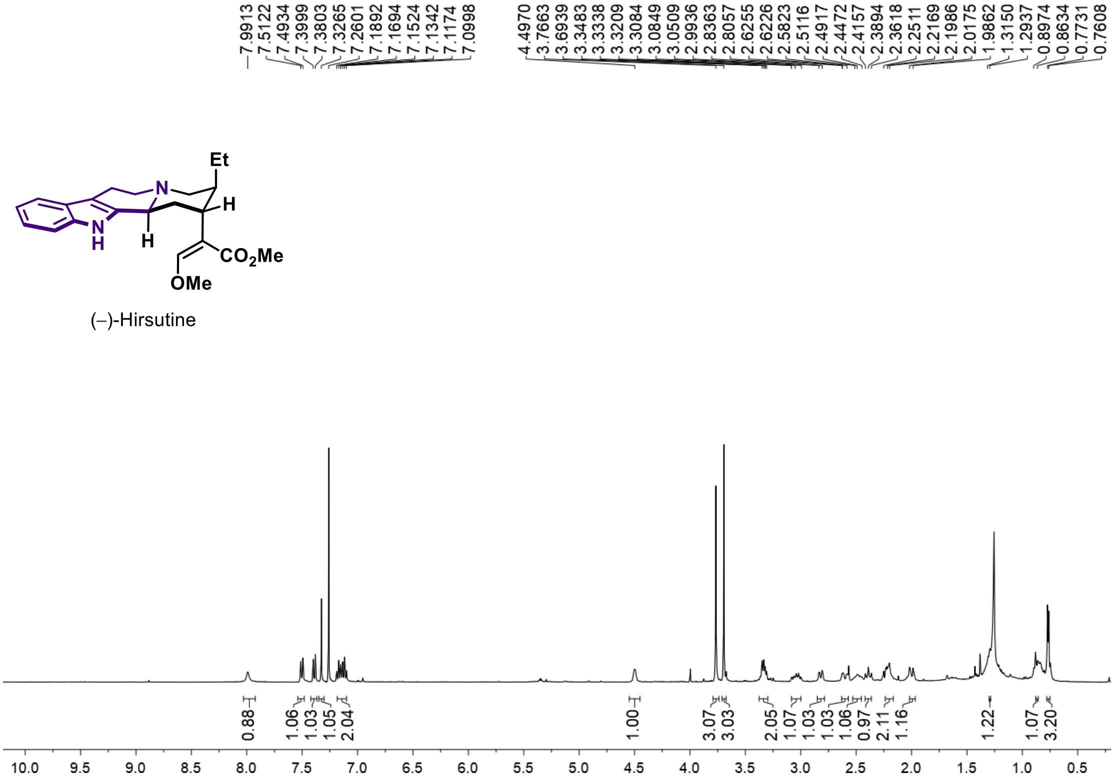

Supplementary Figure 254: ^{13}C NMR of 29 (101 MHz, $CDCl_3$)

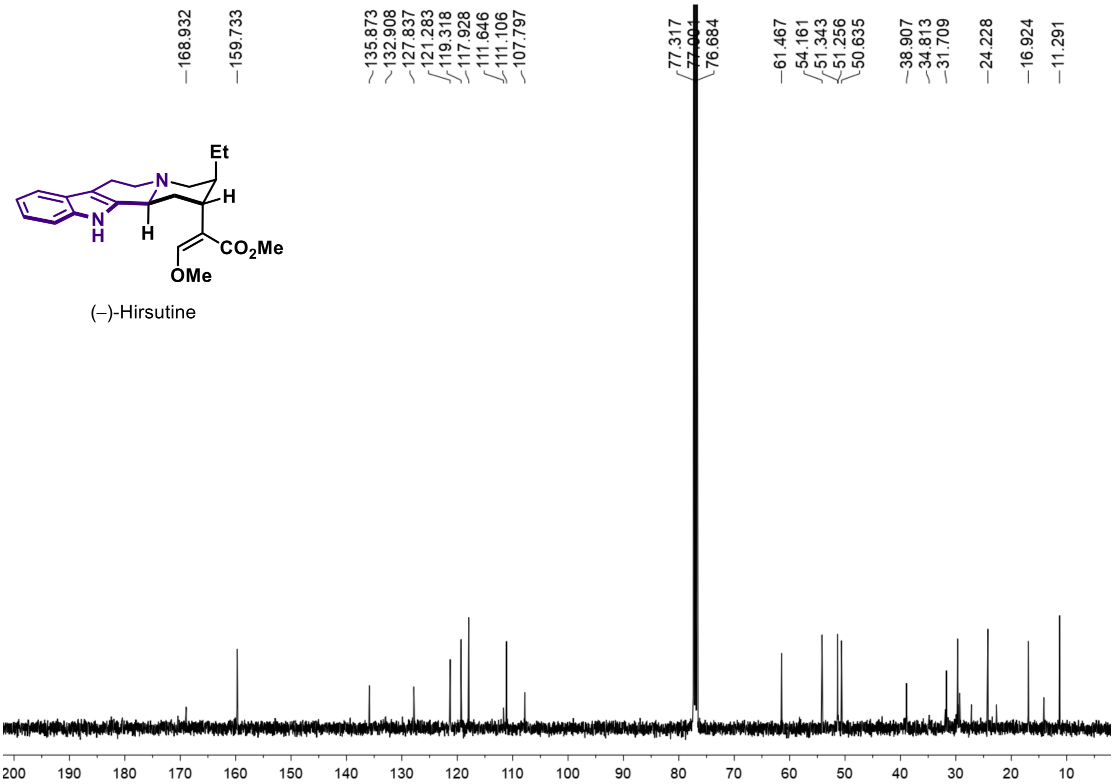

Supplementary Figure 255: ^{1}H NMR of 30 (400 MHz, $CDCl_3$)

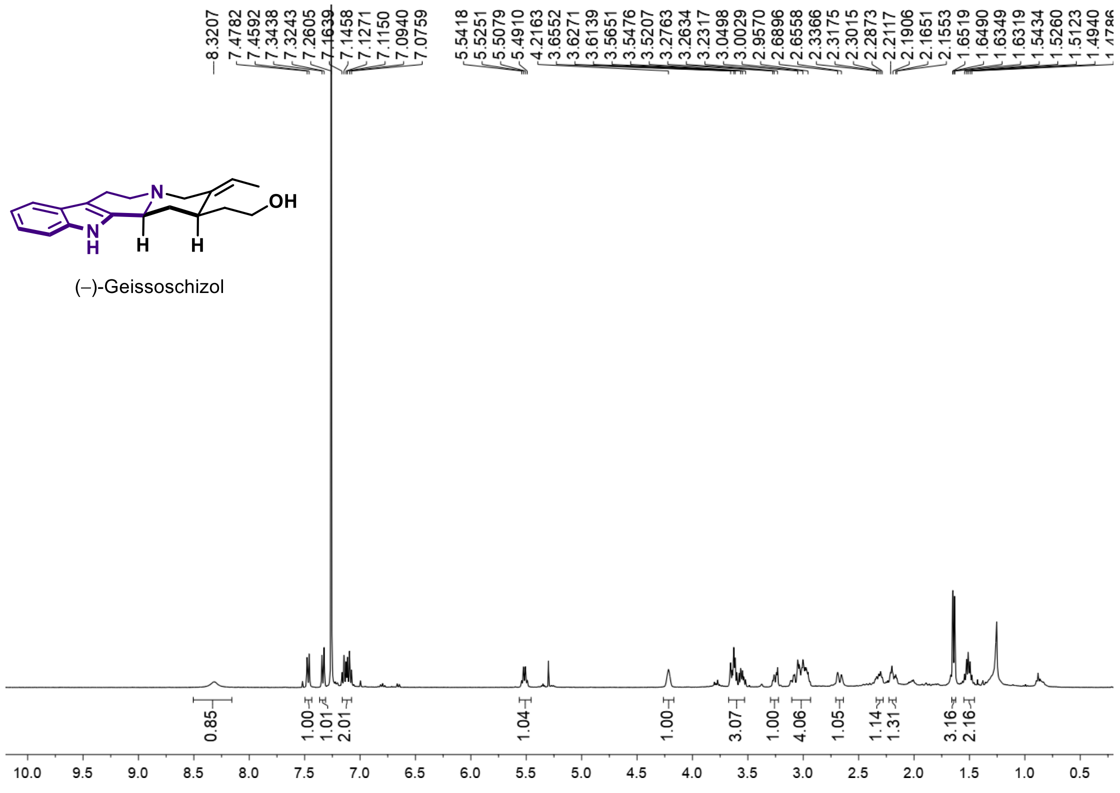

Supplementary Figure 256: ^{1}H NMR of 30 (101 MHz, $CDCl_3$)

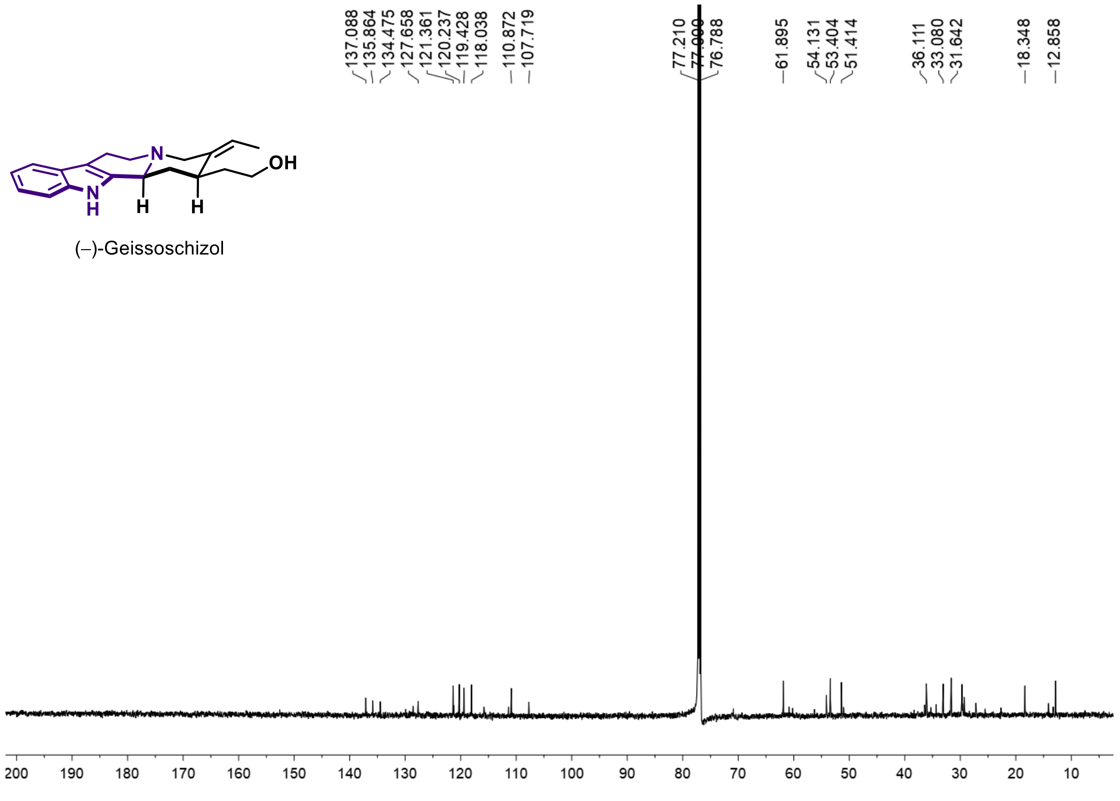

Supplementary Figure 257: ^{1}H NMR of 31 (400 MHz, CDCl$_3$)

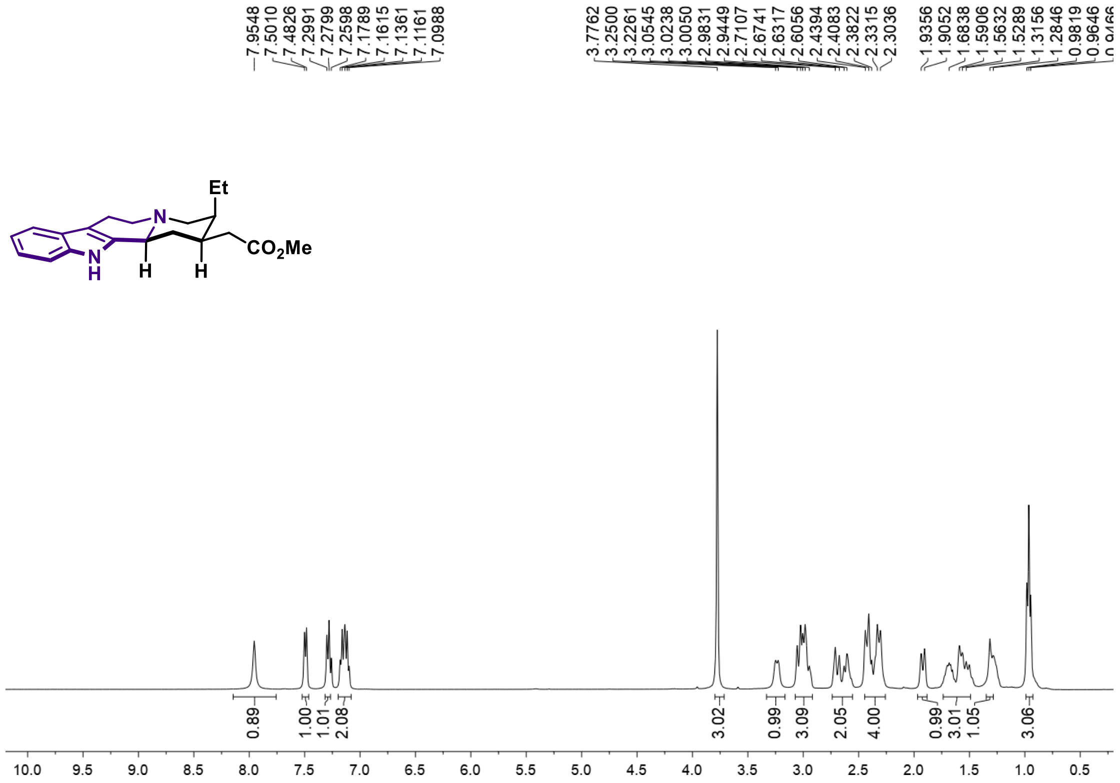

Supplementary Figure 258: ^{13}C NMR of 31 (101 MHz, CDCl$_3$)

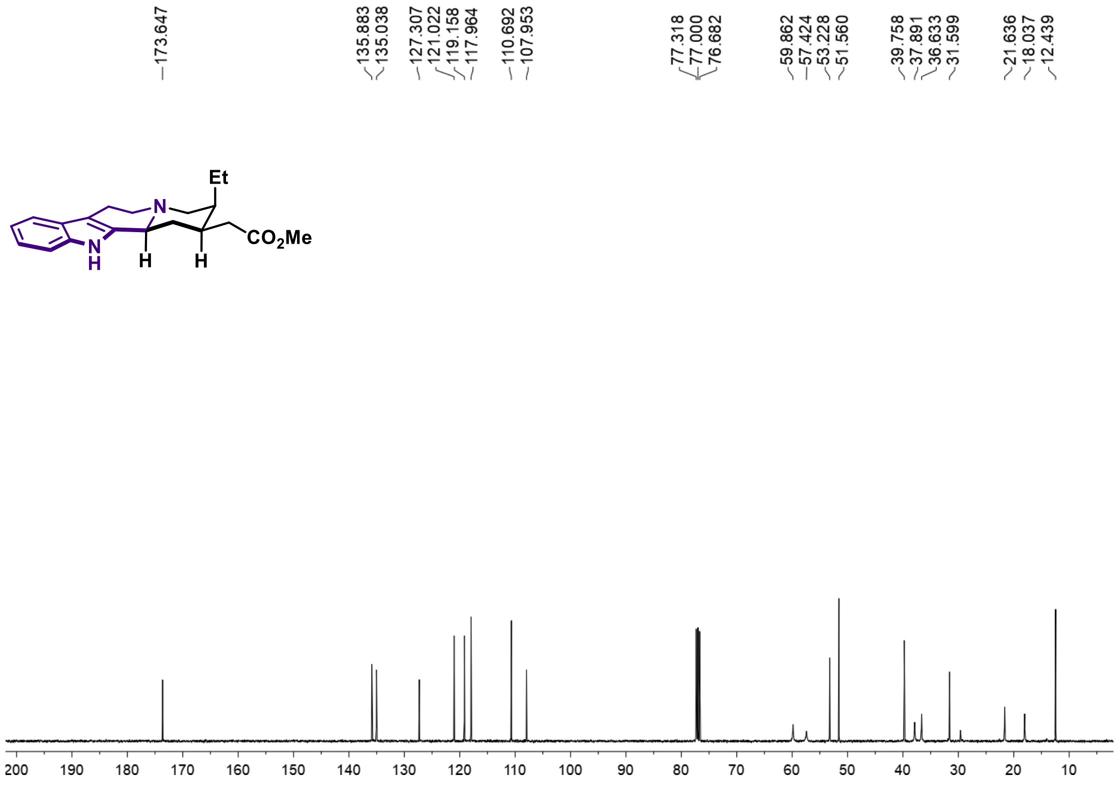

Supplementary Figure 259: ^{1}H NMR of 32 (400 MHz, $CDCl_3$)

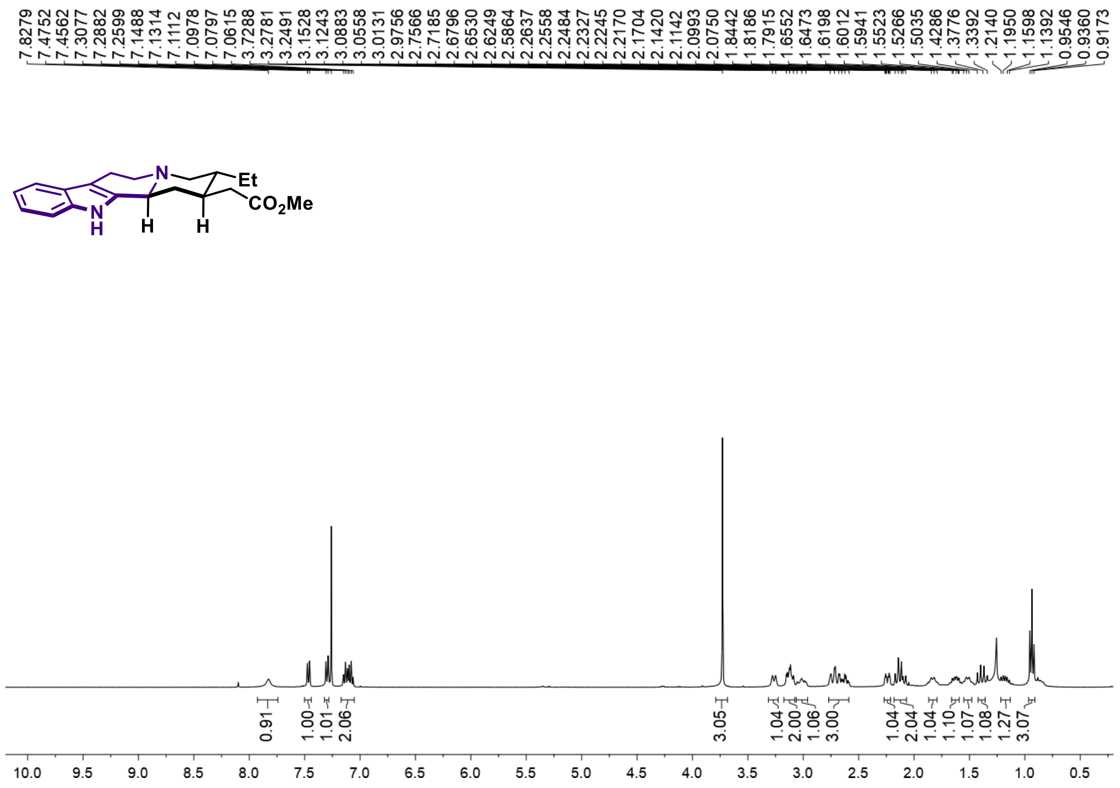

Supplementary Figure 260: ^{13}C NMR of 32 (101 MHz, $CDCl_3$)

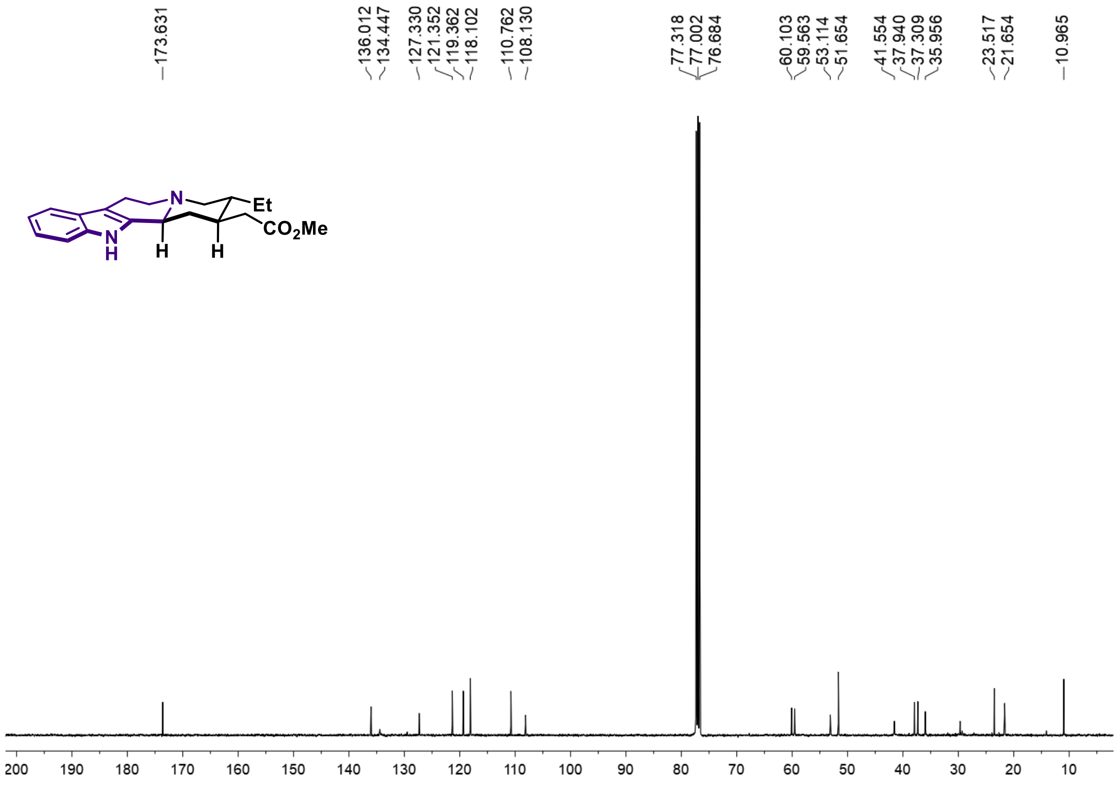

Supplementary Figure 261: ^{1}H NMR of 33 (400 MHz, $CDCl_3$)

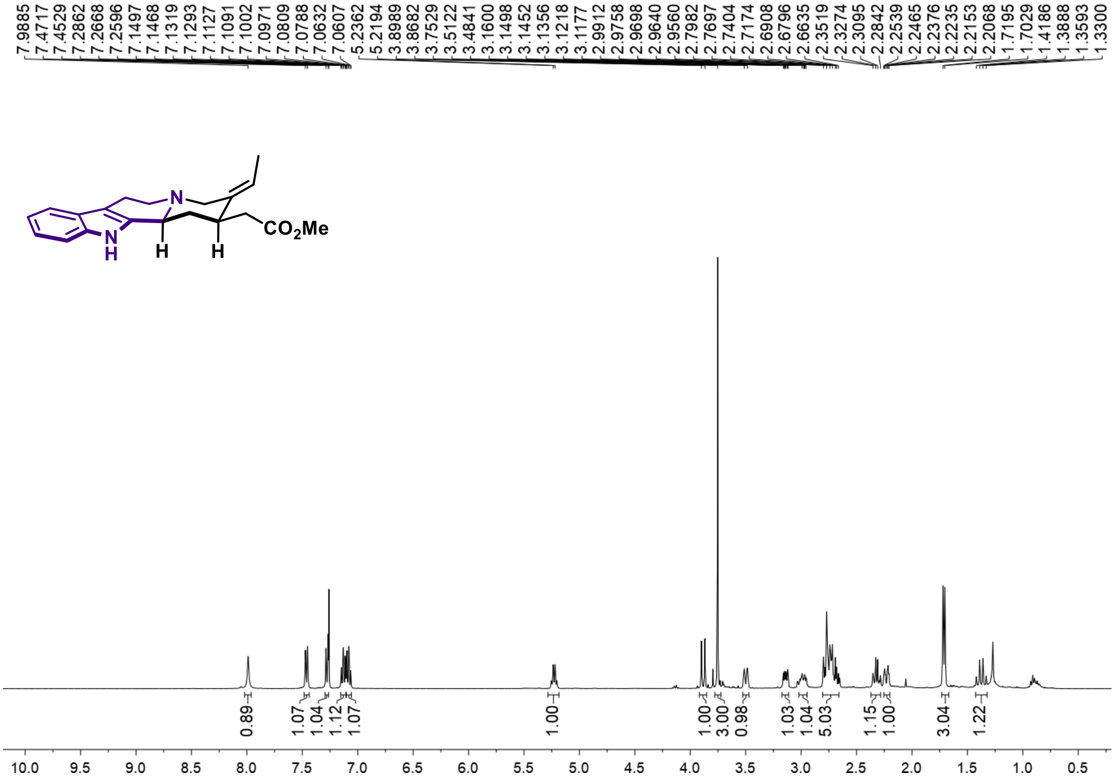

Supplementary Figure 262: ^{13}C NMR of 33 (101 MHz, $CDCl_3$)

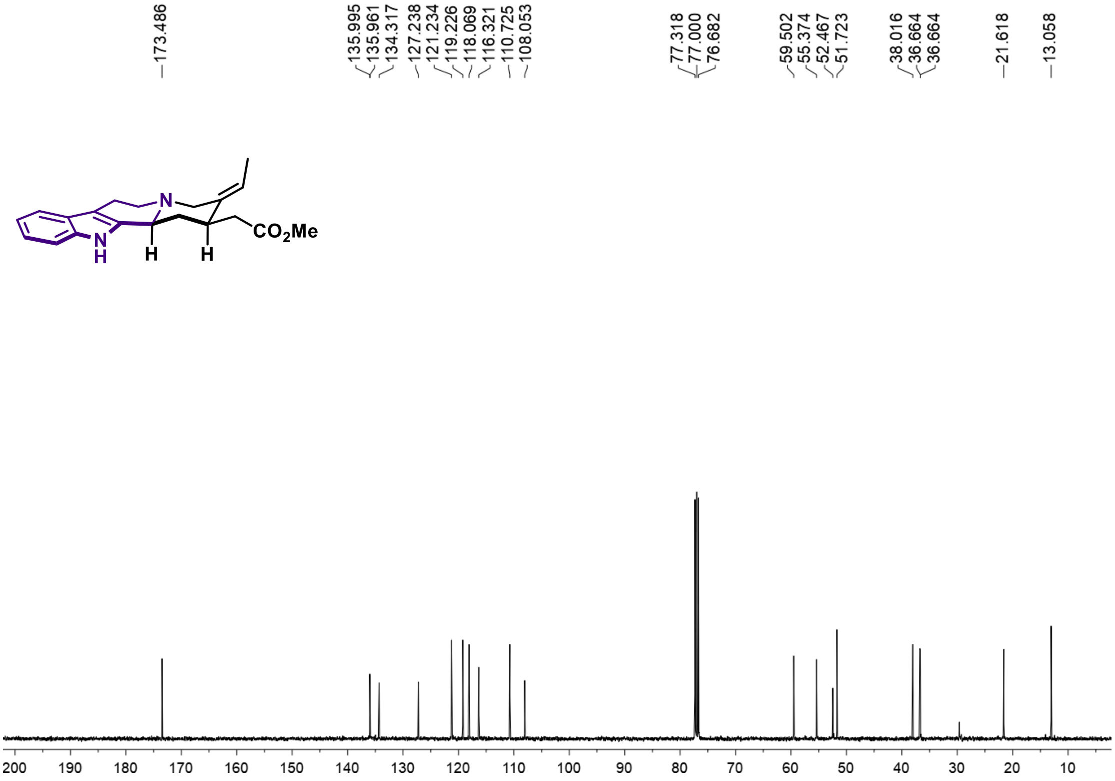

Supplementary Figure 263: ^{1}H NMR of 34 (400 MHz, $CDCl_3$)

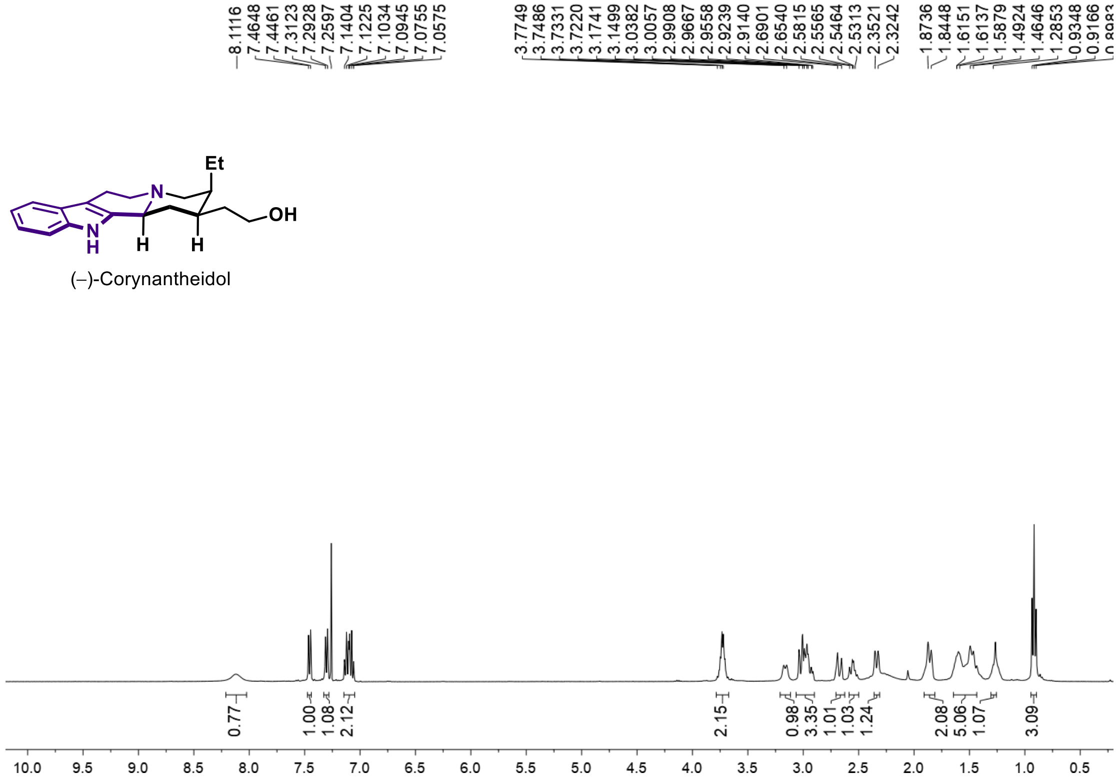

Supplementary Figure 264: ^{13}C NMR of 34 (101 MHz, $CDCl_3$)

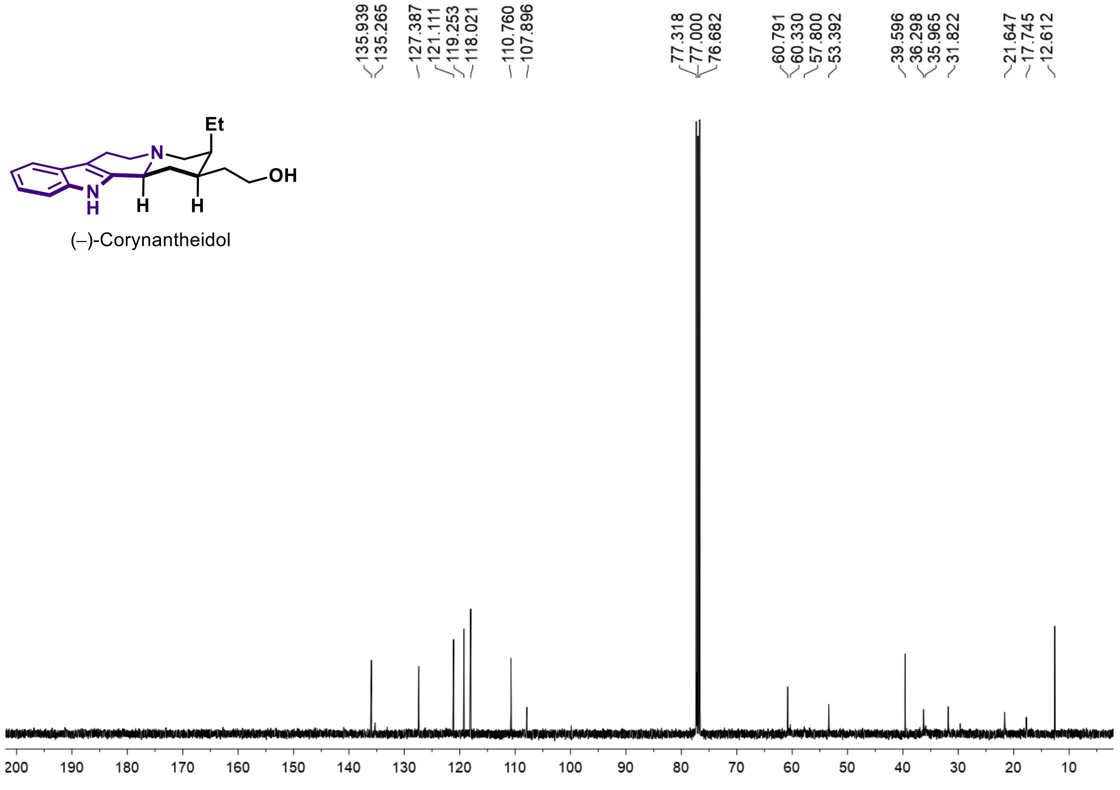

Supplementary Figure 265: ^{1}H NMR of 35 (400 MHz, $CDCl_3$)

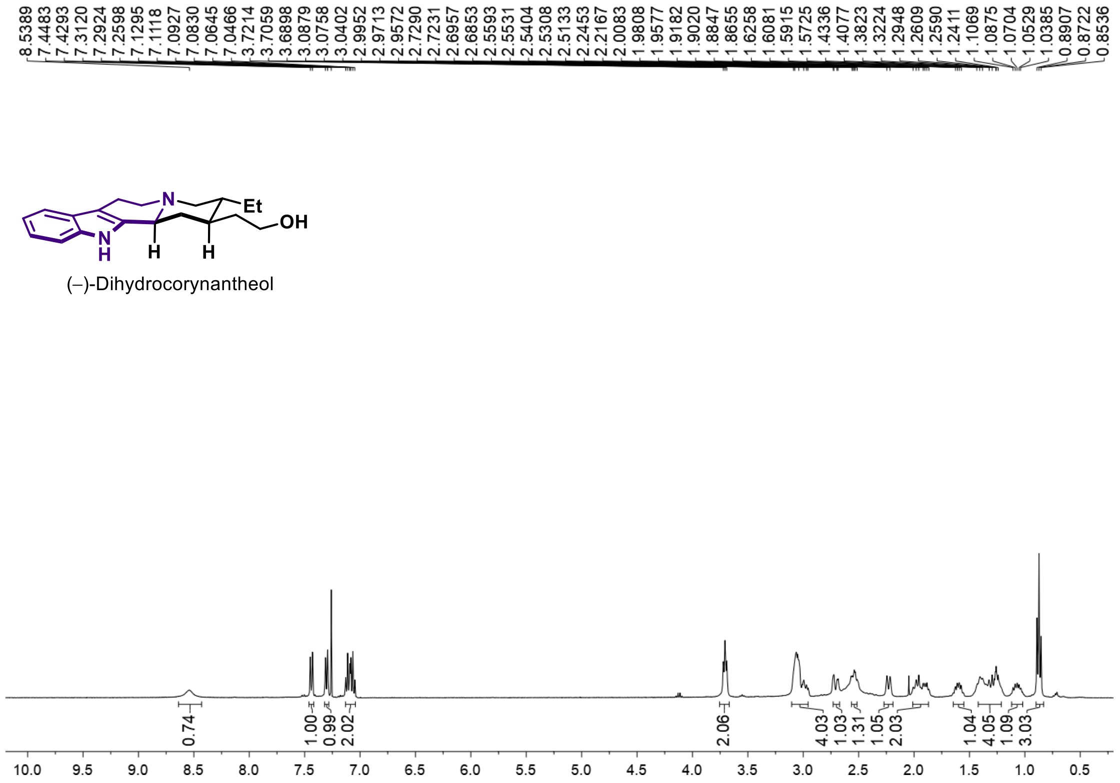

Supplementary Figure 266: ^{13}C NMR of 35 (101 MHz, $CDCl_3$)

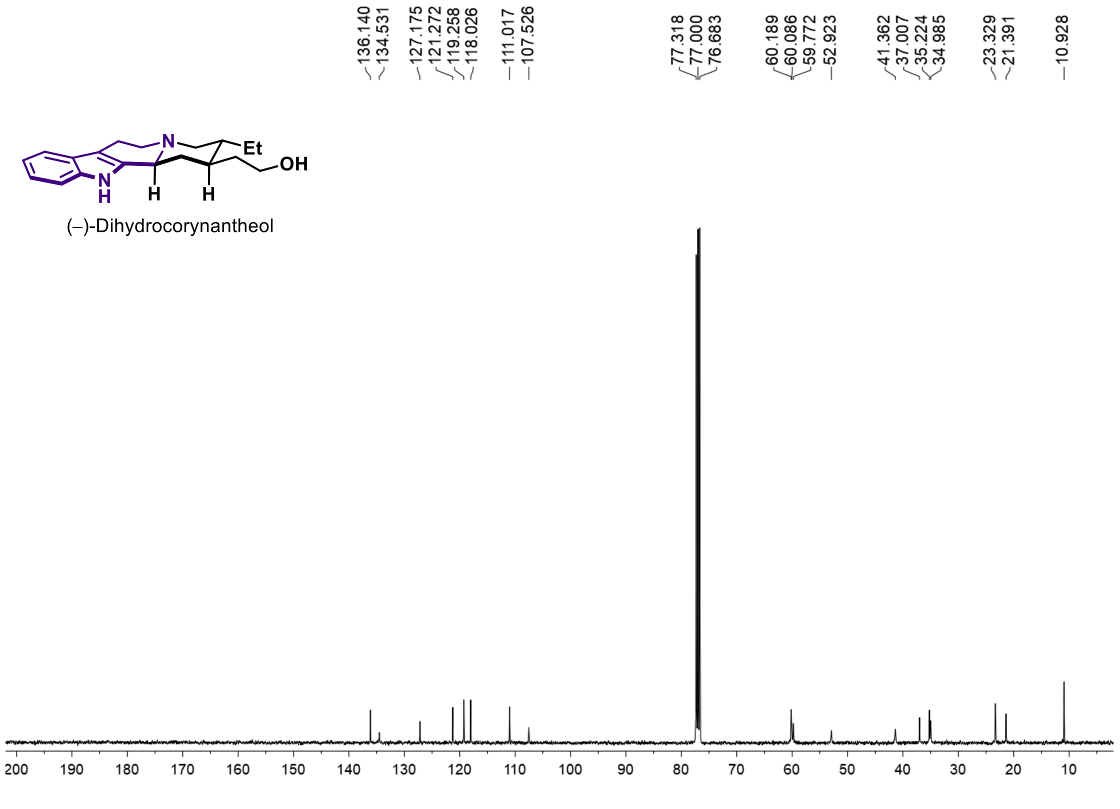

Supplementary Figure 267: ^{1}H NMR of 36 (400 MHz, $CDCl_3$)

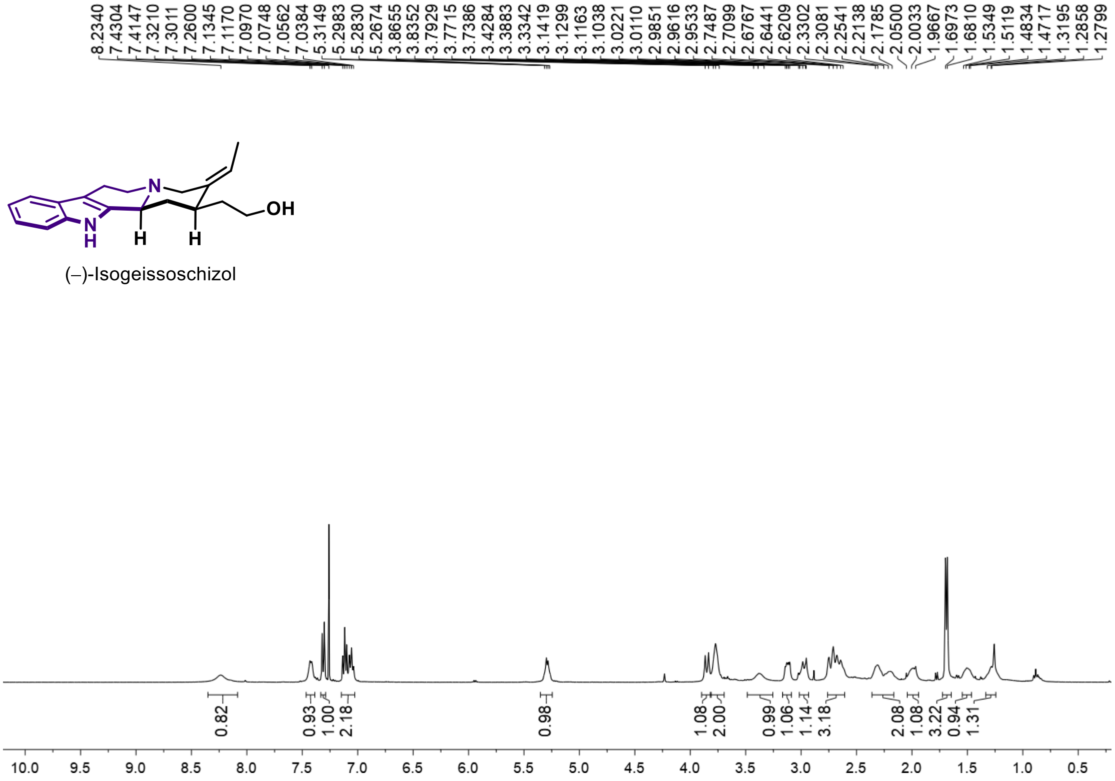

Supplementary Figure 268: ^{13}C NMR of 36 (101 MHz, $CDCl_3$)

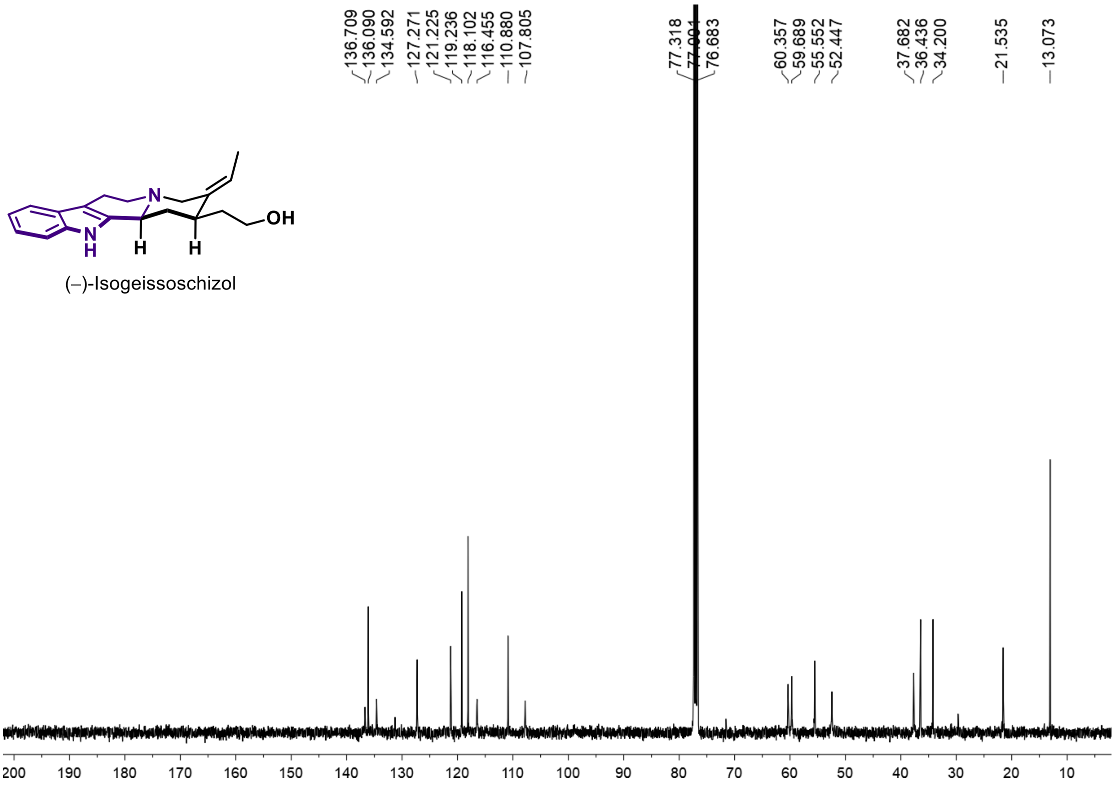

Supplementary Figure 269: ¹H NMR of 37 (400 MHz, CDCl₃)

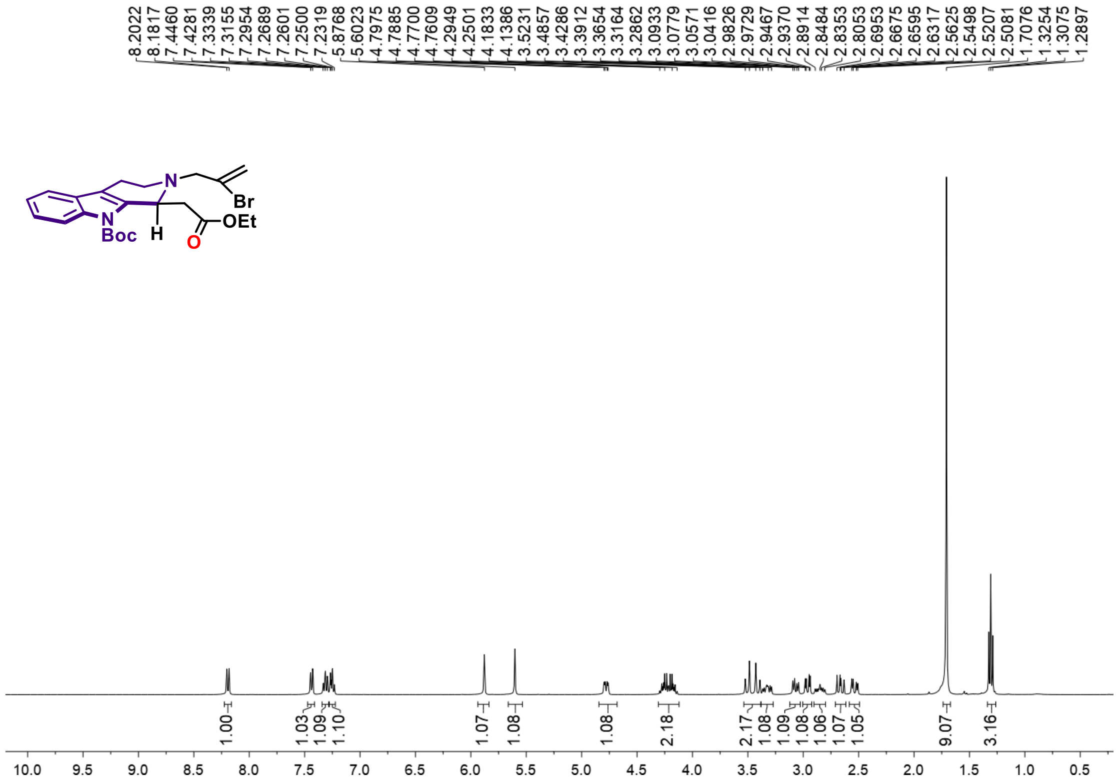

Supplementary Figure 270: ¹³C NMR of 37 (101 MHz, CDCl₃)

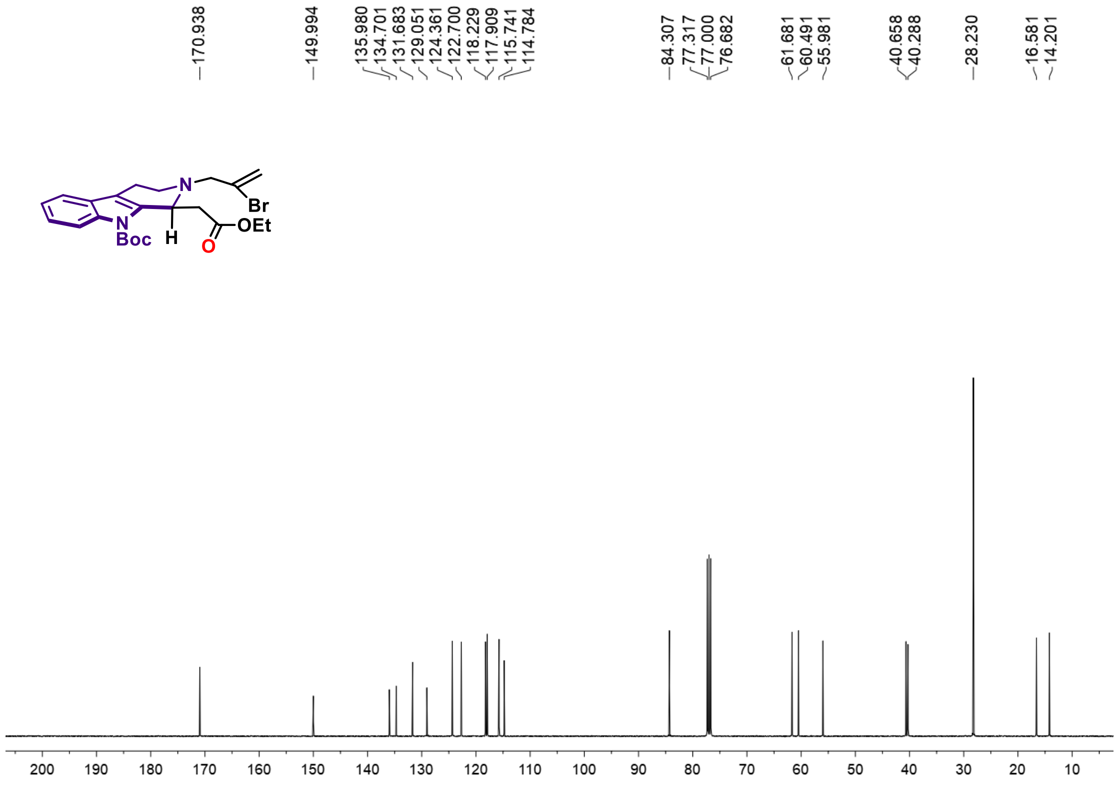

Supplementary Figure 271: ^{1}H NMR of 38 (400 MHz, CDCl$_3$)

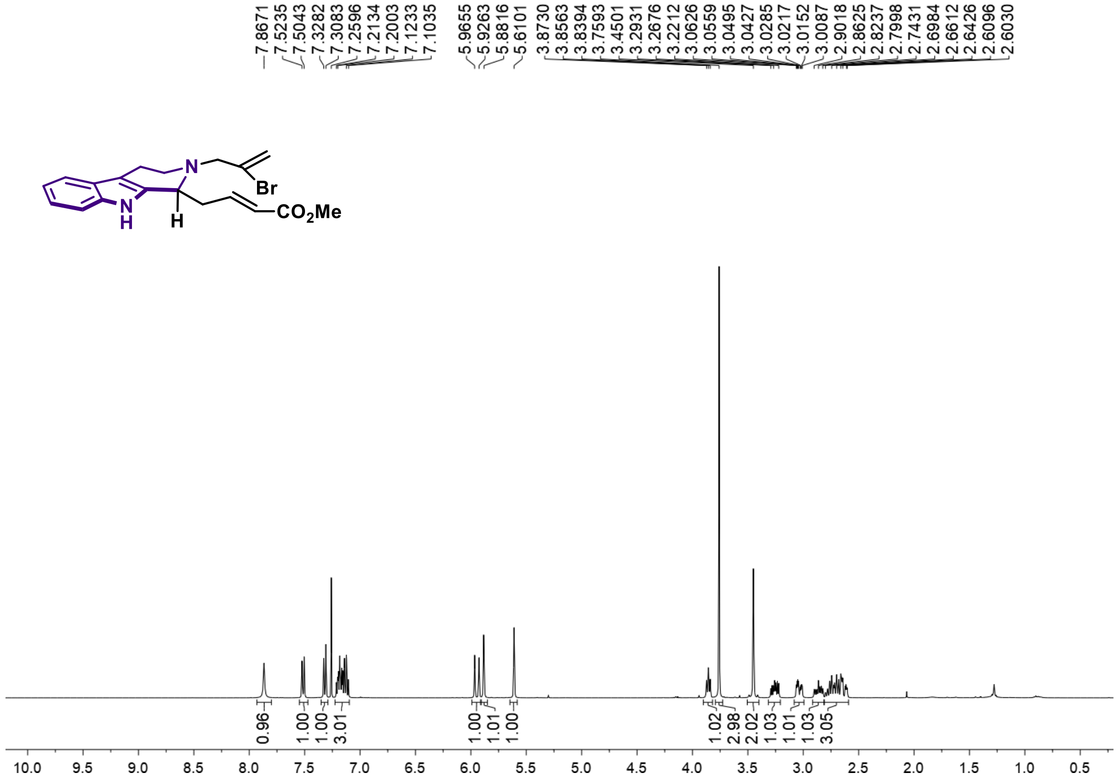

Supplementary Figure 272: ^{13}C NMR of 38 (101 MHz, CDCl$_3$)

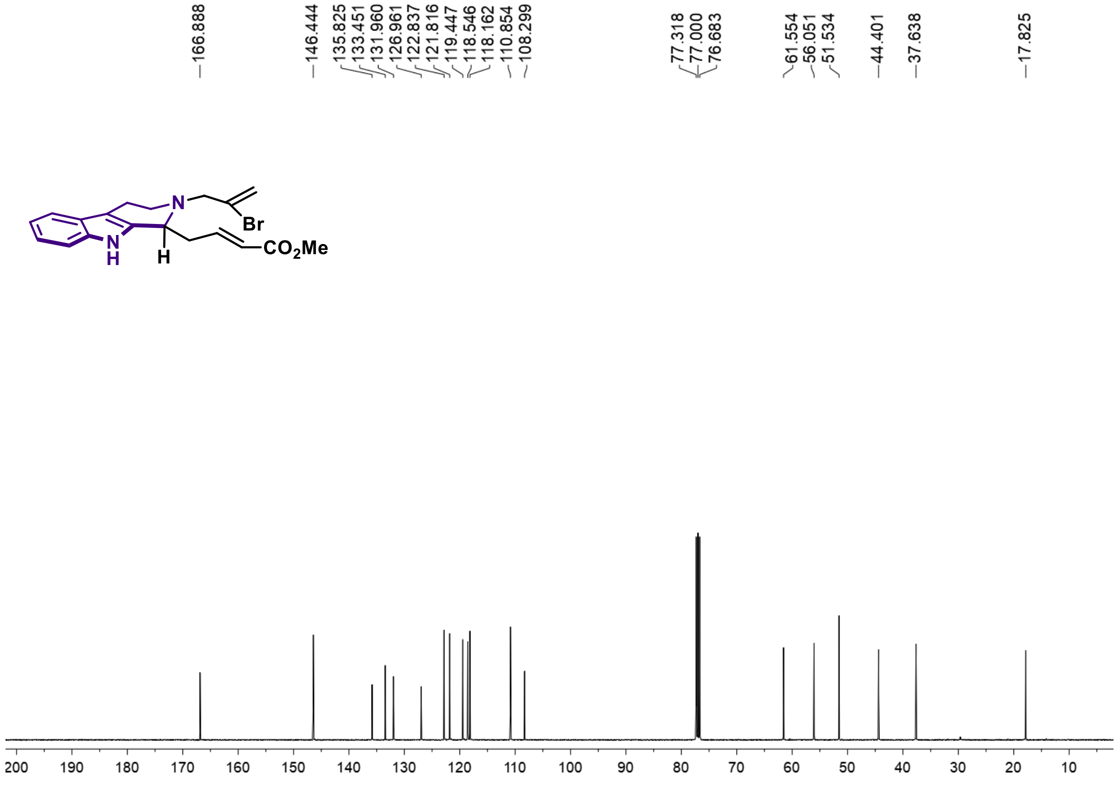

Supplementary Figure 273: ^{1}H NMR of 39 (400 MHz, $CDCl_3$)

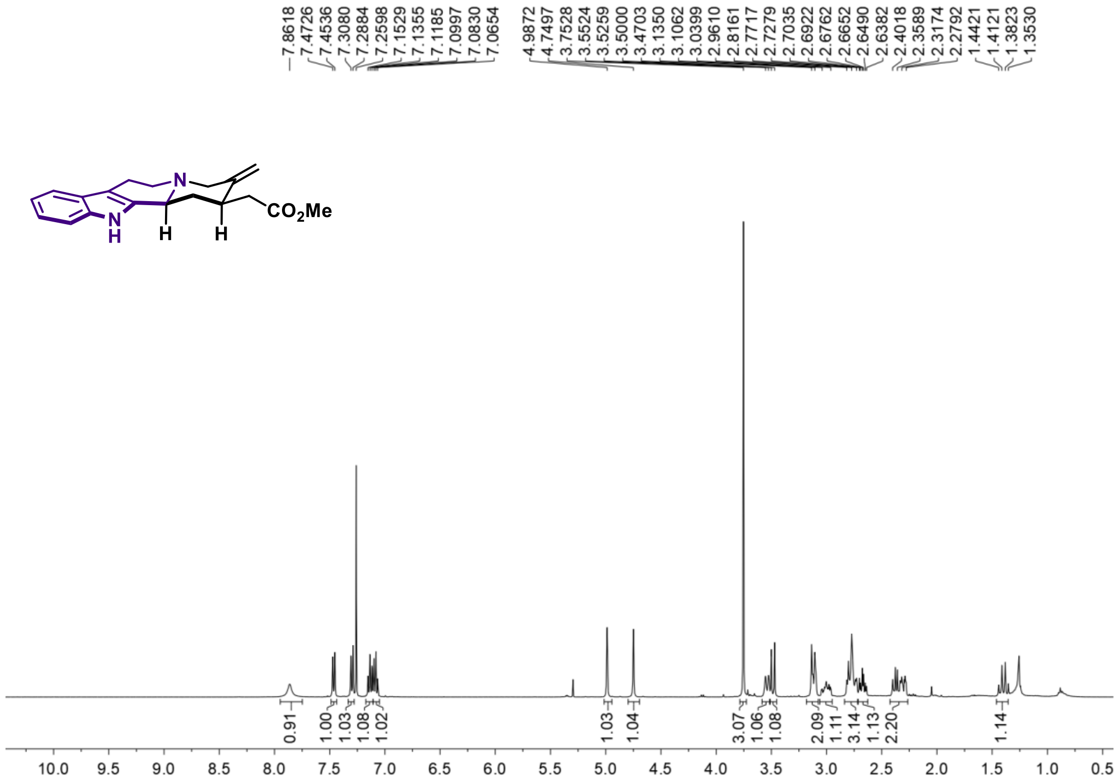

Supplementary Figure 274: ^{13}C NMR of 39 (101 MHz, $CDCl_3$)

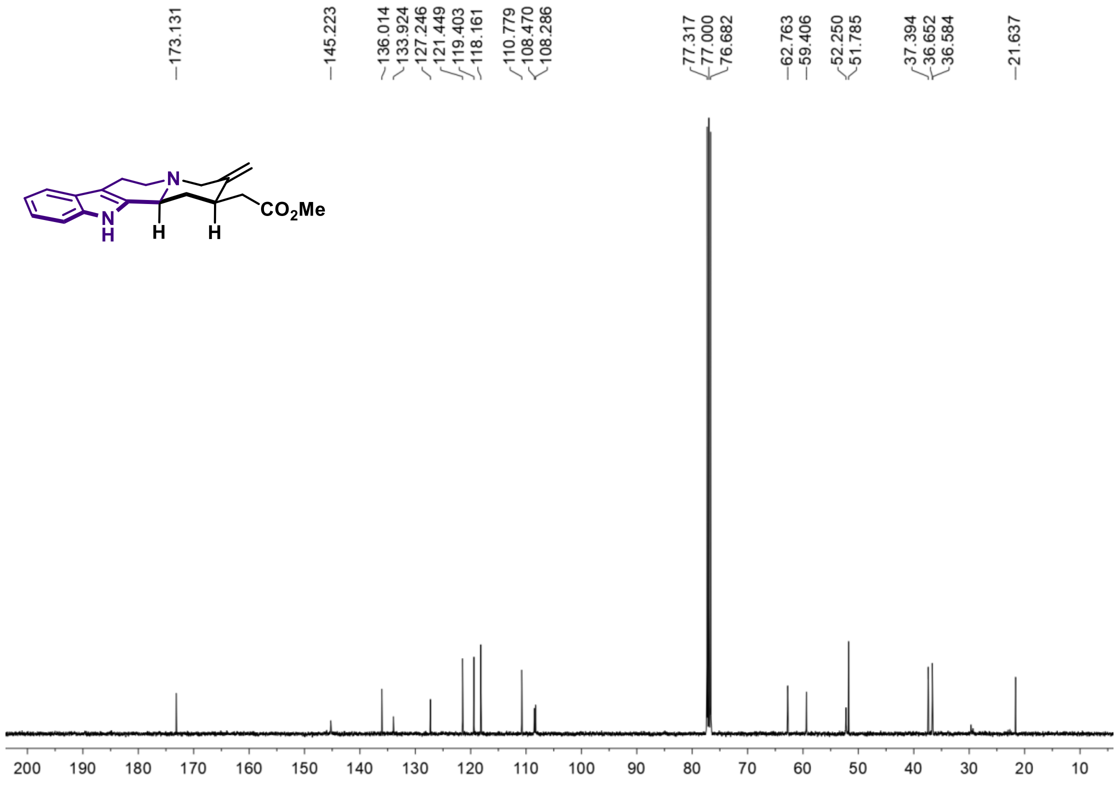

Supplementary Figure 275: ^{1}H NMR of 40 (400 MHz, $CDCl_3$)

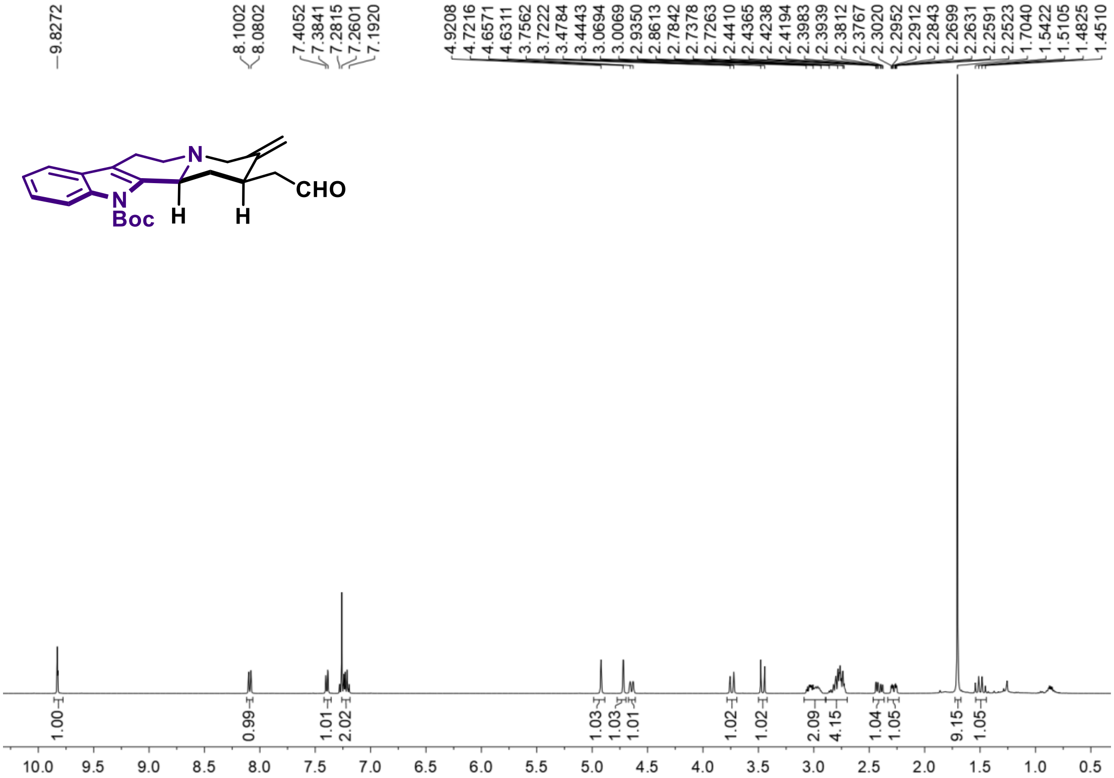

Supplementary Figure 276: ^{13}C NMR of 40 (101 MHz, $CDCl_3$)

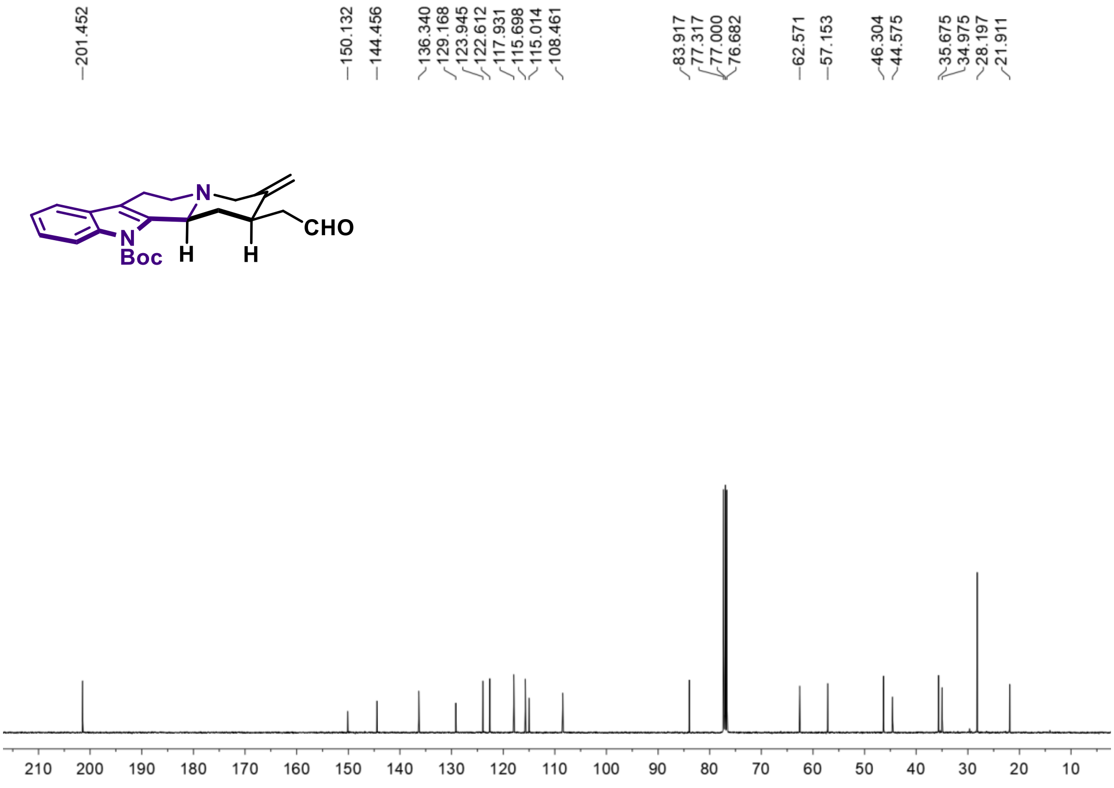

Supplementary Figure 277: ^{1}H NMR of 41 (400 MHz, $CDCl_3$)

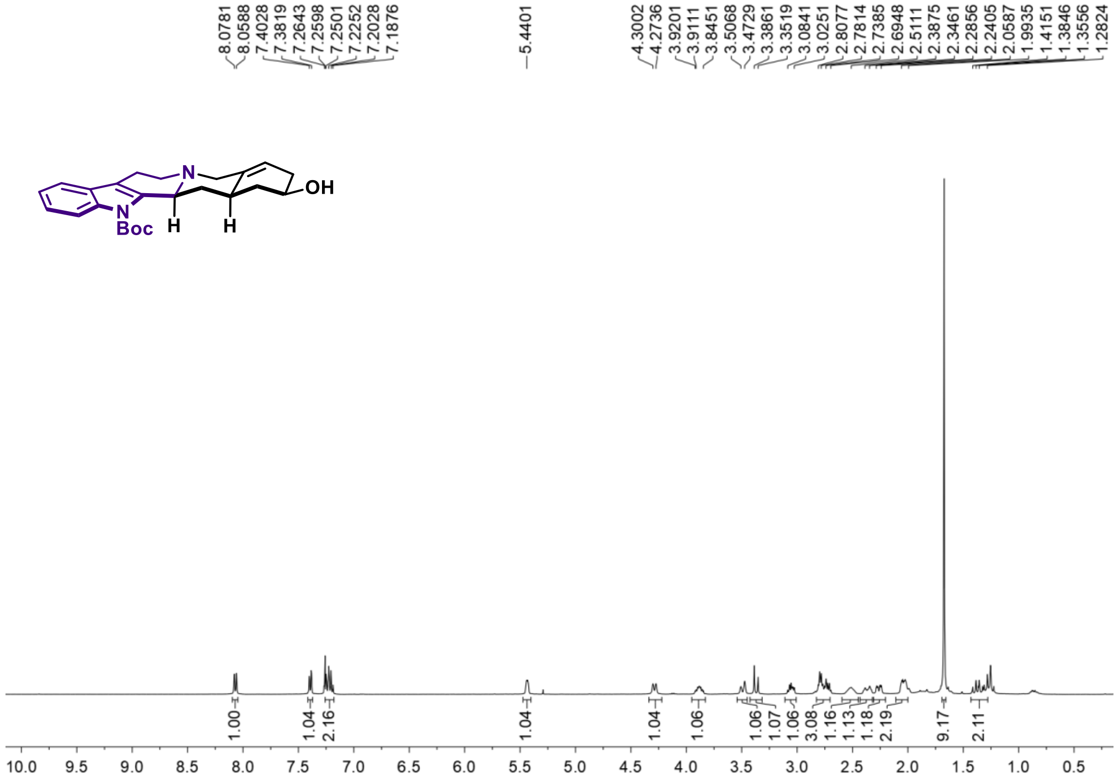

Supplementary Figure 278: ^{13}C NMR of 41 (101 MHz, $CDCl_3$)

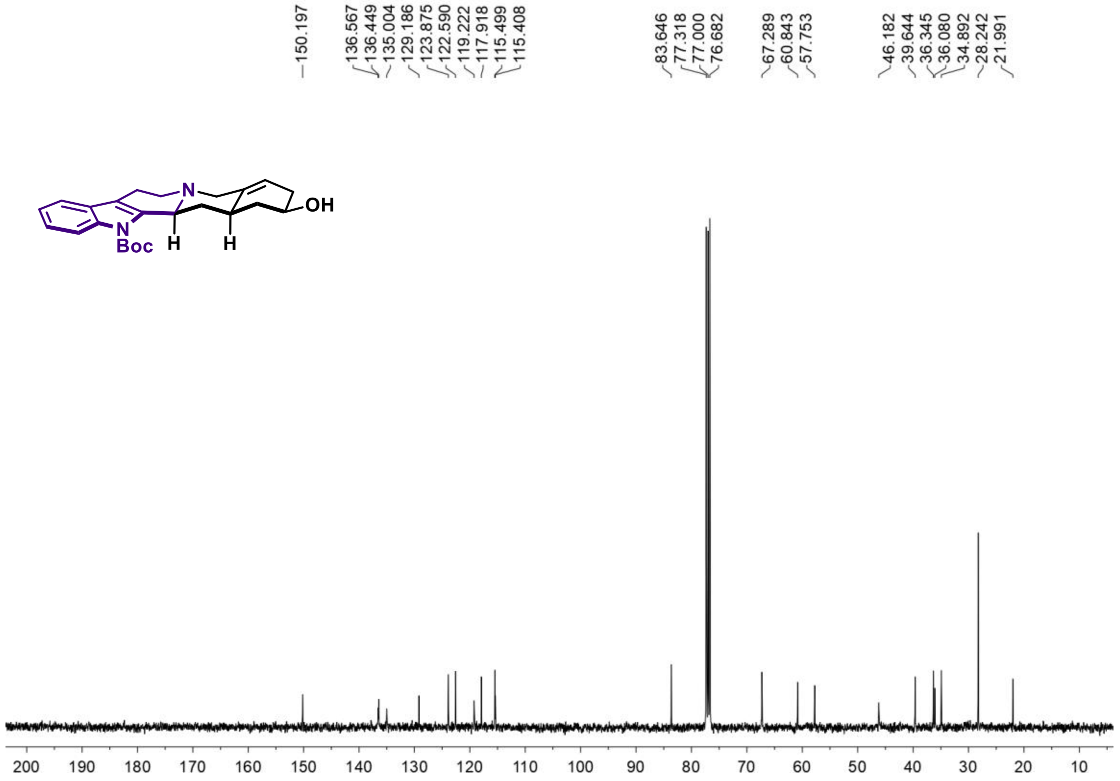

Supplementary Figure 279: ^{1}H NMR of 42 (400 MHz, $CDCl_3$)

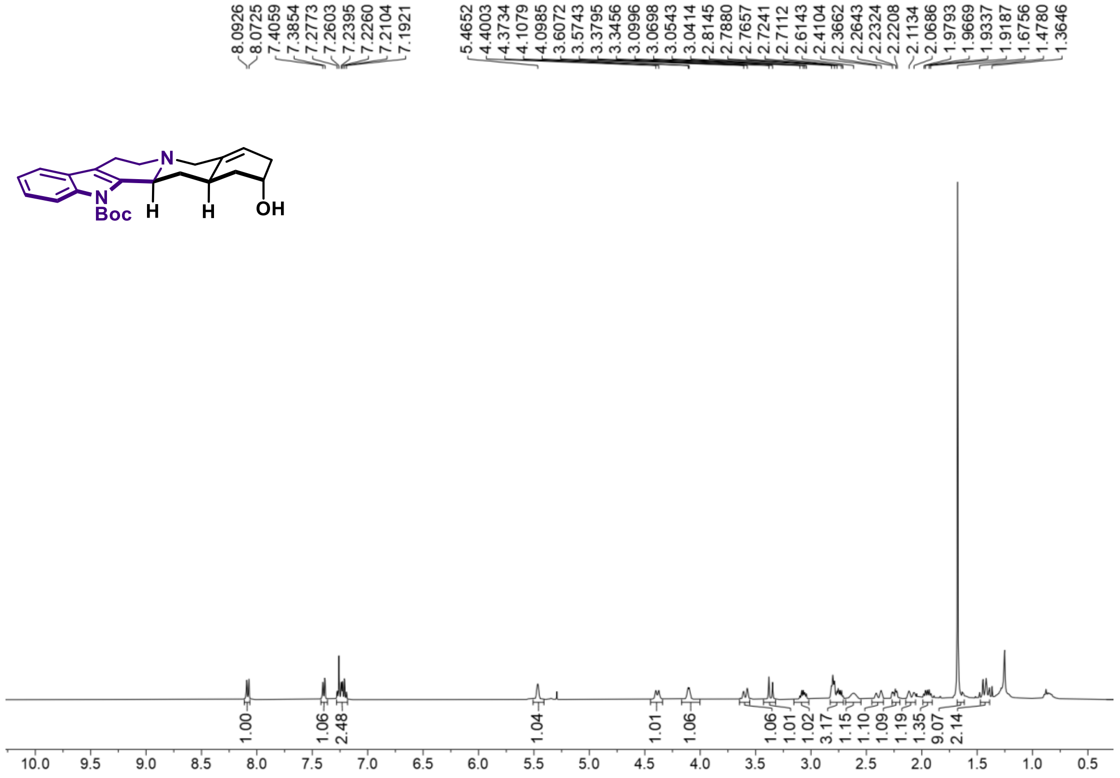

Supplementary Figure 280: ^{13}C NMR of 42 (101 MHz, $CDCl_3$)

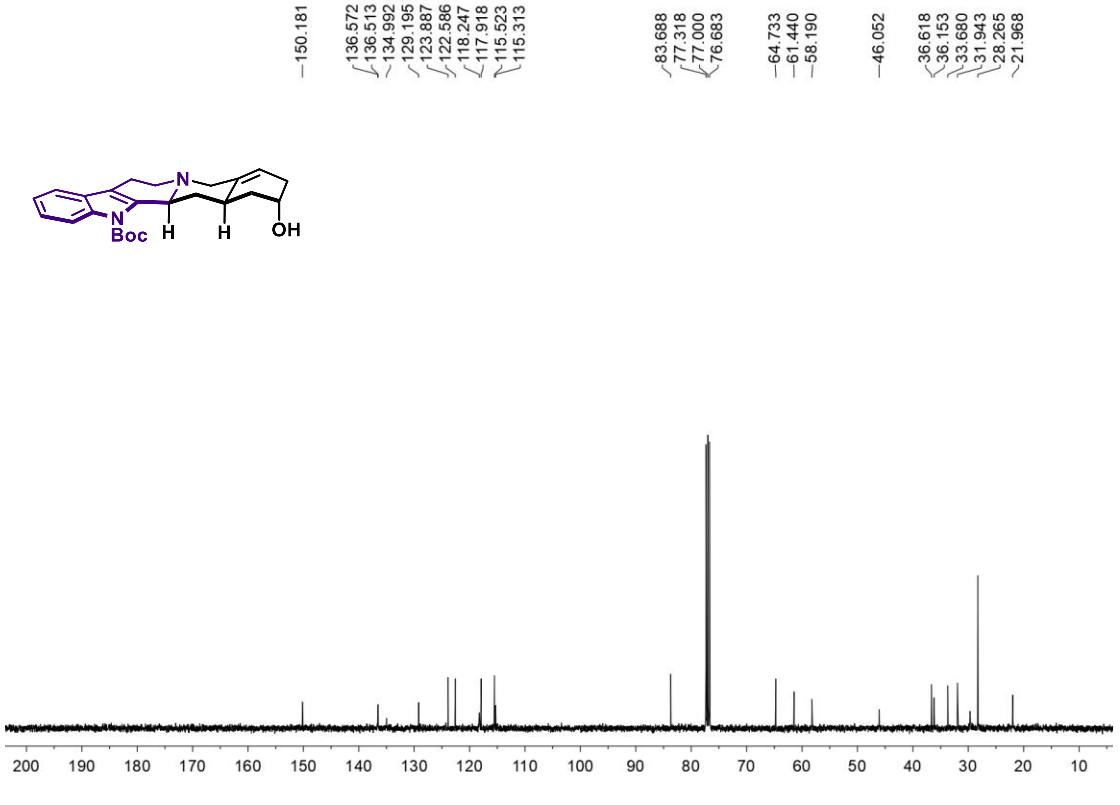

Supplementary Figure 281: ^{1}H NMR of 43 (400 MHz, $CDCl_3$)

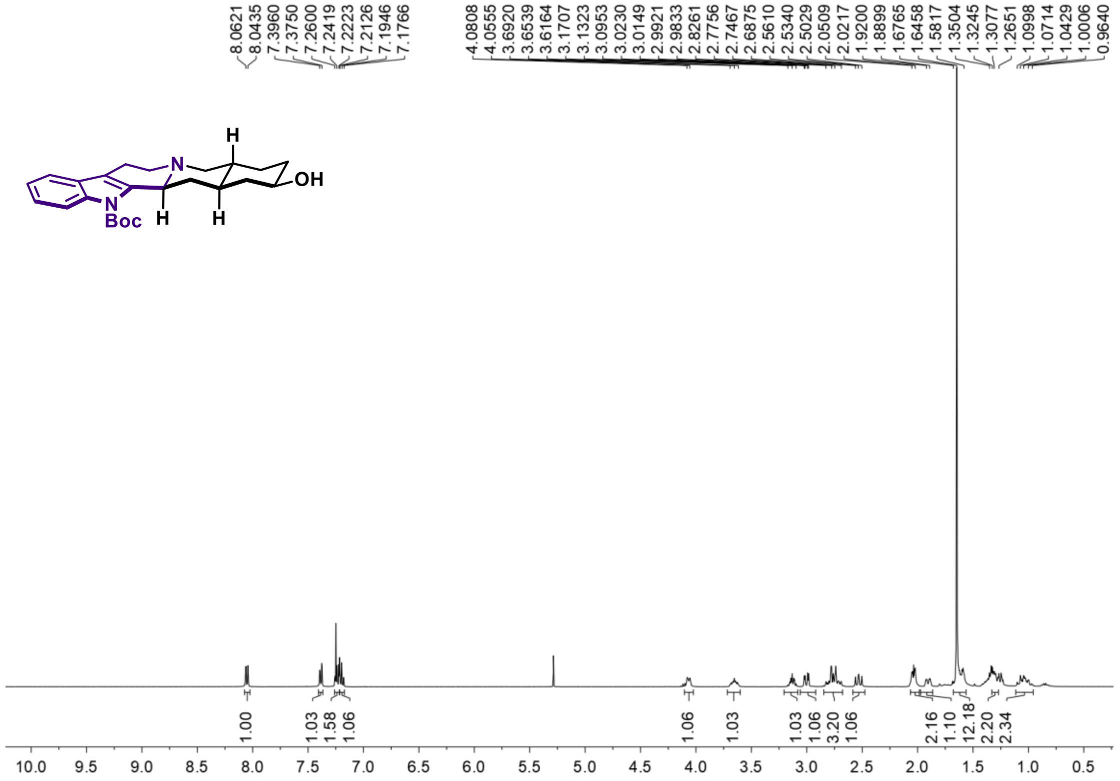

Supplementary Figure 282: ^{13}C NMR of 43 (101 MHz, $CDCl_3$)

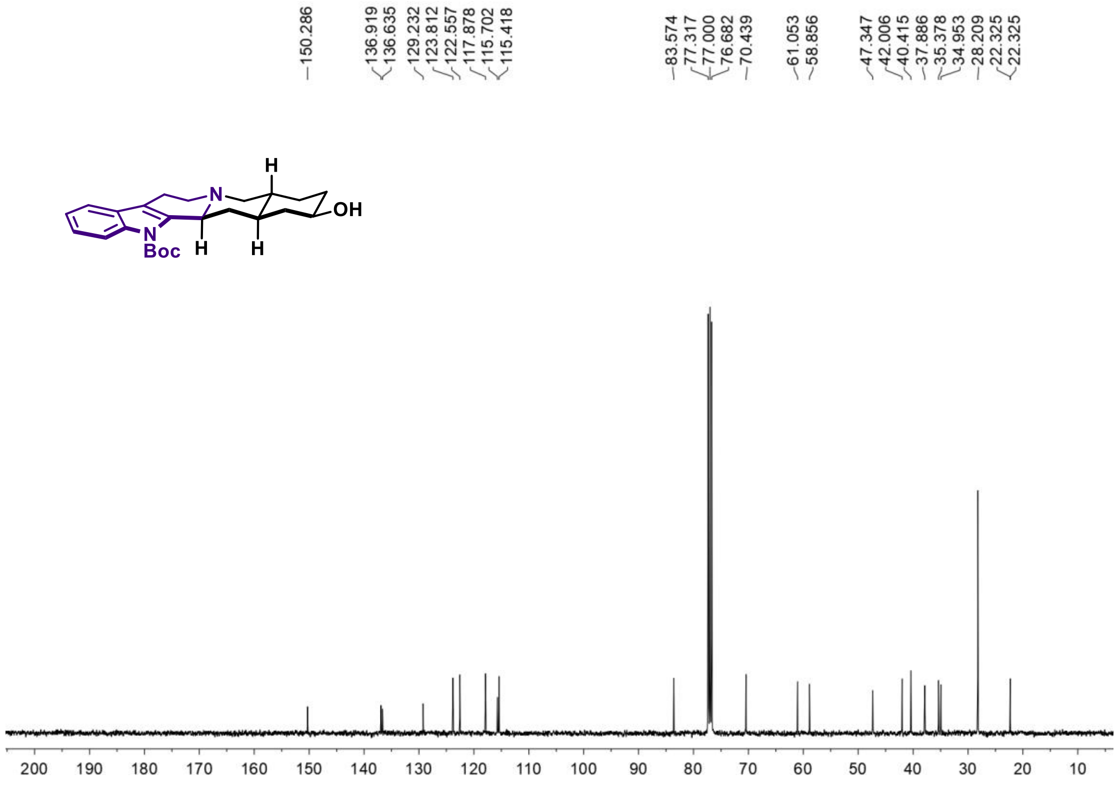

Supplementary Figure 283: ^{1}H NMR of 44 (400 MHz, $CDCl_3$:MeOD = 10:1)

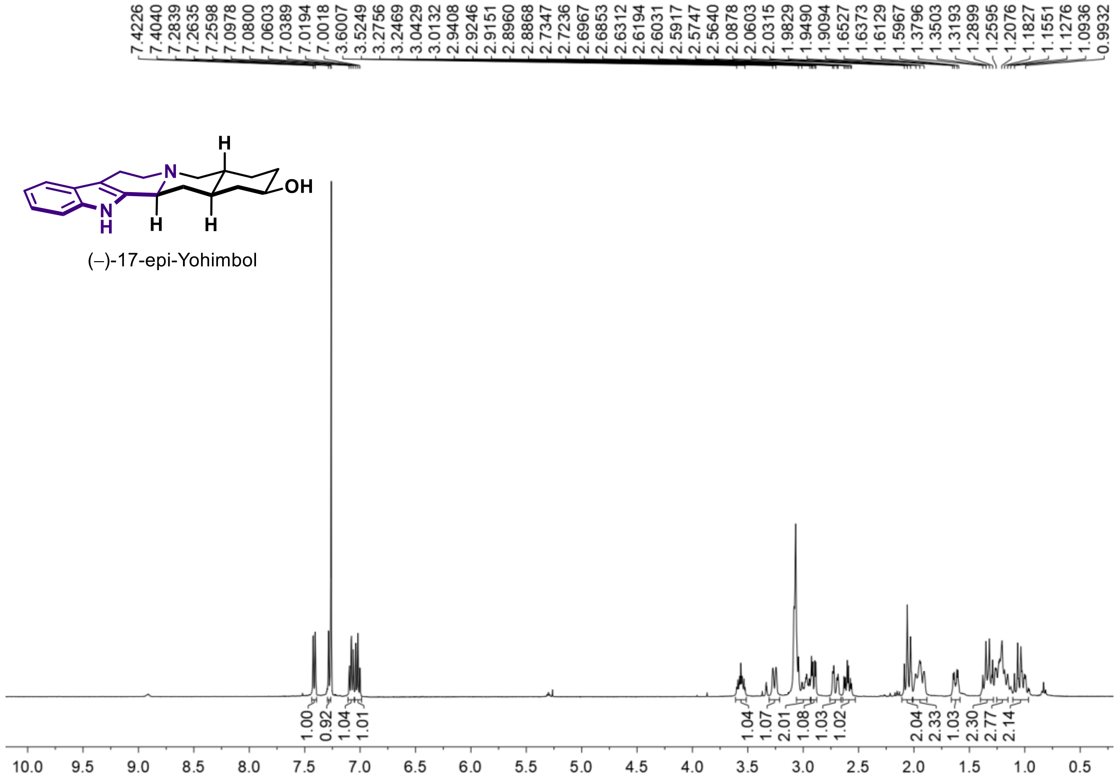

Supplementary Figure 284: ^{13}C NMR of 44 (150 MHz, $CDCl_3$:MeOD = 10:1)

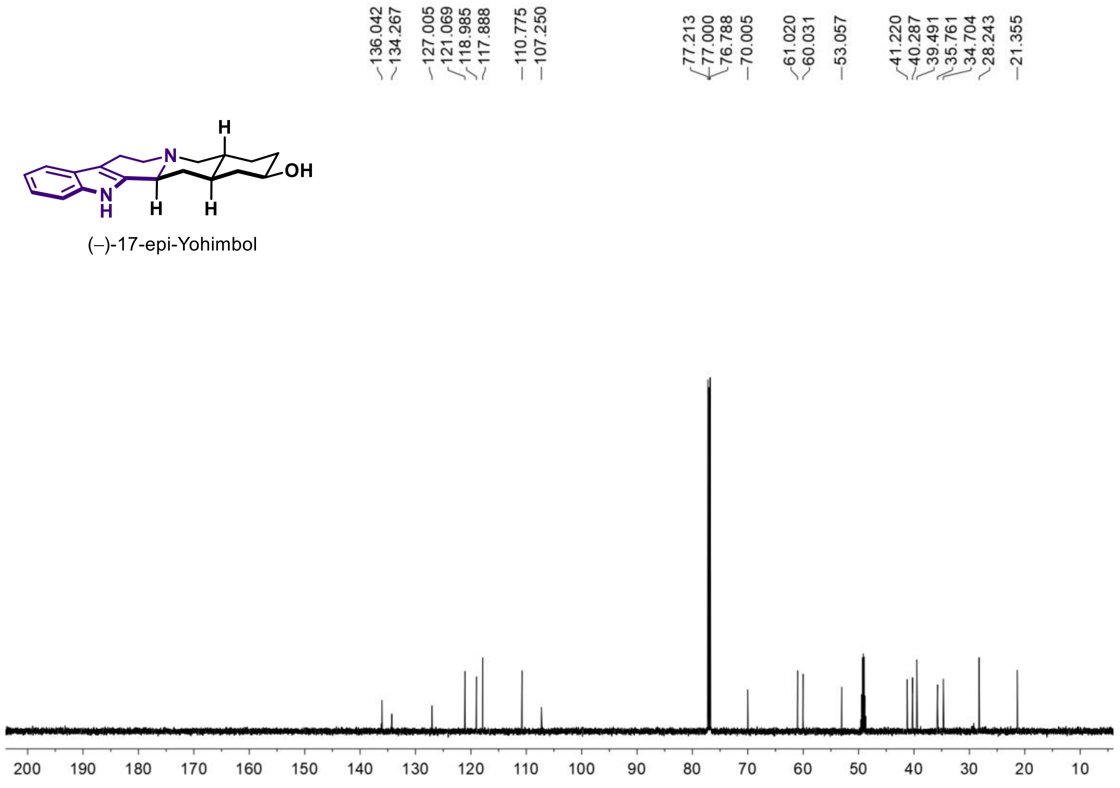

Supplementary Figure 285: ^{1}H NMR of 45 (400 MHz, $CDCl_3$:MeOD = 10:1)

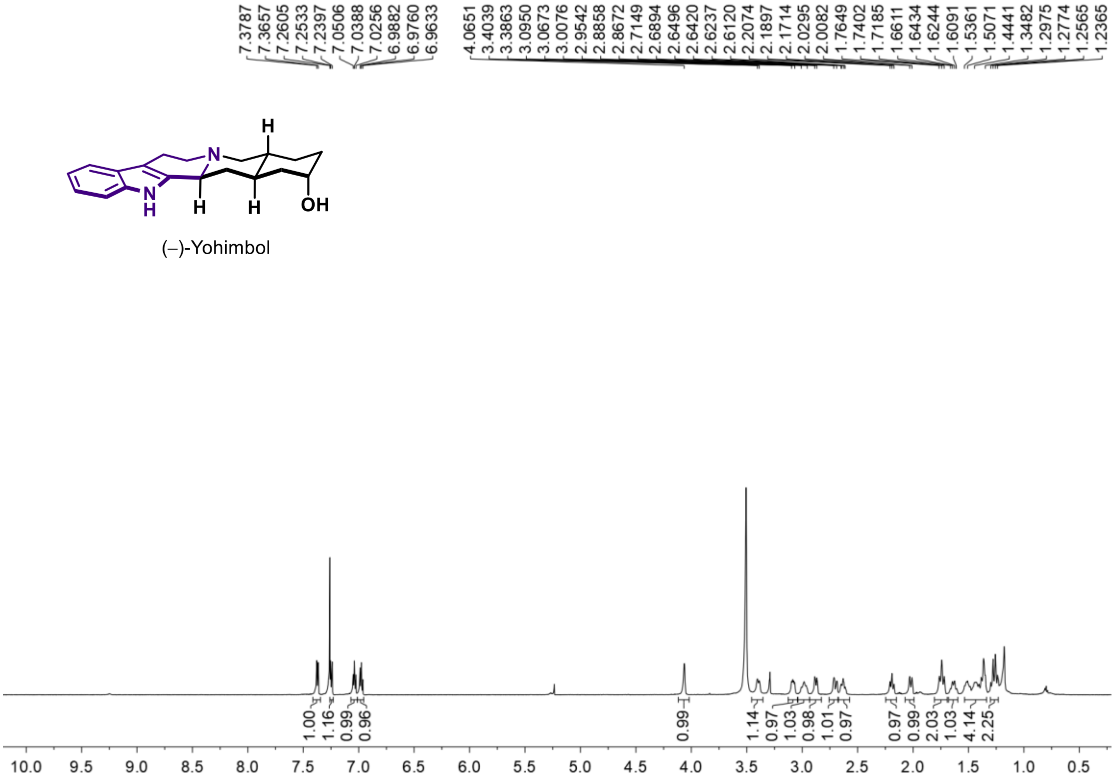

Supplementary Figure 286: ^{13}C NMR of 45 (101 MHz, $CDCl_3$:MeOD = 10:1)

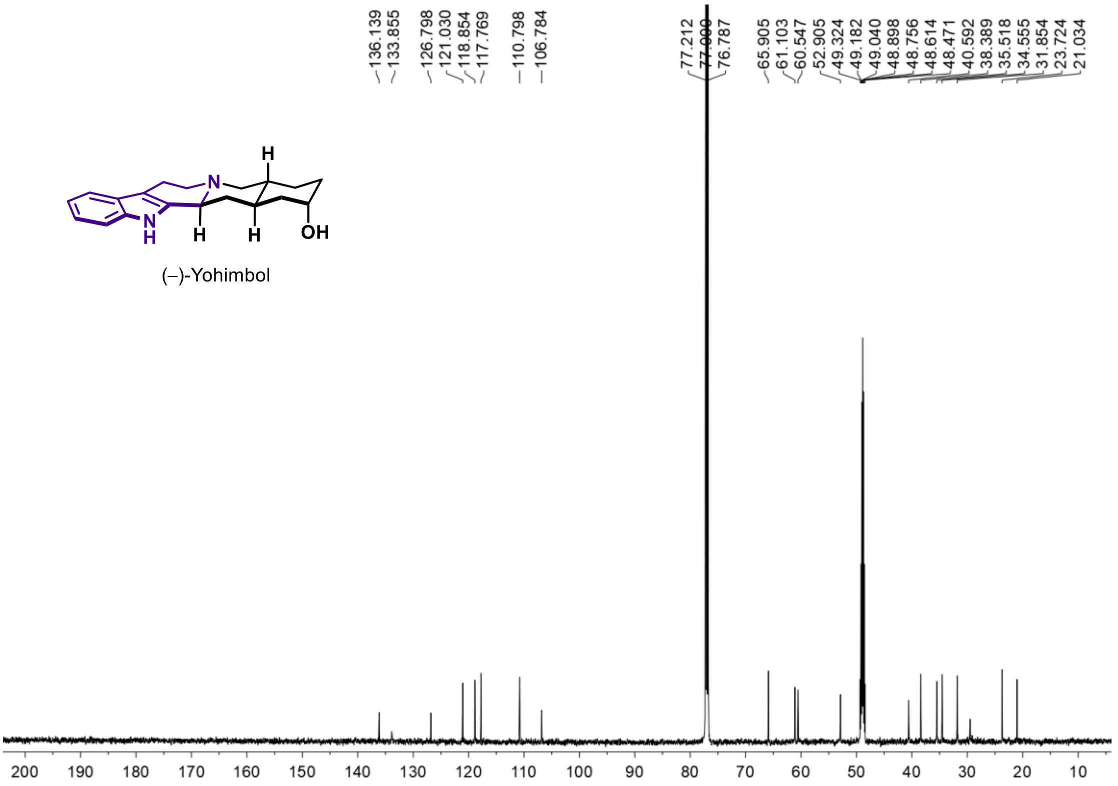

Supplementary Figure 287: ^{1}H NMR of 46 (400 MHz, CDCl$_3$)

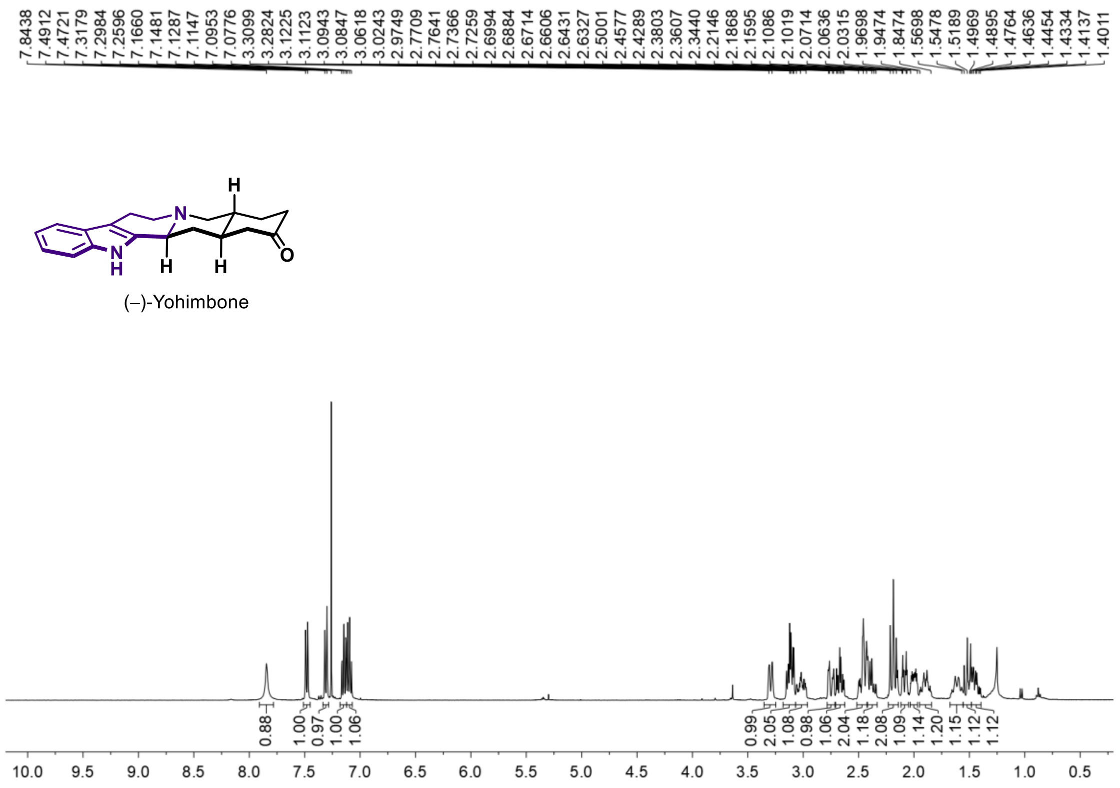

Supplementary Figure 288: ^{13}C NMR of 46 (101 MHz, CDCl$_3$)

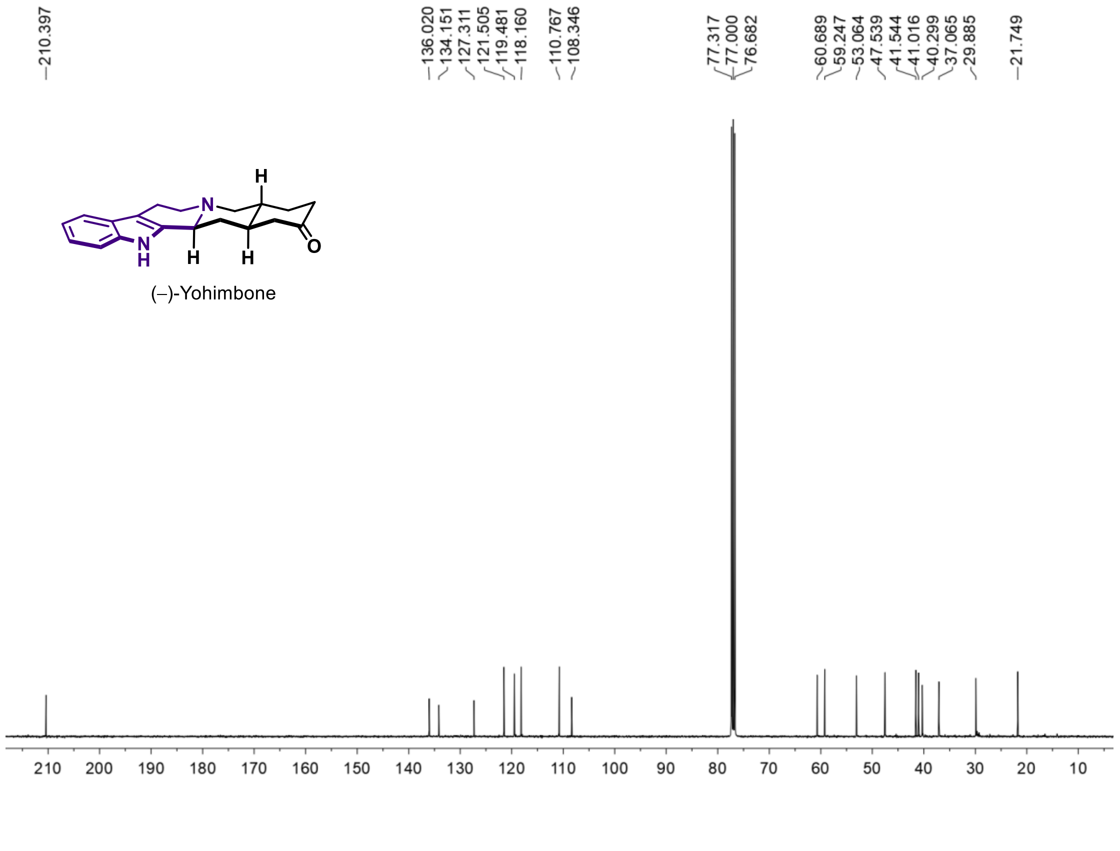

Supplementary Figure 289: ^{1}H NMR of 47 (400 MHz, $CDCl_3$)

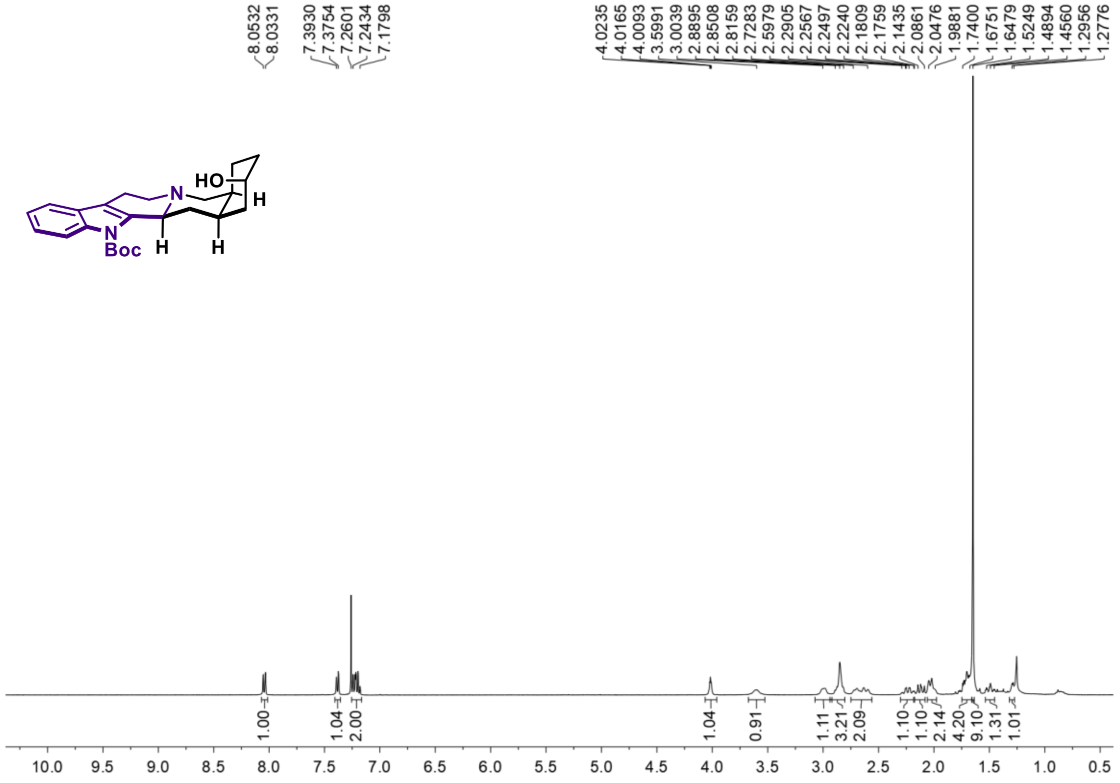

Supplementary Figure 290: ^{13}C NMR of 47 (101 MHz, $CDCl_3$)

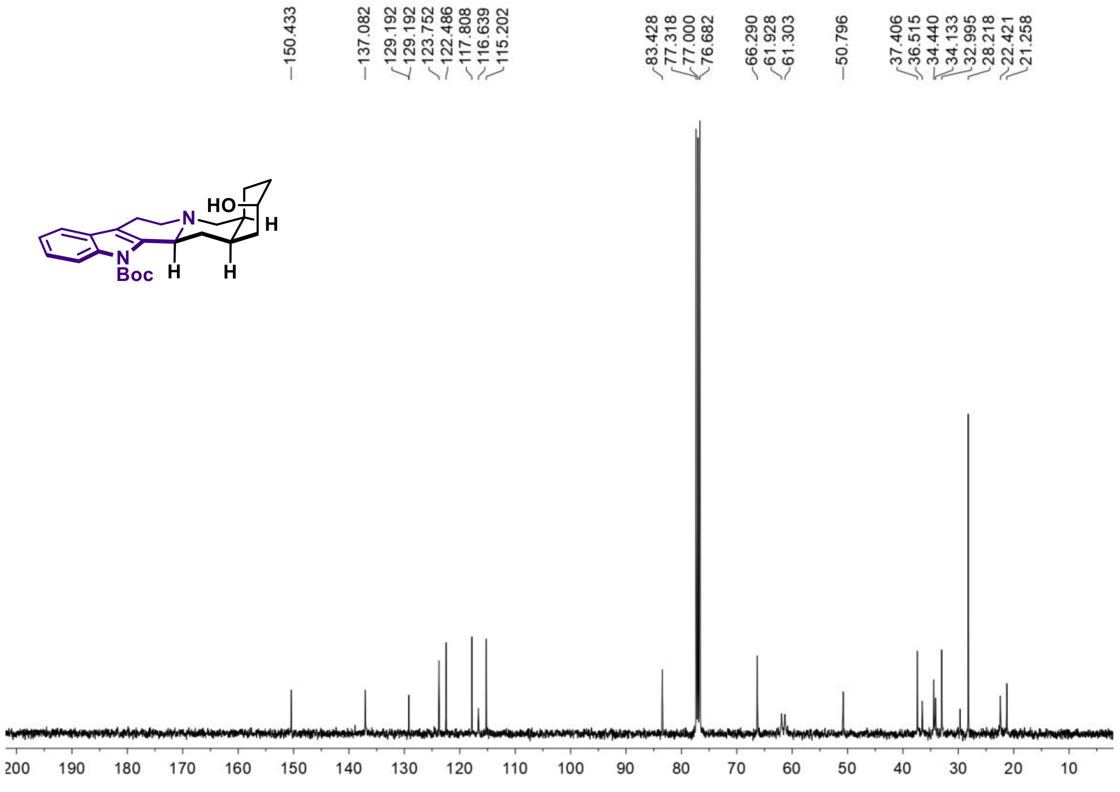

Supplementary Figure 291: ^{1}H NMR of 48 (400 MHz, CDCl$_3$)

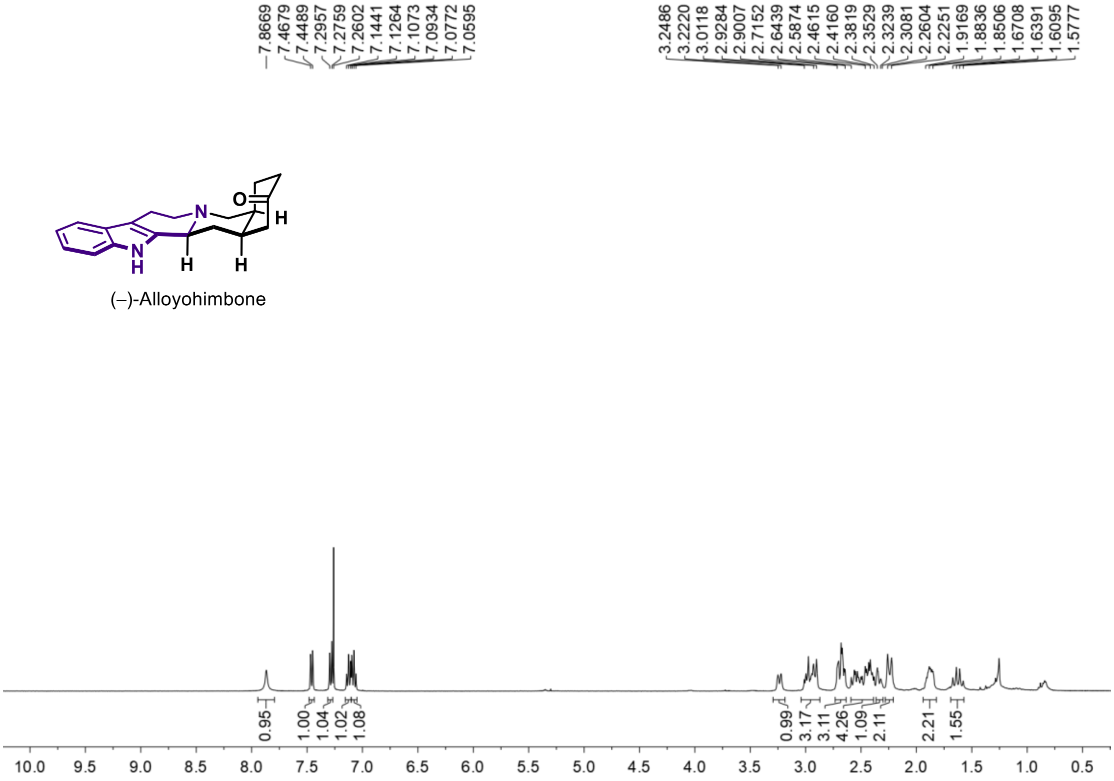

Supplementary Figure 292: ^{13}C NMR of 48 (101 MHz, CDCl$_3$)

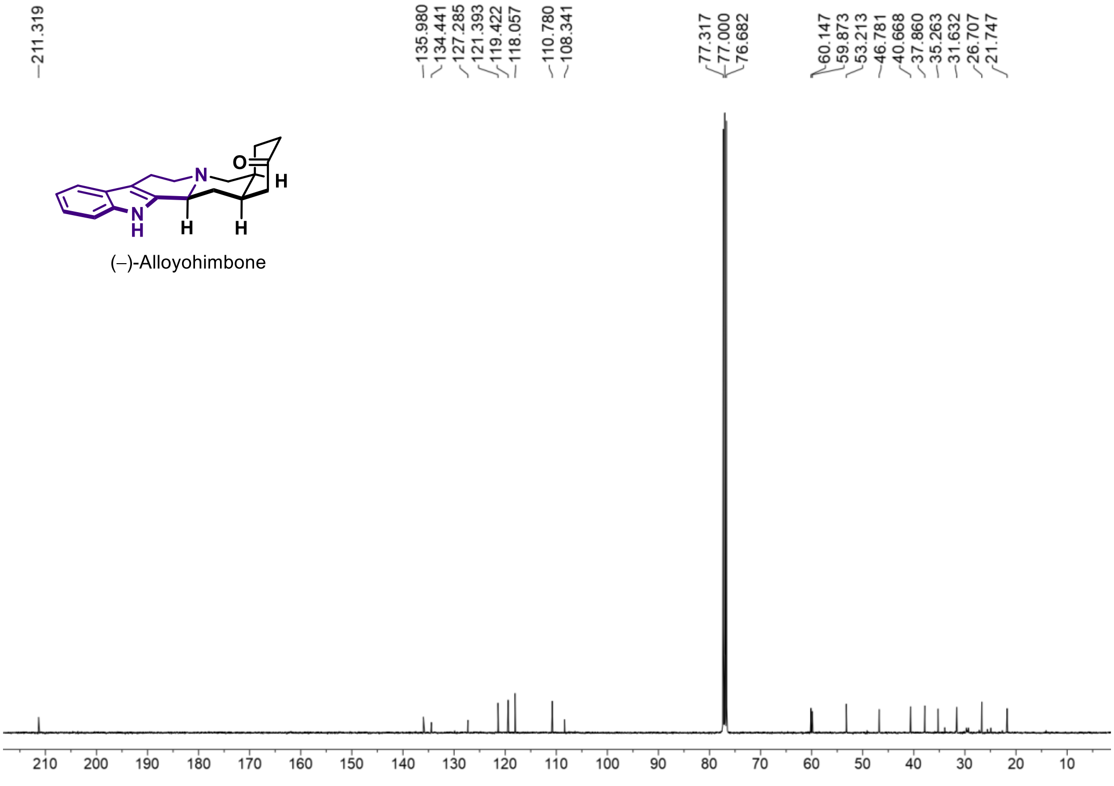

Supplementary Figure 293: ^{1}H NMR of 49 (400 MHz, $CDCl_3$)

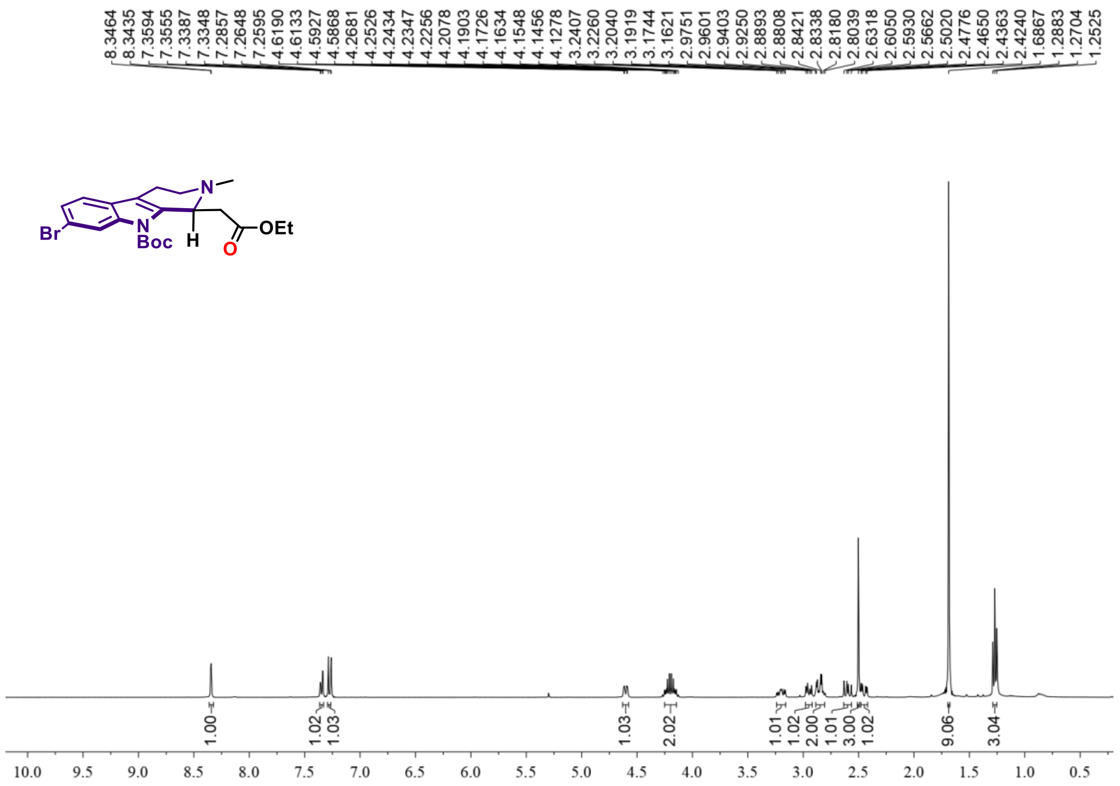

Supplementary Figure 294: ^{13}C NMR of 49 (101 MHz, $CDCl_3$)

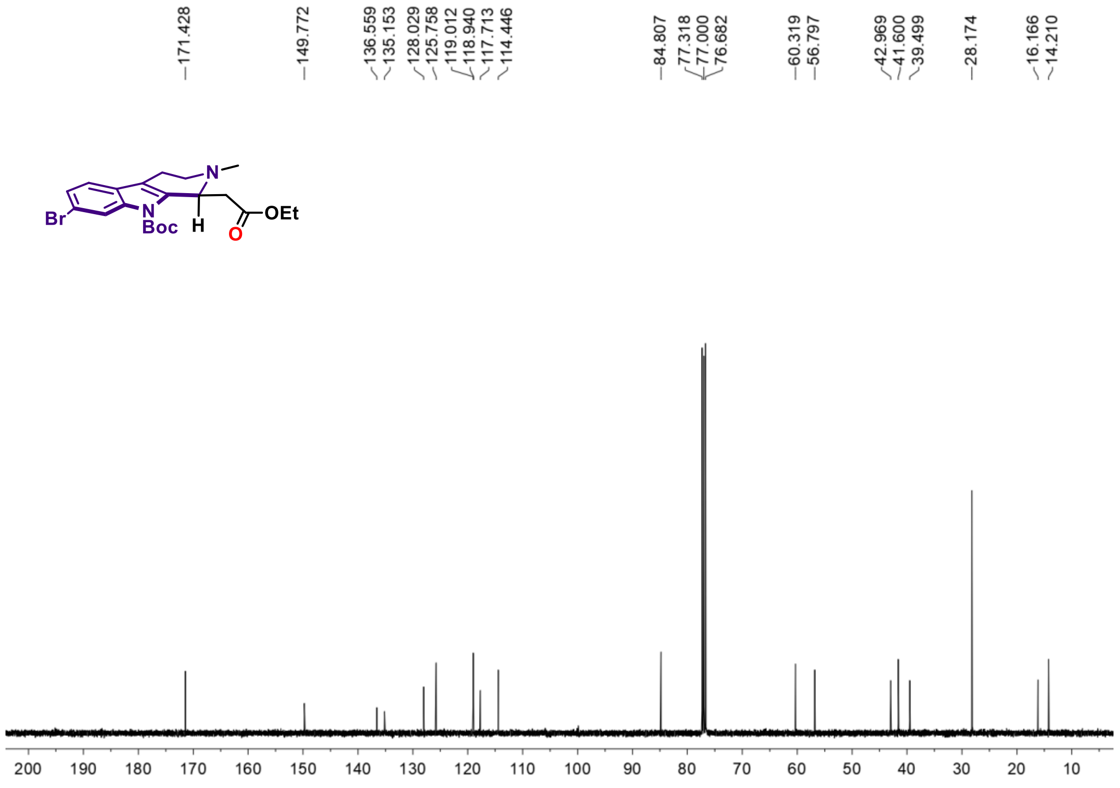

Supplementary Figure 295: ^{1}H NMR of 50 (400 MHz, CDCl$_3$)

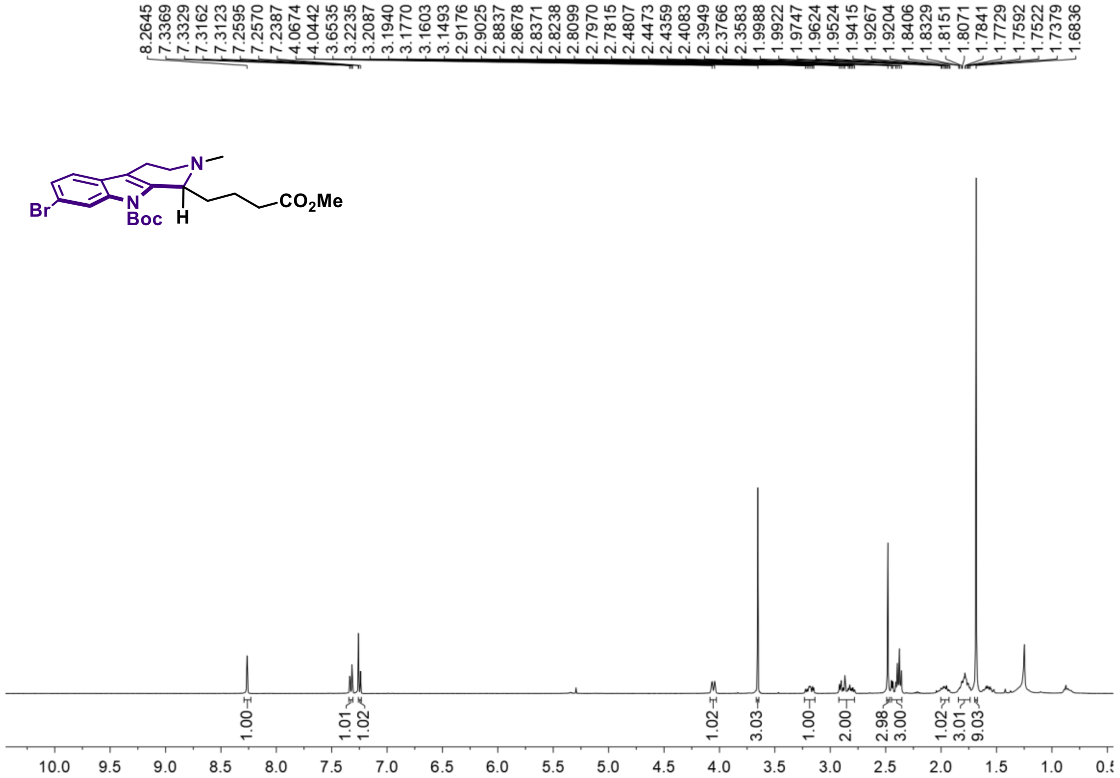

Supplementary Figure 296: ^{13}C NMR of 50 (101 MHz, CDCl$_3$)

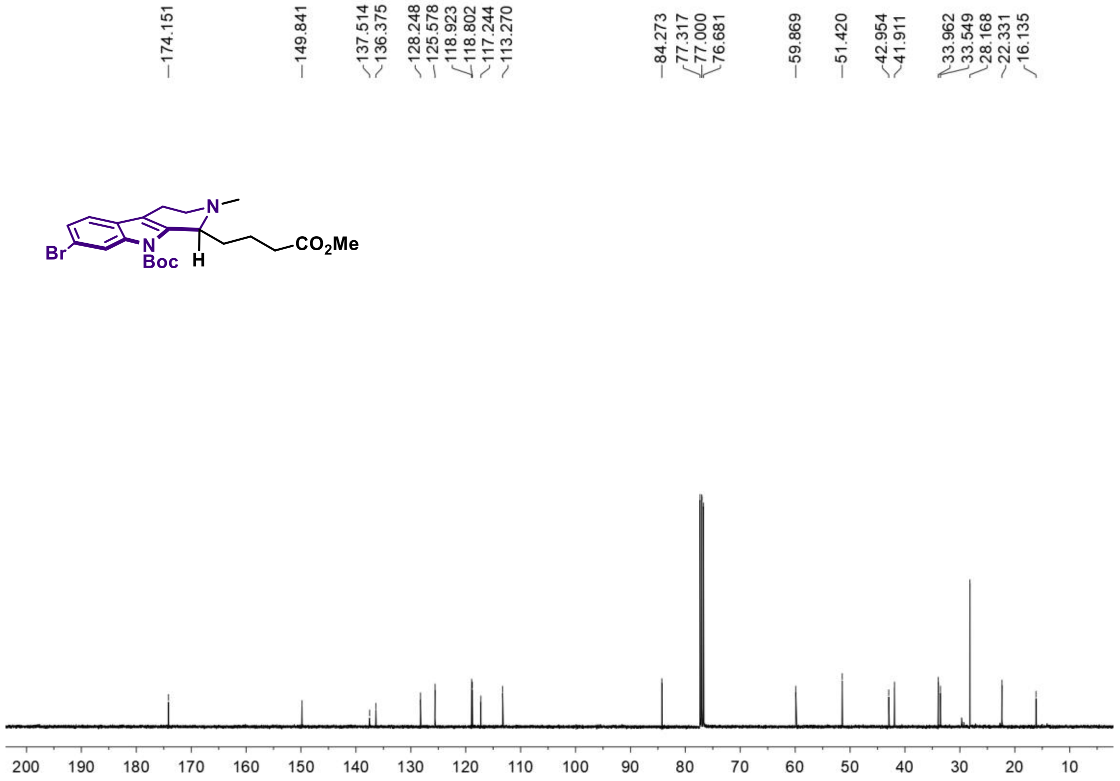

Supplementary Figure 297: ^{1}H NMR of 51 (400 MHz, $CDCl_3$)

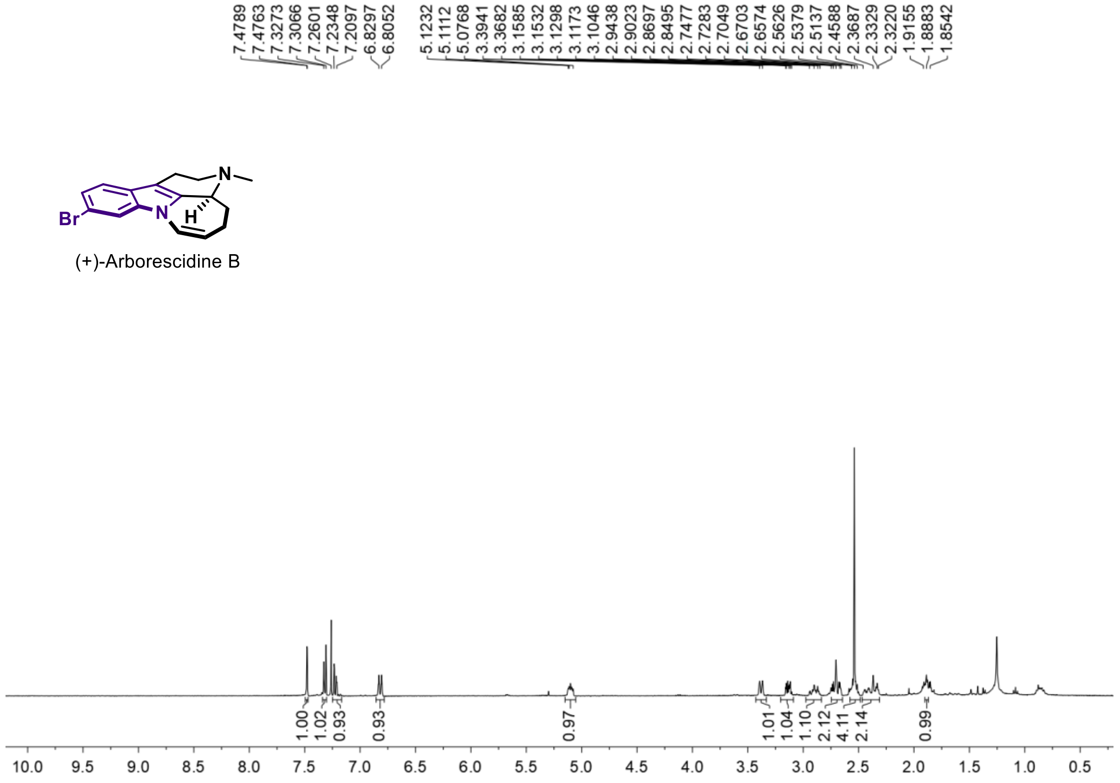

Supplementary Figure 298: ^{13}C NMR of 51 (101 MHz, $CDCl_3$)

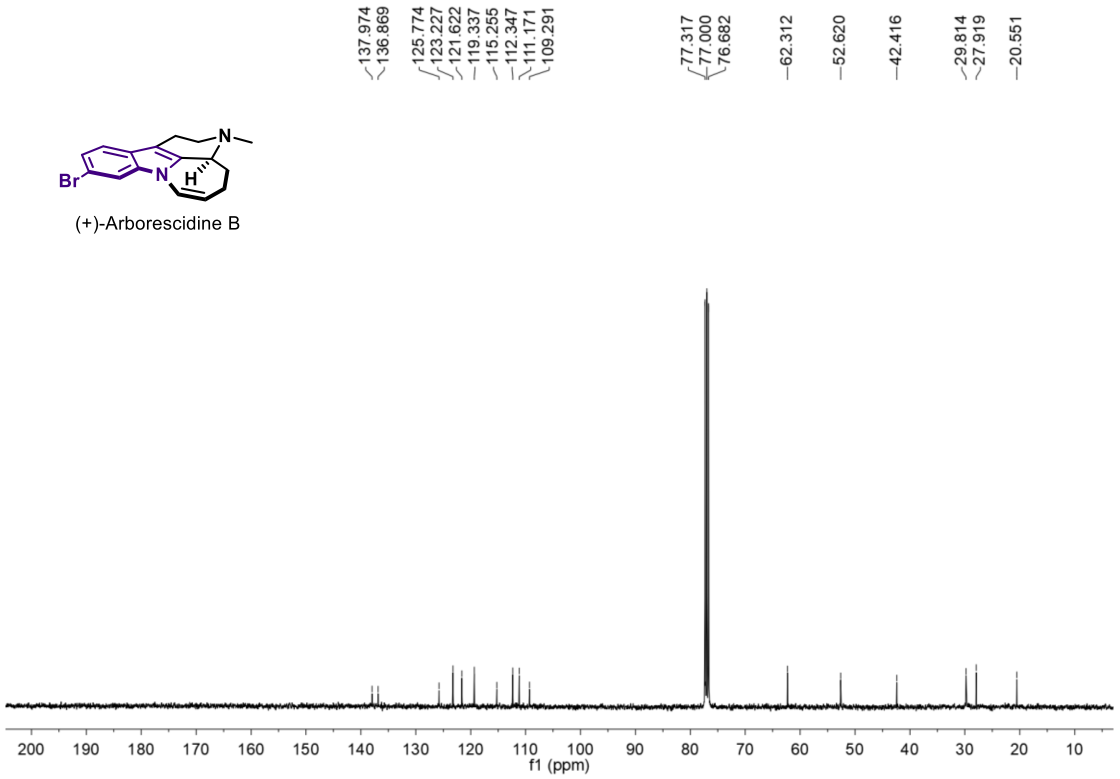

Supplementary Figure 299: ^{1}H NMR of 52 (400 MHz, $CDCl_3$)

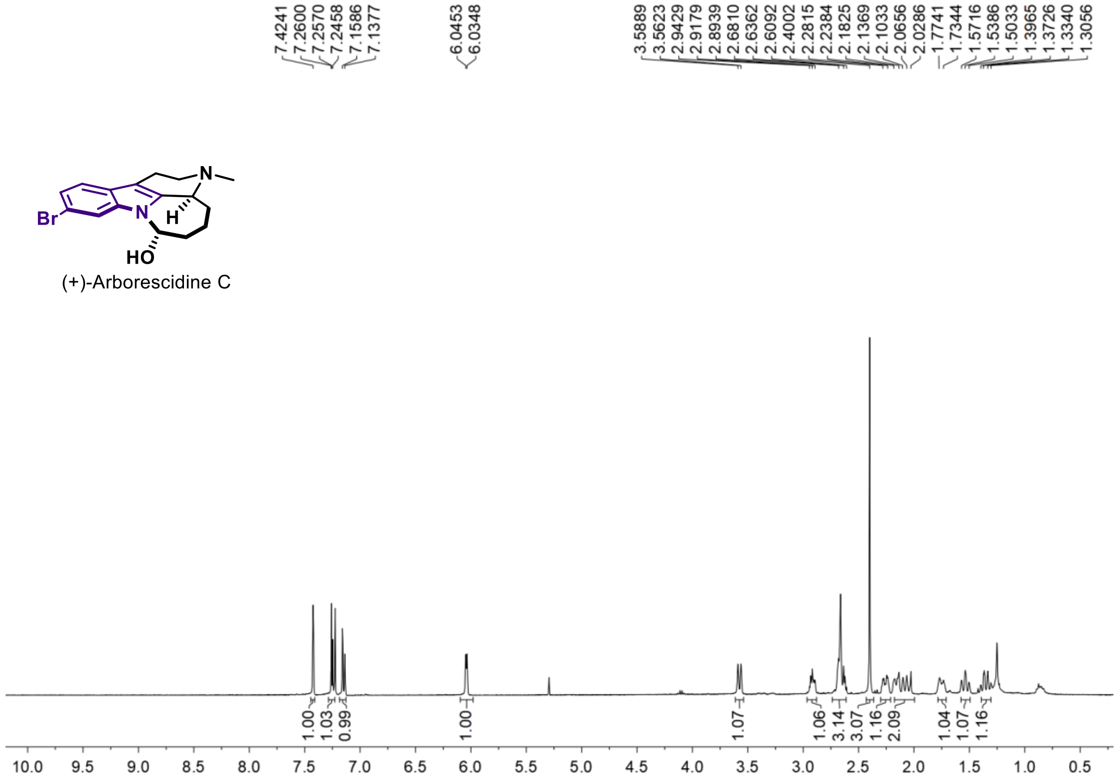

Supplementary Figure 300: ^{13}C NMR of 52 (101 MHz, $CDCl_3$)

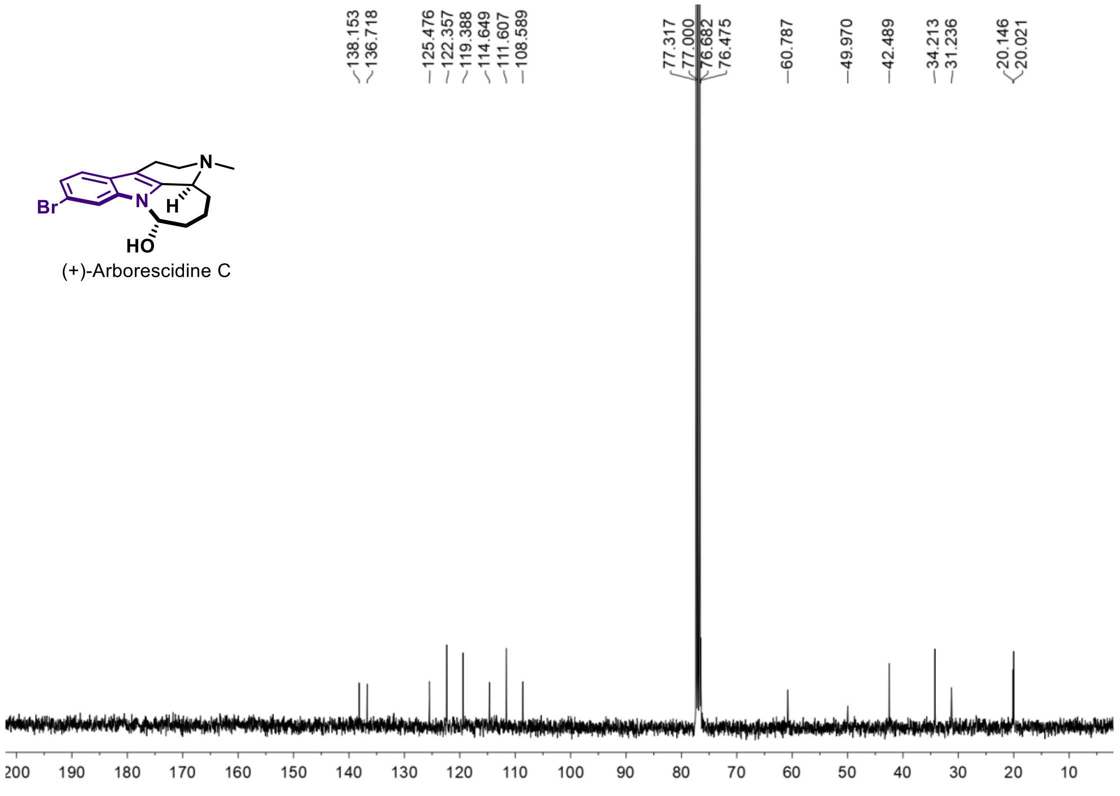

Supplementary Figure 301: ^{1}H NMR of 53 (400 MHz, $CDCl_3$)

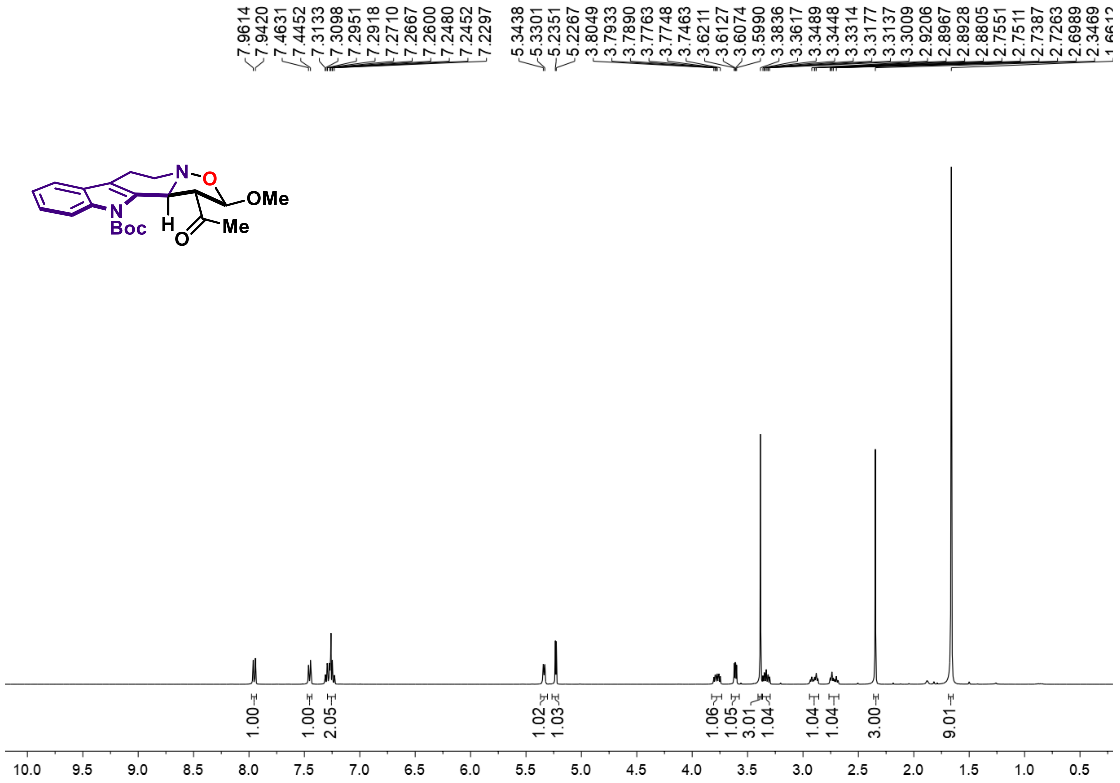

Supplementary Figure 302: ^{13}C NMR of 53 (101 MHz, $CDCl_3$)

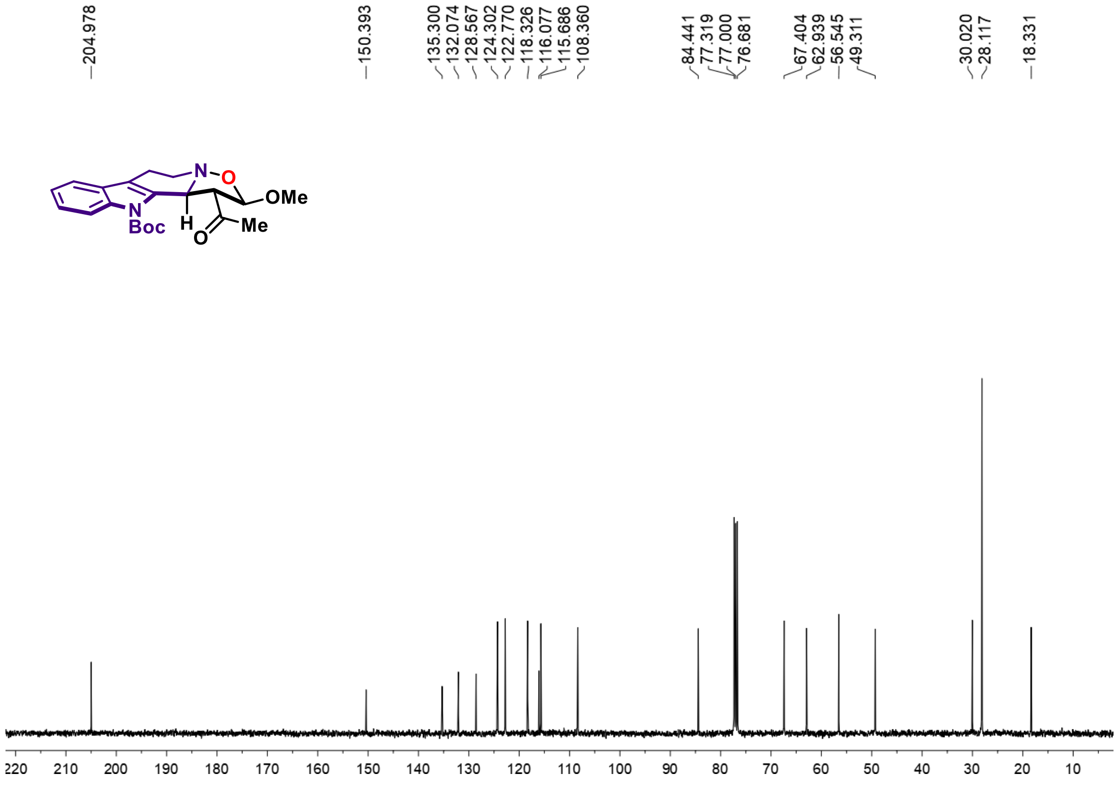

Supplementary Figure 303: ^{1}H NMR of 54 (400 MHz, $CDCl_3$)

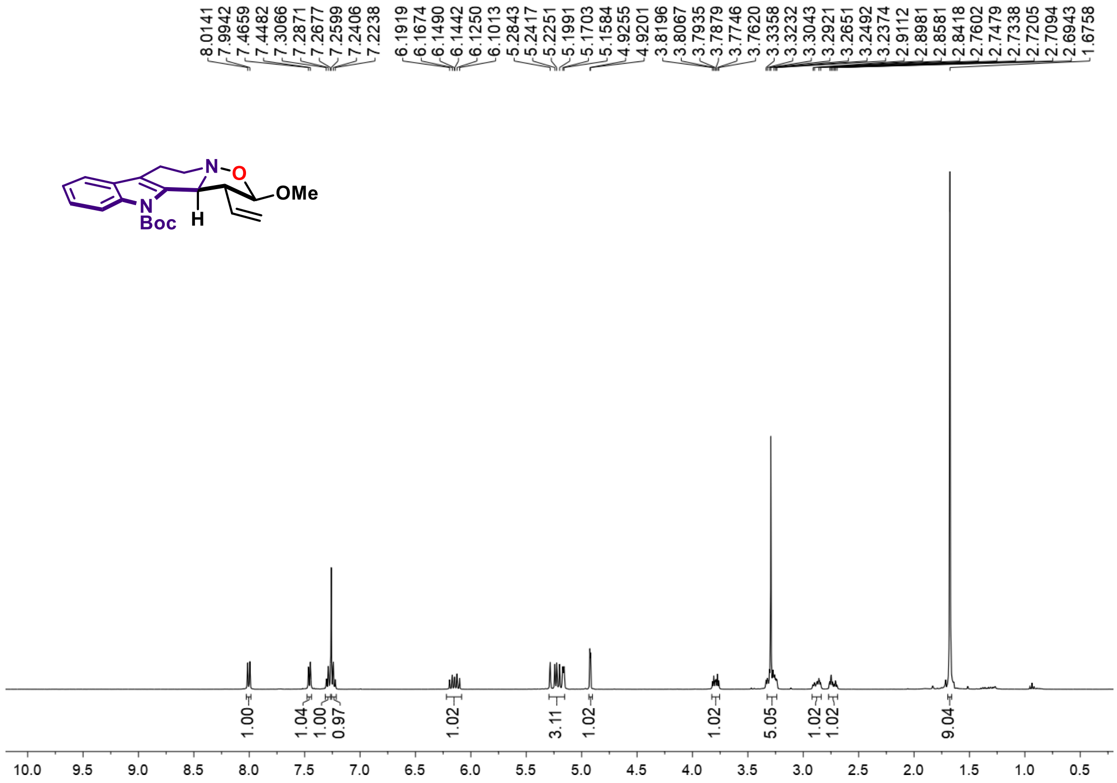

Supplementary Figure 304: ^{13}C NMR of 54 (101 MHz, $CDCl_3$)

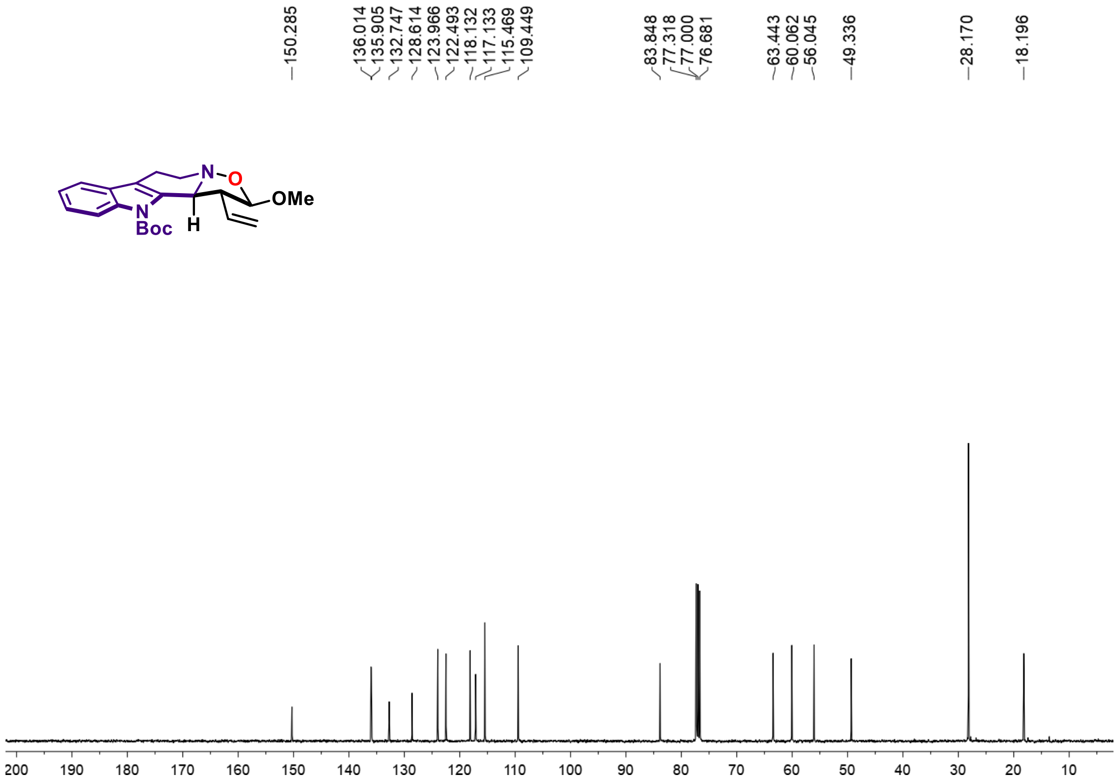

Supplementary Figure 305: ^{1}H NMR of 55 (400 MHz, $CDCl_3$)

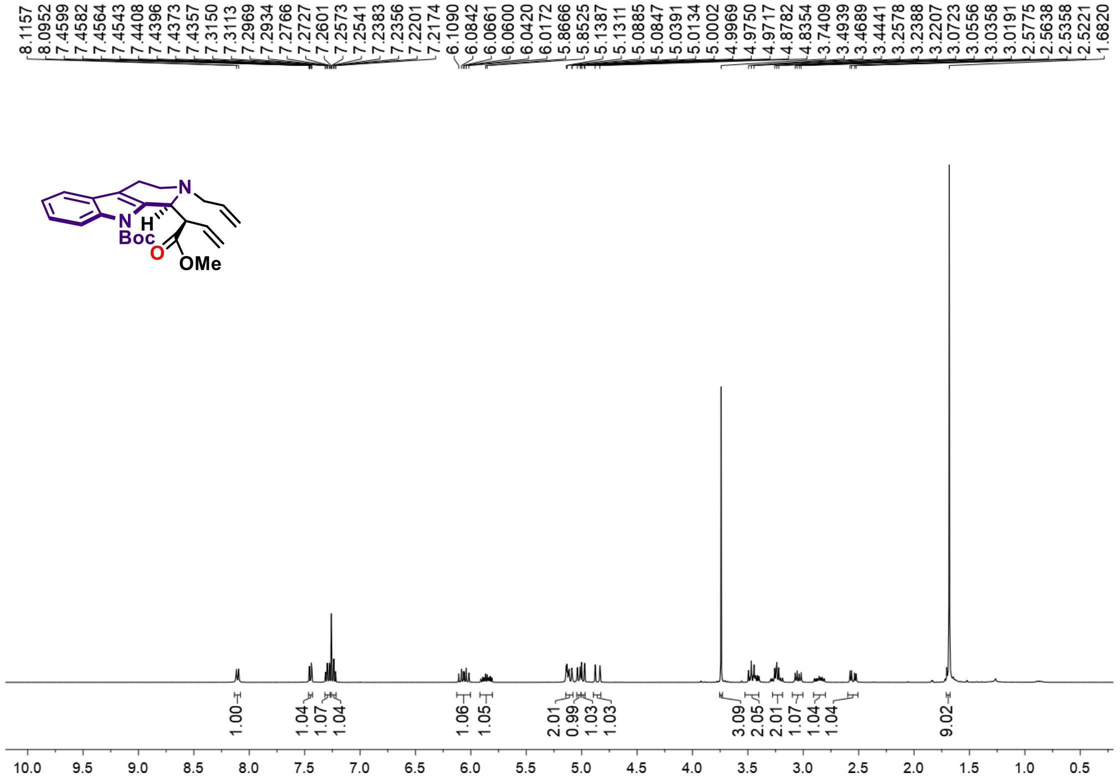

Supplementary Figure 306: ^{13}C NMR of 55 (101 MHz, $CDCl_3$)

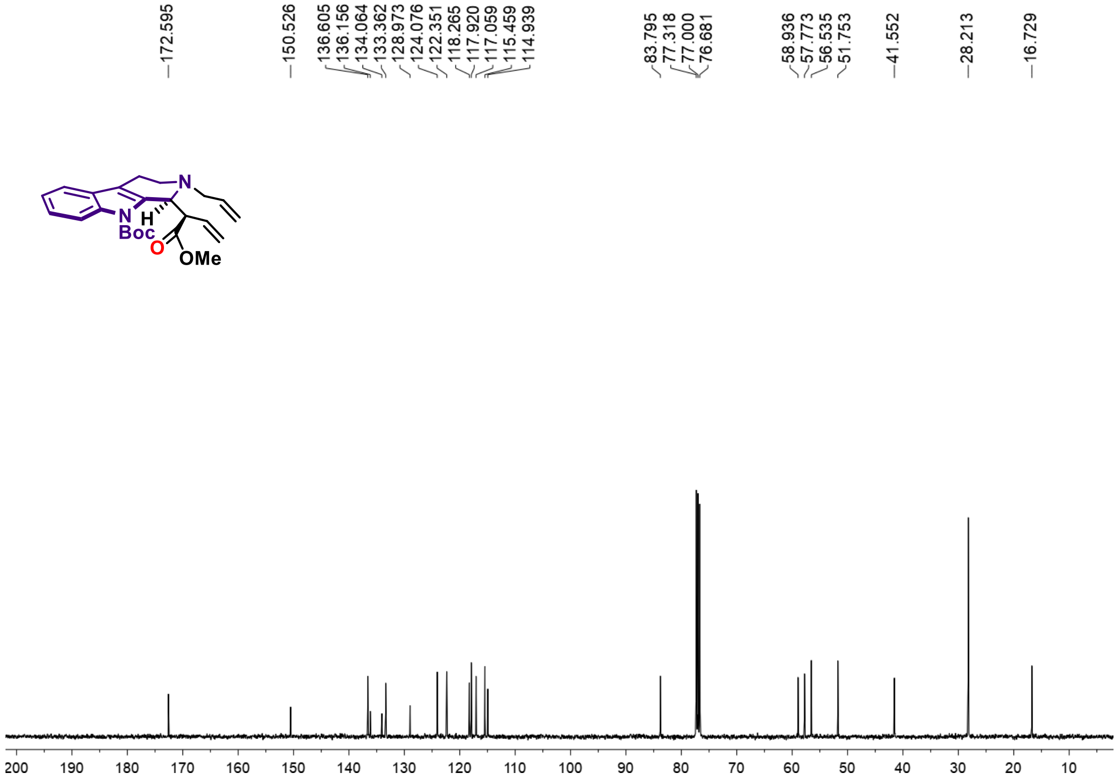

Supplementary Figure 307: ^{1}H NMR of 56 (400 MHz, $CDCl_3$)

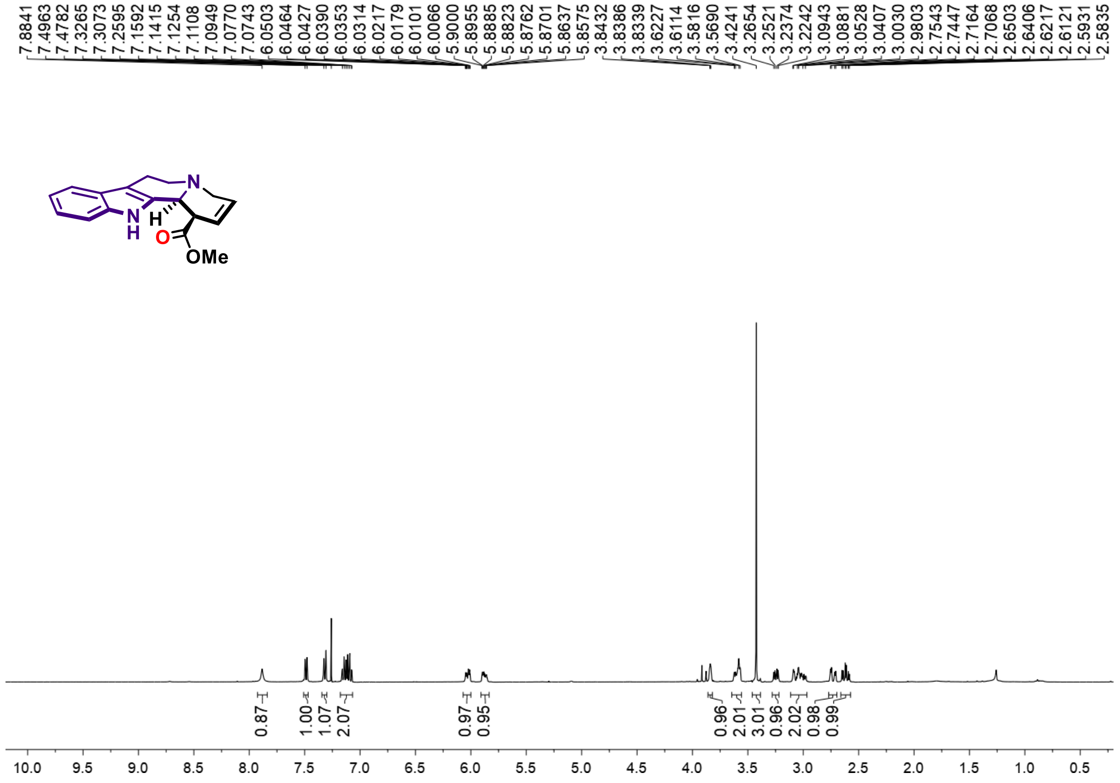

Supplementary Figure 308: ^{13}C NMR of 56 (101 MHz, $CDCl_3$)

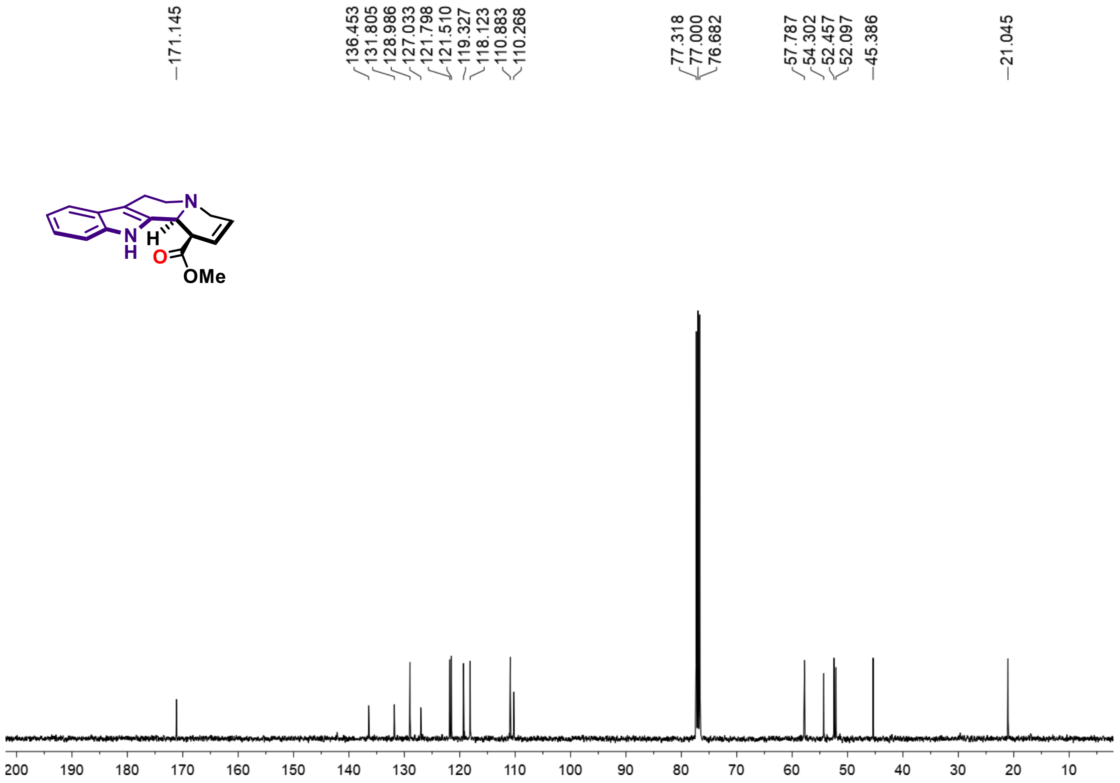

Supplementary Figure 309: ^{1}H NMR of 57 (400 MHz, $CDCl_3$)

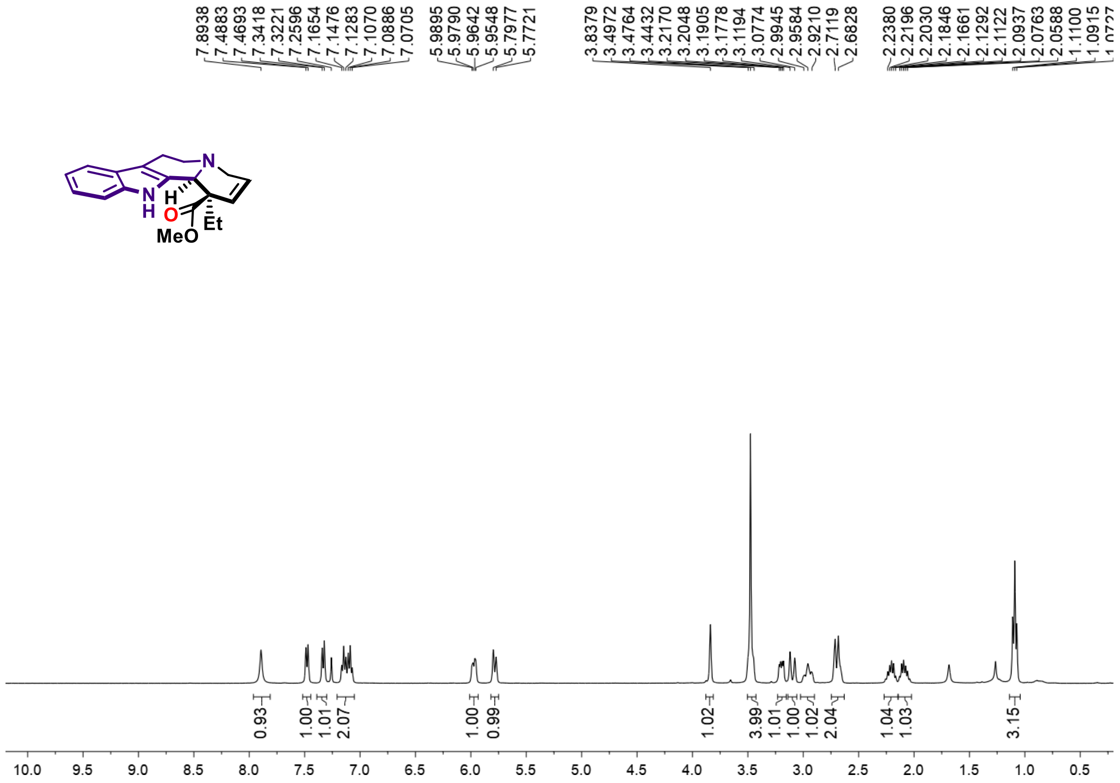

Supplementary Figure 310: ^{13}C NMR of 57 (101 MHz, $CDCl_3$)

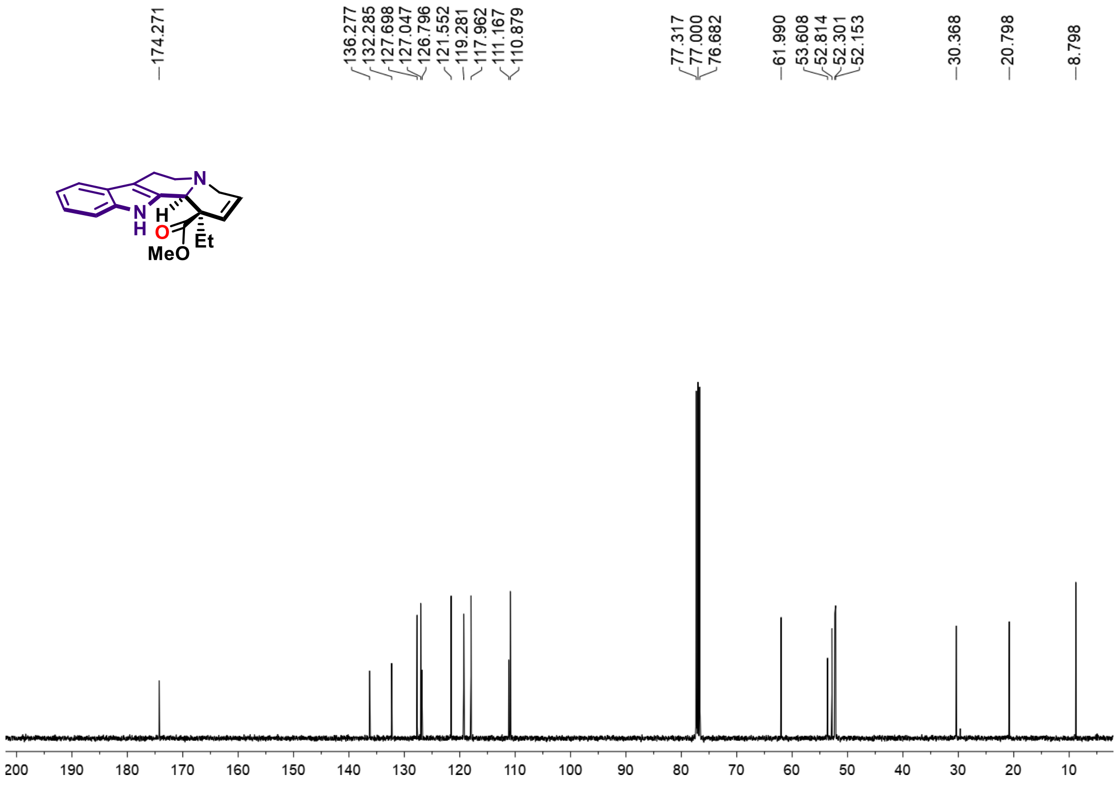

Supplementary Figure 311: ^{1}H NMR of 58 (400 MHz, $CDCl_3$)

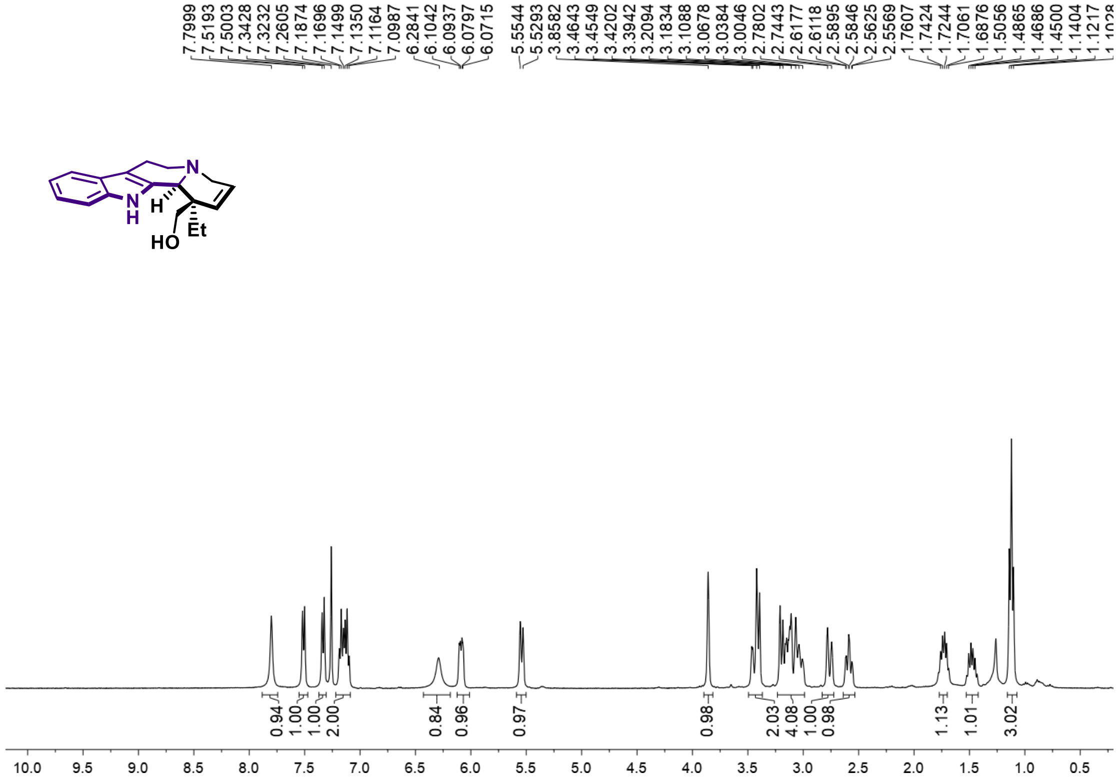

Supplementary Figure 312: ^{13}C NMR of 58 (101 MHz, $CDCl_3$)

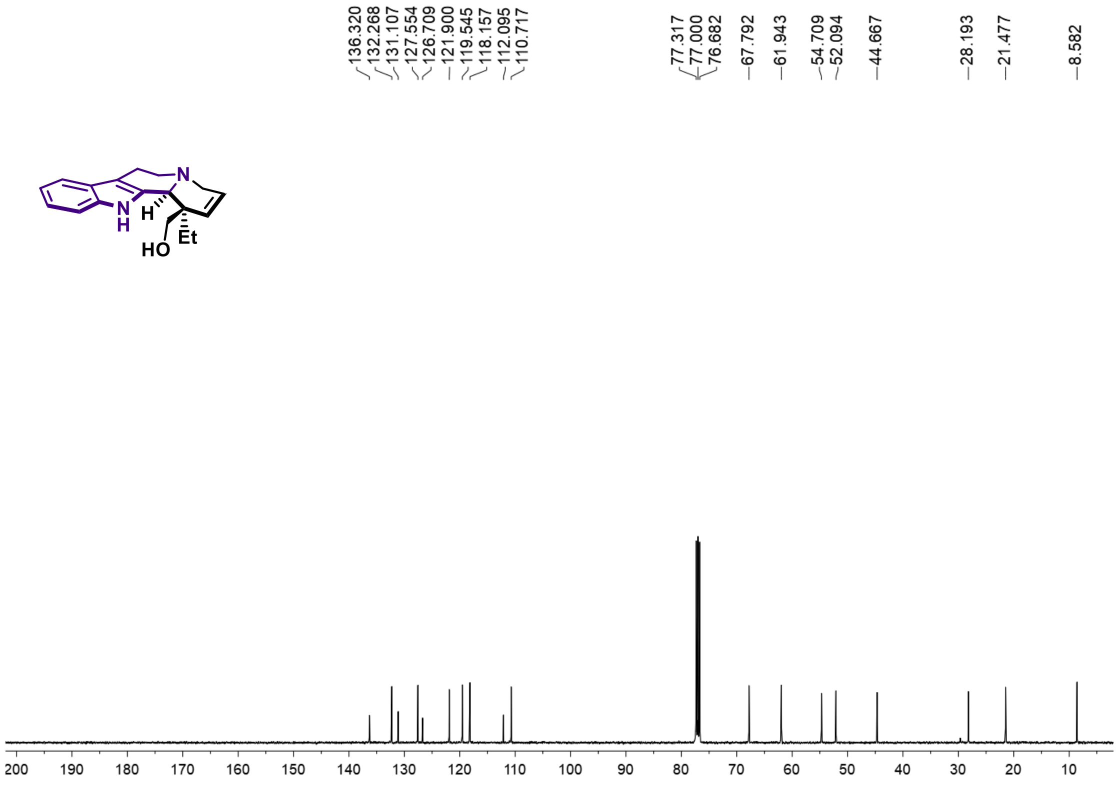

Supplementary Figure 313: ^{1}H NMR of 59 (400 MHz, $CDCl_3$)

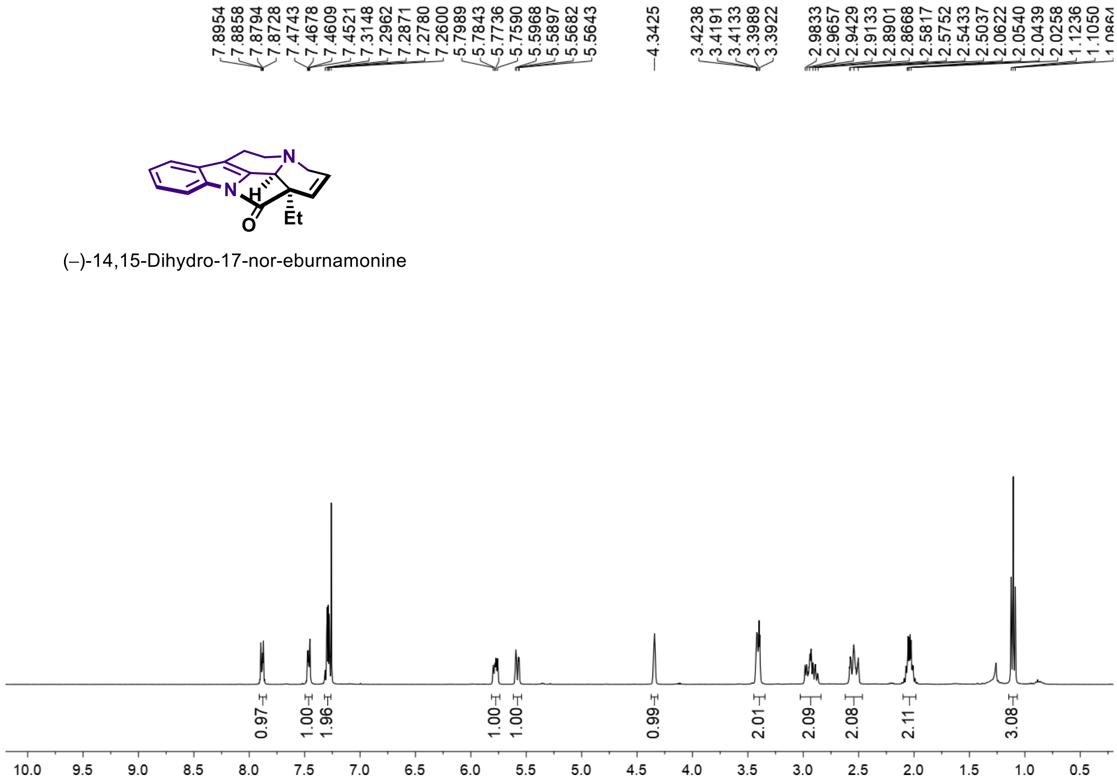

(–)-14,15-Dihydro-17-nor-eburnamonine

Supplementary Figure 314: ^{13}C NMR of 59 (101 MHz, $CDCl_3$)

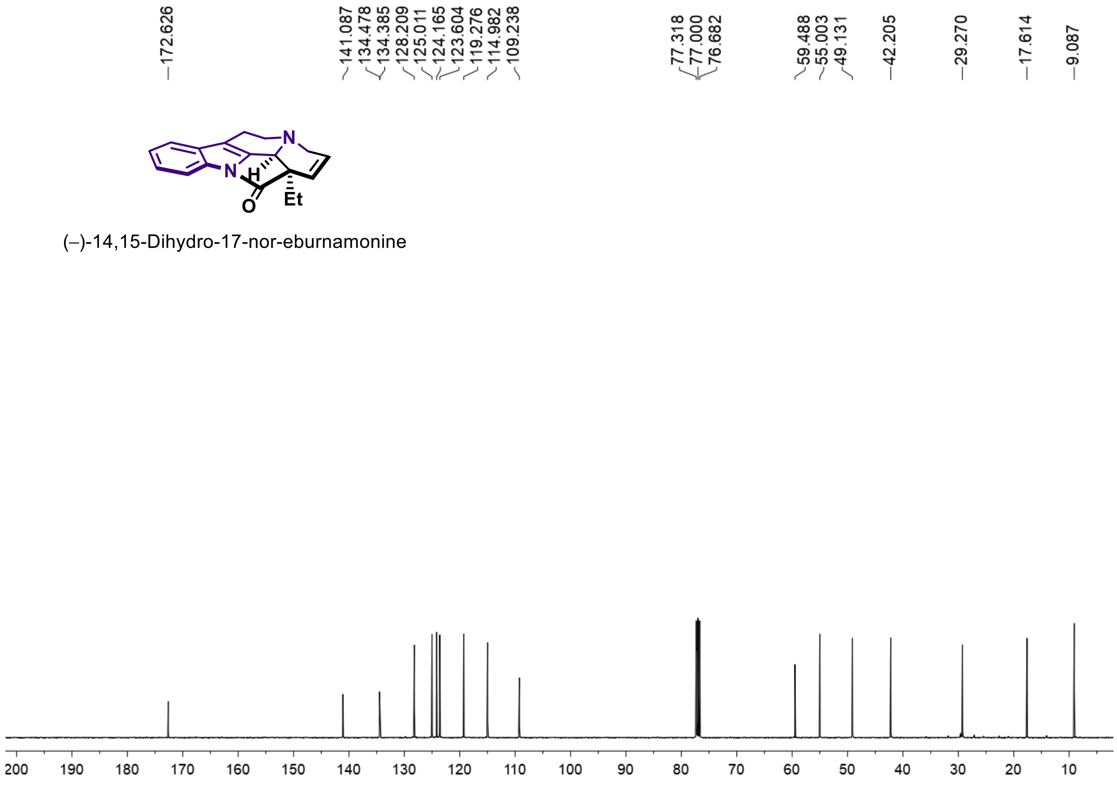

(–)-14,15-Dihydro-17-nor-eburnamonine

Supplementary Figure 315: ^{1}H NMR of 60 (400 MHz, CDCl$_3$)

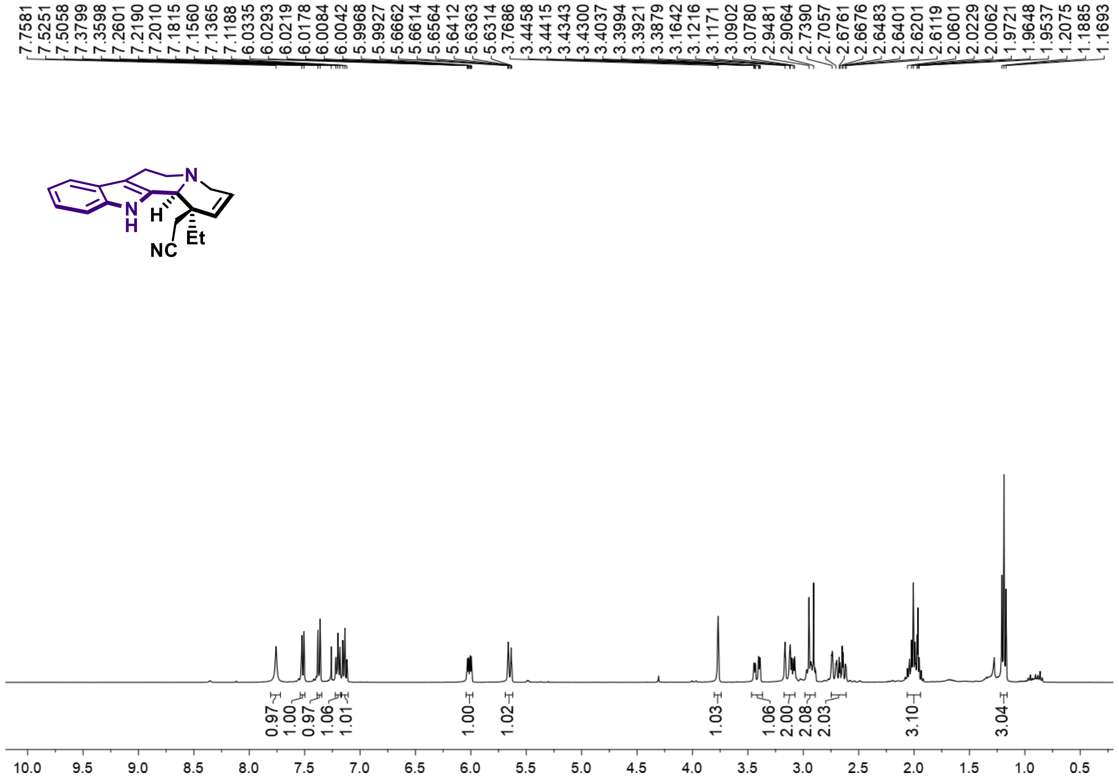

Supplementary Figure 316: ^{13}C NMR of 60 (101 MHz, CDCl$_3$)

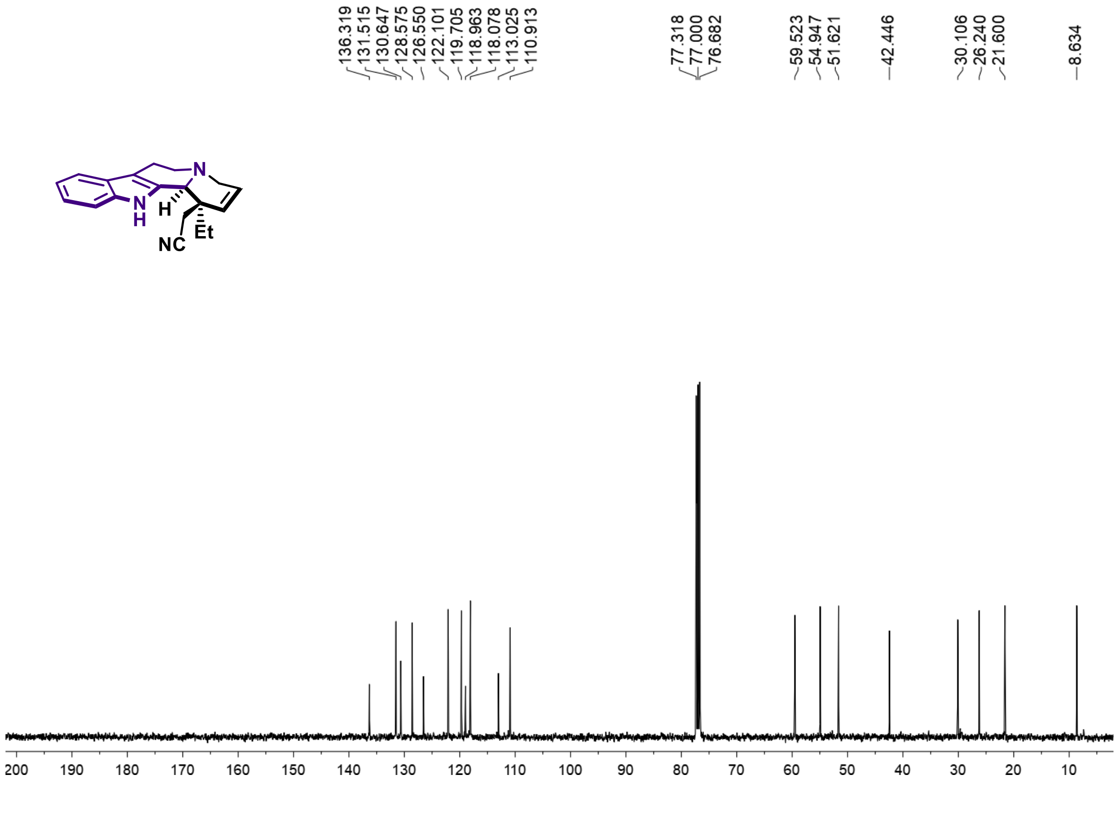

Supplementary Figure 317: ^{1}H NMR of 61 (400 MHz, $CDCl_3$)

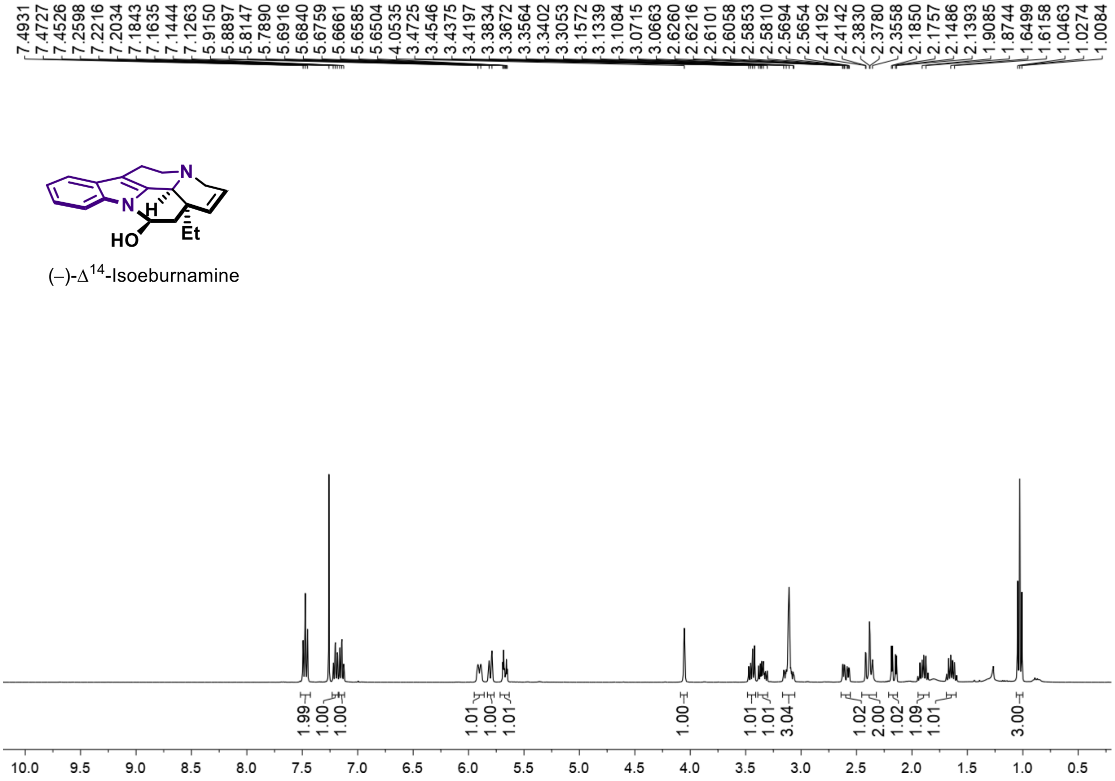

Supplementary Figure 318: ^{13}C NMR of 61 (101 MHz, $CDCl_3$)

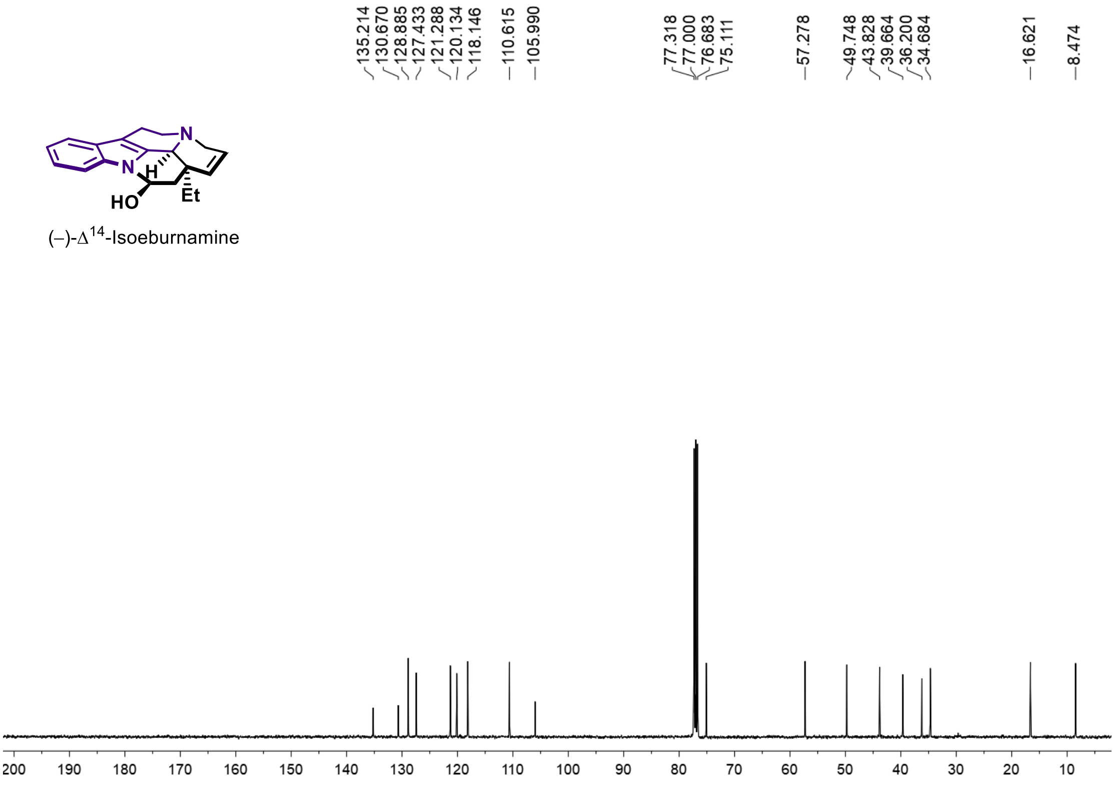

Supplementary Figure 319: ^{1}H NMR of 62 (400 MHz, $CDCl_3$)

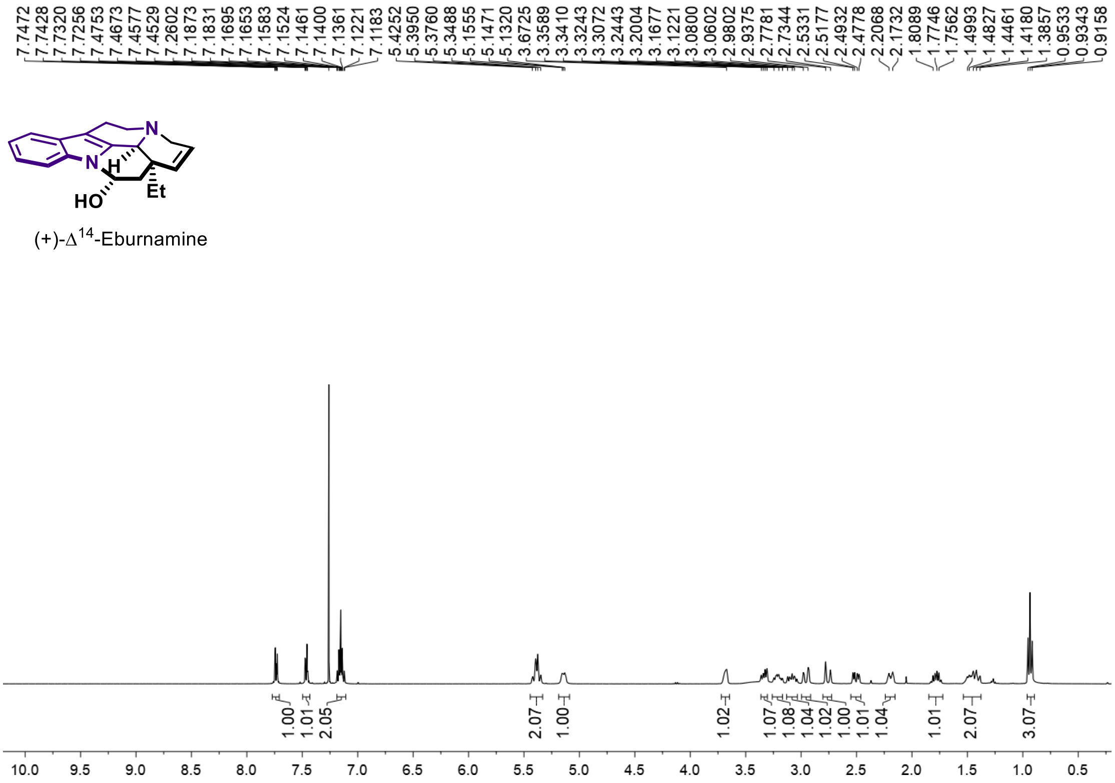

Supplementary Figure 320: ^{13}C NMR of 62 (101 MHz, $CDCl_3$)

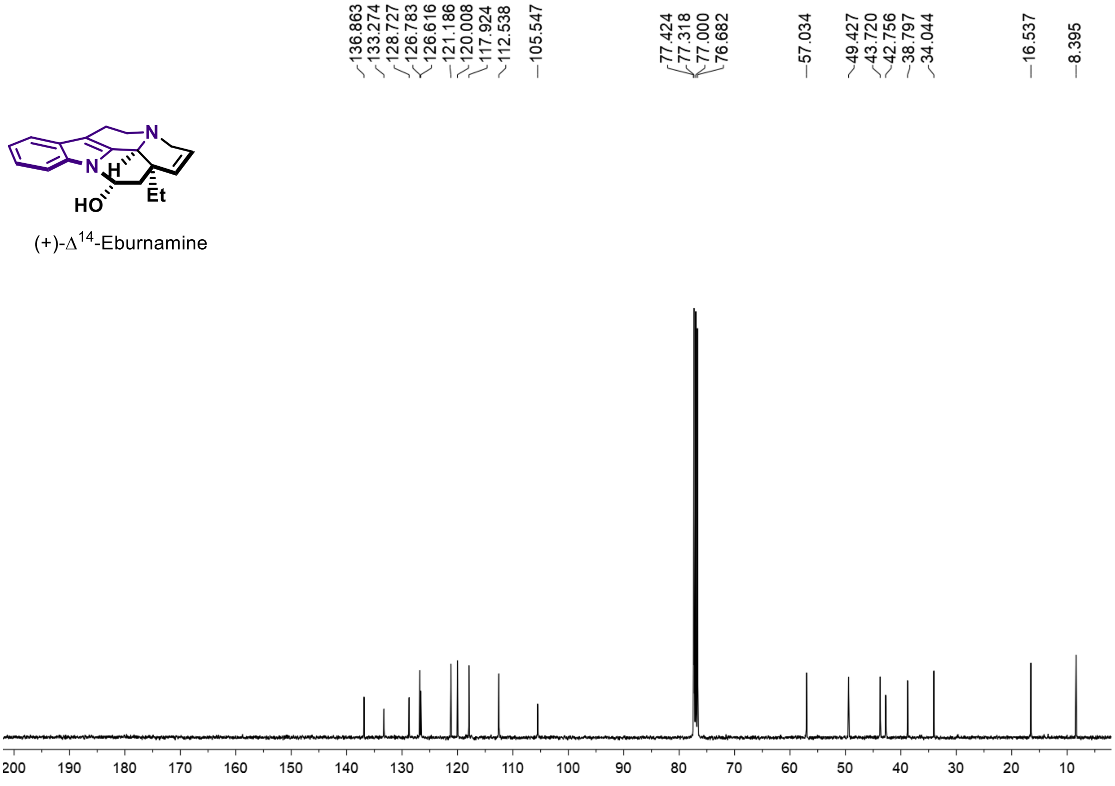

Supplementary Figure 321: ^{1}H NMR of 63 (400 MHz, CDCl$_3$)

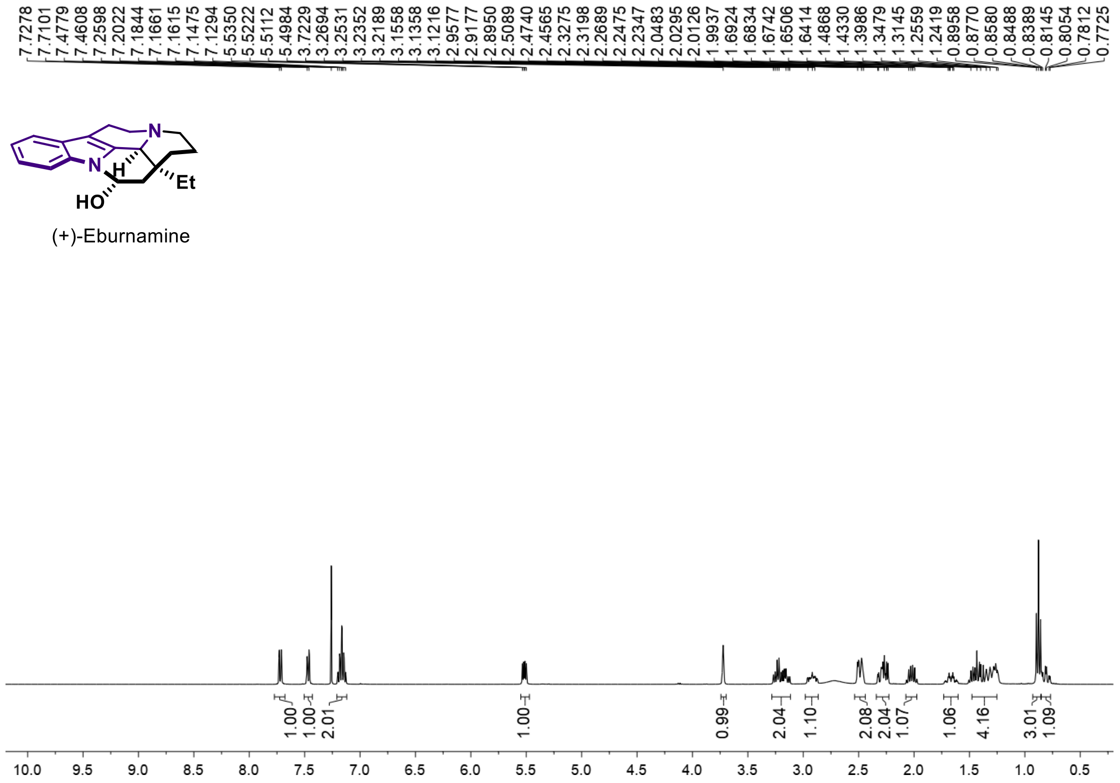

Supplementary Figure 322: ^{13}C NMR of 63 (101 MHz, CDCl$_3$)

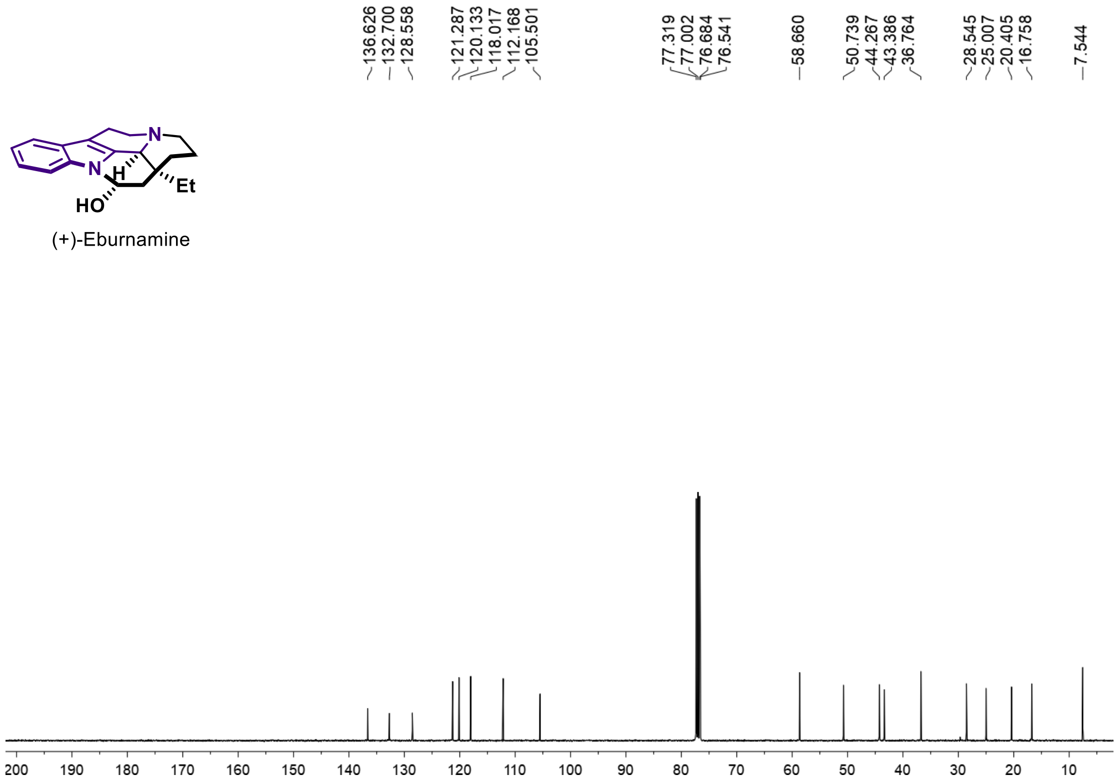

Supplementary Figure 323: ^{1}H NMR of 64 (400 MHz, CDCl$_3$)

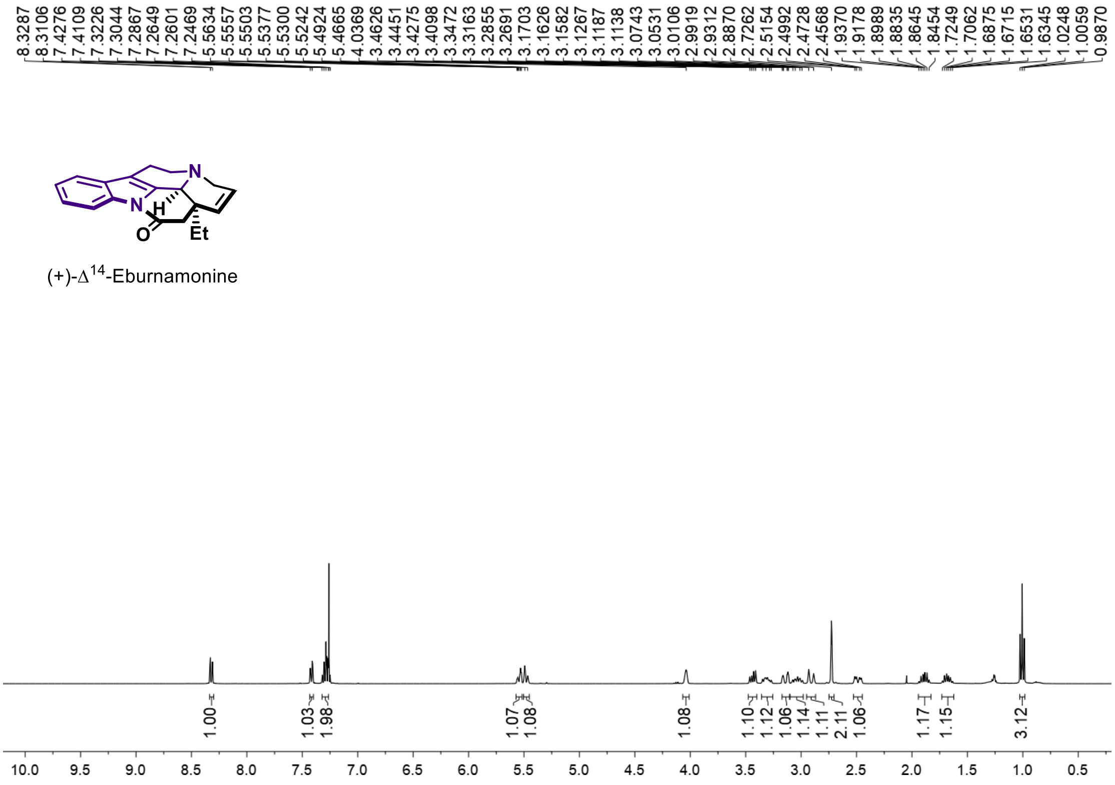

Supplementary Figure 324: ^{13}C NMR of 64 (101 MHz, CDCl$_3$)

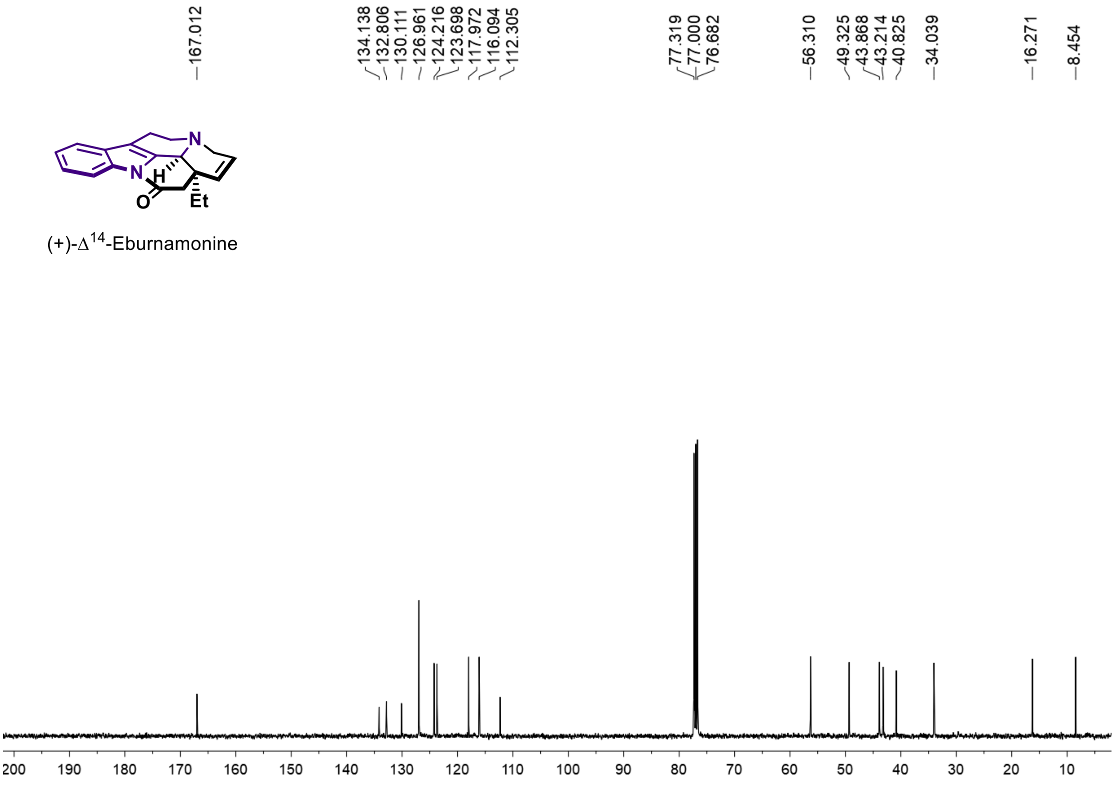

Supplementary Figure 325: ^{1}H NMR of 65 (400 MHz, $CDCl_3$)

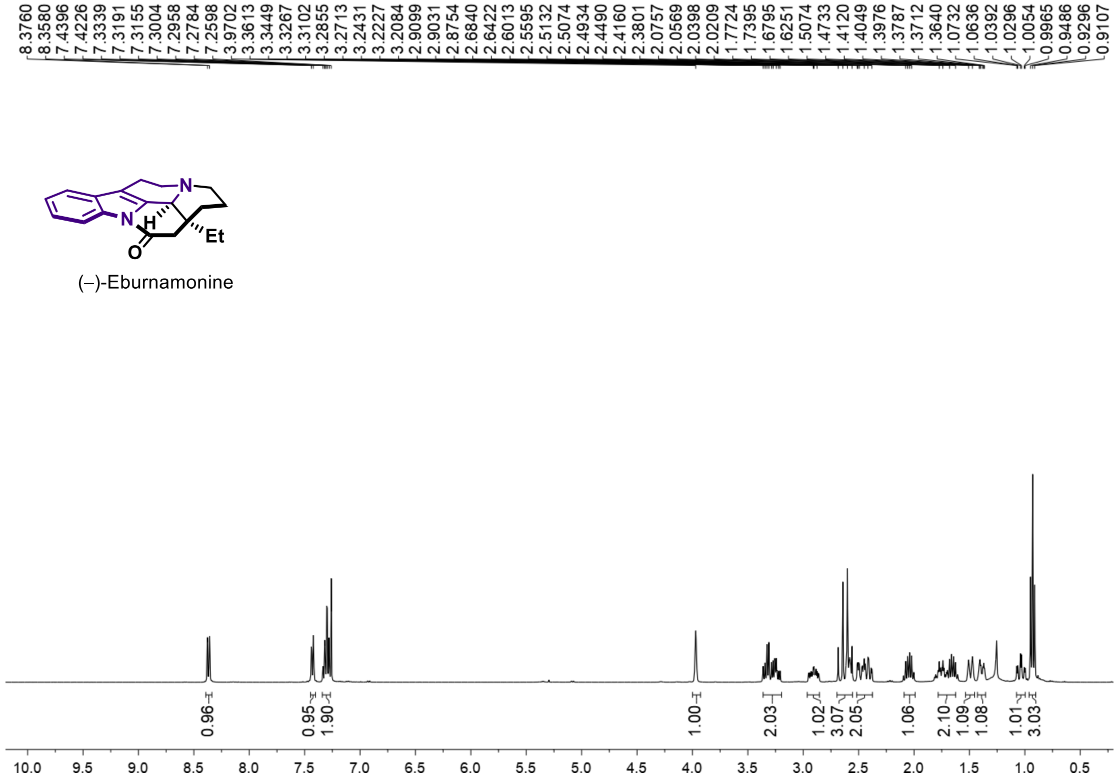

Supplementary Figure 326: ^{13}C NMR of 65 (101 MHz, $CDCl_3$)

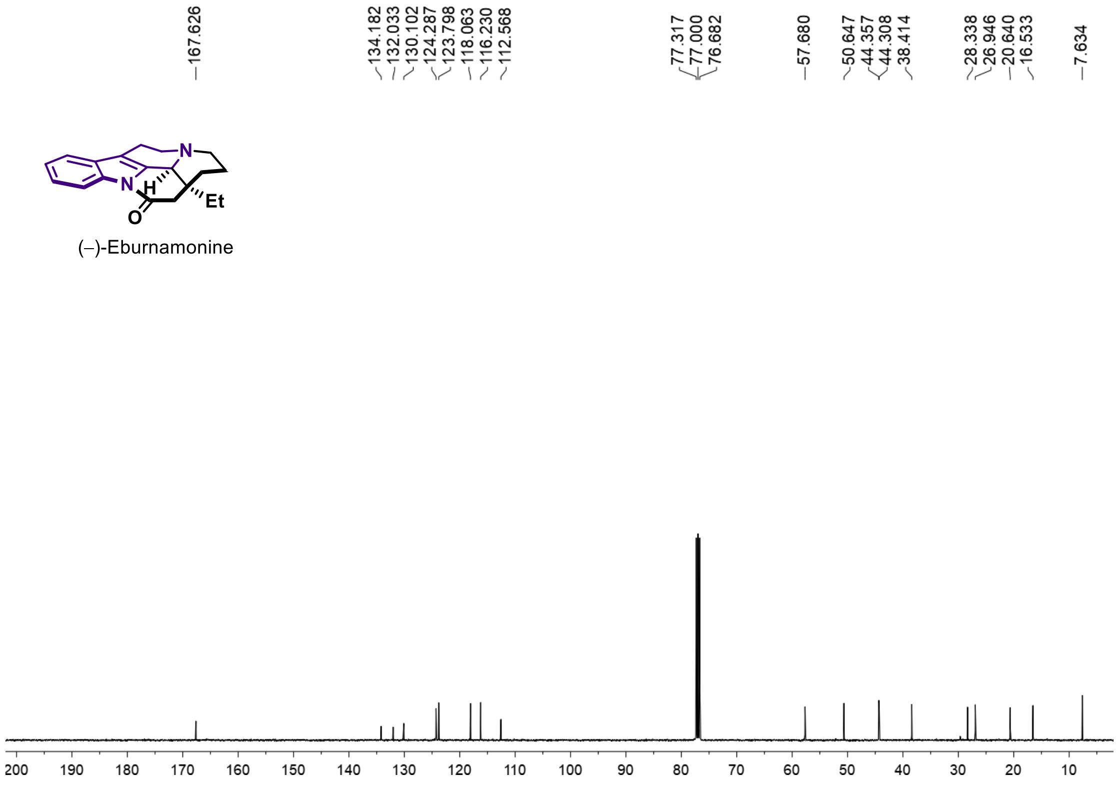

Supplementary Figure 327: ^{1}H NMR of 66 (400 MHz, $CDCl_3$)

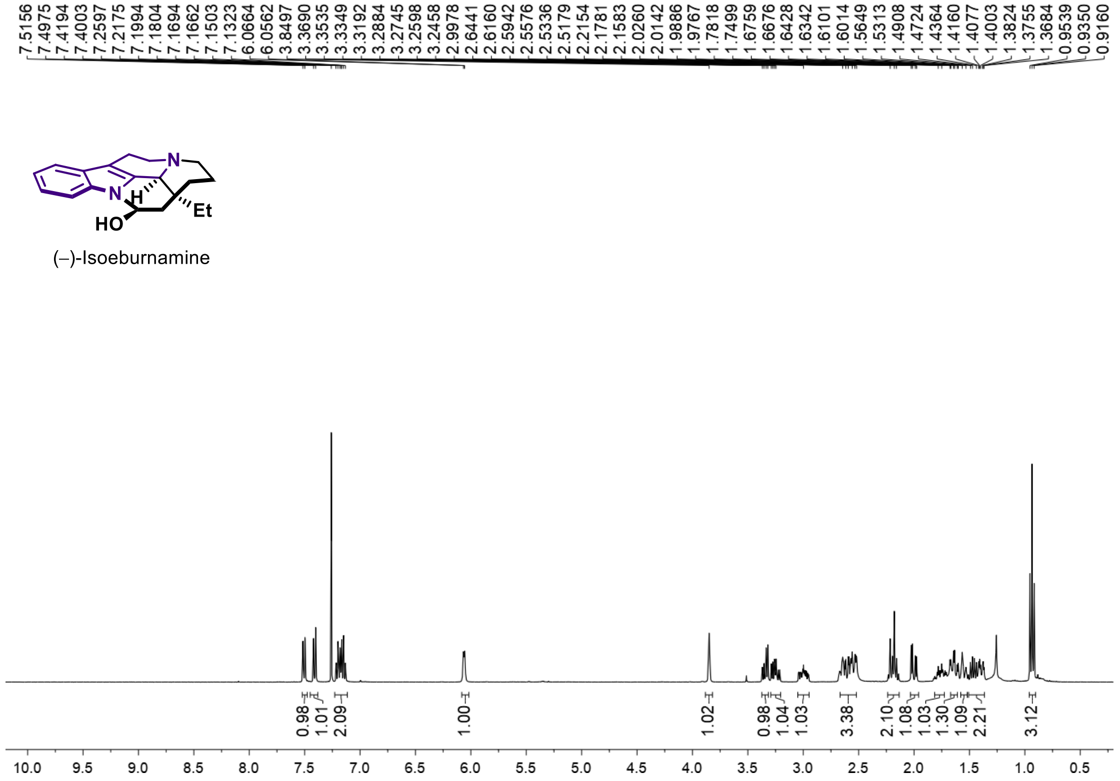

Supplementary Figure 328: ^{13}C NMR of 66 (101 MHz, $CDCl_3$)

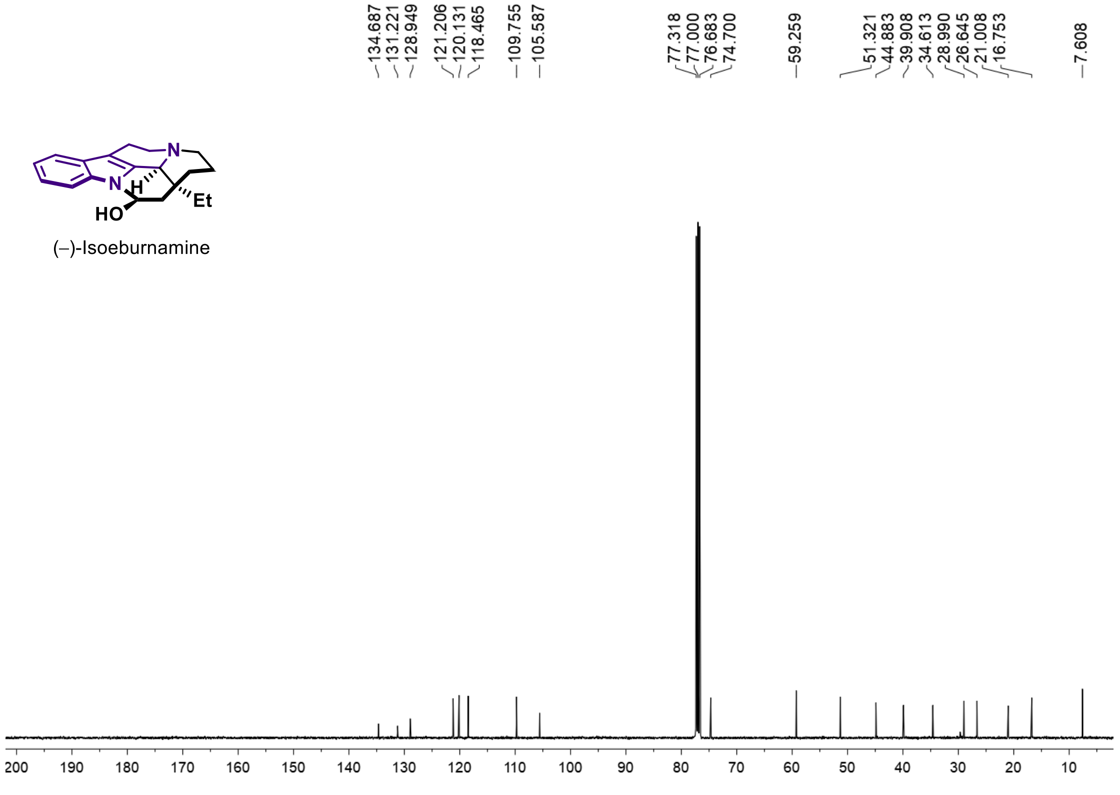

Supplementary Figure 329: ^{1}H NMR of 67 (400 MHz, $CDCl_3$)

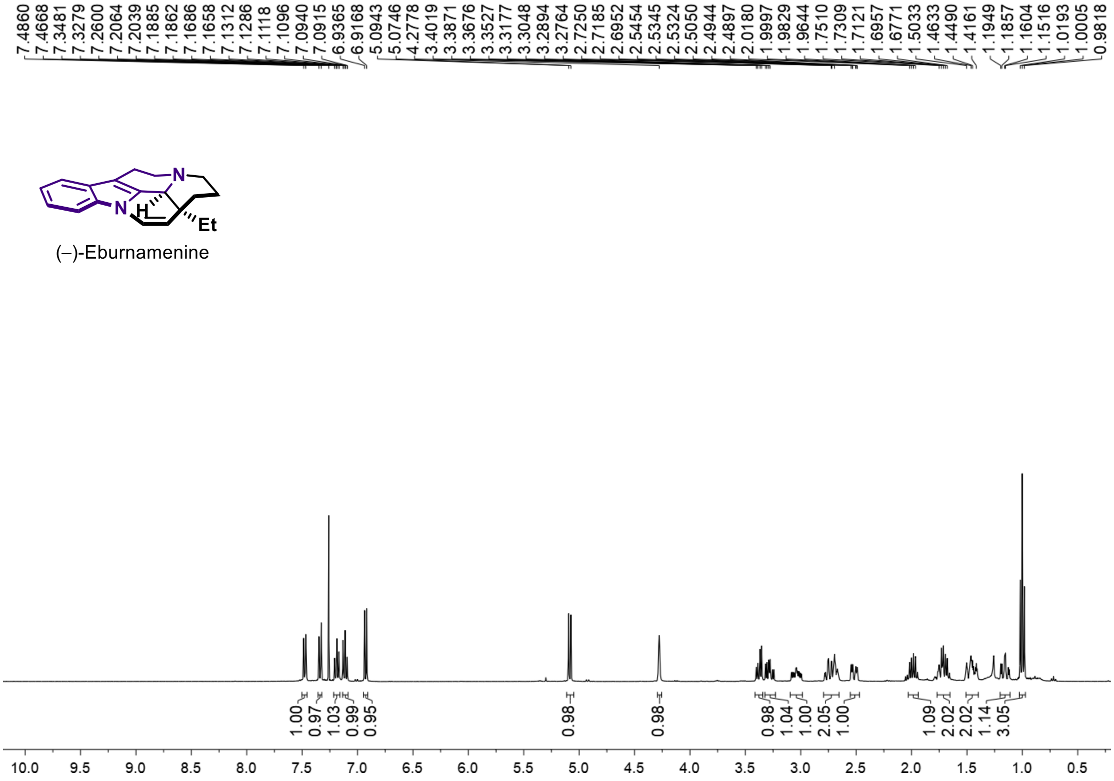

Supplementary Figure 330: ^{13}C NMR of 67 (101 MHz, $CDCl_3$)

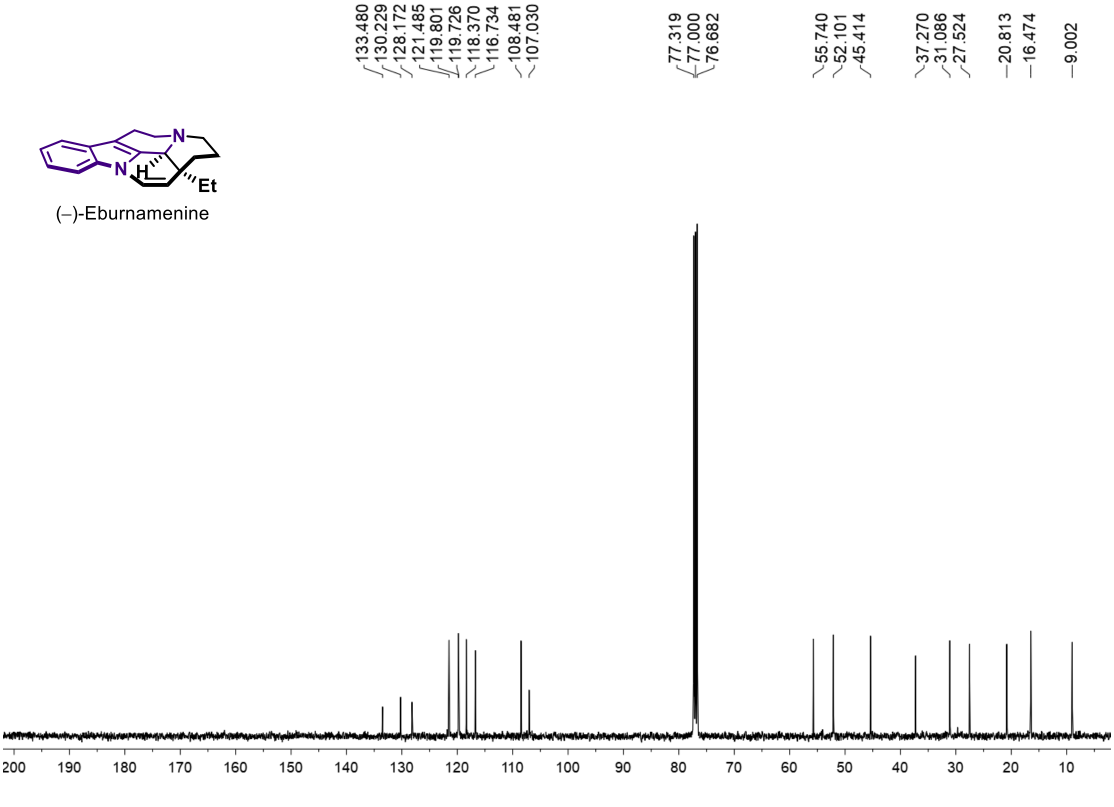

Supplementary Figure 331: ^{1}H NMR of 68 (400 MHz, $CDCl_3$)

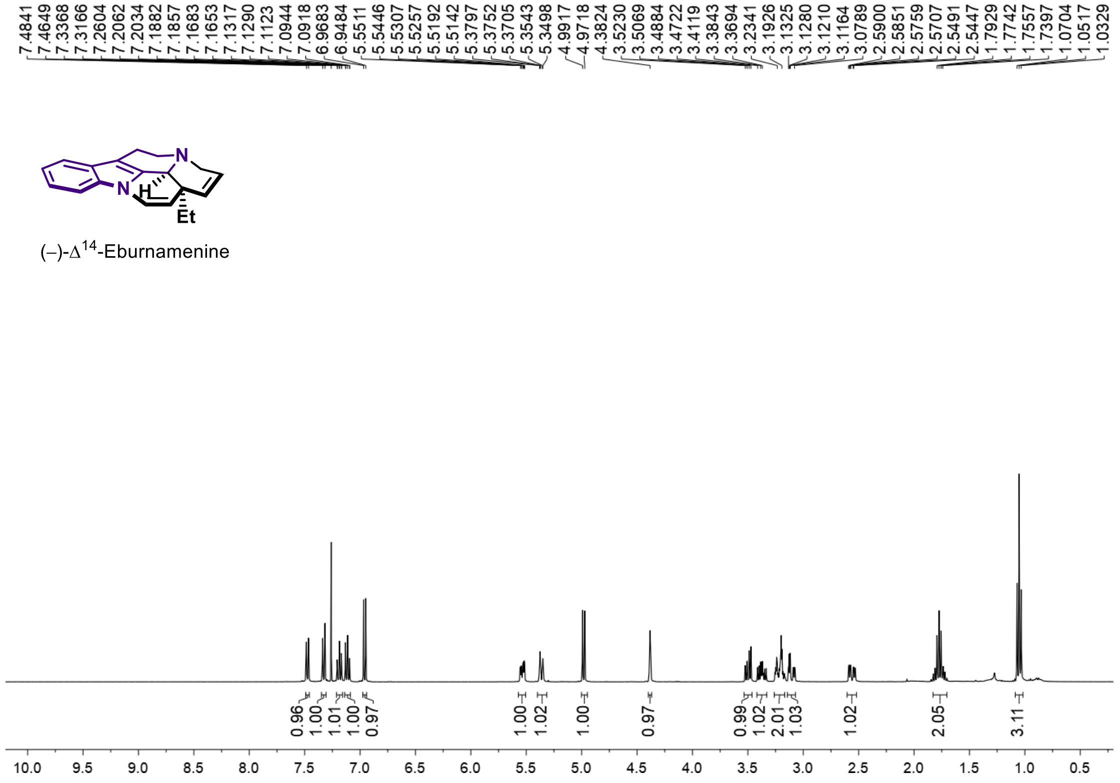

Supplementary Figure 332: ^{13}C NMR of 68 (101 MHz, $CDCl_3$)

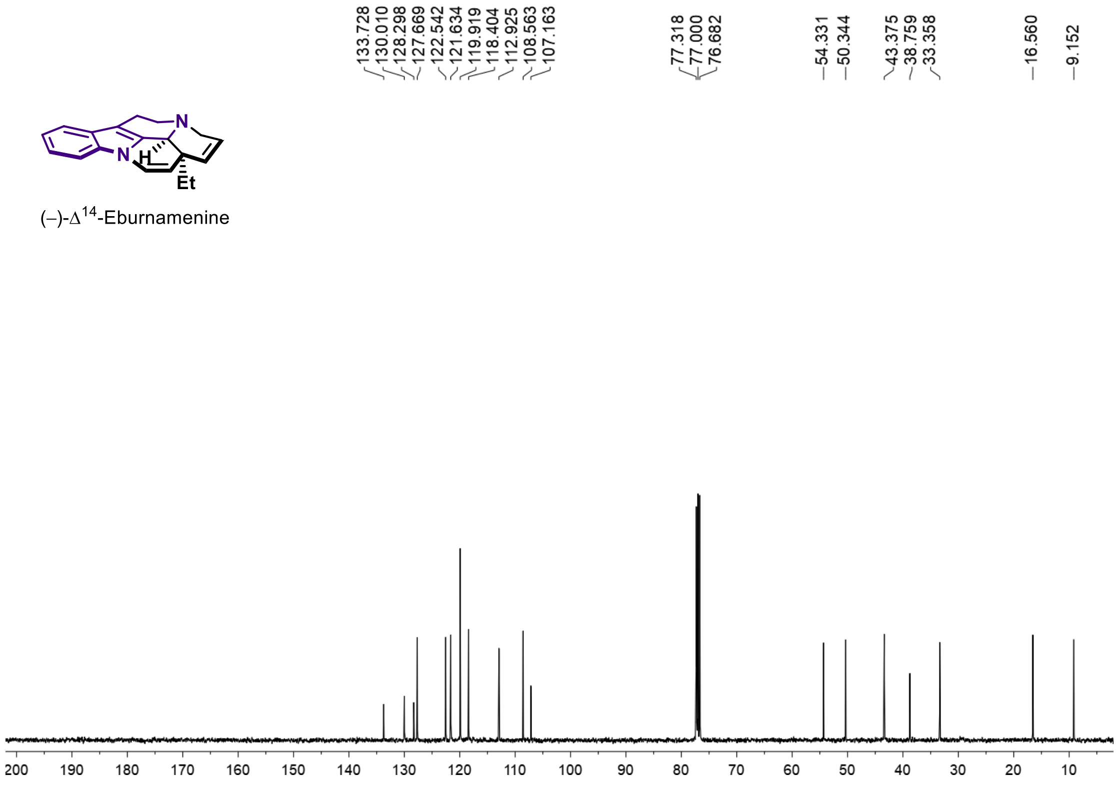

Supplementary Figure 333: ^{1}H NMR of 69 (400 MHz, $CDCl_3$)

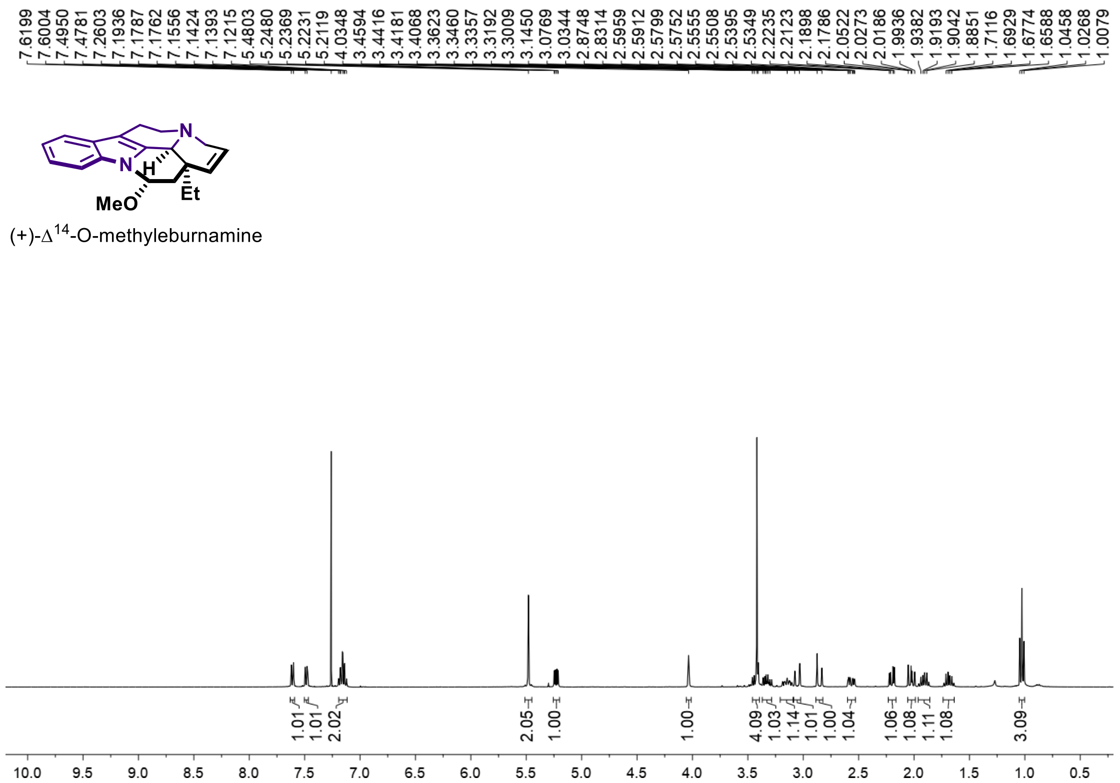

Supplementary Figure 334: ^{13}C NMR of 69 (101 MHz, $CDCl_3$)

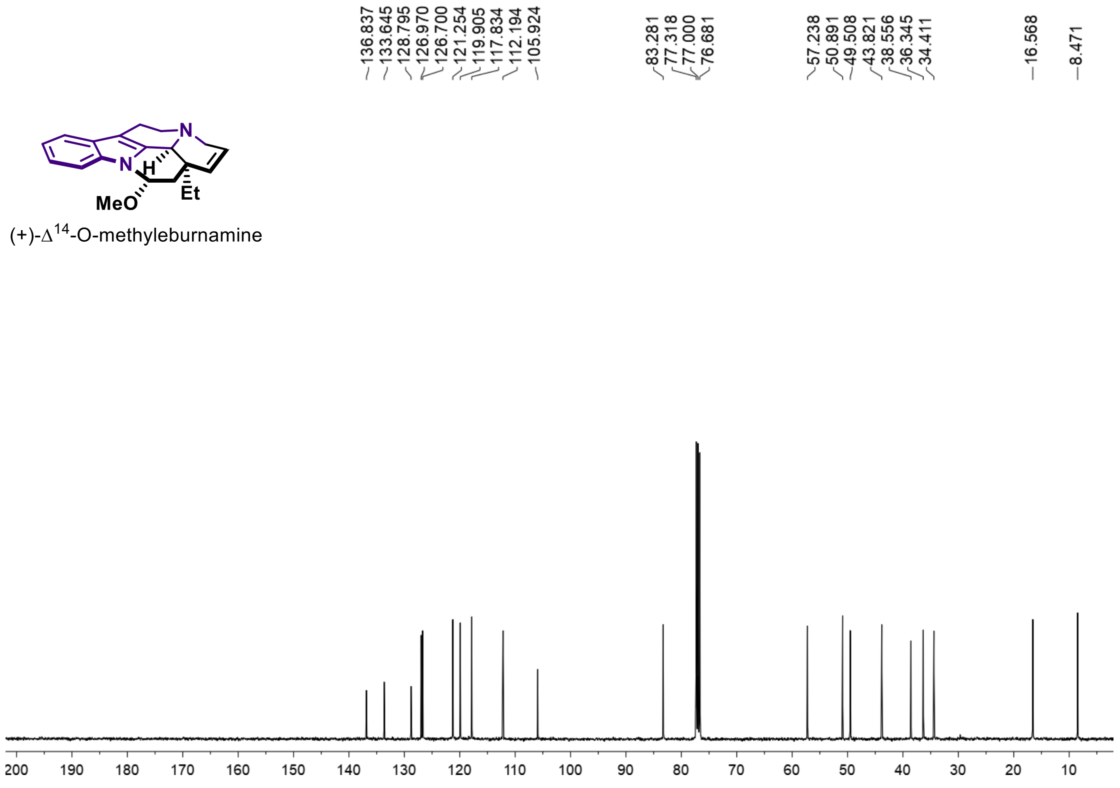

Supplementary Figure 335: ^{1}H NMR of 70 (400 MHz, $CDCl_3$)

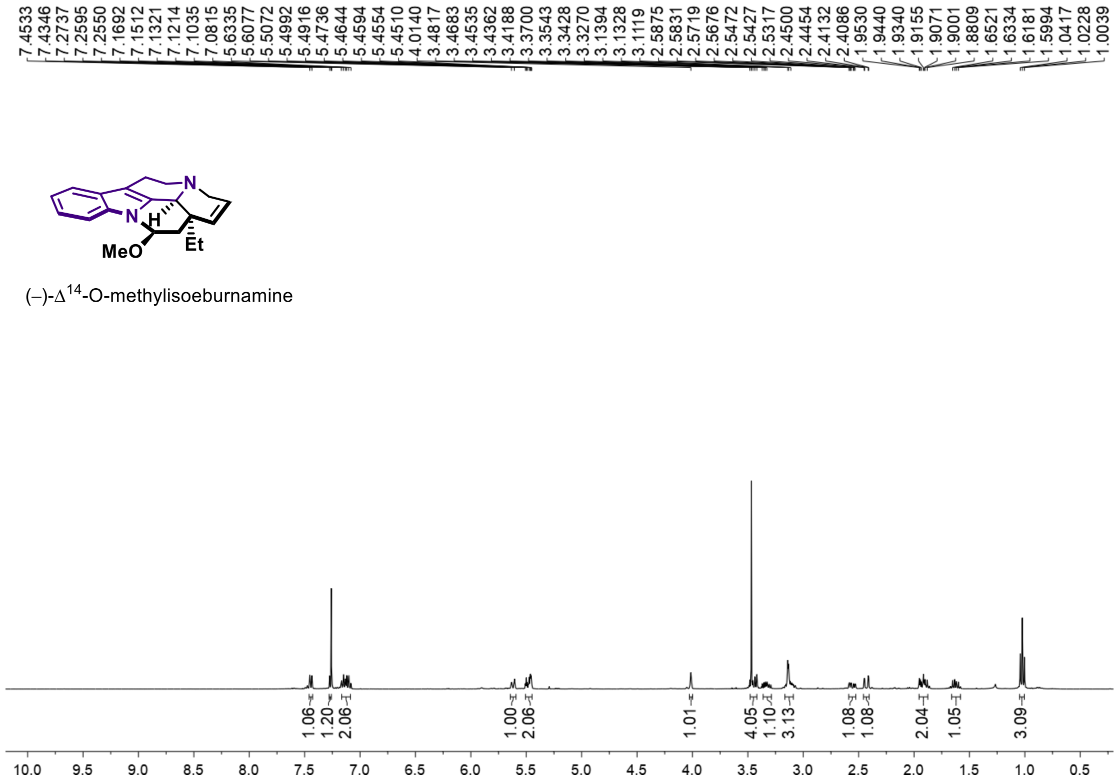

Supplementary Figure 336: ^{13}C NMR of 70 (101 MHz, $CDCl_3$)

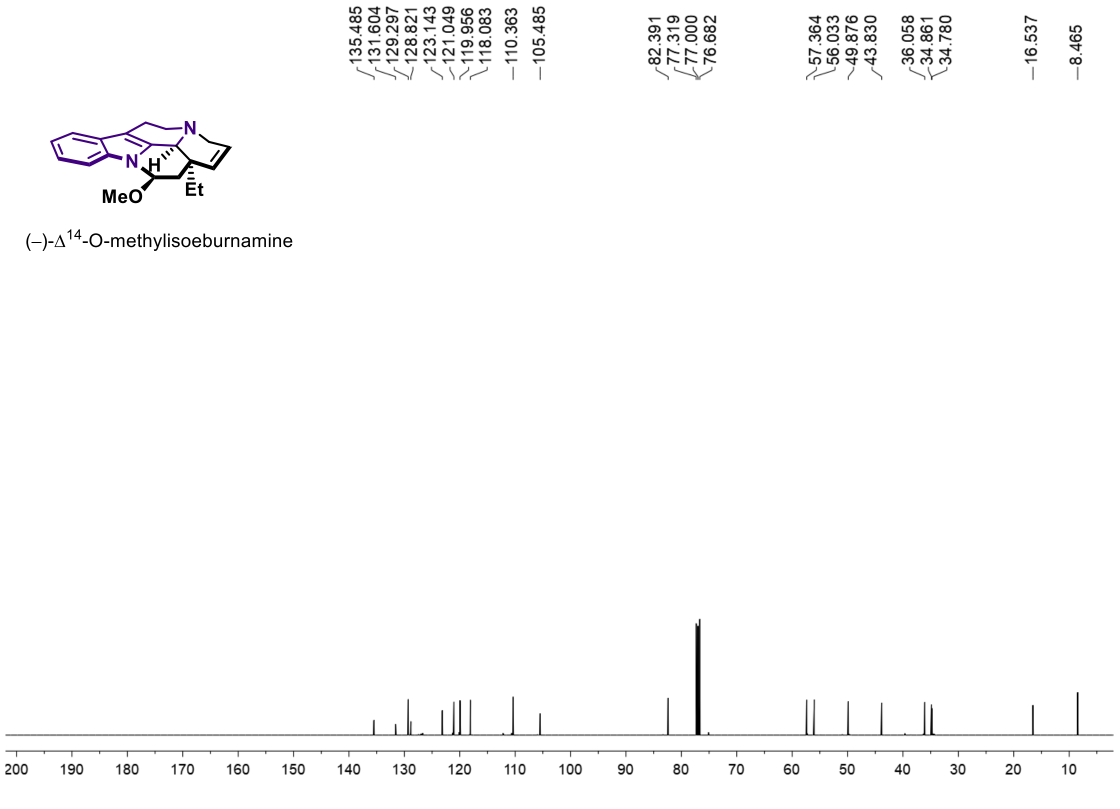

Supplementary Figure 337: ^{1}H NMR of 71 (400 MHz, $CDCl_3$)

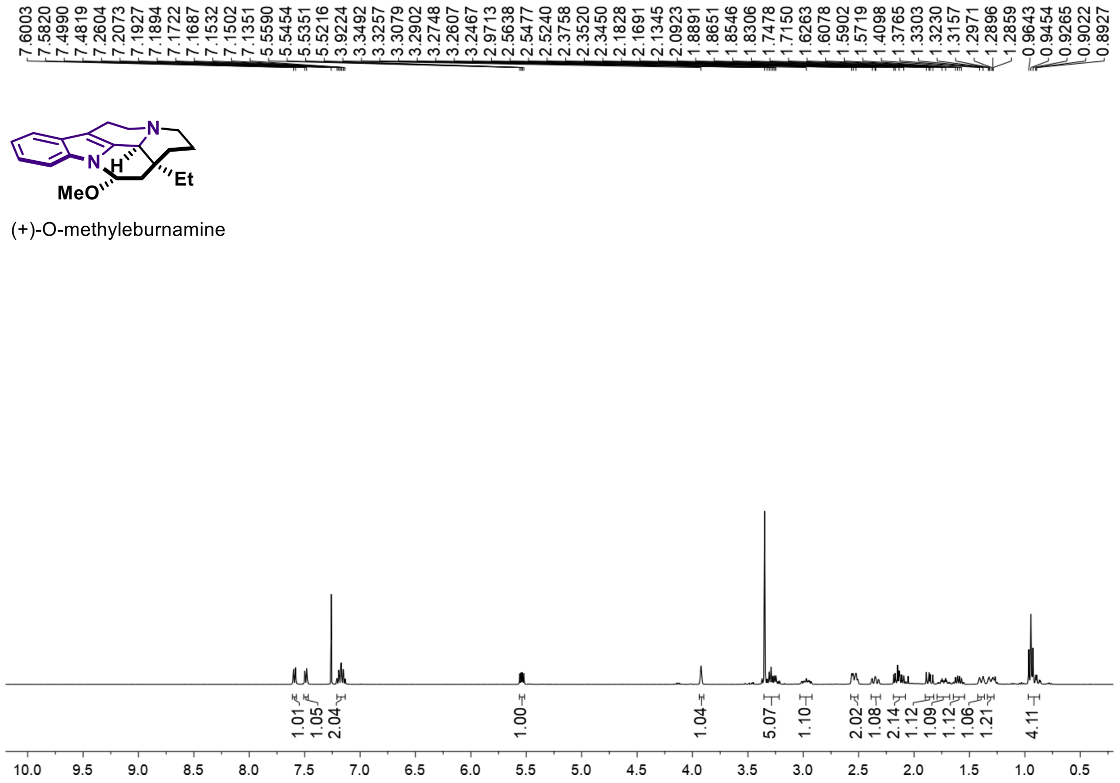

Supplementary Figure 338: ^{13}C NMR of 71 (101 MHz, $CDCl_3$)

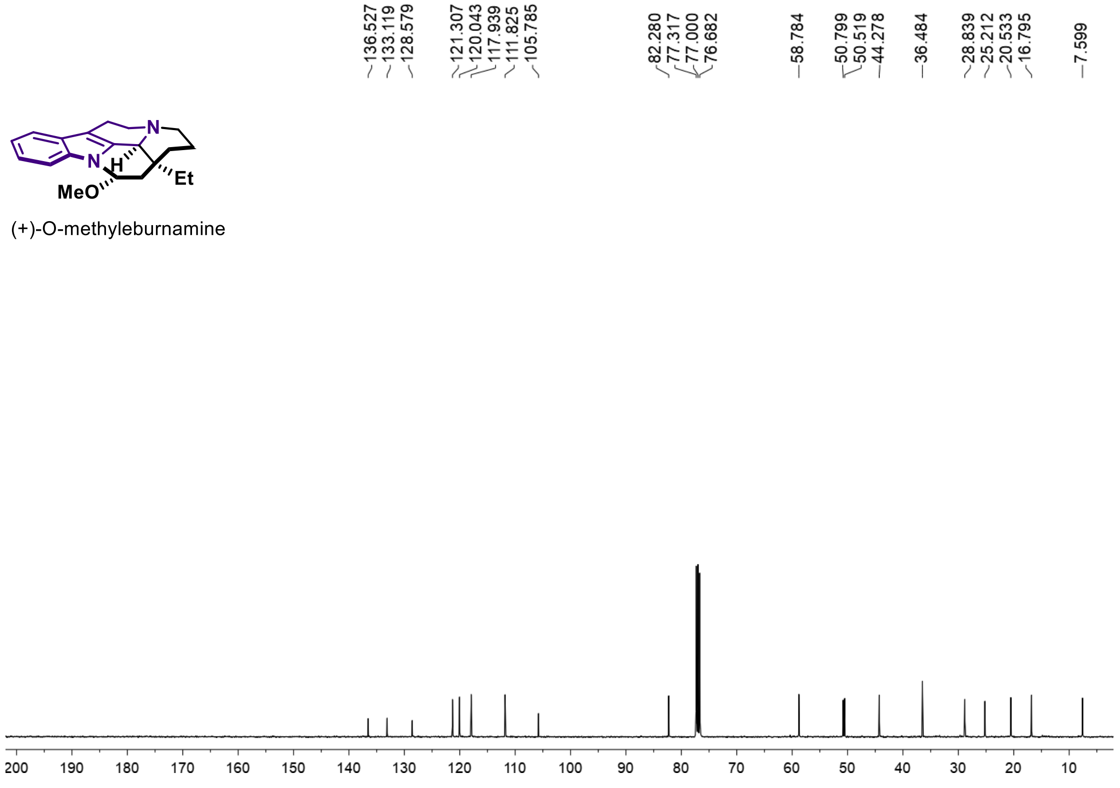

Supplementary Figure 339: ^{1}H NMR of 72 (400 MHz, $CDCl_3$)

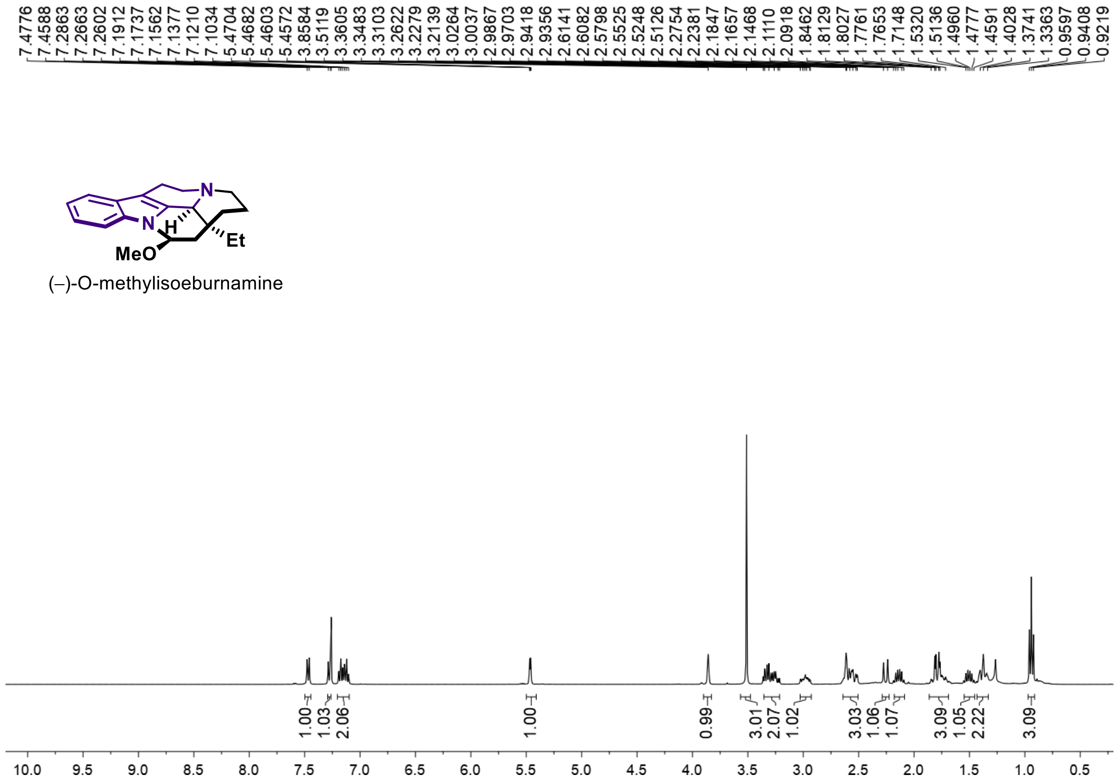

Supplementary Figure 340: ^{13}C NMR of 72 (101 MHz, $CDCl_3$)

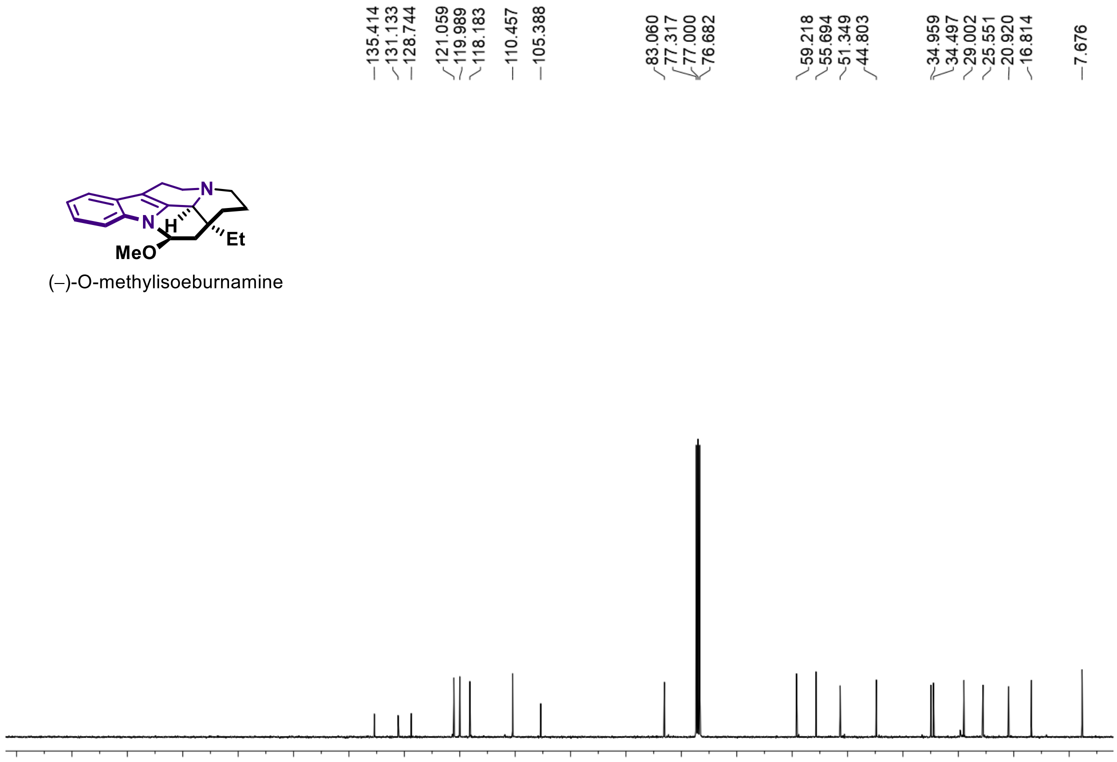

Supplementary Figure 341: ^{1}H NMR of 73 (400 MHz, $CDCl_3$)

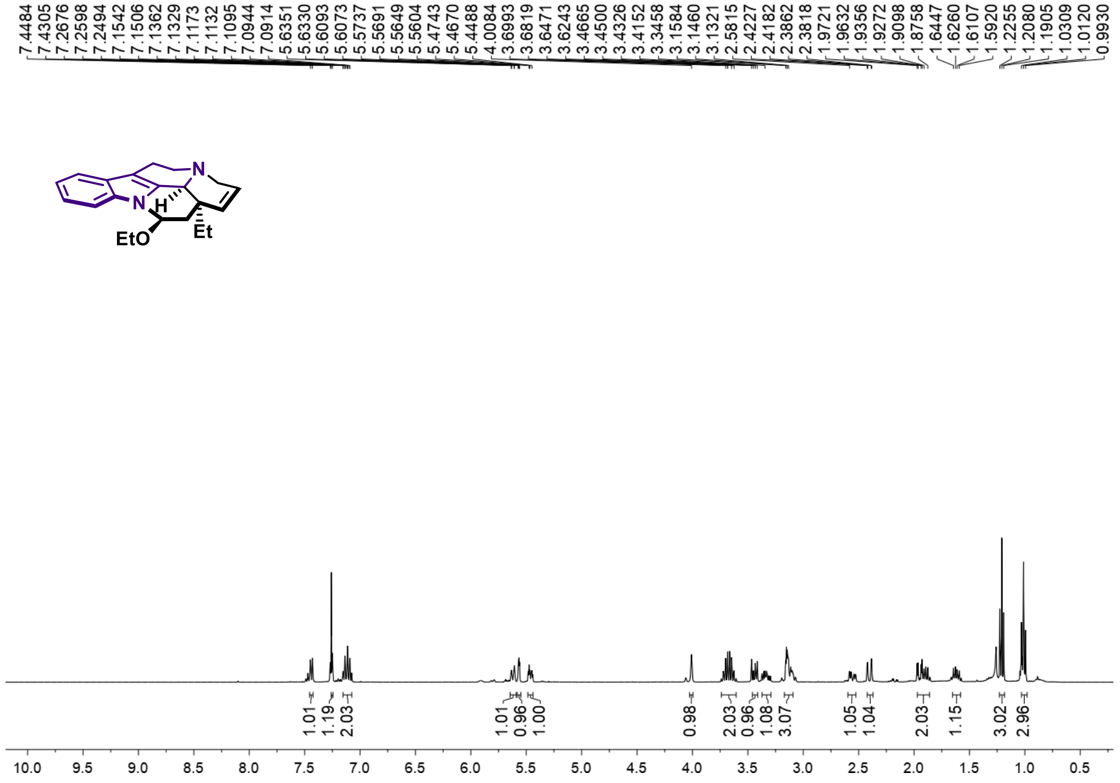

Supplementary Figure 342: ^{13}C NMR of 73 (101 MHz, $CDCl_3$)

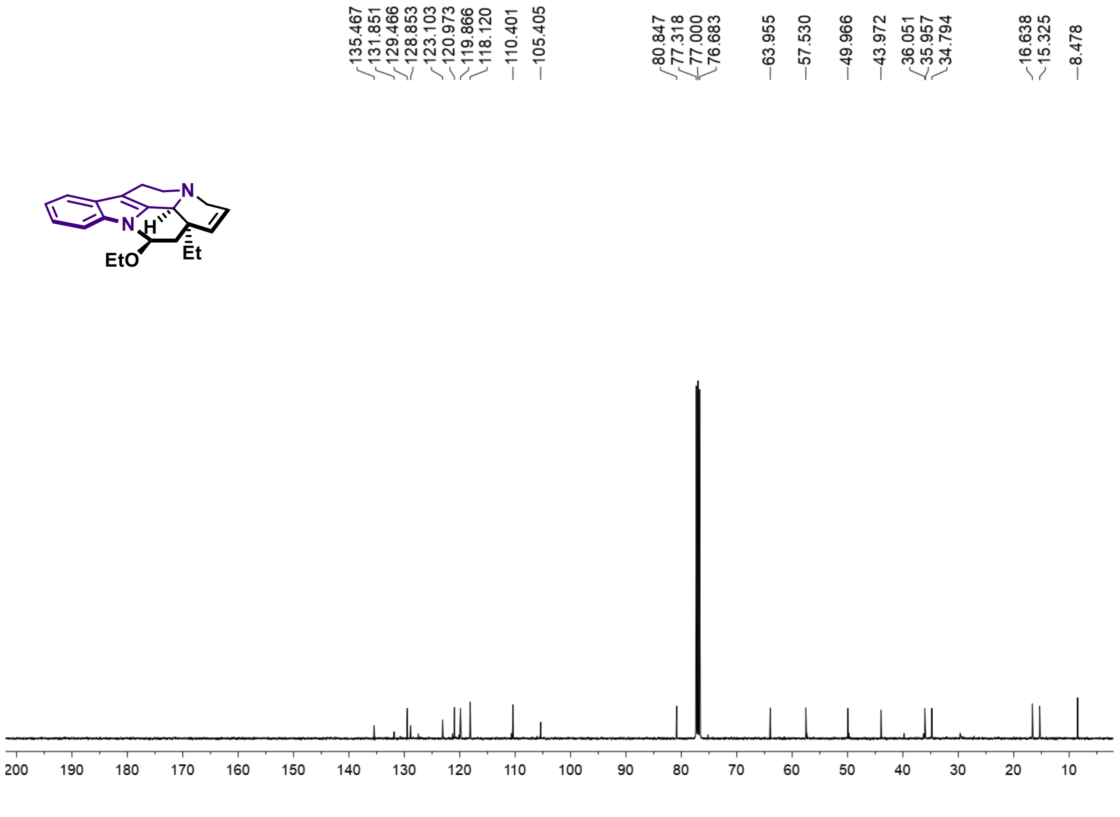

Supplementary Figure 343: ^{1}H NMR of 74 (400 MHz, $CDCl_3$)

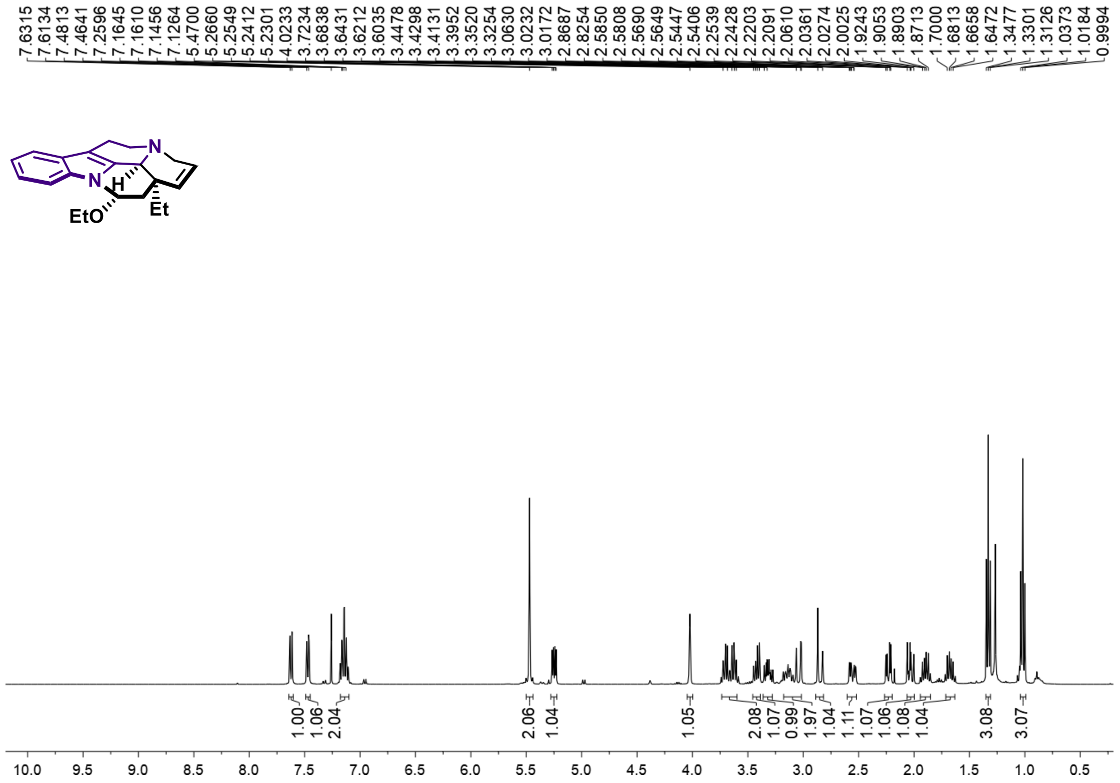

Supplementary Figure 344: ^{13}C NMR of 74 (101 MHz, $CDCl_3$)

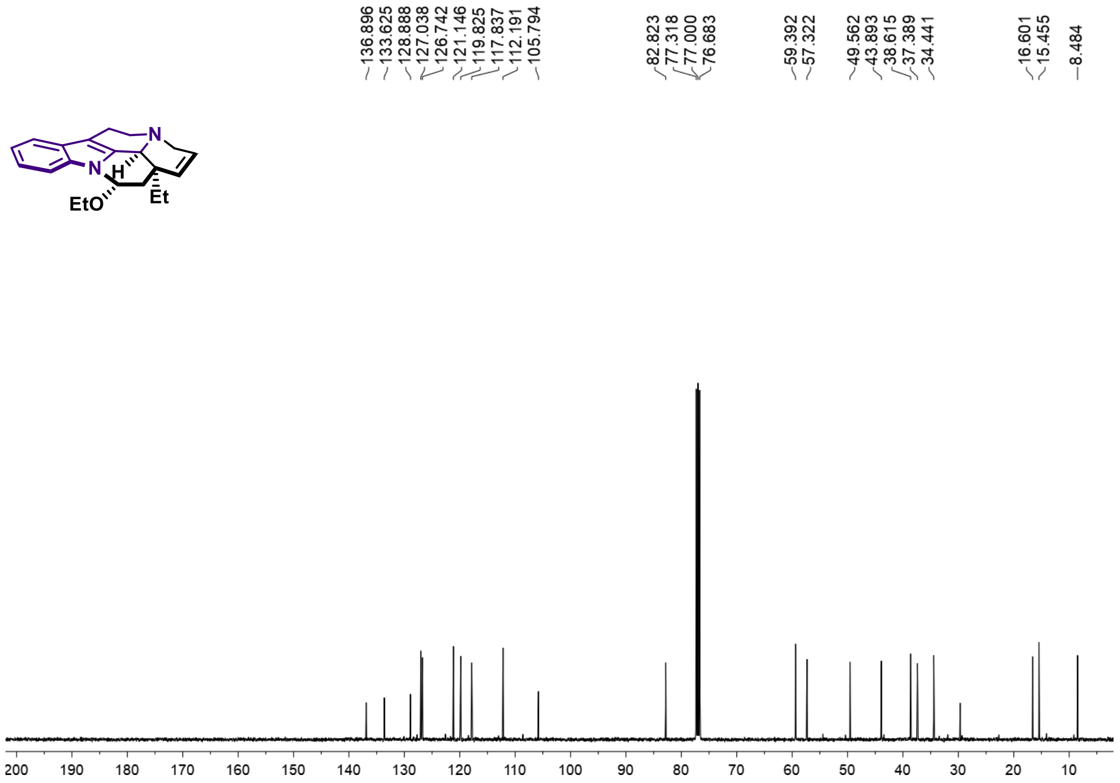

Supplementary Figure 345: ^{1}H NMR of 75 (400 MHz, $CDCl_3$)

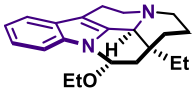

(+)-O-ethyleburnamine

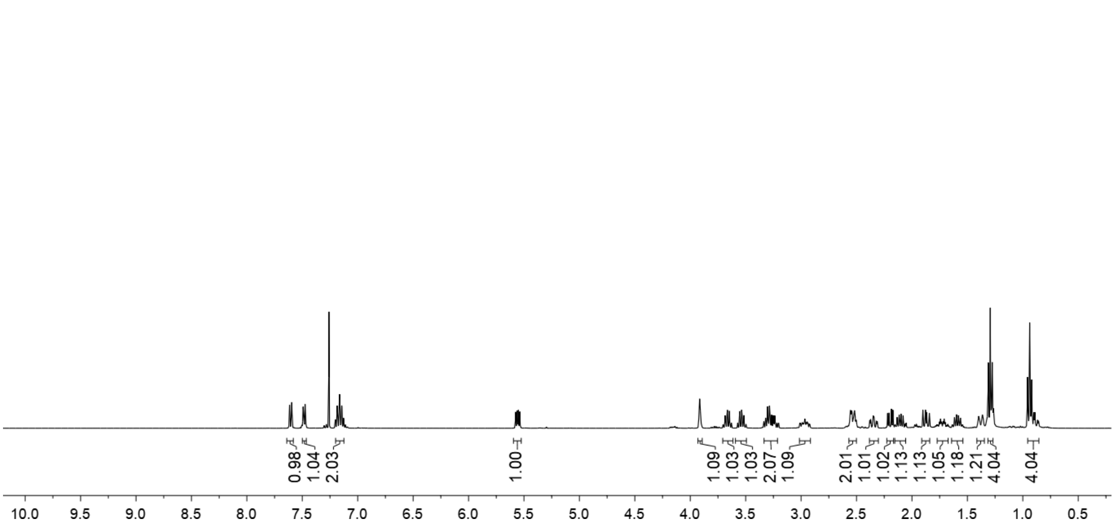

Supplementary Figure 346: ^{13}C NMR of 75 (101 MHz, $CDCl_3$)

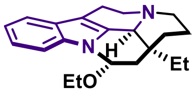

(+)-O-ethyleburnamine

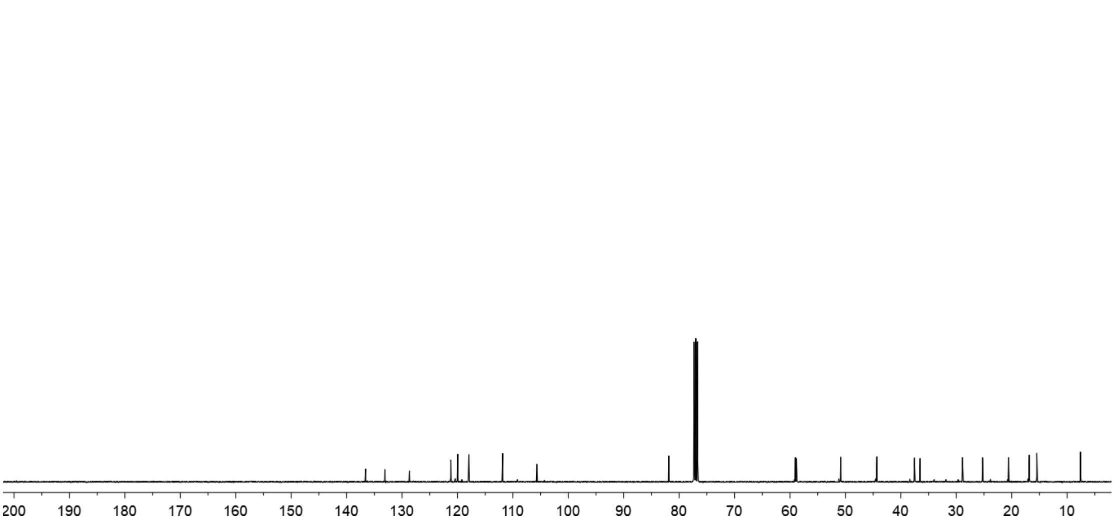

Supplementary Figure 347: ^{1}H NMR of 76 (400 MHz, $CDCl_3$)

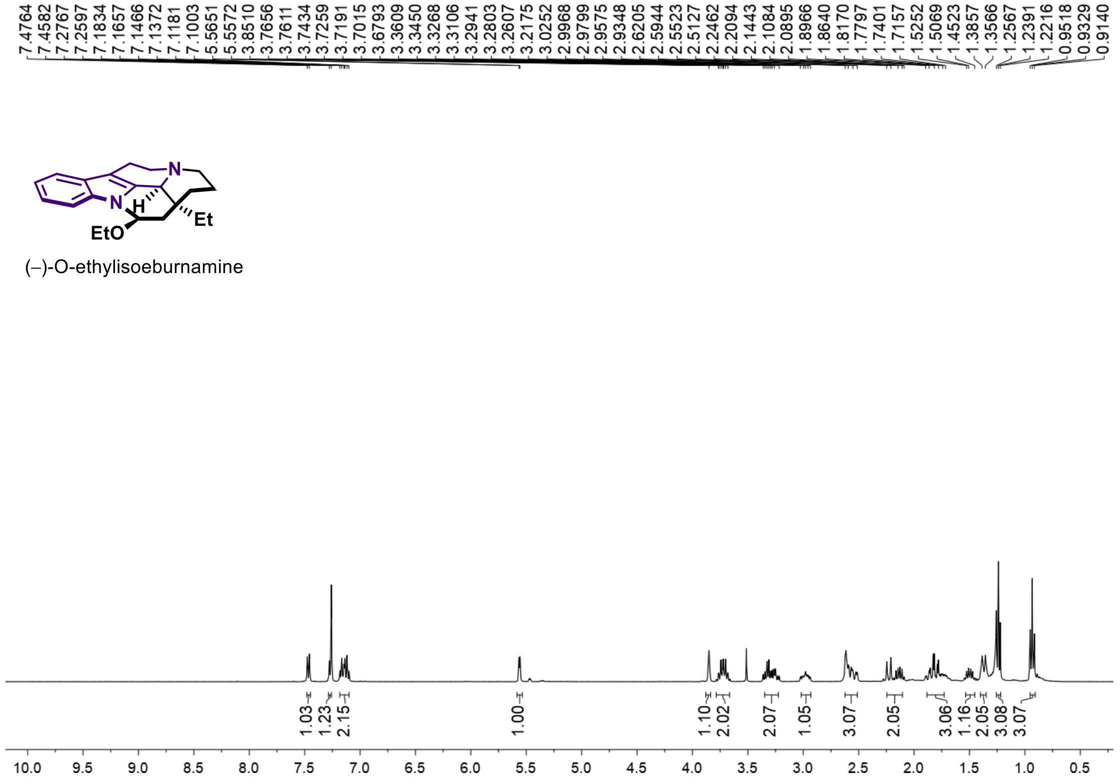

Supplementary Figure 348: ^{13}C NMR of 76 (101 MHz, $CDCl_3$)

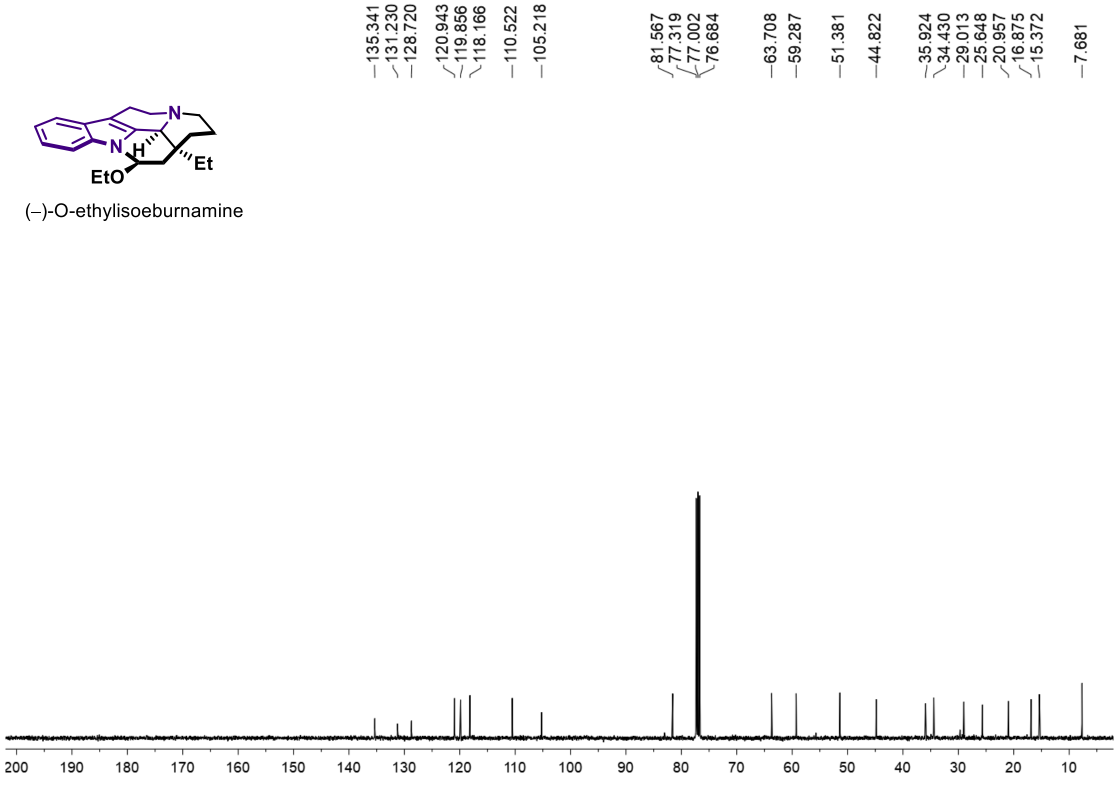

Supplementary Figure 349: ^{1}H NMR of 77 (400 MHz, $CDCl_3$)

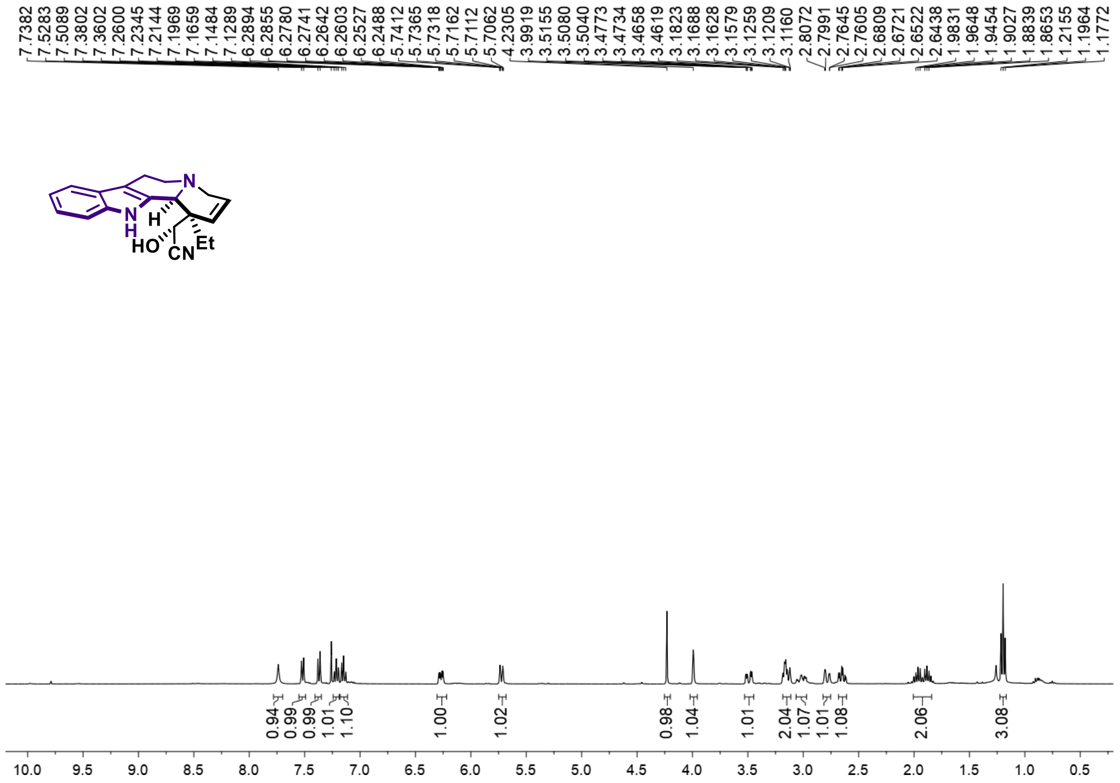

Supplementary Figure 350: ^{13}C NMR of 77 (101 MHz, $CDCl_3$)

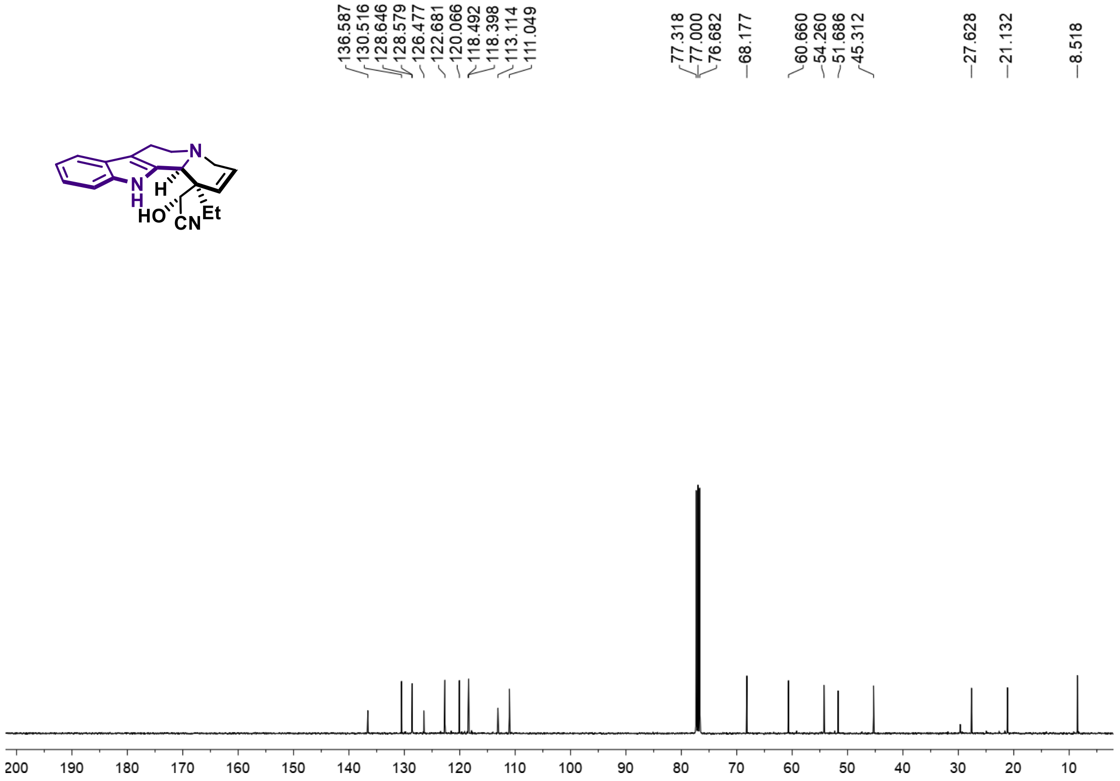

Supplementary Figure 351: ^{1}H NMR of 78 (400 MHz, $CDCl_3$)

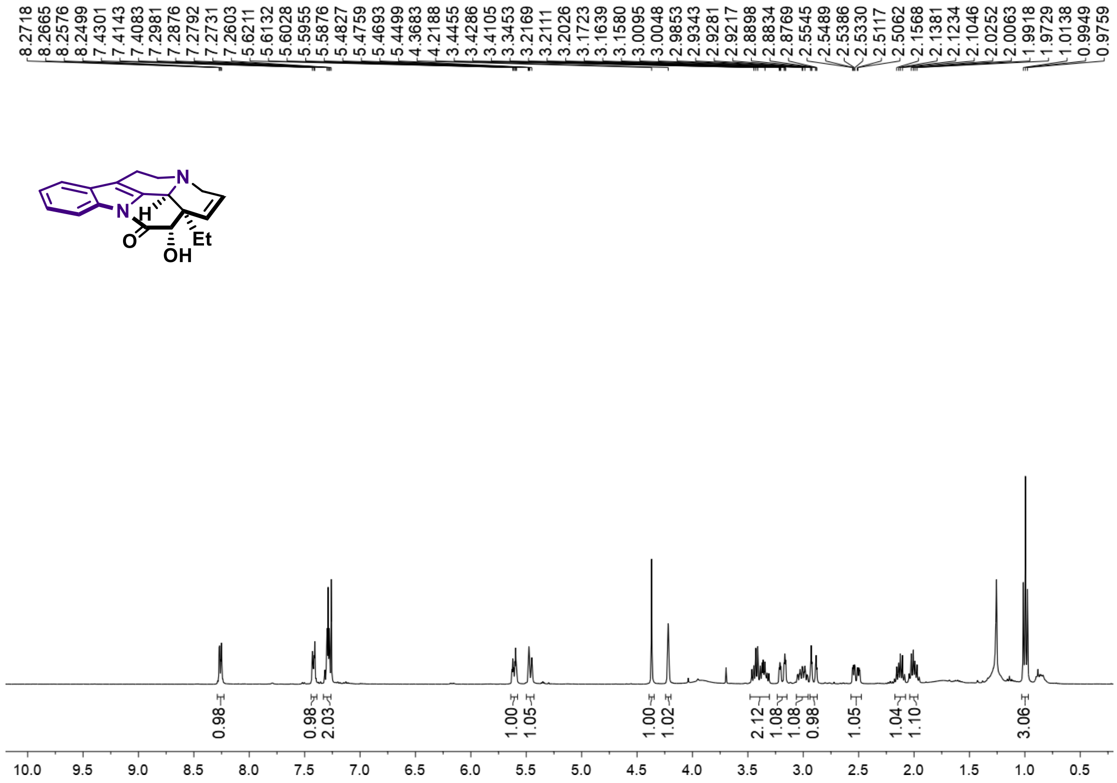

Supplementary Figure 352: ^{13}C NMR of 78 (101 MHz, $CDCl_3$)

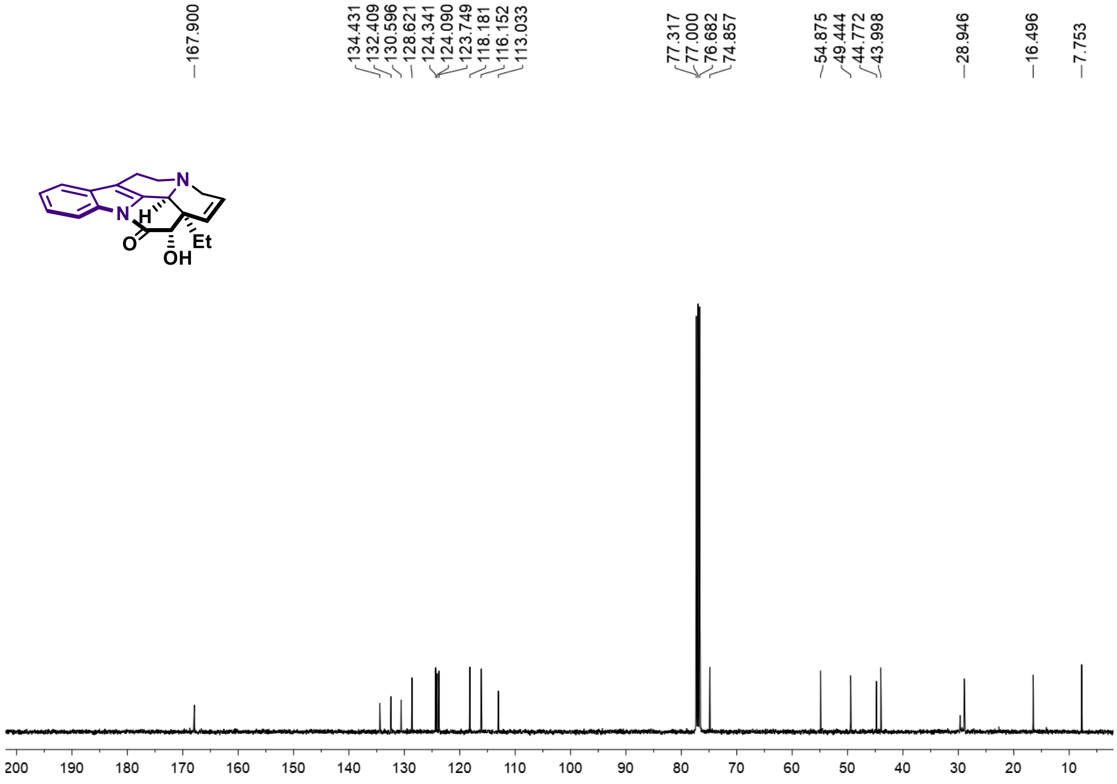

Supplementary Figure 353: ^{1}H NMR of 79 (400 MHz, $CDCl_3$)

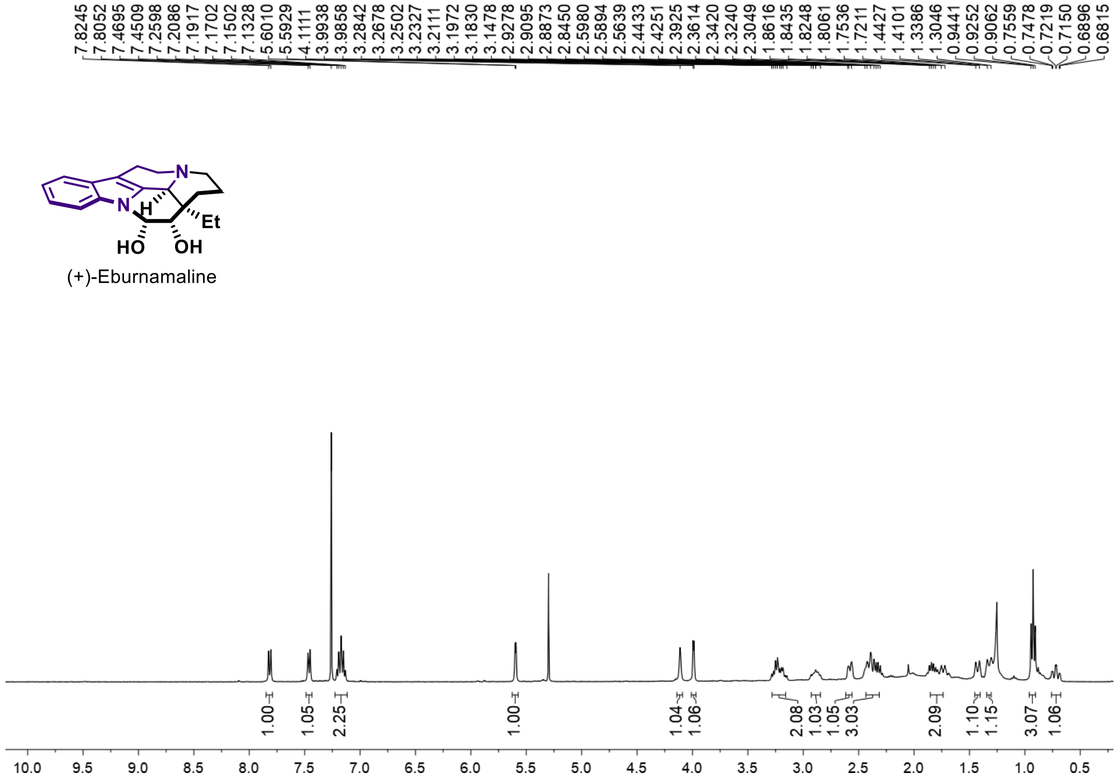

Supplementary Figure 354: ^{13}C NMR of 79 (101 MHz, $CDCl_3$)

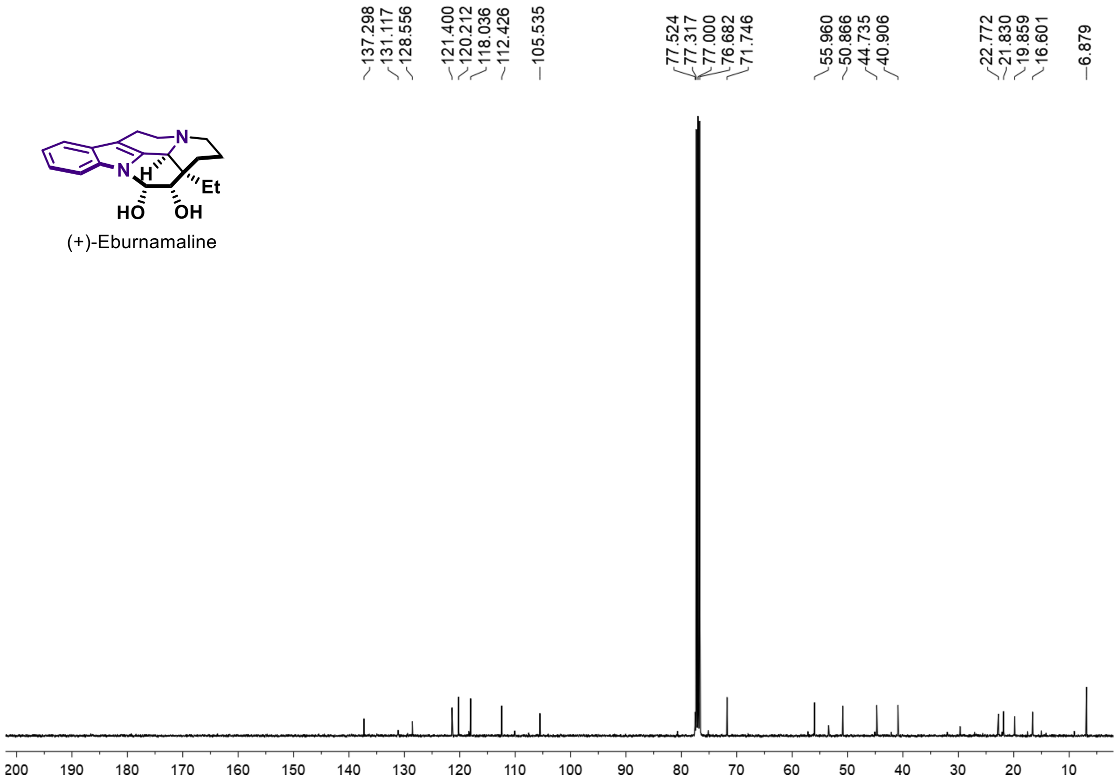

Supplementary Figure 355: ^{1}H NMR of 80 (400 MHz, $CDCl_3$)

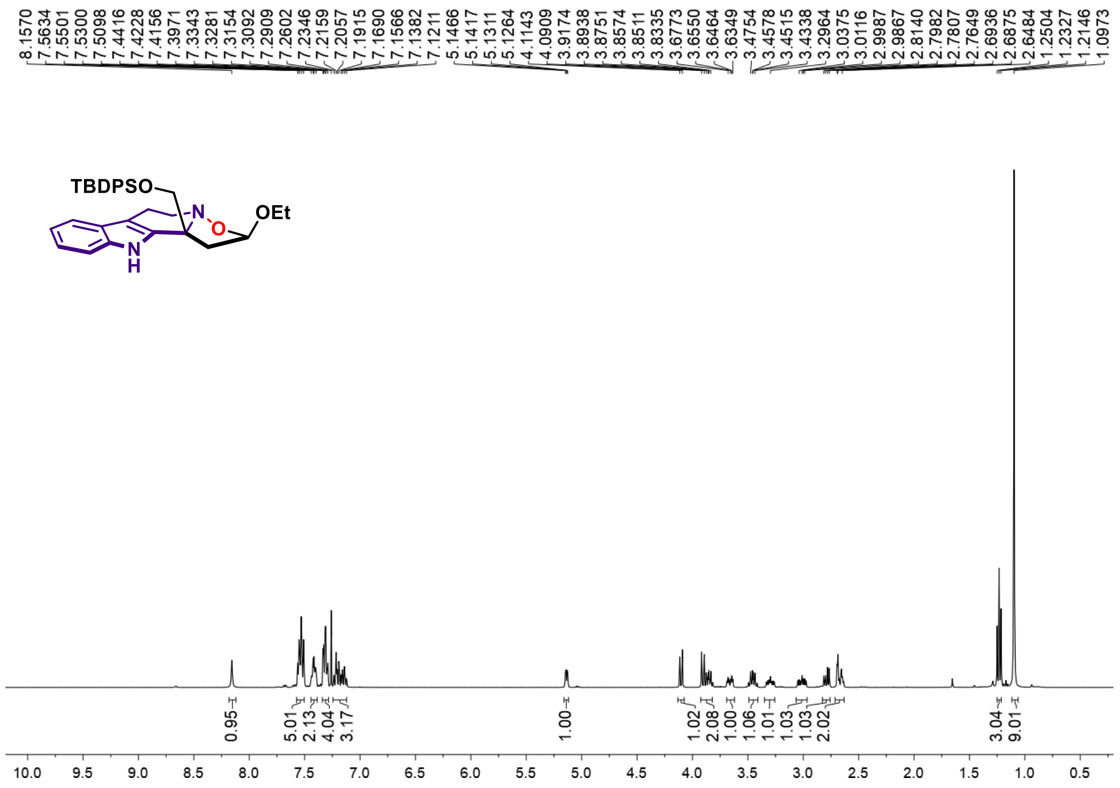

Supplementary Figure 356: ^{13}C NMR of 80 (101 MHz, $CDCl_3$)

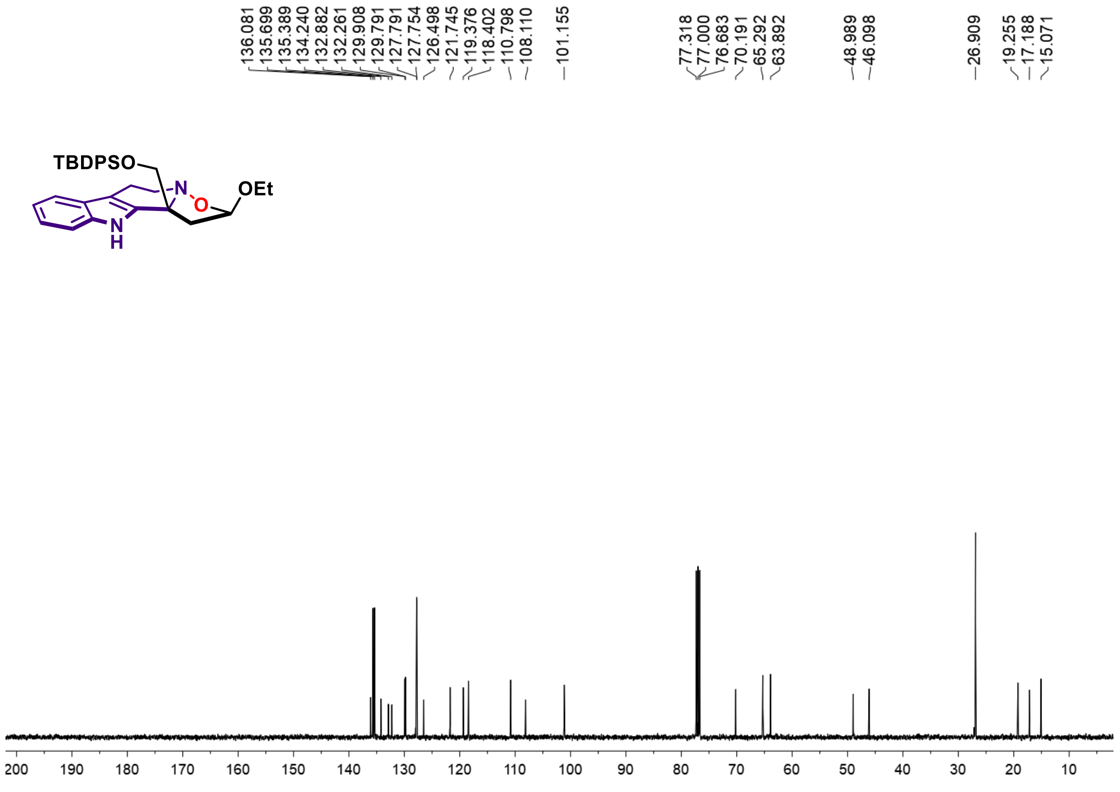

Supplementary Figure 357: ^{1}H NMR of 81 (400 MHz, CDCl$_3$)

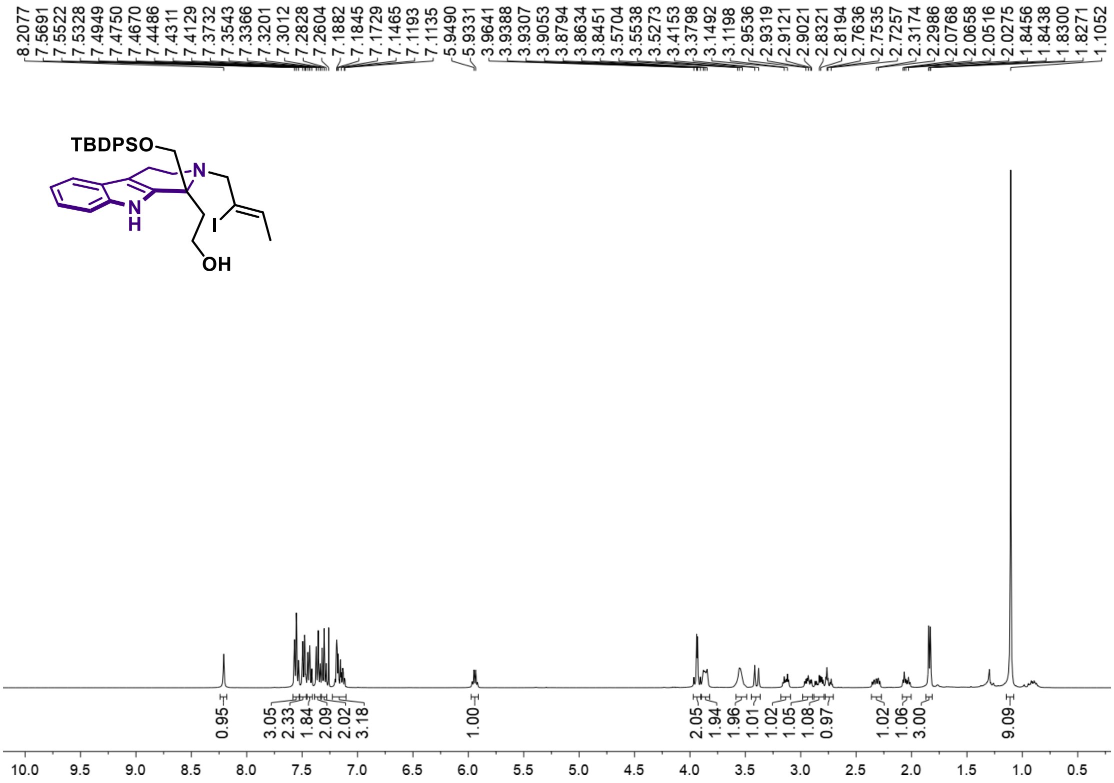

Supplementary Figure 358: ^{13}C NMR of 81 (101 MHz, CDCl$_3$)

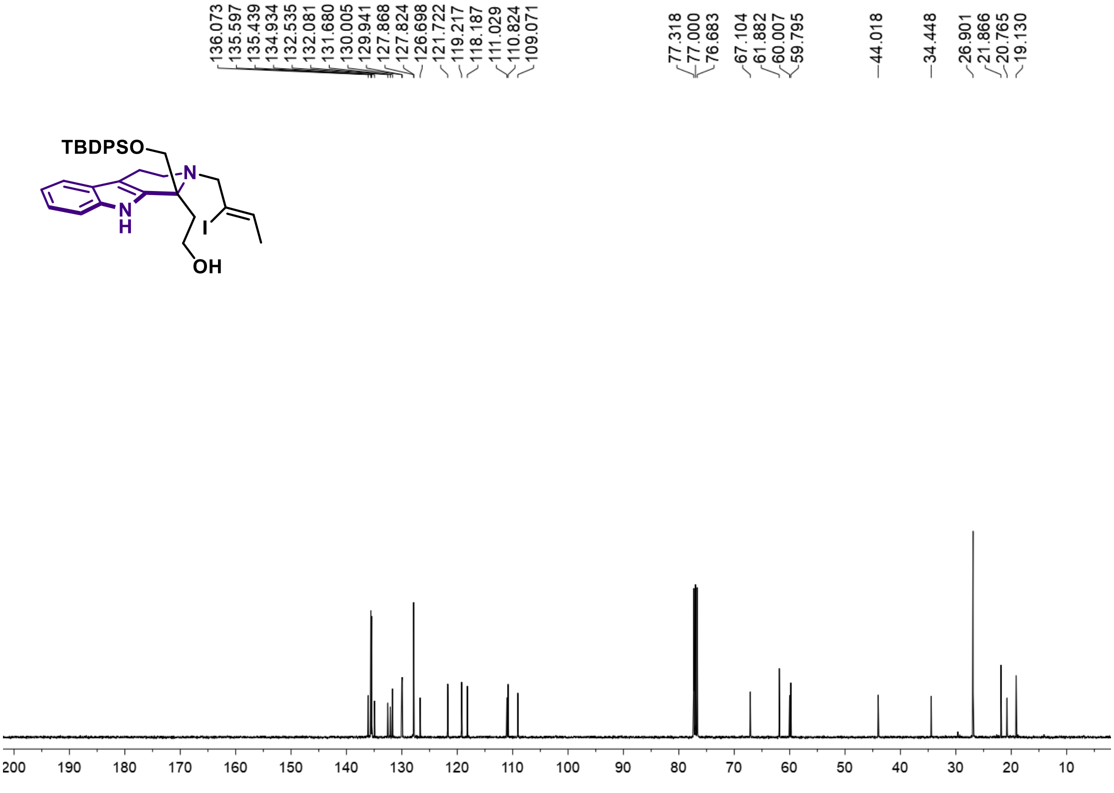

Supplementary Figure 359: ^{1}H NMR of 82 (400 MHz, $CDCl_3$)

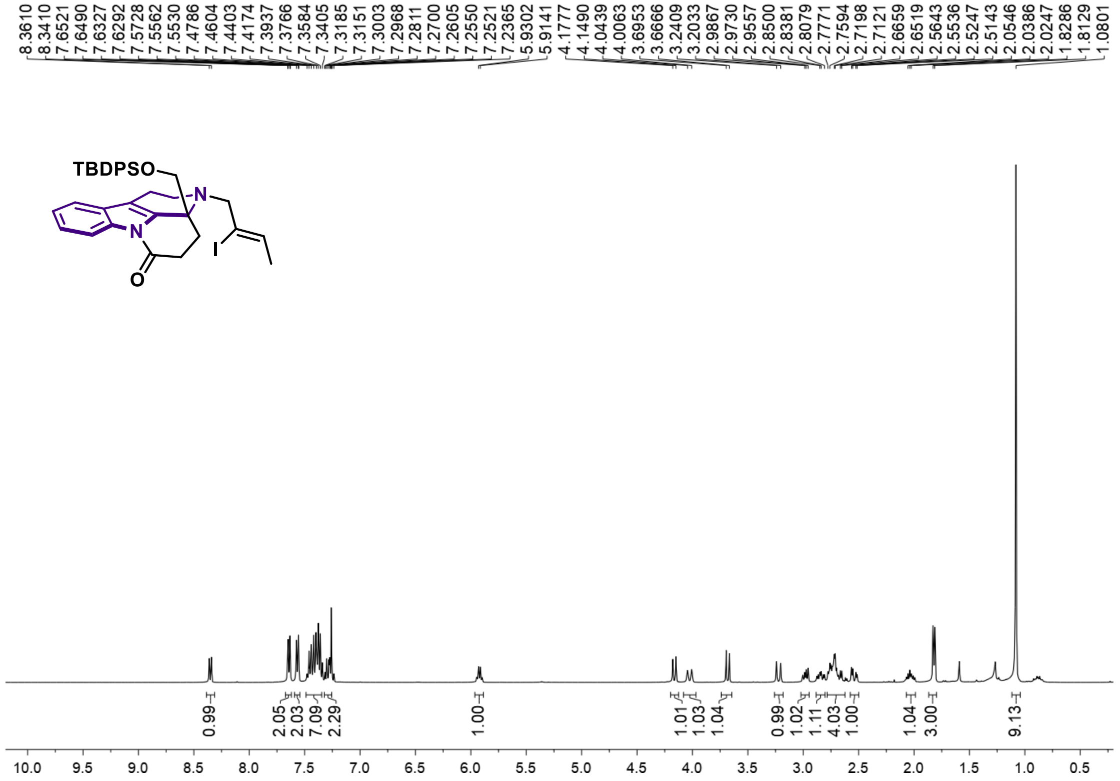

Supplementary Figure 360: ^{13}C NMR of 82 (101 MHz, $CDCl_3$)

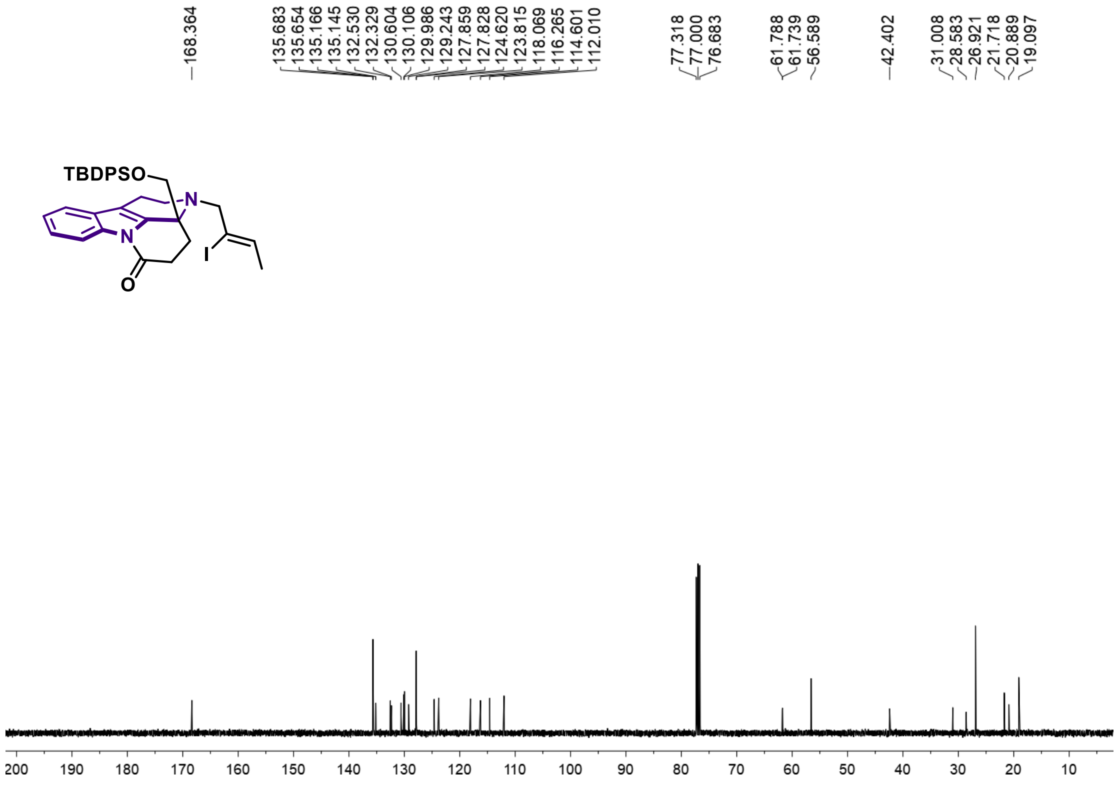

Supplementary Figure 361: ^{1}H NMR of 83 (400 MHz, $CDCl_3$)

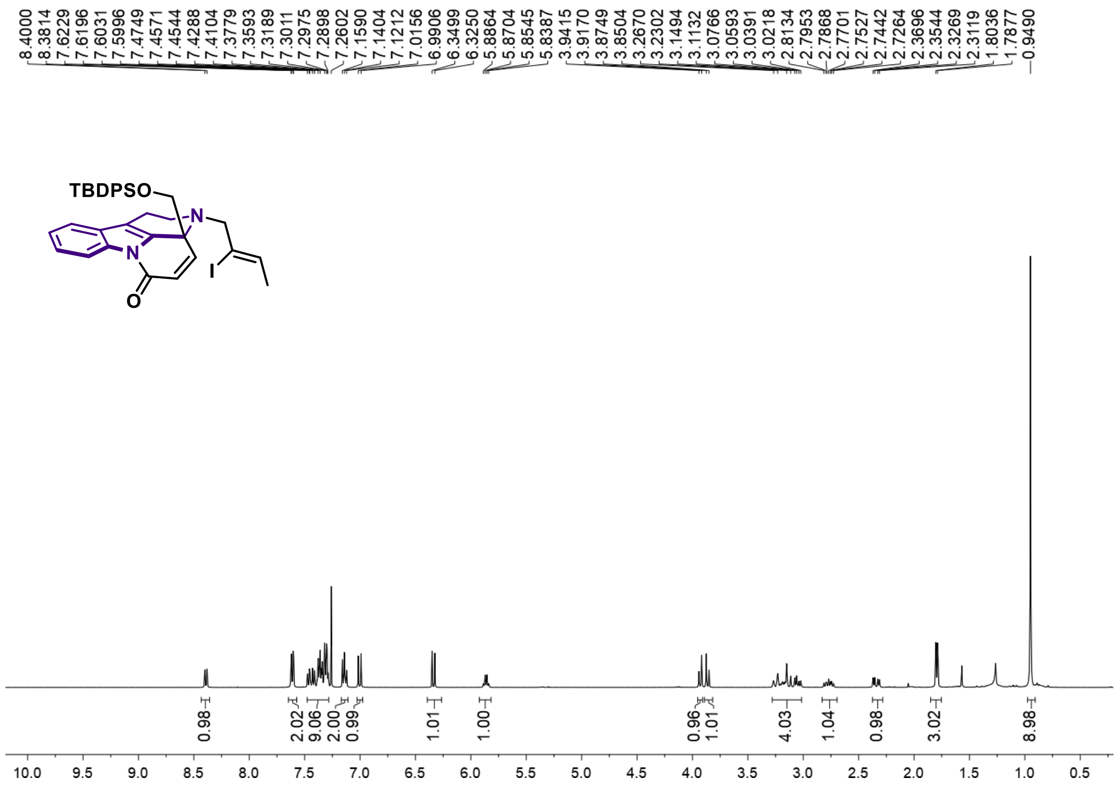

Supplementary Figure 362: ^{13}C NMR of 83 (101 MHz, $CDCl_3$)

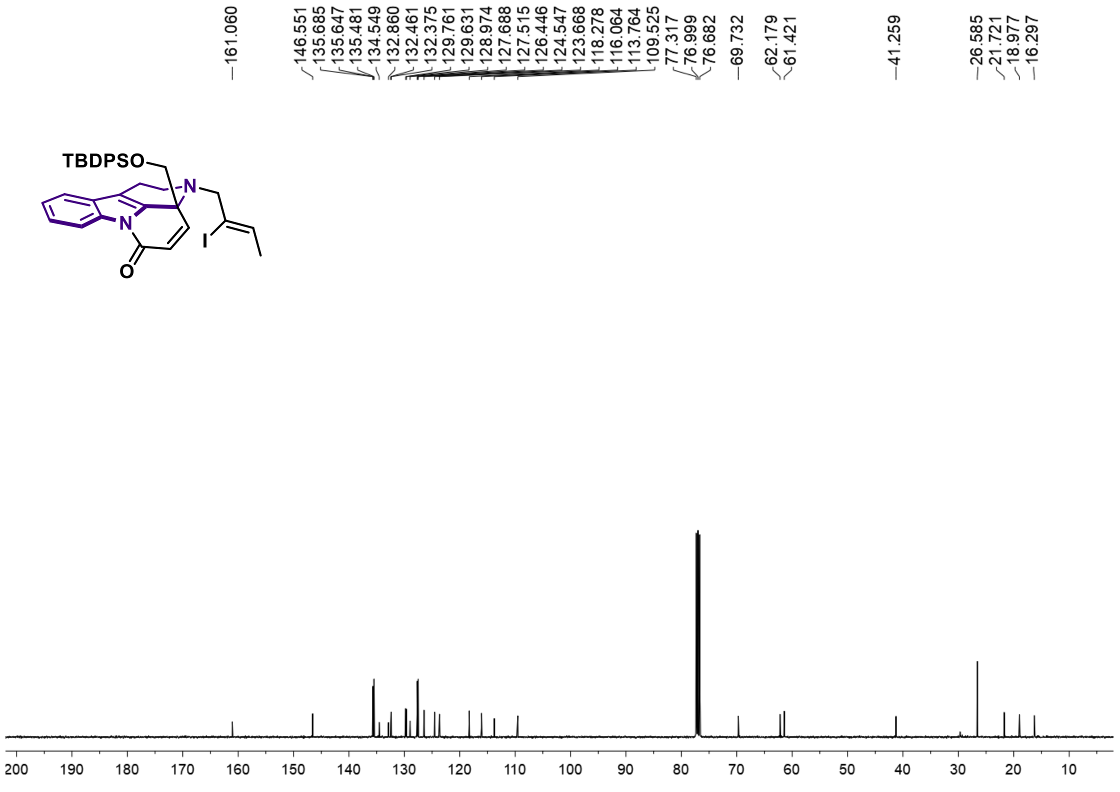

Supplementary Figure 363: ^{1}H NMR of 84 (400 MHz, CDCl$_3$)

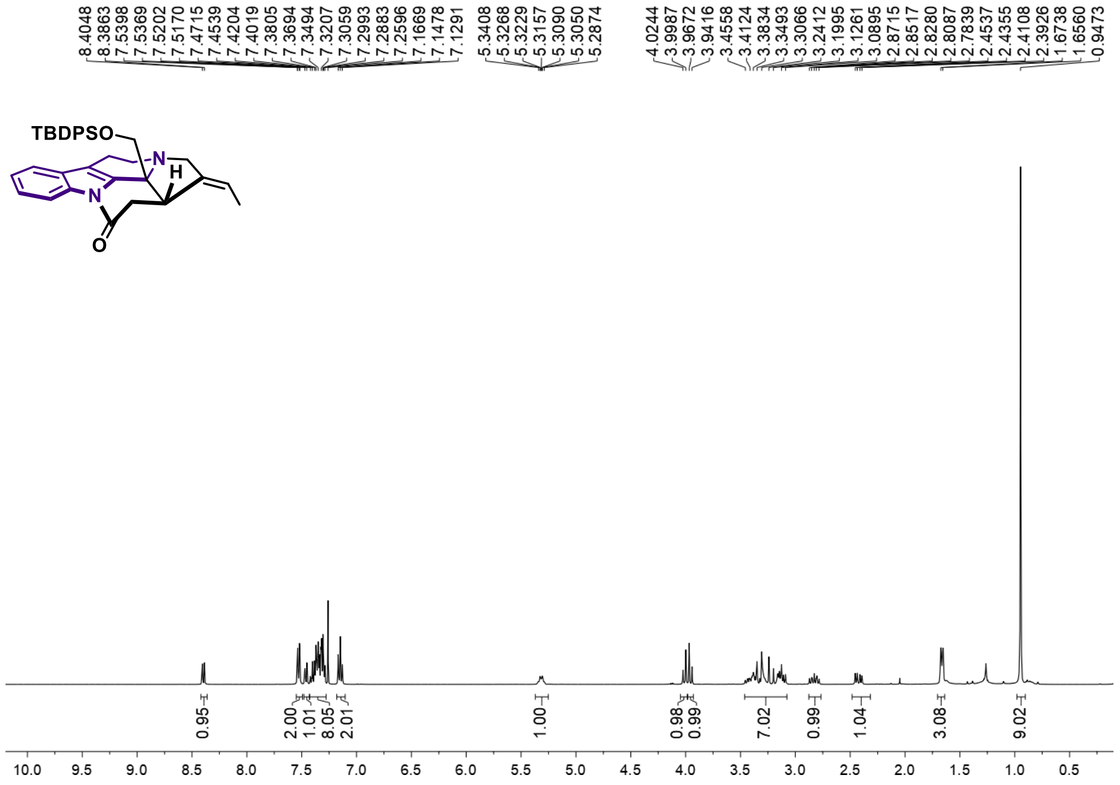

Supplementary Figure 364: ^{13}C NMR of 84 (101 MHz, CDCl$_3$)

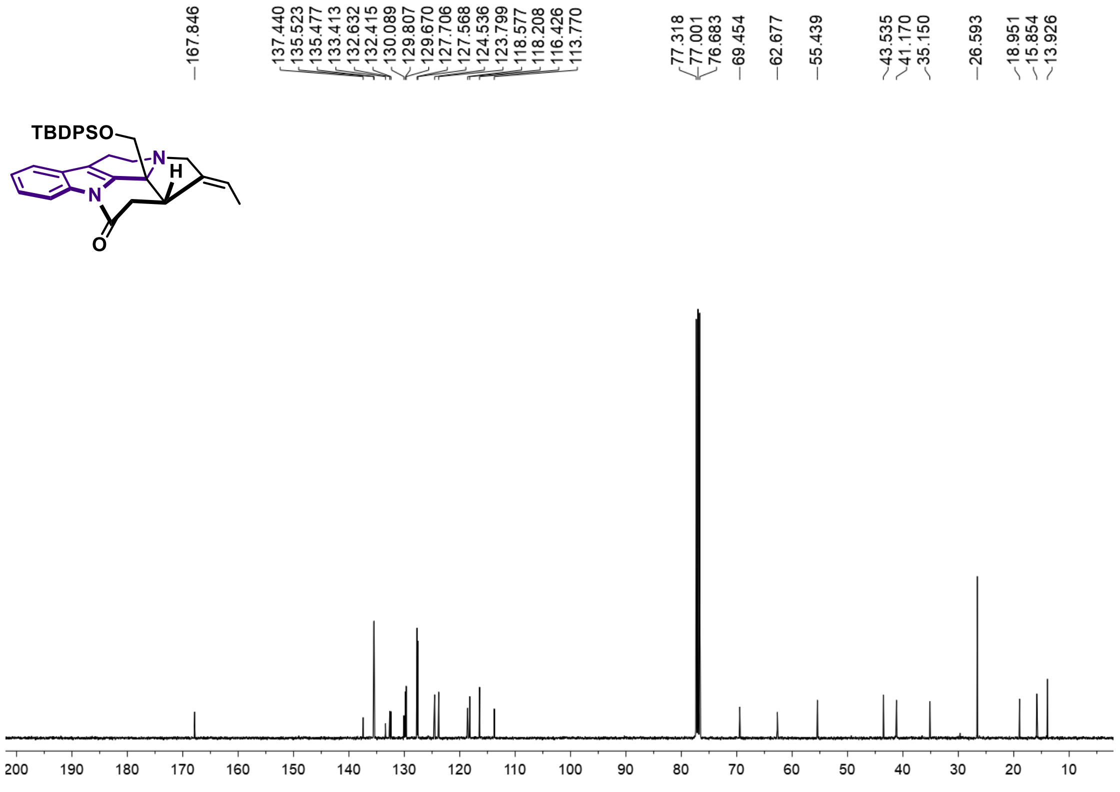

Supplementary Figure 365: ^{1}H NMR of 85 (400 MHz, $CDCl_3$)

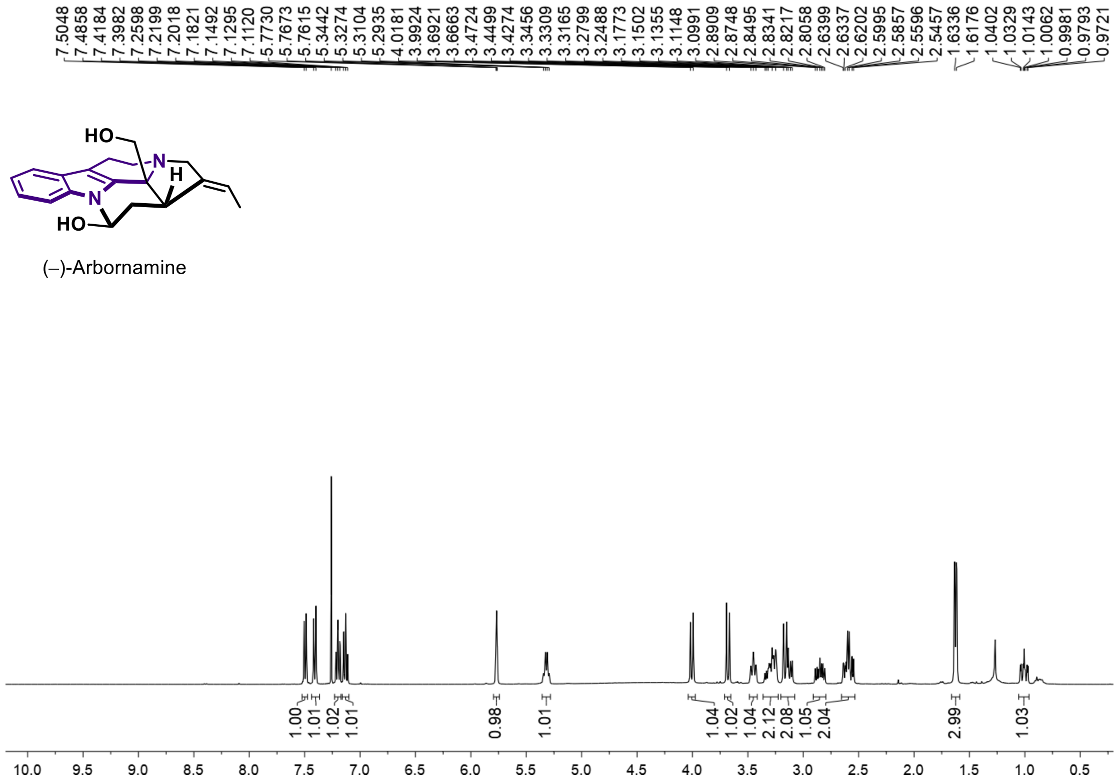

Supplementary Figure 366: ^{13}C NMR of 85 (101 MHz, $CDCl_3$)

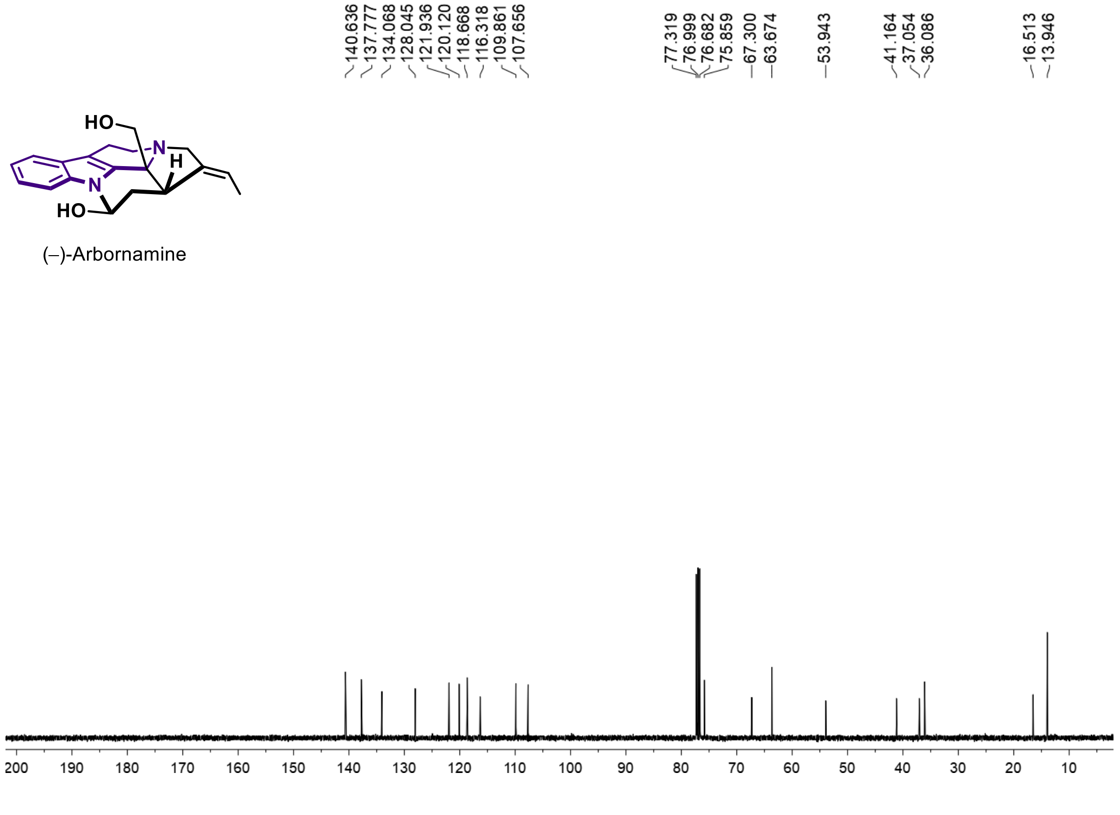

6. Supplementary references

[1] (a) Nelson, H. M., Reisberg, H. S., Shunatona, H. P., Patel, J. S. & Toste, F. D. Chiral anion phase transfer of aryldiazonium cations: an enantioselective synthesis of C3-diazenated pyrroloindolines. *Angew. Chem. Int. Ed.* **53**, 5600-5603 (2014). (b) Jing, D., Lu, C., Chen, Z., Jin, S., Xie, L., Meng, Z., Su, Z. & Zheng, K. Light-driven intramolecular C–N cross-coupling *via* a long-lived photoactive photoisomer complex. *Angew. Chem. Int. Ed.* **58**, 14666-14672 (2019).

[2] Li, Z. & Yu, B. HFIP-Promoted de novo synthesis of biologically relevant nonnatural α-arylated amino esters and dipeptide mimetics. *Chem. Eur. J.* **25**, 16528-16532 (2019).

[3] Fandrick, D. R., Hart, C. A., Okafor, I. S., Mercadante, M. A., Sanyal, S., Masters, J. T., Sarvestani, M., Fandrick, K. R., Stockdill, J. L., Grinberg, N. et al. Copper-catalyzed asymmetric propargylation of cyclic aldimines. *Org. Lett.* **18**, 6192-6195 (2016).

[4] Wang, Y., Zhang, P., Di, X., Dai, Q., Zhang, Z.-M. & Zhang, J. Gold-catalyzed asymmetric intramolecular cyclization of *N*-allenamides for the synthesis of chiral tetrahydrocarbolines. *Angew. Chem. Int. Ed.* **56**, 15905-15909 (2017).

[5] Wanner, M. J., Boots, R. N. A., Eradus, B., de Gelder, R., van Maarseveen, J. H. & Hiemstra, H. Organocatalytic enantioselective total synthesis of (−)-arboricine. *Org. Lett.* **11**, 2579-2581 (2009).

[6] Wang, X., Xia, D., Qin, W., Zhou, R., Zhou, X., Zhou, Q., Liu, W., Dai, X., Wang, H., Wang, S. et al. A radical cascade enabling collective syntheses of natural products. *Chem* **2**, 803-816 (2017).

[7] Shellard, E. J., Tantivatana, P. & Beckett, A. H. The *mitragyna* species of Asia. Planta Med. **15**, 366-370 (1967).

[8] Villa, R. A., Xu, Q. & Kwon, O. Total synthesis of (±)-hirsutine: application of phosphine-catalyzed imine-allene [4+2] annulation. *Org. Lett.* **14**, 4634-4637 (2012).

[9] Liang, L., Zhou, S., Zhang, W. & Tong, R. Catalytic asymmetric alkynylation of 3,4-dihydro-β-carbolinium ions enables collective total syntheses of indole alkaloids. *Angew. Chem. Int. Ed.* **60**, 25135-25142 (2021).

[10] Liu, Y., Wang, Q., Zhang, Y., Huang, J., Nie, L., Chen, J., Cao, W. & Wu, X. Enantioselective synthesis of indoloquinolizidines via asymmetric catalytic hydrogenation/lactamization of imino diesters. *J. Org. Chem.* **78**, 12009-12017. (2013).

[11] Weniger, B. & Anton, R. Indole alkaloids from Antirhea Portoricenszs. *J. Nat. Prod.* **57**, 287-290 (1994).

[12] Miller, E. R., Hovey, M. T. & Scheidt, K. A. A concise, enantioselective approach for the synthesis of yohimbine alkaloids. *J. Am. Chem. Soc.* **142**, 2187-2192 (2020).

[13] Sheth, V. M., Hong, B. C. & Lee, G. H. Enantioselective total synthesis of (+)-arborescidine C and related tetracyclic indole alkaloids using organocatalysis. *Org. Biomol. Chem*. **15**, 3408-3412 (2017).

[14] Santos, L. S., Pilli, R. A. & Rawa, V. H. Enantioselective total syntheses of (+)-arborescidine A, (212)-arborescidine B, and (−)-arborescidine C. *J. Org. Chem*. **69**, 1283-1289 (2004).

[15] Andriamialisoa, R. Z., Langlois, N., Langlois, Y., Gillet, B. & Beloeil, J. C. (±) Dehydro-14,15 nor-17 eburnamonine et (±) dihydro-2,7 nor-17 eburnamonine. *Tetrahedron* **44**, 1953-1958 (1988).

[16] Nemes, A., Szántay Jr., C., Czibula, L. & Greiner, I. Synthesis of 18-hydroxyvincamines and epoxy-1,14-secovincamines; a new proof for the aspidospermane-eburnane rearrangement. *Heterocycles*, **71**, 2347-2362 (2007).

[17] Goh, S. H., Ali, A. R. M. & Wong, W. H. Alkaloids of leuconotis griffithii and L. Eugenifolia (Apocynaceae). *Tetrahedron* **45**, 7899-7920 (1989).

[18] Lancefield, C. S., Zhou, L., Lébl, T., Slawin, A. M. Z. & Westwood, N. J. The synthesis of melohenine B and a related natural product. *Org. Lett.* **14**, 6166-6169 (2012).
[19] Arambewela, L. S. R. & Khoung-Huu, F. Indole alkaloids from *Hunteria Zeylanzca*. *Phytochemistry*, **20**, 349-350 (1981).
[20] Gan, C.-Y., Low, Y.-Y., Etoh, T., Hayashi, M., Komiyama, K., Kam, T.-S. Leuconicines A-G and (−)-eburnamaline, biologically active strychnan and eburnan alkaloids from Leuconotis. *J. Nat. Prod.* **72**, 2098-2103 (2009).
[21] Wong, S.-P., Chong, K.-W., Lim, K.-H., Lim, S.-H., Low, Y.-Y. & Kam, T.-S. Arborisidine and arbornamine, two monoterpenoid indole alkaloids with new polycyclic carbon-nitrogen skeletons derived from a common pericine precursor. *Org. Lett.* **18**, 1618-1621 (2016).
